# Regulation of microglia related neuroinflammation contributes to the protective effect of Gelsevirine on ischemic stroke

**DOI:** 10.3389/fimmu.2023.1164278

**Published:** 2023-03-30

**Authors:** Chunlei Xing, Juan Lv, Zhihui Zhu, Wei Cong, Huihui Bian, Chenxi Zhang, Ruxin Gu, Dagui Chen, Xiying Tan, Li Su, Yu Zhang

**Affiliations:** ^1^ Institute of Translational Medicine, Shanghai University, Shanghai, China; ^2^ School of Pharmacy, Nanjing Medical University, Nanjing, China; ^3^ Department of Geriatric Neurology, The Affiliated Brain Hospital of Nanjing Medical University, Nanjing, China; ^4^ Department of Pharmacy, Affiliated Hospital of Nanjing University of Chinese Medicine, Nanjing, China

**Keywords:** Gelsevirine, ischemic stroke, neuroinflammation, JAK2-STAT3, microglia

## Abstract

Stroke, especially ischemic stroke, is an important cause of neurological morbidity and mortality worldwide. Growing evidence suggests that the immune system plays an intricate function in the pathophysiology of stroke. Gelsevirine (Gs), an alkaloid from *Gelsemium elegans*, has been proven to decrease inflammation and neuralgia in osteoarthritis previously, but its role in stroke is unknown. In this study, the middle cerebral artery occlusion (MCAO) mice model was used to evaluate the protective effect of Gs on stroke, and the administration of Gs significantly improved infarct volume, Bederson score, neurobiological function, apoptosis of neurons, and inflammation state *in vivo*. According to the data *in vivo* and the conditioned medium (CM) stimulated model *in vitro*, the beneficial effect of Gs came from the downregulation of the over-activity of microglia, such as the generation of inflammatory factors, dysfunction of mitochondria, production of ROS and so on. By RNA-seq analysis and Western-blot analysis, the JAK-STAT signal pathway plays a critical role in the anti-inflammatory effect of Gs. According to the results of molecular docking, inhibition assay, and thermal shift assay, the binding of Gs on JAK2 inhibited the activity of JAK2 which inhibited the over-activity of JAK2 and downregulated the phosphorylation of STAT3. Over-expression of a gain-of-function STAT3 mutation (K392R) abolished the beneficial effects of Gs. So, the downregulation of JAK2-STAT3 signaling pathway by Gs contributed to its anti-inflammatory effect on microglia in stroke. Our study revealed that Gs was benefit to stroke treatment by decreasing neuroinflammation in stroke as a potential drug candidate regulating the JAK2-STAT3 signal pathway.

## 1 Introduction

Stroke, a primary cause of severe disability, has become the second leading cause of death worldwide, and ischemic stroke constitutes 75–80% of all strokes (1, 2). In the clinic, the therapies restoring blood flow, such as intravenous thrombolysis and thrombectomy, benefit patients in acute ischemic stroke (1, 3). Nevertheless, progressive neuronal degeneration and loss of function are still challenging to solve because of the lack of effective drugs (4–6). Therefore, it needs to develop new approaches and discover new reagents for stroke.

Neuroinflammation, a specific event in stroke, is tightly related to the pathophysiological process during stroke, enhancing neuron death and dysfunction (2, 7, 8). Especially in the acute phase, multiple components are released from the ischemic core, and they can increase astrocyte and microglial activation as danger signals (9, 10). In the progress of neuroinflammation in stroke, microglia, resident immune cells in the brain, plays an essential role in this pathophysiological process. In the acute phase, microglia are often over-activated, and they mainly transform into an M1 phenotype which is the pro-inflammatory type and secretes pro-inflammation factors, such as interleukin-1β (IL-1β), interleukin-6 (IL-6), and tumor necrosis factor (TNF) promoting inflammation. Over-activated microglia also secrete other neurotoxic substances aggravating brain damage (11, 12).

*Gelsemium elegans* is a traditional Chinese medicine widely used to treat neuralgia, sciatica, rheumatoid arthritis, and acute pain (13, 14). Although multiple compounds are involved in the beneficial effect of *Gelsemium elegans*, alkaloidal constituents are found to play critical pharmaceutical roles in *Gelsemium elegans*, including anxiolytic, antitumor, antistress, antipsoriatic, and analgesic activities (15, 16). In these pharmaceutical roles, anti-inflammatory is a typical character of alkaloidal constituents of *Gelsemium elegans*. Some alkaloidal constituents are reported to alleviate neuroinflammation, which benefits cognitive function and neuropathic pain (17–19). Gelsevirine (Gs), an alkaloid from *Gelsemium elegans*, has a novel hexacyclic cage structure and a favorable safety profile. Gs improves age-related and surgically induced osteoarthritis in mice by reducing local inflammation (20). The potential ability to decrease inflammation makes Gs possible to be used in treating other inflammation-related diseases in the central nervous system, such as neurodegenerative diseases and stroke.

In the present research, we investigated the protective effects of Gs on stroke and potential mechanisms. Our study revealed that Gs decreased neuroinflammation and protected mice from stroke through the JAK2-STAT3 signal. It provided a potential regent for the treatment of stroke.

## 2 Materials and methods

### 2.1 Animals

Male C57BL/6 mice, about 8-9 weeks of age, were purchased from Changzhou Cavens Company. The mice were fed standard pellet diet with sterilized tap water for 12 h in black and white light and were free to move around. C57BL/6 mice were maintained according to the Animals (Scientific Procedures) Act, 1986 of the UK Parliament, Directive 2010/63/EU of the European Parliament and the Guide for the Care and Use of Laboratory Animals published by the US National Institutes of Health (NIH Publication No. 85–23, revised 1996). Animal studies were approved by Ethics Committee of Shanghai University (Approval NO. ECSHU2021-167) and reported in compliance with the ARRIVE guidelines.

### 2.2 Transmit middle cerebral artery occlusion (tMCAO)

Model group, low dose group, high dose group of preparation on the right side of the tMCAO model, mice were anaesthetized with 2% isoflurane in an air mixture before the MCAO. The isoflurane concentration was maintained at 1.4% isoflurane in an air mixture in the progress of MCAO. The supine position is fixed, the mice carotid midline incision, the separation of the right common carotid artery, external carotid artery and internal carotid artery, then in the common carotid artery, distal external carotid artery ligation, the clamp of artery, internal carotid artery (21). A small incision was cut in the free segment of the external carotid artery and the threaded plug was inserted. The thread plug was inserted into the cranium to the middle cerebral artery through the internal carotid artery at the branch of the common carotid artery. The insertion depth of the threaded plug was (18.5 + 0.5) mm. The model of the sham operation group was prepared until the right common carotid artery, external carotid artery and internal carotid artery were exposed and then sutured (22). Following reperfusion, mice were sacrificed for research at the indicated time. A homoeothermic heating blanket was used to maintain core body temperature in the mice at 37°C during ischemia/reperfusion (tMCAO) operation (21).

### 2.3 Neurological symptom scoring

After 24 h of cerebral ischemia-reperfusion, mice were scored for neurobehavioral deficits by an observer unaware of the grouping according to the Longa method five-point scale (23).

0 points: no signs of neurological deficits.

1 point: inability to fully extend the contralateral forelimb.

2 points: rotation to the contralateral side when walking, “tail-chasing” phenomenon.

3 points: unstable standing, leaning to the opposite side.

4 points: inability to walk spontaneously and impaired consciousness. Random evaluation test within the specified time, a score greater than 1 means that the tMCAO model is successfully established.

### 2.4 Rotarod test

The fixed-speed rotarod was used to test neurological deficits in mice (Dunham and Miya, 1957). In the present test, animals were briefly pre-trained at a fixed speed (10 rpm). For the test, mice were placed on the rod at 40 rpm for a maximum of 300 s. The animals were tested two times with a rest of 60 min between each test (24).

### 2.5 Measurement of cerebral infarct volume

After 24 h of cerebral ischemia-reperfusion, the brains of mice were removed from the heads, placed in the refrigerator (-20°C) for several minutes, the olfactory bulb, cerebellum, and low brainstem were removed, and coronal cuts were made in 4 cuts into 5 slices (2 mm), with the first cut at the midpoint of the line connecting the anterior pole of the brain and the optic cross, the second at the optic cross, the third at the funicular stalk, and the fourth between the funicular stalk and the caudal pole. Brain slices were stained with red tetrazolium (TTC), and the staining solution consisted of 1.5 mL 1% TTC, 0.1 mL 1 mol/L K_2_HPO_4_, 3.4 mL saline, and stained for 20 min at 37°C protected from light, with normal tissue being red and infarcted tissue being white. 4% formaldehyde was fixed for two days, and then the infarcted brain tissue was removed by absorbing the liquid with filter paper, and the weight of the infarcted brain tissue as a percentage of the total brain weight was used as the percentage of infarcted brain tissue to total brain weight was used as an indicator of brain infarct volume (23).

### 2.6 Brain slice preparation

Animals were deeply anesthetized with sodium pentobarbital (50 mg/kg, i.p.) and underwent sternotomy, followed by intracardiac perfusion with 200 mL saline and 200 mL 4% ice-cold paraformaldehyde in 0.1 M phosphate-buffered saline. The brain was removed, post-fixed in 4% paraformaldehyde for 4 h, and subsequently allowed to equilibrate in 30% sucrose in phosphate-buffered saline overnight at 4°C.

### 2.7 Immunofluorescence

Mice were anesthetized with isoflurane and perfused intracardially with saline followed by paraformaldehyde (PFA, 4%) in phosphate buffer (pH 7.4). The brain was removed, post-fixed with PFA overnight, cryoprotected in 30% sucrose, frozen, and 14-μm-thick sections were obtained in a cryostat. The sections were fixed with ethanol, blocked with normal serum and incubated overnight at 4°C with combinations of primary antibodies. Then, sections were incubated for 2 h at room temperature with secondary antibodies (Alexa Fluor-488, -546, -647, LifeTechnologies). Immunoreaction controls were always carried out by omission of the primary antibodies. Sections were counterstained with either 4’6-diamidino-2-phenylindole (DAPI) to visualize the cell nuclei and they were observed under a confocal laser microscope (Leica, SP5 or TCS SPE) (25).

### 2.8 Western blot

After 24 h of cerebral ischemia-reperfusion, mice were anaesthetized with 2% isoflurane in an air mixture and sacrificed, and the brains were removed and placed on ice. We used the scraping method to collect the treated cells, which were also placed on ice. The cells were fully lysed with a whole protein extraction kit. After centrifugation and separation, the supernatant was absorbed and the protein concentration was determined according to the BCA Protein Concentration Assay Kit to adjust each sample to equal amounts of protein. The samples were then denatured by adding the corresponding reducing loading buffer and cooking for 10 min in an ALLSHENG dry thermostat. Denatured proteins were separated by 10% SDS-PAGE gel electrophoresis and then transferred to PVDF membranes. To prevent non-specific protein binding sites from binding to antibodies, PVDF membranes were closed in TBS containing 0.1% Tween20 and 5% BSA for 90 min. PVDF membranes were cut at the corresponding molecular weights and incubated with rabbit anti-PARP-1 (1:1000), cleaved-caspase 9 (1:1000), Bcl-2 (1:1000), Bax (1:1000) and cleaved-caspase 3 (1:1000), respectively, overnight at 4°Cin the refrigerator. The PVDF membranes were incubated with the corresponding secondary antibodies for 2h at room temperature, then exposed to ultrasensitive ECL chemiluminescent reagents and visualized by Bio-Rad automated gel imaging system. Finally, the bands were analyzed with Image for grayscale values to evaluate the relative expression levels of the proteins (26).

### 2.9 Oxygen glucose deprivation (OGD)

Briefly, HT22 cells were inoculated in 6-well plates at a density of 1x10^6^ per well overnight and the culture medium was changed into glucose-free DMEM and washed with 1 × PBS three times. Cultures were then transferred to an incubator containing 5% CO_2_ and 95% N_2_ at 37°C for 4 h. Then, the culture medium was changed into complete medium and cells were cultured at 37°C in a humidified 5% CO_2_ incubator for 20 h and the medium were collected as conditioned medium (CM) for the stimulation of BV2 cells (27).

### 2.10 The administration of LPS, CM and Gs on BV2 cells

Briefly, BV2 cells were inoculated in 6-well plates at a density of 1×10^6^ per well or 96-well plates at a density of 5000 per well overnight. The culture medium was changed into fresh complete medium with 50 ng/mL LPS or mixed medium (1:1, fresh complete medium: CM). Meanwhile, Gs was administrated with different doses. Then, cultures were then transferred to an incubator containing 5% CO_2_ and 95% air at 37°C for 8 h.

### 2.11 Quantitative analysis of cytokine mRNA expression

mRNA transcription of cytokines was analyzed by quantitative reverse-transcription polymerase chain reaction (qRT-PCR). Using the Hybrid-R™ (GeneAll, Seoul, Korea), we extracted total RNA from the BV2 microglial cells, and measured the concentration using a NanoDrop ND-2000 spectrophotometer (Thermo Fisher Scientific Inc., Waltham, MA, USA). Next, 3 μg RNA samples were converted to cDNA using TOPscript™ RT DryMIX. The cDNA was analyzed by qRT-PCR using TOPreal™ qPCR 2× PreMIX (SYBR Green; Enzynomics) and the CFX Connect Real-Time PCR System (Bio-Rad Laboratories, CA, USA). Primers, synthesized at COSMO Genetech (Seoul, Korea), were as follows; iNOS: forward, 5’-GTGTTC TTTGCTTCCATG CT-3’, reverse, 5’-AGTTGCTCCTCTTCCAAG GT-3’; TNF-α: forward, 5’- GAGTGACAAGCCTGTAGCCCA-3’, reverse, 5’- AGCTCCACGCCATTGGC-3’; IL-1β: forward, 5’-CCCAAGCAATACCCA AAG AA-3’, reverse, 5’-GCT TGTGCTCTGCTTGTGAG-3’; IL-10: forward, 5’-CTAGAGCTGCGGACTGCCTTC-3’, reverse, 5’-TTGATTTCTGGGCCATGC-3’; COX2: forward, 5’-TCATTG GTGGAGAGGTGTAT-3’, reverse, 5’-ACCCCACTCAGGATGCTCCT-3’; GAPDH: forward, 5’-TGA ATACGGCTACAGCAACA-3’, reverse, 5’- AGGCCCCTCCTGTTATTATG-3’.IL-6: forward, 5’-TAGTCCTTCCTACCCCAATTTCC-3’, reverse, 5’- TTGGTCCTTAGCCACTCCTTC-3’; IFN-γ: forward, 5’- TGTTACTGCCACGGCACAGT-3’, reverse, 5’- CTGGCTCTGCAGGATTtpTTCAT -3’.

### 2.12 Recombinant JAK2 inhibition assay

50 ng of recombinant JAK2 protein was incubated with 100 μM of the substrate (poly Glu : Tyr) in the presence of increasing amounts of Gs. After a 30 minutes incubation at 37°C, ATP levels were measured with ATP assay kit (Nanjing Jiancheng Bioengineering Institute, A095-1-1). The samples treated with vehicle were defined as having 0% inhibition, while the samples without substrate were defined as having 100% inhibition. CEP-33779 was used as a positive inhibitor of JAK2. Each point was assayed in duplicate (N = 2) and is expressed as the mean ± SD (28).

### 2.13 Statistical analysis

Results are presented as mean ± SD. Comparison among groups was analyzed using a two-way ANOVA followed by Bonferroni t-test or one-way ANOVA followed by Tukey’s *post hoc* analysis. Statistical analyses were done using statistical software of GraphPad Prism (version 7.0, GraphPad Software, San Diego, CA, USA), and a P value < 0.05 was considered significant statistically.

## 3 Results

### 3.1 Gelsevirine has an anti-inflammatory effect on microglia *in vitro* models

**Figure 1:**
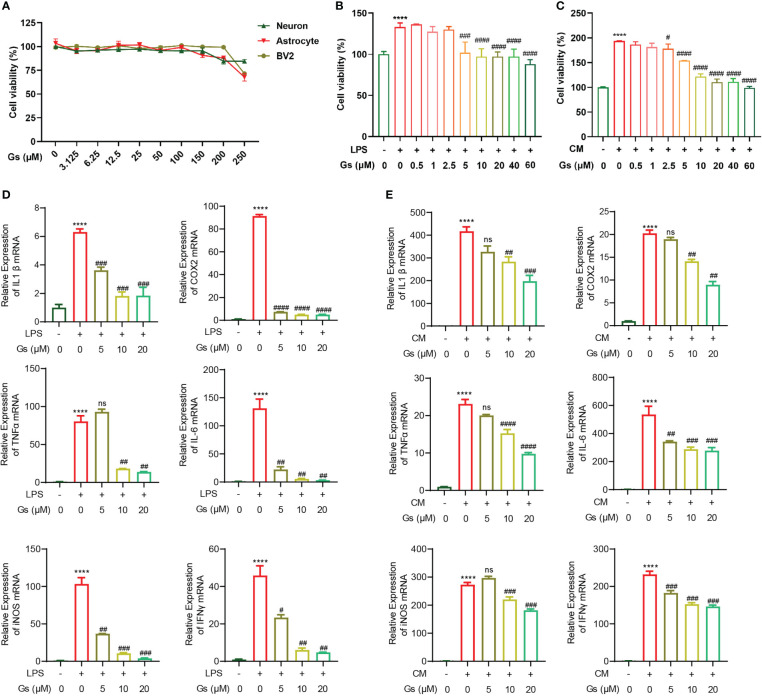
Gelsevirine decreased inflammatory activities of microglia with limited toxicity to major types of cells from brain *in vitro*. **(A)** Gelsevirine did not decrease the viability of primary neuron, astrocyte and BV2 *in vitro*. **(B, C)** Gelsevirine downregulated cell proliferation of BV2 cells induced by LPS or OGD neuron-conditioned medium (named CM). **(D, E)** Gelsevirine decreased the level of inflammatory facts upregulated by LPS or CM. (****, compared with untreated group, P < 0.0001; ####, compared with LPS or CM treated group, P < 0.0001; ###, compared with LPS or CM treated group, P < 0.001; ##, compared with LPS or CM treated group; #, compared with LPS or CM treated group, P < 0.05; ns, compared with CM or LPS treated group, P > 0.05; n = 3).

To evaluate the protective effect of Gelsevirine (Gs) on stroke, the toxicity of Gs on brain cells was first evaluated. By CCK8 assay, Gs showed no significant influence on cell viability of primary neurons, astrocytes and BV2 as high as 100 μM *in vitro* (**Figure 1A**), which indicated the administration of Gs had no toxicity on major types of brain cells. Microglia, the major resident immune cells in the brain, are critical participants in the acute phase of stroke and brain injuries. As our previous report, Gs inhibited the inflammatory response of osteoclasts and injury, both of which are related to the downregulation of the activity of macrophage or macrophage-like cells just like microglia (20). Gs significantly decreased the proliferation of BV2 after the administration of LPS (100ng/mL) or OGD neuron-conditioned medium (named CM) which contributes to the inhibition of inflammation (**Figures 1B, C**). Gs also downregulated the production of pro-inflammatory cytokines in BV2 stimulated by the LPS or CM *in vitro* (**Figures 1D, E**).

These results confirmed that Gs has an anti-inflammatory effect on microglia with less cytotoxicity.

### 3.2 Gelsevirine decreased infarct volumes and improved neurological functions in ischemia/reperfusion mice (tMCAO mice)

**Figure 2:**
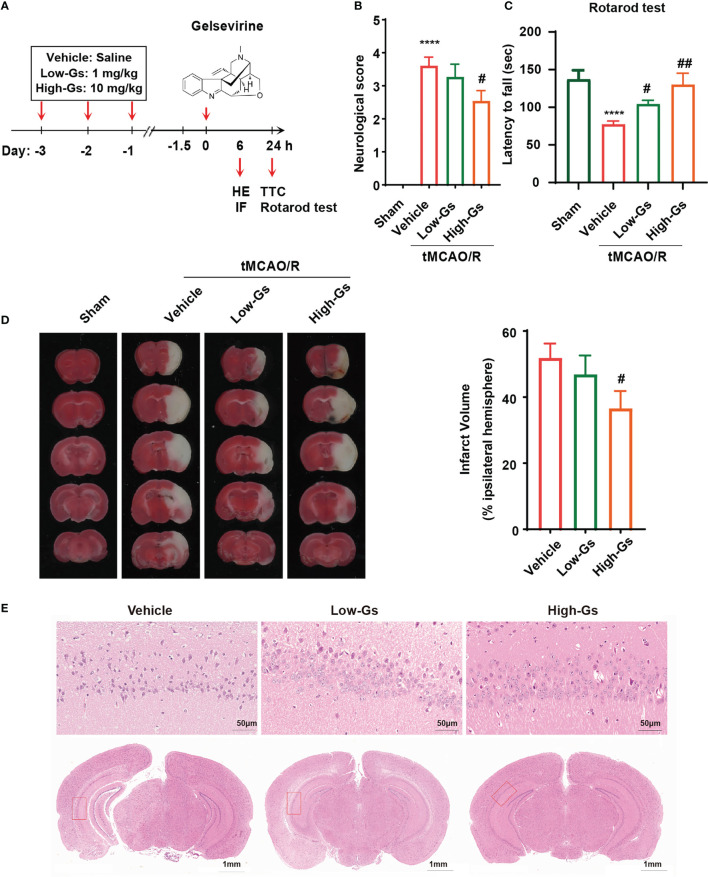
Gelsevirine deceased brain damage and improved neurological functions in tMCAO model mice. **(A)** Molecular structure of Gelsevirine and the design of animal experiment. **(B)** Functional scores were used to evaluate the benefit effect of Gelsevirine on the treatment of stroke, and **(C)** the ability of motor and balance was found significantly rescued by the administration of Gelsevirine in rotarod test. **(D)** Analysis of the infarct volume by TTC staining also proved the protective effect of Gelsevirine on neuron in tMCAO model mice. **(E)** Gelsevirine decreased neuron loss in ischemia penumbra zone (in immunofluorescence n=9-10; in function test and TTC n=12; ****, compared with sham group, P < 0.0001; ## compared with Vehicle, P<0.01; # compared with Vehicle, P<0.05).

Because of the anti-inflammatory effect of Gs on microglia, it should decrease the inflammation in stroke which might protect the brain from injuries. The ischemia/reperfusion mice (tMCAO) model was used to induce ischemia/reperfusion insult, and the administrate protocol is shown in **Figure 2A**. Briefly, 8 weeks old male C57BL/6 mice were administrated with Gs or vehicle 1 hour before the tMCAO, and mice received another treatment with the same protocol at reperfusion onset 24 hours after tMCAO. Functional scores were also applied to evaluate the neurological outcome 24 h post-tMCAO. Compared with the vehicle group, Gs significantly rescued neurological deficits in the Bederson score for the vehicle vs the high-dose Gs group (p < 0.05, N = 12 per group; **Figure 2B**). In the rotarod test, high dose of Gs significantly increased the dropping time of tMCAO mice, which indicated that Gs rescued the motor and balance ability in tMCAO model mice (p < 0.05, n = 12 per group; **Figure 2C**). After the functional evaluations of different administrations of Gs, infarct volume was assessed by TTC staining, which was significantly smaller in high-dose Gs-treated mice than in vehicle-treated mice (**Figure 2D**). By HE staining, high-dose Gs decreased the tissue edema and the loss of neuron of hippocampus in the ischemic-reperfusion side compared with model mice (**Figure 2E**).

These data proved that Gs could decrease brain damage and rescue neurobiological function after a stroke.

### 3.3 Gelsevirine reduced over-activity of microglia in tMCAO mice and *in vitro*

**Figure 3:**
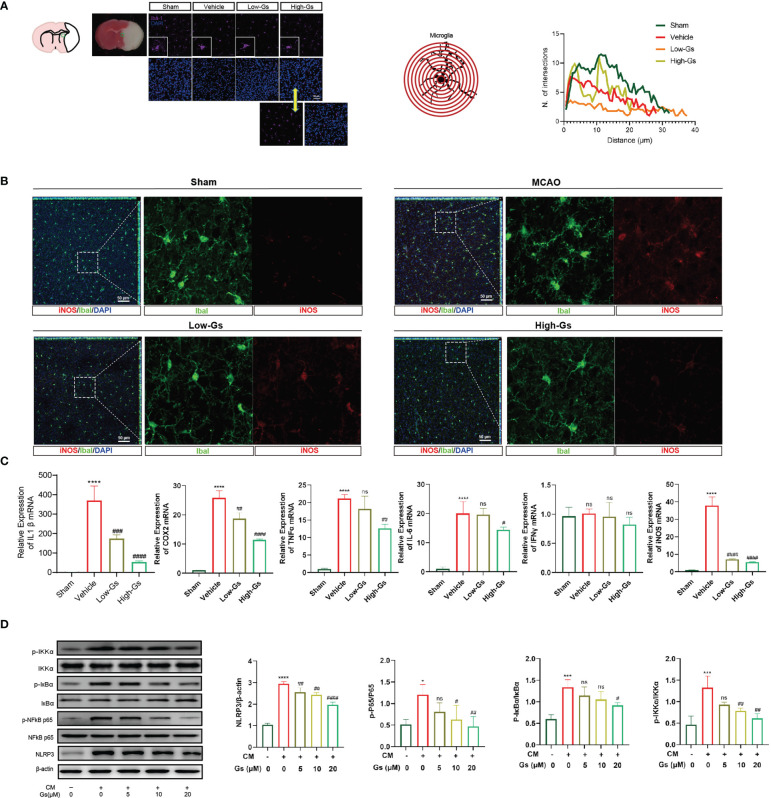
Gelsevirine deceased activity of microglia in tMCAO mice and NFκB pathway, autophagy pathway and ROS-related signal pathway in CM treated BV2 cells. **(A)** In the ischemia penumbra zone of tMCAO mice, microglia were significantly activated by sholl analysis on morphologic characteristics and the activity of microglia could be decreased by Gs. **(B)** In the ischemia penumbra zone of tMCAO mice, increased expression of iNOS in microglia was significantly downregulated by Gs. **(C, D)** *In vitro*, Gs decreased the expression of inflammatory cytokines and inflammatory related signaling pathways in CM stimulated microglia model (****, compared with sham group or untreated group, P < 0.0001; ***, compared with sham group or untreated group, P < 0.001; *, compared with sham group or untreated group, P < 0.05; ####, compared with vehicle group or CM group, P < 0.0001; ###, compared with CM or Vehicle group, P<0.001; ##, compared with CM or Vehicle group, P<0.01; #compared with CM or Vehicle group, P<0.05; ns, compared with CM or Vehicle group, P > 0.05; n=3.

Over-activated inflammatory status is vital to start and/or enhance the damage to the brain in stroke, and the regulation of inflammation is an important treatment strategy. As our previous report, Gs decreased the inflammation in peripheral tissue, so we evaluated the influence of Gs on neuroinflammation in stroke. The activation of microglia, a primary resident immune cell, was evaluated by their morphologic characteristics (**Figure 3A**), and the administration of Gs in ischemia penumbra significantly decreased over-activated microglia. The pro-inflammatory type of microglia near the ischemia penumbra was marked by the stain of Iba1 and iNOS, and the administration of Gs significantly reduced the ratio of iNOS-positive microglia near the ischemia penumbra (**Figure 3B**). To further certify the anti-inflammatory effect of Gs, an array of inflammatory cytokines, including IL-1β, TNF-α, IL-6, cyclooxygenase (COX)-2 and IFN-γ, were analyzed by qPCR. These cytokines were quickly increased after stroke (**Figure 3C**), whereas the administration of Gs significantly rescued the over-production of pro-inflammatory cytokines. The downregulation of NFκB signaling pathway and NLRP3 by the administration of Gs in a microglia model indicated that the beneficial effects of Gs should come from the regulation of microglia (**Figure 3D**).

These results indicated that the administration of Gs decreased neuroinflammation by the down-regulation of inflammatory cytokines *in vitro* and *in vivo*.

### 3.4 Gelsevirine reduced the oxide stress in tMCAO mice and microglia *in vitro*

**Figure 4:**
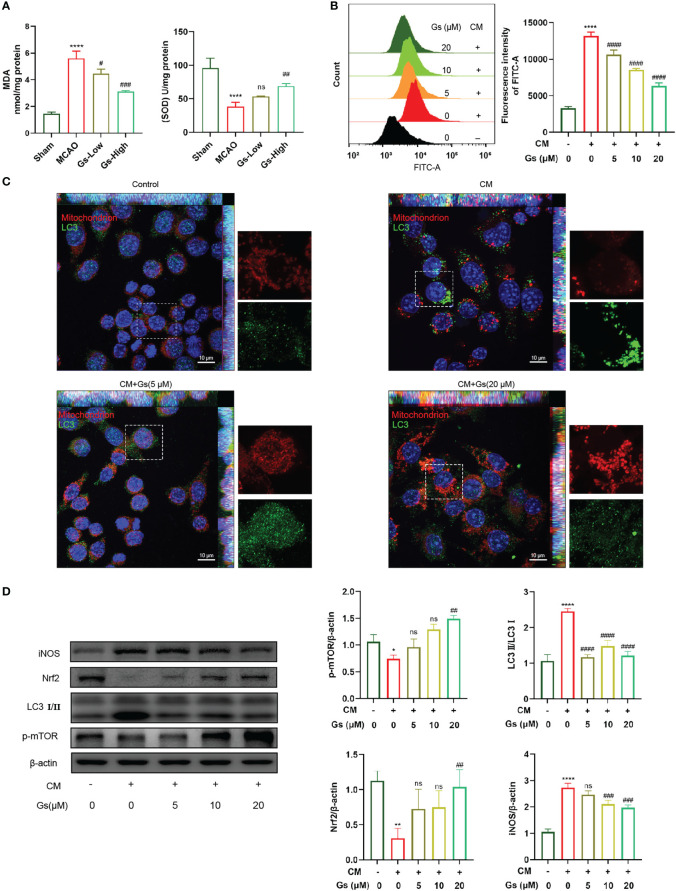
Gelsevirine rescued the level of mitochondria-related damage and ROS. **(A)** Gs rescued levels of MDA and SOD in MCAO mice. **(B)** The administration of Gs significantly decreased ROS by the stimulation of CM. **(C)** Accumulation of autophagy-related protein to mitochondria was attenuated by administering Gs in CM treated BV2 cells. **(D)** Gs increased the expression of Nrf2, an antioxidant gene and downregulated the expressions of iNOS, p-mTOR and LC3 which are tightly related to the ROS and autophagy. (****, compared with sham group or untreated group, P < 0.0001; **, compared with untreated group, P < 0.01; *, compared with untreated group, P < 0.05; ####, compared with CM group, P < 0.0001; ###, compared with sham group or CM group, P < 0.001; ##, compared with sham group or CM group, P < 0.01; #, compared with CM group, P < 0.05; ns compared with CM group, P > 0.05; n = 3).

ROS and NO from neuroinflammation induce cell damage in the ischemic penumbra. As shown in **Figure 4A**, the levels of SOD and MDA, which reflect the level of ROS in the ischemic penumbra, were rescued by the administration of Gs. *In vitro*, OGD neuron-conditioned medium can significantly induce the production of ROS in microglia. This effect was inhibited by the administration of Gs, which should also contribute to its protective effect on stroke (**Figure 4B**). The production of ROS was related to the dysfunction of mitochondria and the stimulation of OGD neuron-conditioned medium significantly increased the rate of abnormal mitochondria which was rescued by the administration of Gs (**Figure 4C**). Besides ROS production, Gs also rescued the expression of Nrf2, an essential factor activating endogenous antioxidant mechanisms. They decreased the expression of iNOS which positively regulates ROS (**Figure 4D**). LC3I/II and pmTOR which regulates autophagy and metabolic process are also regulated by the administration of Gs (**Figure 4D**).

These results show that Gs downregulated the oxide stress in stroke by normalizing mitochondria, upregulation of endogenous antioxidant mechanisms and downregulation of stress-related factors.

### 3.5 RNAseq analysis indicates JAK-STAT signaling pathway is the key pathway rescued in MCAO mice by the administration of Gelsevirine

**Figure 5:**
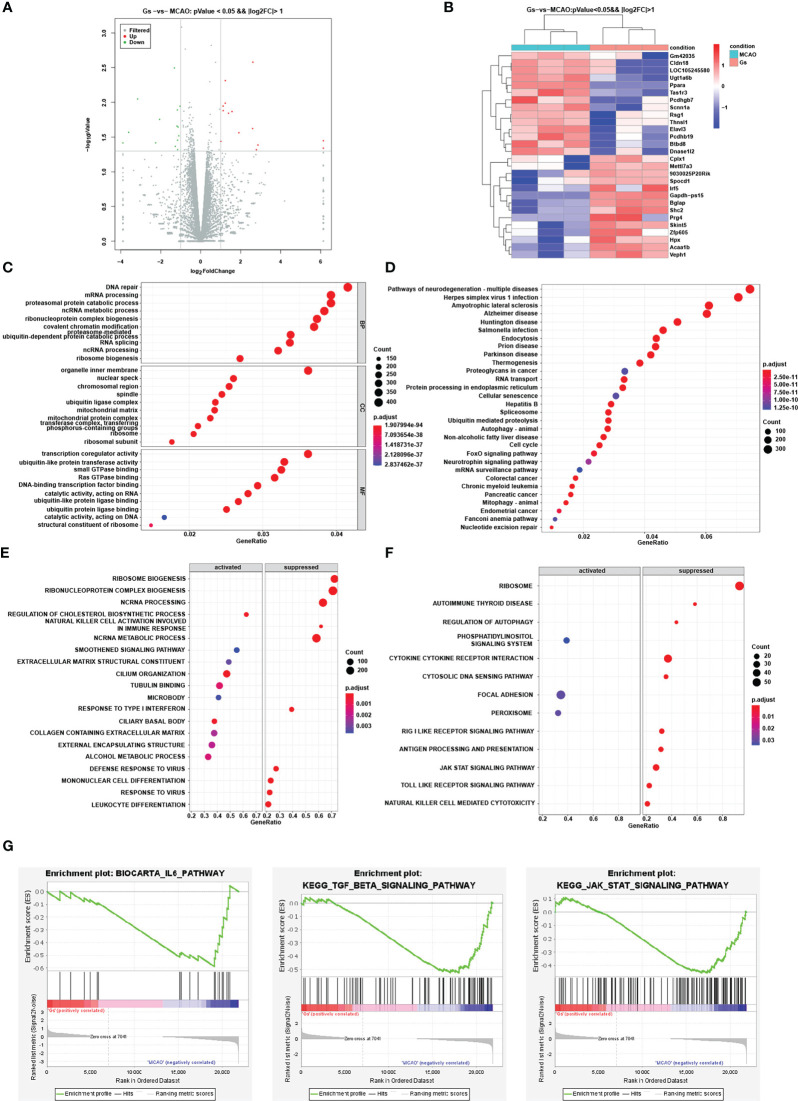
Analysis of the influence and pathways of Gelsevirine in the ischemia penumbra zone by RNA-seq. **(A)** Different expressed genes were showed by volcano and **(B)** the clustering analysis was showed by heatmap. **(C, D)** GO and KEGG enrichment were used to evaluate the influence of Gelsevirine on stroke. **(E, F)** GSEA method was used to GO and KEGG enrichment analysis which can explore the effect of Gelsevirine on stroke. **(G)** Important pathways related to inflammatory changes by GSEA analysis.

Although previous results proved the regulation of Gs on the NFκB pathway, autophagy pathway, and ROS-related signal pathway to decrease the inflammation, the mechanism of Gs in anti-inflammation in stroke is still unclear. To further explore the critical pathway of Gs decreasing neuroinflammation in the stroke, RNAseq was used to explore the effect of Gs in ischemia penumbra. Differential expression genes (DEGs) were evaluated and significantly changed genes were clustering analyzed and shown by volcano plot and heatmap (**Figures 5A, B**). According to Gene Ontology (GO) enrichment analysis, many significant functional sets were influenced by the administration of Gs, such as DNA repair, inner organelle membrane, transcription coregulator activity and so on (**Figure 5C**). Many signal pathways, such as neurodegenerative diseases, endocytosis and so on, were enriched by KEGG analysis in the Gs treated group (**Figure 5D**). Because of the limited number of different genes, the responses to Gs were thought to distribute across the whole network of genes, and individual genes might be subtle. So, a GSEA method was used to evaluate the influence of Gs. As shown in **Figure 5E**, the administration of Gs significantly influenced mangy bioprocesses by GSEA analysis, such as oxidative phosphorylation pathway, proteasome pathway, ribosome pathway, mitochondrial electron transport NADH to ubiquinone, ATP synthesis coupled electron transport establishment of protein localization to the endoplasmic reticulum, and so on. Gs also significantly suppressed many KEGG pathways by GSEA analysis, such as the JAK-STAT signaling pathway, focal adhesion pathway, and phosphatidylinositol signaling system (**Figure 5F**). Signal pathways significantly related to the inflammation, such as IL-6, TGF-β and JAK-STAT, were significantly related to the protective effect (**Figure 5G**).

Based on results from RNAseq and the cross-talk of these signal pathways, it is suggested that the JAK-STAT pathway plays an essential role in attenuating MCAO-induced inflammation dysfunction in ischemia penumbra by Gs.

### 3.6 Direct inhibition of JAK2 by Gelsevirine contributed to the anti-inflammatory effect on microglia activation *in vitro*

**Figure 6:**
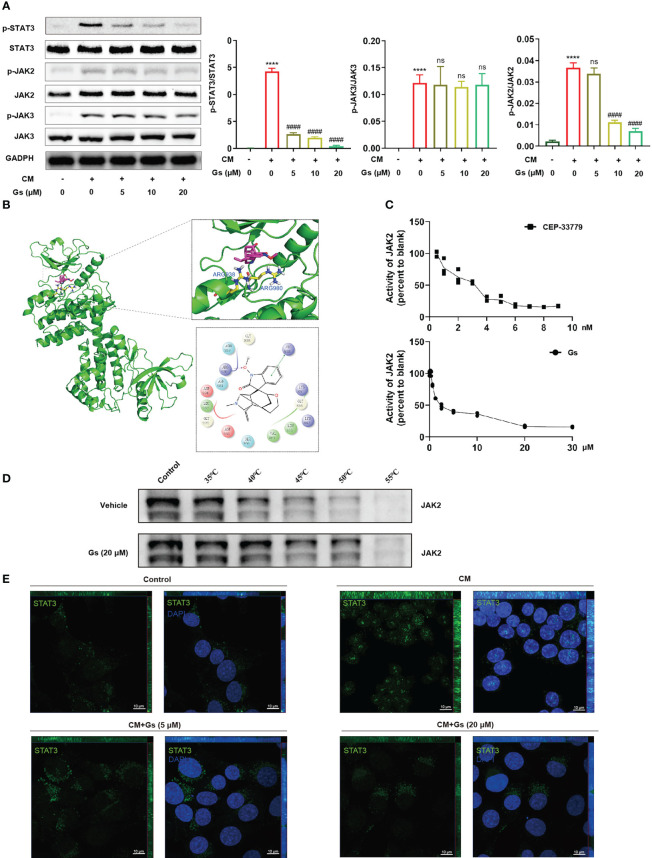
Gelsevirine decreased the JAK2-STAT3 signal pathway by inhibiting the activity of JAK2 *in vitro*. **(A)** Gelsevirine significantly decreased phosphorylation of STAT3 in CM-induced microglia *in vitro*. **(B)** Molecular docking indicated the potential inhibition of Gelsevirine on JAK2 and **(C)** Gelsevirine inhibited the kinase activity of JAK2. **(D)** Gelsevirine inhibited the degradation of JAK2 in thermal shift assay. **(E)** Gelsevirine inhibited the nuclear distribution of STAT3 *in vitro*. (****, compared with untreated group, P < 0.0001; ####, compared with CM group, P < 0.0001; ns compared with CM group, P > 0.05; n = 3).

To certify the critical role of the JAK-STAT pathway, the influences of Gs on JAK and STAT were evaluated in an *in vitro* model. As shown in **Figure 6A**, phosphorylated STAT3 was significantly increased in CM-treated microglia and down-regulated by Gs. Phosphorylated JAK2 but not phosphorylated JAK3, both of which are upstream factors of STAT3, was down-regulated by Gs in the CM treated model (**Figure 6A**). Interestingly, phosphorylated STAT3 was downregulated by low-dose Gs treatment, but the phosphorylated JAK2 was still at a relatively high level (**Figure 6A**). It indicated that Gs could down-regulate the phosphorylation of STAT3 independent on the downregulation of phosphorylation of its upstream signals, such as the direct inhibition of the enzymatic activity of JAK2, which also contributes to inhibition of the JAK2-STAT3 signal pathway. By molecular docking, Gs was consistent with the positive drug (CEP-33779) and embedded in the active binding pocket of the JAK2 protein (PDB:4AQC), where the benzene ring on the Gs structure forms a π-π bond interaction with ARG938 on the JAK2 protein. In addition, the oxygen atom on the side chain in the Gs structure can form a crucial hydrogen bond interaction with the Arg 980 residue of the protein (**Figure 6B**). Then, the inhibiting effect of Gs on JAK2 was compared with CEP-33779, a JAK2 inhibitor used in PDB:4AQC, and the result proved that Gs inhibited the kinase activity of JAK2 *in vitro* (**Figure 6C**). By thermal shift assay, Gs significantly increased the thermal stabilization of JAK2, indicating Gs binds to JAK2 directly (**Figure 6D**). Immunofluorescent staining was conducted to analyze the distribution of STAT3. In quiescent BV2 cells, STAT3 was mainly distributed in the cytoplasm (**Figure 6E**). After the stimulation of CM, STAT3 rapidly translocated to nuclei, and the nuclear distribution of STAT3 can be blocked by Gs (**Figure 6E**).

**Figure 7:**
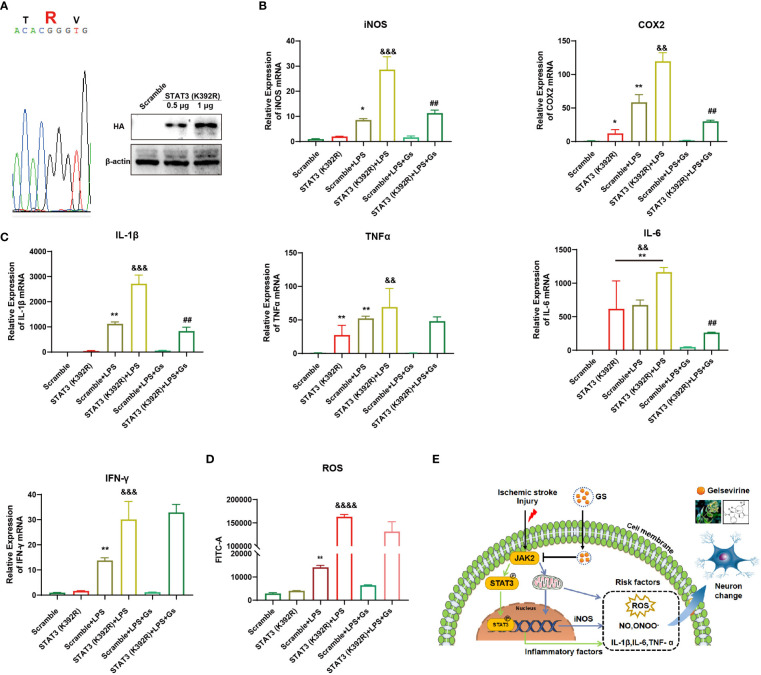
Over-activity of STAT3 abolished the anti-inflammatory effect of Gelsevirine in microglia. A gain-of-function STAT3 mutation (K392R) was over-expressed in BV2 cell by Lipofectamine^®^ 3000 **(A)** which increased the inflammatory response and anti-inflammatory effect of Gelsevirine in BV2, such as the expression of iNOS and COX2 **(B)**, the production of inflammatory cytokines **(C)** and ROS **(D)**. It indicated that the inhibition of Gs on the JAK2-STAT3 signaling pathway decreased the expressions of inflammatory and ROS-related factors, which benefit the rescue of stroke **(E)**. (**, compared with Scramble group, P < 0.01; *, compared with Scramble group, P < 0.05; &&&&, compared with STAT3 (K392R) group, P < 0.0001; &&&, compared with STAT3 (K392R) group, P < 0.001; &&, compared with STAT3 (K392R) group, P < 0.01; ##, compared with STAT3 (K392R) + LPS group, P < 0.01; n = 3).

To further prove the critical role of JAK2-STAT3 on the anti-inflammatory effect of Gs, a gain-of-function STAT3 mutation (K392R) (29) was used to evaluate the role of STAT3 on Gs treatment in microglia. After the over-expression of STAT3(K392R) (**Figure 7A**), inflammatory factors associated with STAT3 were upregulated under basal conditions, and the over-expressions significantly enhanced expressions of ROS-related factors and inflammatory factors on the stimulation of CM (**Figures 7B, C**). The over-expression of STAT3(K392R) also abolished the anti-inflammatory effect of Gs in microglia (**Figures 7B, C**). The level of ROS was also increased by the over-expression of STAT3 mutation (K392R) in basal conditions, and STAT3 mutation (K392R) made microglia more sensitive to the stimulation of CM, which were not rescued by the administration of Gs (**Figure 7D**).

These results proved that the anti-inflammatory effect of Gs depended on the inhibition of JAK2, which decreased the transcriptional activity of STAT3 on inflammatory factors and the production of ROS in microglia (**Figure 7E**). These effects of Gs benefit the decrease of neuroinflammation in stroke.

In conclusion, Gs is beneficial to stroke by the downregulation of neuroinflammation through multiple pathways, in which the inhibition of the JAK2-STAT3 signal pathway plays a critical role, and the attenuation of ROS, mitochondrial dysfunction and other inflammatory pathways also involved in the protective effects of Gs.

## 4 Discussion

In this study, we certified the protective capability of Gelsevirine (Gs) on stroke in the tMCAO model, in which Gs significantly reduced the infarct volumes, ischemia/reperfusion-induced neuronal apoptosis and inflammation, especially in the penumbra zone. The beneficial effects of Gs on stroke were related to the inhibition of the pro-inflammatory activity of microglia, such as the production of inflammatory cytokines, the level of ROS and so on. Furthermore, the anti-inflammatory effect of Gs was tightly related to the inhibition of the JAK-STAT signal pathway by RNAseq analysis. By molecular docking, inhibition assay and thermal shift assay, the inhibition of Gs to JAK2 contributed to the regulation of the JAK2-STAT3 signaling pathway. Over-expression of a gain-of-function STAT3 mutation (K392R) abolished the anti-inflammatory effects of Gs. Consequently, Gs benefited to ischemic stroke by regulating neuroinflammation through the inhibition of JAK2-STAT3 signaling pathway.

In the pathophysiology of ischemic stroke, inflammation has become an essential target for developing stroke therapies (30–32). As a brain-resident immune cell, microglial cells are sensitive to imbalances in the central nervous system (CNS) of stroke because of the expressions of receptors recognizing immune signals and danger signals from dying cells, pathogens and self-antigens (33–35). In the ischemic brain, microglial cells were quickly activated and enriched in the regions around the infarct (12, 36, 37). Although microglial cells can engulf the damaged neurons and participle in debris clearance essential to maintain CNS homeostasis, activated microglia induce the cascades of inflammatory events, oxidative stress and neuron death in the injured brain after stroke (38, 39). Risk factors released from activated microglia, such as TNF-α, glutamate, cathepsin B, reactive oxygen and nitrogen species, are widely certified to induce death in surrounding neurons, including apoptotic, excitotoxic and neuronal death (2, 40, 41). Dysregulated microglia are also proven to engulf neurons in the peri-infarct and increase neuron loss in stroke (42). So, targeting inflammation post-stroke, especially over-activated microglia, is currently considered a potential target for stroke therapies.

Activated microglia, especially the M1 type, release reactive oxygen (ROS) and play an essential role in the inflammatory pathways. The excessive generation of intracellular and extracellular ROS leads to direct cellular damage. It increases the activation of the microglia and leukocytes, which release various damaging inflammatory mediators and effectors, including ROS. It is believed that the inhibition of overproduced ROS can suppress intracellular pro-inflammatory signals. Thus, the modulation of redox balance is an effective way to regulate inflammatory responses.

By RNAseq analysis, the JAK-STAT signaling pathway was tightly related to the effect of Gs. It has been reported to initiate neurotoxicity by regulating the expression of different cytotoxic materials, including ROS. According to our results, Gs downregulated the activity of microglia by inhibiting the JAK2-STAT3 pathway, which is vital to decrease neuroinflammation and brain damage. JAK2, a member of the protein-tyrosine kinase family, is an essential regulator of many other signaling molecules tightly related to neuroinflammation. JAK2 can specifically induce the phosphorylation of STAT3, and mounting evidence proves that the JAK2-STAT3 pathway exerts an essential effect on the inflammatory reaction (43, 44). The JAK2-STAT3 pathway affects the expression of many cytokines, such as TNF-α and IL-6. In ischemia/reperfusion, the JAK2-STAT3 pathway contributes to brain damage (45–47). The activities of NOX family members (including NOX1-5, DUOX1, and DUOX2) are tightly related to the generation of ROS (48). STAT3 is also reported to bind to the NOX1 promoter and increase the production of NOX1 which subsequently stimulates the production of O2^•−^ (49). Therefore, the regulation of overactivated STAT3 in microglia is essential to regulate microglial activation, ROS level and neuroinflammation, all of which are tightly related to neuron damage in stroke.

The JAK2-STAT3 signaling pathway is also tightly related to the regulation of autophagy, a crucial pathway maintaining cellular homeostasis and cell survival by removing damaged organelles and abnormally folded proteins. Mitophagy is a type of autophagy clearing impaired mitochondria essential for sustaining the homeostasis of mitochondria and cells (50). Accumulating evidence suggests that damaged mitochondria increased in stoke and mitophagy cannot be eliminated effectively with abnormal mtROS production and mitochondrial DNA, leading to immune hyperactivation, tissue damage, and increased host mortality (51). The regulation of autophagy is essential to eliminate abnormal mitochondria which also contributes to the attenuation of ROS and inflammation (52, 53). The regulation of Gs on the JAK2-STAT3 signaling pathway is also beneficial to decreasing intracellular stress and attenuating inflammation.

Except the regulation of JAK2-STAT3 signaling pathway, Gs should also influence the Toll-like receptor (TLR) signaling pathway according to the result from RNA-seq, in which the activity of TLR2 and TLR4 in microglia is reported to increase the ischemic injury (54). The suppression of TLR signaling pathway could decrease the cytokine production in microglia and attenuate the neurons death-promoting actions in neuron (55). It indicated that multi-targets contribute to the regulation of the activity of microglia by Gs.

In our previous report, the inhibition of STING is essential to decrease the chronic inflammation in osteoarthritis by Gs (20), but it was not enriched in the MCAO model after the administration of Gs by RNA-seq. STING signal pathway is essential to the over-activation of microglia. The inhibition of the STING signal pathway is reported to suppress the shift of microglia to an inflammatory M1-phenotype after MCAO (56). Moreover, the inhibition of STING signaling also downregulates the recruitment of peripheral immune cells which is related to neuroinflammation in stroke (57). The dose of Gs used in osteoarthritis is 5 mg/kg, but 10 mg/kg of Gs protected the mice from MCAO. The inflammatory status in MCAO is acute but chronic inflammation in osteoarthritis (58). The status of inflammation and the dose of Gs might induce the difference in inflammation-related signaling pathways regulated by Gs.

Taken together, the inhibition of Gs on the JAK-STAT signaling pathway plays an essential role in downregulating the over-activation of microglia in stroke. The downregulation of the over-activation of microglia and the protective effect of Gs make it a potential agent for stroke treatment.

## Data availability statement

The original contributions presented in the study are included in the article/**Supplementary Materials**. Further inquiries can be directed to the corresponding authors.

## Ethics statement

The animal study was reviewed and approved by Ethics Committee of Shanghai University (Approval NO. ECSHU2021-167).

## Author contributions

LS and YZ conceived and designed the experiments; CX, JL, ZZ, DC, RG, WC, and CZ participated in the experiments; CX, JL, HB, and XT analyzed the data; LS, YZ, and XT wrote the manuscript; LS and WC provided funding support for the research. All authors contributed to the article and approved the submitted version.
