# Supplementary figures and images for: Regulation of microglia related neuroinflammation contributes to the protective effect of Gelsevirine on ischemic stroke (part 1 of 2)
Source: Front Immunol. 2023 Mar 30;14:1164278. doi: 10.3389/fimmu.2023.1164278 (PMC10098192; doi:10.3389/fimmu.2023.1164278)

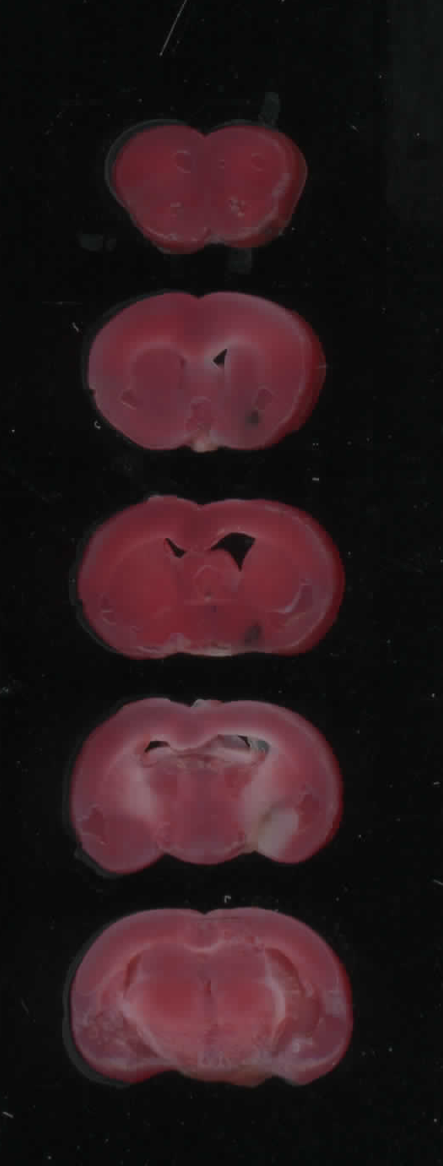

Supplement: Supplementary file 2 [file DataSheet_2.zip › fig 2 raw-D/fig 2-D1 raw/21.png]

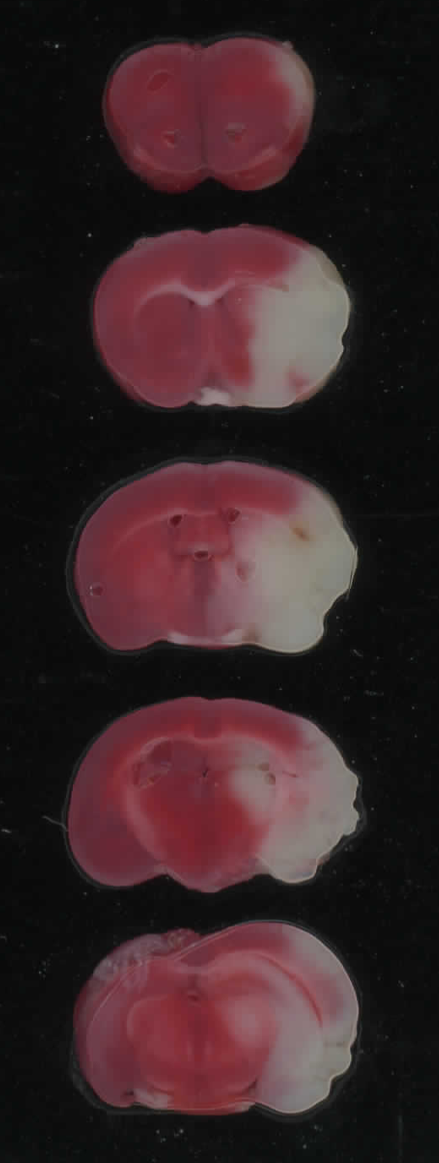

Supplement: Supplementary file 2 [file DataSheet_2.zip › fig 2 raw-D/fig 2-D1 raw/22.png]

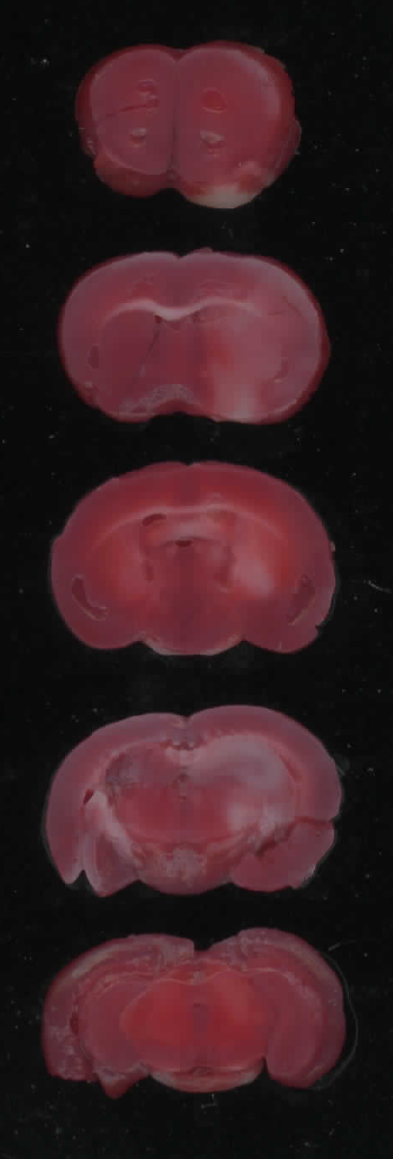

Supplement: Supplementary file 2 [file DataSheet_2.zip › fig 2 raw-D/fig 2-D1 raw/23.png]

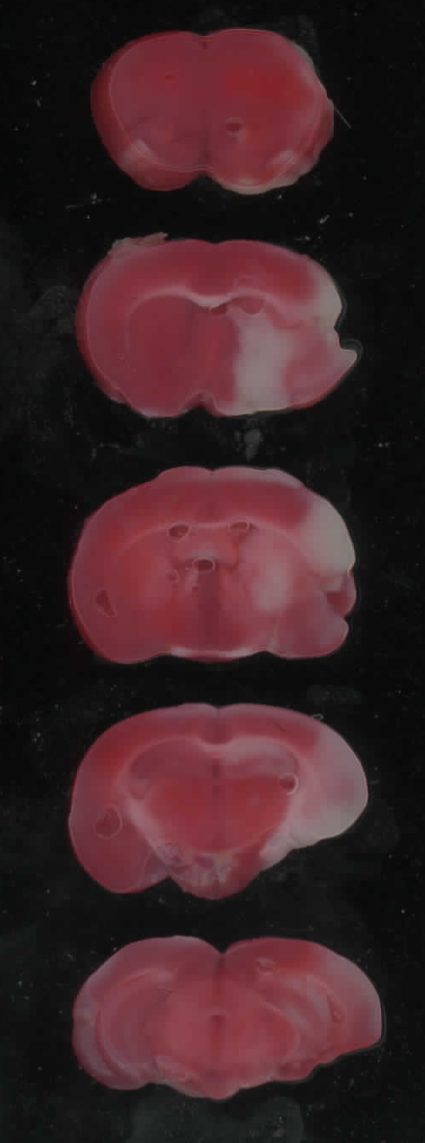

Supplement: Supplementary file 2 [file DataSheet_2.zip › fig 2 raw-D/fig 2-D1 raw/24.png]

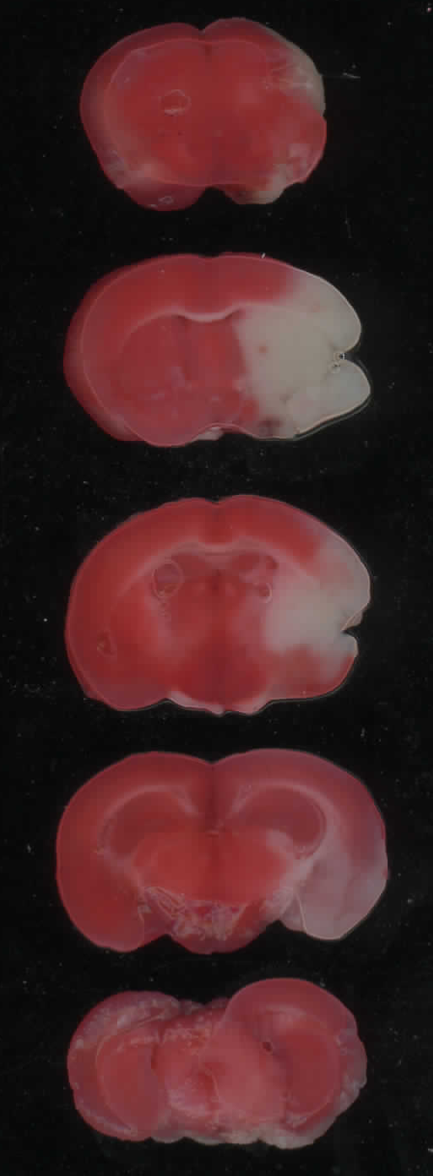

Supplement: Supplementary file 2 [file DataSheet_2.zip › fig 2 raw-D/fig 2-D1 raw/25.png]

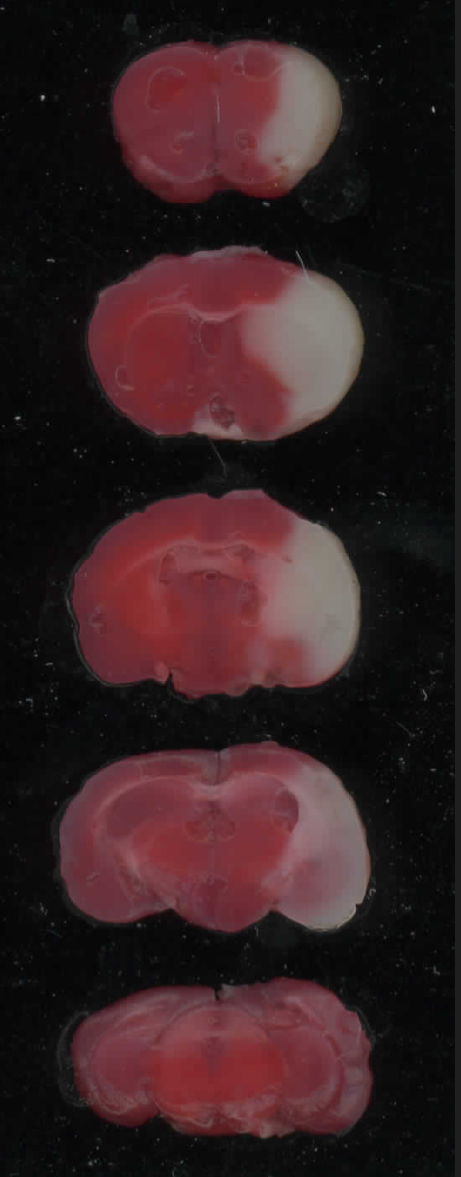

Supplement: Supplementary file 2 [file DataSheet_2.zip › fig 2 raw-D/fig 2-D1 raw/26.png]

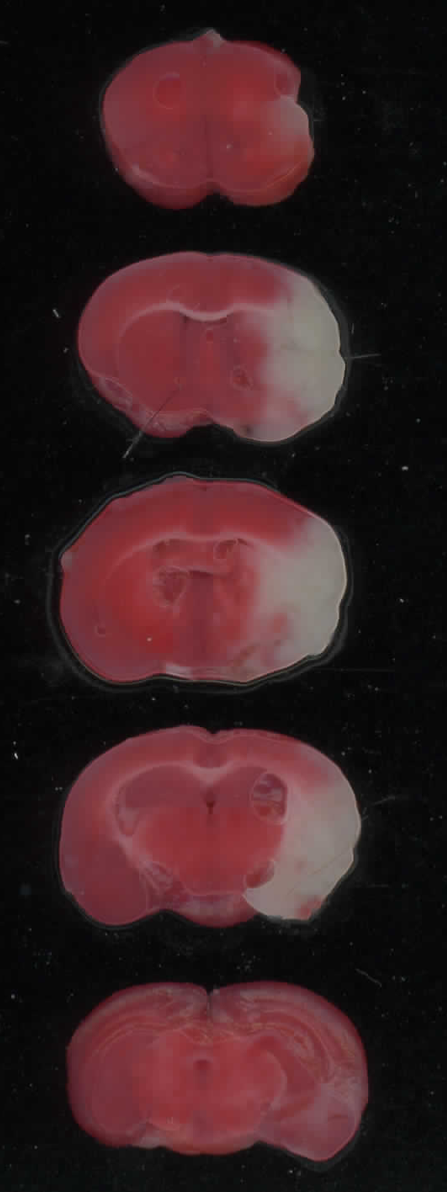

Supplement: Supplementary file 2 [file DataSheet_2.zip › fig 2 raw-D/fig 2-D1 raw/27.png]

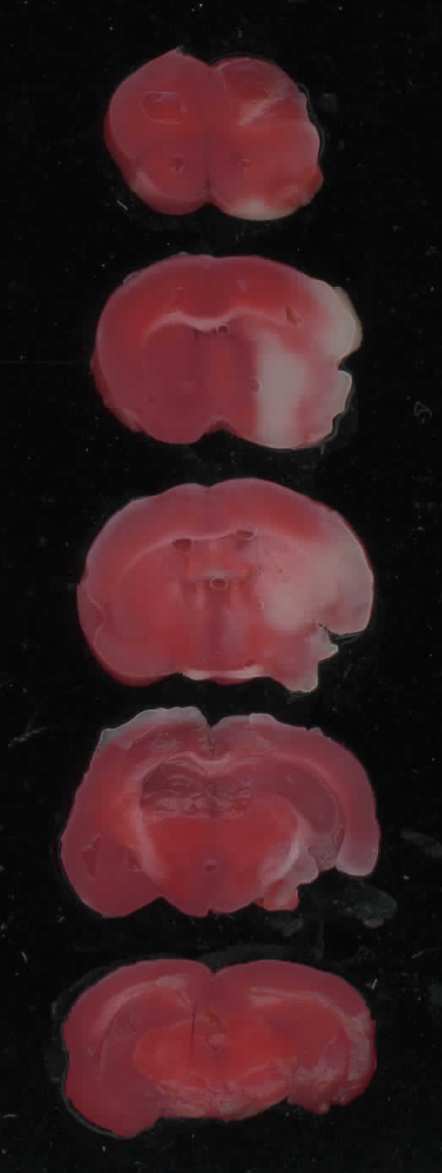

Supplement: Supplementary file 2 [file DataSheet_2.zip › fig 2 raw-D/fig 2-D1 raw/28.png]

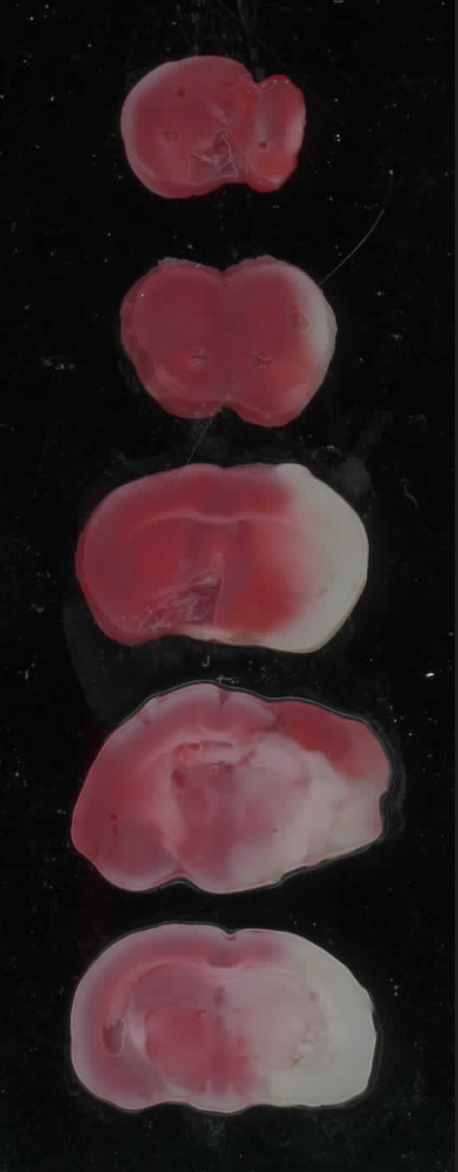

Supplement: Supplementary file 2 [file DataSheet_2.zip › fig 2 raw-D/fig 2-D1 raw/29.png]

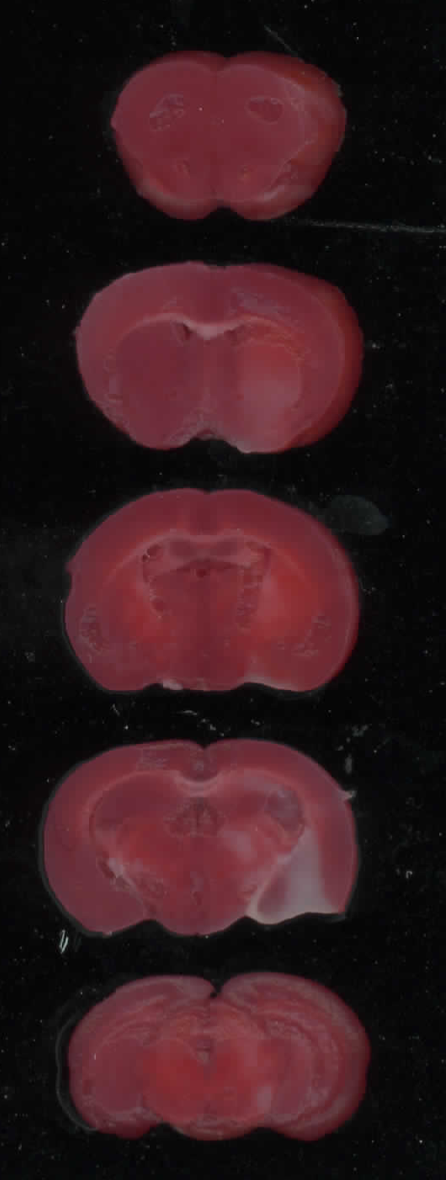

Supplement: Supplementary file 2 [file DataSheet_2.zip › fig 2 raw-D/fig 2-D1 raw/31.png]

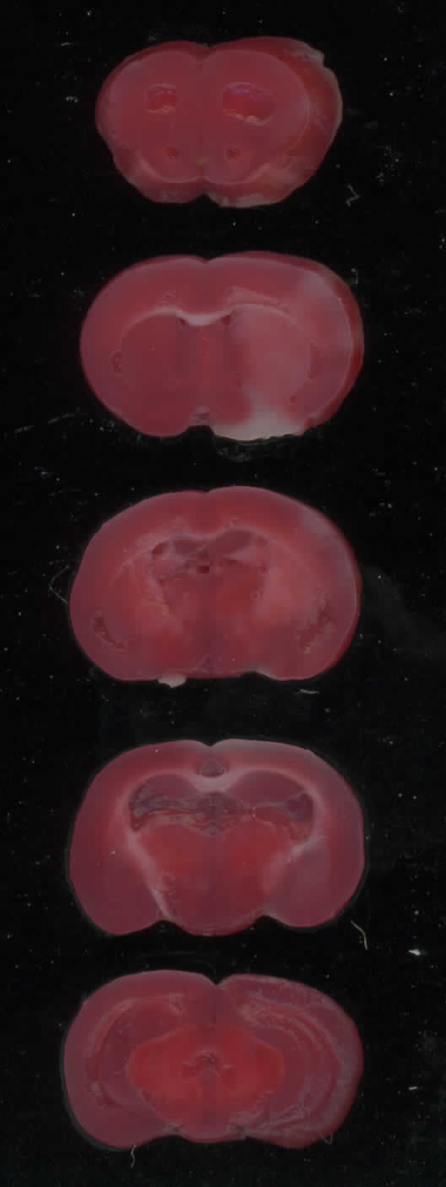

Supplement: Supplementary file 2 [file DataSheet_2.zip › fig 2 raw-D/fig 2-D1 raw/32.png]

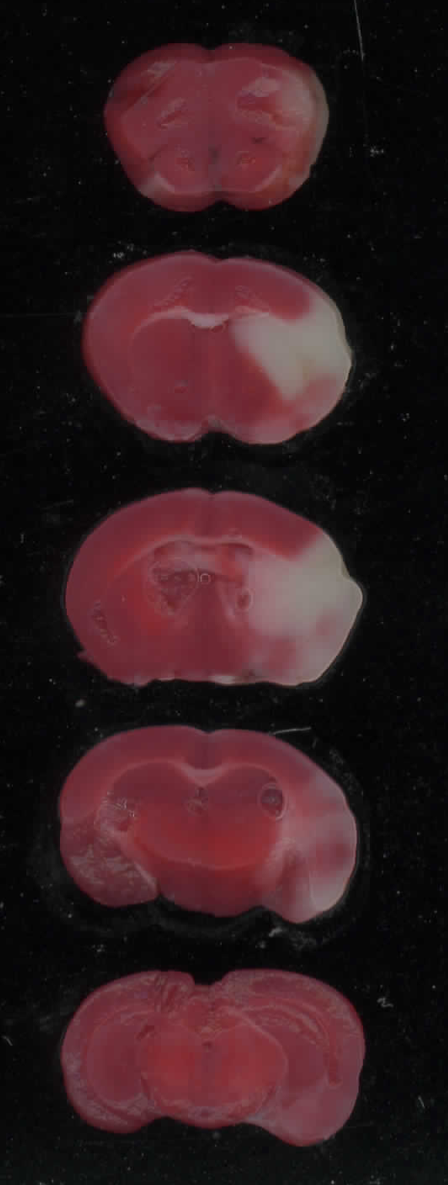

Supplement: Supplementary file 2 [file DataSheet_2.zip › fig 2 raw-D/fig 2-D1 raw/33.png]

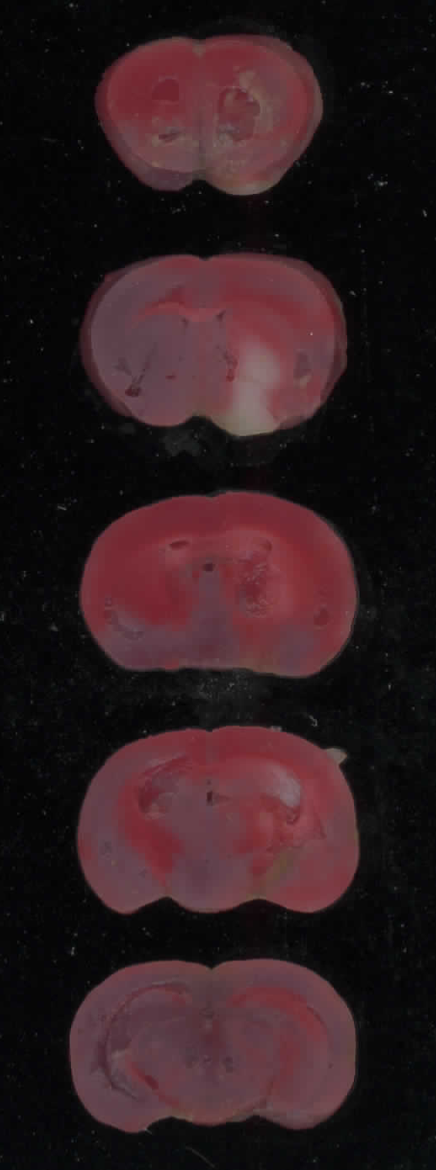

Supplement: Supplementary file 2 [file DataSheet_2.zip › fig 2 raw-D/fig 2-D1 raw/34.png]

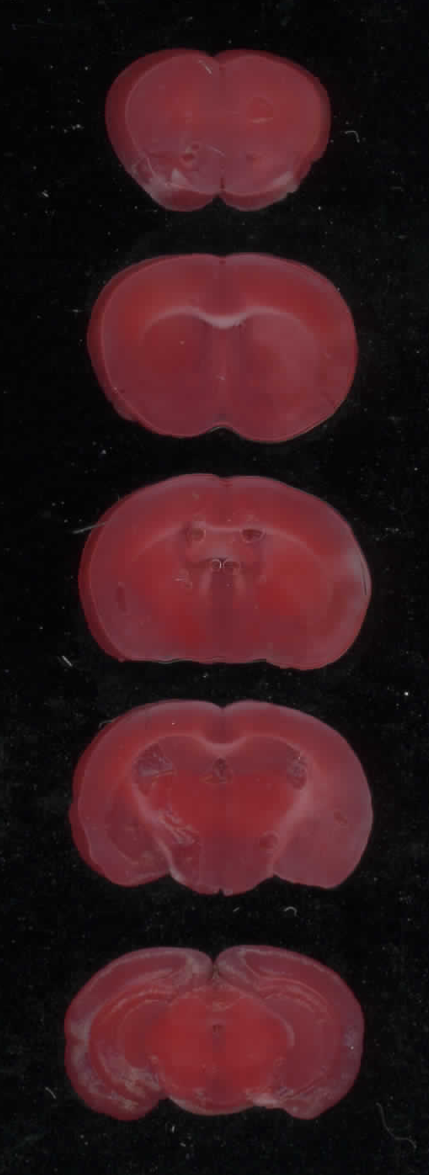

Supplement: Supplementary file 2 [file DataSheet_2.zip › fig 2 raw-D/fig 2-D1 raw/35.png]

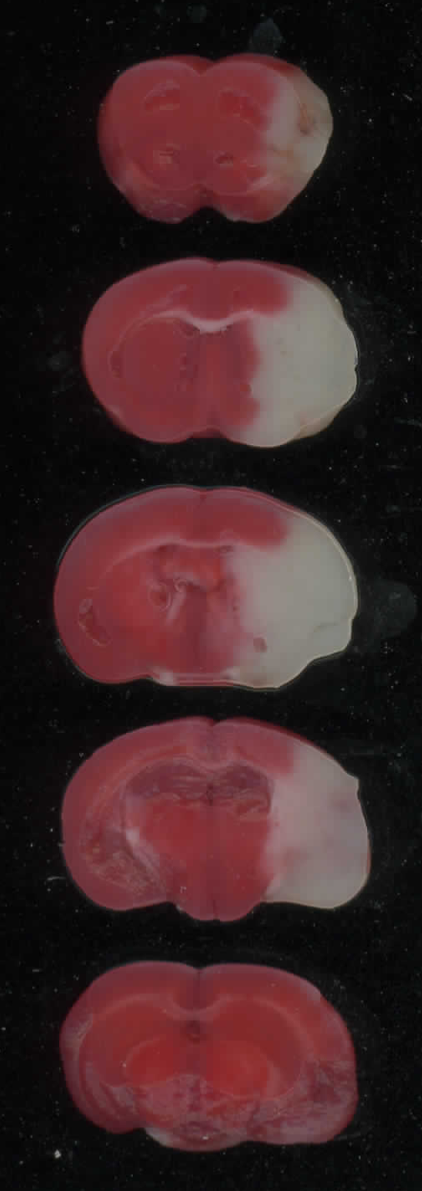

Supplement: Supplementary file 2 [file DataSheet_2.zip › fig 2 raw-D/fig 2-D1 raw/36.png]

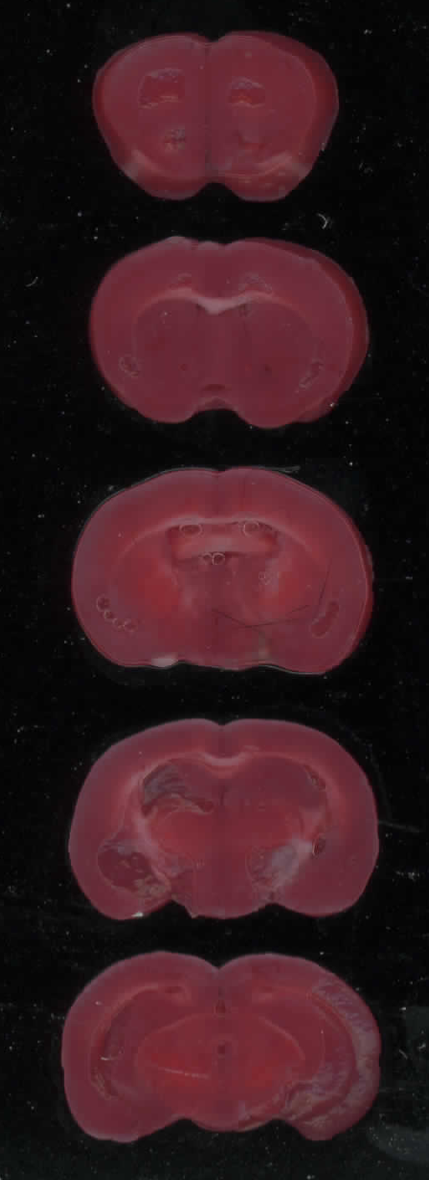

Supplement: Supplementary file 2 [file DataSheet_2.zip › fig 2 raw-D/fig 2-D1 raw/37.png]

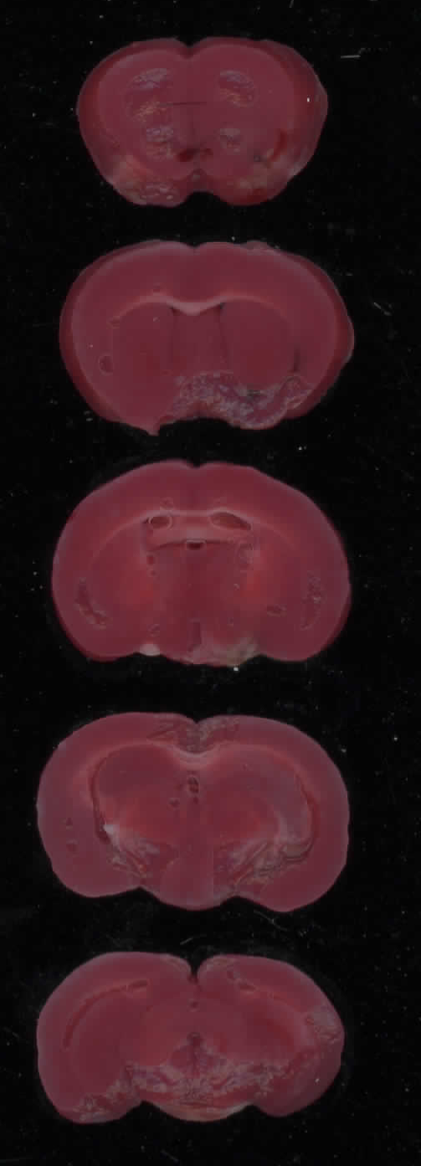

Supplement: Supplementary file 2 [file DataSheet_2.zip › fig 2 raw-D/fig 2-D1 raw/38.png]

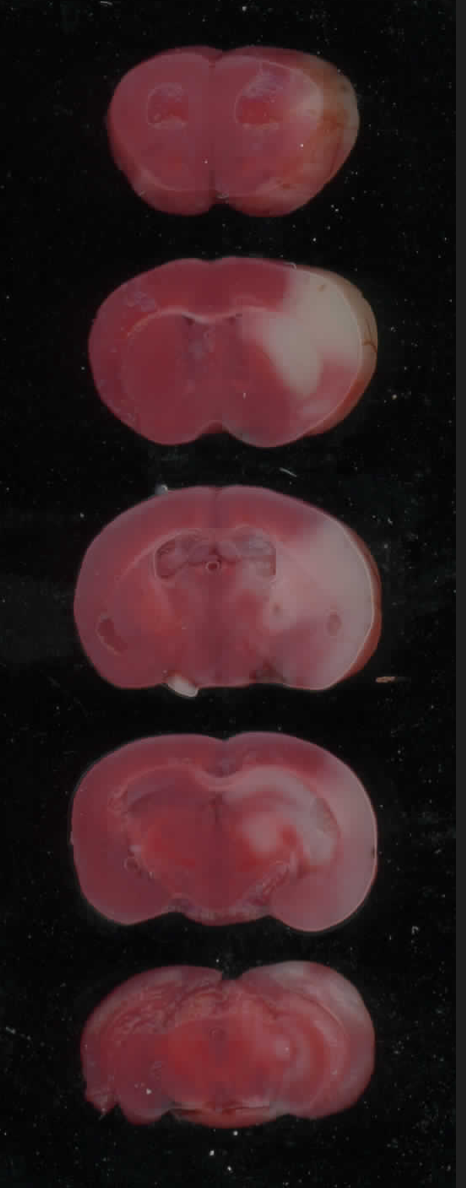

Supplement: Supplementary file 2 [file DataSheet_2.zip › fig 2 raw-D/fig 2-D1 raw/39.png]

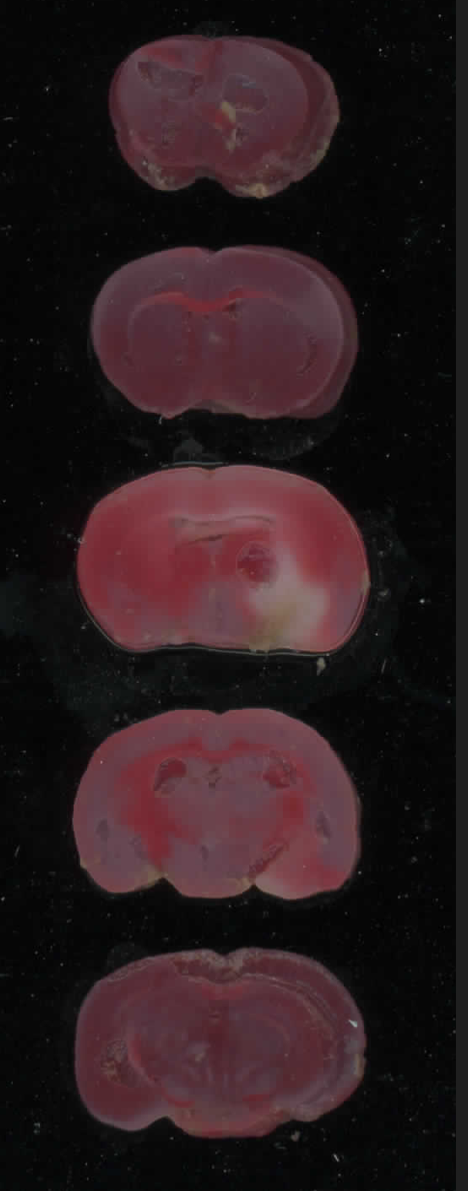

Supplement: Supplementary file 2 [file DataSheet_2.zip › fig 2 raw-D/fig 2-D1 raw/40.png]

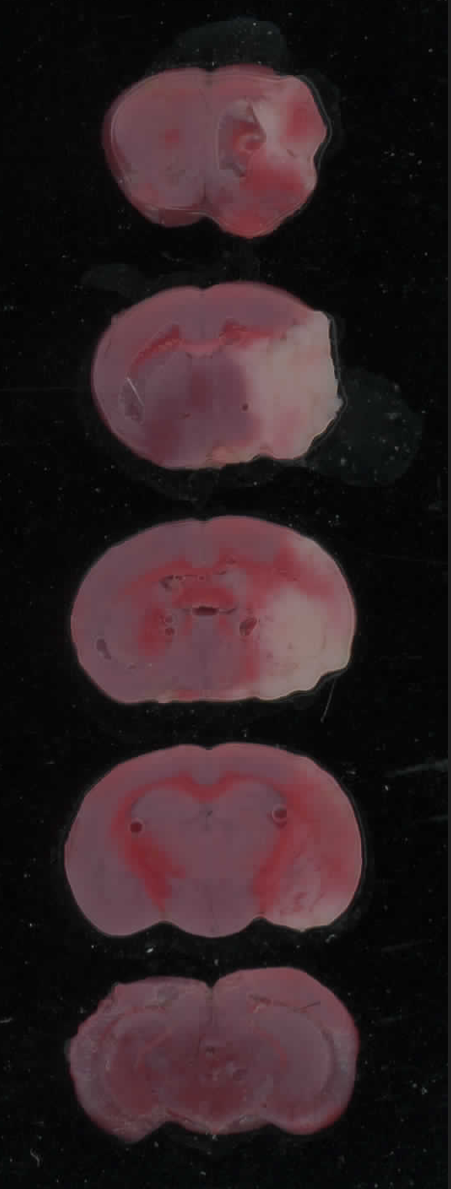

Supplement: Supplementary file 2 [file DataSheet_2.zip › fig 2 raw-D/fig 2-D1 raw/41.png]

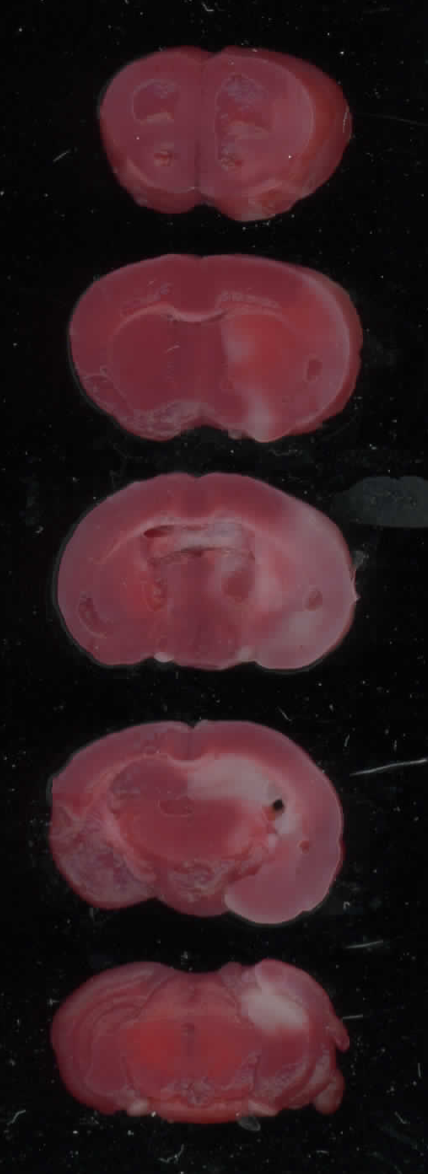

Supplement: Supplementary file 2 [file DataSheet_2.zip › fig 2 raw-D/fig 2-D1 raw/42.png]

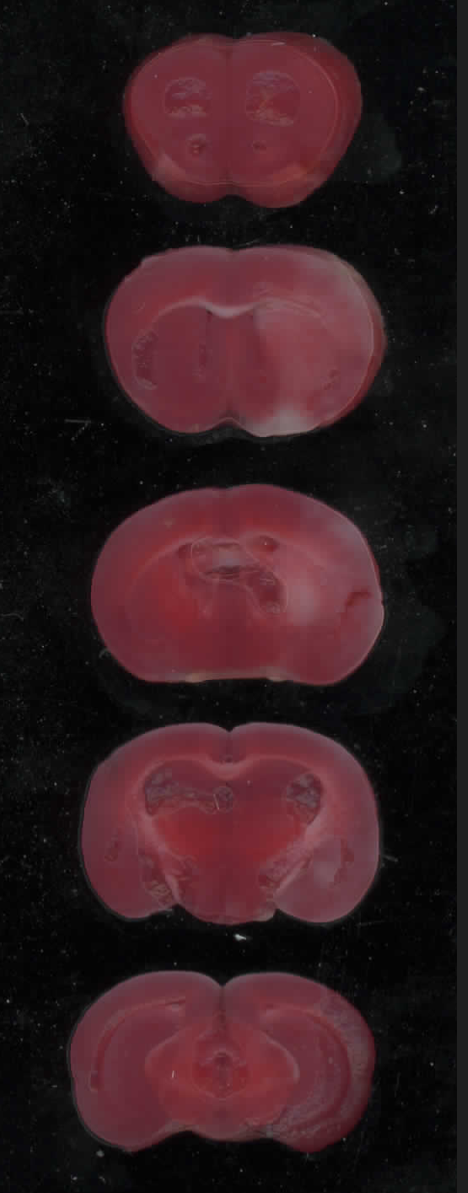

Supplement: Supplementary file 2 [file DataSheet_2.zip › fig 2 raw-D/fig 2-D1 raw/43.png]

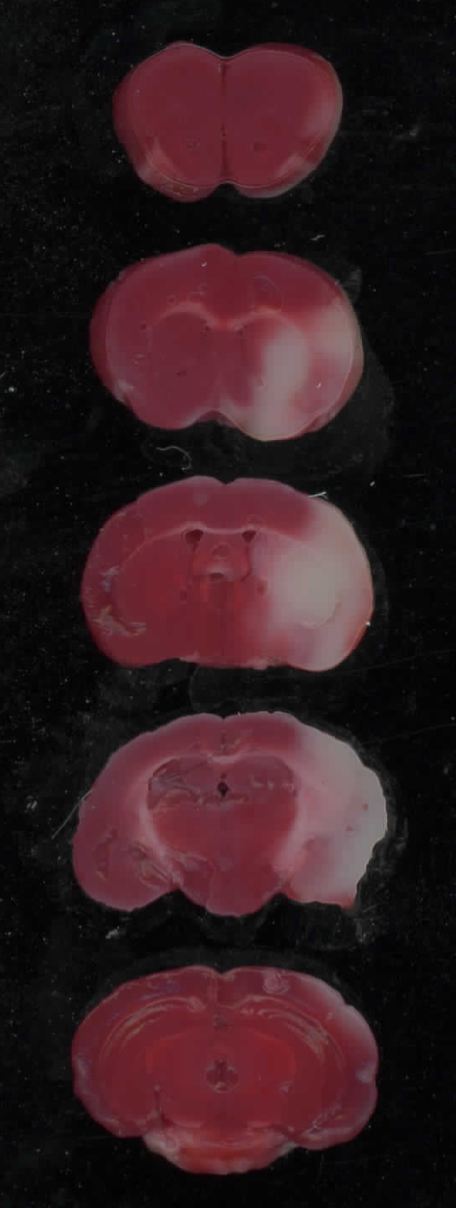

Supplement: Supplementary file 2 [file DataSheet_2.zip › fig 2 raw-D/fig 2-D1 raw/45.png]

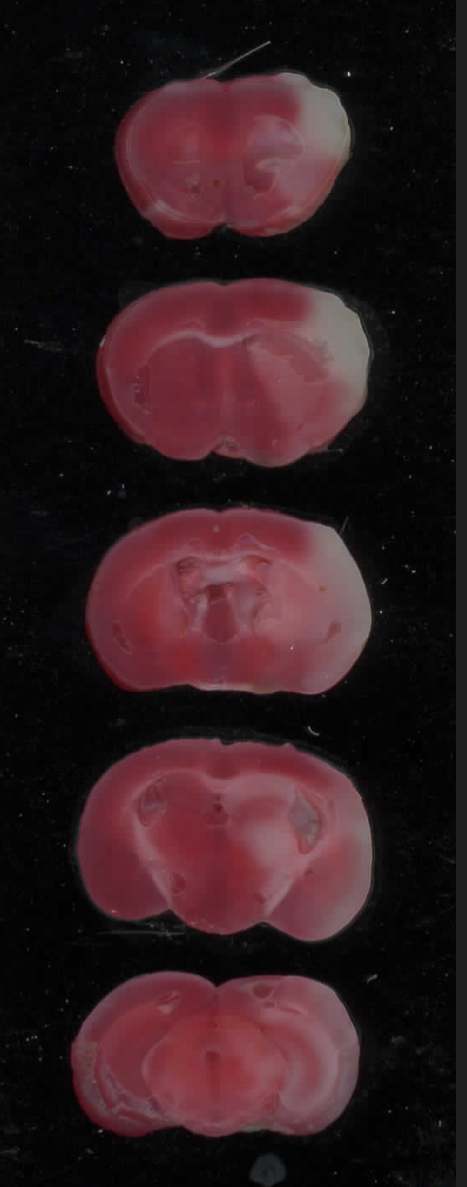

Supplement: Supplementary file 2 [file DataSheet_2.zip › fig 2 raw-D/fig 2-D1 raw/47.png]

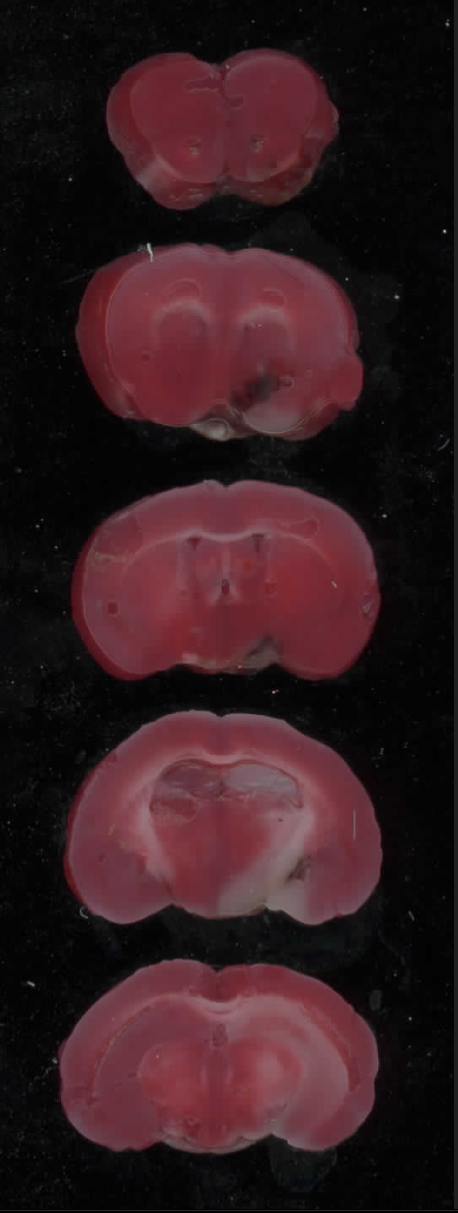

Supplement: Supplementary file 2 [file DataSheet_2.zip › fig 2 raw-D/fig 2-D1 raw/49.png]

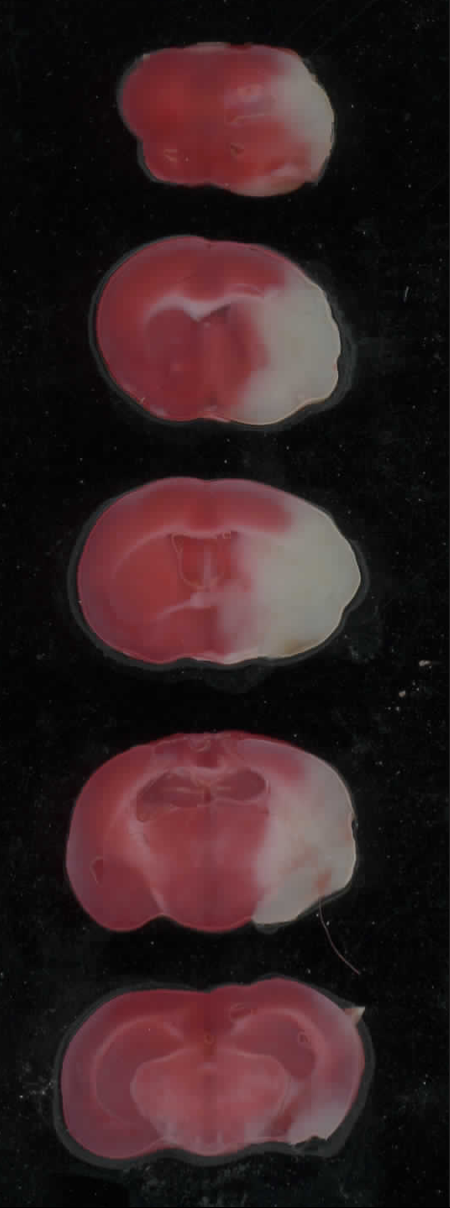

Supplement: Supplementary file 2 [file DataSheet_2.zip › fig 2 raw-D/fig 2-D1 raw/50.png]

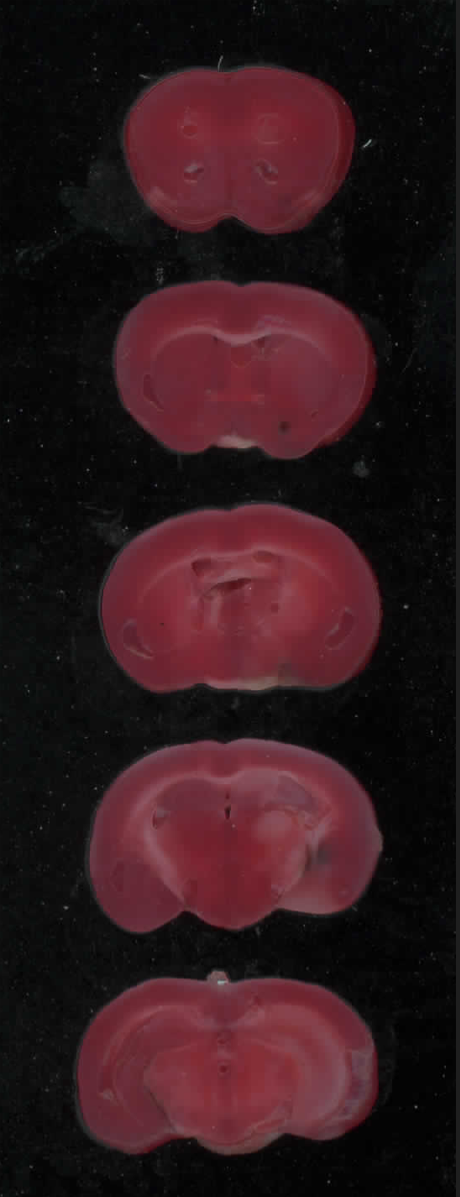

Supplement: Supplementary file 2 [file DataSheet_2.zip › fig 2 raw-D/fig 2-D1 raw/53.png]

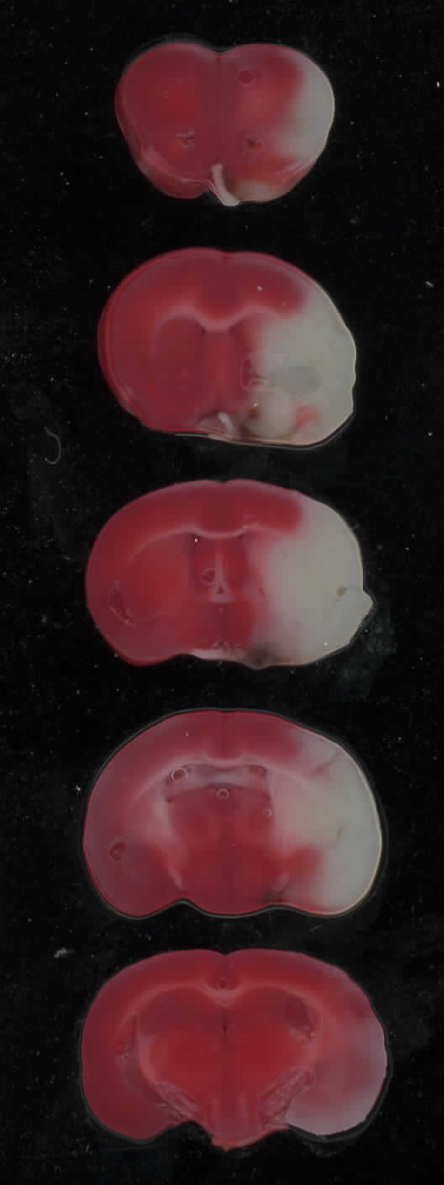

Supplement: Supplementary file 2 [file DataSheet_2.zip › fig 2 raw-D/fig 2-D1 raw/54.png]

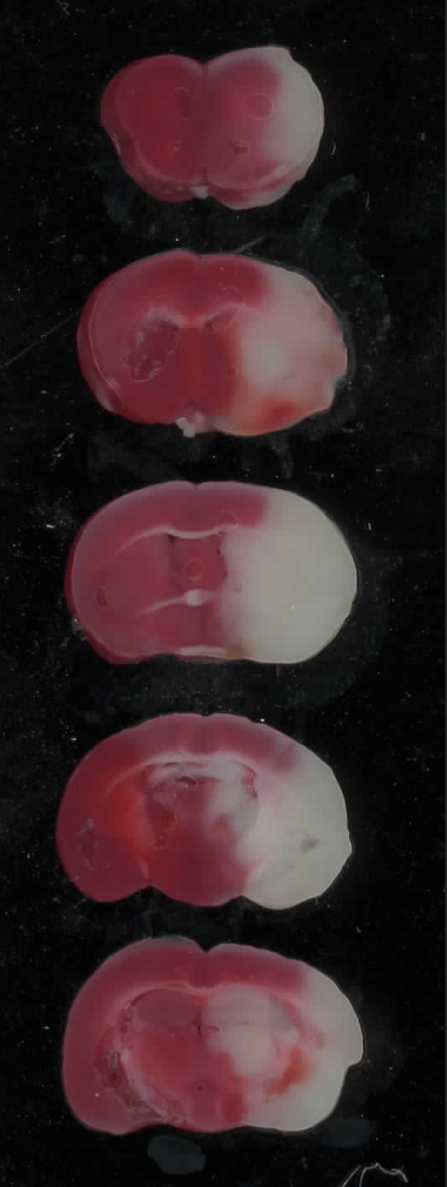

Supplement: Supplementary file 2 [file DataSheet_2.zip › fig 2 raw-D/fig 2-D1 raw/55.png]

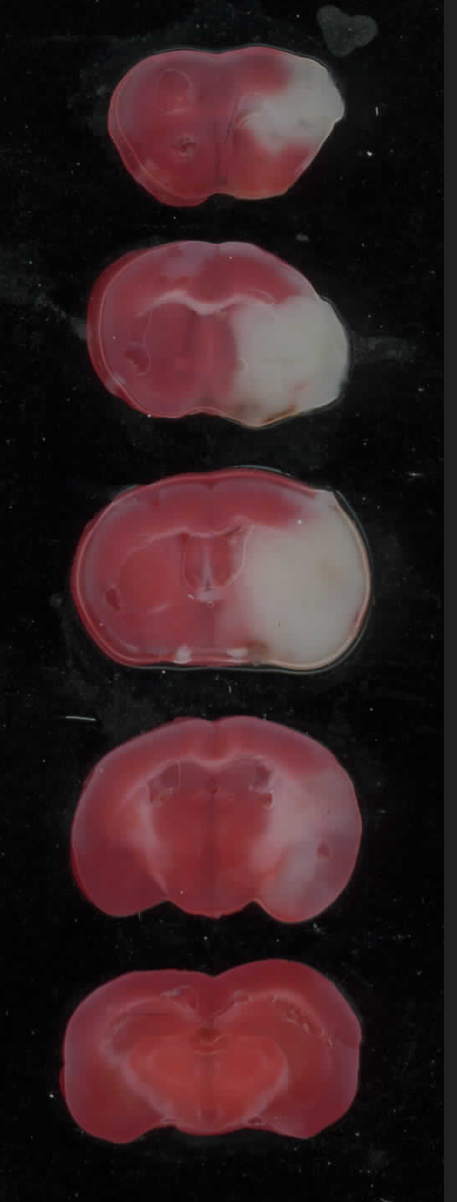

Supplement: Supplementary file 2 [file DataSheet_2.zip › fig 2 raw-D/fig 2-D1 raw/57.png]

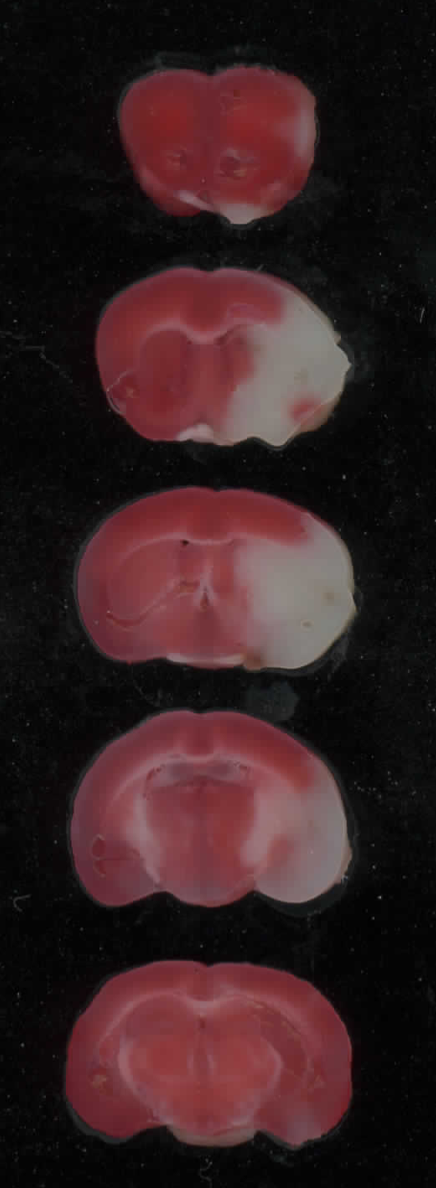

Supplement: Supplementary file 2 [file DataSheet_2.zip › fig 2 raw-D/fig 2-D1 raw/59.png]

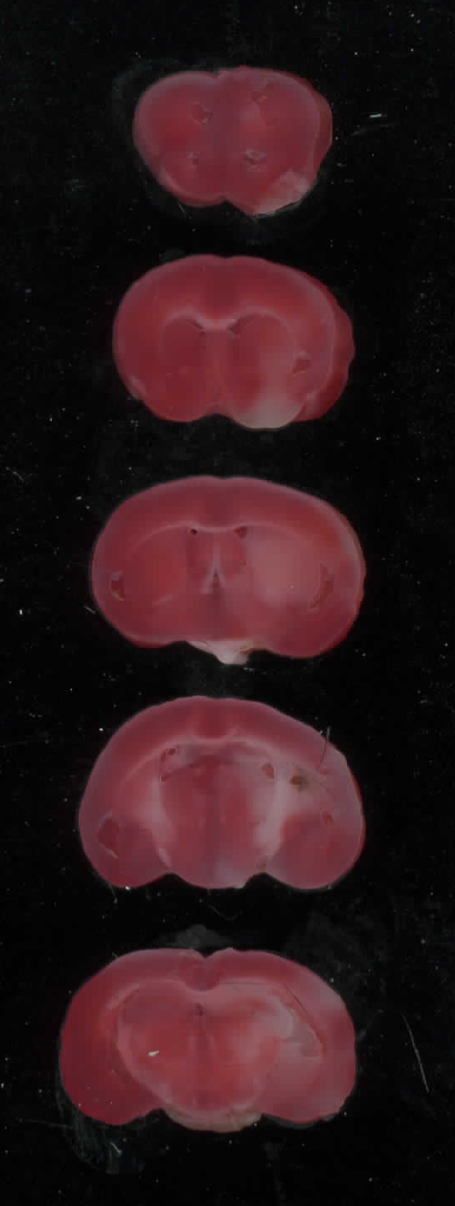

Supplement: Supplementary file 2 [file DataSheet_2.zip › fig 2 raw-D/fig 2-D1 raw/60.png]

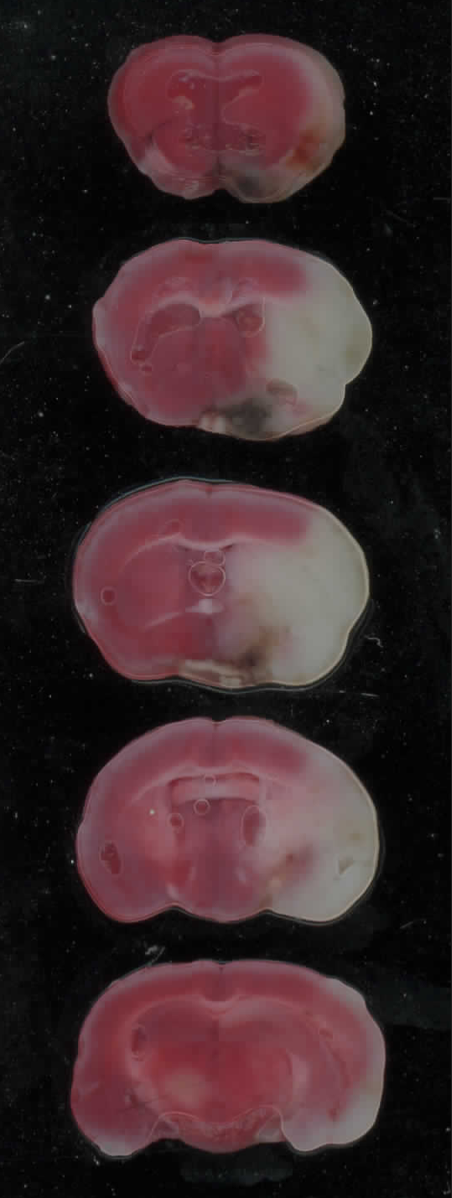

Supplement: Supplementary file 2 [file DataSheet_2.zip › fig 2 raw-D/fig 2-D1 raw/61.png]

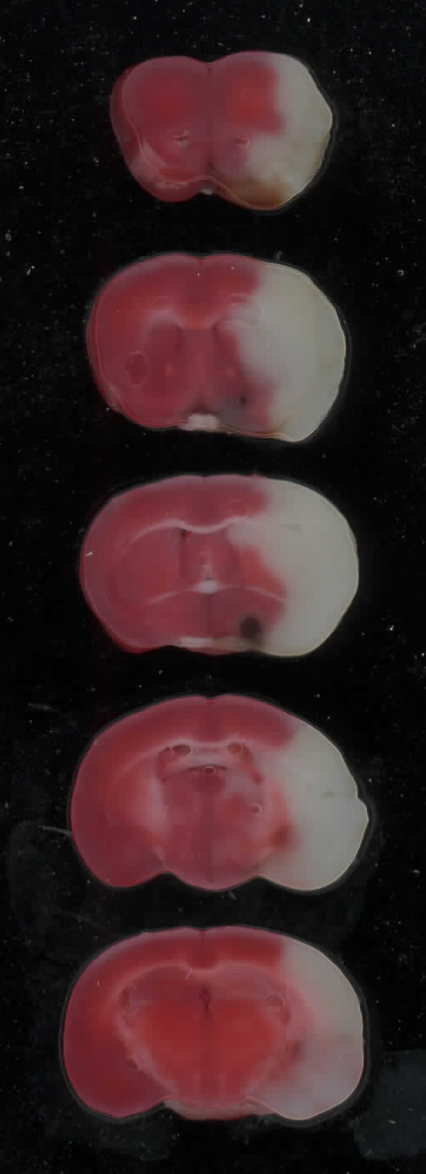

Supplement: Supplementary file 2 [file DataSheet_2.zip › fig 2 raw-D/fig 2-D1 raw/62.png]

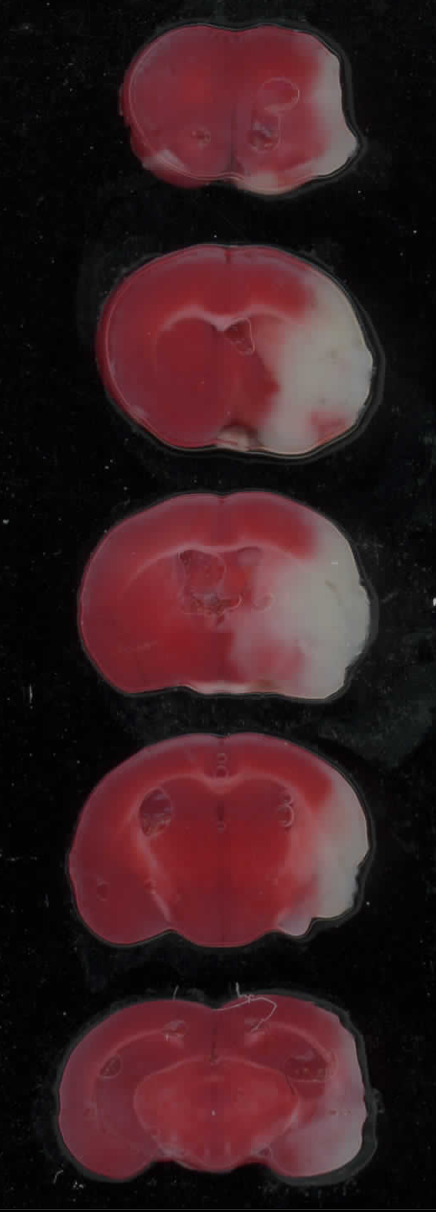

Supplement: Supplementary file 2 [file DataSheet_2.zip › fig 2 raw-D/fig 2-D1 raw/65.png]

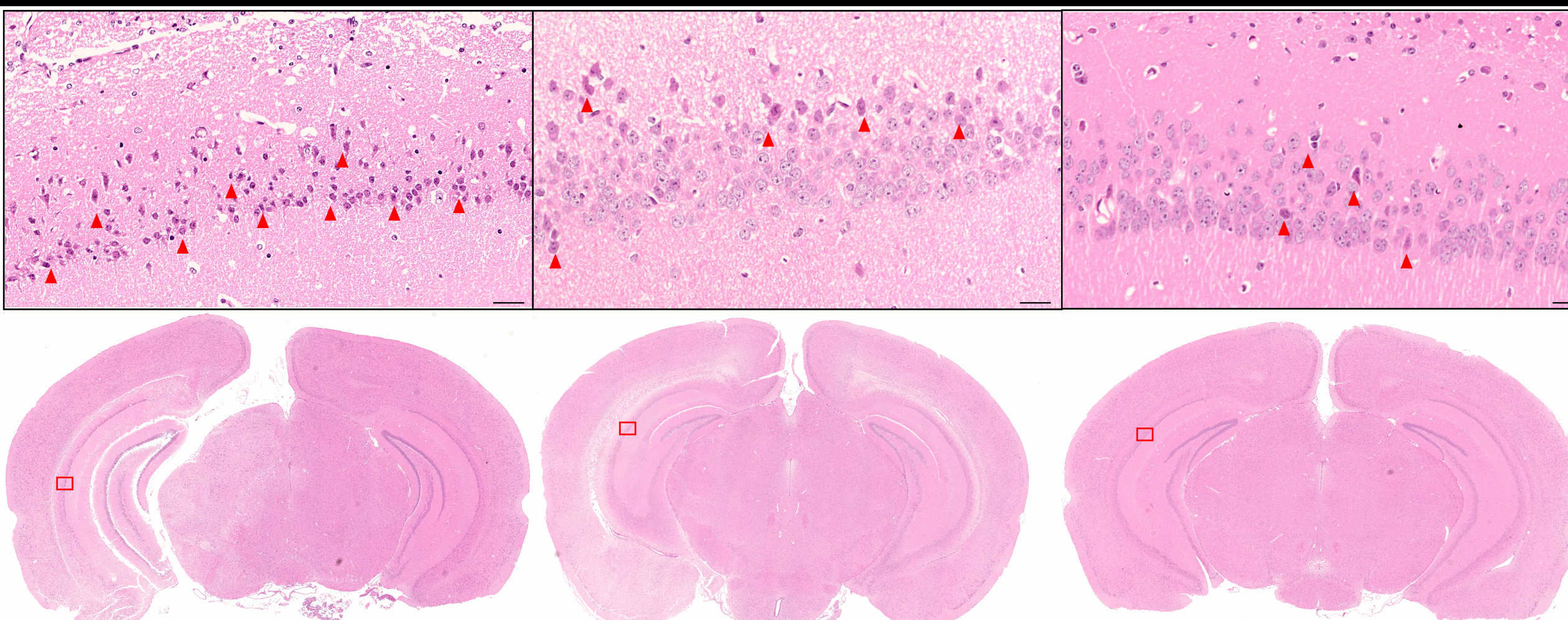

Supplement: Supplementary file 2 [file DataSheet_2.zip › fig 2 raw-E/fig 2-E.png]

fig 3-A sham

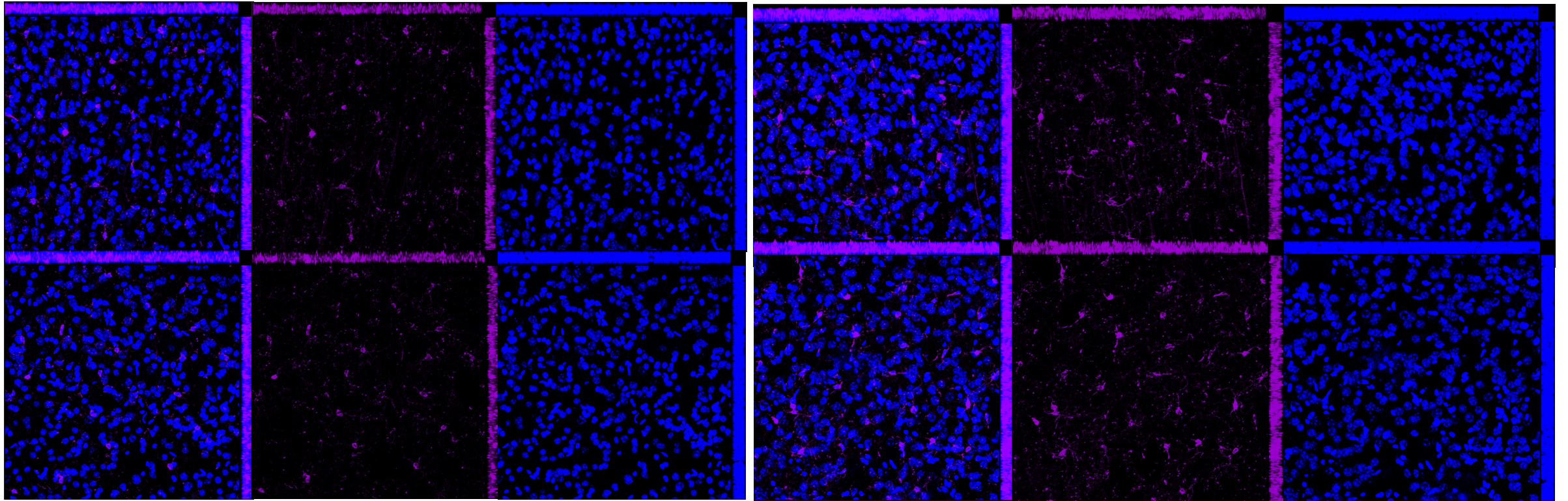

fig 3A Vehicle

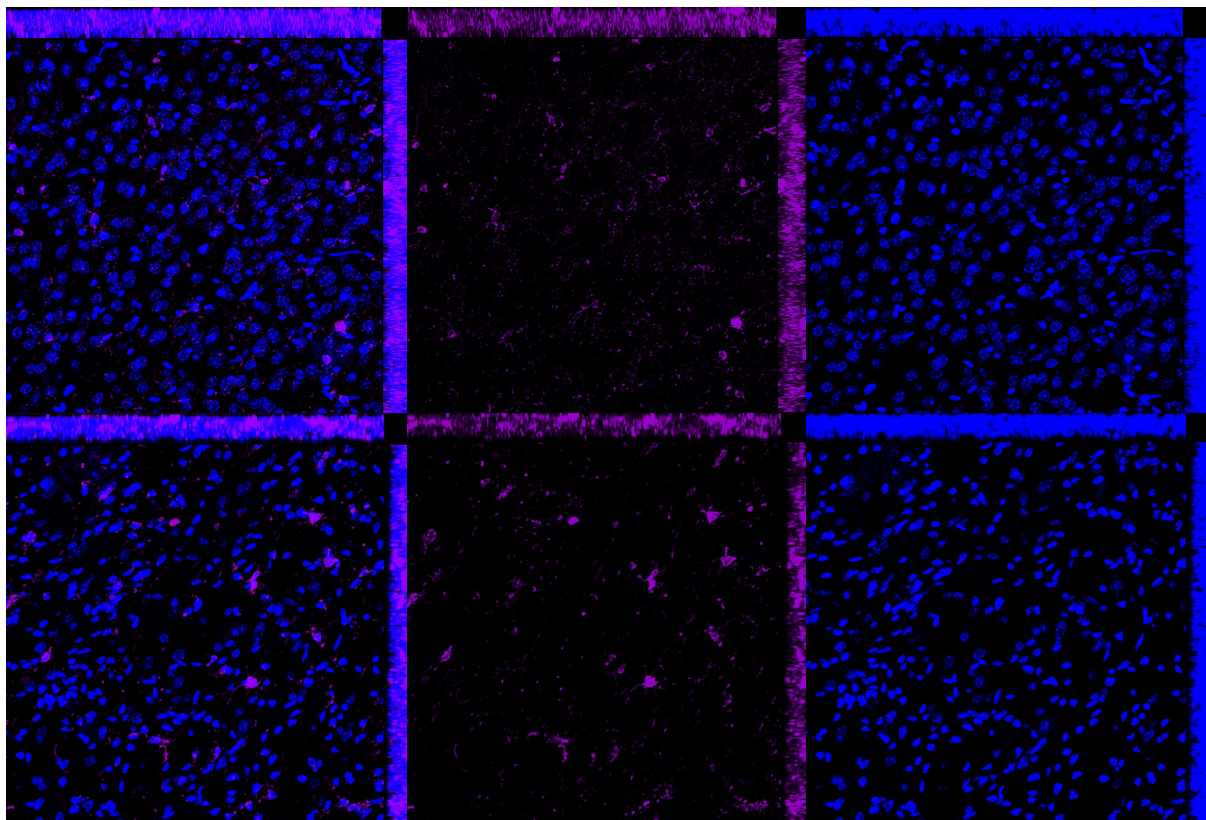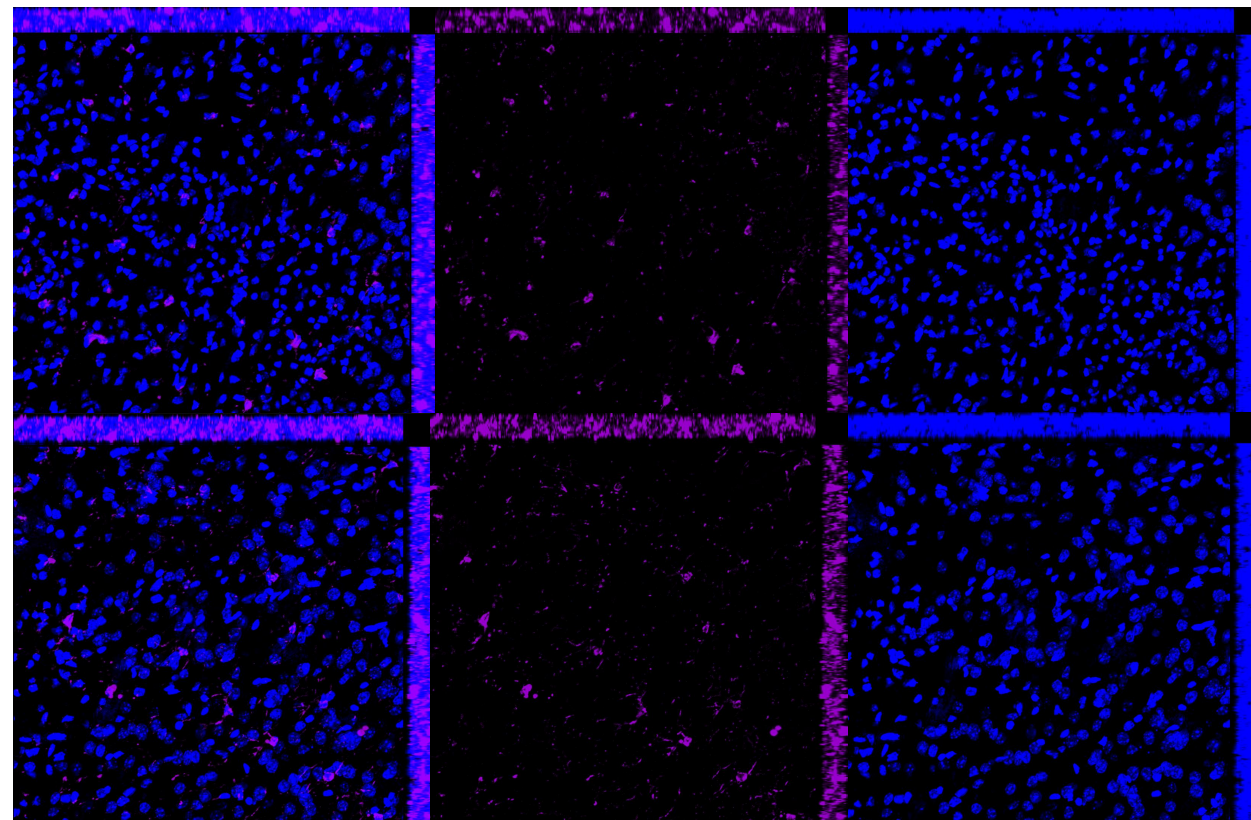

fig 3A Low

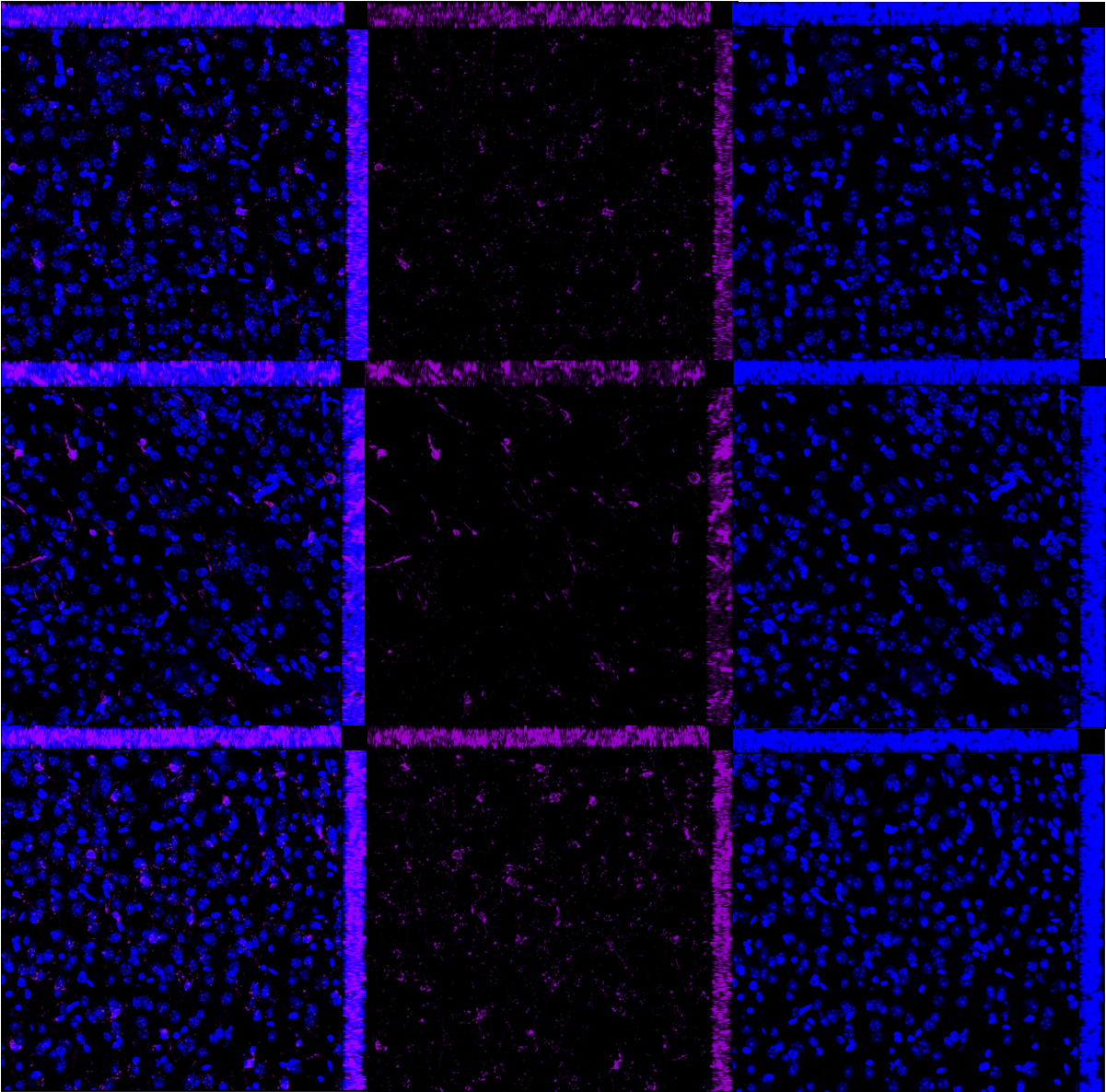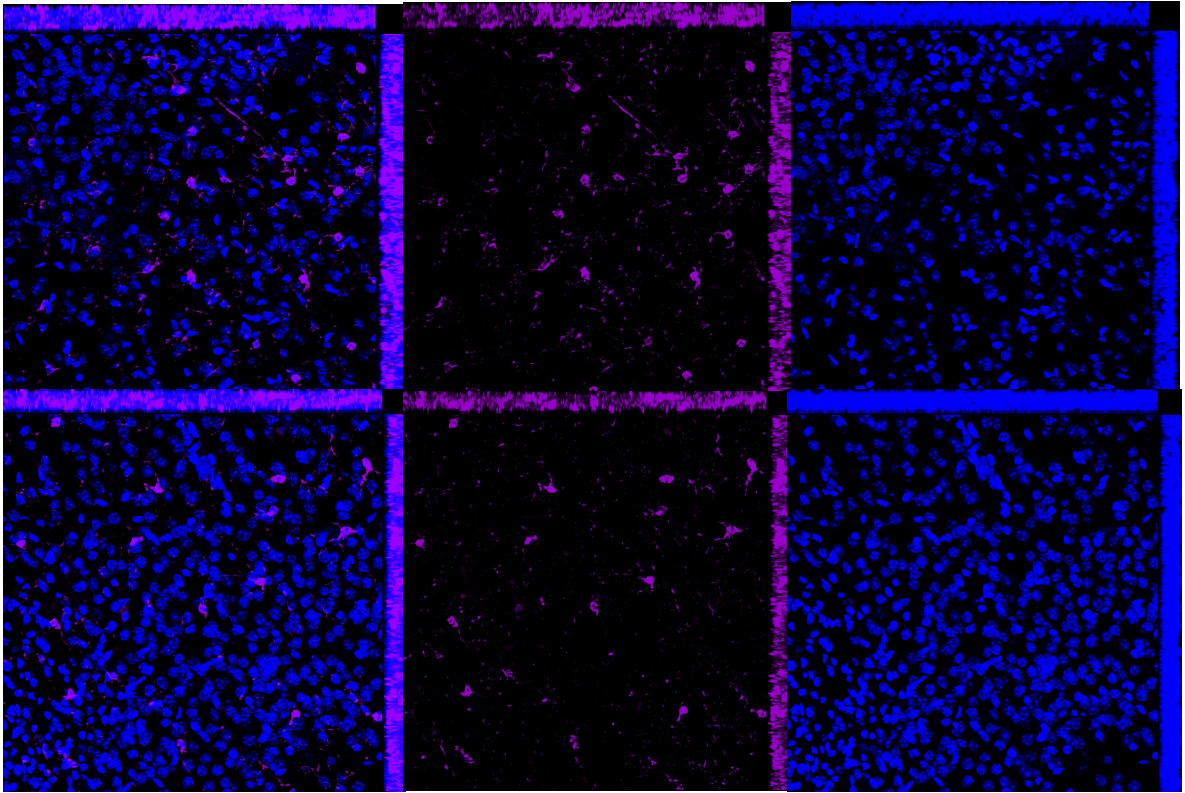

fig 3A High

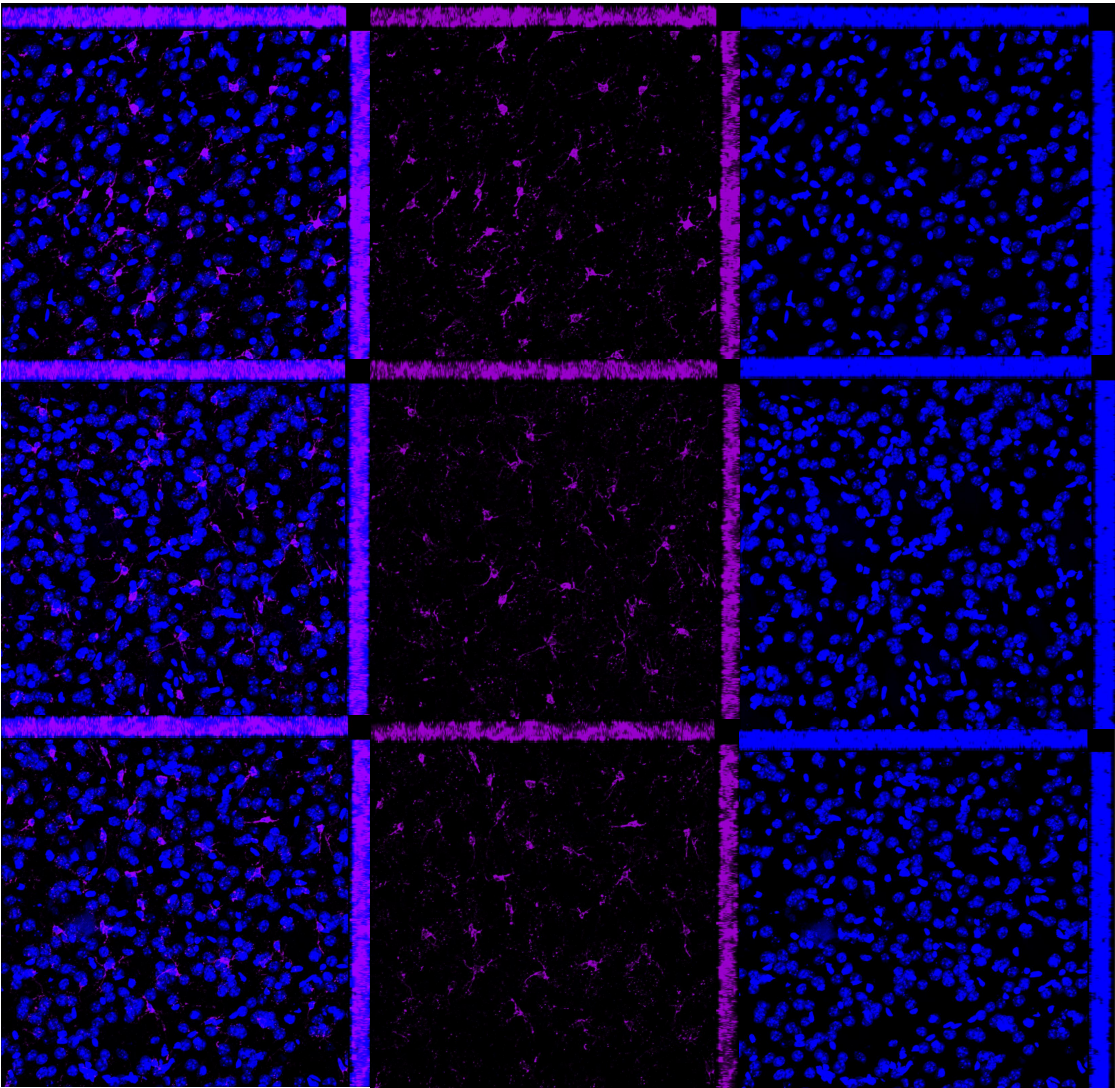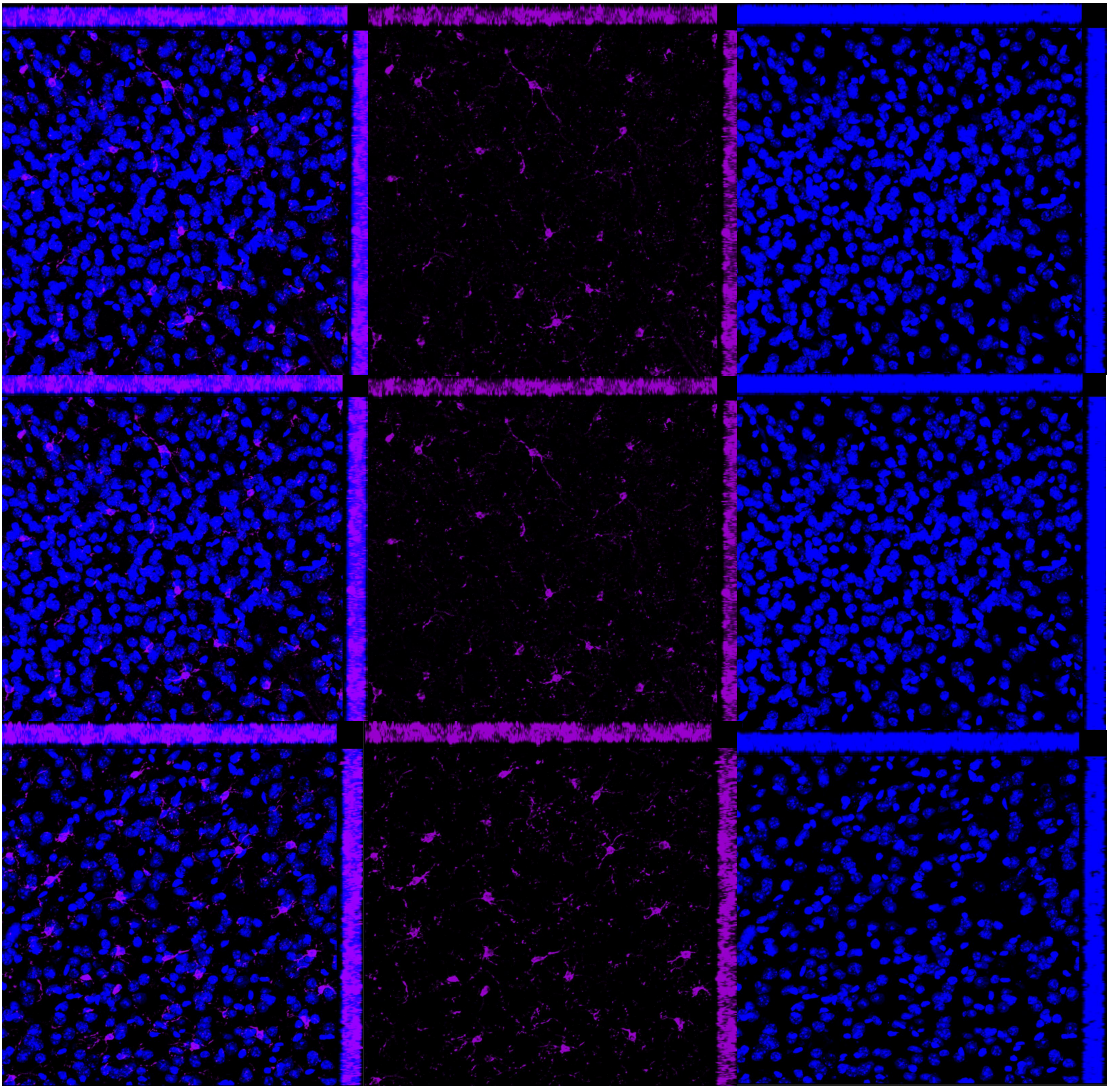

fig 3A High

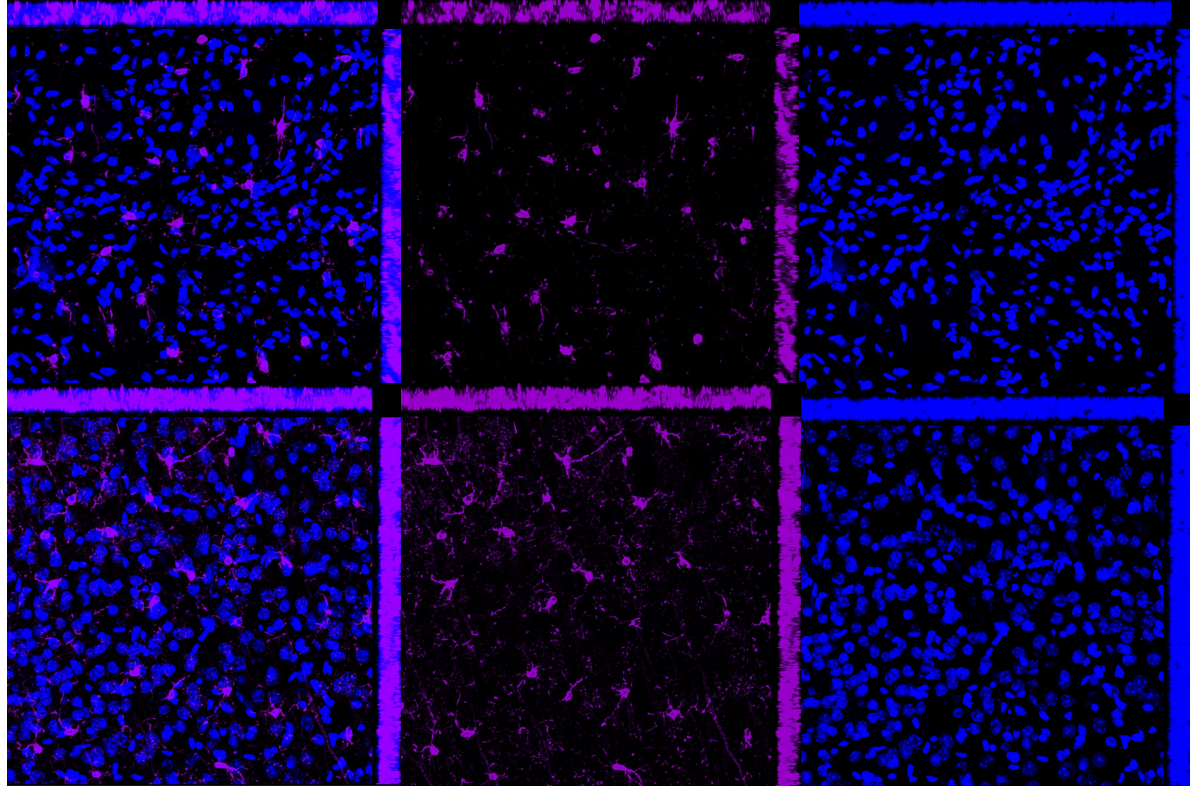

Supplement: Supplementary file 3 [file DataSheet_3.zip › fig 3-A raw/fig 3-A1 raw.pdf]

fig 3-B Vehicle

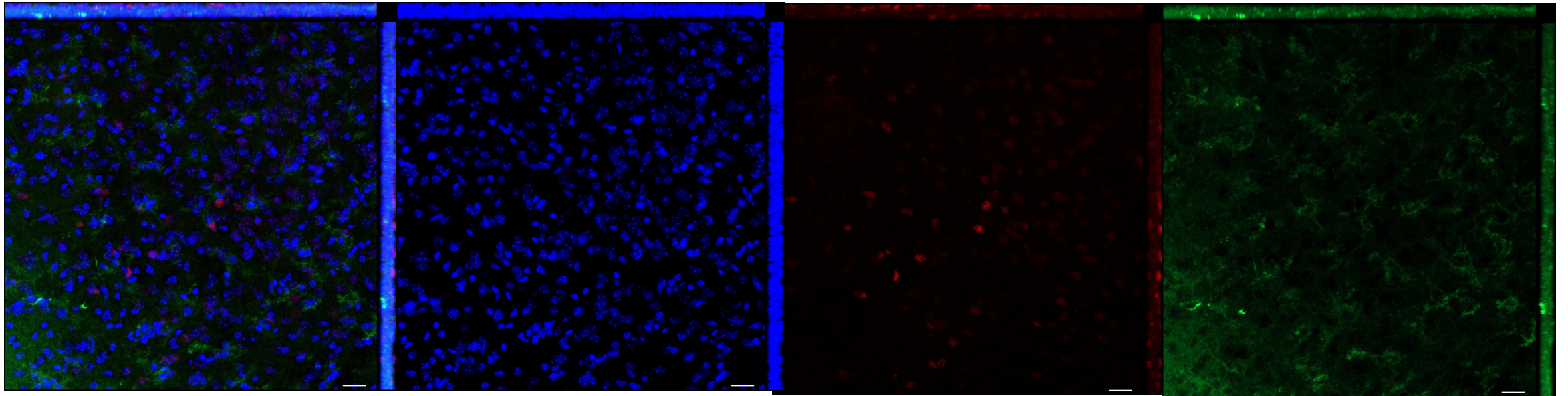

fig 3-B Low

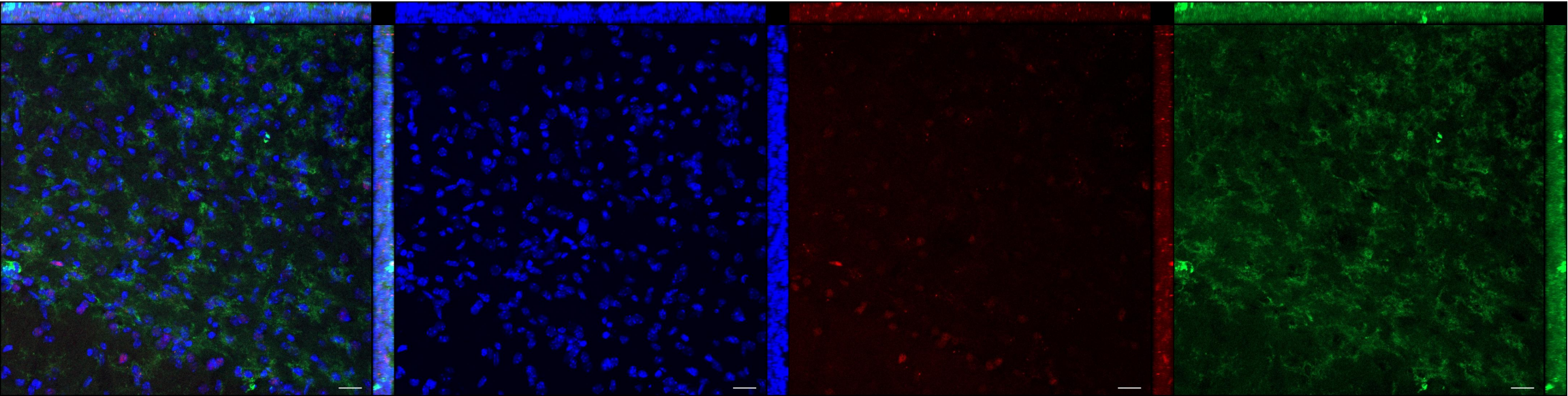

fig 3-B High

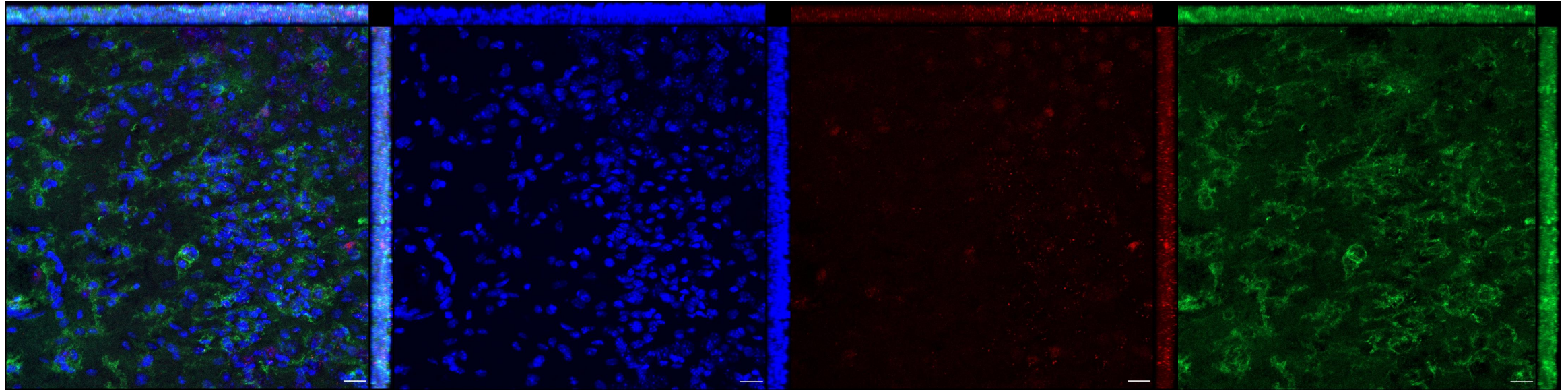

Supplement: Supplementary file 3 [file DataSheet_3.zip › fig 3-B raw/fig 3-B raw.pdf]

fig 4-C control

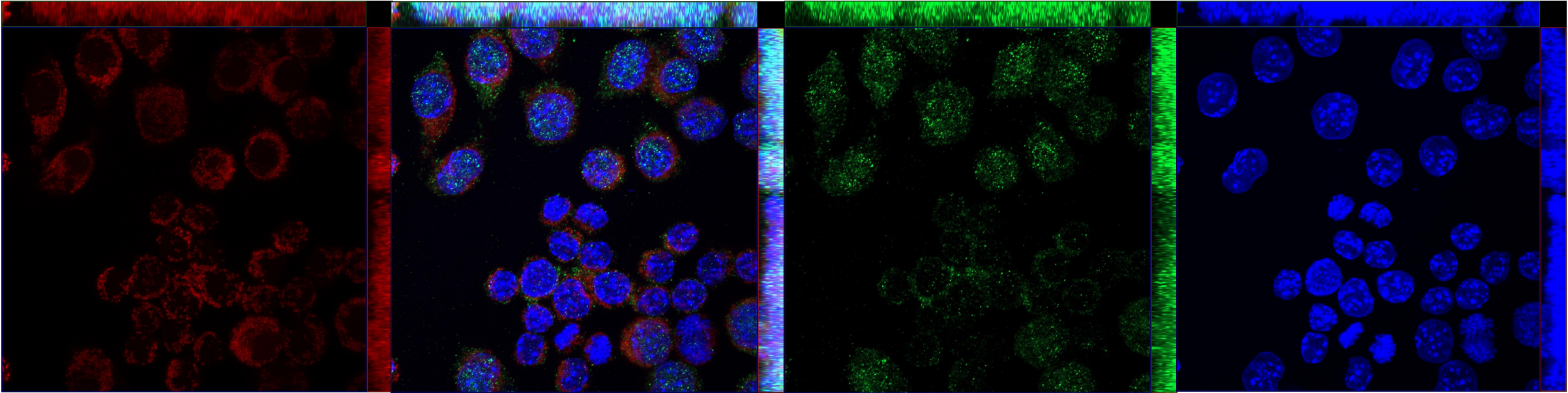

fig 4-C CM

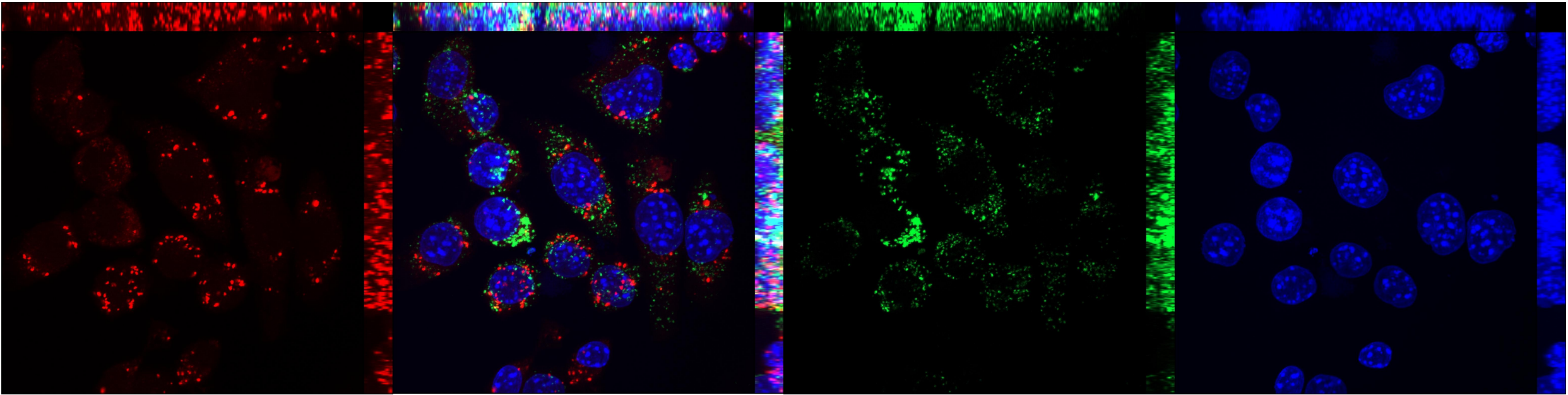

fig 4-C CM+Gs (5  $\mu$ M)

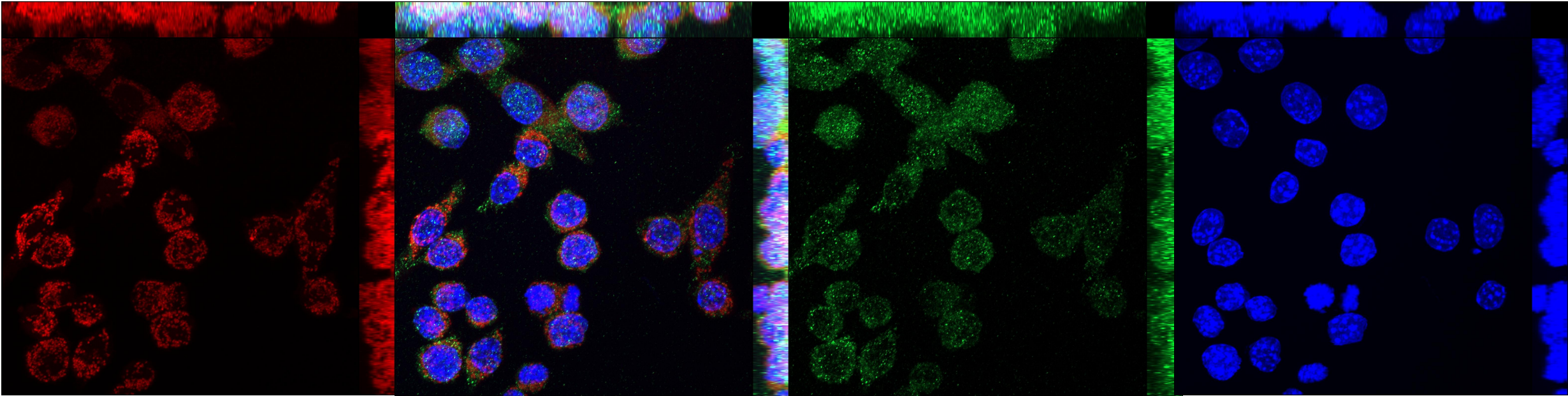

fig 4-C CM+Gs (20  $\mu$ M)

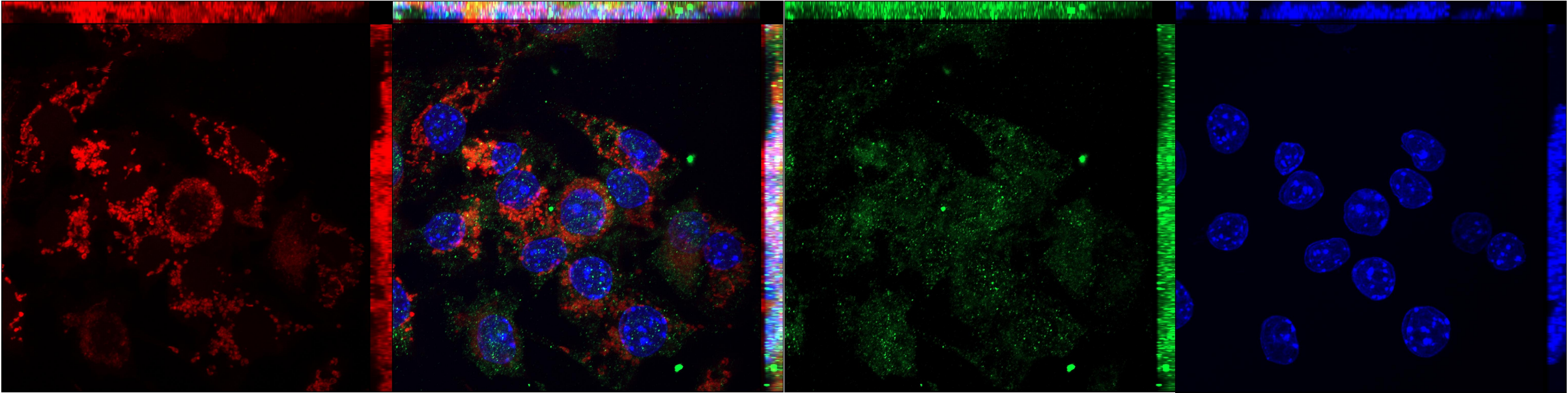

Supplement: Supplementary file 5 [file DataSheet_5.zip › fig 4-C raw/fig 4-C raw.pdf]

treat-vs-model:pValue<0.05&& |log2FC|>1

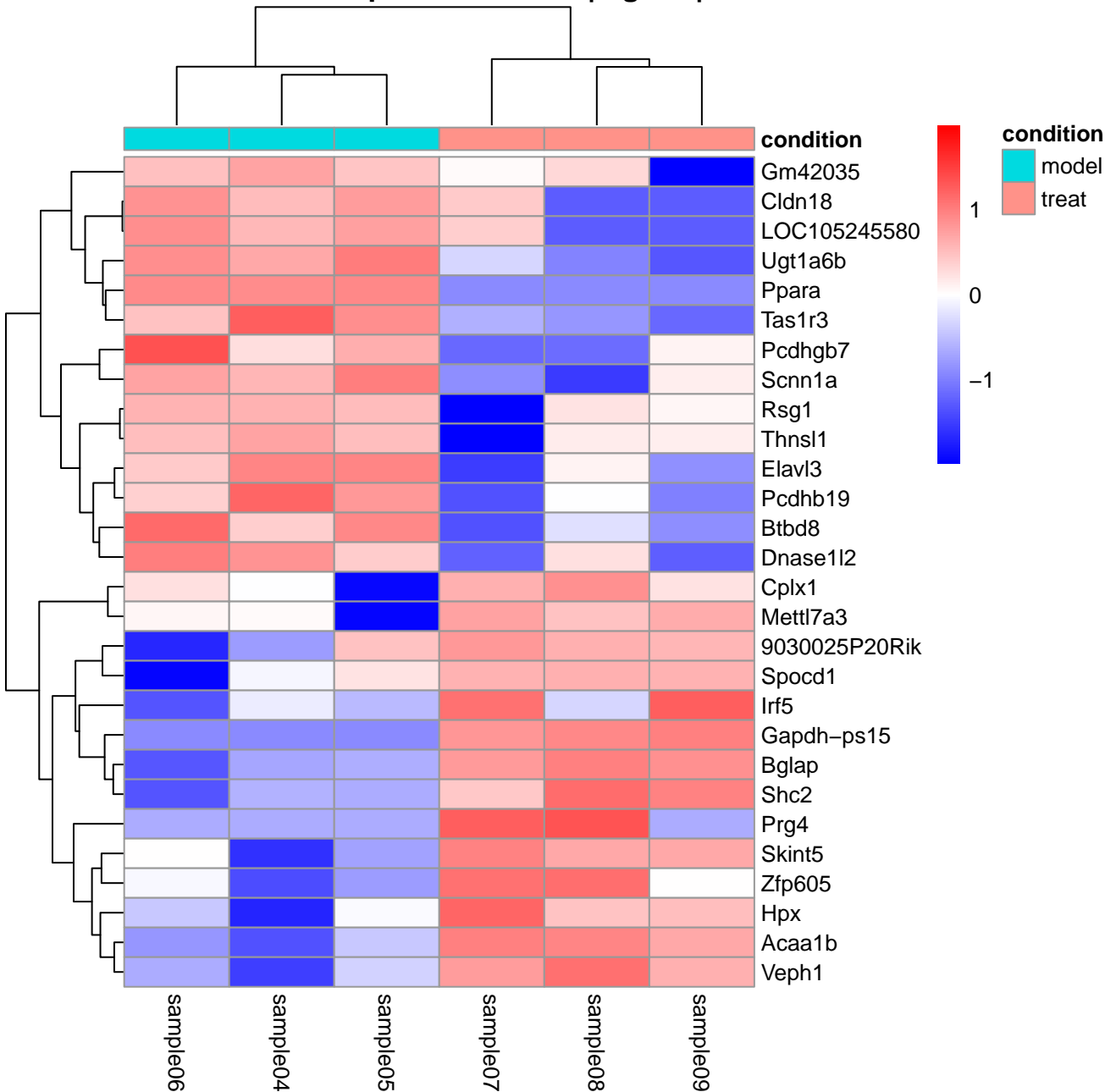

Supplement: Supplementary file 6 [file DataSheet_6.zip › fig 5 raw/fig 5-A B raw/treat-vs-model-heatmap-pval-0.05-FC-2.gene.pdf]

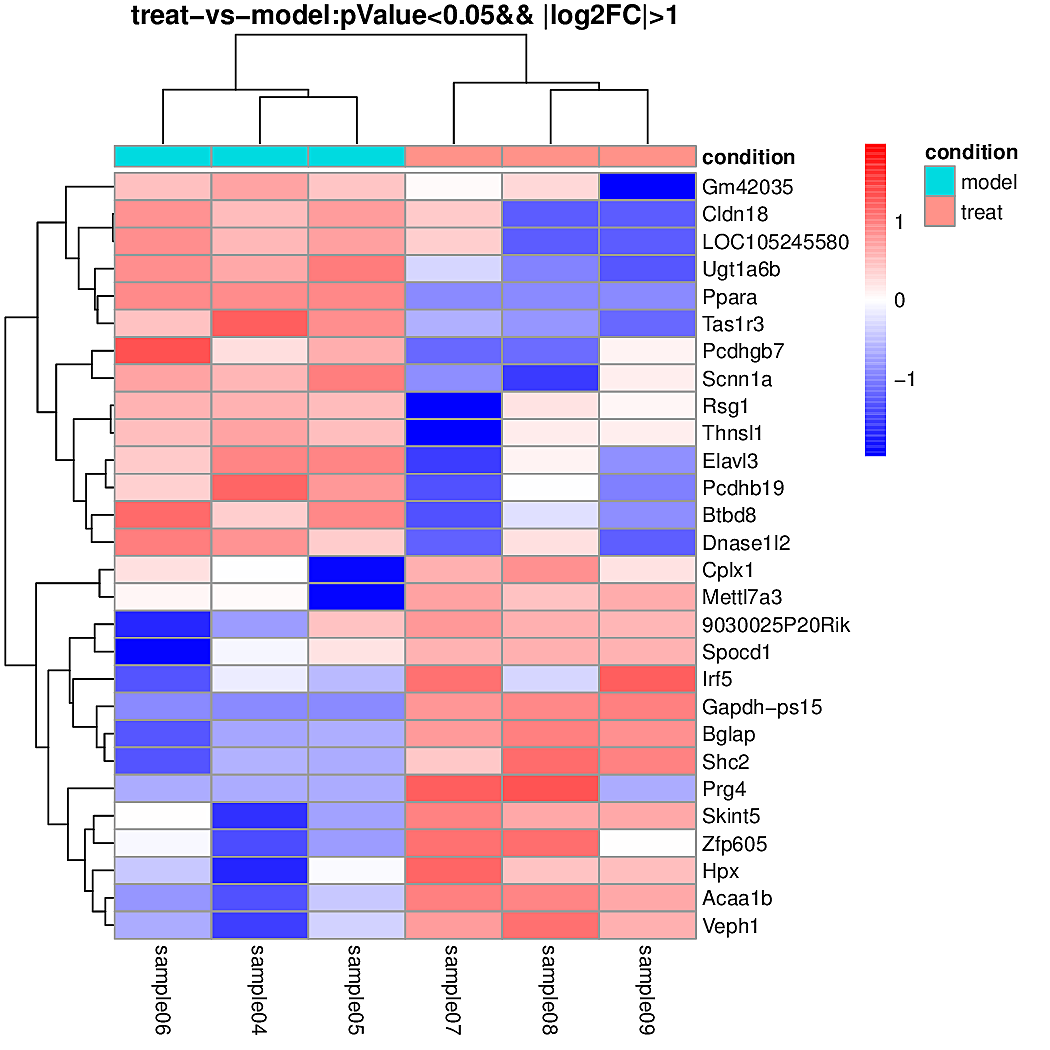

Supplement: Supplementary file 6 [file DataSheet_6.zip › fig 5 raw/fig 5-A B raw/treat-vs-model-heatmap-pval-0.05-FC-2.gene.png]

treat -vs- model : pValue < 0.05 && |log2FC|> 1

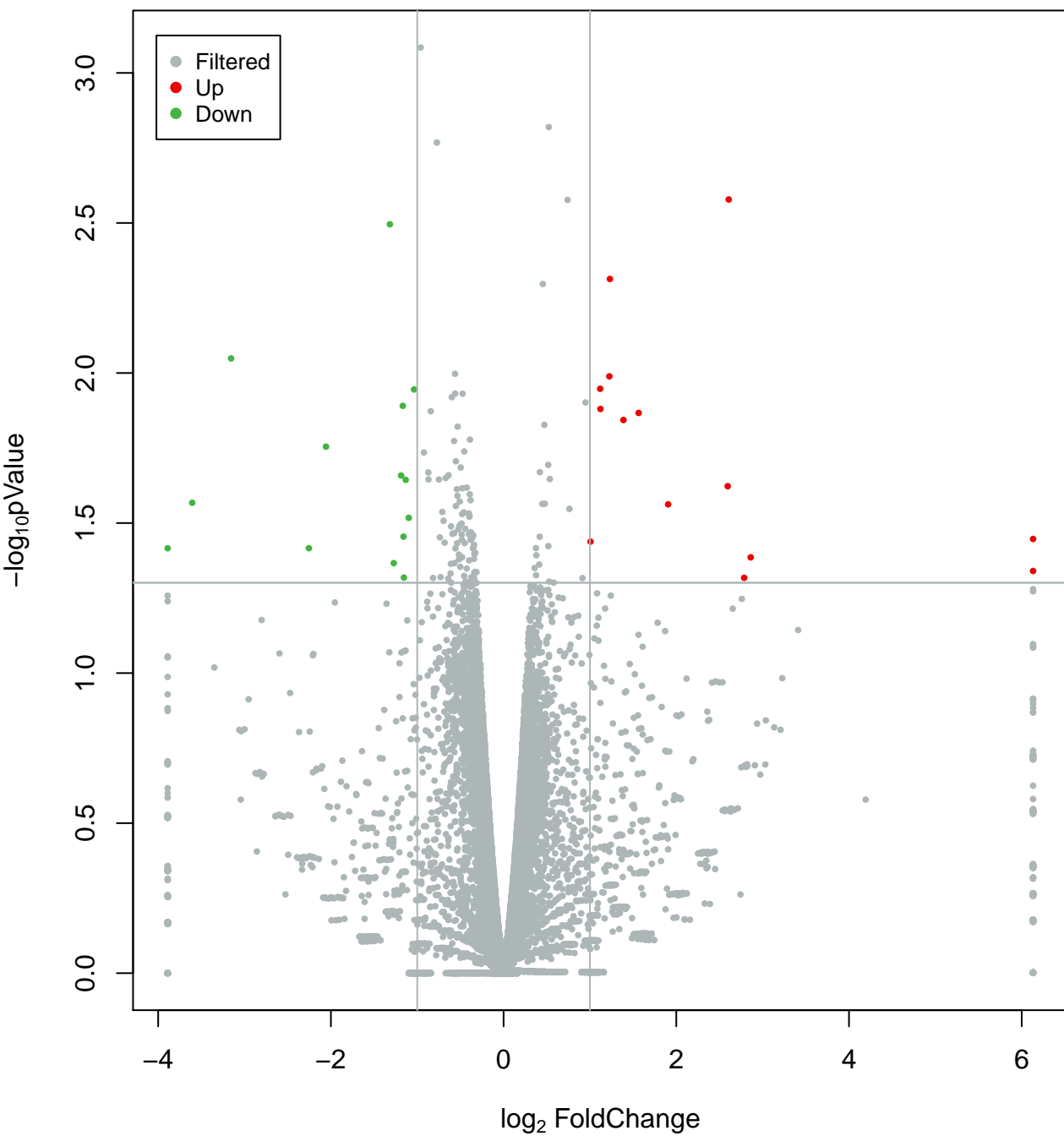

Supplement: Supplementary file 6 [file DataSheet_6.zip › fig 5 raw/fig 5-A B raw/treat-vs-model-volcano-pval-0.05-FC-2.gene.pdf]

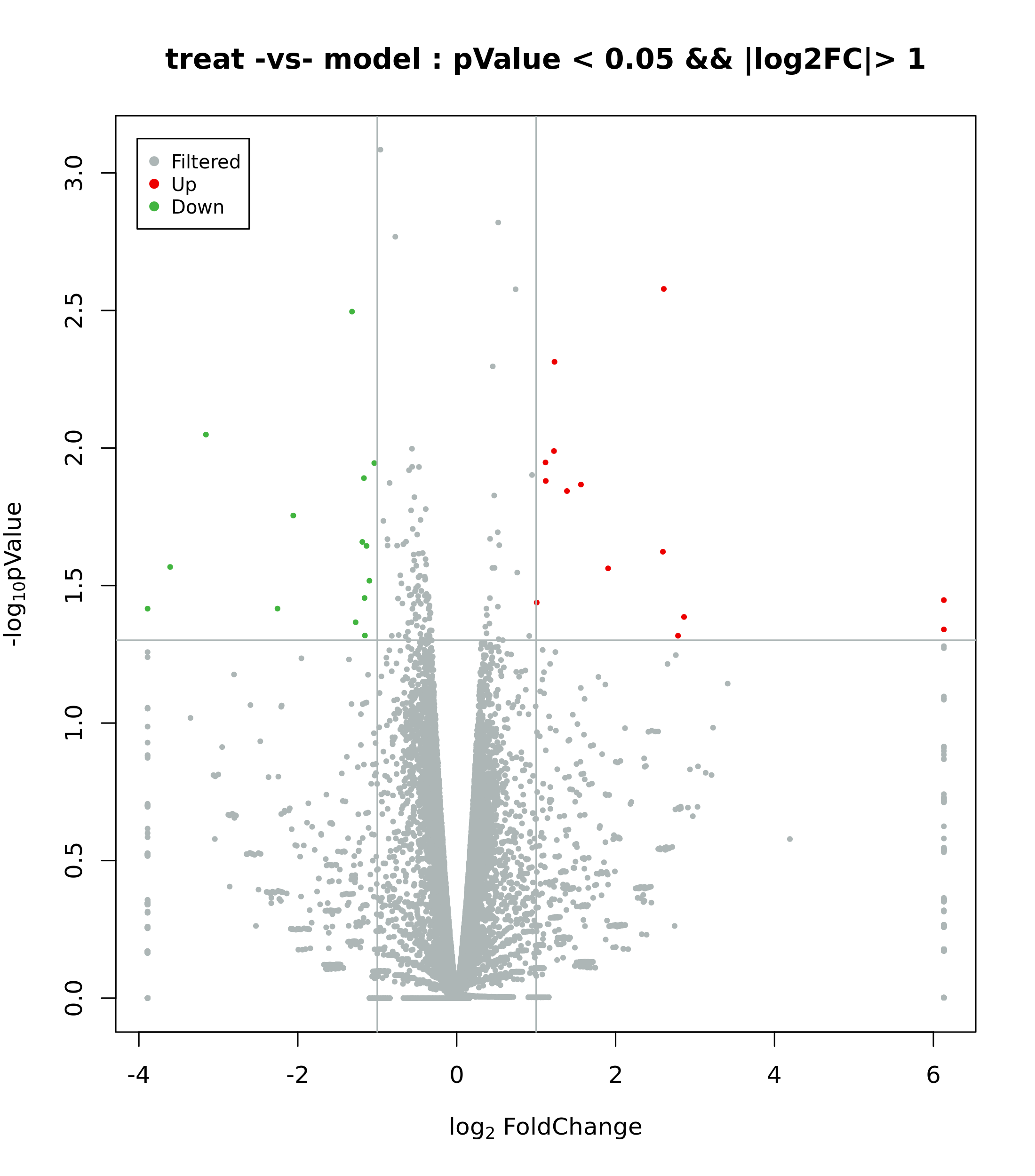

Supplement: Supplementary file 6 [file DataSheet_6.zip › fig 5 raw/fig 5-A B raw/treat-vs-model-volcano-pval-0.05-FC-2.gene.png]

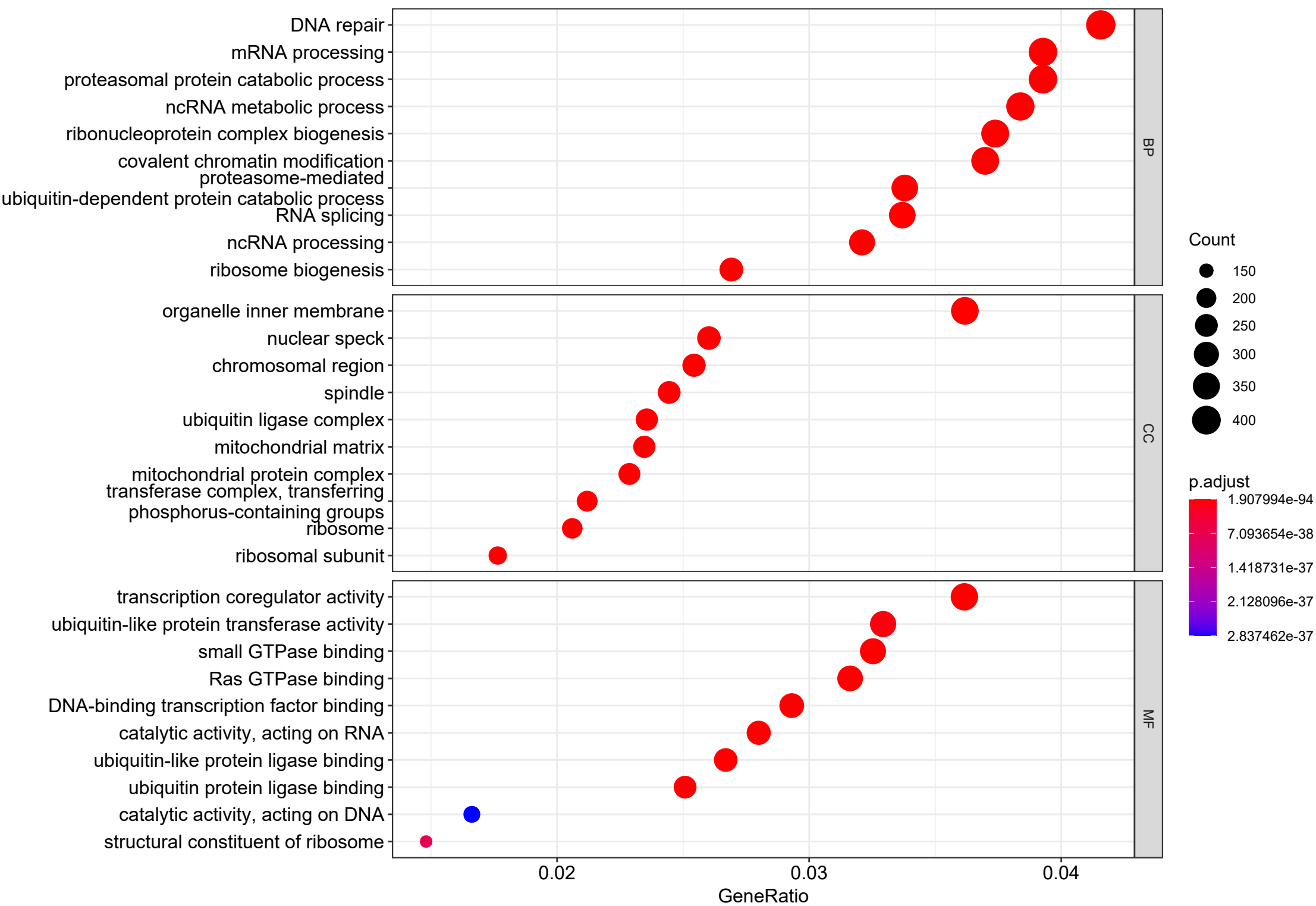

Supplement: Supplementary file 6 [file DataSheet_6.zip › fig 5 raw/fig 5-C D raw/MCAO_vs_drug_edgeR-GO-plot.pdf]

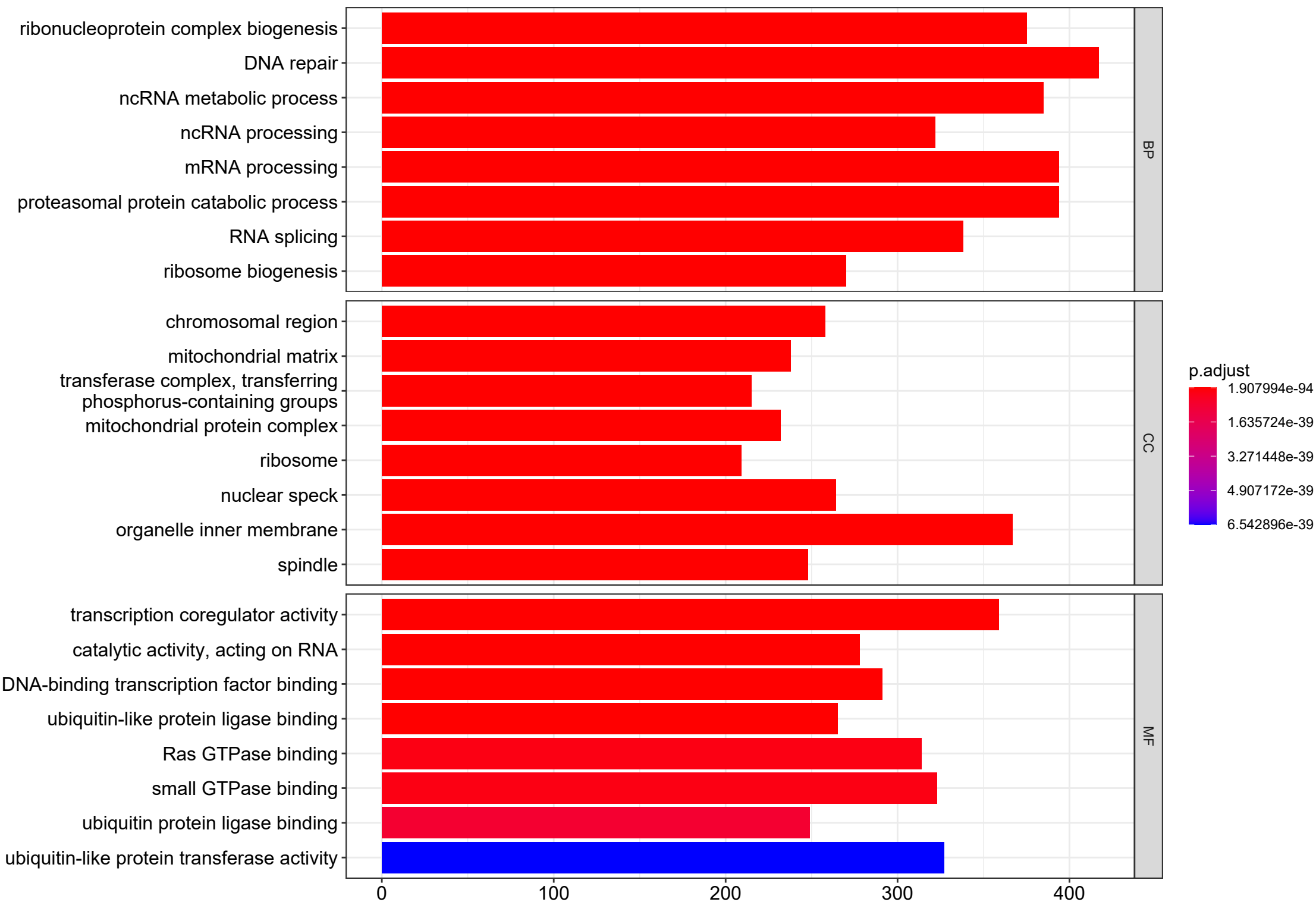

Supplement: Supplementary file 6 [file DataSheet_6.zip › fig 5 raw/fig 5-C D raw/MCAO_vs_drug_edgeR-GO.pdf]

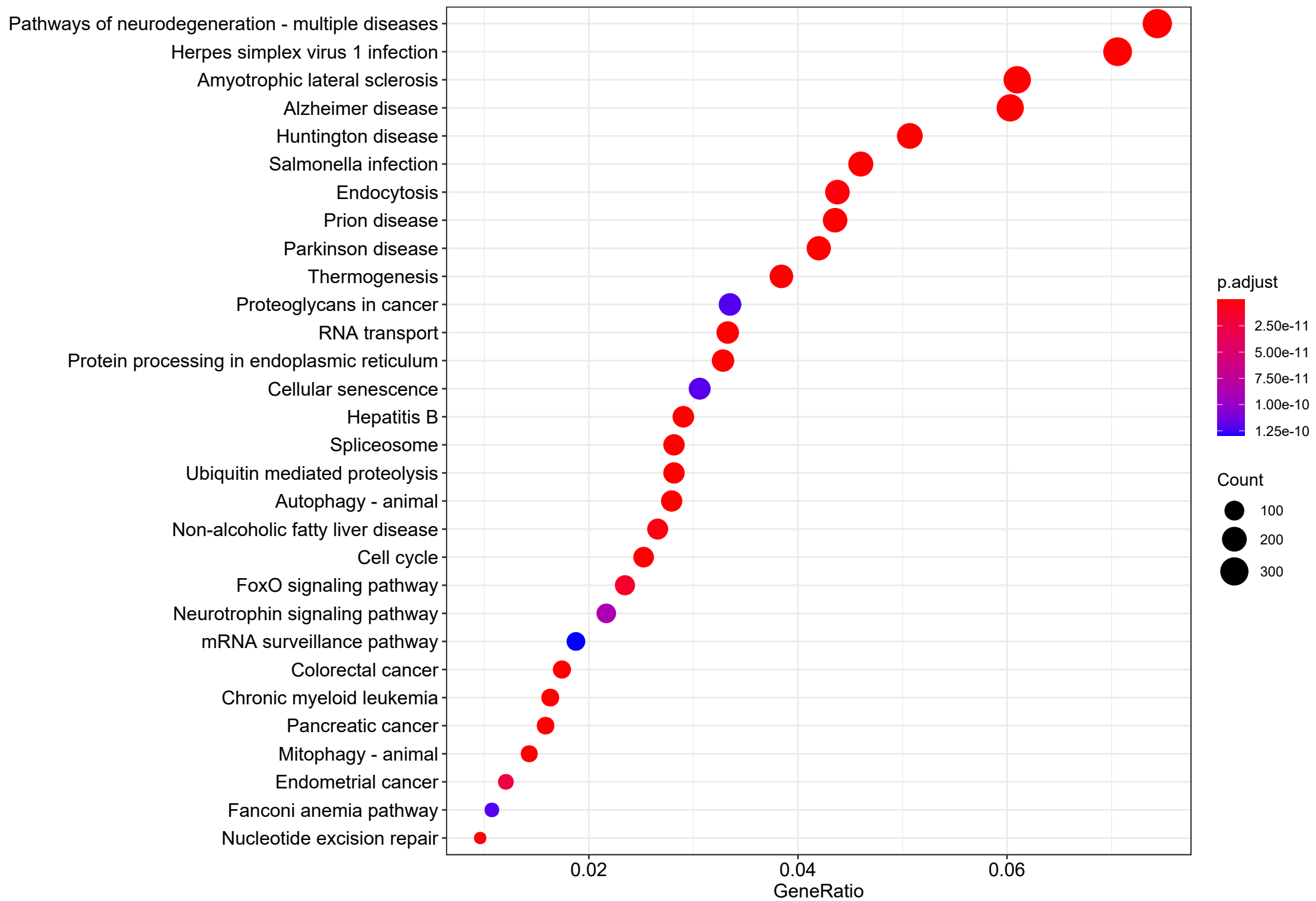

Supplement: Supplementary file 6 [file DataSheet_6.zip › fig 5 raw/fig 5-C D raw/MCAO_vs_drug_edgeR-KEGG-plot.pdf]

# KEGG Pathway

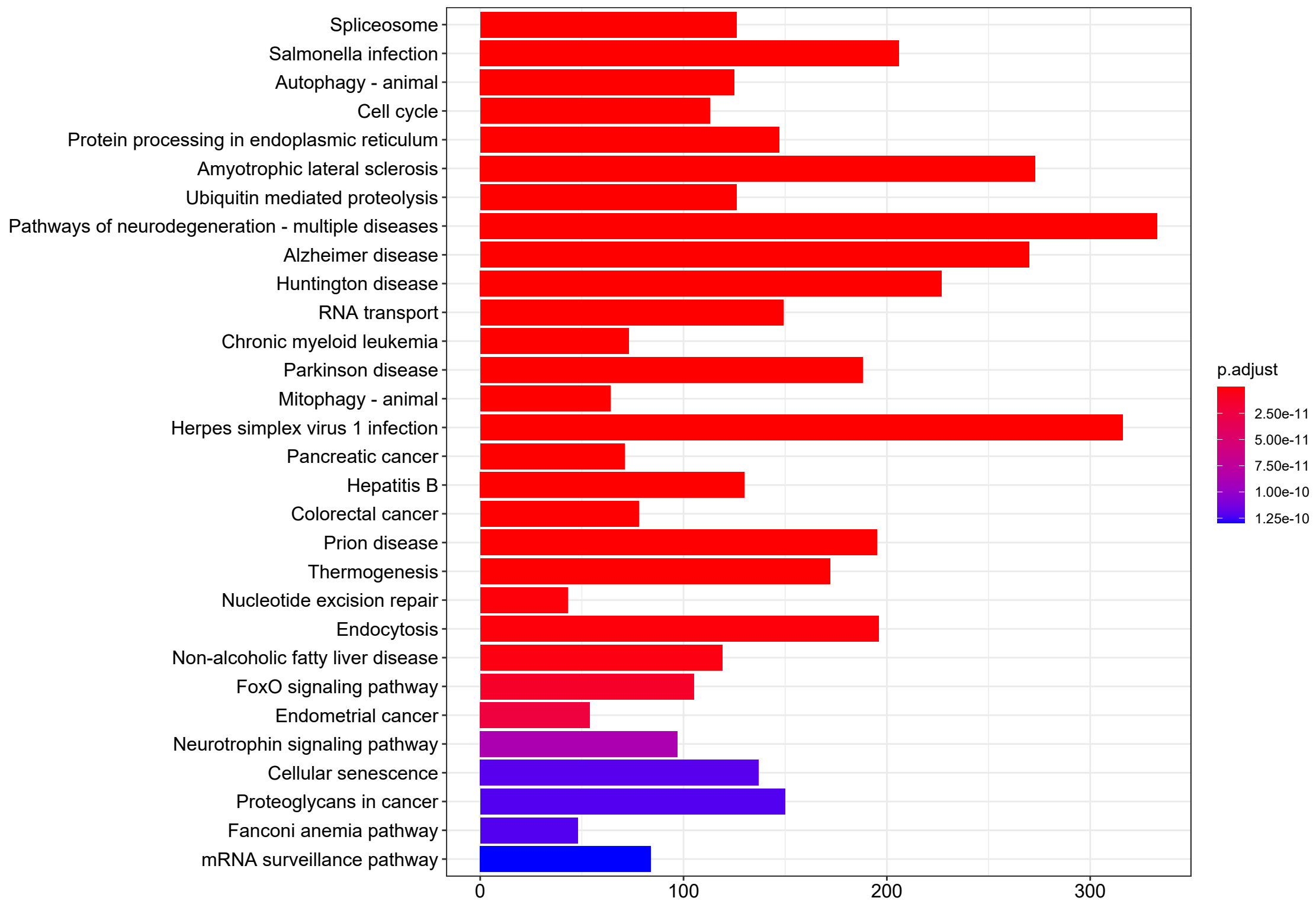

Supplement: Supplementary file 6 [file DataSheet_6.zip › fig 5 raw/fig 5-C D raw/MCAO_vs_drug_edgeR-KEGG.pdf]

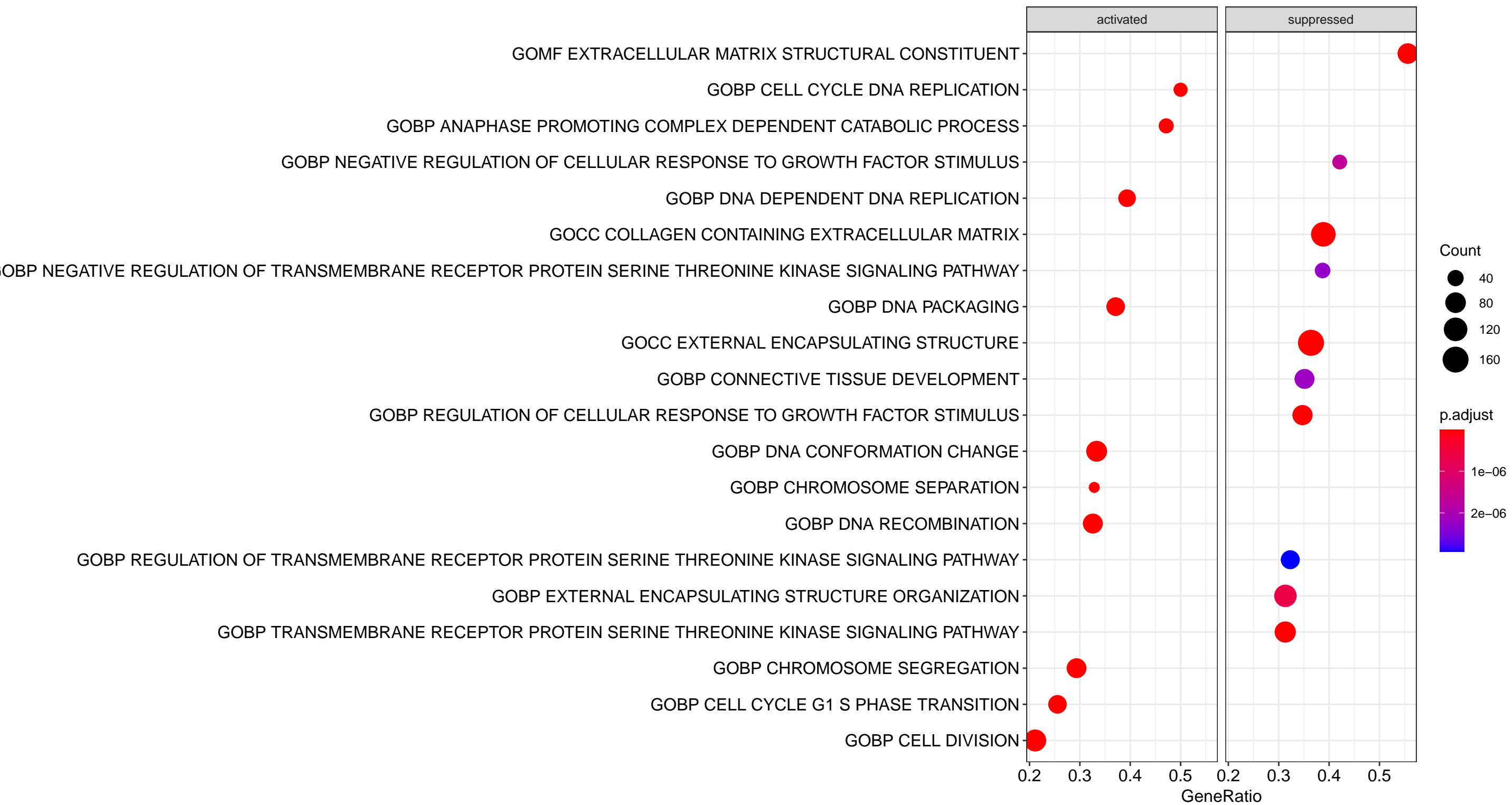

Supplement: Supplementary file 6 [file DataSheet_6.zip › fig 5 raw/fig 5-E F raw/GSEA-GO-2.pdf]

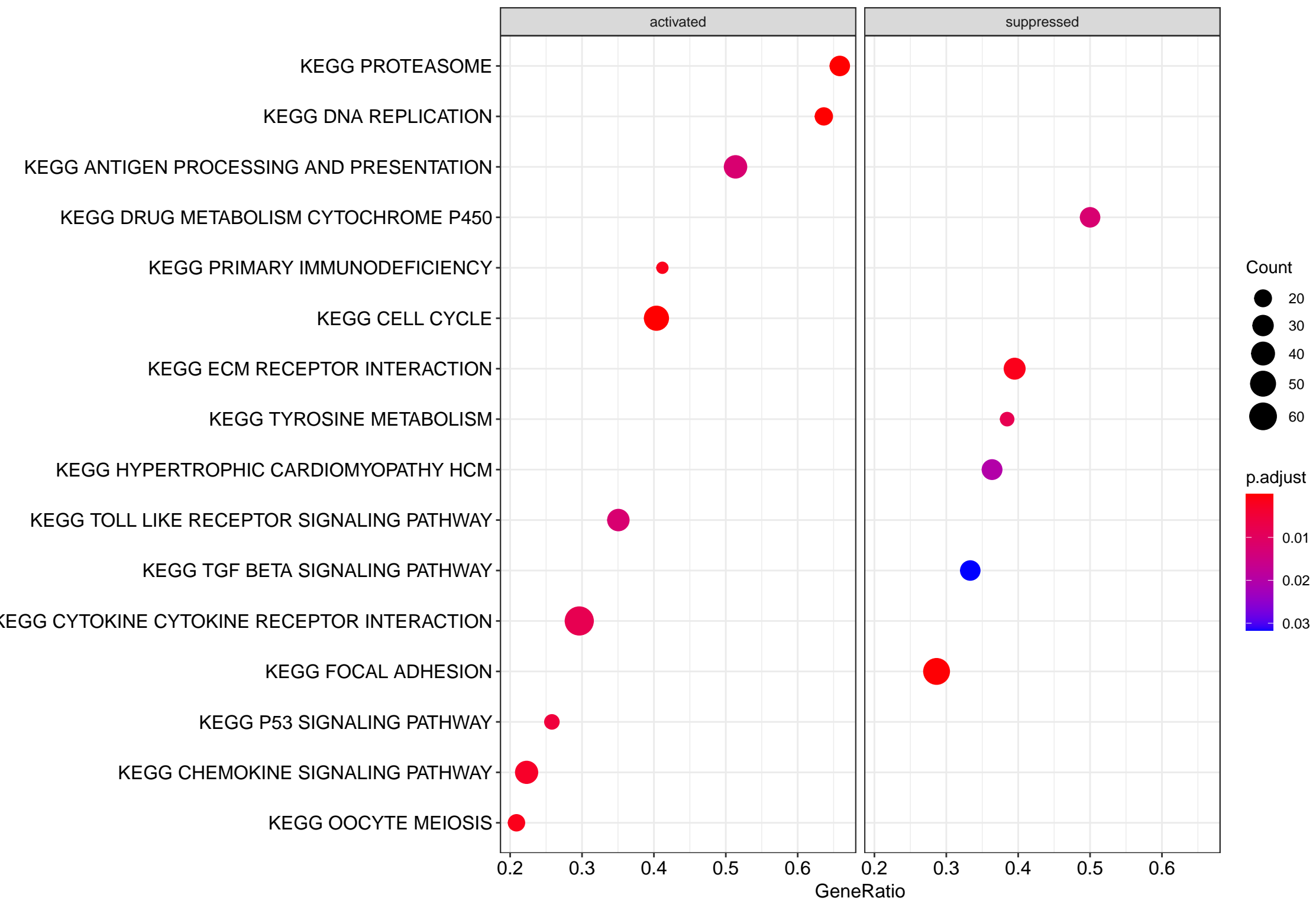

Supplement: Supplementary file 6 [file DataSheet_6.zip › fig 5 raw/fig 5-E F raw/GSEA-KEGG2.pdf]

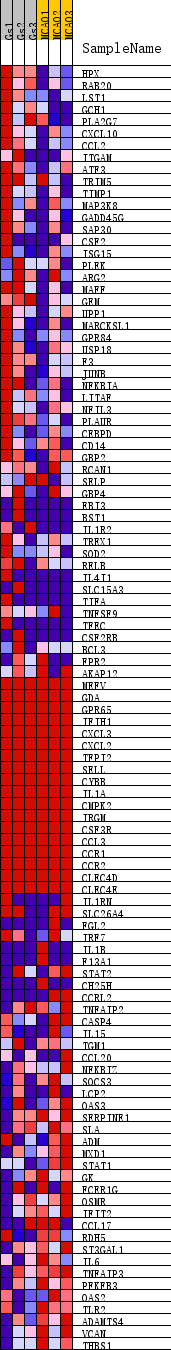

Supplement: Supplementary file 6 [file DataSheet_6.zip › fig 5 raw/fig 5-G raw/inflammation.Gsea.1649955013530/ALTEMEIER_RESPONSE_TO_LPS_WITH_MECHANICAL_VENTILATION_126.png]

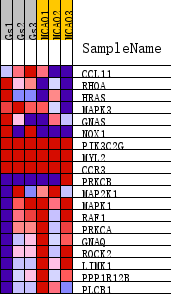

Supplement: Supplementary file 6 [file DataSheet_6.zip › fig 5 raw/fig 5-G raw/inflammation.Gsea.1649955013530/BIOCARTA_CCR3_PATHWAY_150.png]

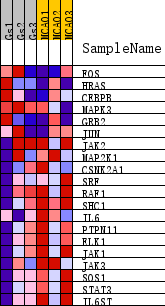

Supplement: Supplementary file 6 [file DataSheet_6.zip › fig 5 raw/fig 5-G raw/inflammation.Gsea.1649955013530/BIOCARTA_IL6_PATHWAY_147.png]

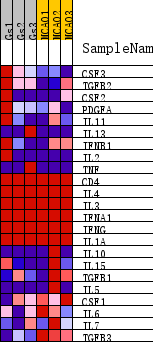

Supplement: Supplementary file 6 [file DataSheet_6.zip › fig 5 raw/fig 5-G raw/inflammation.Gsea.1649955013530/BIOCARTA_INFLAM_PATHWAY_135.png]

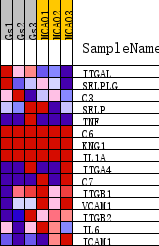

Supplement: Supplementary file 6 [file DataSheet_6.zip › fig 5 raw/fig 5-G raw/inflammation.Gsea.1649955013530/BIOCARTA_LAIR_PATHWAY_138.png]

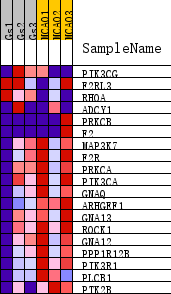

Supplement: Supplementary file 6 [file DataSheet_6.zip › fig 5 raw/fig 5-G raw/inflammation.Gsea.1649955013530/BIOCARTA_PAR1_PATHWAY_144.png]

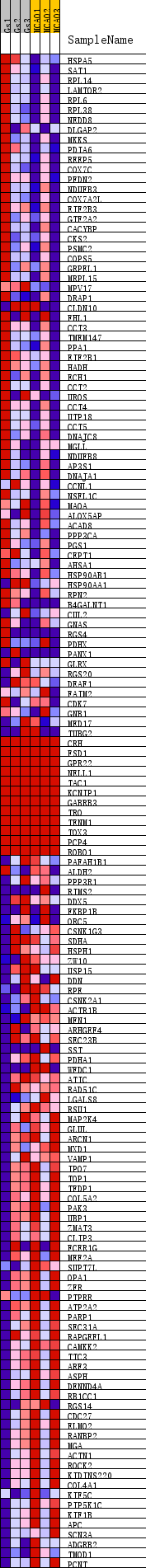

Supplement: Supplementary file 6 [file DataSheet_6.zip › fig 5 raw/fig 5-G raw/inflammation.Gsea.1649955013530/BLALOCK_ALZHEIMERS_DISEASE_INCIPIENT_DN_153.png]

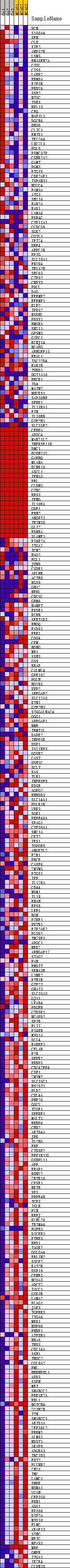

Supplement: Supplementary file 6 [file DataSheet_6.zip › fig 5 raw/fig 5-G raw/inflammation.Gsea.1649955013530/BLALOCK_ALZHEIMERS_DISEASE_INCIPIENT_UP_141.png]

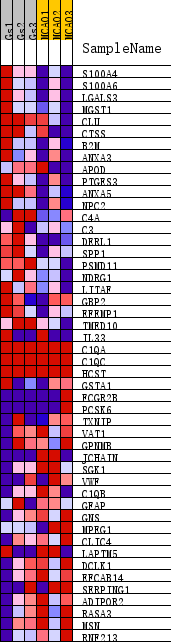

Supplement: Supplementary file 6 [file DataSheet_6.zip › fig 5 raw/fig 5-G raw/inflammation.Gsea.1649955013530/DEMAGALHAES_AGING_UP_129.png]

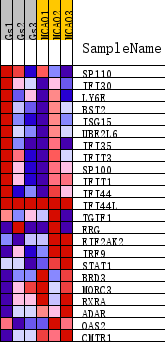

Supplement: Supplementary file 6 [file DataSheet_6.zip › fig 5 raw/fig 5-G raw/inflammation.Gsea.1649955013530/EINAV_INTERFERON_SIGNATURE_IN_CANCER_165.png]

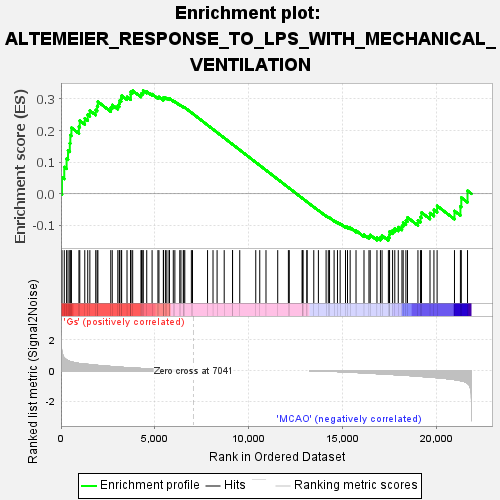

Supplement: Supplementary file 6 [file DataSheet_6.zip › fig 5 raw/fig 5-G raw/inflammation.Gsea.1649955013530/enplot_ALTEMEIER_RESPONSE_TO_LPS_WITH_MECHANICAL_VENTILATION_125.png]

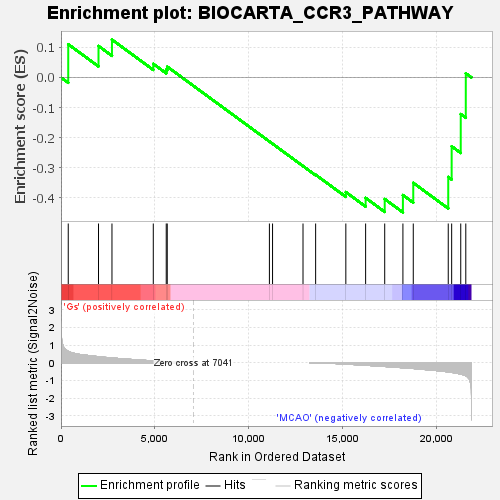

Supplement: Supplementary file 6 [file DataSheet_6.zip › fig 5 raw/fig 5-G raw/inflammation.Gsea.1649955013530/enplot_BIOCARTA_CCR3_PATHWAY_149.png]

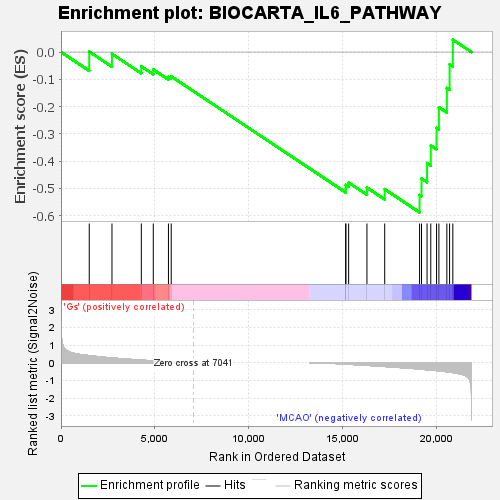

Supplement: Supplementary file 6 [file DataSheet_6.zip › fig 5 raw/fig 5-G raw/inflammation.Gsea.1649955013530/enplot_BIOCARTA_IL6_PATHWAY_146.png]

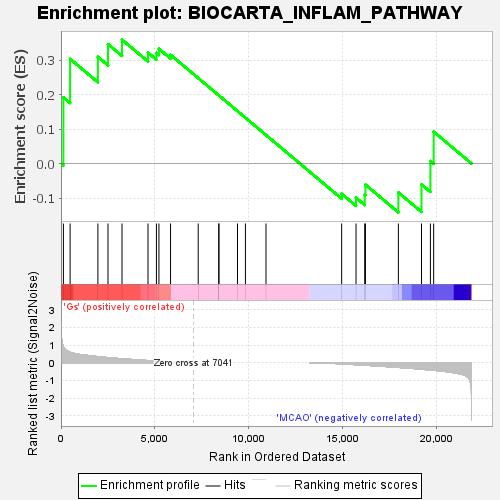

Supplement: Supplementary file 6 [file DataSheet_6.zip › fig 5 raw/fig 5-G raw/inflammation.Gsea.1649955013530/enplot_BIOCARTA_INFLAM_PATHWAY_134.png]

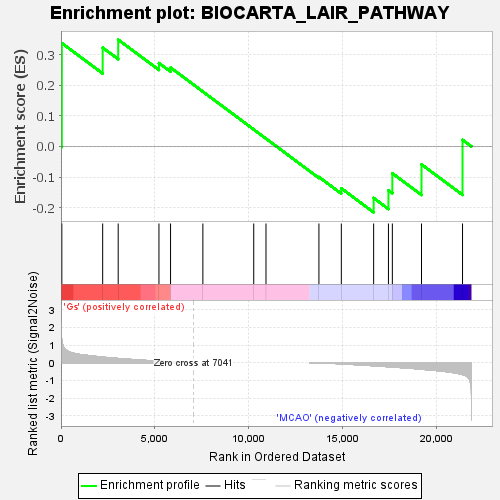

Supplement: Supplementary file 6 [file DataSheet_6.zip › fig 5 raw/fig 5-G raw/inflammation.Gsea.1649955013530/enplot_BIOCARTA_LAIR_PATHWAY_137.png]

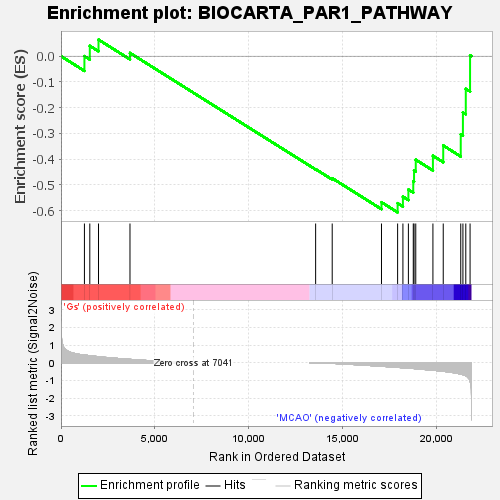

Supplement: Supplementary file 6 [file DataSheet_6.zip › fig 5 raw/fig 5-G raw/inflammation.Gsea.1649955013530/enplot_BIOCARTA_PAR1_PATHWAY_143.png]

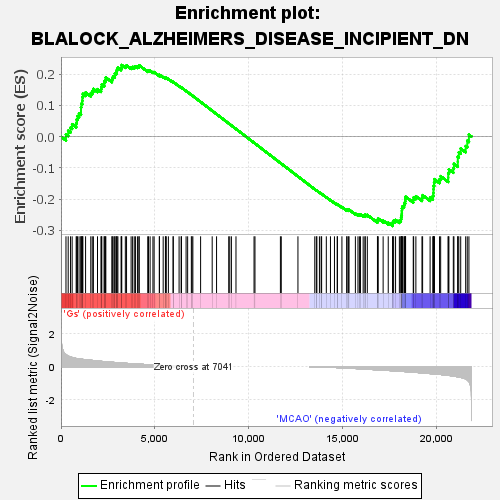

Supplement: Supplementary file 6 [file DataSheet_6.zip › fig 5 raw/fig 5-G raw/inflammation.Gsea.1649955013530/enplot_BLALOCK_ALZHEIMERS_DISEASE_INCIPIENT_DN_152.png]

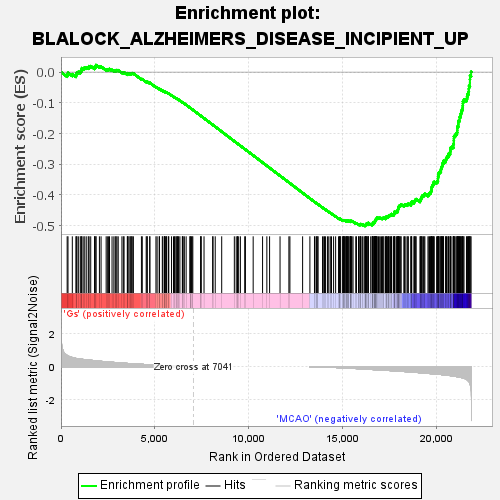

Supplement: Supplementary file 6 [file DataSheet_6.zip › fig 5 raw/fig 5-G raw/inflammation.Gsea.1649955013530/enplot_BLALOCK_ALZHEIMERS_DISEASE_INCIPIENT_UP_140.png]

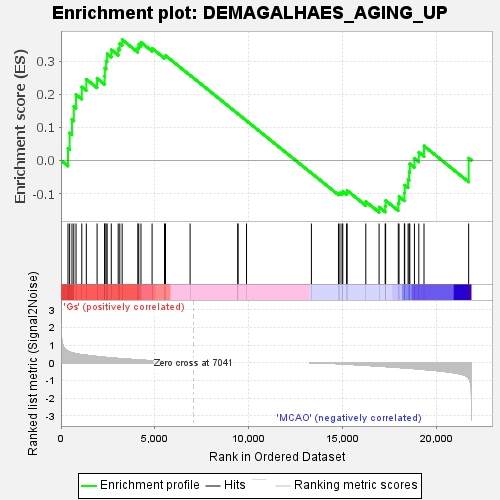

Supplement: Supplementary file 6 [file DataSheet_6.zip › fig 5 raw/fig 5-G raw/inflammation.Gsea.1649955013530/enplot_DEMAGALHAES_AGING_UP_128.png]

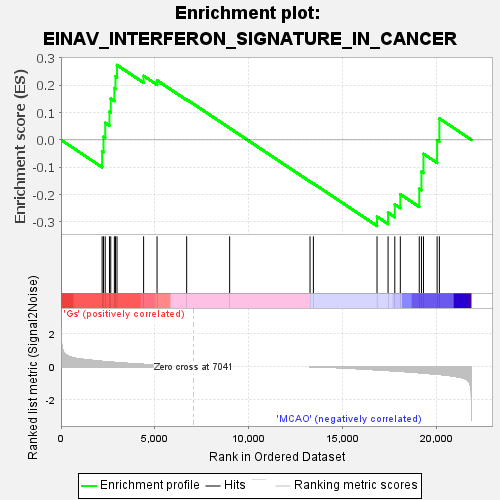

Supplement: Supplementary file 6 [file DataSheet_6.zip › fig 5 raw/fig 5-G raw/inflammation.Gsea.1649955013530/enplot_EINAV_INTERFERON_SIGNATURE_IN_CANCER_164.png]

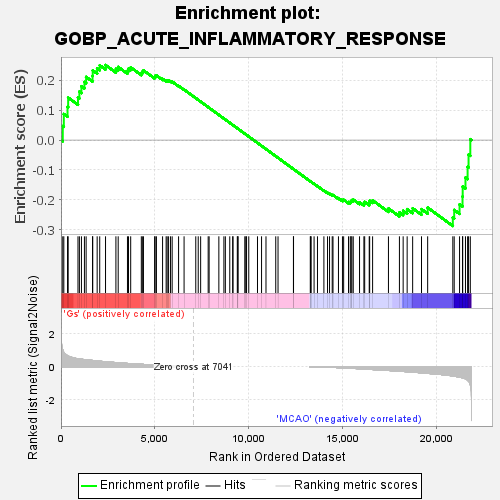

Supplement: Supplementary file 6 [file DataSheet_6.zip › fig 5 raw/fig 5-G raw/inflammation.Gsea.1649955013530/enplot_GOBP_ACUTE_INFLAMMATORY_RESPONSE_155.png]

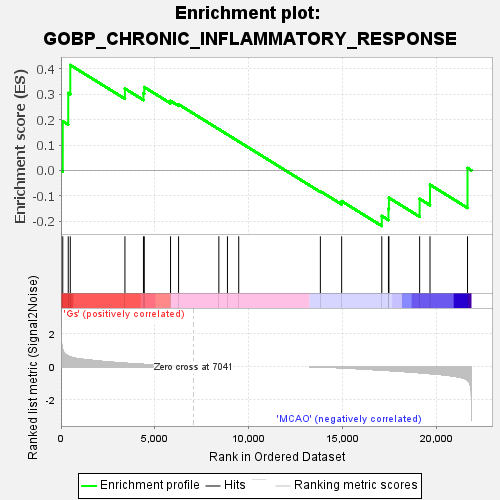

Supplement: Supplementary file 6 [file DataSheet_6.zip › fig 5 raw/fig 5-G raw/inflammation.Gsea.1649955013530/enplot_GOBP_CHRONIC_INFLAMMATORY_RESPONSE_131.png]

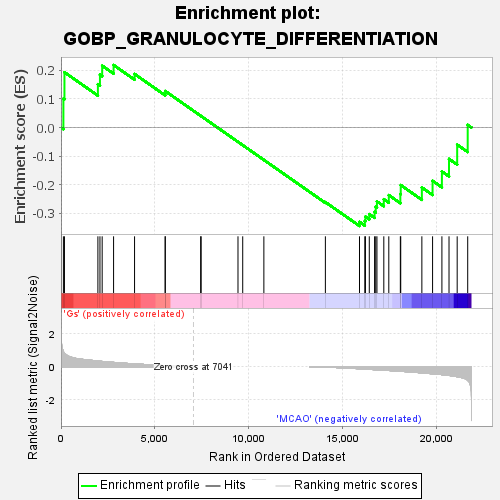

Supplement: Supplementary file 6 [file DataSheet_6.zip › fig 5 raw/fig 5-G raw/inflammation.Gsea.1649955013530/enplot_GOBP_GRANULOCYTE_DIFFERENTIATION_158.png]

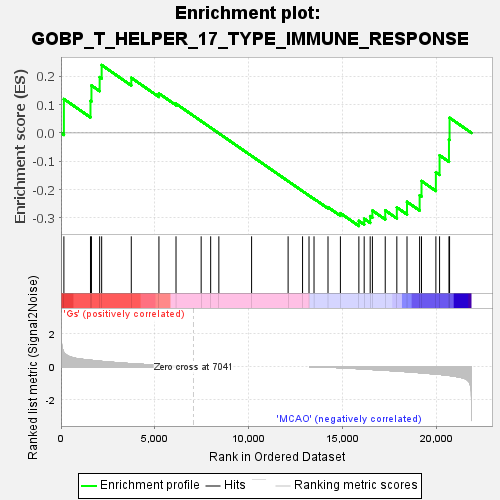

Supplement: Supplementary file 6 [file DataSheet_6.zip › fig 5 raw/fig 5-G raw/inflammation.Gsea.1649955013530/enplot_GOBP_T_HELPER_17_TYPE_IMMUNE_RESPONSE_161.png]

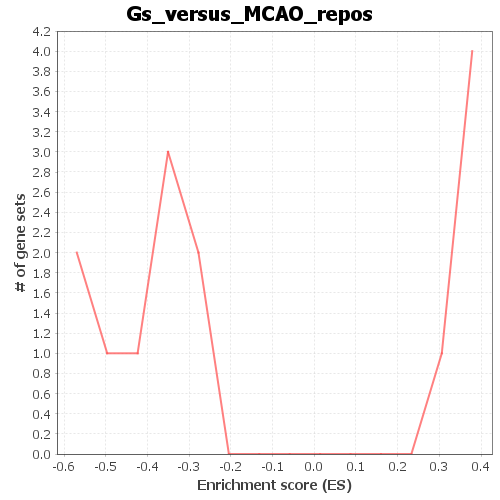

Supplement: Supplementary file 6 [file DataSheet_6.zip › fig 5 raw/fig 5-G raw/inflammation.Gsea.1649955013530/global_es_histogram.png]

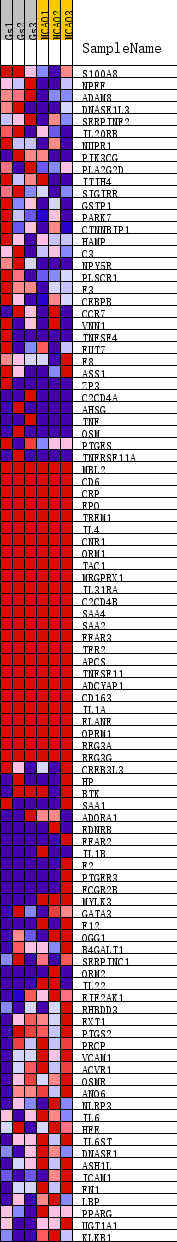

Supplement: Supplementary file 6 [file DataSheet_6.zip › fig 5 raw/fig 5-G raw/inflammation.Gsea.1649955013530/GOBP_ACUTE_INFLAMMATORY_RESPONSE_156.png]

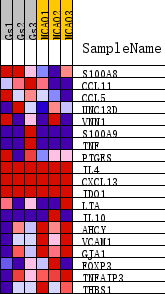

Supplement: Supplementary file 6 [file DataSheet_6.zip › fig 5 raw/fig 5-G raw/inflammation.Gsea.1649955013530/GOBP_CHRONIC_INFLAMMATORY_RESPONSE_132.png]

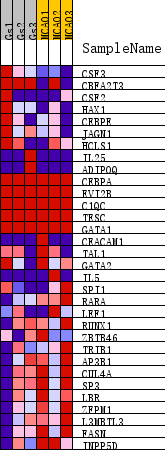

Supplement: Supplementary file 6 [file DataSheet_6.zip › fig 5 raw/fig 5-G raw/inflammation.Gsea.1649955013530/GOBP_GRANULOCYTE_DIFFERENTIATION_159.png]

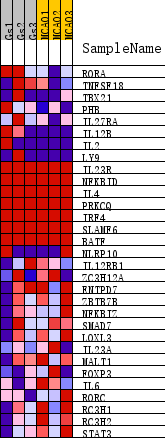

Supplement: Supplementary file 6 [file DataSheet_6.zip › fig 5 raw/fig 5-G raw/inflammation.Gsea.1649955013530/GOBP_T_HELPER_17_TYPE_IMMUNE_RESPONSE_162.png]

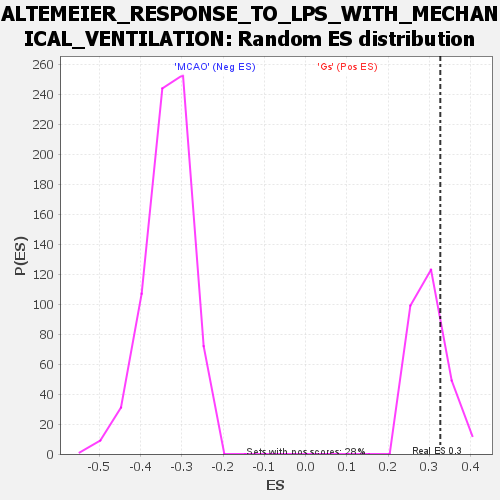

Supplement: Supplementary file 6 [file DataSheet_6.zip › fig 5 raw/fig 5-G raw/inflammation.Gsea.1649955013530/gset_rnd_es_dist_127.png]

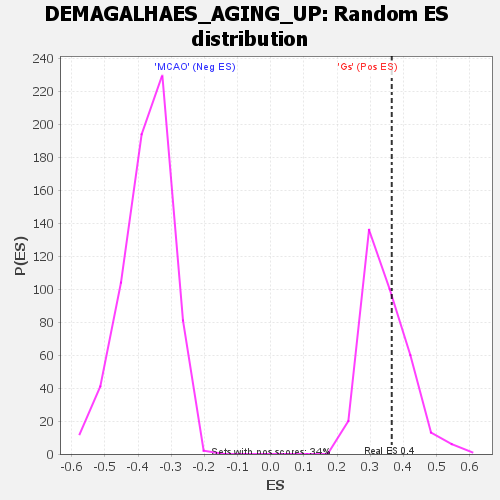

Supplement: Supplementary file 6 [file DataSheet_6.zip › fig 5 raw/fig 5-G raw/inflammation.Gsea.1649955013530/gset_rnd_es_dist_130.png]

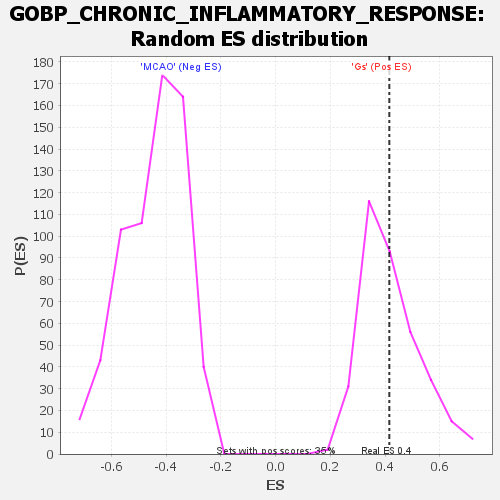

Supplement: Supplementary file 6 [file DataSheet_6.zip › fig 5 raw/fig 5-G raw/inflammation.Gsea.1649955013530/gset_rnd_es_dist_133.png]

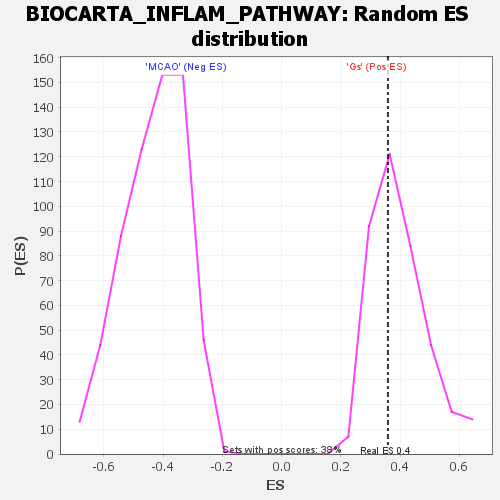

Supplement: Supplementary file 6 [file DataSheet_6.zip › fig 5 raw/fig 5-G raw/inflammation.Gsea.1649955013530/gset_rnd_es_dist_136.png]

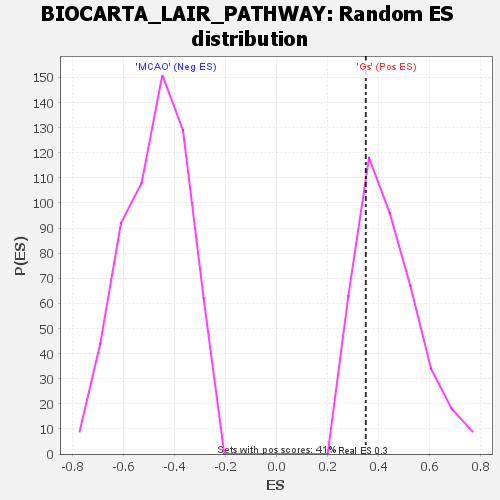

Supplement: Supplementary file 6 [file DataSheet_6.zip › fig 5 raw/fig 5-G raw/inflammation.Gsea.1649955013530/gset_rnd_es_dist_139.png]

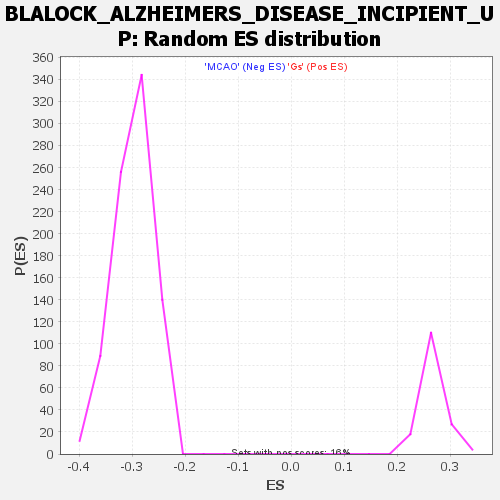

Supplement: Supplementary file 6 [file DataSheet_6.zip › fig 5 raw/fig 5-G raw/inflammation.Gsea.1649955013530/gset_rnd_es_dist_142.png]

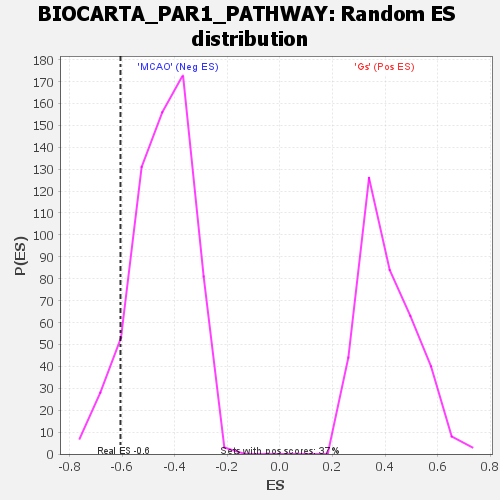

Supplement: Supplementary file 6 [file DataSheet_6.zip › fig 5 raw/fig 5-G raw/inflammation.Gsea.1649955013530/gset_rnd_es_dist_145.png]

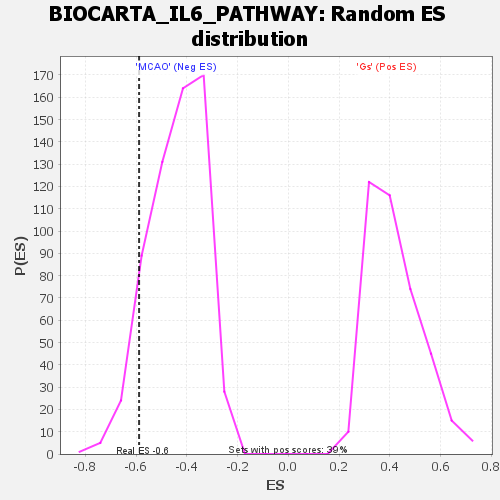

Supplement: Supplementary file 6 [file DataSheet_6.zip › fig 5 raw/fig 5-G raw/inflammation.Gsea.1649955013530/gset_rnd_es_dist_148.png]

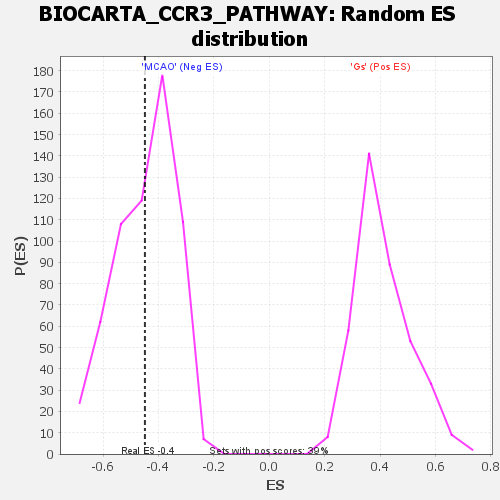

Supplement: Supplementary file 6 [file DataSheet_6.zip › fig 5 raw/fig 5-G raw/inflammation.Gsea.1649955013530/gset_rnd_es_dist_151.png]

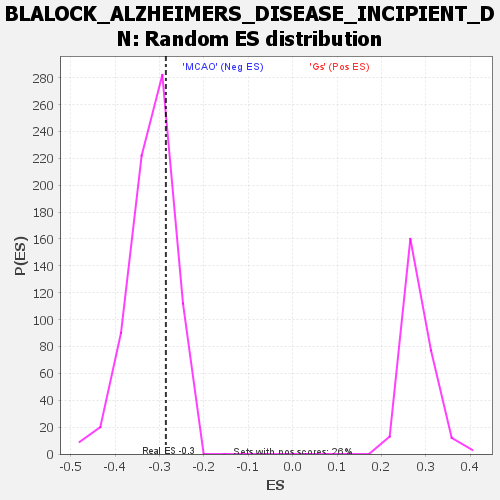

Supplement: Supplementary file 6 [file DataSheet_6.zip › fig 5 raw/fig 5-G raw/inflammation.Gsea.1649955013530/gset_rnd_es_dist_154.png]

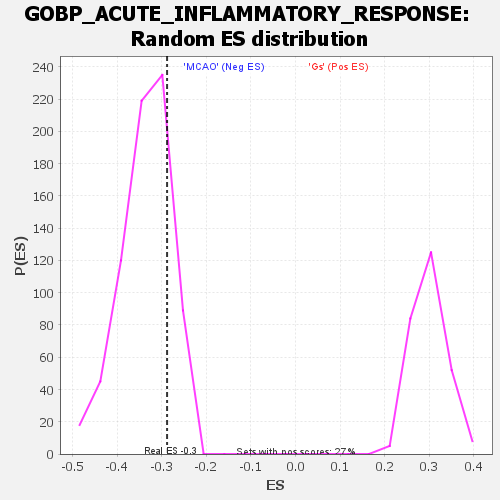

Supplement: Supplementary file 6 [file DataSheet_6.zip › fig 5 raw/fig 5-G raw/inflammation.Gsea.1649955013530/gset_rnd_es_dist_157.png]

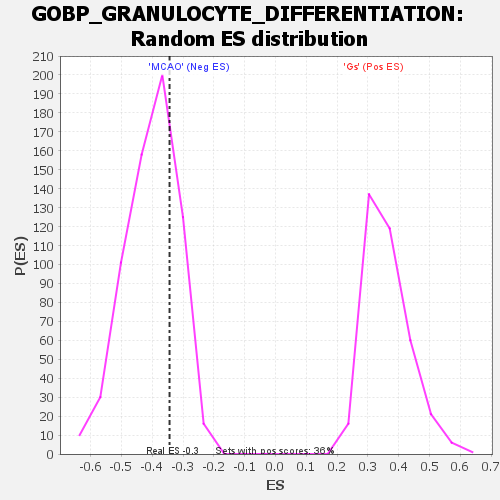

Supplement: Supplementary file 6 [file DataSheet_6.zip › fig 5 raw/fig 5-G raw/inflammation.Gsea.1649955013530/gset_rnd_es_dist_160.png]

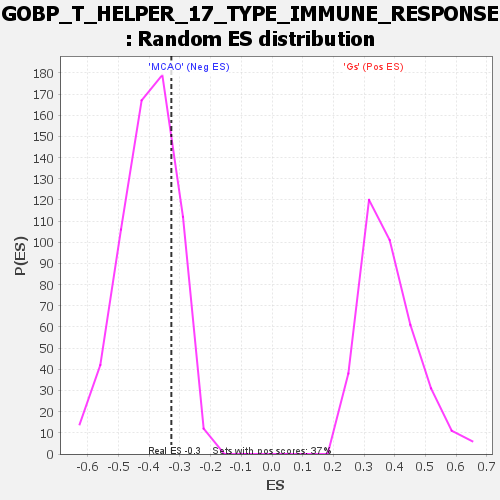

Supplement: Supplementary file 6 [file DataSheet_6.zip › fig 5 raw/fig 5-G raw/inflammation.Gsea.1649955013530/gset_rnd_es_dist_163.png]

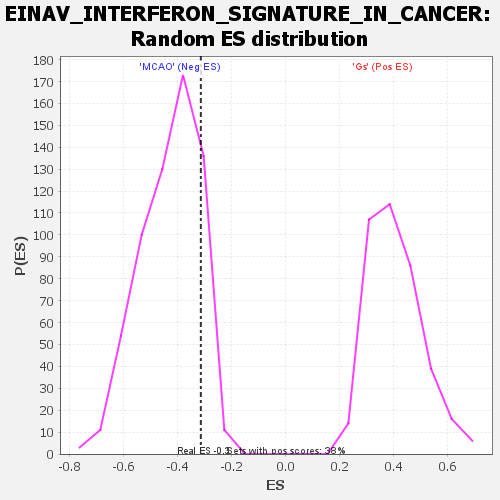

Supplement: Supplementary file 6 [file DataSheet_6.zip › fig 5 raw/fig 5-G raw/inflammation.Gsea.1649955013530/gset_rnd_es_dist_166.png]

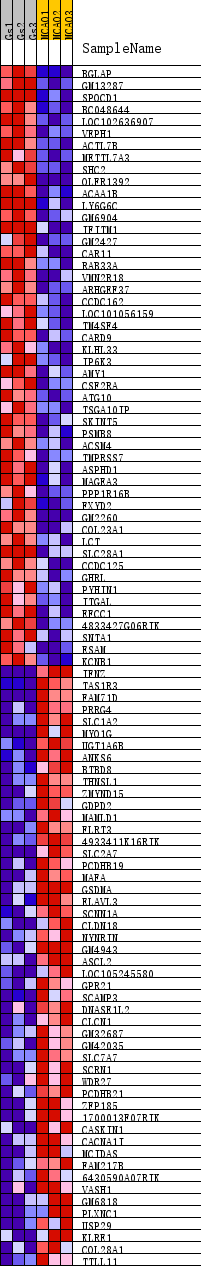

Supplement: Supplementary file 6 [file DataSheet_6.zip › fig 5 raw/fig 5-G raw/inflammation.Gsea.1649955013530/heat_map_123.png]

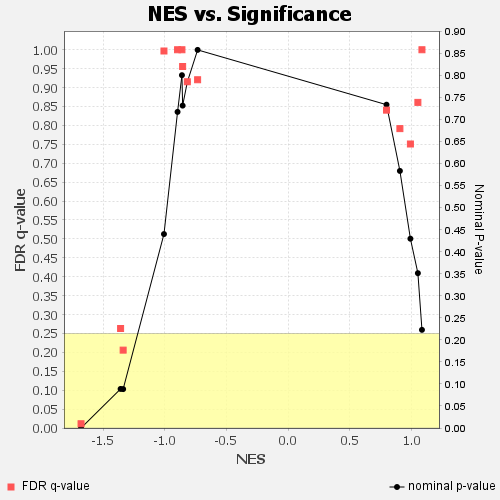

Supplement: Supplementary file 6 [file DataSheet_6.zip › fig 5 raw/fig 5-G raw/inflammation.Gsea.1649955013530/pvalues_vs_nes_plot.png]

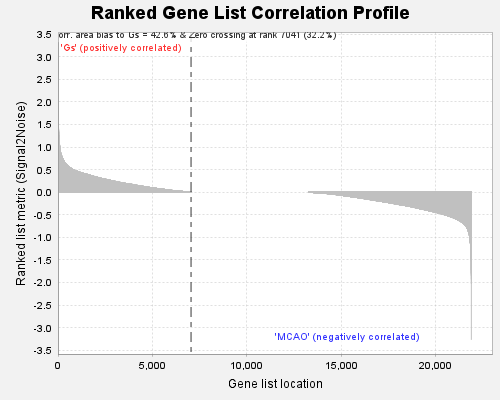

Supplement: Supplementary file 6 [file DataSheet_6.zip › fig 5 raw/fig 5-G raw/inflammation.Gsea.1649955013530/ranked_list_corr_124.png]

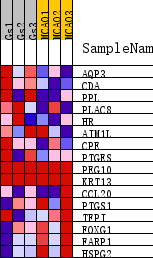

Supplement: Supplementary file 6 [file DataSheet_6.zip › fig 5 raw/fig 5-G raw/inflammation.Gsea.1649955060129/AZARE_NEOPLASTIC_TRANSFORMATION_BY_STAT3_DN_194.png]

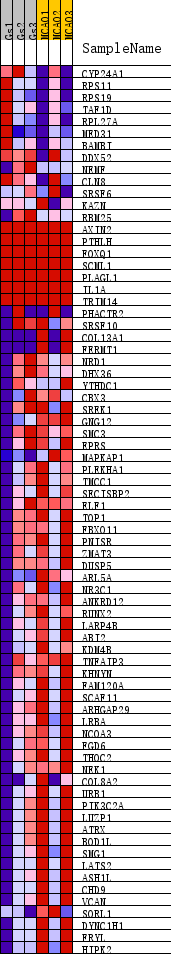

Supplement: Supplementary file 6 [file DataSheet_6.zip › fig 5 raw/fig 5-G raw/inflammation.Gsea.1649955060129/BILD_CTNNB1_ONCOGENIC_SIGNATURE_233.png]

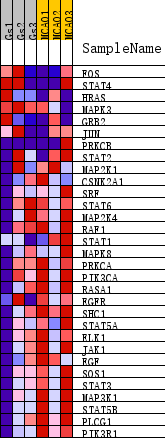

Supplement: Supplementary file 6 [file DataSheet_6.zip › fig 5 raw/fig 5-G raw/inflammation.Gsea.1649955060129/BIOCARTA_EGF_PATHWAY_284.png]

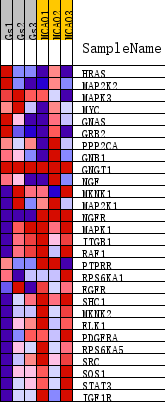

Supplement: Supplementary file 6 [file DataSheet_6.zip › fig 5 raw/fig 5-G raw/inflammation.Gsea.1649955060129/BIOCARTA_ERK_PATHWAY_278.png]

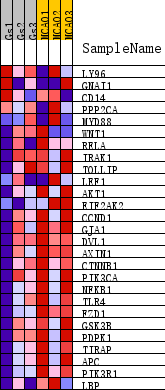

Supplement: Supplementary file 6 [file DataSheet_6.zip › fig 5 raw/fig 5-G raw/inflammation.Gsea.1649955060129/BIOCARTA_GSK3_PATHWAY_272.png]
