# Supplementary material for: Regulation of microglia related neuroinflammation contributes to the protective effect of Gelsevirine on ischemic stroke
Source: Front Immunol. 2023 Mar 30;14:1164278. doi: 10.3389/fimmu.2023.1164278 (PMC10098192; doi:10.3389/fimmu.2023.1164278)
Supplement: Supplementary file 6 [file DataSheet_6.zip › fig 5 raw/fig 5-G raw/inflammation.Gsea.1649955013530/ALTEMEIER_RESPONSE_TO_LPS_WITH_MECHANICAL_VENTILATION.html]

Details for gene set ALTEMEIER\_RESPONSE\_TO\_LPS\_WITH\_MECHANICAL\_VENTILATION[GSEA]

|  || Dataset | OGD\_DRUG\_DRUG.OGD\_FRUG.cls#Gs\_versus\_MCAO.OGD\_FRUG.cls#Gs\_versus\_MCAO\_repos |
| Phenotype | OGD\_FRUG.cls#Gs\_versus\_MCAO\_repos |
| Upregulated in class | Gs |
| GeneSet | ALTEMEIER\_RESPONSE\_TO\_LPS\_WITH\_MECHANICAL\_VENTILATION |
| Enrichment Score (ES) | 0.32658342 |
| Normalized Enrichment Score (NES) | 1.083963 |
| Nominal p-value | 0.22261484 |
| FDR q-value | 1.0 |
| FWER p-Value | 0.758 |
Table: GSEA Results Summary

  

Fig 1: Enrichment plot: ALTEMEIER\_RESPONSE\_TO\_LPS\_WITH\_MECHANICAL\_VENTILATION      
 Profile of the Running ES Score & Positions of GeneSet Members on the Rank Ordered List

  

| SYMBOL | TITLE | RANK IN GENE LIST | RANK METRIC SCORE | RUNNING ES | CORE ENRICHMENT || 1 | HPX | na | 52 | 1.198 | 0.0523 | Yes |
| 2 | RAB20 | na | 174 | 0.828 | 0.0846 | Yes |
| 3 | LST1 | na | 298 | 0.700 | 0.1110 | Yes |
| 4 | GCH1 | na | 375 | 0.643 | 0.1368 | Yes |
| 5 | PLA2G7 | na | 470 | 0.600 | 0.1599 | Yes |
| 6 | CXCL10 | na | 497 | 0.590 | 0.1857 | Yes |
| 7 | CCL2 | na | 556 | 0.569 | 0.2090 | Yes |
| 8 | ITGAM | na | 956 | 0.470 | 0.2122 | Yes |
| 9 | ATF3 | na | 1001 | 0.464 | 0.2314 | Yes |
| 10 | TRIM5 | na | 1267 | 0.426 | 0.2387 | Yes |
| 11 | TIMP1 | na | 1424 | 0.409 | 0.2502 | Yes |
| 12 | MAP3K8 | na | 1538 | 0.395 | 0.2630 | Yes |
| 13 | GADD45G | na | 1851 | 0.356 | 0.2650 | Yes |
| 14 | SAP30 | na | 1925 | 0.346 | 0.2774 | Yes |
| 15 | CSF2 | na | 1966 | 0.342 | 0.2912 | Yes |
| 16 | ISG15 | na | 2652 | 0.274 | 0.2723 | Yes |
| 17 | PLEK | na | 2731 | 0.267 | 0.2809 | Yes |
| 18 | ARG2 | na | 3028 | 0.239 | 0.2782 | Yes |
| 19 | MAFF | na | 3104 | 0.233 | 0.2854 | Yes |
| 20 | GEM | na | 3126 | 0.231 | 0.2950 | Yes |
| 21 | UPP1 | na | 3208 | 0.222 | 0.3014 | Yes |
| 22 | MARCKSL1 | na | 3232 | 0.220 | 0.3104 | Yes |
| 23 | GPR84 | na | 3518 | 0.198 | 0.3063 | Yes |
| 24 | USP18 | na | 3713 | 0.185 | 0.3059 | Yes |
| 25 | F3 | na | 3714 | 0.185 | 0.3144 | Yes |
| 26 | JUNB | na | 3720 | 0.185 | 0.3226 | Yes |
| 27 | NFKBIA | na | 3820 | 0.179 | 0.3262 | Yes |
| 28 | LITAF | na | 4262 | 0.146 | 0.3126 | Yes |
| 29 | NFIL3 | na | 4292 | 0.143 | 0.3178 | Yes |
| 30 | PLAUR | na | 4365 | 0.139 | 0.3208 | Yes |
| 31 | CEBPD | na | 4378 | 0.138 | 0.3266 | Yes |
| 32 | CD14 | na | 4569 | 0.124 | 0.3235 | No |
| 33 | GBP2 | na | 4854 | 0.105 | 0.3153 | No |
| 34 | RCAN1 | na | 5166 | 0.086 | 0.3049 | No |
| 35 | SELP | na | 5220 | 0.084 | 0.3063 | No |
| 36 | GBP4 | na | 5456 | 0.071 | 0.2987 | No |
| 37 | EBI3 | na | 5460 | 0.071 | 0.3018 | No |
| 38 | BST1 | na | 5464 | 0.070 | 0.3049 | No |
| 39 | IL1R2 | na | 5565 | 0.065 | 0.3033 | No |
| 40 | TREX1 | na | 5641 | 0.061 | 0.3026 | No |
| 41 | SOD2 | na | 5759 | 0.054 | 0.2997 | No |
| 42 | RELB | na | 5778 | 0.053 | 0.3013 | No |
| 43 | IL4I1 | na | 5984 | 0.042 | 0.2938 | No |
| 44 | SLC15A3 | na | 6068 | 0.038 | 0.2917 | No |
| 45 | TIFA | na | 6331 | 0.027 | 0.2810 | No |
| 46 | TNFSF9 | na | 6392 | 0.025 | 0.2793 | No |
| 47 | TFEC | na | 6516 | 0.020 | 0.2746 | No |
| 48 | CSF2RB | na | 6547 | 0.019 | 0.2741 | No |
| 49 | BCL3 | na | 6597 | 0.017 | 0.2726 | No |
| 50 | FPR2 | na | 6947 | 0.004 | 0.2567 | No |
| 51 | AKAP12 | na | 7008 | 0.001 | 0.2540 | No |
| 52 | MEFV | na | 7810 | 0.000 | 0.2172 | No |
| 53 | GDA | na | 8101 | 0.000 | 0.2039 | No |
| 54 | GPR65 | na | 8318 | 0.000 | 0.1940 | No |
| 55 | IFIH1 | na | 8697 | 0.000 | 0.1766 | No |
| 56 | CXCL3 | na | 9141 | 0.000 | 0.1562 | No |
| 57 | CXCL2 | na | 9142 | 0.000 | 0.1562 | No |
| 58 | TFPI2 | na | 9526 | 0.000 | 0.1386 | No |
| 59 | SELL | na | 10379 | 0.000 | 0.0995 | No |
| 60 | CYBB | na | 10592 | 0.000 | 0.0898 | No |
| 61 | IL1A | na | 10924 | 0.000 | 0.0746 | No |
| 62 | CMPK2 | na | 11549 | 0.000 | 0.0459 | No |
| 63 | IRGM | na | 12118 | 0.000 | 0.0198 | No |
| 64 | CSF3R | na | 12165 | 0.000 | 0.0177 | No |
| 65 | CCL3 | na | 12847 | 0.000 | -0.0136 | No |
| 66 | CCR1 | na | 12877 | 0.000 | -0.0149 | No |
| 67 | CCR2 | na | 12899 | 0.000 | -0.0159 | No |
| 68 | CLEC4D | na | 13108 | 0.000 | -0.0254 | No |
| 69 | CLEC4E | na | 13109 | 0.000 | -0.0254 | No |
| 70 | IL1RN | na | 13471 | -0.007 | -0.0417 | No |
| 71 | SLC26A4 | na | 13717 | -0.013 | -0.0524 | No |
| 72 | FGL2 | na | 14127 | -0.024 | -0.0701 | No |
| 73 | IRF7 | na | 14245 | -0.029 | -0.0741 | No |
| 74 | IL1B | na | 14311 | -0.032 | -0.0756 | No |
| 75 | F13A1 | na | 14554 | -0.043 | -0.0848 | No |
| 76 | STAT2 | na | 14734 | -0.053 | -0.0906 | No |
| 77 | CH25H | na | 14876 | -0.060 | -0.0943 | No |
| 78 | CCRL2 | na | 15162 | -0.078 | -0.1038 | No |
| 79 | TNFAIP2 | na | 15268 | -0.084 | -0.1048 | No |
| 80 | CASP4 | na | 15409 | -0.093 | -0.1070 | No |
| 81 | IL15 | na | 15722 | -0.110 | -0.1163 | No |
| 82 | TGM1 | na | 16147 | -0.138 | -0.1295 | No |
| 83 | CCL20 | na | 16412 | -0.155 | -0.1345 | No |
| 84 | NFKBIZ | na | 16477 | -0.160 | -0.1302 | No |
| 85 | SOCS3 | na | 16838 | -0.185 | -0.1383 | No |
| 86 | LCP2 | na | 17023 | -0.198 | -0.1377 | No |
| 87 | OAS3 | na | 17110 | -0.203 | -0.1323 | No |
| 88 | SERPINE1 | na | 17446 | -0.226 | -0.1374 | No |
| 89 | SLA | na | 17503 | -0.230 | -0.1295 | No |
| 90 | ADM | na | 17510 | -0.231 | -0.1192 | No |
| 91 | MXD1 | na | 17671 | -0.242 | -0.1155 | No |
| 92 | STAT1 | na | 17788 | -0.252 | -0.1093 | No |
| 93 | GK | na | 17973 | -0.267 | -0.1055 | No |
| 94 | FCER1G | na | 18170 | -0.282 | -0.1016 | No |
| 95 | OSMR | na | 18234 | -0.287 | -0.0914 | No |
| 96 | IFIT2 | na | 18380 | -0.295 | -0.0845 | No |
| 97 | CCL17 | na | 18465 | -0.301 | -0.0746 | No |
| 98 | RDH5 | na | 19022 | -0.351 | -0.0841 | No |
| 99 | ST3GAL1 | na | 19156 | -0.365 | -0.0735 | No |
| 100 | IL6 | na | 19212 | -0.371 | -0.0591 | No |
| 101 | TNFAIP3 | na | 19665 | -0.413 | -0.0610 | No |
| 102 | PFKFB3 | na | 19874 | -0.433 | -0.0508 | No |
| 103 | OAS2 | na | 20043 | -0.449 | -0.0380 | No |
| 104 | TLR2 | na | 20969 | -0.572 | -0.0543 | No |
| 105 | ADAMTS4 | na | 21285 | -0.639 | -0.0396 | No |
| 106 | VCAN | na | 21332 | -0.652 | -0.0119 | No |
| 107 | THBS1 | na | 21664 | -0.806 | 0.0097 | No |
Table: GSEA details [plain text format]

  

Fig 2: ALTEMEIER\_RESPONSE\_TO\_LPS\_WITH\_MECHANICAL\_VENTILATION      
 Blue-Pink O' Gram in the Space of the Analyzed GeneSet

  

Fig 3: ALTEMEIER\_RESPONSE\_TO\_LPS\_WITH\_MECHANICAL\_VENTILATION: Random ES distribution      
 Gene set null distribution of ES for **ALTEMEIER\_RESPONSE\_TO\_LPS\_WITH\_MECHANICAL\_VENTILATION**

  
