# Supplementary material for: Regulation of microglia related neuroinflammation contributes to the protective effect of Gelsevirine on ischemic stroke
Source: Front Immunol. 2023 Mar 30;14:1164278. doi: 10.3389/fimmu.2023.1164278 (PMC10098192; doi:10.3389/fimmu.2023.1164278)
Supplement: Supplementary file 6 [file DataSheet_6.zip › fig 5 raw/fig 5-G raw/inflammation.Gsea.1649955013530/BIOCARTA_CCR3_PATHWAY.html]

Details for gene set BIOCARTA\_CCR3\_PATHWAY[GSEA]

|  || Dataset | OGD\_DRUG\_DRUG.OGD\_FRUG.cls#Gs\_versus\_MCAO.OGD\_FRUG.cls#Gs\_versus\_MCAO\_repos |
| Phenotype | OGD\_FRUG.cls#Gs\_versus\_MCAO\_repos |
| Upregulated in class | MCAO |
| GeneSet | BIOCARTA\_CCR3\_PATHWAY |
| Enrichment Score (ES) | -0.44892243 |
| Normalized Enrichment Score (NES) | -1.006164 |
| Nominal p-value | 0.4398682 |
| FDR q-value | 0.99659735 |
| FWER p-Value | 0.992 |
Table: GSEA Results Summary

  

Fig 1: Enrichment plot: BIOCARTA\_CCR3\_PATHWAY      
 Profile of the Running ES Score & Positions of GeneSet Members on the Rank Ordered List

  

| SYMBOL | TITLE | RANK IN GENE LIST | RANK METRIC SCORE | RUNNING ES | CORE ENRICHMENT || 1 | CCL11 | na | 386 | 0.637 | 0.1105 | No |
| 2 | RHOA | na | 1999 | 0.338 | 0.1047 | No |
| 3 | HRAS | na | 2716 | 0.268 | 0.1259 | No |
| 4 | MAPK3 | na | 4918 | 0.100 | 0.0453 | No |
| 5 | GNAS | na | 5608 | 0.063 | 0.0264 | No |
| 6 | NOX1 | na | 5662 | 0.060 | 0.0360 | No |
| 7 | PIK3C2G | na | 11106 | 0.000 | -0.2130 | No |
| 8 | MYL2 | na | 11274 | 0.000 | -0.2207 | No |
| 9 | CCR3 | na | 12897 | 0.000 | -0.2949 | No |
| 10 | PRKCB | na | 13570 | -0.009 | -0.3237 | No |
| 11 | MAP2K1 | na | 15179 | -0.079 | -0.3814 | No |
| 12 | MAPK1 | na | 16234 | -0.143 | -0.4008 | No |
| 13 | RAF1 | na | 17251 | -0.212 | -0.4046 | Yes |
| 14 | PRKCA | na | 18221 | -0.286 | -0.3914 | Yes |
| 15 | GNAQ | na | 18773 | -0.327 | -0.3507 | Yes |
| 16 | ROCK2 | na | 20637 | -0.521 | -0.3312 | Yes |
| 17 | LIMK1 | na | 20819 | -0.549 | -0.2291 | Yes |
| 18 | PPP1R12B | na | 21303 | -0.642 | -0.1219 | Yes |
| 19 | PLCB1 | na | 21572 | -0.736 | 0.0139 | Yes |
Table: GSEA details [plain text format]

  

Fig 2: BIOCARTA\_CCR3\_PATHWAY      
 Blue-Pink O' Gram in the Space of the Analyzed GeneSet

  

Fig 3: BIOCARTA\_CCR3\_PATHWAY: Random ES distribution      
 Gene set null distribution of ES for **BIOCARTA\_CCR3\_PATHWAY**

  
