# Supplementary material for: Regulation of microglia related neuroinflammation contributes to the protective effect of Gelsevirine on ischemic stroke
Source: Front Immunol. 2023 Mar 30;14:1164278. doi: 10.3389/fimmu.2023.1164278 (PMC10098192; doi:10.3389/fimmu.2023.1164278)
Supplement: Supplementary file 6 [file DataSheet_6.zip › fig 5 raw/fig 5-G raw/inflammation.Gsea.1649955013530/BIOCARTA_IL6_PATHWAY.html]

Details for gene set BIOCARTA\_IL6\_PATHWAY[GSEA]

|  || Dataset | OGD\_DRUG\_DRUG.OGD\_FRUG.cls#Gs\_versus\_MCAO.OGD\_FRUG.cls#Gs\_versus\_MCAO\_repos |
| Phenotype | OGD\_FRUG.cls#Gs\_versus\_MCAO\_repos |
| Upregulated in class | MCAO |
| GeneSet | BIOCARTA\_IL6\_PATHWAY |
| Enrichment Score (ES) | -0.58783954 |
| Normalized Enrichment Score (NES) | -1.3369197 |
| Nominal p-value | 0.0882353 |
| FDR q-value | 0.20598723 |
| FWER p-Value | 0.48 |
Table: GSEA Results Summary

  

Fig 1: Enrichment plot: BIOCARTA\_IL6\_PATHWAY      
 Profile of the Running ES Score & Positions of GeneSet Members on the Rank Ordered List

  

| SYMBOL | TITLE | RANK IN GENE LIST | RANK METRIC SCORE | RUNNING ES | CORE ENRICHMENT || 1 | FOS | na | 1505 | 0.399 | 0.0016 | No |
| 2 | HRAS | na | 2716 | 0.268 | -0.0065 | No |
| 3 | CEBPB | na | 4280 | 0.144 | -0.0527 | No |
| 4 | MAPK3 | na | 4918 | 0.100 | -0.0642 | No |
| 5 | GRB2 | na | 5726 | 0.057 | -0.0911 | No |
| 6 | JUN | na | 5874 | 0.048 | -0.0894 | No |
| 7 | JAK2 | na | 15175 | -0.079 | -0.5010 | No |
| 8 | MAP2K1 | na | 15179 | -0.079 | -0.4872 | No |
| 9 | CSNK2A1 | na | 15329 | -0.088 | -0.4785 | No |
| 10 | SRF | na | 16304 | -0.148 | -0.4970 | No |
| 11 | RAF1 | na | 17251 | -0.212 | -0.5029 | No |
| 12 | SHC1 | na | 19109 | -0.361 | -0.5243 | Yes |
| 13 | IL6 | na | 19212 | -0.371 | -0.4636 | Yes |
| 14 | PTPN11 | na | 19508 | -0.397 | -0.4071 | Yes |
| 15 | ELK1 | na | 19706 | -0.417 | -0.3425 | Yes |
| 16 | JAK1 | na | 20014 | -0.446 | -0.2779 | Yes |
| 17 | JAK3 | na | 20139 | -0.460 | -0.2025 | Yes |
| 18 | SOS1 | na | 20563 | -0.511 | -0.1318 | Yes |
| 19 | STAT3 | na | 20708 | -0.529 | -0.0450 | Yes |
| 20 | IL6ST | na | 20881 | -0.558 | 0.0455 | Yes |
Table: GSEA details [plain text format]

  

Fig 2: BIOCARTA\_IL6\_PATHWAY      
 Blue-Pink O' Gram in the Space of the Analyzed GeneSet

  

Fig 3: BIOCARTA\_IL6\_PATHWAY: Random ES distribution      
 Gene set null distribution of ES for **BIOCARTA\_IL6\_PATHWAY**

  
