# Supplementary material for: Regulation of microglia related neuroinflammation contributes to the protective effect of Gelsevirine on ischemic stroke
Source: Front Immunol. 2023 Mar 30;14:1164278. doi: 10.3389/fimmu.2023.1164278 (PMC10098192; doi:10.3389/fimmu.2023.1164278)
Supplement: Supplementary file 6 [file DataSheet_6.zip › fig 5 raw/fig 5-G raw/inflammation.Gsea.1649955013530/BIOCARTA_INFLAM_PATHWAY.html]

Details for gene set BIOCARTA\_INFLAM\_PATHWAY[GSEA]

|  || Dataset | OGD\_DRUG\_DRUG.OGD\_FRUG.cls#Gs\_versus\_MCAO.OGD\_FRUG.cls#Gs\_versus\_MCAO\_repos |
| Phenotype | OGD\_FRUG.cls#Gs\_versus\_MCAO\_repos |
| Upregulated in class | Gs |
| GeneSet | BIOCARTA\_INFLAM\_PATHWAY |
| Enrichment Score (ES) | 0.35907573 |
| Normalized Enrichment Score (NES) | 0.9053043 |
| Nominal p-value | 0.58311343 |
| FDR q-value | 0.7912752 |
| FWER p-Value | 0.961 |
Table: GSEA Results Summary

  

Fig 1: Enrichment plot: BIOCARTA\_INFLAM\_PATHWAY      
 Profile of the Running ES Score & Positions of GeneSet Members on the Rank Ordered List

  

| SYMBOL | TITLE | RANK IN GENE LIST | RANK METRIC SCORE | RUNNING ES | CORE ENRICHMENT || 1 | CSF3 | na | 128 | 0.917 | 0.1917 | Yes |
| 2 | TGFB2 | na | 485 | 0.594 | 0.3032 | Yes |
| 3 | CSF2 | na | 1966 | 0.342 | 0.3092 | Yes |
| 4 | PDGFA | na | 2504 | 0.286 | 0.3461 | Yes |
| 5 | IL11 | na | 3251 | 0.219 | 0.3591 | Yes |
| 6 | IL13 | na | 4636 | 0.119 | 0.3215 | No |
| 7 | IFNB1 | na | 5085 | 0.091 | 0.3206 | No |
| 8 | IL2 | na | 5219 | 0.084 | 0.3325 | No |
| 9 | TNF | na | 5838 | 0.050 | 0.3150 | No |
| 10 | CD4 | na | 7312 | 0.000 | 0.2476 | No |
| 11 | IL4 | na | 8412 | 0.000 | 0.1973 | No |
| 12 | IL3 | na | 8413 | 0.000 | 0.1973 | No |
| 13 | IFNA1 | na | 9405 | 0.000 | 0.1520 | No |
| 14 | IFNG | na | 9828 | 0.000 | 0.1327 | No |
| 15 | IL1A | na | 10924 | 0.000 | 0.0826 | No |
| 16 | IL10 | na | 14957 | -0.065 | -0.0879 | No |
| 17 | IL15 | na | 15722 | -0.110 | -0.0992 | No |
| 18 | TGFB1 | na | 16186 | -0.140 | -0.0902 | No |
| 19 | IL5 | na | 16224 | -0.142 | -0.0614 | No |
| 20 | CSF1 | na | 17979 | -0.268 | -0.0840 | No |
| 21 | IL6 | na | 19212 | -0.371 | -0.0606 | No |
| 22 | IL7 | na | 19686 | -0.415 | 0.0072 | No |
| 23 | TGFB3 | na | 19860 | -0.432 | 0.0922 | No |
Table: GSEA details [plain text format]

  

Fig 2: BIOCARTA\_INFLAM\_PATHWAY      
 Blue-Pink O' Gram in the Space of the Analyzed GeneSet

  

Fig 3: BIOCARTA\_INFLAM\_PATHWAY: Random ES distribution      
 Gene set null distribution of ES for **BIOCARTA\_INFLAM\_PATHWAY**

  
