# Supplementary material for: Regulation of microglia related neuroinflammation contributes to the protective effect of Gelsevirine on ischemic stroke
Source: Front Immunol. 2023 Mar 30;14:1164278. doi: 10.3389/fimmu.2023.1164278 (PMC10098192; doi:10.3389/fimmu.2023.1164278)
Supplement: Supplementary file 6 [file DataSheet_6.zip › fig 5 raw/fig 5-G raw/inflammation.Gsea.1649955013530/BIOCARTA_LAIR_PATHWAY.html]

Details for gene set BIOCARTA\_LAIR\_PATHWAY[GSEA]

|  || Dataset | OGD\_DRUG\_DRUG.OGD\_FRUG.cls#Gs\_versus\_MCAO.OGD\_FRUG.cls#Gs\_versus\_MCAO\_repos |
| Phenotype | OGD\_FRUG.cls#Gs\_versus\_MCAO\_repos |
| Upregulated in class | Gs |
| GeneSet | BIOCARTA\_LAIR\_PATHWAY |
| Enrichment Score (ES) | 0.34961596 |
| Normalized Enrichment Score (NES) | 0.7968329 |
| Nominal p-value | 0.73333335 |
| FDR q-value | 0.8401687 |
| FWER p-Value | 0.99 |
Table: GSEA Results Summary

  

Fig 1: Enrichment plot: BIOCARTA\_LAIR\_PATHWAY      
 Profile of the Running ES Score & Positions of GeneSet Members on the Rank Ordered List

  

| SYMBOL | TITLE | RANK IN GENE LIST | RANK METRIC SCORE | RUNNING ES | CORE ENRICHMENT || 1 | ITGAL | na | 44 | 1.259 | 0.3383 | Yes |
| 2 | SELPLG | na | 2221 | 0.313 | 0.3234 | Yes |
| 3 | C3 | na | 3048 | 0.237 | 0.3496 | Yes |
| 4 | SELP | na | 5220 | 0.084 | 0.2729 | No |
| 5 | TNF | na | 5838 | 0.050 | 0.2582 | No |
| 6 | C6 | na | 7560 | 0.000 | 0.1795 | No |
| 7 | KNG1 | na | 10271 | 0.000 | 0.0556 | No |
| 8 | IL1A | na | 10924 | 0.000 | 0.0257 | No |
| 9 | ITGA4 | na | 13745 | -0.013 | -0.0998 | No |
| 10 | C7 | na | 14939 | -0.065 | -0.1369 | No |
| 11 | ITGB1 | na | 16661 | -0.174 | -0.1686 | No |
| 12 | VCAM1 | na | 17450 | -0.226 | -0.1435 | No |
| 13 | ITGB2 | na | 17658 | -0.242 | -0.0876 | No |
| 14 | IL6 | na | 19212 | -0.371 | -0.0585 | No |
| 15 | ICAM1 | na | 21398 | -0.667 | 0.0218 | No |
Table: GSEA details [plain text format]

  

Fig 2: BIOCARTA\_LAIR\_PATHWAY      
 Blue-Pink O' Gram in the Space of the Analyzed GeneSet

  

Fig 3: BIOCARTA\_LAIR\_PATHWAY: Random ES distribution      
 Gene set null distribution of ES for **BIOCARTA\_LAIR\_PATHWAY**

  
