# Supplementary material for: Regulation of microglia related neuroinflammation contributes to the protective effect of Gelsevirine on ischemic stroke
Source: Front Immunol. 2023 Mar 30;14:1164278. doi: 10.3389/fimmu.2023.1164278 (PMC10098192; doi:10.3389/fimmu.2023.1164278)
Supplement: Supplementary file 6 [file DataSheet_6.zip › fig 5 raw/fig 5-G raw/inflammation.Gsea.1649955013530/BIOCARTA_PAR1_PATHWAY.html]

Details for gene set BIOCARTA\_PAR1\_PATHWAY[GSEA]

|  || Dataset | OGD\_DRUG\_DRUG.OGD\_FRUG.cls#Gs\_versus\_MCAO.OGD\_FRUG.cls#Gs\_versus\_MCAO\_repos |
| Phenotype | OGD\_FRUG.cls#Gs\_versus\_MCAO\_repos |
| Upregulated in class | MCAO |
| GeneSet | BIOCARTA\_PAR1\_PATHWAY |
| Enrichment Score (ES) | -0.60643816 |
| Normalized Enrichment Score (NES) | -1.357524 |
| Nominal p-value | 0.088607594 |
| FDR q-value | 0.26300213 |
| FWER p-Value | 0.431 |
Table: GSEA Results Summary

  

Fig 1: Enrichment plot: BIOCARTA\_PAR1\_PATHWAY      
 Profile of the Running ES Score & Positions of GeneSet Members on the Rank Ordered List

  

| SYMBOL | TITLE | RANK IN GENE LIST | RANK METRIC SCORE | RUNNING ES | CORE ENRICHMENT || 1 | PIK3CG | na | 1248 | 0.428 | 0.0003 | No |
| 2 | F2RL3 | na | 1535 | 0.396 | 0.0402 | No |
| 3 | RHOA | na | 1999 | 0.338 | 0.0643 | No |
| 4 | ADCY1 | na | 3676 | 0.187 | 0.0126 | No |
| 5 | PRKCB | na | 13570 | -0.009 | -0.4387 | No |
| 6 | F2 | na | 14453 | -0.038 | -0.4740 | No |
| 7 | MAP3K7 | na | 17079 | -0.201 | -0.5671 | No |
| 8 | F2R | na | 17939 | -0.265 | -0.5709 | Yes |
| 9 | PRKCA | na | 18221 | -0.286 | -0.5455 | Yes |
| 10 | PIK3CA | na | 18513 | -0.305 | -0.5180 | Yes |
| 11 | GNAQ | na | 18773 | -0.327 | -0.4860 | Yes |
| 12 | ARHGEF1 | na | 18815 | -0.331 | -0.4435 | Yes |
| 13 | GNA13 | na | 18906 | -0.340 | -0.4021 | Yes |
| 14 | ROCK1 | na | 19820 | -0.427 | -0.3866 | Yes |
| 15 | GNA12 | na | 20370 | -0.485 | -0.3468 | Yes |
| 16 | PPP1R12B | na | 21303 | -0.642 | -0.3034 | Yes |
| 17 | PIK3R1 | na | 21419 | -0.674 | -0.2184 | Yes |
| 18 | PLCB1 | na | 21572 | -0.736 | -0.1268 | Yes |
| 19 | PTK2B | na | 21800 | -1.050 | 0.0034 | Yes |
Table: GSEA details [plain text format]

  

Fig 2: BIOCARTA\_PAR1\_PATHWAY      
 Blue-Pink O' Gram in the Space of the Analyzed GeneSet

  

Fig 3: BIOCARTA\_PAR1\_PATHWAY: Random ES distribution      
 Gene set null distribution of ES for **BIOCARTA\_PAR1\_PATHWAY**

  
