# Supplementary material for: Regulation of microglia related neuroinflammation contributes to the protective effect of Gelsevirine on ischemic stroke
Source: Front Immunol. 2023 Mar 30;14:1164278. doi: 10.3389/fimmu.2023.1164278 (PMC10098192; doi:10.3389/fimmu.2023.1164278)
Supplement: Supplementary file 6 [file DataSheet_6.zip › fig 5 raw/fig 5-G raw/inflammation.Gsea.1649955013530/BLALOCK_ALZHEIMERS_DISEASE_INCIPIENT_DN.html]

Details for gene set BLALOCK\_ALZHEIMERS\_DISEASE\_INCIPIENT\_DN[GSEA]

|  || Dataset | OGD\_DRUG\_DRUG.OGD\_FRUG.cls#Gs\_versus\_MCAO.OGD\_FRUG.cls#Gs\_versus\_MCAO\_repos |
| Phenotype | OGD\_FRUG.cls#Gs\_versus\_MCAO\_repos |
| Upregulated in class | MCAO |
| GeneSet | BLALOCK\_ALZHEIMERS\_DISEASE\_INCIPIENT\_DN |
| Enrichment Score (ES) | -0.2857663 |
| Normalized Enrichment Score (NES) | -0.8960563 |
| Nominal p-value | 0.7170068 |
| FDR q-value | 1.0 |
| FWER p-Value | 1.0 |
Table: GSEA Results Summary

  

Fig 1: Enrichment plot: BLALOCK\_ALZHEIMERS\_DISEASE\_INCIPIENT\_DN      
 Profile of the Running ES Score & Positions of GeneSet Members on the Rank Ordered List

  

| SYMBOL | TITLE | RANK IN GENE LIST | RANK METRIC SCORE | RUNNING ES | CORE ENRICHMENT || 1 | HSPA5 | na | 265 | 0.727 | 0.0071 | No |
| 2 | SAT1 | na | 383 | 0.640 | 0.0188 | No |
| 3 | RPL14 | na | 508 | 0.587 | 0.0287 | No |
| 4 | LAMTOR2 | na | 594 | 0.557 | 0.0396 | No |
| 5 | RPL6 | na | 808 | 0.497 | 0.0430 | No |
| 6 | RPL38 | na | 836 | 0.491 | 0.0548 | No |
| 7 | NEDD8 | na | 881 | 0.480 | 0.0655 | No |
| 8 | DLGAP2 | na | 962 | 0.469 | 0.0743 | No |
| 9 | MKKS | na | 1065 | 0.455 | 0.0817 | No |
| 10 | PDIA6 | na | 1066 | 0.455 | 0.0938 | No |
| 11 | REEP5 | na | 1082 | 0.452 | 0.1052 | No |
| 12 | COX7C | na | 1133 | 0.444 | 0.1147 | No |
| 13 | PFDN2 | na | 1141 | 0.443 | 0.1261 | No |
| 14 | NDUFB3 | na | 1166 | 0.439 | 0.1367 | No |
| 15 | COX7A2L | na | 1310 | 0.419 | 0.1413 | No |
| 16 | EIF2B3 | na | 1587 | 0.389 | 0.1389 | No |
| 17 | GTF2A2 | na | 1664 | 0.378 | 0.1455 | No |
| 18 | CACYBP | na | 1726 | 0.371 | 0.1525 | No |
| 19 | CKS2 | na | 1945 | 0.344 | 0.1516 | No |
| 20 | PSMC2 | na | 2133 | 0.323 | 0.1516 | No |
| 21 | COPS5 | na | 2152 | 0.321 | 0.1593 | No |
| 22 | GRPEL1 | na | 2175 | 0.320 | 0.1668 | No |
| 23 | MRPL15 | na | 2304 | 0.303 | 0.1690 | No |
| 24 | MPV17 | na | 2306 | 0.303 | 0.1770 | No |
| 25 | DRAP1 | na | 2368 | 0.297 | 0.1821 | No |
| 26 | CLDN10 | na | 2389 | 0.297 | 0.1891 | No |
| 27 | FHL1 | na | 2714 | 0.268 | 0.1813 | No |
| 28 | CCT3 | na | 2732 | 0.266 | 0.1876 | No |
| 29 | TMEM147 | na | 2778 | 0.261 | 0.1925 | No |
| 30 | PPA1 | na | 2866 | 0.254 | 0.1952 | No |
| 31 | EIF2B1 | na | 2871 | 0.253 | 0.2017 | No |
| 32 | HADH | na | 2941 | 0.247 | 0.2051 | No |
| 33 | ECH1 | na | 2968 | 0.244 | 0.2104 | No |
| 34 | CCT2 | na | 2987 | 0.243 | 0.2160 | No |
| 35 | UROS | na | 3032 | 0.238 | 0.2204 | No |
| 36 | CCT4 | na | 3202 | 0.223 | 0.2185 | No |
| 37 | UTP18 | na | 3231 | 0.220 | 0.2231 | No |
| 38 | CCT5 | na | 3233 | 0.220 | 0.2289 | No |
| 39 | DNAJC8 | na | 3428 | 0.204 | 0.2254 | No |
| 40 | MGLL | na | 3493 | 0.200 | 0.2277 | No |
| 41 | NDUFB8 | na | 3736 | 0.183 | 0.2215 | No |
| 42 | AP3S1 | na | 3816 | 0.179 | 0.2226 | No |
| 43 | DNAJA1 | na | 3904 | 0.173 | 0.2232 | No |
| 44 | CCNL1 | na | 3974 | 0.167 | 0.2244 | No |
| 45 | NSFL1C | na | 4080 | 0.157 | 0.2238 | No |
| 46 | MAOA | na | 4152 | 0.153 | 0.2246 | No |
| 47 | ALOX5AP | na | 4166 | 0.152 | 0.2280 | No |
| 48 | ACAD8 | na | 4623 | 0.120 | 0.2102 | No |
| 49 | PPP3CA | na | 4664 | 0.117 | 0.2115 | No |
| 50 | PGS1 | na | 4749 | 0.112 | 0.2106 | No |
| 51 | CEPT1 | na | 4899 | 0.101 | 0.2065 | No |
| 52 | AHSA1 | na | 4963 | 0.098 | 0.2062 | No |
| 53 | HSP90AB1 | na | 5242 | 0.082 | 0.1956 | No |
| 54 | HSP90AA1 | na | 5244 | 0.082 | 0.1977 | No |
| 55 | RPN2 | na | 5445 | 0.072 | 0.1904 | No |
| 56 | B4GALNT1 | na | 5564 | 0.065 | 0.1867 | No |
| 57 | CUL2 | na | 5581 | 0.064 | 0.1877 | No |
| 58 | GNAS | na | 5608 | 0.063 | 0.1881 | No |
| 59 | RGS4 | na | 5722 | 0.057 | 0.1844 | No |
| 60 | PDHX | na | 5976 | 0.042 | 0.1739 | No |
| 61 | PANX1 | na | 5980 | 0.042 | 0.1749 | No |
| 62 | GLRX | na | 6293 | 0.029 | 0.1613 | No |
| 63 | RGS20 | na | 6402 | 0.025 | 0.1570 | No |
| 64 | DEAF1 | na | 6422 | 0.024 | 0.1567 | No |
| 65 | FAIM2 | na | 6666 | 0.014 | 0.1459 | No |
| 66 | CDK7 | na | 6749 | 0.011 | 0.1425 | No |
| 67 | GNB1 | na | 6946 | 0.004 | 0.1335 | No |
| 68 | MED17 | na | 6980 | 0.002 | 0.1321 | No |
| 69 | TUBG2 | na | 7027 | 0.000 | 0.1299 | No |
| 70 | CRH | na | 7440 | 0.000 | 0.1110 | No |
| 71 | FSD1 | na | 8057 | 0.000 | 0.0826 | No |
| 72 | GPR22 | na | 8290 | 0.000 | 0.0719 | No |
| 73 | NELL1 | na | 8938 | 0.000 | 0.0422 | No |
| 74 | TAC1 | na | 8994 | 0.000 | 0.0396 | No |
| 75 | KCNIP1 | na | 9081 | 0.000 | 0.0357 | No |
| 76 | GABRB3 | na | 9325 | 0.000 | 0.0245 | No |
| 77 | TRO | na | 10287 | 0.000 | -0.0198 | No |
| 78 | TENM1 | na | 10337 | 0.000 | -0.0220 | No |
| 79 | TOX3 | na | 11692 | 0.000 | -0.0843 | No |
| 80 | PCP4 | na | 11738 | 0.000 | -0.0864 | No |
| 81 | ROBO1 | na | 12627 | 0.000 | -0.1273 | No |
| 82 | PAFAH1B1 | na | 13524 | -0.007 | -0.1683 | No |
| 83 | ALDH2 | na | 13607 | -0.010 | -0.1718 | No |
| 84 | PPP3R1 | na | 13636 | -0.011 | -0.1729 | No |
| 85 | RIMS2 | na | 13772 | -0.013 | -0.1787 | No |
| 86 | DDX5 | na | 13863 | -0.016 | -0.1824 | No |
| 87 | FKBP1B | na | 13889 | -0.017 | -0.1831 | No |
| 88 | ORC5 | na | 14139 | -0.025 | -0.1939 | No |
| 89 | CSNK1G3 | na | 14360 | -0.034 | -0.2031 | No |
| 90 | SDHA | na | 14573 | -0.044 | -0.2117 | No |
| 91 | HSPH1 | na | 14720 | -0.052 | -0.2171 | No |
| 92 | ZW10 | na | 14732 | -0.053 | -0.2162 | No |
| 93 | USP15 | na | 14971 | -0.066 | -0.2254 | No |
| 94 | DDN | na | 15226 | -0.081 | -0.2349 | No |
| 95 | RPE | na | 15244 | -0.082 | -0.2335 | No |
| 96 | CSNK2A1 | na | 15329 | -0.088 | -0.2350 | No |
| 97 | ACTR1B | na | 15346 | -0.089 | -0.2334 | No |
| 98 | MFN1 | na | 15688 | -0.108 | -0.2463 | No |
| 99 | ARHGEF4 | na | 15826 | -0.117 | -0.2495 | No |
| 100 | SEC23B | na | 15903 | -0.122 | -0.2497 | No |
| 101 | SST | na | 15959 | -0.126 | -0.2489 | No |
| 102 | PDHA1 | na | 16103 | -0.134 | -0.2519 | No |
| 103 | WFDC1 | na | 16200 | -0.140 | -0.2526 | No |
| 104 | ATIC | na | 16212 | -0.141 | -0.2493 | No |
| 105 | RAD51C | na | 16324 | -0.149 | -0.2505 | No |
| 106 | LGALS8 | na | 16864 | -0.187 | -0.2704 | No |
| 107 | RSU1 | na | 16872 | -0.187 | -0.2657 | No |
| 108 | MAP2K4 | na | 16904 | -0.189 | -0.2621 | No |
| 109 | GLUL | na | 17167 | -0.207 | -0.2687 | No |
| 110 | ARCN1 | na | 17439 | -0.226 | -0.2751 | No |
| 111 | MXD1 | na | 17671 | -0.242 | -0.2793 | Yes |
| 112 | VAMP1 | na | 17684 | -0.243 | -0.2734 | Yes |
| 113 | IPO7 | na | 17727 | -0.247 | -0.2688 | Yes |
| 114 | TOP1 | na | 17830 | -0.256 | -0.2667 | Yes |
| 115 | TFDP1 | na | 18032 | -0.271 | -0.2687 | Yes |
| 116 | COL5A2 | na | 18110 | -0.277 | -0.2649 | Yes |
| 117 | PAK3 | na | 18126 | -0.279 | -0.2582 | Yes |
| 118 | UBP1 | na | 18148 | -0.281 | -0.2517 | Yes |
| 119 | ZMAT3 | na | 18162 | -0.282 | -0.2448 | Yes |
| 120 | CLIP3 | na | 18165 | -0.282 | -0.2373 | Yes |
| 121 | FCER1G | na | 18170 | -0.282 | -0.2300 | Yes |
| 122 | MEF2A | na | 18192 | -0.284 | -0.2234 | Yes |
| 123 | SUPT7L | na | 18277 | -0.290 | -0.2196 | Yes |
| 124 | OPA1 | na | 18302 | -0.291 | -0.2130 | Yes |
| 125 | ZFR | na | 18342 | -0.294 | -0.2069 | Yes |
| 126 | PTPRR | na | 18352 | -0.294 | -0.1995 | Yes |
| 127 | ATP2A2 | na | 18364 | -0.295 | -0.1922 | Yes |
| 128 | PARP1 | na | 18772 | -0.327 | -0.2022 | Yes |
| 129 | SEC31A | na | 18793 | -0.329 | -0.1944 | Yes |
| 130 | RAPGEFL1 | na | 18912 | -0.340 | -0.1908 | Yes |
| 131 | CAMKK2 | na | 19237 | -0.372 | -0.1958 | Yes |
| 132 | TTC3 | na | 19271 | -0.375 | -0.1874 | Yes |
| 133 | ARF3 | na | 19670 | -0.414 | -0.1947 | Yes |
| 134 | ASPH | na | 19814 | -0.427 | -0.1899 | Yes |
| 135 | DENND4A | na | 19845 | -0.430 | -0.1799 | Yes |
| 136 | RB1CC1 | na | 19851 | -0.430 | -0.1686 | Yes |
| 137 | RGS14 | na | 19857 | -0.431 | -0.1574 | Yes |
| 138 | CDC27 | na | 19885 | -0.434 | -0.1471 | Yes |
| 139 | ELMO2 | na | 19901 | -0.435 | -0.1362 | Yes |
| 140 | RANBP2 | na | 20173 | -0.464 | -0.1363 | Yes |
| 141 | MGA | na | 20233 | -0.470 | -0.1266 | Yes |
| 142 | ACTN1 | na | 20635 | -0.521 | -0.1312 | Yes |
| 143 | ROCK2 | na | 20637 | -0.521 | -0.1174 | Yes |
| 144 | KIDINS220 | na | 20672 | -0.525 | -0.1050 | Yes |
| 145 | COL4A1 | na | 20907 | -0.561 | -0.1008 | Yes |
| 146 | KIF5C | na | 20939 | -0.567 | -0.0872 | Yes |
| 147 | PIP5K1C | na | 21145 | -0.607 | -0.0805 | Yes |
| 148 | KIF1B | na | 21147 | -0.607 | -0.0644 | Yes |
| 149 | APC | na | 21200 | -0.619 | -0.0503 | Yes |
| 150 | SCN3A | na | 21301 | -0.642 | -0.0378 | Yes |
| 151 | ADGRB2 | na | 21566 | -0.734 | -0.0304 | Yes |
| 152 | TMOD1 | na | 21650 | -0.790 | -0.0133 | Yes |
| 153 | PCNT | na | 21739 | -0.887 | 0.0063 | Yes |
Table: GSEA details [plain text format]

  

Fig 2: BLALOCK\_ALZHEIMERS\_DISEASE\_INCIPIENT\_DN      
 Blue-Pink O' Gram in the Space of the Analyzed GeneSet

  

Fig 3: BLALOCK\_ALZHEIMERS\_DISEASE\_INCIPIENT\_DN: Random ES distribution      
 Gene set null distribution of ES for **BLALOCK\_ALZHEIMERS\_DISEASE\_INCIPIENT\_DN**

  
