# Supplementary material for: Regulation of microglia related neuroinflammation contributes to the protective effect of Gelsevirine on ischemic stroke
Source: Front Immunol. 2023 Mar 30;14:1164278. doi: 10.3389/fimmu.2023.1164278 (PMC10098192; doi:10.3389/fimmu.2023.1164278)
Supplement: Supplementary file 6 [file DataSheet_6.zip › fig 5 raw/fig 5-G raw/inflammation.Gsea.1649955013530/BLALOCK_ALZHEIMERS_DISEASE_INCIPIENT_UP.html]

Details for gene set BLALOCK\_ALZHEIMERS\_DISEASE\_INCIPIENT\_UP[GSEA]

|  || Dataset | OGD\_DRUG\_DRUG.OGD\_FRUG.cls#Gs\_versus\_MCAO.OGD\_FRUG.cls#Gs\_versus\_MCAO\_repos |
| Phenotype | OGD\_FRUG.cls#Gs\_versus\_MCAO\_repos |
| Upregulated in class | MCAO |
| GeneSet | BLALOCK\_ALZHEIMERS\_DISEASE\_INCIPIENT\_UP |
| Enrichment Score (ES) | -0.5008197 |
| Normalized Enrichment Score (NES) | -1.6779603 |
| Nominal p-value | 0.0 |
| FDR q-value | 0.011147403 |
| FWER p-Value | 0.011 |
Table: GSEA Results Summary

  

Fig 1: Enrichment plot: BLALOCK\_ALZHEIMERS\_DISEASE\_INCIPIENT\_UP      
 Profile of the Running ES Score & Positions of GeneSet Members on the Rank Ordered List

  

| SYMBOL | TITLE | RANK IN GENE LIST | RANK METRIC SCORE | RUNNING ES | CORE ENRICHMENT || 1 | DCN | na | 328 | 0.673 | -0.0076 | No |
| 2 | S100A4 | na | 373 | 0.645 | -0.0023 | No |
| 3 | SFN | na | 603 | 0.554 | -0.0066 | No |
| 4 | CLU | na | 801 | 0.498 | -0.0101 | No |
| 5 | SYF2 | na | 812 | 0.495 | -0.0049 | No |
| 6 | ANP32B | na | 849 | 0.488 | -0.0010 | No |
| 7 | LSM5 | na | 924 | 0.474 | 0.0010 | No |
| 8 | RNASEH2A | na | 1025 | 0.461 | 0.0016 | No |
| 9 | CTSL | na | 1068 | 0.455 | 0.0048 | No |
| 10 | CTSS | na | 1108 | 0.447 | 0.0081 | No |
| 11 | LARP7 | na | 1111 | 0.446 | 0.0131 | No |
| 12 | RBM8A | na | 1217 | 0.433 | 0.0131 | No |
| 13 | EIF5B | na | 1273 | 0.425 | 0.0154 | No |
| 14 | PRDX6 | na | 1373 | 0.413 | 0.0155 | No |
| 15 | SSR2 | na | 1477 | 0.402 | 0.0153 | No |
| 16 | NT5C | na | 1491 | 0.401 | 0.0193 | No |
| 17 | TSR3 | na | 1584 | 0.390 | 0.0194 | No |
| 18 | RPL13 | na | 1787 | 0.364 | 0.0142 | No |
| 19 | CPQ | na | 1798 | 0.362 | 0.0179 | No |
| 20 | NAP1L1 | na | 1858 | 0.355 | 0.0192 | No |
| 21 | DGCR6 | na | 1864 | 0.354 | 0.0230 | No |
| 22 | UROD | na | 2047 | 0.333 | 0.0183 | No |
| 23 | CLIC1 | na | 2135 | 0.323 | 0.0179 | No |
| 24 | EMID1 | na | 2406 | 0.295 | 0.0088 | No |
| 25 | TEX264 | na | 2469 | 0.289 | 0.0092 | No |
| 26 | LUC7L3 | na | 2536 | 0.283 | 0.0093 | No |
| 27 | PGLS | na | 2585 | 0.280 | 0.0103 | No |
| 28 | PSMC3IP | na | 2720 | 0.268 | 0.0071 | No |
| 29 | CSNK2A2 | na | 2818 | 0.258 | 0.0055 | No |
| 30 | GAMT | na | 2892 | 0.251 | 0.0050 | No |
| 31 | PGM3 | na | 2933 | 0.247 | 0.0060 | No |
| 32 | PTGIS | na | 2983 | 0.243 | 0.0065 | No |
| 33 | CDK2AP1 | na | 3049 | 0.237 | 0.0061 | No |
| 34 | TSPAN31 | na | 3247 | 0.219 | -0.0005 | No |
| 35 | HOXC4 | na | 3321 | 0.213 | -0.0015 | No |
| 36 | FAM3A | na | 3361 | 0.210 | -0.0009 | No |
| 37 | ATG3 | na | 3533 | 0.197 | -0.0066 | No |
| 38 | SNX16 | na | 3552 | 0.195 | -0.0052 | No |
| 39 | RAB2A | na | 3566 | 0.194 | -0.0036 | No |
| 40 | RAD1 | na | 3626 | 0.191 | -0.0042 | No |
| 41 | LAMA4 | na | 3690 | 0.187 | -0.0050 | No |
| 42 | PDE4C | na | 3729 | 0.184 | -0.0046 | No |
| 43 | CYP11A1 | na | 3771 | 0.181 | -0.0045 | No |
| 44 | CCDC59 | na | 3793 | 0.180 | -0.0034 | No |
| 45 | SGK2 | na | 3858 | 0.176 | -0.0044 | No |
| 46 | COTL1 | na | 4286 | 0.144 | -0.0225 | No |
| 47 | IFT74 | na | 4331 | 0.141 | -0.0230 | No |
| 48 | DBF4 | na | 4540 | 0.126 | -0.0312 | No |
| 49 | ARPC1B | na | 4583 | 0.123 | -0.0317 | No |
| 50 | PPIG | na | 4595 | 0.122 | -0.0309 | No |
| 51 | SLC19A1 | na | 4731 | 0.113 | -0.0358 | No |
| 52 | FBXW4 | na | 4734 | 0.113 | -0.0347 | No |
| 53 | TBXA2R | na | 5060 | 0.092 | -0.0487 | No |
| 54 | SEC63 | na | 5122 | 0.089 | -0.0505 | No |
| 55 | CTPS2 | na | 5232 | 0.083 | -0.0546 | No |
| 56 | CNPY3 | na | 5248 | 0.082 | -0.0544 | No |
| 57 | PDCL | na | 5399 | 0.074 | -0.0605 | No |
| 58 | DAO | na | 5429 | 0.072 | -0.0610 | No |
| 59 | EFEMP2 | na | 5513 | 0.067 | -0.0641 | No |
| 60 | EFEMP1 | na | 5516 | 0.067 | -0.0634 | No |
| 61 | KLF2 | na | 5535 | 0.066 | -0.0635 | No |
| 62 | TPD52 | na | 5561 | 0.065 | -0.0639 | No |
| 63 | NSDHL | na | 5618 | 0.062 | -0.0658 | No |
| 64 | PDSS1 | na | 5636 | 0.061 | -0.0659 | No |
| 65 | HMGB3 | na | 5736 | 0.056 | -0.0699 | No |
| 66 | SNX11 | na | 5738 | 0.056 | -0.0693 | No |
| 67 | GPSM3 | na | 5891 | 0.047 | -0.0758 | No |
| 68 | GIPC1 | na | 5996 | 0.041 | -0.0802 | No |
| 69 | KCNJ14 | na | 6053 | 0.038 | -0.0823 | No |
| 70 | NCAPG | na | 6075 | 0.037 | -0.0829 | No |
| 71 | ARHGEF15 | na | 6138 | 0.035 | -0.0854 | No |
| 72 | ERAL1 | na | 6178 | 0.033 | -0.0868 | No |
| 73 | TSC22D4 | na | 6203 | 0.032 | -0.0875 | No |
| 74 | RAB1B | na | 6236 | 0.031 | -0.0887 | No |
| 75 | TUBD1 | na | 6268 | 0.030 | -0.0898 | No |
| 76 | UGT1A10 | na | 6336 | 0.027 | -0.0926 | No |
| 77 | RBCK1 | na | 6468 | 0.022 | -0.0984 | No |
| 78 | ID4 | na | 6472 | 0.022 | -0.0983 | No |
| 79 | MATN2 | na | 6510 | 0.020 | -0.0998 | No |
| 80 | NUCKS1 | na | 6573 | 0.018 | -0.1025 | No |
| 81 | SAP30BP | na | 6669 | 0.014 | -0.1067 | No |
| 82 | SFRP1 | na | 6881 | 0.006 | -0.1164 | No |
| 83 | IL13RA1 | na | 6882 | 0.006 | -0.1164 | No |
| 84 | FYN | na | 6884 | 0.006 | -0.1163 | No |
| 85 | IL10RB | na | 6952 | 0.003 | -0.1194 | No |
| 86 | GTF2H1 | na | 6988 | 0.002 | -0.1210 | No |
| 87 | SLC35F2 | na | 7025 | 0.000 | -0.1227 | No |
| 88 | CEBPA | na | 7443 | 0.000 | -0.1420 | No |
| 89 | APOC4 | na | 7457 | 0.000 | -0.1426 | No |
| 90 | MAB21L2 | na | 7467 | 0.000 | -0.1431 | No |
| 91 | TNFRSF11B | na | 7622 | 0.000 | -0.1502 | No |
| 92 | DMC1 | na | 8082 | 0.000 | -0.1715 | No |
| 93 | PCDH11X | na | 8102 | 0.000 | -0.1724 | No |
| 94 | GABRQ | na | 8219 | 0.000 | -0.1778 | No |
| 95 | MLANA | na | 8564 | 0.000 | -0.1937 | No |
| 96 | KCNK10 | na | 9237 | 0.000 | -0.2249 | No |
| 97 | ASCL1 | na | 9337 | 0.000 | -0.2295 | No |
| 98 | IFNA5 | na | 9402 | 0.000 | -0.2325 | No |
| 99 | PRL | na | 9408 | 0.000 | -0.2327 | No |
| 100 | CLDN5 | na | 9467 | 0.000 | -0.2354 | No |
| 101 | CTRC | na | 9560 | 0.000 | -0.2397 | No |
| 102 | BRS3 | na | 9791 | 0.000 | -0.2504 | No |
| 103 | IFNG | na | 9828 | 0.000 | -0.2521 | No |
| 104 | IL10RA | na | 10241 | 0.000 | -0.2712 | No |
| 105 | GDF1 | na | 10742 | 0.000 | -0.2944 | No |
| 106 | FBN2 | na | 10970 | 0.000 | -0.3049 | No |
| 107 | ANGPT1 | na | 11116 | 0.000 | -0.3117 | No |
| 108 | TRIM38 | na | 11673 | 0.000 | -0.3375 | No |
| 109 | GLI2 | na | 12145 | 0.000 | -0.3593 | No |
| 110 | EDNRA | na | 12199 | 0.000 | -0.3618 | No |
| 111 | SLAMF1 | na | 12876 | 0.000 | -0.3932 | No |
| 112 | PI4K2A | na | 13264 | -0.000 | -0.4111 | No |
| 113 | ITGA7 | na | 13508 | -0.007 | -0.4223 | No |
| 114 | TCN2 | na | 13536 | -0.008 | -0.4235 | No |
| 115 | HAO2 | na | 13625 | -0.011 | -0.4275 | No |
| 116 | FGL1 | na | 13628 | -0.011 | -0.4274 | No |
| 117 | JUND | na | 13688 | -0.012 | -0.4300 | No |
| 118 | CSDE1 | na | 13691 | -0.012 | -0.4300 | No |
| 119 | APLNR | na | 13937 | -0.018 | -0.4412 | No |
| 120 | ACTR8 | na | 14003 | -0.020 | -0.4439 | No |
| 121 | RGS9 | na | 14044 | -0.021 | -0.4456 | No |
| 122 | GNAZ | na | 14099 | -0.024 | -0.4478 | No |
| 123 | NEBL | na | 14199 | -0.028 | -0.4521 | No |
| 124 | CDC5L | na | 14269 | -0.030 | -0.4549 | No |
| 125 | GRM6 | na | 14351 | -0.034 | -0.4583 | No |
| 126 | NAMPT | na | 14416 | -0.036 | -0.4609 | No |
| 127 | EWSR1 | na | 14527 | -0.042 | -0.4655 | No |
| 128 | RXRB | na | 14636 | -0.047 | -0.4700 | No |
| 129 | SERTAD3 | na | 14791 | -0.056 | -0.4765 | No |
| 130 | RHOQ | na | 14853 | -0.059 | -0.4787 | No |
| 131 | FADS1 | na | 14858 | -0.059 | -0.4782 | No |
| 132 | PER1 | na | 14861 | -0.059 | -0.4776 | No |
| 133 | COG4 | na | 14885 | -0.061 | -0.4780 | No |
| 134 | CPM | na | 14993 | -0.067 | -0.4822 | No |
| 135 | NONO | na | 15028 | -0.070 | -0.4829 | No |
| 136 | MR1 | na | 15047 | -0.071 | -0.4830 | No |
| 137 | SSH3 | na | 15066 | -0.072 | -0.4830 | No |
| 138 | OS9 | na | 15080 | -0.073 | -0.4828 | No |
| 139 | BRAP | na | 15086 | -0.073 | -0.4821 | No |
| 140 | CALML4 | na | 15115 | -0.075 | -0.4826 | No |
| 141 | GPR107 | na | 15173 | -0.078 | -0.4844 | No |
| 142 | POLH | na | 15203 | -0.080 | -0.4848 | No |
| 143 | HOXB5 | na | 15205 | -0.080 | -0.4839 | No |
| 144 | WWP2 | na | 15239 | -0.082 | -0.4845 | No |
| 145 | ARFGAP2 | na | 15252 | -0.083 | -0.4841 | No |
| 146 | SLC12A3 | na | 15257 | -0.084 | -0.4834 | No |
| 147 | KTN1 | na | 15296 | -0.086 | -0.4841 | No |
| 148 | GTF2H3 | na | 15340 | -0.088 | -0.4851 | No |
| 149 | ST6GALNAC4 | na | 15344 | -0.089 | -0.4843 | No |
| 150 | OGG1 | na | 15414 | -0.093 | -0.4864 | No |
| 151 | ARFGAP1 | na | 15423 | -0.094 | -0.4857 | No |
| 152 | NMB | na | 15427 | -0.094 | -0.4848 | No |
| 153 | TRMT1L | na | 15446 | -0.095 | -0.4845 | No |
| 154 | DAPK2 | na | 15489 | -0.097 | -0.4854 | No |
| 155 | INPP5K | na | 15497 | -0.097 | -0.4846 | No |
| 156 | USP1 | na | 15563 | -0.102 | -0.4865 | No |
| 157 | TAX1BP3 | na | 15709 | -0.109 | -0.4920 | No |
| 158 | GOSR2 | na | 15745 | -0.112 | -0.4923 | No |
| 159 | CAST | na | 15855 | -0.118 | -0.4960 | No |
| 160 | USH1C | na | 15871 | -0.120 | -0.4954 | No |
| 161 | SCLY | na | 15931 | -0.124 | -0.4967 | No |
| 162 | WAS | na | 15945 | -0.125 | -0.4959 | No |
| 163 | TLR1 | na | 15960 | -0.126 | -0.4951 | No |
| 164 | TNFRSF9 | na | 15985 | -0.127 | -0.4947 | No |
| 165 | KDSR | na | 16055 | -0.132 | -0.4964 | No |
| 166 | AGFG2 | na | 16093 | -0.134 | -0.4966 | No |
| 167 | RHBDD3 | na | 16184 | -0.140 | -0.4992 | Yes |
| 168 | SLC14A1 | na | 16219 | -0.142 | -0.4992 | Yes |
| 169 | POLR1B | na | 16221 | -0.142 | -0.4976 | Yes |
| 170 | VRK3 | na | 16239 | -0.143 | -0.4968 | Yes |
| 171 | SGK1 | na | 16243 | -0.143 | -0.4953 | Yes |
| 172 | PRPF40A | na | 16270 | -0.145 | -0.4948 | Yes |
| 173 | SPAG1 | na | 16275 | -0.145 | -0.4934 | Yes |
| 174 | CYP39A1 | na | 16358 | -0.152 | -0.4955 | Yes |
| 175 | SMC1A | na | 16363 | -0.152 | -0.4939 | Yes |
| 176 | CPT2 | na | 16367 | -0.152 | -0.4923 | Yes |
| 177 | YES1 | na | 16373 | -0.153 | -0.4908 | Yes |
| 178 | TOPORS | na | 16504 | -0.162 | -0.4950 | Yes |
| 179 | ANGPTL2 | na | 16574 | -0.167 | -0.4963 | Yes |
| 180 | FZR1 | na | 16600 | -0.169 | -0.4955 | Yes |
| 181 | PRCP | na | 16606 | -0.169 | -0.4938 | Yes |
| 182 | CASP6 | na | 16611 | -0.170 | -0.4921 | Yes |
| 183 | ZMYM5 | na | 16625 | -0.171 | -0.4907 | Yes |
| 184 | PTGS1 | na | 16639 | -0.172 | -0.4894 | Yes |
| 185 | IPP | na | 16690 | -0.176 | -0.4897 | Yes |
| 186 | IL17RA | na | 16705 | -0.177 | -0.4883 | Yes |
| 187 | CD44 | na | 16720 | -0.178 | -0.4870 | Yes |
| 188 | MDM1 | na | 16723 | -0.178 | -0.4850 | Yes |
| 189 | IL18 | na | 16753 | -0.179 | -0.4843 | Yes |
| 190 | ENAH | na | 16759 | -0.180 | -0.4825 | Yes |
| 191 | EPS8 | na | 16771 | -0.180 | -0.4810 | Yes |
| 192 | LEF1 | na | 16773 | -0.181 | -0.4790 | Yes |
| 193 | BGN | na | 16804 | -0.183 | -0.4783 | Yes |
| 194 | PIBF1 | na | 16815 | -0.183 | -0.4766 | Yes |
| 195 | WWTR1 | na | 16841 | -0.185 | -0.4757 | Yes |
| 196 | EIF2AK2 | na | 16842 | -0.185 | -0.4736 | Yes |
| 197 | PLOD2 | na | 16918 | -0.190 | -0.4749 | Yes |
| 198 | TM7SF3 | na | 16960 | -0.194 | -0.4746 | Yes |
| 199 | APOC1 | na | 17015 | -0.197 | -0.4749 | Yes |
| 200 | NEK7 | na | 17081 | -0.201 | -0.4756 | Yes |
| 201 | ARHGAP17 | na | 17137 | -0.204 | -0.4758 | Yes |
| 202 | STAG2 | na | 17164 | -0.206 | -0.4747 | Yes |
| 203 | PAH | na | 17206 | -0.210 | -0.4742 | Yes |
| 204 | RNGTT | na | 17292 | -0.216 | -0.4757 | Yes |
| 205 | SEMA3B | na | 17328 | -0.219 | -0.4748 | Yes |
| 206 | LIMK2 | na | 17333 | -0.219 | -0.4725 | Yes |
| 207 | PTH1R | na | 17337 | -0.219 | -0.4701 | Yes |
| 208 | GTF2I | na | 17404 | -0.224 | -0.4707 | Yes |
| 209 | GNAI3 | na | 17440 | -0.226 | -0.4697 | Yes |
| 210 | SLC35A3 | na | 17456 | -0.227 | -0.4678 | Yes |
| 211 | GJA1 | na | 17476 | -0.228 | -0.4661 | Yes |
| 212 | LPAR4 | na | 17527 | -0.232 | -0.4658 | Yes |
| 213 | PDGFB | na | 17563 | -0.234 | -0.4647 | Yes |
| 214 | CTDSP1 | na | 17616 | -0.238 | -0.4645 | Yes |
| 215 | NCAPD2 | na | 17622 | -0.238 | -0.4620 | Yes |
| 216 | SPIB | na | 17725 | -0.247 | -0.4639 | Yes |
| 217 | FLII | na | 17750 | -0.248 | -0.4622 | Yes |
| 218 | PI4KB | na | 17757 | -0.249 | -0.4596 | Yes |
| 219 | MYO1A | na | 17768 | -0.250 | -0.4572 | Yes |
| 220 | DLX4 | na | 17769 | -0.250 | -0.4544 | Yes |
| 221 | RABEP1 | na | 17851 | -0.257 | -0.4552 | Yes |
| 222 | CFLAR | na | 17887 | -0.260 | -0.4539 | Yes |
| 223 | F2R | na | 17939 | -0.265 | -0.4532 | Yes |
| 224 | SRPK2 | na | 17949 | -0.266 | -0.4506 | Yes |
| 225 | RBBP5 | na | 17953 | -0.266 | -0.4477 | Yes |
| 226 | CDC42EP4 | na | 17966 | -0.267 | -0.4453 | Yes |
| 227 | CSF1 | na | 17979 | -0.268 | -0.4428 | Yes |
| 228 | ZMYM2 | na | 17982 | -0.268 | -0.4398 | Yes |
| 229 | SLC35C1 | na | 17997 | -0.269 | -0.4374 | Yes |
| 230 | HS2ST1 | na | 18024 | -0.271 | -0.4355 | Yes |
| 231 | PLD2 | na | 18051 | -0.273 | -0.4336 | Yes |
| 232 | CUL4A | na | 18105 | -0.277 | -0.4329 | Yes |
| 233 | PHF20 | na | 18124 | -0.279 | -0.4306 | Yes |
| 234 | GGCX | na | 18272 | -0.289 | -0.4341 | Yes |
| 235 | TCOF1 | na | 18301 | -0.291 | -0.4321 | Yes |
| 236 | SREBF1 | na | 18341 | -0.294 | -0.4306 | Yes |
| 237 | MALT1 | na | 18439 | -0.299 | -0.4317 | Yes |
| 238 | RBBP6 | na | 18481 | -0.302 | -0.4301 | Yes |
| 239 | CHD2 | na | 18509 | -0.304 | -0.4279 | Yes |
| 240 | ARID4A | na | 18649 | -0.317 | -0.4308 | Yes |
| 241 | JRK | na | 18661 | -0.317 | -0.4277 | Yes |
| 242 | IL2RG | na | 18663 | -0.317 | -0.4241 | Yes |
| 243 | ERF | na | 18692 | -0.320 | -0.4218 | Yes |
| 244 | CTDSP2 | na | 18810 | -0.331 | -0.4234 | Yes |
| 245 | PPP1R13L | na | 18829 | -0.332 | -0.4205 | Yes |
| 246 | OSBPL11 | na | 18862 | -0.336 | -0.4181 | Yes |
| 247 | APP | na | 18882 | -0.338 | -0.4152 | Yes |
| 248 | ERAP1 | na | 18928 | -0.342 | -0.4134 | Yes |
| 249 | RUNX2 | na | 19134 | -0.363 | -0.4187 | Yes |
| 250 | ZBTB10 | na | 19150 | -0.365 | -0.4153 | Yes |
| 251 | CPSF1 | na | 19182 | -0.368 | -0.4125 | Yes |
| 252 | NKTR | na | 19197 | -0.369 | -0.4090 | Yes |
| 253 | SP3 | na | 19231 | -0.372 | -0.4063 | Yes |
| 254 | PRPF4B | na | 19244 | -0.373 | -0.4026 | Yes |
| 255 | TCF3 | na | 19312 | -0.378 | -0.4014 | Yes |
| 256 | ISLR | na | 19352 | -0.382 | -0.3989 | Yes |
| 257 | PXN | na | 19391 | -0.386 | -0.3962 | Yes |
| 258 | RBPJ | na | 19556 | -0.403 | -0.3993 | Yes |
| 259 | KLHL20 | na | 19604 | -0.407 | -0.3968 | Yes |
| 260 | TRIM44 | na | 19637 | -0.410 | -0.3936 | Yes |
| 261 | FGFR3 | na | 19694 | -0.416 | -0.3915 | Yes |
| 262 | DICER1 | na | 19713 | -0.418 | -0.3876 | Yes |
| 263 | PTBP3 | na | 19742 | -0.420 | -0.3841 | Yes |
| 264 | BRD1 | na | 19748 | -0.421 | -0.3795 | Yes |
| 265 | TAOK2 | na | 19752 | -0.421 | -0.3748 | Yes |
| 266 | GOLGA4 | na | 19773 | -0.424 | -0.3710 | Yes |
| 267 | ERLIN2 | na | 19803 | -0.426 | -0.3674 | Yes |
| 268 | SYCP2 | na | 19842 | -0.429 | -0.3643 | Yes |
| 269 | KAT2B | na | 19843 | -0.430 | -0.3594 | Yes |
| 270 | USP19 | na | 19892 | -0.435 | -0.3567 | Yes |
| 271 | CPNE3 | na | 20017 | -0.446 | -0.3574 | Yes |
| 272 | NCOA3 | na | 20068 | -0.451 | -0.3546 | Yes |
| 273 | ADCY7 | na | 20088 | -0.453 | -0.3503 | Yes |
| 274 | TACC1 | na | 20090 | -0.454 | -0.3452 | Yes |
| 275 | GSK3B | na | 20096 | -0.455 | -0.3402 | Yes |
| 276 | LAMA2 | na | 20114 | -0.457 | -0.3358 | Yes |
| 277 | PLAG1 | na | 20126 | -0.458 | -0.3311 | Yes |
| 278 | JAK3 | na | 20139 | -0.460 | -0.3264 | Yes |
| 279 | TGFBR3 | na | 20195 | -0.466 | -0.3237 | Yes |
| 280 | ITGA6 | na | 20245 | -0.471 | -0.3206 | Yes |
| 281 | MED1 | na | 20248 | -0.472 | -0.3153 | Yes |
| 282 | NUP98 | na | 20273 | -0.474 | -0.3110 | Yes |
| 283 | FNBP1 | na | 20296 | -0.477 | -0.3066 | Yes |
| 284 | ATP8B1 | na | 20324 | -0.480 | -0.3024 | Yes |
| 285 | NBAS | na | 20326 | -0.480 | -0.2970 | Yes |
| 286 | TNS3 | na | 20379 | -0.485 | -0.2938 | Yes |
| 287 | CDC14A | na | 20387 | -0.486 | -0.2886 | Yes |
| 288 | SSH1 | na | 20496 | -0.502 | -0.2879 | Yes |
| 289 | THOC2 | na | 20513 | -0.503 | -0.2829 | Yes |
| 290 | COL8A2 | na | 20547 | -0.508 | -0.2787 | Yes |
| 291 | PML | na | 20576 | -0.512 | -0.2742 | Yes |
| 292 | HNRNPUL1 | na | 20645 | -0.522 | -0.2714 | Yes |
| 293 | ADD3 | na | 20650 | -0.522 | -0.2656 | Yes |
| 294 | SSPN | na | 20733 | -0.535 | -0.2633 | Yes |
| 295 | MET | na | 20741 | -0.536 | -0.2576 | Yes |
| 296 | SMARCC2 | na | 20750 | -0.537 | -0.2518 | Yes |
| 297 | PRKAR2A | na | 20760 | -0.539 | -0.2461 | Yes |
| 298 | RBL1 | na | 20834 | -0.551 | -0.2432 | Yes |
| 299 | NOTCH4 | na | 20914 | -0.562 | -0.2405 | Yes |
| 300 | ZC3H7B | na | 20915 | -0.563 | -0.2341 | Yes |
| 301 | TTN | na | 20933 | -0.565 | -0.2284 | Yes |
| 302 | SMARCC1 | na | 20934 | -0.565 | -0.2220 | Yes |
| 303 | ARID1A | na | 20938 | -0.566 | -0.2157 | Yes |
| 304 | ZFP36L2 | na | 20941 | -0.567 | -0.2093 | Yes |
| 305 | PBRM1 | na | 20989 | -0.576 | -0.2049 | Yes |
| 306 | ALMS1 | na | 21050 | -0.588 | -0.2010 | Yes |
| 307 | NDST1 | na | 21098 | -0.598 | -0.1964 | Yes |
| 308 | AKAP9 | na | 21119 | -0.601 | -0.1905 | Yes |
| 309 | ADGRA3 | na | 21122 | -0.602 | -0.1837 | Yes |
| 310 | TBC1D5 | na | 21125 | -0.603 | -0.1769 | Yes |
| 311 | FUT2 | na | 21171 | -0.611 | -0.1721 | Yes |
| 312 | PLXNB2 | na | 21175 | -0.614 | -0.1652 | Yes |
| 313 | ZHX3 | na | 21180 | -0.615 | -0.1584 | Yes |
| 314 | TNC | na | 21238 | -0.628 | -0.1539 | Yes |
| 315 | LAMC1 | na | 21239 | -0.629 | -0.1467 | Yes |
| 316 | SNRK | na | 21284 | -0.639 | -0.1415 | Yes |
| 317 | NUMA1 | na | 21305 | -0.643 | -0.1351 | Yes |
| 318 | VCAN | na | 21332 | -0.652 | -0.1289 | Yes |
| 319 | CEP350 | na | 21362 | -0.658 | -0.1227 | Yes |
| 320 | FBN1 | na | 21403 | -0.669 | -0.1170 | Yes |
| 321 | AGO1 | na | 21410 | -0.673 | -0.1096 | Yes |
| 322 | EP300 | na | 21416 | -0.674 | -0.1022 | Yes |
| 323 | DIP2A | na | 21421 | -0.675 | -0.0947 | Yes |
| 324 | MYO10 | na | 21477 | -0.696 | -0.0893 | Yes |
| 325 | FLNC | na | 21608 | -0.761 | -0.0867 | Yes |
| 326 | AKAP13 | na | 21642 | -0.784 | -0.0793 | Yes |
| 327 | SYNC | na | 21668 | -0.807 | -0.0712 | Yes |
| 328 | NFIC | na | 21719 | -0.864 | -0.0637 | Yes |
| 329 | NPAS3 | na | 21735 | -0.881 | -0.0544 | Yes |
| 330 | MBP | na | 21744 | -0.895 | -0.0446 | Yes |
| 331 | GMPR | na | 21791 | -1.024 | -0.0350 | Yes |
| 332 | ITGA1 | na | 21792 | -1.028 | -0.0233 | Yes |
| 333 | IKZF4 | na | 21798 | -1.048 | -0.0116 | Yes |
| 334 | CLDN18 | na | 21854 | -1.329 | 0.0010 | Yes |
Table: GSEA details [plain text format]

  

Fig 2: BLALOCK\_ALZHEIMERS\_DISEASE\_INCIPIENT\_UP      
 Blue-Pink O' Gram in the Space of the Analyzed GeneSet

  

Fig 3: BLALOCK\_ALZHEIMERS\_DISEASE\_INCIPIENT\_UP: Random ES distribution      
 Gene set null distribution of ES for **BLALOCK\_ALZHEIMERS\_DISEASE\_INCIPIENT\_UP**

  
