# Supplementary material for: Regulation of microglia related neuroinflammation contributes to the protective effect of Gelsevirine on ischemic stroke
Source: Front Immunol. 2023 Mar 30;14:1164278. doi: 10.3389/fimmu.2023.1164278 (PMC10098192; doi:10.3389/fimmu.2023.1164278)
Supplement: Supplementary file 6 [file DataSheet_6.zip › fig 5 raw/fig 5-G raw/inflammation.Gsea.1649955013530/DEMAGALHAES_AGING_UP.html]

Details for gene set DEMAGALHAES\_AGING\_UP[GSEA]

|  || Dataset | OGD\_DRUG\_DRUG.OGD\_FRUG.cls#Gs\_versus\_MCAO.OGD\_FRUG.cls#Gs\_versus\_MCAO\_repos |
| Phenotype | OGD\_FRUG.cls#Gs\_versus\_MCAO\_repos |
| Upregulated in class | Gs |
| GeneSet | DEMAGALHAES\_AGING\_UP |
| Enrichment Score (ES) | 0.3649141 |
| Normalized Enrichment Score (NES) | 1.0510118 |
| Nominal p-value | 0.35119048 |
| FDR q-value | 0.8608415 |
| FWER p-Value | 0.818 |
Table: GSEA Results Summary

  

Fig 1: Enrichment plot: DEMAGALHAES\_AGING\_UP      
 Profile of the Running ES Score & Positions of GeneSet Members on the Rank Ordered List

  

| SYMBOL | TITLE | RANK IN GENE LIST | RANK METRIC SCORE | RUNNING ES | CORE ENRICHMENT || 1 | S100A4 | na | 373 | 0.645 | 0.0364 | Yes |
| 2 | S100A6 | na | 460 | 0.607 | 0.0828 | Yes |
| 3 | LGALS3 | na | 585 | 0.559 | 0.1234 | Yes |
| 4 | MGST1 | na | 682 | 0.531 | 0.1631 | Yes |
| 5 | CLU | na | 801 | 0.498 | 0.1989 | Yes |
| 6 | CTSS | na | 1108 | 0.447 | 0.2220 | Yes |
| 7 | B2M | na | 1349 | 0.415 | 0.2454 | Yes |
| 8 | ANXA3 | na | 1923 | 0.346 | 0.2479 | Yes |
| 9 | APOD | na | 2322 | 0.301 | 0.2546 | Yes |
| 10 | PTGES3 | na | 2335 | 0.301 | 0.2791 | Yes |
| 11 | ANXA5 | na | 2403 | 0.296 | 0.3005 | Yes |
| 12 | NPC2 | na | 2459 | 0.291 | 0.3221 | Yes |
| 13 | C4A | na | 2679 | 0.271 | 0.3346 | Yes |
| 14 | C3 | na | 3048 | 0.237 | 0.3374 | Yes |
| 15 | DERL1 | na | 3133 | 0.230 | 0.3526 | Yes |
| 16 | SPP1 | na | 3259 | 0.218 | 0.3649 | Yes |
| 17 | PSMD11 | na | 4094 | 0.156 | 0.3397 | No |
| 18 | NDRG1 | na | 4142 | 0.154 | 0.3503 | No |
| 19 | LITAF | na | 4262 | 0.146 | 0.3569 | No |
| 20 | GBP2 | na | 4854 | 0.105 | 0.3385 | No |
| 21 | EFEMP1 | na | 5516 | 0.067 | 0.3139 | No |
| 22 | TMED10 | na | 5569 | 0.065 | 0.3168 | No |
| 23 | IL33 | na | 6879 | 0.006 | 0.2574 | No |
| 24 | C1QA | na | 9422 | 0.000 | 0.1409 | No |
| 25 | C1QC | na | 9432 | 0.000 | 0.1405 | No |
| 26 | HCST | na | 9887 | 0.000 | 0.1197 | No |
| 27 | GSTA1 | na | 13344 | -0.003 | -0.0384 | No |
| 28 | FCGR2B | na | 14786 | -0.055 | -0.0998 | No |
| 29 | PCSK6 | na | 14864 | -0.059 | -0.0984 | No |
| 30 | TXNIP | na | 14977 | -0.066 | -0.0980 | No |
| 31 | VAT1 | na | 15017 | -0.069 | -0.0941 | No |
| 32 | GPNMB | na | 15220 | -0.081 | -0.0966 | No |
| 33 | JCHAIN | na | 15248 | -0.083 | -0.0910 | No |
| 34 | SGK1 | na | 16243 | -0.143 | -0.1247 | No |
| 35 | VWF | na | 16951 | -0.193 | -0.1411 | No |
| 36 | C1QB | na | 17277 | -0.215 | -0.1382 | No |
| 37 | GFAP | na | 17300 | -0.216 | -0.1212 | No |
| 38 | GNS | na | 17974 | -0.267 | -0.1299 | No |
| 39 | MPEG1 | na | 18014 | -0.270 | -0.1092 | No |
| 40 | CLIC4 | na | 18300 | -0.291 | -0.0981 | No |
| 41 | LAPTM5 | na | 18316 | -0.292 | -0.0746 | No |
| 42 | DCLK1 | na | 18498 | -0.303 | -0.0577 | No |
| 43 | EFCAB14 | na | 18552 | -0.308 | -0.0346 | No |
| 44 | SERPING1 | na | 18589 | -0.311 | -0.0105 | No |
| 45 | ADIPOR2 | na | 18835 | -0.333 | 0.0059 | No |
| 46 | RASA3 | na | 19068 | -0.356 | 0.0248 | No |
| 47 | MSN | na | 19345 | -0.381 | 0.0438 | No |
| 48 | RNF213 | na | 21724 | -0.869 | 0.0069 | No |
Table: GSEA details [plain text format]

  

Fig 2: DEMAGALHAES\_AGING\_UP      
 Blue-Pink O' Gram in the Space of the Analyzed GeneSet

  

Fig 3: DEMAGALHAES\_AGING\_UP: Random ES distribution      
 Gene set null distribution of ES for **DEMAGALHAES\_AGING\_UP**

  
