# Supplementary material for: Regulation of microglia related neuroinflammation contributes to the protective effect of Gelsevirine on ischemic stroke
Source: Front Immunol. 2023 Mar 30;14:1164278. doi: 10.3389/fimmu.2023.1164278 (PMC10098192; doi:10.3389/fimmu.2023.1164278)
Supplement: Supplementary file 6 [file DataSheet_6.zip › fig 5 raw/fig 5-G raw/inflammation.Gsea.1649955013530/EINAV_INTERFERON_SIGNATURE_IN_CANCER.html]

Details for gene set EINAV\_INTERFERON\_SIGNATURE\_IN\_CANCER[GSEA]

|  || Dataset | OGD\_DRUG\_DRUG.OGD\_FRUG.cls#Gs\_versus\_MCAO.OGD\_FRUG.cls#Gs\_versus\_MCAO\_repos |
| Phenotype | OGD\_FRUG.cls#Gs\_versus\_MCAO\_repos |
| Upregulated in class | MCAO |
| GeneSet | EINAV\_INTERFERON\_SIGNATURE\_IN\_CANCER |
| Enrichment Score (ES) | -0.31487617 |
| Normalized Enrichment Score (NES) | -0.7335486 |
| Nominal p-value | 0.85760516 |
| FDR q-value | 0.9206459 |
| FWER p-Value | 1.0 |
Table: GSEA Results Summary

  

Fig 1: Enrichment plot: EINAV\_INTERFERON\_SIGNATURE\_IN\_CANCER      
 Profile of the Running ES Score & Positions of GeneSet Members on the Rank Ordered List

  

| SYMBOL | TITLE | RANK IN GENE LIST | RANK METRIC SCORE | RUNNING ES | CORE ENRICHMENT || 1 | SP110 | na | 2191 | 0.317 | -0.0420 | No |
| 2 | IFI30 | na | 2266 | 0.308 | 0.0114 | No |
| 3 | LY6E | na | 2357 | 0.299 | 0.0623 | No |
| 4 | BST2 | na | 2583 | 0.280 | 0.1035 | No |
| 5 | ISG15 | na | 2652 | 0.274 | 0.1509 | No |
| 6 | UBE2L6 | na | 2844 | 0.255 | 0.1892 | No |
| 7 | IFI35 | na | 2904 | 0.250 | 0.2326 | No |
| 8 | IFIT3 | na | 2985 | 0.243 | 0.2737 | No |
| 9 | SP100 | na | 4402 | 0.136 | 0.2340 | No |
| 10 | IFIT1 | na | 5119 | 0.089 | 0.2176 | No |
| 11 | IFI44 | na | 6697 | 0.013 | 0.1479 | No |
| 12 | IFI44L | na | 8987 | 0.000 | 0.0432 | No |
| 13 | TGIF1 | na | 13271 | -0.001 | -0.1527 | No |
| 14 | ERG | na | 13450 | -0.006 | -0.1597 | No |
| 15 | EIF2AK2 | na | 16842 | -0.185 | -0.2808 | Yes |
| 16 | IRF9 | na | 17430 | -0.226 | -0.2661 | Yes |
| 17 | STAT1 | na | 17788 | -0.252 | -0.2361 | Yes |
| 18 | BRD3 | na | 18083 | -0.275 | -0.1989 | Yes |
| 19 | MORC3 | na | 19092 | -0.359 | -0.1789 | Yes |
| 20 | RXRA | na | 19209 | -0.370 | -0.1160 | Yes |
| 21 | ADAR | na | 19317 | -0.379 | -0.0511 | Yes |
| 22 | OAS2 | na | 20043 | -0.449 | -0.0015 | Yes |
| 23 | CMTR1 | na | 20164 | -0.463 | 0.0783 | Yes |
Table: GSEA details [plain text format]

  

Fig 2: EINAV\_INTERFERON\_SIGNATURE\_IN\_CANCER      
 Blue-Pink O' Gram in the Space of the Analyzed GeneSet

  

Fig 3: EINAV\_INTERFERON\_SIGNATURE\_IN\_CANCER: Random ES distribution      
 Gene set null distribution of ES for **EINAV\_INTERFERON\_SIGNATURE\_IN\_CANCER**

  
