# Supplementary material for: Regulation of microglia related neuroinflammation contributes to the protective effect of Gelsevirine on ischemic stroke
Source: Front Immunol. 2023 Mar 30;14:1164278. doi: 10.3389/fimmu.2023.1164278 (PMC10098192; doi:10.3389/fimmu.2023.1164278)
Supplement: Supplementary file 6 [file DataSheet_6.zip › fig 5 raw/fig 5-G raw/inflammation.Gsea.1649955013530/GOBP_ACUTE_INFLAMMATORY_RESPONSE.html]

Details for gene set GOBP\_ACUTE\_INFLAMMATORY\_RESPONSE[GSEA]

|  || Dataset | OGD\_DRUG\_DRUG.OGD\_FRUG.cls#Gs\_versus\_MCAO.OGD\_FRUG.cls#Gs\_versus\_MCAO\_repos |
| Phenotype | OGD\_FRUG.cls#Gs\_versus\_MCAO\_repos |
| Upregulated in class | MCAO |
| GeneSet | GOBP\_ACUTE\_INFLAMMATORY\_RESPONSE |
| Enrichment Score (ES) | -0.28805578 |
| Normalized Enrichment Score (NES) | -0.86056834 |
| Nominal p-value | 0.8002755 |
| FDR q-value | 1.0 |
| FWER p-Value | 1.0 |
Table: GSEA Results Summary

  

Fig 1: Enrichment plot: GOBP\_ACUTE\_INFLAMMATORY\_RESPONSE      
 Profile of the Running ES Score & Positions of GeneSet Members on the Rank Ordered List

  

| SYMBOL | TITLE | RANK IN GENE LIST | RANK METRIC SCORE | RUNNING ES | CORE ENRICHMENT || 1 | S100A8 | na | 94 | 1.012 | 0.0468 | No |
| 2 | NPFF | na | 150 | 0.853 | 0.0873 | No |
| 3 | ADAM8 | na | 348 | 0.658 | 0.1115 | No |
| 4 | DNASE1L3 | na | 382 | 0.641 | 0.1423 | No |
| 5 | SERPINF2 | na | 905 | 0.478 | 0.1425 | No |
| 6 | IL20RB | na | 980 | 0.467 | 0.1627 | No |
| 7 | NUPR1 | na | 1089 | 0.451 | 0.1805 | No |
| 8 | PIK3CG | na | 1248 | 0.428 | 0.1948 | No |
| 9 | PLA2G2D | na | 1339 | 0.416 | 0.2117 | No |
| 10 | ITIH4 | na | 1680 | 0.377 | 0.2151 | No |
| 11 | SIGIRR | na | 1691 | 0.375 | 0.2336 | No |
| 12 | GSTP1 | na | 1927 | 0.346 | 0.2403 | No |
| 13 | PARK7 | na | 2064 | 0.331 | 0.2508 | No |
| 14 | CTNNBIP1 | na | 2373 | 0.297 | 0.2516 | No |
| 15 | HAMP | na | 2926 | 0.248 | 0.2388 | No |
| 16 | C3 | na | 3048 | 0.237 | 0.2452 | No |
| 17 | NPY5R | na | 3542 | 0.197 | 0.2325 | No |
| 18 | PLSCR1 | na | 3598 | 0.192 | 0.2397 | No |
| 19 | F3 | na | 3714 | 0.185 | 0.2437 | No |
| 20 | CEBPB | na | 4280 | 0.144 | 0.2251 | No |
| 21 | CCR7 | na | 4335 | 0.141 | 0.2297 | No |
| 22 | VNN1 | na | 4394 | 0.137 | 0.2339 | No |
| 23 | TNFSF4 | na | 4989 | 0.096 | 0.2115 | No |
| 24 | FUT7 | na | 5016 | 0.095 | 0.2151 | No |
| 25 | F8 | na | 5086 | 0.091 | 0.2166 | No |
| 26 | ASS1 | na | 5413 | 0.073 | 0.2053 | No |
| 27 | ZP3 | na | 5603 | 0.063 | 0.1998 | No |
| 28 | C2CD4A | na | 5657 | 0.060 | 0.2004 | No |
| 29 | AHSG | na | 5733 | 0.056 | 0.1997 | No |
| 30 | TNF | na | 5838 | 0.050 | 0.1975 | No |
| 31 | OSM | na | 5924 | 0.045 | 0.1959 | No |
| 32 | PTGES | na | 6266 | 0.030 | 0.1817 | No |
| 33 | TNFRSF11A | na | 6559 | 0.018 | 0.1692 | No |
| 34 | MBL2 | na | 7182 | 0.000 | 0.1407 | No |
| 35 | CD6 | na | 7313 | 0.000 | 0.1347 | No |
| 36 | CRP | na | 7442 | 0.000 | 0.1288 | No |
| 37 | EPO | na | 7843 | 0.000 | 0.1105 | No |
| 38 | TREM1 | na | 7894 | 0.000 | 0.1082 | No |
| 39 | IL4 | na | 8412 | 0.000 | 0.0844 | No |
| 40 | CNR1 | na | 8681 | 0.000 | 0.0721 | No |
| 41 | ORM1 | na | 8759 | 0.000 | 0.0686 | No |
| 42 | TAC1 | na | 8994 | 0.000 | 0.0579 | No |
| 43 | MRGPRX1 | na | 9149 | 0.000 | 0.0508 | No |
| 44 | IL31RA | na | 9167 | 0.000 | 0.0500 | No |
| 45 | C2CD4B | na | 9395 | 0.000 | 0.0396 | No |
| 46 | SAA4 | na | 9425 | 0.000 | 0.0382 | No |
| 47 | SAA2 | na | 9431 | 0.000 | 0.0380 | No |
| 48 | FFAR3 | na | 9795 | 0.000 | 0.0213 | No |
| 49 | TFR2 | na | 9849 | 0.000 | 0.0189 | No |
| 50 | APCS | na | 9888 | 0.000 | 0.0172 | No |
| 51 | TNFSF11 | na | 10006 | 0.000 | 0.0118 | No |
| 52 | ADCYAP1 | na | 10471 | 0.000 | -0.0095 | No |
| 53 | CD163 | na | 10688 | 0.000 | -0.0194 | No |
| 54 | IL1A | na | 10924 | 0.000 | -0.0302 | No |
| 55 | ELANE | na | 11444 | 0.000 | -0.0540 | No |
| 56 | OPRM1 | na | 11566 | 0.000 | -0.0596 | No |
| 57 | REG3A | na | 12387 | 0.000 | -0.0973 | No |
| 58 | REG3G | na | 12392 | 0.000 | -0.0974 | No |
| 59 | CREB3L3 | na | 13285 | -0.001 | -0.1383 | No |
| 60 | HP | na | 13303 | -0.002 | -0.1390 | No |
| 61 | BTK | na | 13329 | -0.002 | -0.1400 | No |
| 62 | SAA1 | na | 13487 | -0.007 | -0.1469 | No |
| 63 | ADORA1 | na | 13668 | -0.011 | -0.1546 | No |
| 64 | EDNRB | na | 14010 | -0.021 | -0.1692 | No |
| 65 | FFAR2 | na | 14204 | -0.028 | -0.1767 | No |
| 66 | IL1B | na | 14311 | -0.032 | -0.1799 | No |
| 67 | F2 | na | 14453 | -0.038 | -0.1844 | No |
| 68 | PTGER3 | na | 14495 | -0.040 | -0.1843 | No |
| 69 | FCGR2B | na | 14786 | -0.055 | -0.1948 | No |
| 70 | MYLK3 | na | 14999 | -0.068 | -0.2011 | No |
| 71 | GATA3 | na | 15063 | -0.072 | -0.2004 | No |
| 72 | F12 | na | 15322 | -0.087 | -0.2078 | No |
| 73 | OGG1 | na | 15414 | -0.093 | -0.2073 | No |
| 74 | B4GALT1 | na | 15457 | -0.095 | -0.2044 | No |
| 75 | SERPINC1 | na | 15502 | -0.098 | -0.2015 | No |
| 76 | ORM2 | na | 15574 | -0.102 | -0.1996 | No |
| 77 | IL22 | na | 15910 | -0.122 | -0.2088 | No |
| 78 | EIF2AK1 | na | 16142 | -0.137 | -0.2125 | No |
| 79 | RHBDD3 | na | 16184 | -0.140 | -0.2073 | No |
| 80 | EXT1 | na | 16426 | -0.156 | -0.2105 | No |
| 81 | PTGS2 | na | 16449 | -0.158 | -0.2036 | No |
| 82 | PRCP | na | 16606 | -0.169 | -0.2022 | No |
| 83 | VCAM1 | na | 17450 | -0.226 | -0.2295 | No |
| 84 | ACVR1 | na | 18035 | -0.272 | -0.2426 | No |
| 85 | OSMR | na | 18234 | -0.287 | -0.2372 | No |
| 86 | ANO6 | na | 18447 | -0.300 | -0.2318 | No |
| 87 | NLRP3 | na | 18740 | -0.325 | -0.2288 | No |
| 88 | IL6 | na | 19212 | -0.371 | -0.2317 | No |
| 89 | HFE | na | 19540 | -0.401 | -0.2265 | No |
| 90 | IL6ST | na | 20881 | -0.558 | -0.2599 | Yes |
| 91 | DNASE1 | na | 20954 | -0.570 | -0.2344 | Yes |
| 92 | ASH1L | na | 21240 | -0.629 | -0.2158 | Yes |
| 93 | ICAM1 | na | 21398 | -0.667 | -0.1893 | Yes |
| 94 | FN1 | na | 21407 | -0.672 | -0.1558 | Yes |
| 95 | LBP | na | 21555 | -0.728 | -0.1257 | Yes |
| 96 | PPARG | na | 21669 | -0.808 | -0.0901 | Yes |
| 97 | UGT1A1 | na | 21720 | -0.867 | -0.0487 | Yes |
| 98 | KLKB1 | na | 21813 | -1.104 | 0.0028 | Yes |
Table: GSEA details [plain text format]

  

Fig 2: GOBP\_ACUTE\_INFLAMMATORY\_RESPONSE      
 Blue-Pink O' Gram in the Space of the Analyzed GeneSet

  

Fig 3: GOBP\_ACUTE\_INFLAMMATORY\_RESPONSE: Random ES distribution      
 Gene set null distribution of ES for **GOBP\_ACUTE\_INFLAMMATORY\_RESPONSE**

  
