# Supplementary material for: Regulation of microglia related neuroinflammation contributes to the protective effect of Gelsevirine on ischemic stroke
Source: Front Immunol. 2023 Mar 30;14:1164278. doi: 10.3389/fimmu.2023.1164278 (PMC10098192; doi:10.3389/fimmu.2023.1164278)
Supplement: Supplementary file 6 [file DataSheet_6.zip › fig 5 raw/fig 5-G raw/inflammation.Gsea.1649955013530/GOBP_CHRONIC_INFLAMMATORY_RESPONSE.html]

Details for gene set GOBP\_CHRONIC\_INFLAMMATORY\_RESPONSE[GSEA]

|  || Dataset | OGD\_DRUG\_DRUG.OGD\_FRUG.cls#Gs\_versus\_MCAO.OGD\_FRUG.cls#Gs\_versus\_MCAO\_repos |
| Phenotype | OGD\_FRUG.cls#Gs\_versus\_MCAO\_repos |
| Upregulated in class | Gs |
| GeneSet | GOBP\_CHRONIC\_INFLAMMATORY\_RESPONSE |
| Enrichment Score (ES) | 0.4154289 |
| Normalized Enrichment Score (NES) | 0.99037576 |
| Nominal p-value | 0.42937854 |
| FDR q-value | 0.7507854 |
| FWER p-Value | 0.899 |
Table: GSEA Results Summary

  

Fig 1: Enrichment plot: GOBP\_CHRONIC\_INFLAMMATORY\_RESPONSE      
 Profile of the Running ES Score & Positions of GeneSet Members on the Rank Ordered List

  

| SYMBOL | TITLE | RANK IN GENE LIST | RANK METRIC SCORE | RUNNING ES | CORE ENRICHMENT || 1 | S100A8 | na | 94 | 1.012 | 0.1937 | Yes |
| 2 | CCL11 | na | 386 | 0.637 | 0.3050 | Yes |
| 3 | CCL5 | na | 495 | 0.590 | 0.4154 | Yes |
| 4 | UNC13D | na | 3407 | 0.206 | 0.3226 | No |
| 5 | VNN1 | na | 4394 | 0.137 | 0.3043 | No |
| 6 | S100A9 | na | 4438 | 0.133 | 0.3284 | No |
| 7 | TNF | na | 5838 | 0.050 | 0.2742 | No |
| 8 | PTGES | na | 6266 | 0.030 | 0.2604 | No |
| 9 | IL4 | na | 8412 | 0.000 | 0.1623 | No |
| 10 | CXCL13 | na | 8870 | 0.000 | 0.1414 | No |
| 11 | IDO1 | na | 9471 | 0.000 | 0.1140 | No |
| 12 | LTA | na | 13822 | -0.015 | -0.0821 | No |
| 13 | IL10 | na | 14957 | -0.065 | -0.1212 | No |
| 14 | AHCY | na | 17095 | -0.202 | -0.1795 | No |
| 15 | VCAM1 | na | 17450 | -0.226 | -0.1514 | No |
| 16 | GJA1 | na | 17476 | -0.228 | -0.1079 | No |
| 17 | FOXP3 | na | 19112 | -0.361 | -0.1120 | No |
| 18 | TNFAIP3 | na | 19665 | -0.413 | -0.0565 | No |
| 19 | THBS1 | na | 21664 | -0.806 | 0.0097 | No |
Table: GSEA details [plain text format]

  

Fig 2: GOBP\_CHRONIC\_INFLAMMATORY\_RESPONSE      
 Blue-Pink O' Gram in the Space of the Analyzed GeneSet

  

Fig 3: GOBP\_CHRONIC\_INFLAMMATORY\_RESPONSE: Random ES distribution      
 Gene set null distribution of ES for **GOBP\_CHRONIC\_INFLAMMATORY\_RESPONSE**

  
