# Supplementary material for: Regulation of microglia related neuroinflammation contributes to the protective effect of Gelsevirine on ischemic stroke
Source: Front Immunol. 2023 Mar 30;14:1164278. doi: 10.3389/fimmu.2023.1164278 (PMC10098192; doi:10.3389/fimmu.2023.1164278)
Supplement: Supplementary file 6 [file DataSheet_6.zip › fig 5 raw/fig 5-G raw/inflammation.Gsea.1649955013530/GOBP_GRANULOCYTE_DIFFERENTIATION.html]

Details for gene set GOBP\_GRANULOCYTE\_DIFFERENTIATION[GSEA]

|  || Dataset | OGD\_DRUG\_DRUG.OGD\_FRUG.cls#Gs\_versus\_MCAO.OGD\_FRUG.cls#Gs\_versus\_MCAO\_repos |
| Phenotype | OGD\_FRUG.cls#Gs\_versus\_MCAO\_repos |
| Upregulated in class | MCAO |
| GeneSet | GOBP\_GRANULOCYTE\_DIFFERENTIATION |
| Enrichment Score (ES) | -0.3436539 |
| Normalized Enrichment Score (NES) | -0.85494494 |
| Nominal p-value | 0.73125 |
| FDR q-value | 0.95571864 |
| FWER p-Value | 1.0 |
Table: GSEA Results Summary

  

Fig 1: Enrichment plot: GOBP\_GRANULOCYTE\_DIFFERENTIATION      
 Profile of the Running ES Score & Positions of GeneSet Members on the Rank Ordered List

  

| SYMBOL | TITLE | RANK IN GENE LIST | RANK METRIC SCORE | RUNNING ES | CORE ENRICHMENT || 1 | CSF3 | na | 128 | 0.917 | 0.1006 | No |
| 2 | CBFA2T3 | na | 184 | 0.817 | 0.1929 | No |
| 3 | CSF2 | na | 1966 | 0.342 | 0.1511 | No |
| 4 | HAX1 | na | 2076 | 0.330 | 0.1844 | No |
| 5 | CEBPE | na | 2194 | 0.316 | 0.2158 | No |
| 6 | JAGN1 | na | 2802 | 0.259 | 0.2180 | No |
| 7 | HCLS1 | na | 3920 | 0.171 | 0.1868 | No |
| 8 | IL25 | na | 5553 | 0.065 | 0.1197 | No |
| 9 | ADIPOQ | na | 5554 | 0.065 | 0.1273 | No |
| 10 | CEBPA | na | 7443 | 0.000 | 0.0408 | No |
| 11 | EVI2B | na | 7468 | 0.000 | 0.0397 | No |
| 12 | C1QC | na | 9432 | 0.000 | -0.0501 | No |
| 13 | TESC | na | 9684 | 0.000 | -0.0616 | No |
| 14 | GATA1 | na | 10811 | 0.000 | -0.1132 | No |
| 15 | CEACAM1 | na | 14089 | -0.023 | -0.2605 | No |
| 16 | TAL1 | na | 15907 | -0.122 | -0.3295 | Yes |
| 17 | GATA2 | na | 16191 | -0.140 | -0.3262 | Yes |
| 18 | IL5 | na | 16224 | -0.142 | -0.3112 | Yes |
| 19 | SPI1 | na | 16429 | -0.156 | -0.3024 | Yes |
| 20 | RARA | na | 16711 | -0.178 | -0.2947 | Yes |
| 21 | LEF1 | na | 16773 | -0.181 | -0.2765 | Yes |
| 22 | RUNX1 | na | 16837 | -0.185 | -0.2580 | Yes |
| 23 | ZBTB46 | na | 17203 | -0.209 | -0.2504 | Yes |
| 24 | TRIB1 | na | 17469 | -0.228 | -0.2361 | Yes |
| 25 | AP3B1 | na | 18084 | -0.275 | -0.2322 | Yes |
| 26 | CUL4A | na | 18105 | -0.277 | -0.2010 | Yes |
| 27 | SP3 | na | 19231 | -0.372 | -0.2094 | Yes |
| 28 | LBR | na | 19802 | -0.426 | -0.1861 | Yes |
| 29 | ZFPM1 | na | 20299 | -0.477 | -0.1534 | Yes |
| 30 | L3MBTL3 | na | 20678 | -0.526 | -0.1097 | Yes |
| 31 | FASN | na | 21113 | -0.600 | -0.0599 | Yes |
| 32 | INPP5D | na | 21674 | -0.816 | 0.0092 | Yes |
Table: GSEA details [plain text format]

  

Fig 2: GOBP\_GRANULOCYTE\_DIFFERENTIATION      
 Blue-Pink O' Gram in the Space of the Analyzed GeneSet

  

Fig 3: GOBP\_GRANULOCYTE\_DIFFERENTIATION: Random ES distribution      
 Gene set null distribution of ES for **GOBP\_GRANULOCYTE\_DIFFERENTIATION**

  
