# Supplementary material for: Regulation of microglia related neuroinflammation contributes to the protective effect of Gelsevirine on ischemic stroke
Source: Front Immunol. 2023 Mar 30;14:1164278. doi: 10.3389/fimmu.2023.1164278 (PMC10098192; doi:10.3389/fimmu.2023.1164278)
Supplement: Supplementary file 6 [file DataSheet_6.zip › fig 5 raw/fig 5-G raw/inflammation.Gsea.1649955013530/GOBP_T_HELPER_17_TYPE_IMMUNE_RESPONSE.html]

Details for gene set GOBP\_T\_HELPER\_17\_TYPE\_IMMUNE\_RESPONSE[GSEA]

|  || Dataset | OGD\_DRUG\_DRUG.OGD\_FRUG.cls#Gs\_versus\_MCAO.OGD\_FRUG.cls#Gs\_versus\_MCAO\_repos |
| Phenotype | OGD\_FRUG.cls#Gs\_versus\_MCAO\_repos |
| Upregulated in class | MCAO |
| GeneSet | GOBP\_T\_HELPER\_17\_TYPE\_IMMUNE\_RESPONSE |
| Enrichment Score (ES) | -0.3282042 |
| Normalized Enrichment Score (NES) | -0.8169963 |
| Nominal p-value | 0.7848101 |
| FDR q-value | 0.91521156 |
| FWER p-Value | 1.0 |
Table: GSEA Results Summary

  

Fig 1: Enrichment plot: GOBP\_T\_HELPER\_17\_TYPE\_IMMUNE\_RESPONSE      
 Profile of the Running ES Score & Positions of GeneSet Members on the Rank Ordered List

  

| SYMBOL | TITLE | RANK IN GENE LIST | RANK METRIC SCORE | RUNNING ES | CORE ENRICHMENT || 1 | RORA | na | 154 | 0.852 | 0.1195 | No |
| 2 | TNFSF18 | na | 1571 | 0.391 | 0.1128 | No |
| 3 | TBX21 | na | 1623 | 0.384 | 0.1674 | No |
| 4 | PHB | na | 2060 | 0.331 | 0.1967 | No |
| 5 | IL27RA | na | 2167 | 0.320 | 0.2394 | No |
| 6 | IL12B | na | 3745 | 0.183 | 0.1944 | No |
| 7 | IL2 | na | 5219 | 0.084 | 0.1393 | No |
| 8 | LY9 | na | 6129 | 0.035 | 0.1030 | No |
| 9 | IL23R | na | 7469 | 0.000 | 0.0417 | No |
| 10 | NFKBID | na | 7976 | 0.000 | 0.0185 | No |
| 11 | IL4 | na | 8412 | 0.000 | -0.0014 | No |
| 12 | PRKCQ | na | 10157 | 0.000 | -0.0812 | No |
| 13 | IRF4 | na | 12103 | 0.000 | -0.1703 | No |
| 14 | SLAMF6 | na | 12873 | 0.000 | -0.2055 | No |
| 15 | BATF | na | 13217 | 0.000 | -0.2212 | No |
| 16 | NLRP10 | na | 13484 | -0.007 | -0.2324 | No |
| 17 | IL12RB1 | na | 14229 | -0.029 | -0.2621 | No |
| 18 | ZC3H12A | na | 14890 | -0.062 | -0.2832 | No |
| 19 | ENTPD7 | na | 15874 | -0.120 | -0.3104 | Yes |
| 20 | ZBTB7B | na | 16157 | -0.138 | -0.3028 | Yes |
| 21 | NFKBIZ | na | 16477 | -0.160 | -0.2936 | Yes |
| 22 | SMAD7 | na | 16595 | -0.169 | -0.2739 | Yes |
| 23 | LOXL3 | na | 17279 | -0.215 | -0.2733 | Yes |
| 24 | IL23A | na | 17897 | -0.261 | -0.2628 | Yes |
| 25 | MALT1 | na | 18439 | -0.299 | -0.2431 | Yes |
| 26 | FOXP3 | na | 19112 | -0.361 | -0.2202 | Yes |
| 27 | IL6 | na | 19212 | -0.371 | -0.1697 | Yes |
| 28 | RORC | na | 19978 | -0.442 | -0.1390 | Yes |
| 29 | RC3H1 | na | 20175 | -0.464 | -0.0790 | Yes |
| 30 | RC3H2 | na | 20676 | -0.526 | -0.0238 | Yes |
| 31 | STAT3 | na | 20708 | -0.529 | 0.0534 | Yes |
Table: GSEA details [plain text format]

  

Fig 2: GOBP\_T\_HELPER\_17\_TYPE\_IMMUNE\_RESPONSE      
 Blue-Pink O' Gram in the Space of the Analyzed GeneSet

  

Fig 3: GOBP\_T\_HELPER\_17\_TYPE\_IMMUNE\_RESPONSE: Random ES distribution      
 Gene set null distribution of ES for **GOBP\_T\_HELPER\_17\_TYPE\_IMMUNE\_RESPONSE**

  
