# Supplementary material for: Regulation of microglia related neuroinflammation contributes to the protective effect of Gelsevirine on ischemic stroke
Source: Front Immunol. 2023 Mar 30;14:1164278. doi: 10.3389/fimmu.2023.1164278 (PMC10098192; doi:10.3389/fimmu.2023.1164278)
Supplement: Supplementary file 6 [file DataSheet_6.zip › fig 5 raw/fig 5-G raw/inflammation.Gsea.1649955013530/gsea_report_for_Gs_1649955013530.html]

Report for Gs 1649955013530 [GSEA]

| GS  follow link to MSigDB | GS DETAILS | SIZE | ES | NES | NOM p-val | FDR q-val | FWER p-val | RANK AT MAX | LEADING EDGE || 1 | ALTEMEIER\_RESPONSE\_TO\_LPS\_WITH\_MECHANICAL\_VENTILATION | Details ... | 107 | 0.33 | 1.08 | 0.223 | 1.000 | 0.758 | 4378 | tags=29%, list=20%, signal=36% |
| 2 | DEMAGALHAES\_AGING\_UP | Details ... | 48 | 0.36 | 1.05 | 0.351 | 0.861 | 0.818 | 3259 | tags=33%, list=15%, signal=39% |
| 3 | GOBP\_CHRONIC\_INFLAMMATORY\_RESPONSE | Details ... | 19 | 0.42 | 0.99 | 0.429 | 0.751 | 0.899 | 495 | tags=16%, list=2%, signal=16% |
| 4 | BIOCARTA\_INFLAM\_PATHWAY | Details ... | 23 | 0.36 | 0.91 | 0.583 | 0.791 | 0.961 | 3251 | tags=22%, list=15%, signal=26% |
| 5 | BIOCARTA\_LAIR\_PATHWAY | Details ... | 15 | 0.35 | 0.80 | 0.733 | 0.840 | 0.990 | 3048 | tags=20%, list=14%, signal=23% |
Table: Gene sets enriched in phenotype **Gs (3 samples)**[plain text format]****

  
