# Supplementary material for: Regulation of microglia related neuroinflammation contributes to the protective effect of Gelsevirine on ischemic stroke
Source: Front Immunol. 2023 Mar 30;14:1164278. doi: 10.3389/fimmu.2023.1164278 (PMC10098192; doi:10.3389/fimmu.2023.1164278)
Supplement: Supplementary file 6 [file DataSheet_6.zip › fig 5 raw/fig 5-G raw/inflammation.Gsea.1649955013530/gsea_report_for_MCAO_1649955013530.html]

Report for MCAO 1649955013530 [GSEA]

| GS  follow link to MSigDB | GS DETAILS | SIZE | ES | NES | NOM p-val | FDR q-val | FWER p-val | RANK AT MAX | LEADING EDGE || 1 | BLALOCK\_ALZHEIMERS\_DISEASE\_INCIPIENT\_UP | Details ... | 334 | -0.50 | -1.68 | 0.000 | 0.011 | 0.011 | 5693 | tags=50%, list=26%, signal=67% |
| 2 | BIOCARTA\_PAR1\_PATHWAY | Details ... | 19 | -0.61 | -1.36 | 0.089 | 0.263 | 0.431 | 3938 | tags=63%, list=18%, signal=77% |
| 3 | BIOCARTA\_IL6\_PATHWAY | Details ... | 20 | -0.59 | -1.34 | 0.088 | 0.206 | 0.480 | 2768 | tags=45%, list=13%, signal=51% |
| 4 | BIOCARTA\_CCR3\_PATHWAY | Details ... | 19 | -0.45 | -1.01 | 0.440 | 0.997 | 0.992 | 3656 | tags=32%, list=17%, signal=38% |
| 5 | BLALOCK\_ALZHEIMERS\_DISEASE\_INCIPIENT\_DN | Details ... | 153 | -0.29 | -0.90 | 0.717 | 1.000 | 1.000 | 4206 | tags=28%, list=19%, signal=35% |
| 6 | GOBP\_ACUTE\_INFLAMMATORY\_RESPONSE | Details ... | 98 | -0.29 | -0.86 | 0.800 | 1.000 | 1.000 | 996 | tags=9%, list=5%, signal=10% |
| 7 | GOBP\_GRANULOCYTE\_DIFFERENTIATION | Details ... | 32 | -0.34 | -0.85 | 0.731 | 0.956 | 1.000 | 5970 | tags=53%, list=27%, signal=73% |
| 8 | GOBP\_T\_HELPER\_17\_TYPE\_IMMUNE\_RESPONSE | Details ... | 31 | -0.33 | -0.82 | 0.785 | 0.915 | 1.000 | 6003 | tags=42%, list=27%, signal=58% |
| 9 | EINAV\_INTERFERON\_SIGNATURE\_IN\_CANCER | Details ... | 23 | -0.31 | -0.73 | 0.858 | 0.921 | 1.000 | 5035 | tags=39%, list=23%, signal=51% |
Table: Gene sets enriched in phenotype **MCAO (3 samples)**[plain text format]****

  
