# Supplementary material for: Regulation of microglia related neuroinflammation contributes to the protective effect of Gelsevirine on ischemic stroke
Source: Front Immunol. 2023 Mar 30;14:1164278. doi: 10.3389/fimmu.2023.1164278 (PMC10098192; doi:10.3389/fimmu.2023.1164278)
Supplement: Supplementary file 6 [file DataSheet_6.zip › fig 5 raw/fig 5-G raw/inflammation.Gsea.1649955060129/AZARE_NEOPLASTIC_TRANSFORMATION_BY_STAT3_DN.html]

Details for gene set AZARE\_NEOPLASTIC\_TRANSFORMATION\_BY\_STAT3\_DN[GSEA]

|  || Dataset | OGD\_DRUG\_DRUG.OGD\_FRUG.cls#Gs\_versus\_MCAO.OGD\_FRUG.cls#Gs\_versus\_MCAO\_repos |
| Phenotype | OGD\_FRUG.cls#Gs\_versus\_MCAO\_repos |
| Upregulated in class | Gs |
| GeneSet | AZARE\_NEOPLASTIC\_TRANSFORMATION\_BY\_STAT3\_DN |
| Enrichment Score (ES) | 0.4781993 |
| Normalized Enrichment Score (NES) | 1.0814818 |
| Nominal p-value | 0.34210527 |
| FDR q-value | 0.6348132 |
| FWER p-Value | 1.0 |
Table: GSEA Results Summary

  

Fig 1: Enrichment plot: AZARE\_NEOPLASTIC\_TRANSFORMATION\_BY\_STAT3\_DN      
 Profile of the Running ES Score & Positions of GeneSet Members on the Rank Ordered List

  

| SYMBOL | TITLE | RANK IN GENE LIST | RANK METRIC SCORE | RUNNING ES | CORE ENRICHMENT || 1 | AQP3 | na | 175 | 0.828 | 0.1439 | Yes |
| 2 | CDA | na | 393 | 0.633 | 0.2503 | Yes |
| 3 | PPL | na | 571 | 0.564 | 0.3458 | Yes |
| 4 | PLAC8 | na | 951 | 0.471 | 0.4148 | Yes |
| 5 | HR | na | 1272 | 0.425 | 0.4782 | Yes |
| 6 | AIM1L | na | 4050 | 0.159 | 0.3804 | No |
| 7 | CPE | na | 4096 | 0.156 | 0.4070 | No |
| 8 | PTGES | na | 6266 | 0.030 | 0.3133 | No |
| 9 | PEG10 | na | 10005 | 0.000 | 0.1423 | No |
| 10 | KRT13 | na | 12242 | 0.000 | 0.0400 | No |
| 11 | CCL20 | na | 16412 | -0.155 | -0.1222 | No |
| 12 | PTGS1 | na | 16639 | -0.172 | -0.1009 | No |
| 13 | TFPI | na | 17150 | -0.206 | -0.0865 | No |
| 14 | FOXG1 | na | 17442 | -0.226 | -0.0584 | No |
| 15 | FARP1 | na | 21381 | -0.662 | -0.1169 | No |
| 16 | HSPG2 | na | 21606 | -0.760 | 0.0123 | No |
Table: GSEA details [plain text format]

  

Fig 2: AZARE\_NEOPLASTIC\_TRANSFORMATION\_BY\_STAT3\_DN      
 Blue-Pink O' Gram in the Space of the Analyzed GeneSet

  

Fig 3: AZARE\_NEOPLASTIC\_TRANSFORMATION\_BY\_STAT3\_DN: Random ES distribution      
 Gene set null distribution of ES for **AZARE\_NEOPLASTIC\_TRANSFORMATION\_BY\_STAT3\_DN**

  
