# Supplementary material for: Regulation of microglia related neuroinflammation contributes to the protective effect of Gelsevirine on ischemic stroke
Source: Front Immunol. 2023 Mar 30;14:1164278. doi: 10.3389/fimmu.2023.1164278 (PMC10098192; doi:10.3389/fimmu.2023.1164278)
Supplement: Supplementary file 6 [file DataSheet_6.zip › fig 5 raw/fig 5-G raw/inflammation.Gsea.1649955060129/BILD_CTNNB1_ONCOGENIC_SIGNATURE.html]

Details for gene set BILD\_CTNNB1\_ONCOGENIC\_SIGNATURE[GSEA]

|  || Dataset | OGD\_DRUG\_DRUG.OGD\_FRUG.cls#Gs\_versus\_MCAO.OGD\_FRUG.cls#Gs\_versus\_MCAO\_repos |
| Phenotype | OGD\_FRUG.cls#Gs\_versus\_MCAO\_repos |
| Upregulated in class | MCAO |
| GeneSet | BILD\_CTNNB1\_ONCOGENIC\_SIGNATURE |
| Enrichment Score (ES) | -0.5950741 |
| Normalized Enrichment Score (NES) | -1.7056209 |
| Nominal p-value | 0.0 |
| FDR q-value | 0.05703427 |
| FWER p-Value | 0.087 |
Table: GSEA Results Summary

  

Fig 1: Enrichment plot: BILD\_CTNNB1\_ONCOGENIC\_SIGNATURE      
 Profile of the Running ES Score & Positions of GeneSet Members on the Rank Ordered List

  

| SYMBOL | TITLE | RANK IN GENE LIST | RANK METRIC SCORE | RUNNING ES | CORE ENRICHMENT || 1 | CYP24A1 | na | 496 | 0.590 | 0.0027 | No |
| 2 | RPS11 | na | 542 | 0.572 | 0.0254 | No |
| 3 | RPS19 | na | 763 | 0.508 | 0.0372 | No |
| 4 | TAF1D | na | 826 | 0.493 | 0.0557 | No |
| 5 | RPL27A | na | 1302 | 0.421 | 0.0521 | No |
| 6 | MED31 | na | 1812 | 0.360 | 0.0443 | No |
| 7 | BAMBI | na | 2822 | 0.257 | 0.0091 | No |
| 8 | DDX52 | na | 4660 | 0.117 | -0.0701 | No |
| 9 | NEMF | na | 5419 | 0.073 | -0.1017 | No |
| 10 | CLN8 | na | 5624 | 0.062 | -0.1084 | No |
| 11 | SRSF6 | na | 6659 | 0.015 | -0.1552 | No |
| 12 | KAZN | na | 6937 | 0.004 | -0.1678 | No |
| 13 | RBM25 | na | 7001 | 0.001 | -0.1706 | No |
| 14 | AXIN2 | na | 8241 | 0.000 | -0.2274 | No |
| 15 | PTHLH | na | 8426 | 0.000 | -0.2359 | No |
| 16 | FOXQ1 | na | 8874 | 0.000 | -0.2564 | No |
| 17 | SCML1 | na | 9563 | 0.000 | -0.2879 | No |
| 18 | PLAGL1 | na | 9891 | 0.000 | -0.3029 | No |
| 19 | IL1A | na | 10924 | 0.000 | -0.3503 | No |
| 20 | TRIM14 | na | 11675 | 0.000 | -0.3847 | No |
| 21 | PHACTR2 | na | 13437 | -0.006 | -0.4652 | No |
| 22 | SRSF10 | na | 14060 | -0.022 | -0.4927 | No |
| 23 | COL13A1 | na | 14160 | -0.026 | -0.4961 | No |
| 24 | FERMT1 | na | 14345 | -0.033 | -0.5031 | No |
| 25 | NRD1 | na | 14575 | -0.044 | -0.5118 | No |
| 26 | DHX36 | na | 14647 | -0.048 | -0.5129 | No |
| 27 | YTHDC1 | na | 14941 | -0.065 | -0.5236 | No |
| 28 | CBX3 | na | 15297 | -0.086 | -0.5362 | No |
| 29 | SREK1 | na | 15872 | -0.120 | -0.5573 | No |
| 30 | GNG12 | na | 16015 | -0.130 | -0.5582 | No |
| 31 | SMC3 | na | 16488 | -0.161 | -0.5729 | No |
| 32 | EPRS | na | 16551 | -0.165 | -0.5687 | No |
| 33 | MAPKAP1 | na | 17128 | -0.204 | -0.5863 | Yes |
| 34 | PLEKHA1 | na | 17258 | -0.213 | -0.5830 | Yes |
| 35 | TMCC1 | na | 17390 | -0.223 | -0.5794 | Yes |
| 36 | SECISBP2 | na | 17524 | -0.232 | -0.5755 | Yes |
| 37 | ELF1 | na | 17655 | -0.241 | -0.5710 | Yes |
| 38 | TOP1 | na | 17830 | -0.256 | -0.5679 | Yes |
| 39 | FBXO11 | na | 17980 | -0.268 | -0.5632 | Yes |
| 40 | PNISR | na | 17992 | -0.269 | -0.5521 | Yes |
| 41 | ZMAT3 | na | 18162 | -0.282 | -0.5477 | Yes |
| 42 | DUSP5 | na | 18336 | -0.293 | -0.5429 | Yes |
| 43 | ARL5A | na | 18478 | -0.302 | -0.5364 | Yes |
| 44 | NR3C1 | na | 18932 | -0.342 | -0.5424 | Yes |
| 45 | ANKRD12 | na | 19131 | -0.363 | -0.5358 | Yes |
| 46 | RUNX2 | na | 19134 | -0.363 | -0.5202 | Yes |
| 47 | LARP4B | na | 19184 | -0.368 | -0.5065 | Yes |
| 48 | ABI2 | na | 19288 | -0.377 | -0.4950 | Yes |
| 49 | KDM4B | na | 19586 | -0.405 | -0.4911 | Yes |
| 50 | TNFAIP3 | na | 19665 | -0.413 | -0.4769 | Yes |
| 51 | KHNYN | na | 19729 | -0.419 | -0.4616 | Yes |
| 52 | FAM120A | na | 19787 | -0.424 | -0.4459 | Yes |
| 53 | SCAF11 | na | 19998 | -0.444 | -0.4364 | Yes |
| 54 | ARHGAP29 | na | 20007 | -0.445 | -0.4175 | Yes |
| 55 | LRBA | na | 20057 | -0.450 | -0.4003 | Yes |
| 56 | NCOA3 | na | 20068 | -0.451 | -0.3812 | Yes |
| 57 | FGD6 | na | 20330 | -0.481 | -0.3724 | Yes |
| 58 | THOC2 | na | 20513 | -0.503 | -0.3590 | Yes |
| 59 | NEK1 | na | 20520 | -0.504 | -0.3376 | Yes |
| 60 | COL8A2 | na | 20547 | -0.508 | -0.3168 | Yes |
| 61 | URB1 | na | 20558 | -0.510 | -0.2952 | Yes |
| 62 | PIK3C2A | na | 20684 | -0.527 | -0.2782 | Yes |
| 63 | LUZP1 | na | 20853 | -0.555 | -0.2619 | Yes |
| 64 | ATRX | na | 20975 | -0.573 | -0.2427 | Yes |
| 65 | BOD1L | na | 21039 | -0.586 | -0.2203 | Yes |
| 66 | SMG1 | na | 21090 | -0.597 | -0.1968 | Yes |
| 67 | LATS2 | na | 21104 | -0.599 | -0.1715 | Yes |
| 68 | ASH1L | na | 21240 | -0.629 | -0.1506 | Yes |
| 69 | CHD9 | na | 21256 | -0.632 | -0.1239 | Yes |
| 70 | VCAN | na | 21332 | -0.652 | -0.0992 | Yes |
| 71 | SORL1 | na | 21492 | -0.700 | -0.0762 | Yes |
| 72 | DYNC1H1 | na | 21518 | -0.713 | -0.0466 | Yes |
| 73 | FRYL | na | 21529 | -0.718 | -0.0161 | Yes |
| 74 | HIPK2 | na | 21575 | -0.739 | 0.0138 | Yes |
Table: GSEA details [plain text format]

  

Fig 2: BILD\_CTNNB1\_ONCOGENIC\_SIGNATURE      
 Blue-Pink O' Gram in the Space of the Analyzed GeneSet

  

Fig 3: BILD\_CTNNB1\_ONCOGENIC\_SIGNATURE: Random ES distribution      
 Gene set null distribution of ES for **BILD\_CTNNB1\_ONCOGENIC\_SIGNATURE**

  
