# Supplementary material for: Regulation of microglia related neuroinflammation contributes to the protective effect of Gelsevirine on ischemic stroke
Source: Front Immunol. 2023 Mar 30;14:1164278. doi: 10.3389/fimmu.2023.1164278 (PMC10098192; doi:10.3389/fimmu.2023.1164278)
Supplement: Supplementary file 6 [file DataSheet_6.zip › fig 5 raw/fig 5-G raw/inflammation.Gsea.1649955060129/BIOCARTA_EGF_PATHWAY.html]

Details for gene set BIOCARTA\_EGF\_PATHWAY[GSEA]

|  || Dataset | OGD\_DRUG\_DRUG.OGD\_FRUG.cls#Gs\_versus\_MCAO.OGD\_FRUG.cls#Gs\_versus\_MCAO\_repos |
| Phenotype | OGD\_FRUG.cls#Gs\_versus\_MCAO\_repos |
| Upregulated in class | MCAO |
| GeneSet | BIOCARTA\_EGF\_PATHWAY |
| Enrichment Score (ES) | -0.59922636 |
| Normalized Enrichment Score (NES) | -1.4894547 |
| Nominal p-value | 0.02685624 |
| FDR q-value | 0.09235908 |
| FWER p-Value | 0.759 |
Table: GSEA Results Summary

  

Fig 1: Enrichment plot: BIOCARTA\_EGF\_PATHWAY      
 Profile of the Running ES Score & Positions of GeneSet Members on the Rank Ordered List

  

| SYMBOL | TITLE | RANK IN GENE LIST | RANK METRIC SCORE | RUNNING ES | CORE ENRICHMENT || 1 | FOS | na | 1505 | 0.399 | -0.0265 | No |
| 2 | STAT4 | na | 2555 | 0.282 | -0.0446 | No |
| 3 | HRAS | na | 2716 | 0.268 | -0.0235 | No |
| 4 | MAPK3 | na | 4918 | 0.100 | -0.1137 | No |
| 5 | GRB2 | na | 5726 | 0.057 | -0.1446 | No |
| 6 | JUN | na | 5874 | 0.048 | -0.1463 | No |
| 7 | PRKCB | na | 13570 | -0.009 | -0.4975 | No |
| 8 | STAT2 | na | 14734 | -0.053 | -0.5452 | No |
| 9 | MAP2K1 | na | 15179 | -0.079 | -0.5571 | No |
| 10 | CSNK2A1 | na | 15329 | -0.088 | -0.5546 | No |
| 11 | SRF | na | 16304 | -0.148 | -0.5836 | Yes |
| 12 | STAT6 | na | 16496 | -0.161 | -0.5752 | Yes |
| 13 | MAP2K4 | na | 16904 | -0.189 | -0.5738 | Yes |
| 14 | RAF1 | na | 17251 | -0.212 | -0.5671 | Yes |
| 15 | STAT1 | na | 17788 | -0.252 | -0.5649 | Yes |
| 16 | MAPK8 | na | 17866 | -0.258 | -0.5411 | Yes |
| 17 | PRKCA | na | 18221 | -0.286 | -0.5269 | Yes |
| 18 | PIK3CA | na | 18513 | -0.305 | -0.5079 | Yes |
| 19 | RASA1 | na | 18622 | -0.313 | -0.4797 | Yes |
| 20 | EGFR | na | 19043 | -0.354 | -0.4614 | Yes |
| 21 | SHC1 | na | 19109 | -0.361 | -0.4261 | Yes |
| 22 | STAT5A | na | 19487 | -0.396 | -0.4014 | Yes |
| 23 | ELK1 | na | 19706 | -0.417 | -0.3671 | Yes |
| 24 | JAK1 | na | 20014 | -0.446 | -0.3339 | Yes |
| 25 | EGF | na | 20244 | -0.471 | -0.2944 | Yes |
| 26 | SOS1 | na | 20563 | -0.511 | -0.2548 | Yes |
| 27 | STAT3 | na | 20708 | -0.529 | -0.2052 | Yes |
| 28 | MAP3K1 | na | 20919 | -0.563 | -0.1551 | Yes |
| 29 | STAT5B | na | 20976 | -0.573 | -0.0970 | Yes |
| 30 | PLCG1 | na | 21230 | -0.627 | -0.0420 | Yes |
| 31 | PIK3R1 | na | 21419 | -0.674 | 0.0209 | Yes |
Table: GSEA details [plain text format]

  

Fig 2: BIOCARTA\_EGF\_PATHWAY      
 Blue-Pink O' Gram in the Space of the Analyzed GeneSet

  

Fig 3: BIOCARTA\_EGF\_PATHWAY: Random ES distribution      
 Gene set null distribution of ES for **BIOCARTA\_EGF\_PATHWAY**

  
