# Supplementary material for: Regulation of microglia related neuroinflammation contributes to the protective effect of Gelsevirine on ischemic stroke
Source: Front Immunol. 2023 Mar 30;14:1164278. doi: 10.3389/fimmu.2023.1164278 (PMC10098192; doi:10.3389/fimmu.2023.1164278)
Supplement: Supplementary file 6 [file DataSheet_6.zip › fig 5 raw/fig 5-G raw/inflammation.Gsea.1649955060129/BIOCARTA_ERK_PATHWAY.html]

Details for gene set BIOCARTA\_ERK\_PATHWAY[GSEA]

|  || Dataset | OGD\_DRUG\_DRUG.OGD\_FRUG.cls#Gs\_versus\_MCAO.OGD\_FRUG.cls#Gs\_versus\_MCAO\_repos |
| Phenotype | OGD\_FRUG.cls#Gs\_versus\_MCAO\_repos |
| Upregulated in class | MCAO |
| GeneSet | BIOCARTA\_ERK\_PATHWAY |
| Enrichment Score (ES) | -0.6242331 |
| Normalized Enrichment Score (NES) | -1.4977862 |
| Nominal p-value | 0.0155521 |
| FDR q-value | 0.09387621 |
| FWER p-Value | 0.721 |
Table: GSEA Results Summary

  

Fig 1: Enrichment plot: BIOCARTA\_ERK\_PATHWAY      
 Profile of the Running ES Score & Positions of GeneSet Members on the Rank Ordered List

  

| SYMBOL | TITLE | RANK IN GENE LIST | RANK METRIC SCORE | RUNNING ES | CORE ENRICHMENT || 1 | HRAS | na | 2716 | 0.268 | -0.0836 | No |
| 2 | MAP2K2 | na | 4692 | 0.115 | -0.1565 | No |
| 3 | MAPK3 | na | 4918 | 0.100 | -0.1516 | No |
| 4 | MYC | na | 5587 | 0.064 | -0.1725 | No |
| 5 | GNAS | na | 5608 | 0.063 | -0.1639 | No |
| 6 | GRB2 | na | 5726 | 0.057 | -0.1607 | No |
| 7 | PPP2CA | na | 6335 | 0.027 | -0.1843 | No |
| 8 | GNB1 | na | 6946 | 0.004 | -0.2117 | No |
| 9 | GNGT1 | na | 7714 | 0.000 | -0.2468 | No |
| 10 | NGF | na | 13604 | -0.010 | -0.5148 | No |
| 11 | MKNK1 | na | 13934 | -0.018 | -0.5271 | No |
| 12 | MAP2K1 | na | 15179 | -0.079 | -0.5720 | No |
| 13 | NGFR | na | 15225 | -0.081 | -0.5617 | No |
| 14 | MAPK1 | na | 16234 | -0.143 | -0.5861 | Yes |
| 15 | ITGB1 | na | 16661 | -0.174 | -0.5792 | Yes |
| 16 | RAF1 | na | 17251 | -0.212 | -0.5739 | Yes |
| 17 | PTPRR | na | 18352 | -0.294 | -0.5796 | Yes |
| 18 | RPS6KA1 | na | 18696 | -0.320 | -0.5466 | Yes |
| 19 | EGFR | na | 19043 | -0.354 | -0.5087 | Yes |
| 20 | SHC1 | na | 19109 | -0.361 | -0.4569 | Yes |
| 21 | MKNK2 | na | 19425 | -0.390 | -0.4120 | Yes |
| 22 | ELK1 | na | 19706 | -0.417 | -0.3615 | Yes |
| 23 | PDGFRA | na | 19955 | -0.440 | -0.3059 | Yes |
| 24 | RPS6KA5 | na | 20147 | -0.461 | -0.2446 | Yes |
| 25 | SRC | na | 20255 | -0.472 | -0.1778 | Yes |
| 26 | SOS1 | na | 20563 | -0.511 | -0.1142 | Yes |
| 27 | STAT3 | na | 20708 | -0.529 | -0.0404 | Yes |
| 28 | IGF1R | na | 21187 | -0.617 | 0.0315 | Yes |
Table: GSEA details [plain text format]

  

Fig 2: BIOCARTA\_ERK\_PATHWAY      
 Blue-Pink O' Gram in the Space of the Analyzed GeneSet

  

Fig 3: BIOCARTA\_ERK\_PATHWAY: Random ES distribution      
 Gene set null distribution of ES for **BIOCARTA\_ERK\_PATHWAY**

  
