# Supplementary material for: Regulation of microglia related neuroinflammation contributes to the protective effect of Gelsevirine on ischemic stroke
Source: Front Immunol. 2023 Mar 30;14:1164278. doi: 10.3389/fimmu.2023.1164278 (PMC10098192; doi:10.3389/fimmu.2023.1164278)
Supplement: Supplementary file 6 [file DataSheet_6.zip › fig 5 raw/fig 5-G raw/inflammation.Gsea.1649955060129/BIOCARTA_GSK3_PATHWAY.html]

Details for gene set BIOCARTA\_GSK3\_PATHWAY[GSEA]

|  || Dataset | OGD\_DRUG\_DRUG.OGD\_FRUG.cls#Gs\_versus\_MCAO.OGD\_FRUG.cls#Gs\_versus\_MCAO\_repos |
| Phenotype | OGD\_FRUG.cls#Gs\_versus\_MCAO\_repos |
| Upregulated in class | MCAO |
| GeneSet | BIOCARTA\_GSK3\_PATHWAY |
| Enrichment Score (ES) | -0.6250816 |
| Normalized Enrichment Score (NES) | -1.51021 |
| Nominal p-value | 0.016 |
| FDR q-value | 0.09214507 |
| FWER p-Value | 0.673 |
Table: GSEA Results Summary

  

Fig 1: Enrichment plot: BIOCARTA\_GSK3\_PATHWAY      
 Profile of the Running ES Score & Positions of GeneSet Members on the Rank Ordered List

  

| SYMBOL | TITLE | RANK IN GENE LIST | RANK METRIC SCORE | RUNNING ES | CORE ENRICHMENT || 1 | LY96 | na | 1426 | 0.408 | -0.0135 | No |
| 2 | GNAI1 | na | 4304 | 0.143 | -0.1271 | No |
| 3 | CD14 | na | 4569 | 0.124 | -0.1234 | No |
| 4 | PPP2CA | na | 6335 | 0.027 | -0.2007 | No |
| 5 | MYD88 | na | 6996 | 0.001 | -0.2308 | No |
| 6 | WNT1 | na | 15037 | -0.070 | -0.5899 | No |
| 7 | RELA | na | 15584 | -0.103 | -0.6018 | No |
| 8 | IRAK1 | na | 15809 | -0.115 | -0.5974 | No |
| 9 | TOLLIP | na | 16008 | -0.129 | -0.5901 | No |
| 10 | LEF1 | na | 16773 | -0.181 | -0.6022 | Yes |
| 11 | AKT1 | na | 16827 | -0.184 | -0.5813 | Yes |
| 12 | EIF2AK2 | na | 16842 | -0.185 | -0.5585 | Yes |
| 13 | CCND1 | na | 17421 | -0.225 | -0.5564 | Yes |
| 14 | GJA1 | na | 17476 | -0.228 | -0.5299 | Yes |
| 15 | DVL1 | na | 17888 | -0.261 | -0.5156 | Yes |
| 16 | AXIN1 | na | 18098 | -0.276 | -0.4902 | Yes |
| 17 | CTNNB1 | na | 18285 | -0.290 | -0.4619 | Yes |
| 18 | PIK3CA | na | 18513 | -0.305 | -0.4336 | Yes |
| 19 | NFKB1 | na | 19213 | -0.371 | -0.4186 | Yes |
| 20 | TLR4 | na | 19232 | -0.372 | -0.3723 | Yes |
| 21 | FZD1 | na | 19601 | -0.406 | -0.3375 | Yes |
| 22 | GSK3B | na | 20096 | -0.455 | -0.3025 | Yes |
| 23 | PDPK1 | na | 20100 | -0.455 | -0.2448 | Yes |
| 24 | TIRAP | na | 20814 | -0.548 | -0.2080 | Yes |
| 25 | APC | na | 21200 | -0.619 | -0.1471 | Yes |
| 26 | PIK3R1 | na | 21419 | -0.674 | -0.0715 | Yes |
| 27 | LBP | na | 21555 | -0.728 | 0.0146 | Yes |
Table: GSEA details [plain text format]

  

Fig 2: BIOCARTA\_GSK3\_PATHWAY      
 Blue-Pink O' Gram in the Space of the Analyzed GeneSet

  

Fig 3: BIOCARTA\_GSK3\_PATHWAY: Random ES distribution      
 Gene set null distribution of ES for **BIOCARTA\_GSK3\_PATHWAY**

  
