# Supplementary material for: Regulation of microglia related neuroinflammation contributes to the protective effect of Gelsevirine on ischemic stroke
Source: Front Immunol. 2023 Mar 30;14:1164278. doi: 10.3389/fimmu.2023.1164278 (PMC10098192; doi:10.3389/fimmu.2023.1164278)
Supplement: Supplementary file 6 [file DataSheet_6.zip › fig 5 raw/fig 5-G raw/inflammation.Gsea.1649955060129/CREIGHTON_AKT1_SIGNALING_VIA_MTOR_DN.html]

Details for gene set CREIGHTON\_AKT1\_SIGNALING\_VIA\_MTOR\_DN[GSEA]

|  || Dataset | OGD\_DRUG\_DRUG.OGD\_FRUG.cls#Gs\_versus\_MCAO.OGD\_FRUG.cls#Gs\_versus\_MCAO\_repos |
| Phenotype | OGD\_FRUG.cls#Gs\_versus\_MCAO\_repos |
| Upregulated in class | Gs |
| GeneSet | CREIGHTON\_AKT1\_SIGNALING\_VIA\_MTOR\_DN |
| Enrichment Score (ES) | 0.5942556 |
| Normalized Enrichment Score (NES) | 1.4628794 |
| Nominal p-value | 0.04032258 |
| FDR q-value | 0.10743543 |
| FWER p-Value | 0.708 |
Table: GSEA Results Summary

  

Fig 1: Enrichment plot: CREIGHTON\_AKT1\_SIGNALING\_VIA\_MTOR\_DN      
 Profile of the Running ES Score & Positions of GeneSet Members on the Rank Ordered List

  

| SYMBOL | TITLE | RANK IN GENE LIST | RANK METRIC SCORE | RUNNING ES | CORE ENRICHMENT || 1 | ATP6V1F | na | 1110 | 0.447 | 0.0634 | Yes |
| 2 | ATP6V0B | na | 1590 | 0.389 | 0.1410 | Yes |
| 3 | PPP4C | na | 1666 | 0.378 | 0.2342 | Yes |
| 4 | CIB1 | na | 2050 | 0.332 | 0.3017 | Yes |
| 5 | MIF | na | 2907 | 0.250 | 0.3264 | Yes |
| 6 | GOT1 | na | 2969 | 0.244 | 0.3860 | Yes |
| 7 | ATP6V0C | na | 3364 | 0.209 | 0.4215 | Yes |
| 8 | MRPS7 | na | 3451 | 0.202 | 0.4693 | Yes |
| 9 | CTSA | na | 3541 | 0.197 | 0.5155 | Yes |
| 10 | PAFAH1B3 | na | 3879 | 0.175 | 0.5448 | Yes |
| 11 | YWHAB | na | 4657 | 0.117 | 0.5392 | Yes |
| 12 | TNFRSF12A | na | 4833 | 0.106 | 0.5583 | Yes |
| 13 | TSPAN1 | na | 4877 | 0.103 | 0.5827 | Yes |
| 14 | ALDOA | na | 5121 | 0.089 | 0.5943 | Yes |
| 15 | TUBB4B | na | 5567 | 0.065 | 0.5905 | No |
| 16 | KRT8 | na | 13333 | -0.002 | 0.2357 | No |
| 17 | ATP6AP1 | na | 13950 | -0.019 | 0.2124 | No |
| 18 | PPP2R1A | na | 14223 | -0.029 | 0.2072 | No |
| 19 | PFKL | na | 15100 | -0.074 | 0.1860 | No |
| 20 | TOM1 | na | 15597 | -0.104 | 0.1898 | No |
| 21 | RGL2 | na | 16174 | -0.139 | 0.1990 | No |
| 22 | DHCR7 | na | 17666 | -0.242 | 0.1926 | No |
Table: GSEA details [plain text format]

  

Fig 2: CREIGHTON\_AKT1\_SIGNALING\_VIA\_MTOR\_DN      
 Blue-Pink O' Gram in the Space of the Analyzed GeneSet

  

Fig 3: CREIGHTON\_AKT1\_SIGNALING\_VIA\_MTOR\_DN: Random ES distribution      
 Gene set null distribution of ES for **CREIGHTON\_AKT1\_SIGNALING\_VIA\_MTOR\_DN**

  
