# Supplementary material for: Regulation of microglia related neuroinflammation contributes to the protective effect of Gelsevirine on ischemic stroke
Source: Front Immunol. 2023 Mar 30;14:1164278. doi: 10.3389/fimmu.2023.1164278 (PMC10098192; doi:10.3389/fimmu.2023.1164278)
Supplement: Supplementary file 6 [file DataSheet_6.zip › fig 5 raw/fig 5-G raw/inflammation.Gsea.1649955060129/DAUER_STAT3_TARGETS_DN.html]

Details for gene set DAUER\_STAT3\_TARGETS\_DN[GSEA]

|  || Dataset | OGD\_DRUG\_DRUG.OGD\_FRUG.cls#Gs\_versus\_MCAO.OGD\_FRUG.cls#Gs\_versus\_MCAO\_repos |
| Phenotype | OGD\_FRUG.cls#Gs\_versus\_MCAO\_repos |
| Upregulated in class | Gs |
| GeneSet | DAUER\_STAT3\_TARGETS\_DN |
| Enrichment Score (ES) | 0.3582559 |
| Normalized Enrichment Score (NES) | 1.0198072 |
| Nominal p-value | 0.40369394 |
| FDR q-value | 0.7133107 |
| FWER p-Value | 1.0 |
Table: GSEA Results Summary

  

Fig 1: Enrichment plot: DAUER\_STAT3\_TARGETS\_DN      
 Profile of the Running ES Score & Positions of GeneSet Members on the Rank Ordered List

  

| SYMBOL | TITLE | RANK IN GENE LIST | RANK METRIC SCORE | RUNNING ES | CORE ENRICHMENT || 1 | IFITM1 | na | 13 | 1.732 | 0.1597 | Yes |
| 2 | AQP3 | na | 175 | 0.828 | 0.2290 | Yes |
| 3 | TRIM5 | na | 1267 | 0.426 | 0.2184 | Yes |
| 4 | GRB14 | na | 1413 | 0.410 | 0.2497 | Yes |
| 5 | HSPA1B | na | 1633 | 0.382 | 0.2751 | Yes |
| 6 | SP110 | na | 2191 | 0.317 | 0.2789 | Yes |
| 7 | ISG15 | na | 2652 | 0.274 | 0.2832 | Yes |
| 8 | IFI35 | na | 2904 | 0.250 | 0.2949 | Yes |
| 9 | IFIT3 | na | 2985 | 0.243 | 0.3137 | Yes |
| 10 | ID3 | na | 3103 | 0.233 | 0.3299 | Yes |
| 11 | USP18 | na | 3713 | 0.185 | 0.3191 | Yes |
| 12 | DNAJB4 | na | 3803 | 0.180 | 0.3317 | Yes |
| 13 | PGRMC2 | na | 3910 | 0.172 | 0.3427 | Yes |
| 14 | LAMP3 | na | 3919 | 0.172 | 0.3583 | Yes |
| 15 | TMEM97 | na | 4449 | 0.133 | 0.3463 | No |
| 16 | IFIT1 | na | 5119 | 0.089 | 0.3239 | No |
| 17 | IFI27 | na | 5188 | 0.085 | 0.3287 | No |
| 18 | LAP3 | na | 5388 | 0.074 | 0.3265 | No |
| 19 | HEXIM1 | na | 5597 | 0.063 | 0.3228 | No |
| 20 | HSPA1A | na | 5784 | 0.053 | 0.3191 | No |
| 21 | IFI44 | na | 6697 | 0.013 | 0.2786 | No |
| 22 | IFIH1 | na | 8697 | 0.000 | 0.1870 | No |
| 23 | TRANK1 | na | 10330 | 0.000 | 0.1123 | No |
| 24 | TRIM14 | na | 11675 | 0.000 | 0.0507 | No |
| 25 | HPGD | na | 12280 | 0.000 | 0.0231 | No |
| 26 | ST8SIA4 | na | 12870 | 0.000 | -0.0039 | No |
| 27 | PARP12 | na | 13472 | -0.007 | -0.0308 | No |
| 28 | HERC6 | na | 13768 | -0.013 | -0.0431 | No |
| 29 | IRF7 | na | 14245 | -0.029 | -0.0622 | No |
| 30 | PDK4 | na | 16081 | -0.133 | -0.1339 | No |
| 31 | TLR3 | na | 16204 | -0.141 | -0.1264 | No |
| 32 | EIF2AK2 | na | 16842 | -0.185 | -0.1385 | No |
| 33 | OAS3 | na | 17110 | -0.203 | -0.1319 | No |
| 34 | STAT1 | na | 17788 | -0.252 | -0.1396 | No |
| 35 | BCL2L13 | na | 18525 | -0.306 | -0.1450 | No |
| 36 | PRPF4B | na | 19244 | -0.373 | -0.1434 | No |
| 37 | TDRD7 | na | 19395 | -0.387 | -0.1144 | No |
| 38 | DDX58 | na | 19861 | -0.432 | -0.0957 | No |
| 39 | OAS2 | na | 20043 | -0.449 | -0.0624 | No |
| 40 | DDX60 | na | 21581 | -0.742 | -0.0642 | No |
| 41 | TNFSF10 | na | 21692 | -0.838 | 0.0084 | No |
Table: GSEA details [plain text format]

  

Fig 2: DAUER\_STAT3\_TARGETS\_DN      
 Blue-Pink O' Gram in the Space of the Analyzed GeneSet

  

Fig 3: DAUER\_STAT3\_TARGETS\_DN: Random ES distribution      
 Gene set null distribution of ES for **DAUER\_STAT3\_TARGETS\_DN**

  
