# Supplementary material for: Regulation of microglia related neuroinflammation contributes to the protective effect of Gelsevirine on ischemic stroke
Source: Front Immunol. 2023 Mar 30;14:1164278. doi: 10.3389/fimmu.2023.1164278 (PMC10098192; doi:10.3389/fimmu.2023.1164278)
Supplement: Supplementary file 6 [file DataSheet_6.zip › fig 5 raw/fig 5-G raw/inflammation.Gsea.1649955060129/DEBOSSCHER_NFKB_TARGETS_REPRESSED_BY_GLUCOCORTICOIDS.html]

Details for gene set DEBOSSCHER\_NFKB\_TARGETS\_REPRESSED\_BY\_GLUCOCORTICOIDS[GSEA]

|  || Dataset | OGD\_DRUG\_DRUG.OGD\_FRUG.cls#Gs\_versus\_MCAO.OGD\_FRUG.cls#Gs\_versus\_MCAO\_repos |
| Phenotype | OGD\_FRUG.cls#Gs\_versus\_MCAO\_repos |
| Upregulated in class | Gs |
| GeneSet | DEBOSSCHER\_NFKB\_TARGETS\_REPRESSED\_BY\_GLUCOCORTICOIDS |
| Enrichment Score (ES) | 0.3109582 |
| Normalized Enrichment Score (NES) | 0.7658361 |
| Nominal p-value | 0.86445016 |
| FDR q-value | 0.9653211 |
| FWER p-Value | 1.0 |
Table: GSEA Results Summary

  

Fig 1: Enrichment plot: DEBOSSCHER\_NFKB\_TARGETS\_REPRESSED\_BY\_GLUCOCORTICOIDS      
 Profile of the Running ES Score & Positions of GeneSet Members on the Rank Ordered List

  

| SYMBOL | TITLE | RANK IN GENE LIST | RANK METRIC SCORE | RUNNING ES | CORE ENRICHMENT || 1 | CCL11 | na | 386 | 0.637 | 0.1555 | Yes |
| 2 | CCL5 | na | 495 | 0.590 | 0.3110 | Yes |
| 3 | IL11 | na | 3251 | 0.219 | 0.2443 | No |
| 4 | EDN1 | na | 4228 | 0.148 | 0.2398 | No |
| 5 | IL13 | na | 4636 | 0.119 | 0.2537 | No |
| 6 | IL2 | na | 5219 | 0.084 | 0.2498 | No |
| 7 | NOS2 | na | 5724 | 0.057 | 0.2421 | No |
| 8 | TNF | na | 5838 | 0.050 | 0.2506 | No |
| 9 | IL4 | na | 8412 | 0.000 | 0.1328 | No |
| 10 | IL3 | na | 8413 | 0.000 | 0.1328 | No |
| 11 | IL9 | na | 8414 | 0.000 | 0.1328 | No |
| 12 | SELE | na | 10376 | 0.000 | 0.0431 | No |
| 13 | CCL3 | na | 12847 | 0.000 | -0.0699 | No |
| 14 | IL12A | na | 12867 | 0.000 | -0.0708 | No |
| 15 | IL1B | na | 14311 | -0.032 | -0.1280 | No |
| 16 | IL5 | na | 16224 | -0.142 | -0.1769 | No |
| 17 | PTGS2 | na | 16449 | -0.158 | -0.1443 | No |
| 18 | IL18 | na | 16753 | -0.179 | -0.1093 | No |
| 19 | VCAM1 | na | 17450 | -0.226 | -0.0797 | No |
| 20 | IL6 | na | 19212 | -0.371 | -0.0595 | No |
| 21 | ICAM1 | na | 21398 | -0.667 | 0.0218 | No |
Table: GSEA details [plain text format]

  

Fig 2: DEBOSSCHER\_NFKB\_TARGETS\_REPRESSED\_BY\_GLUCOCORTICOIDS      
 Blue-Pink O' Gram in the Space of the Analyzed GeneSet

  

Fig 3: DEBOSSCHER\_NFKB\_TARGETS\_REPRESSED\_BY\_GLUCOCORTICOIDS: Random ES distribution      
 Gene set null distribution of ES for **DEBOSSCHER\_NFKB\_TARGETS\_REPRESSED\_BY\_GLUCOCORTICOIDS**

  
