# Supplementary material for: Regulation of microglia related neuroinflammation contributes to the protective effect of Gelsevirine on ischemic stroke
Source: Front Immunol. 2023 Mar 30;14:1164278. doi: 10.3389/fimmu.2023.1164278 (PMC10098192; doi:10.3389/fimmu.2023.1164278)
Supplement: Supplementary file 6 [file DataSheet_6.zip › fig 5 raw/fig 5-G raw/inflammation.Gsea.1649955060129/FIRESTEIN_CTNNB1_PATHWAY.html]

Details for gene set FIRESTEIN\_CTNNB1\_PATHWAY[GSEA]

|  || Dataset | OGD\_DRUG\_DRUG.OGD\_FRUG.cls#Gs\_versus\_MCAO.OGD\_FRUG.cls#Gs\_versus\_MCAO\_repos |
| Phenotype | OGD\_FRUG.cls#Gs\_versus\_MCAO\_repos |
| Upregulated in class | MCAO |
| GeneSet | FIRESTEIN\_CTNNB1\_PATHWAY |
| Enrichment Score (ES) | -0.63810915 |
| Normalized Enrichment Score (NES) | -1.5980067 |
| Nominal p-value | 0.0109375 |
| FDR q-value | 0.058850575 |
| FWER p-Value | 0.345 |
Table: GSEA Results Summary

  

Fig 1: Enrichment plot: FIRESTEIN\_CTNNB1\_PATHWAY      
 Profile of the Running ES Score & Positions of GeneSet Members on the Rank Ordered List

  

| SYMBOL | TITLE | RANK IN GENE LIST | RANK METRIC SCORE | RUNNING ES | CORE ENRICHMENT || 1 | LTK | na | 2492 | 0.287 | -0.0819 | No |
| 2 | NRAS | na | 4669 | 0.117 | -0.1685 | No |
| 3 | PDK3 | na | 5403 | 0.073 | -0.1938 | No |
| 4 | DKC1 | na | 5645 | 0.060 | -0.1980 | No |
| 5 | CHEK2 | na | 6729 | 0.012 | -0.2463 | No |
| 6 | SRPK3 | na | 8530 | 0.000 | -0.3287 | No |
| 7 | GABRA3 | na | 9343 | 0.000 | -0.3659 | No |
| 8 | KSR2 | na | 13429 | -0.006 | -0.5522 | No |
| 9 | CSNK1G3 | na | 14360 | -0.034 | -0.5910 | No |
| 10 | STK25 | na | 15043 | -0.071 | -0.6143 | No |
| 11 | MAP2K1 | na | 15179 | -0.079 | -0.6116 | No |
| 12 | CSNK1E | na | 15223 | -0.081 | -0.6044 | No |
| 13 | CDK8 | na | 15275 | -0.084 | -0.5973 | No |
| 14 | DAPK2 | na | 15489 | -0.097 | -0.5962 | No |
| 15 | FER | na | 15870 | -0.120 | -0.6002 | No |
| 16 | PLK4 | na | 16699 | -0.177 | -0.6183 | Yes |
| 17 | FOXO4 | na | 16849 | -0.186 | -0.6043 | Yes |
| 18 | PKN1 | na | 17142 | -0.205 | -0.5947 | Yes |
| 19 | EPHA2 | na | 17359 | -0.220 | -0.5799 | Yes |
| 20 | PLK1 | na | 17988 | -0.269 | -0.5786 | Yes |
| 21 | DCLK2 | na | 18674 | -0.319 | -0.5742 | Yes |
| 22 | RPS6KA1 | na | 18696 | -0.320 | -0.5392 | Yes |
| 23 | SLK | na | 19230 | -0.372 | -0.5220 | Yes |
| 24 | CAMKK2 | na | 19237 | -0.372 | -0.4805 | Yes |
| 25 | SYT11 | na | 19365 | -0.383 | -0.4434 | Yes |
| 26 | ZAK | na | 19557 | -0.403 | -0.4070 | Yes |
| 27 | LATS1 | na | 20439 | -0.495 | -0.3919 | Yes |
| 28 | ULK1 | na | 20527 | -0.506 | -0.3392 | Yes |
| 29 | TAOK1 | na | 20806 | -0.547 | -0.2906 | Yes |
| 30 | DGKH | na | 21106 | -0.599 | -0.2372 | Yes |
| 31 | MAP3K14 | na | 21380 | -0.662 | -0.1754 | Yes |
| 32 | PEAK1 | na | 21524 | -0.717 | -0.1016 | Yes |
| 33 | PTK2B | na | 21800 | -1.050 | 0.0034 | Yes |
Table: GSEA details [plain text format]

  

Fig 2: FIRESTEIN\_CTNNB1\_PATHWAY      
 Blue-Pink O' Gram in the Space of the Analyzed GeneSet

  

Fig 3: FIRESTEIN\_CTNNB1\_PATHWAY: Random ES distribution      
 Gene set null distribution of ES for **FIRESTEIN\_CTNNB1\_PATHWAY**

  
