# Supplementary material for: Regulation of microglia related neuroinflammation contributes to the protective effect of Gelsevirine on ischemic stroke
Source: Front Immunol. 2023 Mar 30;14:1164278. doi: 10.3389/fimmu.2023.1164278 (PMC10098192; doi:10.3389/fimmu.2023.1164278)
Supplement: Supplementary file 6 [file DataSheet_6.zip › fig 5 raw/fig 5-G raw/inflammation.Gsea.1649955060129/GRESHOCK_CANCER_COPY_NUMBER_UP.html]

Details for gene set GRESHOCK\_CANCER\_COPY\_NUMBER\_UP[GSEA]

|  || Dataset | OGD\_DRUG\_DRUG.OGD\_FRUG.cls#Gs\_versus\_MCAO.OGD\_FRUG.cls#Gs\_versus\_MCAO\_repos |
| Phenotype | OGD\_FRUG.cls#Gs\_versus\_MCAO\_repos |
| Upregulated in class | MCAO |
| GeneSet | GRESHOCK\_CANCER\_COPY\_NUMBER\_UP |
| Enrichment Score (ES) | -0.5061559 |
| Normalized Enrichment Score (NES) | -1.6436057 |
| Nominal p-value | 0.0 |
| FDR q-value | 0.05762045 |
| FWER p-Value | 0.204 |
Table: GSEA Results Summary

  

Fig 1: Enrichment plot: GRESHOCK\_CANCER\_COPY\_NUMBER\_UP      
 Profile of the Running ES Score & Positions of GeneSet Members on the Rank Ordered List

  

| SYMBOL | TITLE | RANK IN GENE LIST | RANK METRIC SCORE | RUNNING ES | CORE ENRICHMENT || 1 | CBFA2T3 | na | 184 | 0.817 | 0.0049 | No |
| 2 | HIST1H4I | na | 232 | 0.756 | 0.0152 | No |
| 3 | ACSL6 | na | 312 | 0.686 | 0.0228 | No |
| 4 | COX6C | na | 440 | 0.616 | 0.0270 | No |
| 5 | DDIT3 | na | 490 | 0.591 | 0.0345 | No |
| 6 | TFPT | na | 491 | 0.591 | 0.0442 | No |
| 7 | RPL22 | na | 517 | 0.582 | 0.0526 | No |
| 8 | MLF1 | na | 659 | 0.537 | 0.0549 | No |
| 9 | XPA | na | 710 | 0.524 | 0.0612 | No |
| 10 | CCNB1IP1 | na | 809 | 0.496 | 0.0648 | No |
| 11 | NACA | na | 1083 | 0.452 | 0.0596 | No |
| 12 | FANCC | na | 1528 | 0.397 | 0.0456 | No |
| 13 | LYL1 | na | 1684 | 0.376 | 0.0447 | No |
| 14 | SRSF3 | na | 1888 | 0.351 | 0.0411 | No |
| 15 | CNBP | na | 1932 | 0.345 | 0.0447 | No |
| 16 | HNF1A | na | 2324 | 0.301 | 0.0316 | No |
| 17 | SET | na | 2415 | 0.295 | 0.0323 | No |
| 18 | CHIC2 | na | 2437 | 0.293 | 0.0361 | No |
| 19 | FANCF | na | 2618 | 0.277 | 0.0324 | No |
| 20 | HRAS | na | 2716 | 0.268 | 0.0323 | No |
| 21 | SDHC | na | 2894 | 0.251 | 0.0282 | No |
| 22 | CDK4 | na | 3240 | 0.219 | 0.0159 | No |
| 23 | DDX10 | na | 3815 | 0.179 | -0.0077 | No |
| 24 | DDB2 | na | 3976 | 0.167 | -0.0123 | No |
| 25 | CARS | na | 4092 | 0.156 | -0.0151 | No |
| 26 | RPN1 | na | 4205 | 0.149 | -0.0178 | No |
| 27 | ETV4 | na | 4383 | 0.138 | -0.0237 | No |
| 28 | TFG | na | 4555 | 0.125 | -0.0296 | No |
| 29 | FIP1L1 | na | 4575 | 0.124 | -0.0284 | No |
| 30 | DEK | na | 4586 | 0.123 | -0.0269 | No |
| 31 | NRAS | na | 4669 | 0.117 | -0.0288 | No |
| 32 | EIF4A2 | na | 4770 | 0.110 | -0.0316 | No |
| 33 | TCEA1 | na | 4818 | 0.107 | -0.0320 | No |
| 34 | PRCC | na | 4959 | 0.098 | -0.0368 | No |
| 35 | GPHN | na | 5053 | 0.093 | -0.0396 | No |
| 36 | HSP90AB1 | na | 5242 | 0.082 | -0.0469 | No |
| 37 | HSP90AA1 | na | 5244 | 0.082 | -0.0456 | No |
| 38 | STK11 | na | 5246 | 0.082 | -0.0443 | No |
| 39 | MUTYH | na | 5431 | 0.072 | -0.0517 | No |
| 40 | VHL | na | 5444 | 0.072 | -0.0510 | No |
| 41 | SMARCB1 | na | 5459 | 0.071 | -0.0505 | No |
| 42 | LCK | na | 5511 | 0.068 | -0.0518 | No |
| 43 | MYC | na | 5587 | 0.064 | -0.0542 | No |
| 44 | SFPQ | na | 5734 | 0.056 | -0.0600 | No |
| 45 | TPM3 | na | 5818 | 0.051 | -0.0630 | No |
| 46 | TSHR | na | 5821 | 0.051 | -0.0623 | No |
| 47 | GOLGA5 | na | 5909 | 0.046 | -0.0655 | No |
| 48 | LHFP | na | 6014 | 0.040 | -0.0697 | No |
| 49 | NTRK1 | na | 6192 | 0.033 | -0.0773 | No |
| 50 | SH3GL1 | na | 6292 | 0.029 | -0.0814 | No |
| 51 | FAS | na | 6465 | 0.023 | -0.0890 | No |
| 52 | ITK | na | 6528 | 0.019 | -0.0915 | No |
| 53 | BCL3 | na | 6597 | 0.017 | -0.0944 | No |
| 54 | ASPSCR1 | na | 6690 | 0.013 | -0.0984 | No |
| 55 | CHEK2 | na | 6729 | 0.012 | -0.1000 | No |
| 56 | RANBP17 | na | 6793 | 0.010 | -0.1027 | No |
| 57 | HMGA2 | na | 6817 | 0.009 | -0.1037 | No |
| 58 | CCND3 | na | 6975 | 0.002 | -0.1109 | No |
| 59 | CEBPA | na | 7443 | 0.000 | -0.1325 | No |
| 60 | IL21R | na | 7454 | 0.000 | -0.1329 | No |
| 61 | PHOX2B | na | 7761 | 0.000 | -0.1471 | No |
| 62 | CHN1 | na | 7829 | 0.000 | -0.1502 | No |
| 63 | FEV | na | 7974 | 0.000 | -0.1568 | No |
| 64 | NTRK3 | na | 8060 | 0.000 | -0.1608 | No |
| 65 | KIT | na | 8667 | 0.000 | -0.1888 | No |
| 66 | JAZF1 | na | 9378 | 0.000 | -0.2216 | No |
| 67 | LMO2 | na | 9438 | 0.000 | -0.2243 | No |
| 68 | BCL11B | na | 9786 | 0.000 | -0.2403 | No |
| 69 | BCL11A | na | 9787 | 0.000 | -0.2403 | No |
| 70 | SYK | na | 10020 | 0.000 | -0.2511 | No |
| 71 | PRDM16 | na | 10170 | 0.000 | -0.2580 | No |
| 72 | CDKN2A | na | 10240 | 0.000 | -0.2611 | No |
| 73 | HOXD11 | na | 10652 | 0.000 | -0.2801 | No |
| 74 | HOXC13 | na | 10817 | 0.000 | -0.2877 | No |
| 75 | HOXC11 | na | 10819 | 0.000 | -0.2878 | No |
| 76 | WT1 | na | 10827 | 0.000 | -0.2881 | No |
| 77 | HOXA13 | na | 11187 | 0.000 | -0.3047 | No |
| 78 | HOXA11 | na | 11188 | 0.000 | -0.3047 | No |
| 79 | MYCN | na | 11199 | 0.000 | -0.3051 | No |
| 80 | TLX3 | na | 11221 | 0.000 | -0.3061 | No |
| 81 | TLX1 | na | 11223 | 0.000 | -0.3062 | No |
| 82 | HOXA9 | na | 11428 | 0.000 | -0.3156 | No |
| 83 | RHOH | na | 11519 | 0.000 | -0.3197 | No |
| 84 | OLIG2 | na | 11798 | 0.000 | -0.3326 | No |
| 85 | IRF4 | na | 12103 | 0.000 | -0.3466 | No |
| 86 | POU2AF1 | na | 12421 | 0.000 | -0.3613 | No |
| 87 | ROS1 | na | 12807 | 0.000 | -0.3791 | No |
| 88 | MAFB | na | 13119 | 0.000 | -0.3935 | No |
| 89 | MAF | na | 13283 | -0.001 | -0.4010 | No |
| 90 | TFEB | na | 13389 | -0.005 | -0.4058 | No |
| 91 | ZBTB16 | na | 13418 | -0.006 | -0.4070 | No |
| 92 | ERG | na | 13450 | -0.006 | -0.4083 | No |
| 93 | FUS | na | 13538 | -0.008 | -0.4122 | No |
| 94 | RET | na | 13693 | -0.012 | -0.4191 | No |
| 95 | LIFR | na | 13712 | -0.013 | -0.4197 | No |
| 96 | FLT3 | na | 13729 | -0.013 | -0.4202 | No |
| 97 | CDH11 | na | 13754 | -0.013 | -0.4211 | No |
| 98 | BCL10 | na | 14057 | -0.022 | -0.4347 | No |
| 99 | PAX3 | na | 14058 | -0.022 | -0.4344 | No |
| 100 | FLT4 | na | 14284 | -0.031 | -0.4443 | No |
| 101 | FLI1 | na | 14298 | -0.032 | -0.4443 | No |
| 102 | MSH2 | na | 14302 | -0.032 | -0.4440 | No |
| 103 | ATF1 | na | 14349 | -0.034 | -0.4455 | No |
| 104 | EWSR1 | na | 14527 | -0.042 | -0.4530 | No |
| 105 | TPM4 | na | 14555 | -0.043 | -0.4536 | No |
| 106 | SMAD4 | na | 14652 | -0.048 | -0.4572 | No |
| 107 | AFF3 | na | 14714 | -0.052 | -0.4592 | No |
| 108 | FCGR2B | na | 14786 | -0.055 | -0.4616 | No |
| 109 | TEC | na | 14828 | -0.057 | -0.4625 | No |
| 110 | ELL | na | 14892 | -0.062 | -0.4644 | No |
| 111 | ABI1 | na | 14894 | -0.062 | -0.4634 | No |
| 112 | PRKAR1A | na | 14987 | -0.067 | -0.4666 | No |
| 113 | HOXD13 | na | 15013 | -0.068 | -0.4666 | No |
| 114 | JAK2 | na | 15175 | -0.079 | -0.4728 | No |
| 115 | KTN1 | na | 15296 | -0.086 | -0.4769 | No |
| 116 | HMGA1 | na | 15308 | -0.086 | -0.4760 | No |
| 117 | BCL6 | na | 15341 | -0.088 | -0.4760 | No |
| 118 | ARHGAP26 | na | 15568 | -0.102 | -0.4848 | No |
| 119 | MLLT3 | na | 15649 | -0.106 | -0.4868 | No |
| 120 | ERCC3 | na | 15689 | -0.108 | -0.4868 | No |
| 121 | RAD51B | na | 15708 | -0.109 | -0.4858 | No |
| 122 | TMPRSS2 | na | 15729 | -0.110 | -0.4849 | No |
| 123 | TRIM24 | na | 15880 | -0.120 | -0.4899 | No |
| 124 | TAL1 | na | 15907 | -0.122 | -0.4891 | No |
| 125 | PAX8 | na | 16102 | -0.134 | -0.4959 | No |
| 126 | PIM1 | na | 16160 | -0.138 | -0.4962 | No |
| 127 | ATIC | na | 16212 | -0.141 | -0.4962 | No |
| 128 | SS18 | na | 16390 | -0.154 | -0.5019 | No |
| 129 | KLF6 | na | 16483 | -0.160 | -0.5035 | Yes |
| 130 | FLCN | na | 16507 | -0.162 | -0.5019 | Yes |
| 131 | BLM | na | 16562 | -0.166 | -0.5017 | Yes |
| 132 | PICALM | na | 16569 | -0.167 | -0.4992 | Yes |
| 133 | FBXW7 | na | 16581 | -0.168 | -0.4970 | Yes |
| 134 | PMS1 | na | 16589 | -0.168 | -0.4945 | Yes |
| 135 | EXT2 | na | 16706 | -0.177 | -0.4970 | Yes |
| 136 | RARA | na | 16711 | -0.178 | -0.4943 | Yes |
| 137 | AKT2 | na | 16784 | -0.181 | -0.4946 | Yes |
| 138 | RUNX1 | na | 16837 | -0.185 | -0.4940 | Yes |
| 139 | MAP2K4 | na | 16904 | -0.189 | -0.4939 | Yes |
| 140 | BUB1B | na | 16946 | -0.193 | -0.4927 | Yes |
| 141 | GOPC | na | 16965 | -0.194 | -0.4903 | Yes |
| 142 | TNFRSF17 | na | 17041 | -0.198 | -0.4905 | Yes |
| 143 | TAL2 | na | 17045 | -0.198 | -0.4874 | Yes |
| 144 | BIRC3 | na | 17295 | -0.216 | -0.4953 | Yes |
| 145 | FANCE | na | 17323 | -0.218 | -0.4930 | Yes |
| 146 | XPC | na | 17360 | -0.220 | -0.4911 | Yes |
| 147 | RAP1GDS1 | na | 17374 | -0.222 | -0.4880 | Yes |
| 148 | EPS15 | na | 17416 | -0.225 | -0.4862 | Yes |
| 149 | PDGFB | na | 17563 | -0.234 | -0.4891 | Yes |
| 150 | ETV1 | na | 17695 | -0.244 | -0.4912 | Yes |
| 151 | THRAP3 | na | 17713 | -0.245 | -0.4879 | Yes |
| 152 | MYH11 | na | 17806 | -0.253 | -0.4880 | Yes |
| 153 | TOP1 | na | 17830 | -0.256 | -0.4849 | Yes |
| 154 | RABEP1 | na | 17851 | -0.257 | -0.4816 | Yes |
| 155 | ZMYM2 | na | 17982 | -0.268 | -0.4832 | Yes |
| 156 | FGFR1 | na | 18053 | -0.273 | -0.4819 | Yes |
| 157 | CDH1 | na | 18147 | -0.281 | -0.4816 | Yes |
| 158 | MLLT6 | na | 18188 | -0.284 | -0.4788 | Yes |
| 159 | CCND2 | na | 18282 | -0.290 | -0.4783 | Yes |
| 160 | CTNNB1 | na | 18285 | -0.290 | -0.4736 | Yes |
| 161 | PRRX1 | na | 18305 | -0.292 | -0.4697 | Yes |
| 162 | MEN1 | na | 18322 | -0.292 | -0.4657 | Yes |
| 163 | MALT1 | na | 18439 | -0.299 | -0.4661 | Yes |
| 164 | PIK3CA | na | 18513 | -0.305 | -0.4645 | Yes |
| 165 | ERCC4 | na | 18538 | -0.307 | -0.4605 | Yes |
| 166 | TSC1 | na | 18564 | -0.309 | -0.4566 | Yes |
| 167 | TFRC | na | 18581 | -0.311 | -0.4522 | Yes |
| 168 | TTL | na | 18734 | -0.324 | -0.4540 | Yes |
| 169 | CIITA | na | 18800 | -0.330 | -0.4515 | Yes |
| 170 | ERCC5 | na | 18886 | -0.339 | -0.4499 | Yes |
| 171 | NR4A3 | na | 18890 | -0.339 | -0.4445 | Yes |
| 172 | RBM15 | na | 18920 | -0.341 | -0.4402 | Yes |
| 173 | NSD1 | na | 18969 | -0.346 | -0.4367 | Yes |
| 174 | CYLD | na | 18996 | -0.348 | -0.4322 | Yes |
| 175 | MLH1 | na | 19016 | -0.350 | -0.4273 | Yes |
| 176 | FOXO3 | na | 19023 | -0.351 | -0.4218 | Yes |
| 177 | WHSC1 | na | 19028 | -0.352 | -0.4163 | Yes |
| 178 | EGFR | na | 19043 | -0.354 | -0.4111 | Yes |
| 179 | BMPR1A | na | 19148 | -0.365 | -0.4099 | Yes |
| 180 | TRIM33 | na | 19153 | -0.365 | -0.4041 | Yes |
| 181 | SS18L1 | na | 19286 | -0.376 | -0.4040 | Yes |
| 182 | MLLT10 | na | 19305 | -0.378 | -0.3986 | Yes |
| 183 | TCF3 | na | 19312 | -0.378 | -0.3927 | Yes |
| 184 | CLTC | na | 19369 | -0.383 | -0.3890 | Yes |
| 185 | PCM1 | na | 19498 | -0.397 | -0.3884 | Yes |
| 186 | BRCA1 | na | 19570 | -0.404 | -0.3850 | Yes |
| 187 | MSI2 | na | 19591 | -0.405 | -0.3793 | Yes |
| 188 | SMO | na | 19616 | -0.409 | -0.3737 | Yes |
| 189 | FGFR3 | na | 19694 | -0.416 | -0.3704 | Yes |
| 190 | ARNT | na | 19728 | -0.419 | -0.3650 | Yes |
| 191 | STIL | na | 19730 | -0.420 | -0.3582 | Yes |
| 192 | LASP1 | na | 19840 | -0.429 | -0.3562 | Yes |
| 193 | TPR | na | 19907 | -0.436 | -0.3521 | Yes |
| 194 | TET1 | na | 19925 | -0.437 | -0.3457 | Yes |
| 195 | COL1A1 | na | 19936 | -0.438 | -0.3389 | Yes |
| 196 | CCDC6 | na | 19937 | -0.438 | -0.3317 | Yes |
| 197 | PDGFRA | na | 19955 | -0.440 | -0.3253 | Yes |
| 198 | MLLT1 | na | 20000 | -0.445 | -0.3200 | Yes |
| 199 | SUFU | na | 20032 | -0.448 | -0.3141 | Yes |
| 200 | NF2 | na | 20065 | -0.451 | -0.3082 | Yes |
| 201 | ABL2 | na | 20073 | -0.452 | -0.3011 | Yes |
| 202 | NCOA4 | na | 20174 | -0.464 | -0.2981 | Yes |
| 203 | FOXO1 | na | 20223 | -0.469 | -0.2926 | Yes |
| 204 | NUP98 | na | 20273 | -0.474 | -0.2871 | Yes |
| 205 | FNBP1 | na | 20296 | -0.477 | -0.2802 | Yes |
| 206 | TCF12 | na | 20311 | -0.478 | -0.2730 | Yes |
| 207 | DDX6 | na | 20395 | -0.487 | -0.2689 | Yes |
| 208 | FANCD2 | na | 20434 | -0.494 | -0.2625 | Yes |
| 209 | PML | na | 20576 | -0.512 | -0.2606 | Yes |
| 210 | CDK6 | na | 20612 | -0.518 | -0.2537 | Yes |
| 211 | NOTCH1 | na | 20749 | -0.537 | -0.2512 | Yes |
| 212 | AFF4 | na | 20773 | -0.542 | -0.2433 | Yes |
| 213 | ATM | na | 20784 | -0.544 | -0.2349 | Yes |
| 214 | ABL1 | na | 20795 | -0.545 | -0.2264 | Yes |
| 215 | BRAF | na | 20807 | -0.547 | -0.2179 | Yes |
| 216 | REL | na | 20808 | -0.547 | -0.2089 | Yes |
| 217 | NCOA2 | na | 20947 | -0.569 | -0.2059 | Yes |
| 218 | HIP1 | na | 20961 | -0.571 | -0.1971 | Yes |
| 219 | PDGFRB | na | 20968 | -0.572 | -0.1880 | Yes |
| 220 | NUP214 | na | 21004 | -0.580 | -0.1801 | Yes |
| 221 | ERC1 | na | 21041 | -0.586 | -0.1721 | Yes |
| 222 | MLLT4 | na | 21105 | -0.599 | -0.1652 | Yes |
| 223 | BCR | na | 21121 | -0.601 | -0.1560 | Yes |
| 224 | APC | na | 21200 | -0.619 | -0.1495 | Yes |
| 225 | LPP | na | 21204 | -0.621 | -0.1394 | Yes |
| 226 | CBL | na | 21212 | -0.623 | -0.1295 | Yes |
| 227 | CNTRL | na | 21219 | -0.625 | -0.1195 | Yes |
| 228 | MYH9 | na | 21236 | -0.628 | -0.1099 | Yes |
| 229 | CREBBP | na | 21304 | -0.643 | -0.1024 | Yes |
| 230 | NUMA1 | na | 21305 | -0.643 | -0.0919 | Yes |
| 231 | PBX1 | na | 21314 | -0.646 | -0.0816 | Yes |
| 232 | CASC5 | na | 21341 | -0.654 | -0.0721 | Yes |
| 233 | ARHGEF12 | na | 21366 | -0.658 | -0.0624 | Yes |
| 234 | EP300 | na | 21416 | -0.674 | -0.0536 | Yes |
| 235 | TRIP11 | na | 21437 | -0.681 | -0.0433 | Yes |
| 236 | WRN | na | 21479 | -0.697 | -0.0338 | Yes |
| 237 | MN1 | na | 21558 | -0.729 | -0.0254 | Yes |
| 238 | PATZ1 | na | 21586 | -0.746 | -0.0144 | Yes |
| 239 | MAML2 | na | 21672 | -0.812 | -0.0049 | Yes |
| 240 | RNF213 | na | 21724 | -0.869 | 0.0070 | Yes |
Table: GSEA details [plain text format]

  

Fig 2: GRESHOCK\_CANCER\_COPY\_NUMBER\_UP      
 Blue-Pink O' Gram in the Space of the Analyzed GeneSet

  

Fig 3: GRESHOCK\_CANCER\_COPY\_NUMBER\_UP: Random ES distribution      
 Gene set null distribution of ES for **GRESHOCK\_CANCER\_COPY\_NUMBER\_UP**

  
