# Supplementary material for: Regulation of microglia related neuroinflammation contributes to the protective effect of Gelsevirine on ischemic stroke
Source: Front Immunol. 2023 Mar 30;14:1164278. doi: 10.3389/fimmu.2023.1164278 (PMC10098192; doi:10.3389/fimmu.2023.1164278)
Supplement: Supplementary file 6 [file DataSheet_6.zip › fig 5 raw/fig 5-G raw/inflammation.Gsea.1649955060129/gsea_report_for_Gs_1649955060129.html]

Report for Gs 1649955060129 [GSEA]

| GS  follow link to MSigDB | GS DETAILS | SIZE | ES | NES | NOM p-val | FDR q-val | FWER p-val | RANK AT MAX | LEADING EDGE || 1 | HALLMARK\_MYC\_TARGETS\_V1 | Details ... | 197 | 0.62 | 2.21 | 0.000 | 0.000 | 0.000 | 4188 | tags=58%, list=19%, signal=71% |
| 2 | REACTOME\_SIGNALING\_BY\_WNT | Details ... | 61 | 0.61 | 1.85 | 0.000 | 0.006 | 0.026 | 3503 | tags=61%, list=16%, signal=72% |
| 3 | JAZAG\_TGFB1\_SIGNALING\_DN | Details ... | 32 | 0.59 | 1.62 | 0.009 | 0.048 | 0.277 | 3088 | tags=41%, list=14%, signal=47% |
| 4 | HALLMARK\_MTORC1\_SIGNALING | Details ... | 193 | 0.42 | 1.50 | 0.000 | 0.099 | 0.598 | 4351 | tags=42%, list=20%, signal=53% |
| 5 | CREIGHTON\_AKT1\_SIGNALING\_VIA\_MTOR\_DN | Details ... | 22 | 0.59 | 1.46 | 0.040 | 0.107 | 0.708 | 5121 | tags=64%, list=23%, signal=83% |
| 6 | HALLMARK\_MYC\_TARGETS\_V2 | Details ... | 58 | 0.48 | 1.43 | 0.020 | 0.114 | 0.785 | 5701 | tags=67%, list=26%, signal=91% |
| 7 | SCHURINGA\_STAT5A\_TARGETS\_DN | Details ... | 15 | 0.60 | 1.33 | 0.121 | 0.187 | 0.946 | 195 | tags=13%, list=1%, signal=13% |
| 8 | NUMATA\_CSF3\_SIGNALING\_VIA\_STAT3 | Details ... | 21 | 0.50 | 1.22 | 0.151 | 0.323 | 0.994 | 969 | tags=19%, list=4%, signal=20% |
| 9 | AZARE\_NEOPLASTIC\_TRANSFORMATION\_BY\_STAT3\_DN | Details ... | 16 | 0.48 | 1.08 | 0.342 | 0.635 | 1.000 | 1272 | tags=31%, list=6%, signal=33% |
| 10 | KEGG\_PPAR\_SIGNALING\_PATHWAY | Details ... | 63 | 0.34 | 1.03 | 0.383 | 0.756 | 1.000 | 2501 | tags=24%, list=11%, signal=27% |
| 11 | DAUER\_STAT3\_TARGETS\_DN | Details ... | 41 | 0.36 | 1.02 | 0.404 | 0.713 | 1.000 | 3919 | tags=34%, list=18%, signal=42% |
| 12 | HAN\_JNK\_SINGALING\_DN | Details ... | 37 | 0.36 | 0.99 | 0.459 | 0.769 | 1.000 | 3939 | tags=35%, list=18%, signal=43% |
| 13 | JAZAG\_TGFB1\_SIGNALING\_UP | Details ... | 100 | 0.28 | 0.93 | 0.609 | 0.900 | 1.000 | 3987 | tags=32%, list=18%, signal=39% |
| 14 | JAZAG\_TGFB1\_SIGNALING\_VIA\_SMAD4\_UP | Details ... | 99 | 0.27 | 0.91 | 0.704 | 0.907 | 1.000 | 3088 | tags=21%, list=14%, signal=25% |
| 15 | SCHURINGA\_STAT5A\_TARGETS\_UP | Details ... | 17 | 0.37 | 0.88 | 0.636 | 0.943 | 1.000 | 194 | tags=12%, list=1%, signal=12% |
| 16 | WIERENGA\_STAT5A\_TARGETS\_GROUP2 | Details ... | 50 | 0.29 | 0.86 | 0.764 | 0.942 | 1.000 | 3259 | tags=24%, list=15%, signal=28% |
| 17 | PARENT\_MTOR\_SIGNALING\_DN | Details ... | 40 | 0.30 | 0.84 | 0.742 | 0.935 | 1.000 | 1004 | tags=15%, list=5%, signal=16% |
| 18 | KENNY\_CTNNB1\_TARGETS\_UP | Details ... | 47 | 0.27 | 0.81 | 0.914 | 0.958 | 1.000 | 4063 | tags=38%, list=19%, signal=47% |
| 19 | DEBOSSCHER\_NFKB\_TARGETS\_REPRESSED\_BY\_GLUCOCORTICOIDS | Details ... | 21 | 0.31 | 0.77 | 0.864 | 0.965 | 1.000 | 495 | tags=10%, list=2%, signal=10% |
| 20 | WILLERT\_WNT\_SIGNALING | Details ... | 20 | 0.29 | 0.71 | 0.939 | 0.965 | 1.000 | 1357 | tags=15%, list=6%, signal=16% |
Table: Gene sets enriched in phenotype **Gs (3 samples)**[plain text format]****

  
