# Supplementary material for: Regulation of microglia related neuroinflammation contributes to the protective effect of Gelsevirine on ischemic stroke
Source: Front Immunol. 2023 Mar 30;14:1164278. doi: 10.3389/fimmu.2023.1164278 (PMC10098192; doi:10.3389/fimmu.2023.1164278)
Supplement: Supplementary file 6 [file DataSheet_6.zip › fig 5 raw/fig 5-G raw/inflammation.Gsea.1649955060129/gsea_report_for_MCAO_1649955060129.html]

Report for MCAO 1649955060129 [GSEA]

| GS  follow link to MSigDB | GS DETAILS | SIZE | ES | NES | NOM p-val | FDR q-val | FWER p-val | RANK AT MAX | LEADING EDGE || 1 | PID\_FAK\_PATHWAY | Details ... | 58 | -0.66 | -1.84 | 0.000 | 0.010 | 0.008 | 5643 | tags=67%, list=26%, signal=90% |
| 2 | BILD\_CTNNB1\_ONCOGENIC\_SIGNATURE | Details ... | 74 | -0.60 | -1.71 | 0.000 | 0.057 | 0.087 | 4749 | tags=57%, list=22%, signal=72% |
| 3 | PID\_WNT\_CANONICAL\_PATHWAY | Details ... | 20 | -0.72 | -1.65 | 0.003 | 0.085 | 0.182 | 5704 | tags=60%, list=26%, signal=81% |
| 4 | PID\_IGF1\_PATHWAY | Details ... | 29 | -0.67 | -1.65 | 0.008 | 0.065 | 0.184 | 5237 | tags=76%, list=24%, signal=100% |
| 5 | GRESHOCK\_CANCER\_COPY\_NUMBER\_UP | Details ... | 240 | -0.51 | -1.64 | 0.000 | 0.058 | 0.204 | 5394 | tags=47%, list=25%, signal=61% |
| 6 | REACTOME\_SIGNALING\_BY\_HIPPO | Details ... | 20 | -0.72 | -1.63 | 0.008 | 0.054 | 0.227 | 4399 | tags=75%, list=20%, signal=94% |
| 7 | PID\_SHP2\_PATHWAY | Details ... | 56 | -0.59 | -1.62 | 0.000 | 0.060 | 0.278 | 4626 | tags=50%, list=21%, signal=63% |
| 8 | PID\_RHOA\_REG\_PATHWAY | Details ... | 45 | -0.60 | -1.60 | 0.003 | 0.066 | 0.343 | 4061 | tags=56%, list=19%, signal=68% |
| 9 | FIRESTEIN\_CTNNB1\_PATHWAY | Details ... | 33 | -0.64 | -1.60 | 0.011 | 0.059 | 0.345 | 5178 | tags=55%, list=24%, signal=71% |
| 10 | PHONG\_TNF\_RESPONSE\_VIA\_P38\_COMPLETE | Details ... | 212 | -0.48 | -1.57 | 0.000 | 0.074 | 0.440 | 5487 | tags=52%, list=25%, signal=69% |
| 11 | PID\_BETA\_CATENIN\_NUC\_PATHWAY | Details ... | 77 | -0.54 | -1.56 | 0.003 | 0.075 | 0.475 | 5112 | tags=42%, list=23%, signal=54% |
| 12 | KEGG\_TGF\_BETA\_SIGNALING\_PATHWAY | Details ... | 84 | -0.53 | -1.53 | 0.000 | 0.089 | 0.581 | 3860 | tags=36%, list=18%, signal=43% |
| 13 | ST\_WNT\_BETA\_CATENIN\_PATHWAY | Details ... | 34 | -0.61 | -1.52 | 0.014 | 0.098 | 0.638 | 5749 | tags=50%, list=26%, signal=68% |
| 14 | KEGG\_WNT\_SIGNALING\_PATHWAY | Details ... | 143 | -0.48 | -1.52 | 0.000 | 0.091 | 0.638 | 5199 | tags=40%, list=24%, signal=52% |
| 15 | BIOCARTA\_GSK3\_PATHWAY | Details ... | 27 | -0.63 | -1.51 | 0.016 | 0.092 | 0.673 | 5104 | tags=67%, list=23%, signal=87% |
| 16 | HALLMARK\_WNT\_BETA\_CATENIN\_SIGNALING | Details ... | 41 | -0.58 | -1.50 | 0.008 | 0.096 | 0.703 | 5104 | tags=51%, list=23%, signal=67% |
| 17 | BIOCARTA\_ERK\_PATHWAY | Details ... | 28 | -0.62 | -1.50 | 0.016 | 0.094 | 0.721 | 3525 | tags=43%, list=16%, signal=51% |
| 18 | KEGG\_TIGHT\_JUNCTION | Details ... | 130 | -0.49 | -1.49 | 0.004 | 0.097 | 0.756 | 4660 | tags=38%, list=21%, signal=49% |
| 19 | BIOCARTA\_EGF\_PATHWAY | Details ... | 31 | -0.60 | -1.49 | 0.027 | 0.092 | 0.759 | 5573 | tags=68%, list=25%, signal=91% |
| 20 | ST\_JNK\_MAPK\_PATHWAY | Details ... | 39 | -0.57 | -1.49 | 0.017 | 0.090 | 0.770 | 5050 | tags=62%, list=23%, signal=80% |
| 21 | REACTOME\_PI3K\_AKT\_ACTIVATION |  | 36 | -0.59 | -1.48 | 0.018 | 0.098 | 0.807 | 5093 | tags=64%, list=23%, signal=83% |
| 22 | SA\_PTEN\_PATHWAY |  | 17 | -0.69 | -1.47 | 0.040 | 0.095 | 0.814 | 5643 | tags=71%, list=26%, signal=95% |
| 23 | REACTOME\_ERK\_MAPK\_TARGETS |  | 21 | -0.64 | -1.46 | 0.039 | 0.106 | 0.854 | 4200 | tags=52%, list=19%, signal=65% |
| 24 | PID\_RHOA\_PATHWAY |  | 45 | -0.55 | -1.46 | 0.016 | 0.103 | 0.862 | 5216 | tags=60%, list=24%, signal=79% |
| 25 | KOINUMA\_TARGETS\_OF\_SMAD2\_OR\_SMAD3 |  | 234 | -0.44 | -1.46 | 0.000 | 0.101 | 0.864 | 5394 | tags=47%, list=25%, signal=62% |
| 26 | BHAT\_ESR1\_TARGETS\_VIA\_AKT1\_DN |  | 76 | -0.51 | -1.45 | 0.015 | 0.102 | 0.880 | 4346 | tags=46%, list=20%, signal=57% |
| 27 | BIOCARTA\_NFKB\_PATHWAY |  | 23 | -0.63 | -1.45 | 0.041 | 0.101 | 0.884 | 6293 | tags=65%, list=29%, signal=91% |
| 28 | WNT\_SIGNALING |  | 88 | -0.49 | -1.44 | 0.015 | 0.109 | 0.913 | 5234 | tags=39%, list=24%, signal=51% |
| 29 | REACTOME\_PI3K\_EVENTS\_IN\_ERBB2\_SIGNALING |  | 42 | -0.55 | -1.44 | 0.027 | 0.105 | 0.914 | 5093 | tags=62%, list=23%, signal=81% |
| 30 | PID\_WNT\_SIGNALING\_PATHWAY |  | 28 | -0.59 | -1.43 | 0.047 | 0.111 | 0.939 | 2336 | tags=29%, list=11%, signal=32% |
| 31 | REACTOME\_SIGNALLING\_BY\_NGF |  | 211 | -0.44 | -1.42 | 0.005 | 0.112 | 0.946 | 5093 | tags=45%, list=23%, signal=59% |
| 32 | PHONG\_TNF\_RESPONSE\_VIA\_P38\_PARTIAL |  | 152 | -0.45 | -1.42 | 0.008 | 0.112 | 0.949 | 4547 | tags=37%, list=21%, signal=46% |
| 33 | KEGG\_JAK\_STAT\_SIGNALING\_PATHWAY |  | 138 | -0.46 | -1.41 | 0.004 | 0.113 | 0.953 | 5093 | tags=36%, list=23%, signal=47% |
| 34 | BIOCARTA\_MAPK\_PATHWAY |  | 86 | -0.48 | -1.40 | 0.024 | 0.117 | 0.964 | 6046 | tags=57%, list=28%, signal=78% |
| 35 | KEGG\_MAPK\_SIGNALING\_PATHWAY |  | 249 | -0.43 | -1.40 | 0.000 | 0.117 | 0.965 | 5146 | tags=40%, list=24%, signal=52% |
| 36 | BREDEMEYER\_RAG\_SIGNALING\_VIA\_ATM\_NOT\_VIA\_NFKB\_DN |  | 35 | -0.55 | -1.39 | 0.057 | 0.122 | 0.972 | 4977 | tags=60%, list=23%, signal=78% |
| 37 | PHONG\_TNF\_RESPONSE\_NOT\_VIA\_P38 |  | 233 | -0.43 | -1.39 | 0.001 | 0.119 | 0.972 | 5686 | tags=44%, list=26%, signal=59% |
| 38 | PID\_SMAD2\_3PATHWAY |  | 17 | -0.64 | -1.39 | 0.060 | 0.120 | 0.973 | 5990 | tags=71%, list=27%, signal=97% |
| 39 | BIOCARTA\_IL6\_PATHWAY |  | 21 | -0.61 | -1.39 | 0.058 | 0.118 | 0.975 | 2768 | tags=48%, list=13%, signal=54% |
| 40 | KEGG\_NOTCH\_SIGNALING\_PATHWAY |  | 47 | -0.52 | -1.38 | 0.037 | 0.128 | 0.981 | 5462 | tags=53%, list=25%, signal=71% |
| 41 | REACTOME\_SIGNALING\_BY\_EGFR\_IN\_CANCER |  | 104 | -0.46 | -1.37 | 0.022 | 0.132 | 0.988 | 5093 | tags=49%, list=23%, signal=64% |
| 42 | PID\_PI3KCI\_AKT\_PATHWAY |  | 35 | -0.53 | -1.36 | 0.076 | 0.136 | 0.992 | 5093 | tags=57%, list=23%, signal=74% |
| 43 | REACTOME\_PI\_3K\_CASCADE |  | 53 | -0.50 | -1.35 | 0.048 | 0.144 | 0.996 | 5093 | tags=47%, list=23%, signal=61% |
| 44 | PID\_PI3K\_PLC\_TRK\_PATHWAY |  | 36 | -0.53 | -1.35 | 0.085 | 0.143 | 0.996 | 3651 | tags=39%, list=17%, signal=47% |
| 45 | PID\_PI3KPLCTRKPATHWAY |  | 36 | -0.53 | -1.35 | 0.094 | 0.145 | 0.999 | 3651 | tags=39%, list=17%, signal=47% |
| 46 | REACTOME\_PIP3\_ACTIVATES\_AKT\_SIGNALING |  | 27 | -0.55 | -1.34 | 0.088 | 0.149 | 0.999 | 5093 | tags=63%, list=23%, signal=82% |
| 47 | REACTOME\_SIGNALING\_BY\_NOTCH1 |  | 66 | -0.47 | -1.32 | 0.053 | 0.163 | 1.000 | 3697 | tags=38%, list=17%, signal=45% |
| 48 | DAUER\_STAT3\_TARGETS\_UP |  | 44 | -0.50 | -1.32 | 0.079 | 0.165 | 1.000 | 2741 | tags=34%, list=13%, signal=39% |
| 49 | PID\_TGFBRPATHWAY |  | 55 | -0.48 | -1.31 | 0.075 | 0.177 | 1.000 | 4815 | tags=55%, list=22%, signal=70% |
| 50 | AZARE\_STAT3\_TARGETS |  | 22 | -0.57 | -1.31 | 0.115 | 0.175 | 1.000 | 4379 | tags=50%, list=20%, signal=62% |
| 51 | KEGG\_HEDGEHOG\_SIGNALING\_PATHWAY |  | 54 | -0.47 | -1.30 | 0.078 | 0.177 | 1.000 | 5234 | tags=35%, list=24%, signal=46% |
| 52 | REACTOME\_CTNNB1\_PHOSPHORYLATION\_CASCADE |  | 15 | -0.62 | -1.30 | 0.139 | 0.178 | 1.000 | 5199 | tags=60%, list=24%, signal=79% |
| 53 | KEGG\_MTOR\_SIGNALING\_PATHWAY |  | 50 | -0.47 | -1.29 | 0.105 | 0.181 | 1.000 | 5230 | tags=54%, list=24%, signal=71% |
| 54 | REACTOME\_PI3K\_EVENTS\_IN\_ERBB4\_SIGNALING |  | 36 | -0.50 | -1.29 | 0.108 | 0.179 | 1.000 | 5093 | tags=58%, list=23%, signal=76% |
| 55 | ST\_ERK1\_ERK2\_MAPK\_PATHWAY |  | 32 | -0.51 | -1.27 | 0.117 | 0.212 | 1.000 | 3908 | tags=41%, list=18%, signal=49% |
| 56 | PARENT\_MTOR\_SIGNALING\_UP |  | 228 | -0.39 | -1.27 | 0.024 | 0.210 | 1.000 | 4135 | tags=34%, list=19%, signal=41% |
| 57 | BIOCARTA\_AKT\_PATHWAY |  | 20 | -0.56 | -1.27 | 0.152 | 0.207 | 1.000 | 5087 | tags=60%, list=23%, signal=78% |
| 58 | ONDER\_CDH1\_SIGNALING\_VIA\_CTNNB1 |  | 77 | -0.44 | -1.26 | 0.072 | 0.213 | 1.000 | 4813 | tags=39%, list=22%, signal=50% |
| 59 | SANSOM\_WNT\_PATHWAY\_REQUIRE\_MYC |  | 57 | -0.45 | -1.26 | 0.094 | 0.209 | 1.000 | 3595 | tags=23%, list=16%, signal=27% |
| 60 | PID\_IL6\_7PATHWAY |  | 46 | -0.47 | -1.25 | 0.126 | 0.224 | 1.000 | 5555 | tags=48%, list=25%, signal=64% |
| 61 | JAZAG\_TGFB1\_SIGNALING\_VIA\_SMAD4\_DN |  | 58 | -0.44 | -1.24 | 0.111 | 0.230 | 1.000 | 5448 | tags=40%, list=25%, signal=53% |
| 62 | BIOCARTA\_PPARA\_PATHWAY |  | 55 | -0.45 | -1.23 | 0.130 | 0.244 | 1.000 | 2799 | tags=31%, list=13%, signal=35% |
| 63 | REACTOME\_TOLL\_RECEPTOR\_CASCADES |  | 109 | -0.41 | -1.22 | 0.089 | 0.250 | 1.000 | 5694 | tags=46%, list=26%, signal=62% |
| 64 | BIOCARTA\_IGF1\_PATHWAY |  | 21 | -0.54 | -1.22 | 0.201 | 0.256 | 1.000 | 4626 | tags=57%, list=21%, signal=72% |
| 65 | REACTOME\_P130CAS\_LINKAGE\_TO\_MAPK\_SIGNALING\_FOR\_INTEGRINS |  | 15 | -0.57 | -1.21 | 0.186 | 0.255 | 1.000 | 5625 | tags=67%, list=26%, signal=90% |
| 66 | WIERENGA\_STAT5A\_TARGETS\_GROUP1 |  | 117 | -0.40 | -1.21 | 0.105 | 0.255 | 1.000 | 4913 | tags=40%, list=22%, signal=52% |
| 67 | BIOCARTA\_MTOR\_PATHWAY |  | 23 | -0.52 | -1.20 | 0.196 | 0.265 | 1.000 | 5050 | tags=61%, list=23%, signal=79% |
| 68 | PID\_MTOR\_4PATHWAY |  | 69 | -0.42 | -1.18 | 0.188 | 0.311 | 1.000 | 6131 | tags=55%, list=28%, signal=76% |
| 69 | REACTOME\_JNK\_C\_JUN\_KINASES\_PHOSPHORYLATION\_AND\_ACTIVATION\_MEDIATED\_BY\_ACTIVATED\_HUMAN\_TAK1 |  | 16 | -0.54 | -1.17 | 0.258 | 0.318 | 1.000 | 6068 | tags=63%, list=28%, signal=86% |
| 70 | WIERENGA\_STAT5A\_TARGETS\_DN |  | 178 | -0.36 | -1.14 | 0.136 | 0.363 | 1.000 | 5207 | tags=35%, list=24%, signal=45% |
| 71 | REACTOME\_PROLONGED\_ERK\_ACTIVATION\_EVENTS |  | 19 | -0.51 | -1.13 | 0.286 | 0.389 | 1.000 | 1732 | tags=26%, list=8%, signal=29% |
| 72 | WIERENGA\_STAT5A\_TARGETS\_UP |  | 187 | -0.35 | -1.13 | 0.186 | 0.386 | 1.000 | 4913 | tags=35%, list=22%, signal=44% |
| 73 | PID\_P38\_MK2PATHWAY |  | 21 | -0.48 | -1.11 | 0.321 | 0.414 | 1.000 | 5573 | tags=48%, list=25%, signal=64% |
| 74 | REACTOME\_NFKB\_AND\_MAP\_KINASES\_ACTIVATION\_MEDIATED\_BY\_TLR4\_SIGNALING\_REPERTOIRE |  | 69 | -0.39 | -1.11 | 0.250 | 0.409 | 1.000 | 5694 | tags=48%, list=26%, signal=64% |
| 75 | PID\_P38\_MK2\_PATHWAY |  | 21 | -0.48 | -1.11 | 0.293 | 0.413 | 1.000 | 5573 | tags=48%, list=25%, signal=64% |
| 76 | BIOCARTA\_IGF1MTOR\_PATHWAY |  | 19 | -0.49 | -1.11 | 0.316 | 0.415 | 1.000 | 5050 | tags=63%, list=23%, signal=82% |
| 77 | AZARE\_NEOPLASTIC\_TRANSFORMATION\_BY\_STAT3\_UP |  | 103 | -0.36 | -1.09 | 0.261 | 0.441 | 1.000 | 4080 | tags=34%, list=19%, signal=42% |
| 78 | HAN\_JNK\_SINGALING\_UP |  | 34 | -0.43 | -1.09 | 0.321 | 0.439 | 1.000 | 5634 | tags=53%, list=26%, signal=71% |
| 79 | BIOCARTA\_HIF\_PATHWAY |  | 15 | -0.51 | -1.08 | 0.336 | 0.448 | 1.000 | 3651 | tags=33%, list=17%, signal=40% |
| 80 | REACTOME\_TRAF6\_MEDIATED\_NFKB\_ACTIVATION |  | 20 | -0.48 | -1.08 | 0.312 | 0.444 | 1.000 | 5096 | tags=50%, list=23%, signal=65% |
| 81 | KEGG\_TOLL\_LIKE\_RECEPTOR\_SIGNALING\_PATHWAY |  | 96 | -0.36 | -1.06 | 0.329 | 0.479 | 1.000 | 6068 | tags=44%, list=28%, signal=60% |
| 82 | MANTOVANI\_NFKB\_TARGETS\_UP |  | 41 | -0.40 | -1.06 | 0.344 | 0.478 | 1.000 | 4427 | tags=32%, list=20%, signal=40% |
| 83 | TIAN\_TNF\_SIGNALING\_VIA\_NFKB |  | 27 | -0.44 | -1.06 | 0.368 | 0.481 | 1.000 | 6742 | tags=59%, list=31%, signal=86% |
| 84 | PID\_PI3KCI\_PATHWAY |  | 49 | -0.39 | -1.04 | 0.356 | 0.510 | 1.000 | 3680 | tags=31%, list=17%, signal=37% |
| 85 | HINATA\_NFKB\_TARGETS\_KERATINOCYTE\_DN |  | 20 | -0.45 | -1.03 | 0.398 | 0.537 | 1.000 | 5040 | tags=35%, list=23%, signal=45% |
| 86 | KENNY\_CTNNB1\_TARGETS\_DN |  | 51 | -0.37 | -1.00 | 0.446 | 0.608 | 1.000 | 3332 | tags=29%, list=15%, signal=35% |
| 87 | ST\_P38\_MAPK\_PATHWAY |  | 37 | -0.39 | -0.99 | 0.468 | 0.615 | 1.000 | 5915 | tags=51%, list=27%, signal=70% |
| 88 | PID\_AP1\_PATHWAY |  | 65 | -0.34 | -0.97 | 0.518 | 0.675 | 1.000 | 4681 | tags=34%, list=21%, signal=43% |
| 89 | BHAT\_ESR1\_TARGETS\_VIA\_AKT1\_UP |  | 234 | -0.30 | -0.96 | 0.581 | 0.678 | 1.000 | 5656 | tags=36%, list=26%, signal=48% |
| 90 | KEGG\_P53\_SIGNALING\_PATHWAY |  | 61 | -0.34 | -0.96 | 0.547 | 0.686 | 1.000 | 4535 | tags=31%, list=21%, signal=39% |
| 91 | PID\_P38\_ALPHA\_BETA\_PATHWAY |  | 31 | -0.39 | -0.95 | 0.545 | 0.681 | 1.000 | 5555 | tags=39%, list=25%, signal=52% |
| 92 | BREDEMEYER\_RAG\_SIGNALING\_VIA\_ATM\_NOT\_VIA\_NFKB\_UP |  | 44 | -0.36 | -0.95 | 0.551 | 0.685 | 1.000 | 2261 | tags=23%, list=10%, signal=25% |
| 93 | REACTOME\_SMAD2\_SMAD3\_SMAD4\_HETEROTRIMER\_REGULATES\_TRANSCRIPTION |  | 25 | -0.39 | -0.95 | 0.553 | 0.690 | 1.000 | 5282 | tags=44%, list=24%, signal=58% |
| 94 | HINATA\_NFKB\_TARGETS\_FIBROBLAST\_UP |  | 80 | -0.32 | -0.94 | 0.594 | 0.704 | 1.000 | 3027 | tags=24%, list=14%, signal=27% |
| 95 | HALLMARK\_TNFA\_SIGNALING\_VIA\_NFKB |  | 194 | -0.29 | -0.92 | 0.665 | 0.724 | 1.000 | 4456 | tags=27%, list=20%, signal=33% |
| 96 | KEGG\_VEGF\_SIGNALING\_PATHWAY |  | 73 | -0.32 | -0.92 | 0.615 | 0.719 | 1.000 | 5643 | tags=41%, list=26%, signal=55% |
| 97 | FEVR\_CTNNB1\_TARGETS\_UP |  | 229 | -0.28 | -0.91 | 0.739 | 0.752 | 1.000 | 5092 | tags=32%, list=23%, signal=41% |
| 98 | JAIN\_NFKB\_SIGNALING |  | 68 | -0.32 | -0.90 | 0.654 | 0.755 | 1.000 | 4616 | tags=32%, list=21%, signal=41% |
| 99 | PID\_P38\_MKK3\_6PATHWAY |  | 25 | -0.38 | -0.89 | 0.634 | 0.773 | 1.000 | 4798 | tags=44%, list=22%, signal=56% |
| 100 | BIOCARTA\_P38MAPK\_PATHWAY |  | 39 | -0.34 | -0.88 | 0.703 | 0.800 | 1.000 | 4973 | tags=41%, list=23%, signal=53% |
| 101 | WANG\_NFKB\_TARGETS |  | 24 | -0.37 | -0.87 | 0.680 | 0.819 | 1.000 | 3165 | tags=29%, list=14%, signal=34% |
| 102 | HALLMARK\_IL6\_JAK\_STAT3\_SIGNALING |  | 83 | -0.28 | -0.83 | 0.849 | 0.897 | 1.000 | 5172 | tags=30%, list=24%, signal=39% |
| 103 | CREIGHTON\_AKT1\_SIGNALING\_VIA\_MTOR\_UP |  | 33 | -0.32 | -0.82 | 0.780 | 0.895 | 1.000 | 5941 | tags=42%, list=27%, signal=58% |
| 104 | REACTOME\_SIGNALLING\_TO\_ERKS |  | 36 | -0.32 | -0.82 | 0.788 | 0.901 | 1.000 | 1732 | tags=19%, list=8%, signal=21% |
| 105 | PID\_HIF2PATHWAY |  | 34 | -0.32 | -0.81 | 0.781 | 0.908 | 1.000 | 2914 | tags=24%, list=13%, signal=27% |
| 106 | BIOCARTA\_VEGF\_PATHWAY |  | 28 | -0.33 | -0.80 | 0.796 | 0.917 | 1.000 | 3656 | tags=32%, list=17%, signal=39% |
| 107 | HALLMARK\_PI3K\_AKT\_MTOR\_SIGNALING |  | 104 | -0.26 | -0.77 | 0.944 | 0.948 | 1.000 | 4798 | tags=28%, list=22%, signal=36% |
| 108 | HINATA\_NFKB\_TARGETS\_KERATINOCYTE\_UP |  | 84 | -0.26 | -0.77 | 0.939 | 0.945 | 1.000 | 4571 | tags=27%, list=21%, signal=34% |
| 109 | FEVR\_CTNNB1\_TARGETS\_DN |  | 235 | -0.23 | -0.76 | 0.999 | 0.946 | 1.000 | 5709 | tags=33%, list=26%, signal=44% |
| 110 | REACTOME\_TGF\_BETA\_RECEPTOR\_SIGNALING\_ACTIVATES\_SMADS |  | 24 | -0.31 | -0.73 | 0.917 | 0.976 | 1.000 | 3243 | tags=33%, list=15%, signal=39% |
| 111 | SCHOEN\_NFKB\_SIGNALING |  | 32 | -0.28 | -0.70 | 0.948 | 0.988 | 1.000 | 470 | tags=9%, list=2%, signal=10% |
| 112 | BAKER\_HEMATOPOIESIS\_STAT3\_TARGETS |  | 16 | -0.32 | -0.68 | 0.921 | 0.989 | 1.000 | 5717 | tags=50%, list=26%, signal=68% |
| 113 | RASHI\_NFKB1\_TARGETS |  | 18 | -0.28 | -0.62 | 0.980 | 1.000 | 1.000 | 4656 | tags=33%, list=21%, signal=42% |
| 114 | TIAN\_TNF\_SIGNALING\_NOT\_VIA\_NFKB |  | 21 | -0.25 | -0.57 | 0.997 | 0.998 | 1.000 | 5686 | tags=38%, list=26%, signal=51% |
Table: Gene sets enriched in phenotype **MCAO (3 samples)**[plain text format]****

  
