# Supplementary figures and images for: Regulation of microglia related neuroinflammation contributes to the protective effect of Gelsevirine on ischemic stroke (part 2 of 2)
Source: Front Immunol. 2023 Mar 30;14:1164278. doi: 10.3389/fimmu.2023.1164278 (PMC10098192; doi:10.3389/fimmu.2023.1164278)

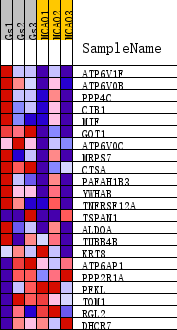

Supplement: Supplementary file 6 [file DataSheet_6.zip › fig 5 raw/fig 5-G raw/inflammation.Gsea.1649955060129/CREIGHTON_AKT1_SIGNALING_VIA_MTOR_DN_182.png]

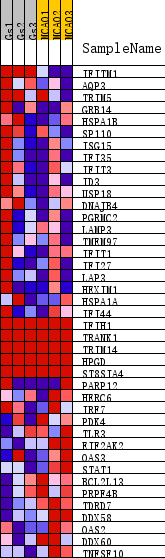

Supplement: Supplementary file 6 [file DataSheet_6.zip › fig 5 raw/fig 5-G raw/inflammation.Gsea.1649955060129/DAUER_STAT3_TARGETS_DN_200.png]

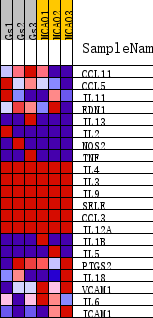

Supplement: Supplementary file 6 [file DataSheet_6.zip › fig 5 raw/fig 5-G raw/inflammation.Gsea.1649955060129/DEBOSSCHER_NFKB_TARGETS_REPRESSED_BY_GLUCOCORTICOIDS_224.png]

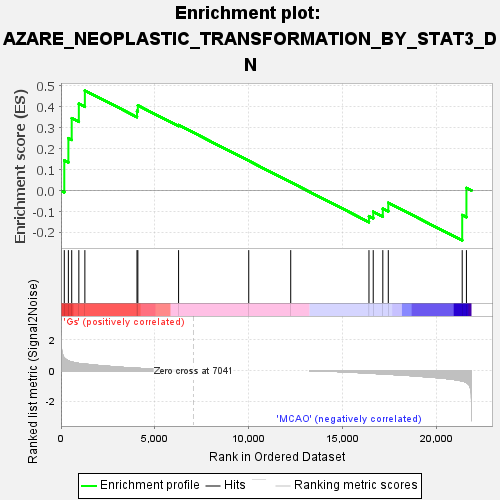

Supplement: Supplementary file 6 [file DataSheet_6.zip › fig 5 raw/fig 5-G raw/inflammation.Gsea.1649955060129/enplot_AZARE_NEOPLASTIC_TRANSFORMATION_BY_STAT3_DN_193.png]

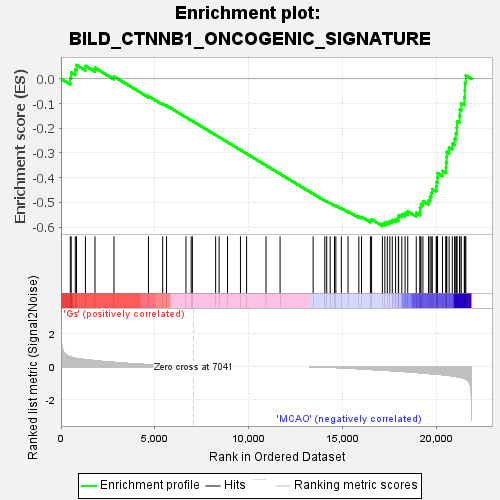

Supplement: Supplementary file 6 [file DataSheet_6.zip › fig 5 raw/fig 5-G raw/inflammation.Gsea.1649955060129/enplot_BILD_CTNNB1_ONCOGENIC_SIGNATURE_232.png]

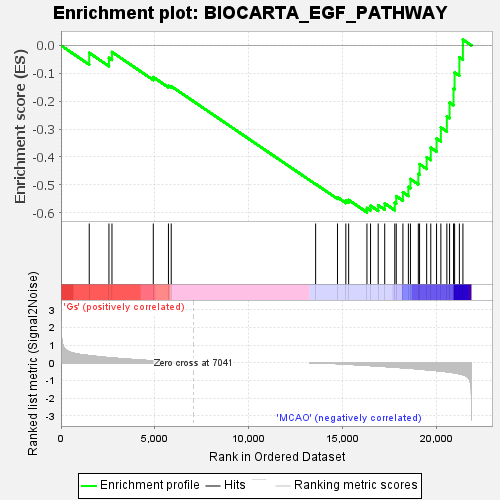

Supplement: Supplementary file 6 [file DataSheet_6.zip › fig 5 raw/fig 5-G raw/inflammation.Gsea.1649955060129/enplot_BIOCARTA_EGF_PATHWAY_283.png]

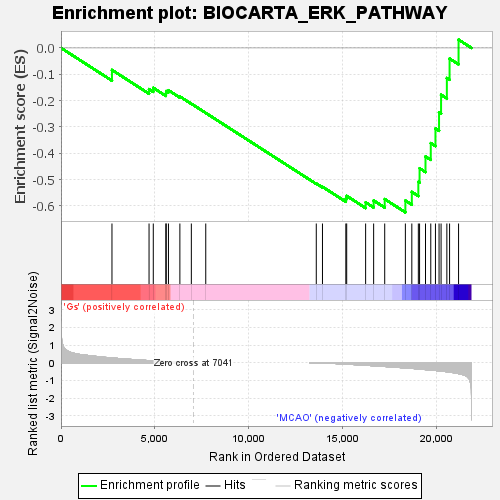

Supplement: Supplementary file 6 [file DataSheet_6.zip › fig 5 raw/fig 5-G raw/inflammation.Gsea.1649955060129/enplot_BIOCARTA_ERK_PATHWAY_277.png]

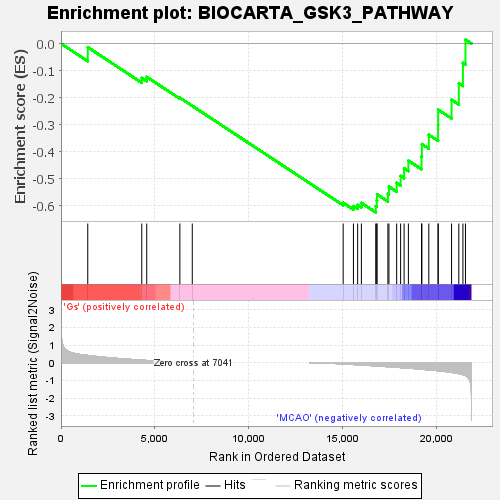

Supplement: Supplementary file 6 [file DataSheet_6.zip › fig 5 raw/fig 5-G raw/inflammation.Gsea.1649955060129/enplot_BIOCARTA_GSK3_PATHWAY_271.png]

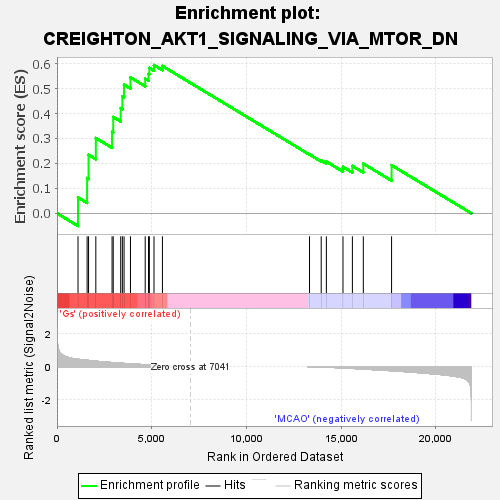

Supplement: Supplementary file 6 [file DataSheet_6.zip › fig 5 raw/fig 5-G raw/inflammation.Gsea.1649955060129/enplot_CREIGHTON_AKT1_SIGNALING_VIA_MTOR_DN_181.png]

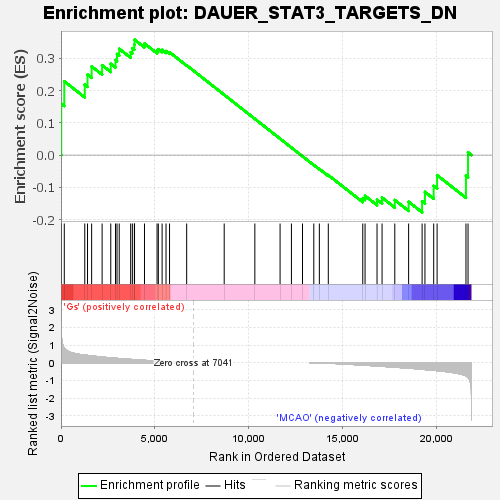

Supplement: Supplementary file 6 [file DataSheet_6.zip › fig 5 raw/fig 5-G raw/inflammation.Gsea.1649955060129/enplot_DAUER_STAT3_TARGETS_DN_199.png]

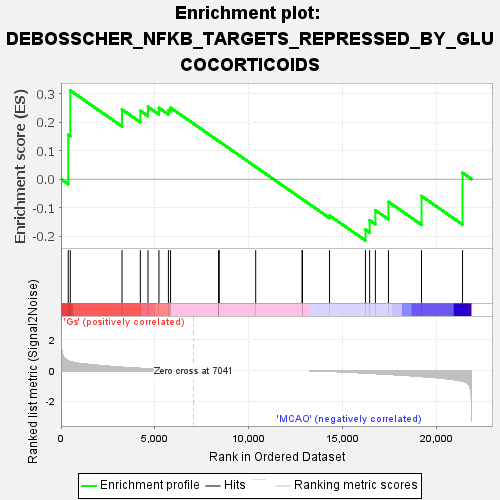

Supplement: Supplementary file 6 [file DataSheet_6.zip › fig 5 raw/fig 5-G raw/inflammation.Gsea.1649955060129/enplot_DEBOSSCHER_NFKB_TARGETS_REPRESSED_BY_GLUCOCORTICOIDS_223.png]

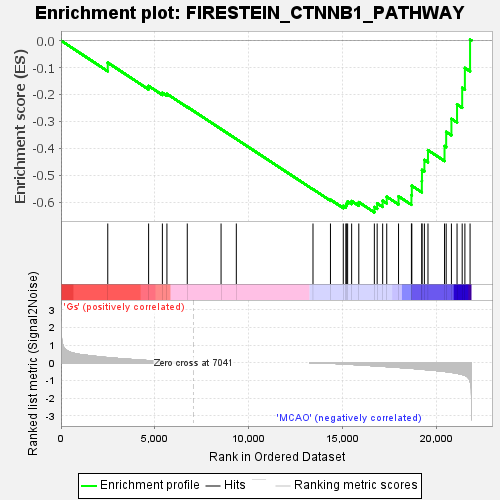

Supplement: Supplementary file 6 [file DataSheet_6.zip › fig 5 raw/fig 5-G raw/inflammation.Gsea.1649955060129/enplot_FIRESTEIN_CTNNB1_PATHWAY_253.png]

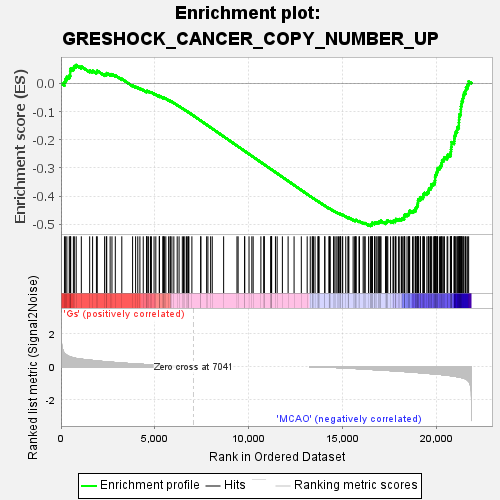

Supplement: Supplementary file 6 [file DataSheet_6.zip › fig 5 raw/fig 5-G raw/inflammation.Gsea.1649955060129/enplot_GRESHOCK_CANCER_COPY_NUMBER_UP_241.png]

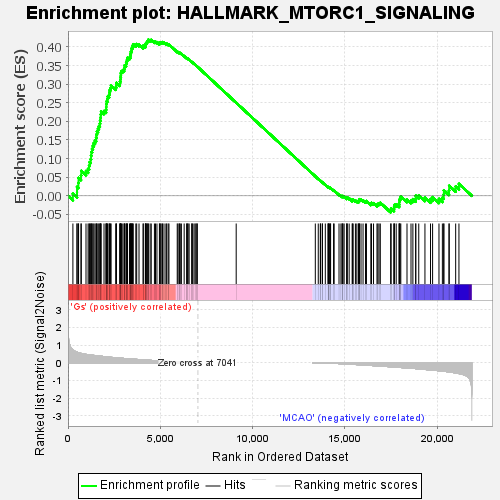

Supplement: Supplementary file 6 [file DataSheet_6.zip › fig 5 raw/fig 5-G raw/inflammation.Gsea.1649955060129/enplot_HALLMARK_MTORC1_SIGNALING_178.png]

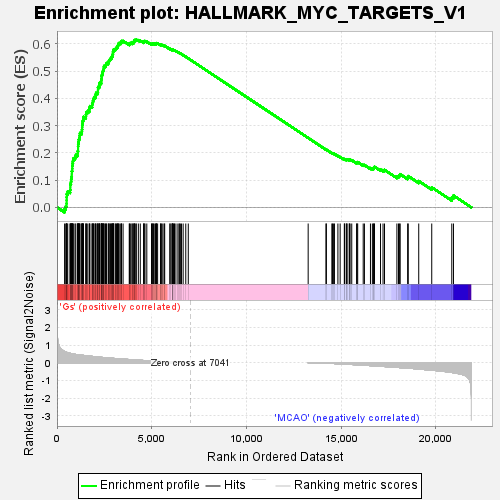

Supplement: Supplementary file 6 [file DataSheet_6.zip › fig 5 raw/fig 5-G raw/inflammation.Gsea.1649955060129/enplot_HALLMARK_MYC_TARGETS_V1_169.png]

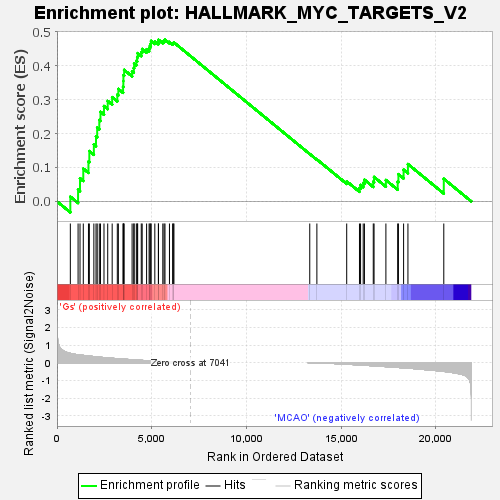

Supplement: Supplementary file 6 [file DataSheet_6.zip › fig 5 raw/fig 5-G raw/inflammation.Gsea.1649955060129/enplot_HALLMARK_MYC_TARGETS_V2_184.png]

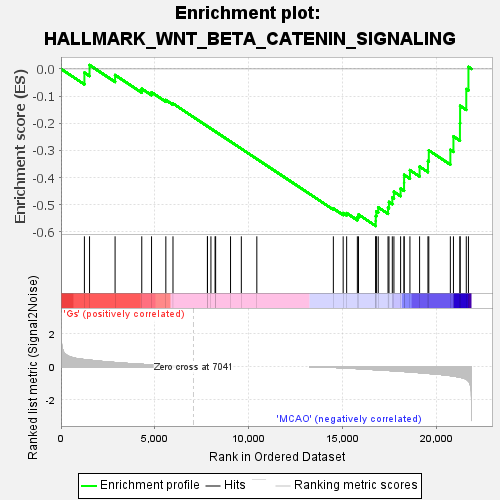

Supplement: Supplementary file 6 [file DataSheet_6.zip › fig 5 raw/fig 5-G raw/inflammation.Gsea.1649955060129/enplot_HALLMARK_WNT_BETA_CATENIN_SIGNALING_274.png]

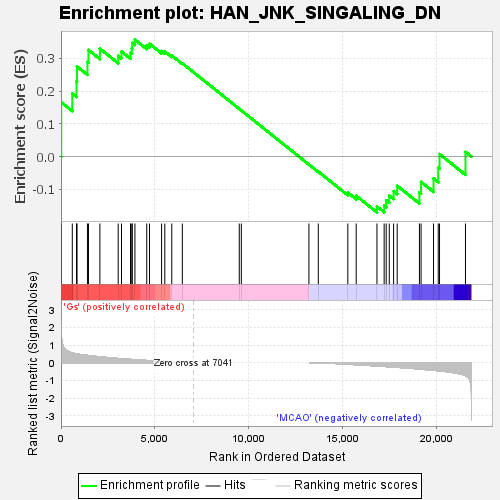

Supplement: Supplementary file 6 [file DataSheet_6.zip › fig 5 raw/fig 5-G raw/inflammation.Gsea.1649955060129/enplot_HAN_JNK_SINGALING_DN_202.png]

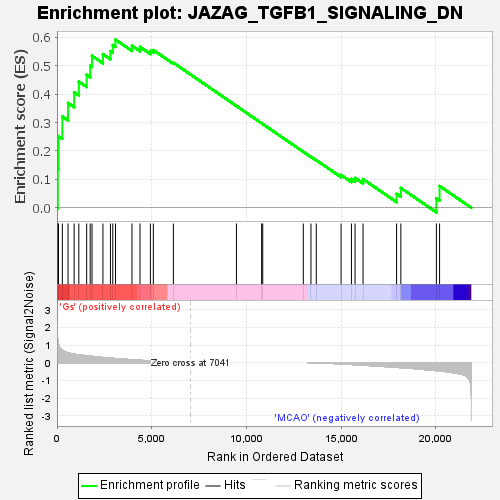

Supplement: Supplementary file 6 [file DataSheet_6.zip › fig 5 raw/fig 5-G raw/inflammation.Gsea.1649955060129/enplot_JAZAG_TGFB1_SIGNALING_DN_175.png]

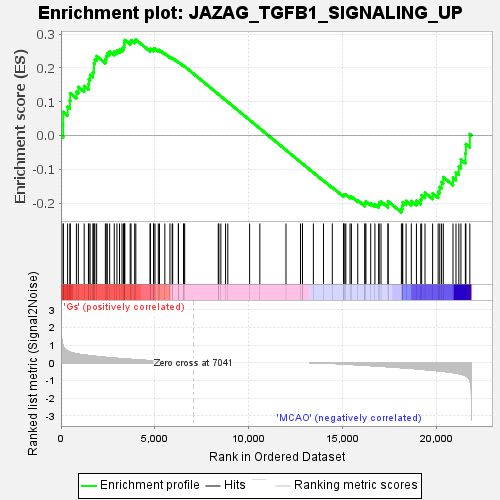

Supplement: Supplementary file 6 [file DataSheet_6.zip › fig 5 raw/fig 5-G raw/inflammation.Gsea.1649955060129/enplot_JAZAG_TGFB1_SIGNALING_UP_205.png]

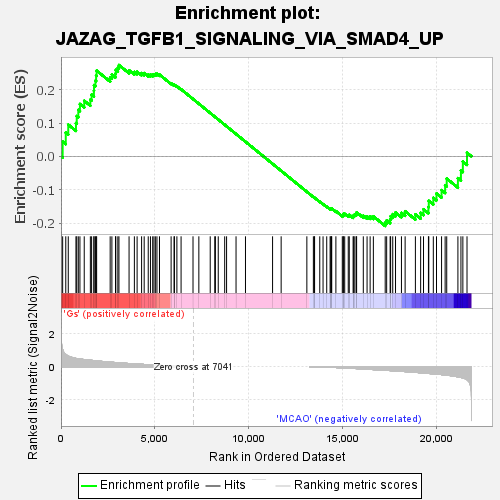

Supplement: Supplementary file 6 [file DataSheet_6.zip › fig 5 raw/fig 5-G raw/inflammation.Gsea.1649955060129/enplot_JAZAG_TGFB1_SIGNALING_VIA_SMAD4_UP_208.png]

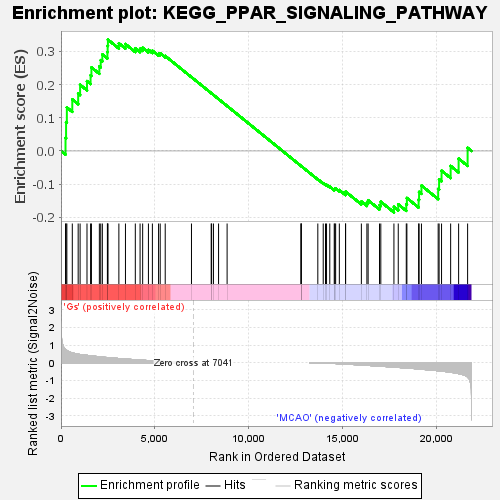

Supplement: Supplementary file 6 [file DataSheet_6.zip › fig 5 raw/fig 5-G raw/inflammation.Gsea.1649955060129/enplot_KEGG_PPAR_SIGNALING_PATHWAY_196.png]

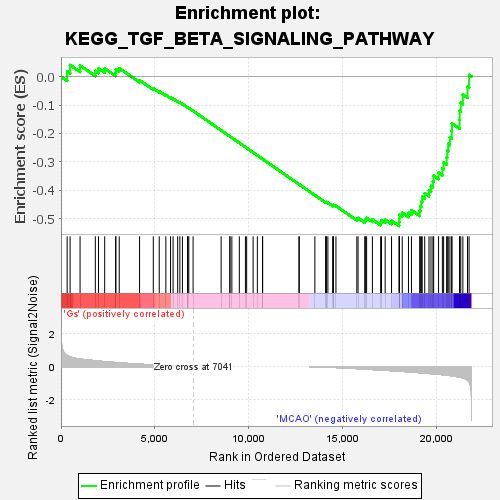

Supplement: Supplementary file 6 [file DataSheet_6.zip › fig 5 raw/fig 5-G raw/inflammation.Gsea.1649955060129/enplot_KEGG_TGF_BETA_SIGNALING_PATHWAY_262.png]

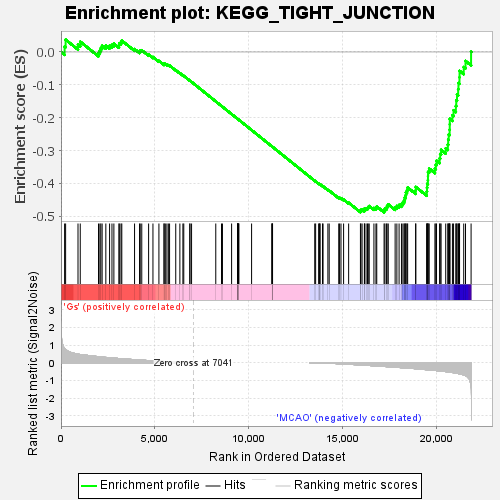

Supplement: Supplementary file 6 [file DataSheet_6.zip › fig 5 raw/fig 5-G raw/inflammation.Gsea.1649955060129/enplot_KEGG_TIGHT_JUNCTION_280.png]

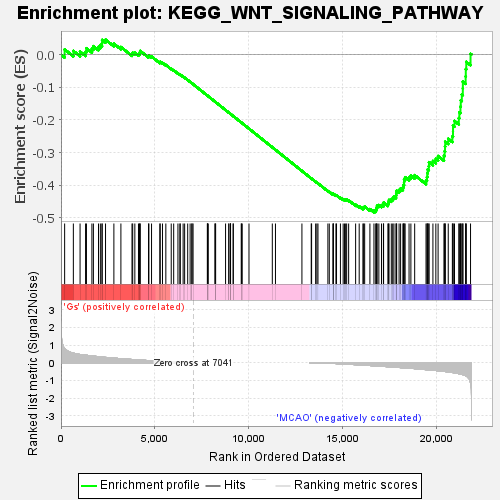

Supplement: Supplementary file 6 [file DataSheet_6.zip › fig 5 raw/fig 5-G raw/inflammation.Gsea.1649955060129/enplot_KEGG_WNT_SIGNALING_PATHWAY_268.png]

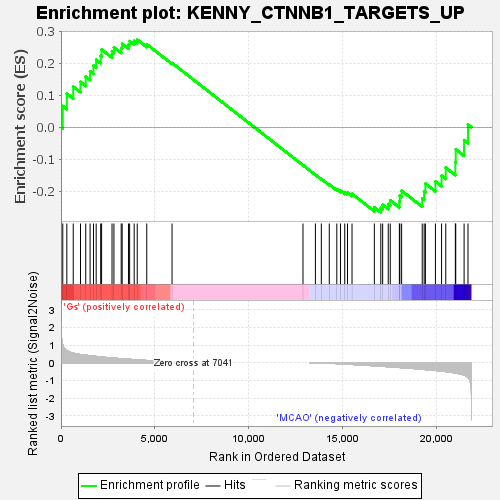

Supplement: Supplementary file 6 [file DataSheet_6.zip › fig 5 raw/fig 5-G raw/inflammation.Gsea.1649955060129/enplot_KENNY_CTNNB1_TARGETS_UP_220.png]

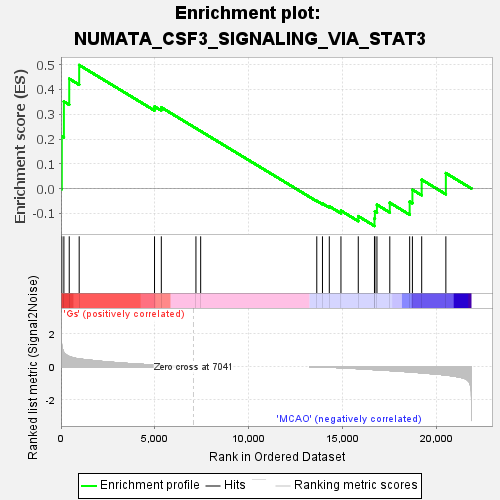

Supplement: Supplementary file 6 [file DataSheet_6.zip › fig 5 raw/fig 5-G raw/inflammation.Gsea.1649955060129/enplot_NUMATA_CSF3_SIGNALING_VIA_STAT3_190.png]

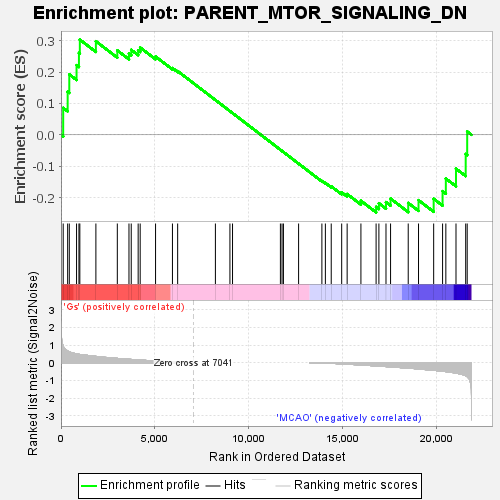

Supplement: Supplementary file 6 [file DataSheet_6.zip › fig 5 raw/fig 5-G raw/inflammation.Gsea.1649955060129/enplot_PARENT_MTOR_SIGNALING_DN_217.png]

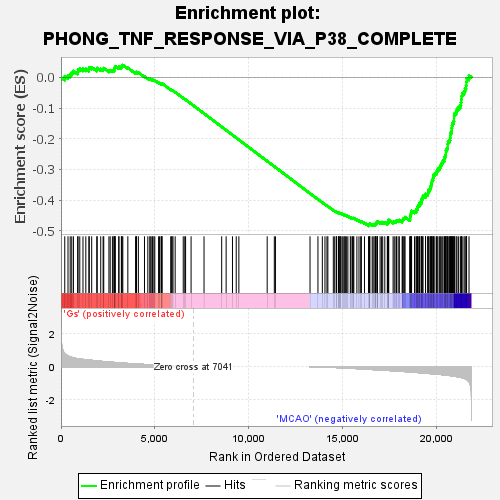

Supplement: Supplementary file 6 [file DataSheet_6.zip › fig 5 raw/fig 5-G raw/inflammation.Gsea.1649955060129/enplot_PHONG_TNF_RESPONSE_VIA_P38_COMPLETE_256.png]

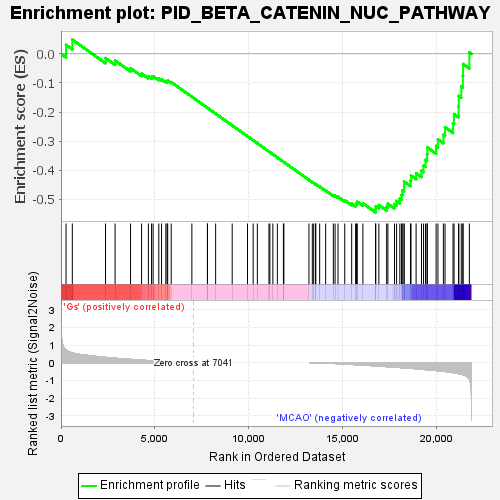

Supplement: Supplementary file 6 [file DataSheet_6.zip › fig 5 raw/fig 5-G raw/inflammation.Gsea.1649955060129/enplot_PID_BETA_CATENIN_NUC_PATHWAY_259.png]

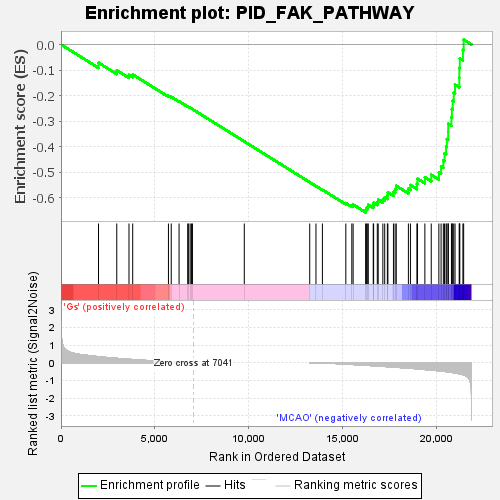

Supplement: Supplementary file 6 [file DataSheet_6.zip › fig 5 raw/fig 5-G raw/inflammation.Gsea.1649955060129/enplot_PID_FAK_PATHWAY_229.png]

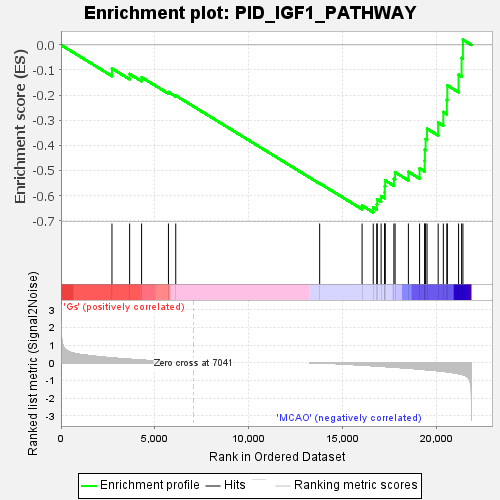

Supplement: Supplementary file 6 [file DataSheet_6.zip › fig 5 raw/fig 5-G raw/inflammation.Gsea.1649955060129/enplot_PID_IGF1_PATHWAY_238.png]

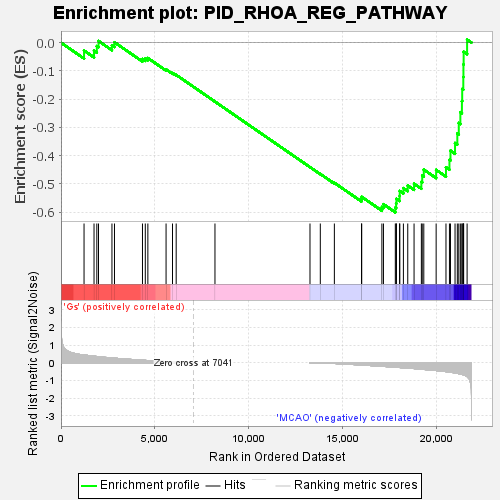

Supplement: Supplementary file 6 [file DataSheet_6.zip › fig 5 raw/fig 5-G raw/inflammation.Gsea.1649955060129/enplot_PID_RHOA_REG_PATHWAY_250.png]

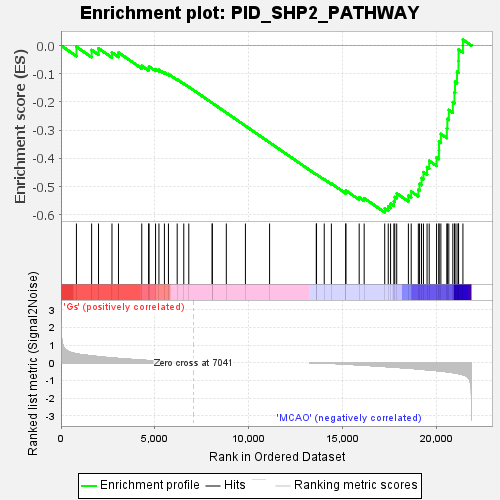

Supplement: Supplementary file 6 [file DataSheet_6.zip › fig 5 raw/fig 5-G raw/inflammation.Gsea.1649955060129/enplot_PID_SHP2_PATHWAY_247.png]

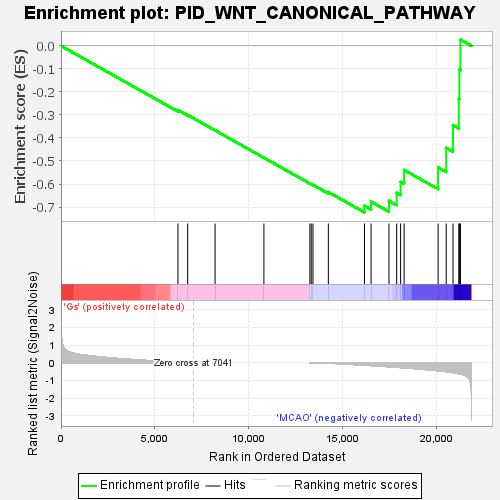

Supplement: Supplementary file 6 [file DataSheet_6.zip › fig 5 raw/fig 5-G raw/inflammation.Gsea.1649955060129/enplot_PID_WNT_CANONICAL_PATHWAY_235.png]

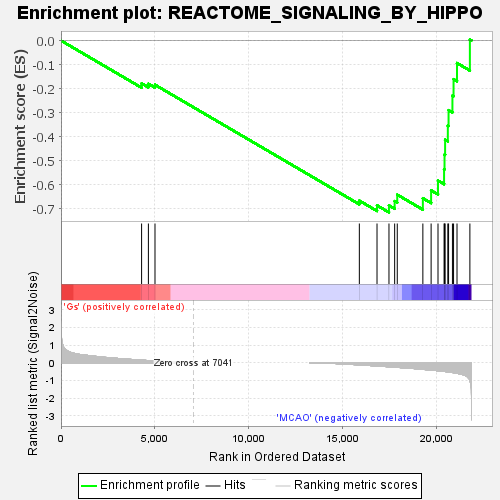

Supplement: Supplementary file 6 [file DataSheet_6.zip › fig 5 raw/fig 5-G raw/inflammation.Gsea.1649955060129/enplot_REACTOME_SIGNALING_BY_HIPPO_244.png]

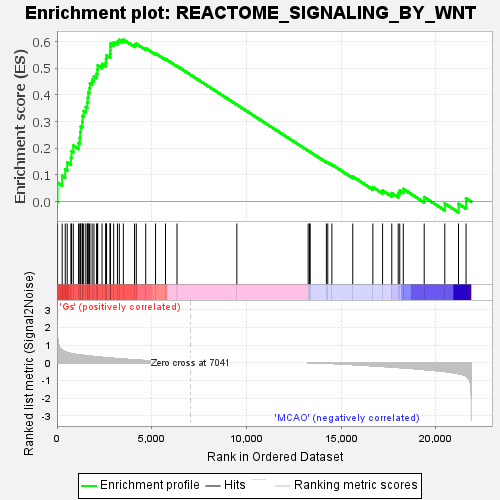

Supplement: Supplementary file 6 [file DataSheet_6.zip › fig 5 raw/fig 5-G raw/inflammation.Gsea.1649955060129/enplot_REACTOME_SIGNALING_BY_WNT_172.png]

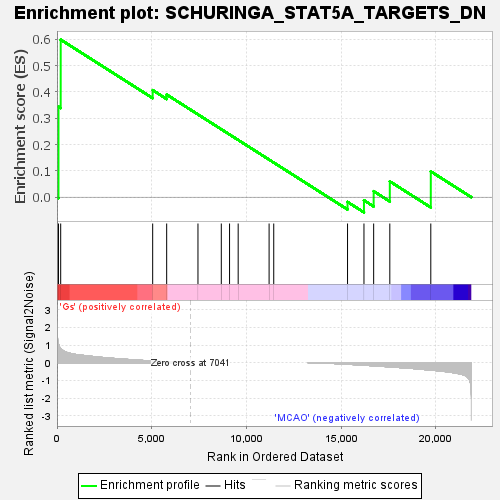

Supplement: Supplementary file 6 [file DataSheet_6.zip › fig 5 raw/fig 5-G raw/inflammation.Gsea.1649955060129/enplot_SCHURINGA_STAT5A_TARGETS_DN_187.png]

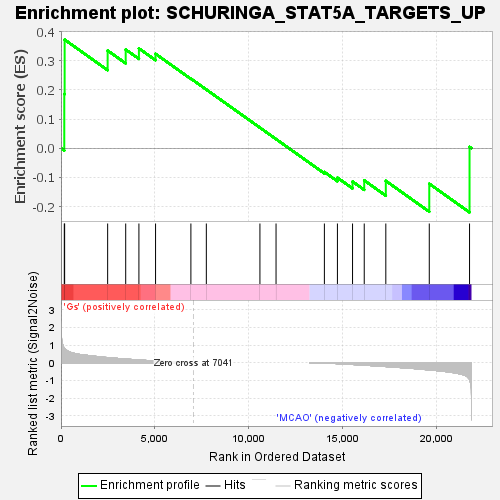

Supplement: Supplementary file 6 [file DataSheet_6.zip › fig 5 raw/fig 5-G raw/inflammation.Gsea.1649955060129/enplot_SCHURINGA_STAT5A_TARGETS_UP_211.png]

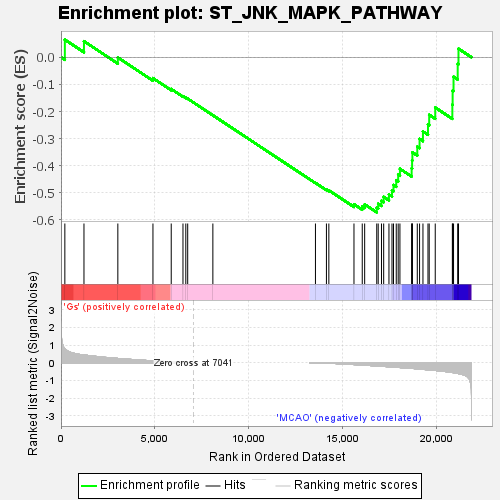

Supplement: Supplementary file 6 [file DataSheet_6.zip › fig 5 raw/fig 5-G raw/inflammation.Gsea.1649955060129/enplot_ST_JNK_MAPK_PATHWAY_286.png]

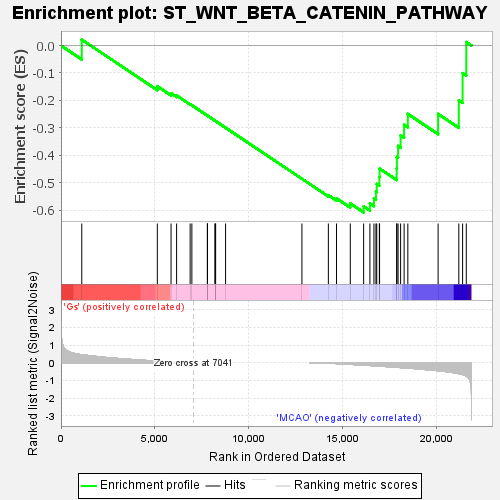

Supplement: Supplementary file 6 [file DataSheet_6.zip › fig 5 raw/fig 5-G raw/inflammation.Gsea.1649955060129/enplot_ST_WNT_BETA_CATENIN_PATHWAY_265.png]

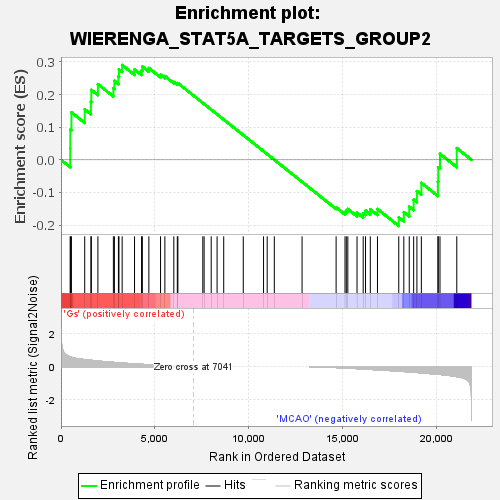

Supplement: Supplementary file 6 [file DataSheet_6.zip › fig 5 raw/fig 5-G raw/inflammation.Gsea.1649955060129/enplot_WIERENGA_STAT5A_TARGETS_GROUP2_214.png]

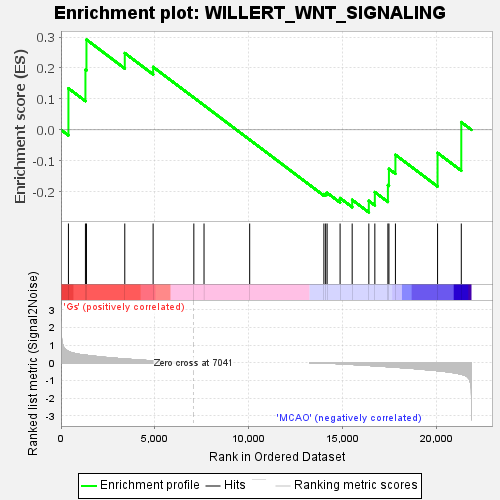

Supplement: Supplementary file 6 [file DataSheet_6.zip › fig 5 raw/fig 5-G raw/inflammation.Gsea.1649955060129/enplot_WILLERT_WNT_SIGNALING_226.png]

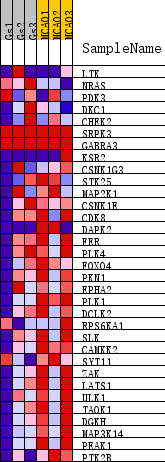

Supplement: Supplementary file 6 [file DataSheet_6.zip › fig 5 raw/fig 5-G raw/inflammation.Gsea.1649955060129/FIRESTEIN_CTNNB1_PATHWAY_254.png]

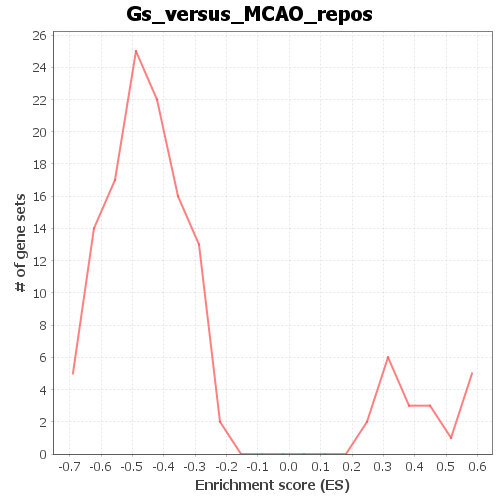

Supplement: Supplementary file 6 [file DataSheet_6.zip › fig 5 raw/fig 5-G raw/inflammation.Gsea.1649955060129/global_es_histogram.png]

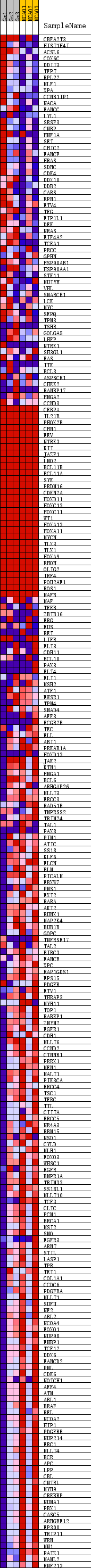

Supplement: Supplementary file 6 [file DataSheet_6.zip › fig 5 raw/fig 5-G raw/inflammation.Gsea.1649955060129/GRESHOCK_CANCER_COPY_NUMBER_UP_242.png]

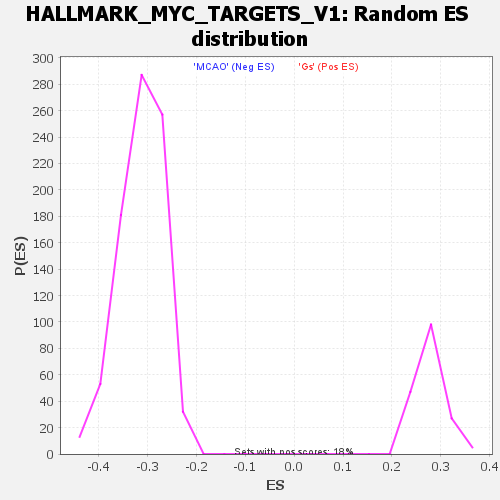

Supplement: Supplementary file 6 [file DataSheet_6.zip › fig 5 raw/fig 5-G raw/inflammation.Gsea.1649955060129/gset_rnd_es_dist_171.png]

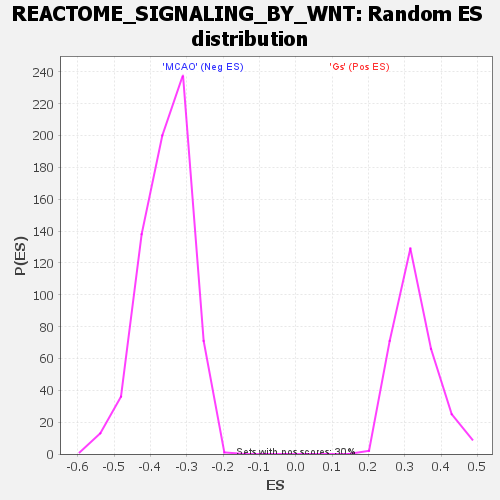

Supplement: Supplementary file 6 [file DataSheet_6.zip › fig 5 raw/fig 5-G raw/inflammation.Gsea.1649955060129/gset_rnd_es_dist_174.png]

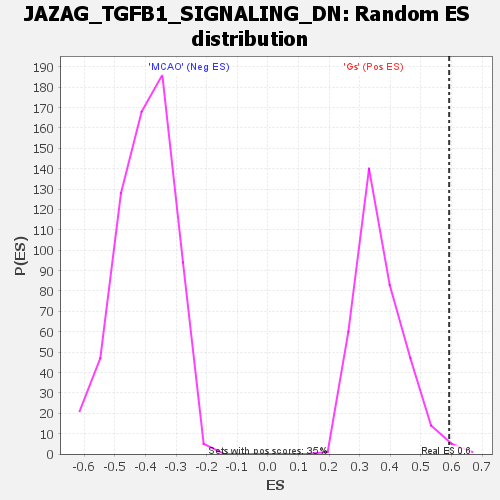

Supplement: Supplementary file 6 [file DataSheet_6.zip › fig 5 raw/fig 5-G raw/inflammation.Gsea.1649955060129/gset_rnd_es_dist_177.png]

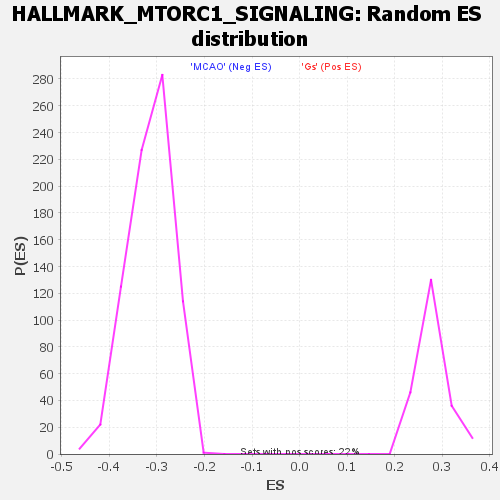

Supplement: Supplementary file 6 [file DataSheet_6.zip › fig 5 raw/fig 5-G raw/inflammation.Gsea.1649955060129/gset_rnd_es_dist_180.png]

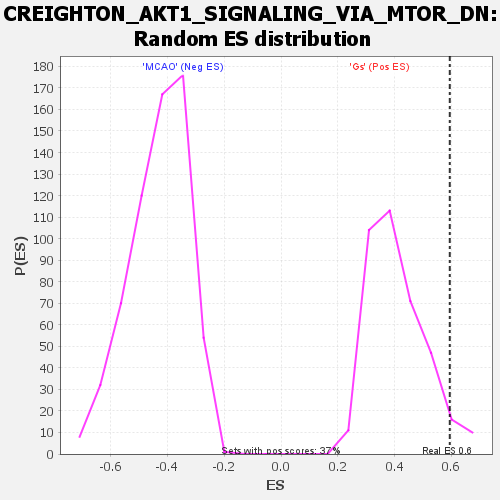

Supplement: Supplementary file 6 [file DataSheet_6.zip › fig 5 raw/fig 5-G raw/inflammation.Gsea.1649955060129/gset_rnd_es_dist_183.png]

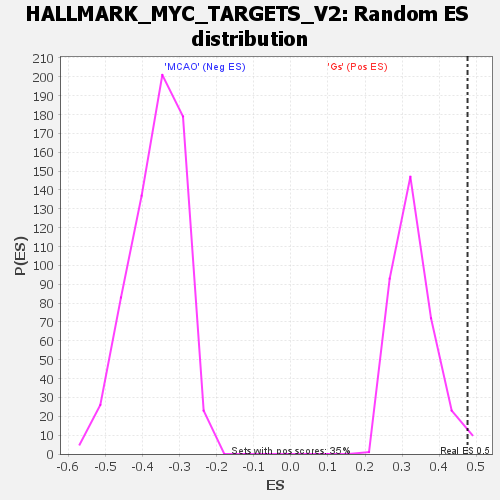

Supplement: Supplementary file 6 [file DataSheet_6.zip › fig 5 raw/fig 5-G raw/inflammation.Gsea.1649955060129/gset_rnd_es_dist_186.png]

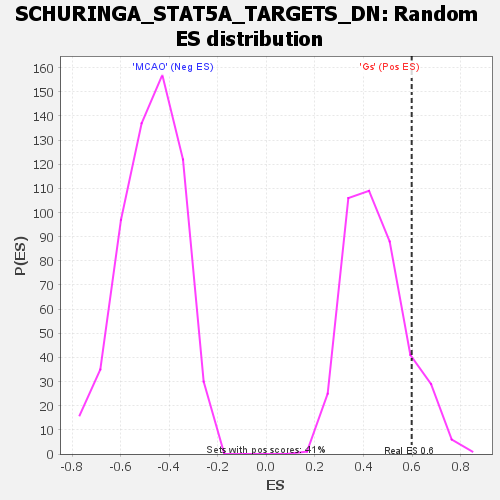

Supplement: Supplementary file 6 [file DataSheet_6.zip › fig 5 raw/fig 5-G raw/inflammation.Gsea.1649955060129/gset_rnd_es_dist_189.png]

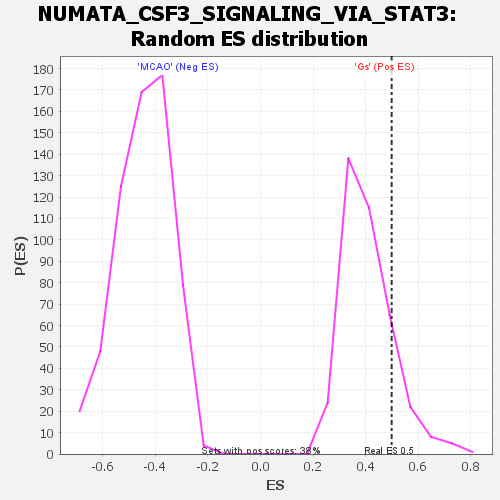

Supplement: Supplementary file 6 [file DataSheet_6.zip › fig 5 raw/fig 5-G raw/inflammation.Gsea.1649955060129/gset_rnd_es_dist_192.png]

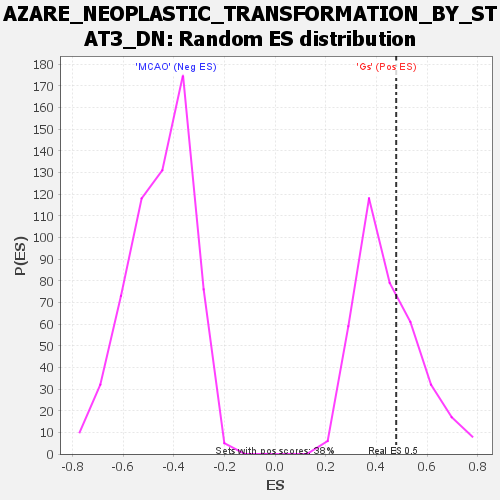

Supplement: Supplementary file 6 [file DataSheet_6.zip › fig 5 raw/fig 5-G raw/inflammation.Gsea.1649955060129/gset_rnd_es_dist_195.png]

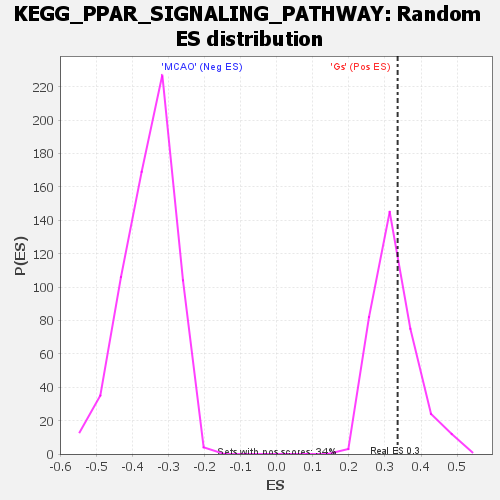

Supplement: Supplementary file 6 [file DataSheet_6.zip › fig 5 raw/fig 5-G raw/inflammation.Gsea.1649955060129/gset_rnd_es_dist_198.png]

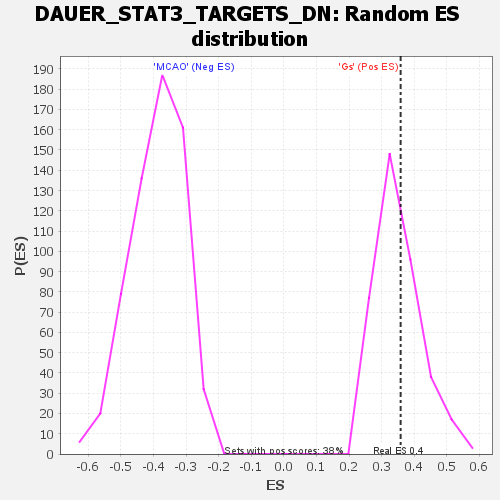

Supplement: Supplementary file 6 [file DataSheet_6.zip › fig 5 raw/fig 5-G raw/inflammation.Gsea.1649955060129/gset_rnd_es_dist_201.png]

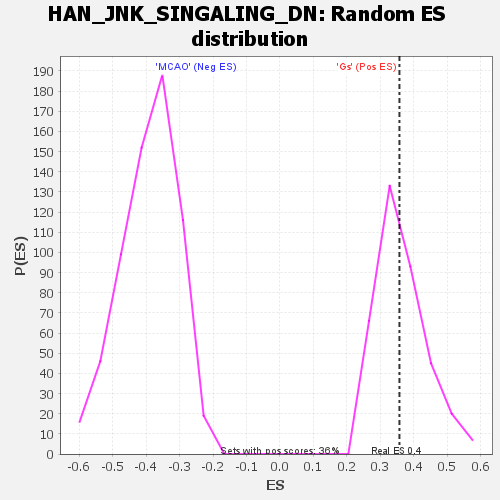

Supplement: Supplementary file 6 [file DataSheet_6.zip › fig 5 raw/fig 5-G raw/inflammation.Gsea.1649955060129/gset_rnd_es_dist_204.png]

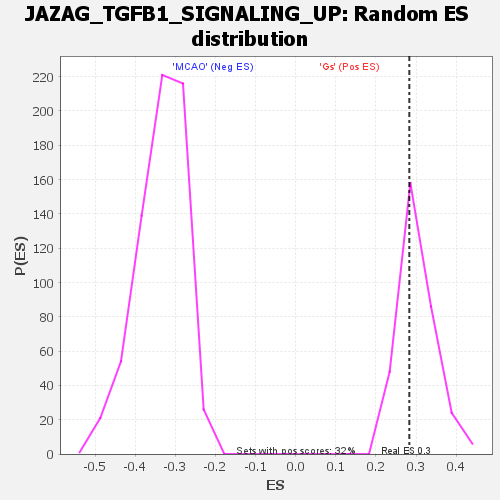

Supplement: Supplementary file 6 [file DataSheet_6.zip › fig 5 raw/fig 5-G raw/inflammation.Gsea.1649955060129/gset_rnd_es_dist_207.png]

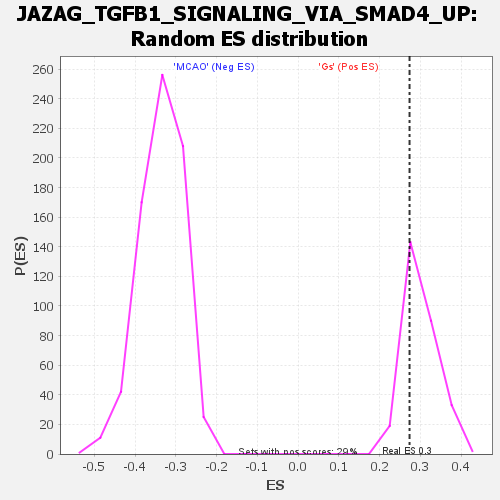

Supplement: Supplementary file 6 [file DataSheet_6.zip › fig 5 raw/fig 5-G raw/inflammation.Gsea.1649955060129/gset_rnd_es_dist_210.png]

JAK2

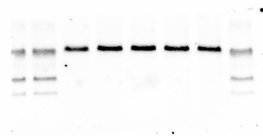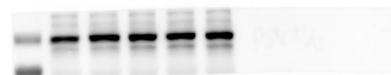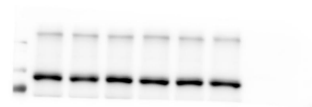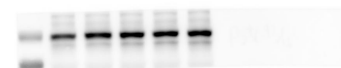

JAK3

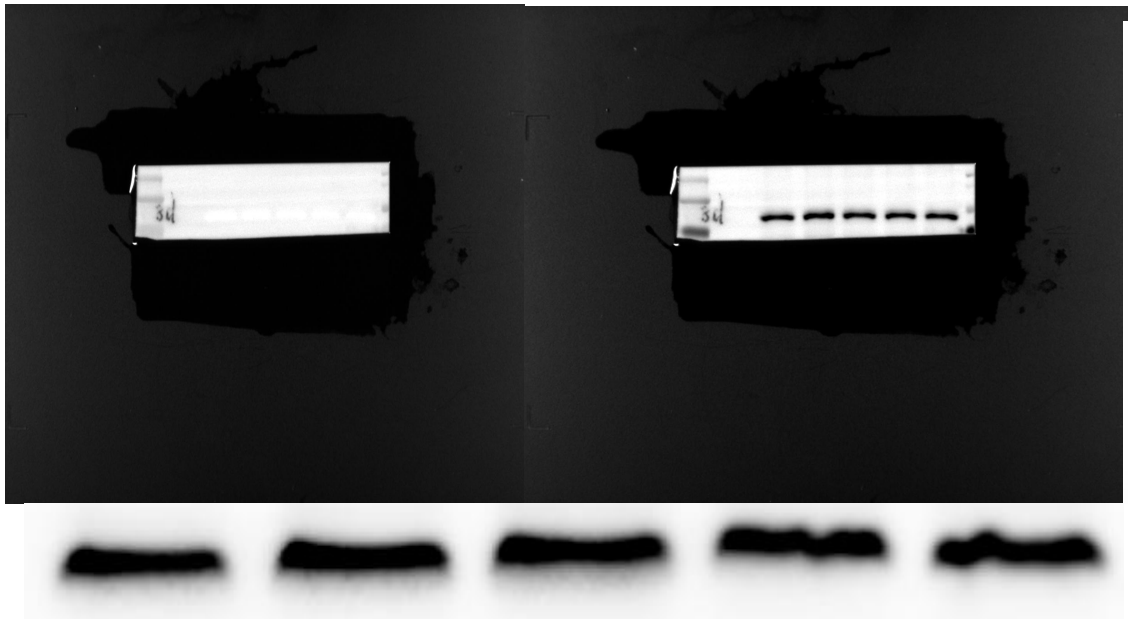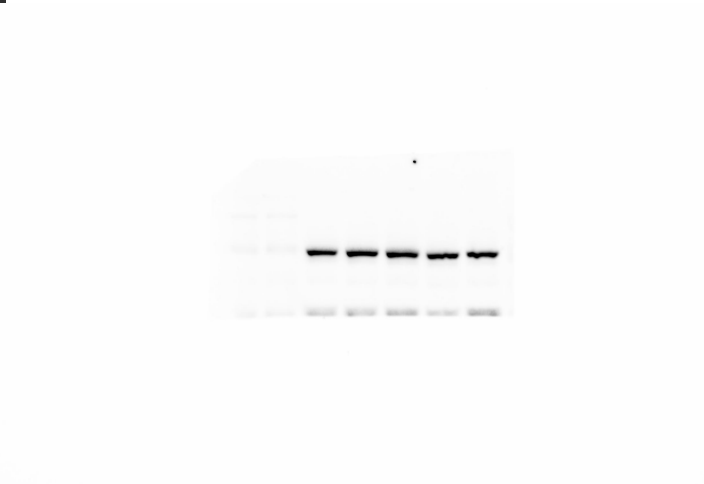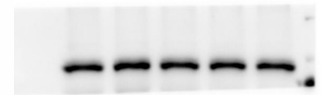

P-JAK2

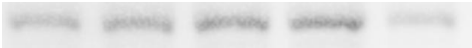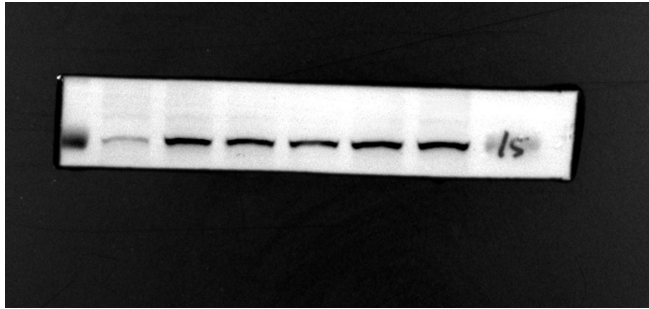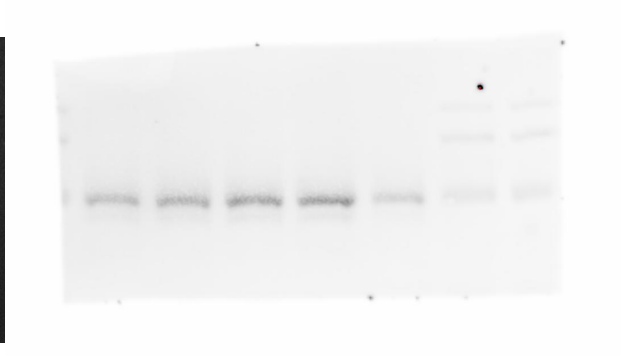

P-JAK3

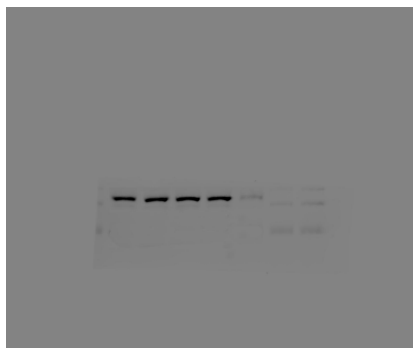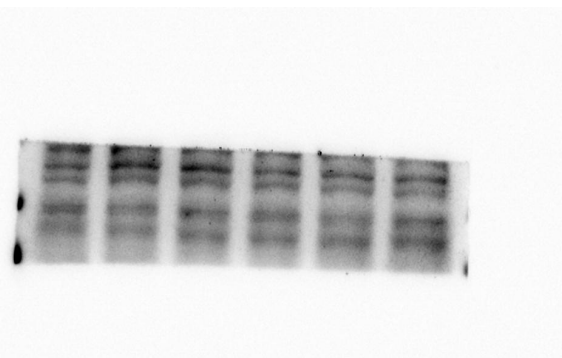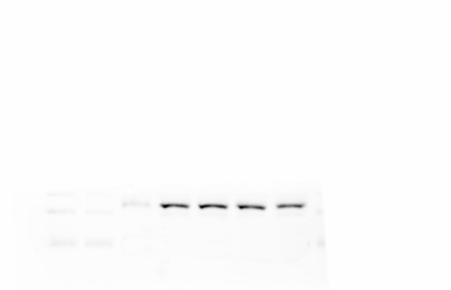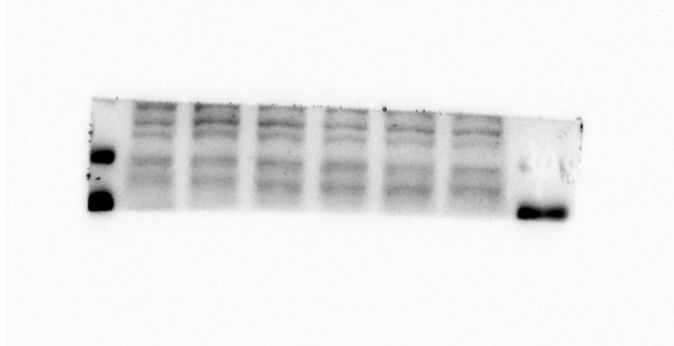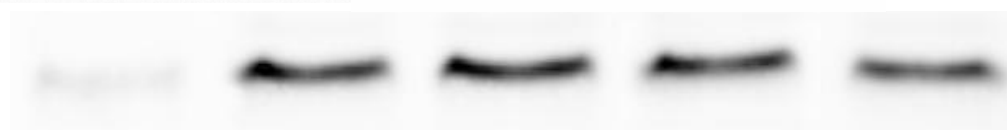

P-STAT3

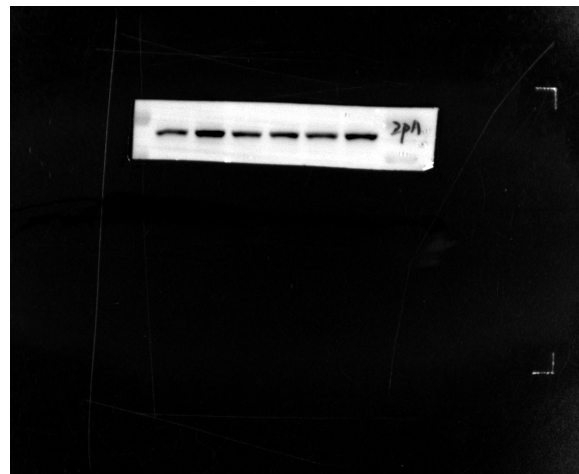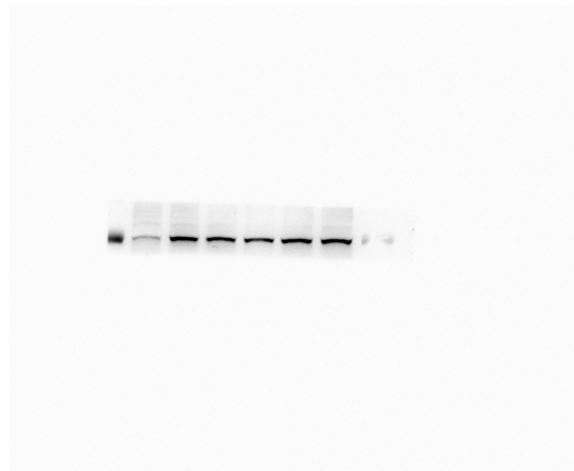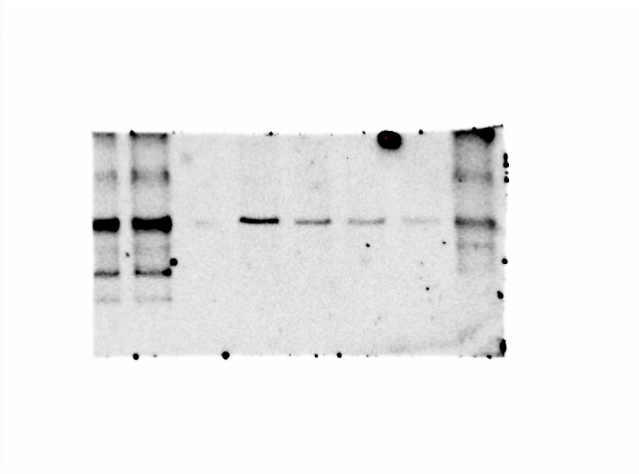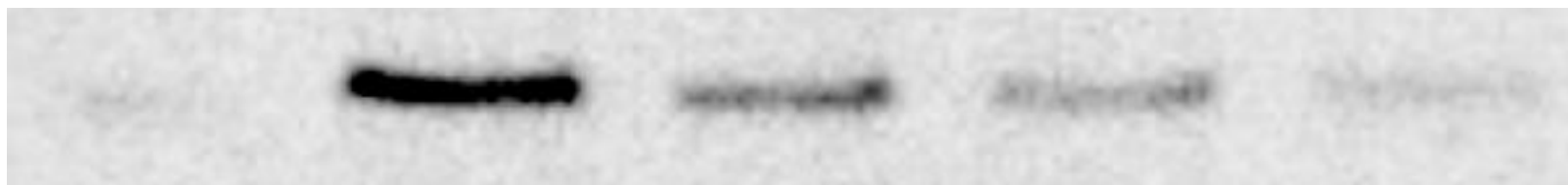

STAT3

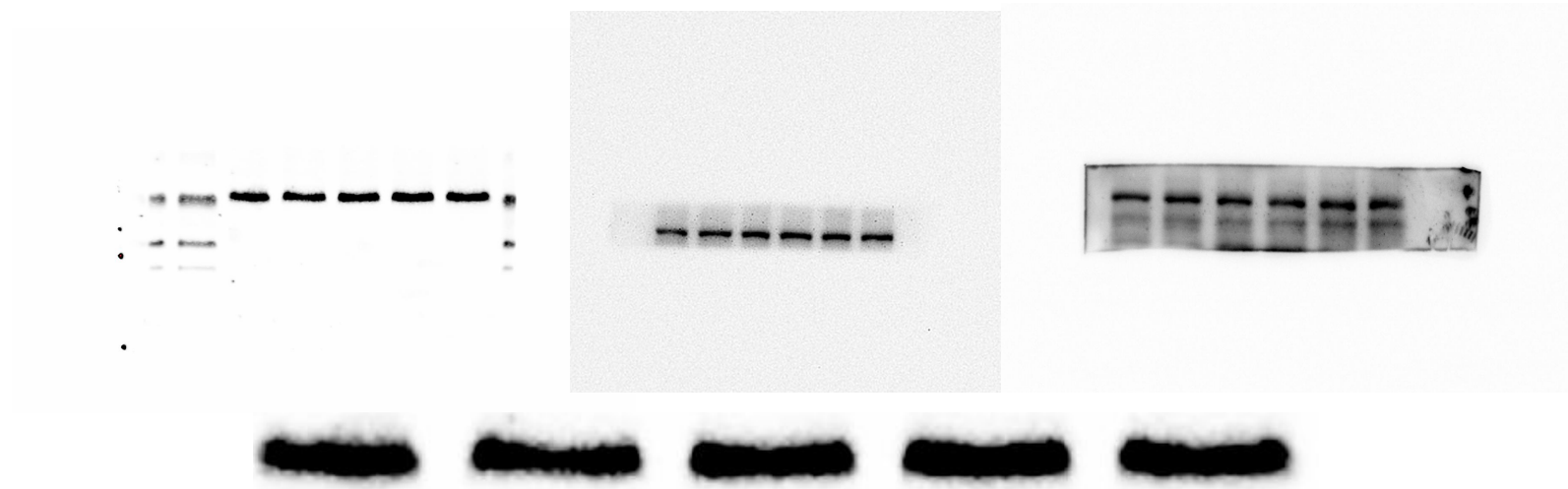

Supplement: Supplementary file 7 [file DataSheet_7.zip › fig 6-A raw/fig 6-A.pdf]

## Slide 1
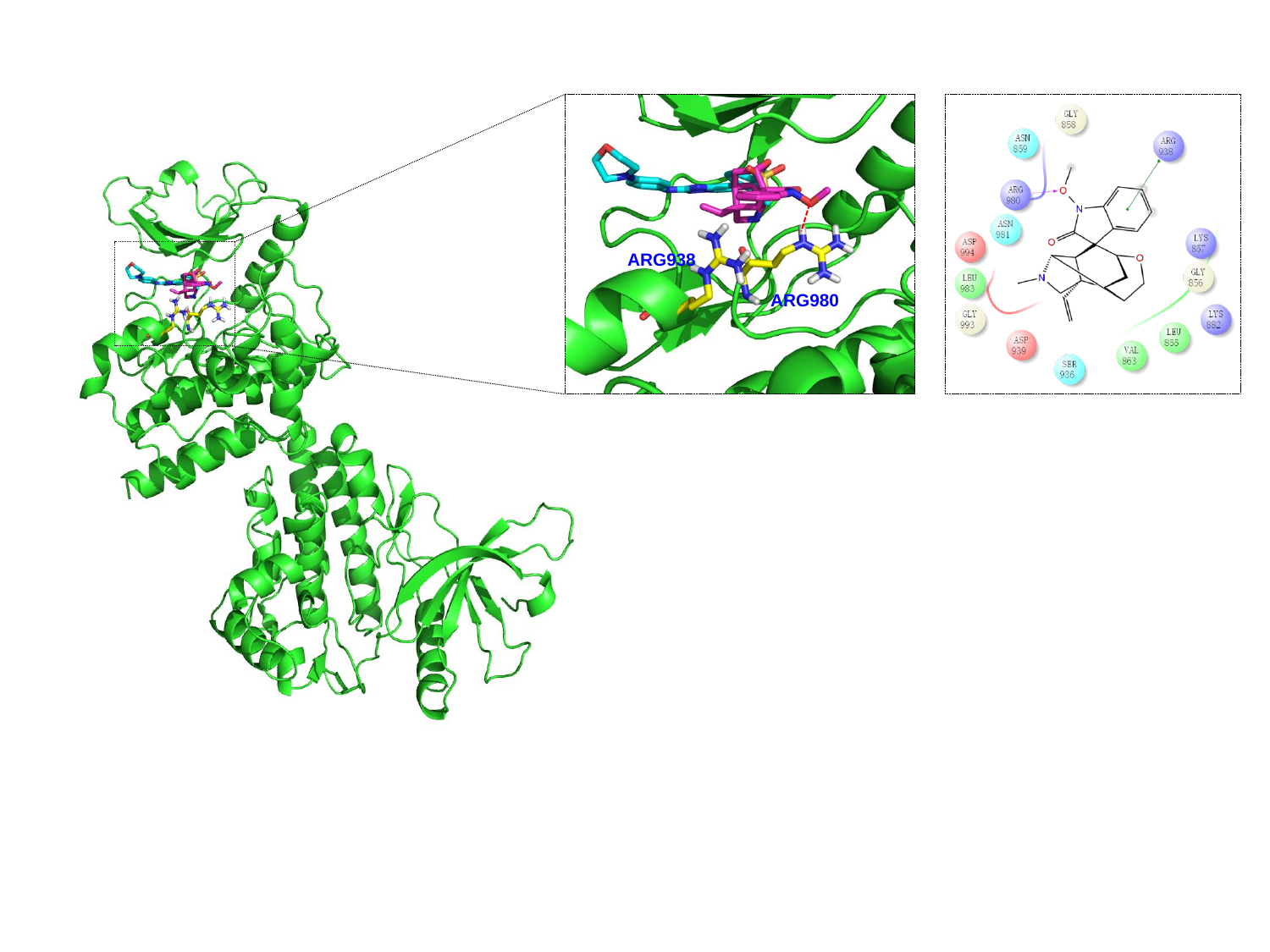

ARG938
ARG980

## Slide 2
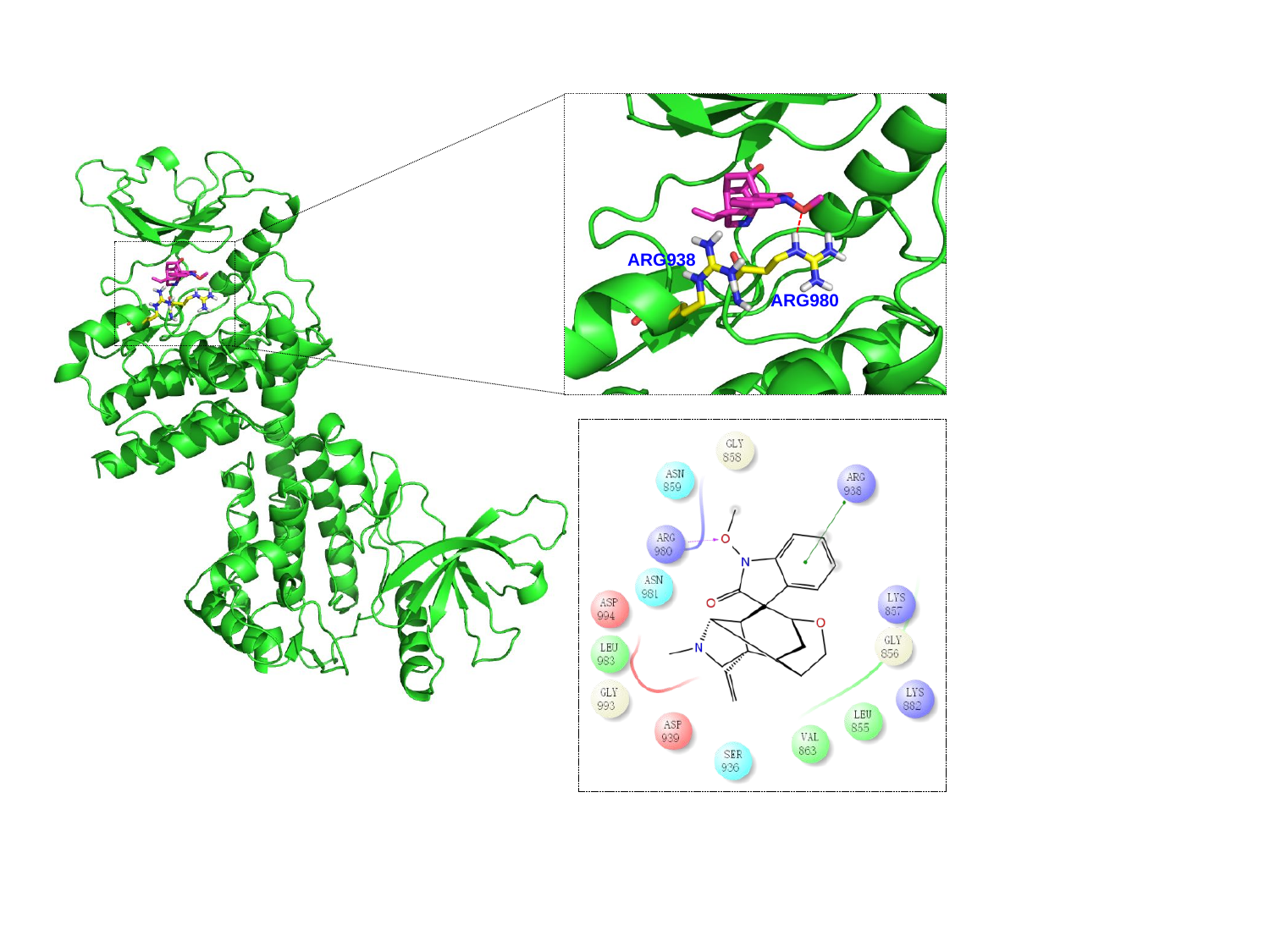

ARG938
ARG980

Supplement: Supplementary file 7 [file DataSheet_7.zip › fig 6-B raw/作图 - 钩吻-JAK2-20220123.pptx]

control

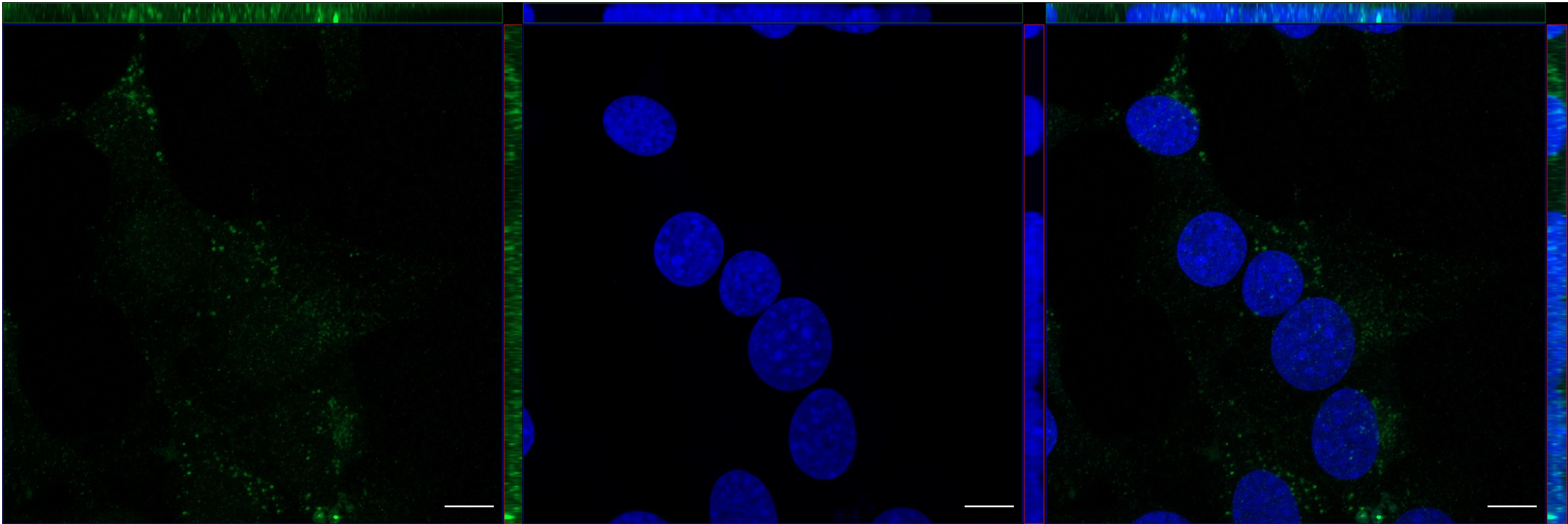

CM

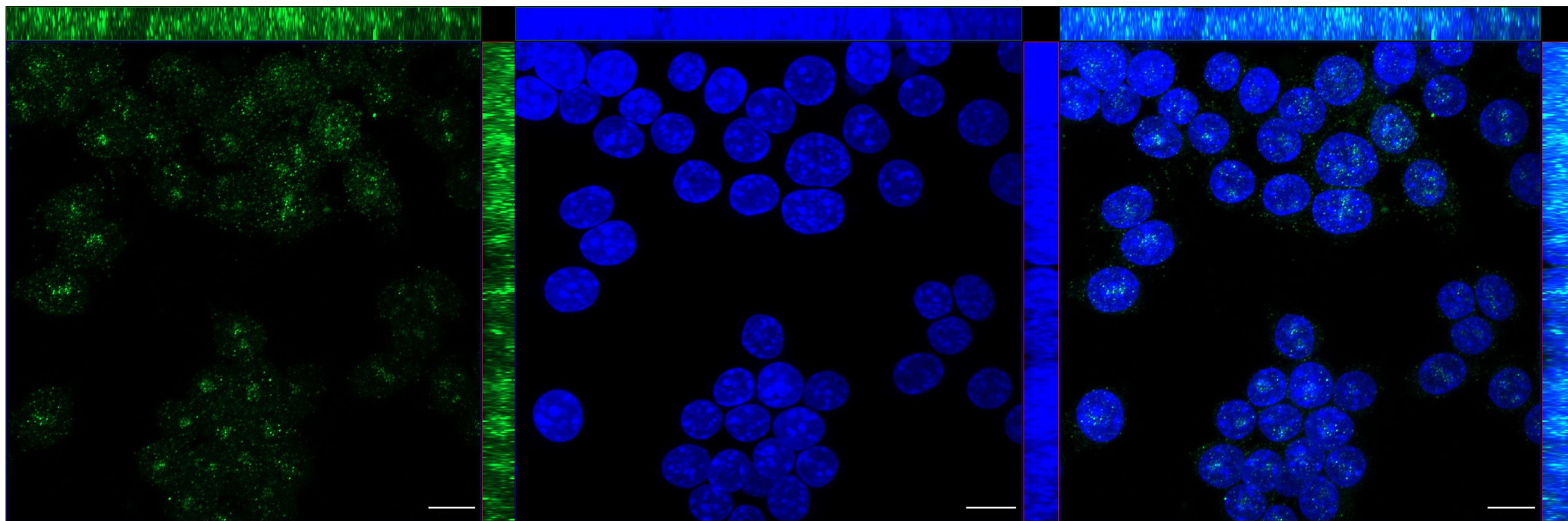

CM+Gs (5  $\mu$ M)

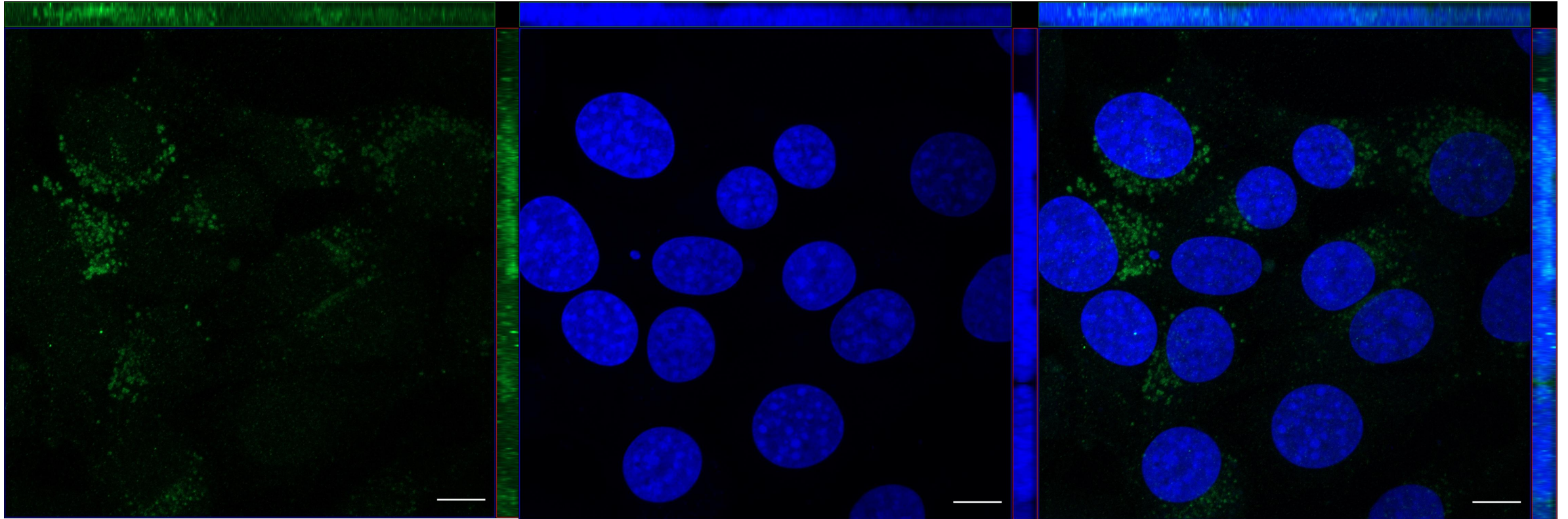

CM+Gs (20  $\mu$ M)

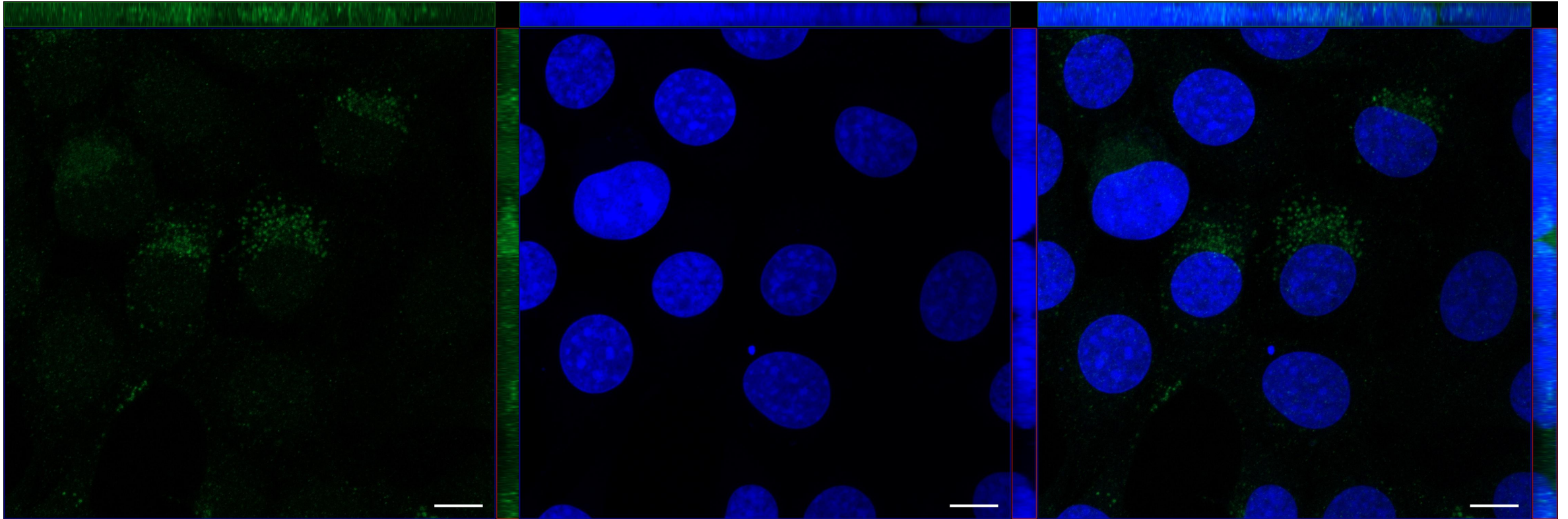

Supplement: Supplementary file 7 [file DataSheet_7.zip › fig 6-E raw/fig 6-E raw.pdf]

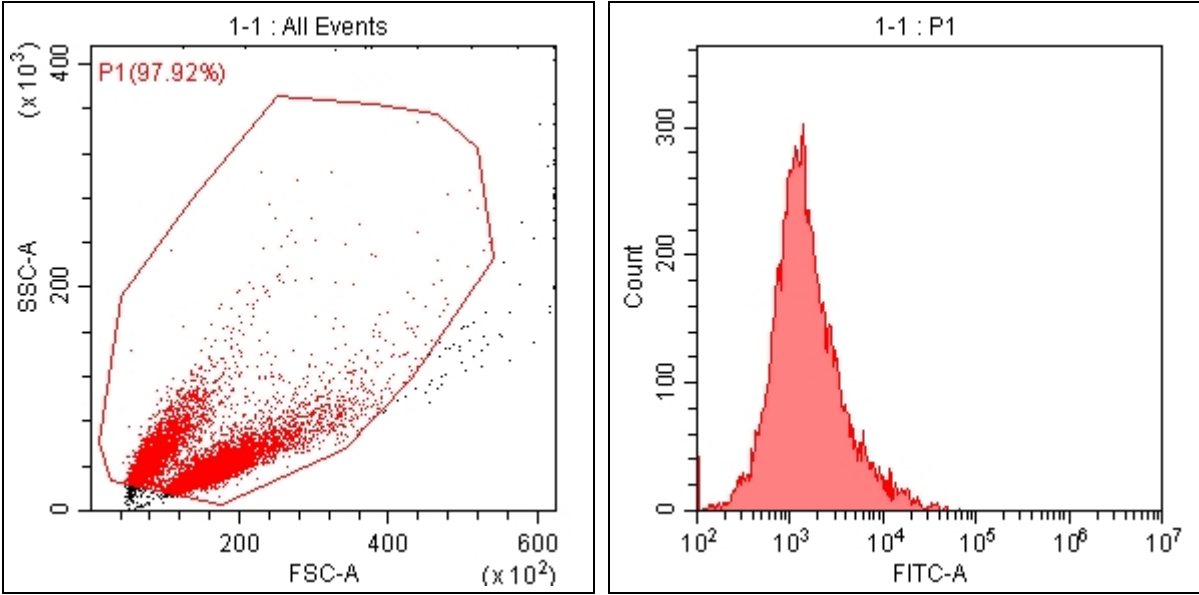

试管名称: 1-1  
样本ID:

| 群体           | %父群     | Mean FITC-A |
|--------------|---------|-------------|
| ● All Events | 100.00% | 2722.4      |
| ● P1         | 97.92%  | 2646.0      |

Supplement: Supplementary file 8 [file DataSheet_8.zip › fig 7-D raw/1-1.pdf]

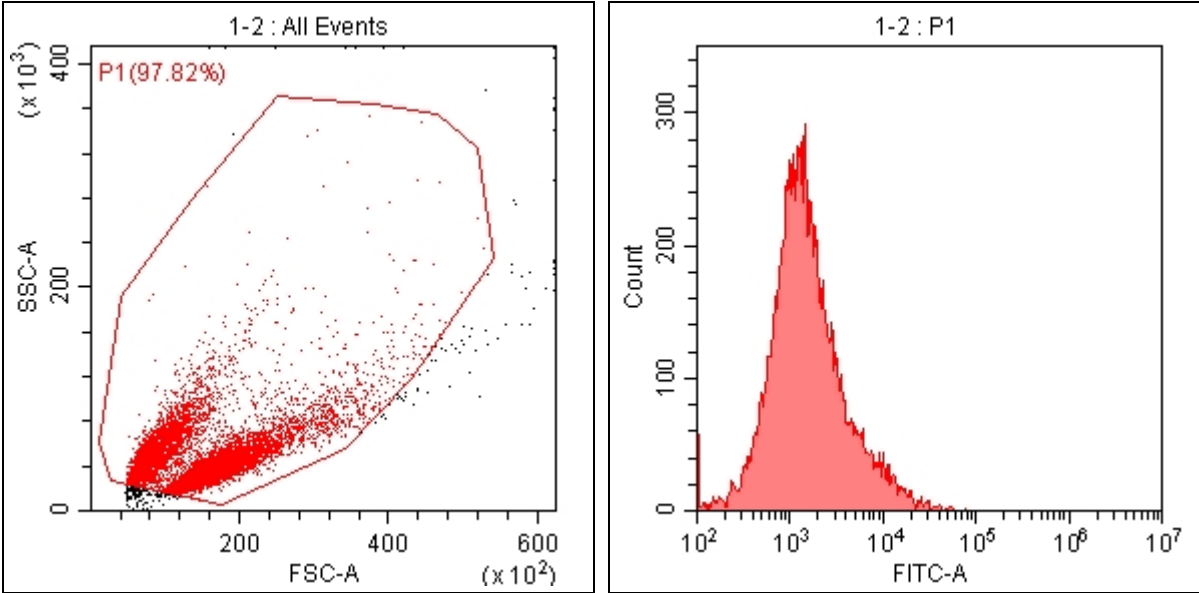

试管名称： 1-2  
样本ID：

| 群体           | %父群     | Mean FITC-A |
|--------------|---------|-------------|
| ● All Events | 100.00% | 2815.3      |
| ● P1         | 97.82%  | 2779.3      |

Supplement: Supplementary file 8 [file DataSheet_8.zip › fig 7-D raw/1-2.pdf]

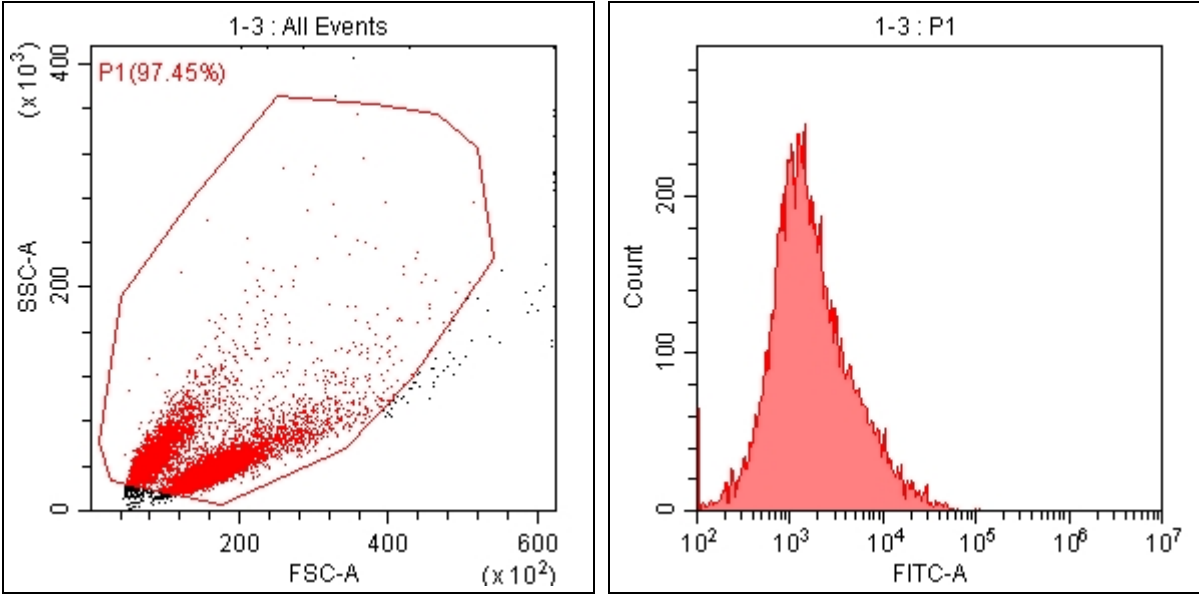

试管名称: 1-3  
样本ID:

| 群体           | %父群     | Mean FITC-A |
|--------------|---------|-------------|
| ● All Events | 100.00% | 3268.1      |
| ● P1         | 97.45%  | 3252.3      |

Supplement: Supplementary file 8 [file DataSheet_8.zip › fig 7-D raw/1-3.pdf]

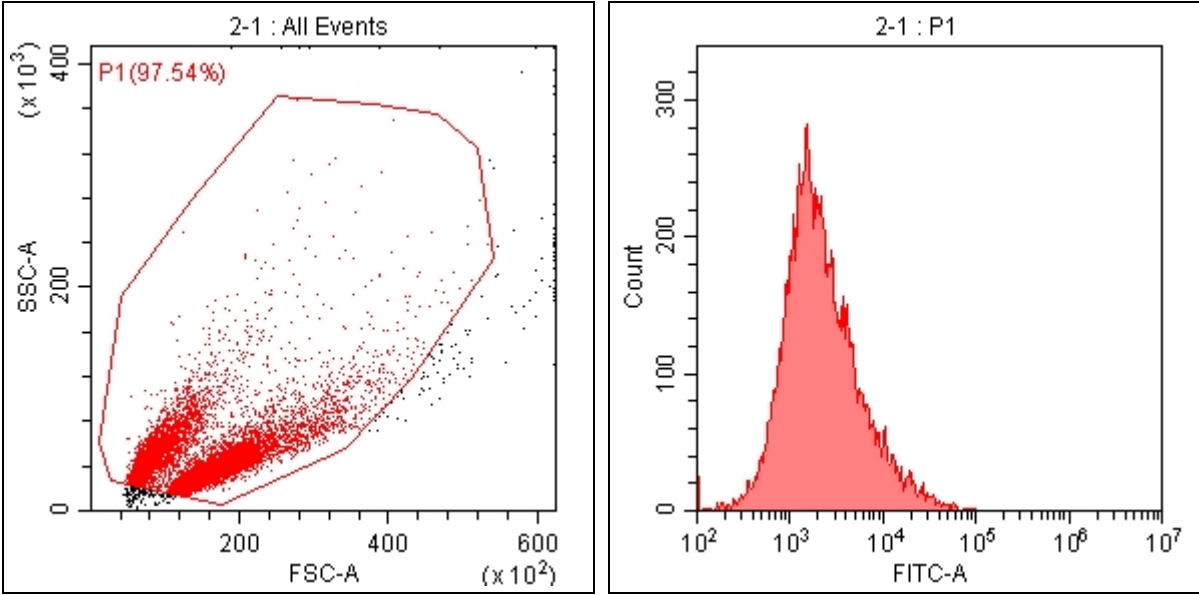

试管名称： 2-1  
样本ID：

| 群体           | %父群     | Mean FITC-A |
|--------------|---------|-------------|
| ● All Events | 100.00% | 4225.4      |
| ● P1         | 97.54%  | 4121.6      |

Supplement: Supplementary file 8 [file DataSheet_8.zip › fig 7-D raw/2-1.pdf]

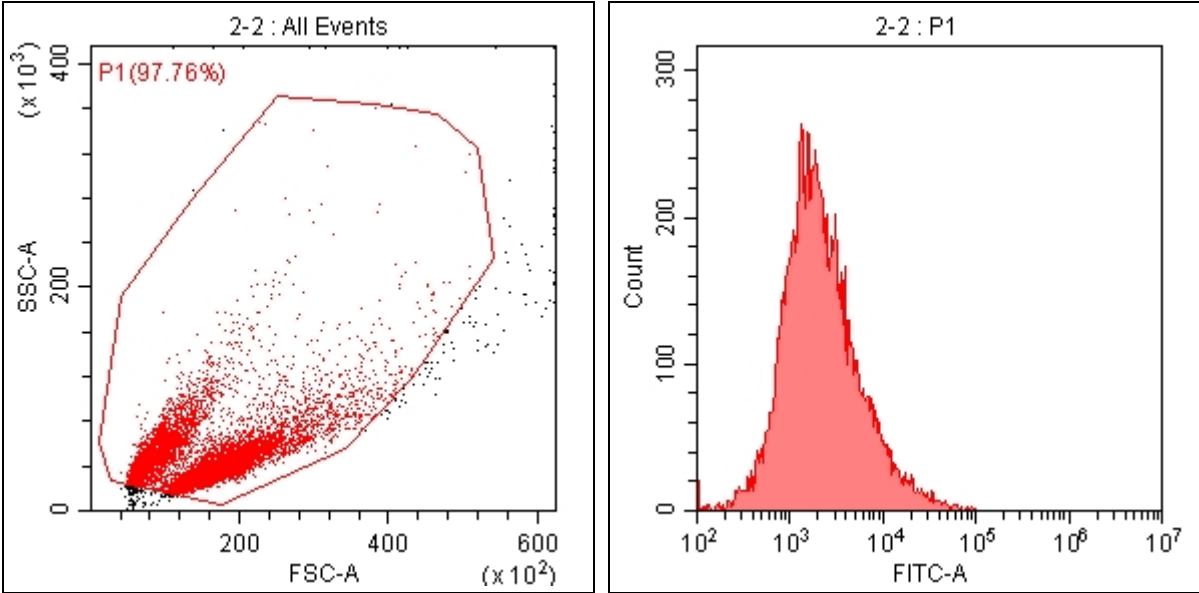

试管名称： 2-2  
样本ID：

| 群体           | %父群     | Mean FITC-A |
|--------------|---------|-------------|
| ● All Events | 100.00% | 4086.6      |
| ● P1         | 97.76%  | 3988.7      |

Supplement: Supplementary file 8 [file DataSheet_8.zip › fig 7-D raw/2-2.pdf]

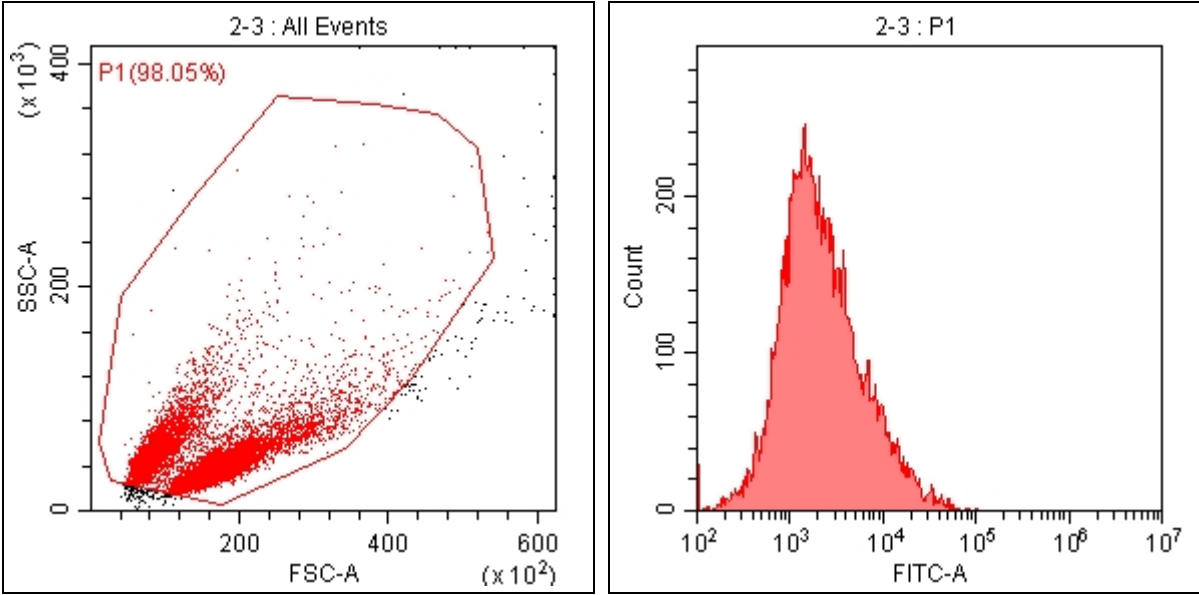

试管名称: 2-3  
样本ID:

| 群体           | %父群     | Mean FITC-A |
|--------------|---------|-------------|
| ● All Events | 100.00% | 4187.0      |
| ● P1         | 98.05%  | 4087.3      |

Supplement: Supplementary file 8 [file DataSheet_8.zip › fig 7-D raw/2-3.pdf]

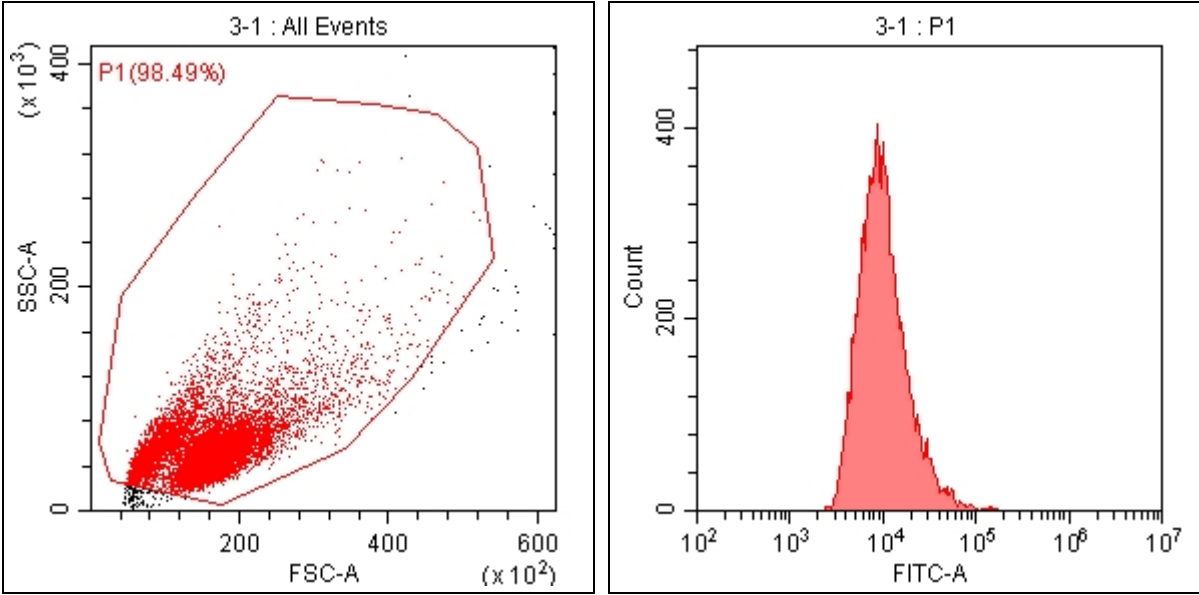

试管名称: 3-1  
样本ID:

| 群体           | %父群     | Mean FITC-A |
|--------------|---------|-------------|
| ● All Events | 100.00% | 13538.5     |
| ● P1         | 98.49%  | 13224.1     |

Supplement: Supplementary file 8 [file DataSheet_8.zip › fig 7-D raw/3-1.pdf]

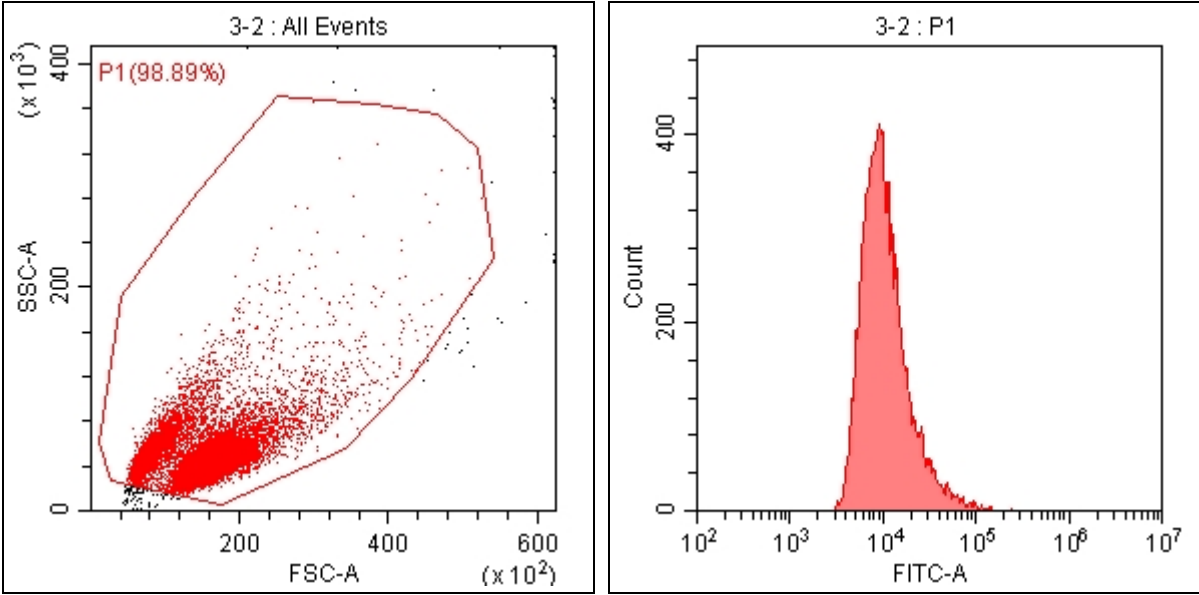

试管名称： 3-2  
样本ID：

| 群体           | %父群     | Mean FITC-A |
|--------------|---------|-------------|
| ● All Events | 100.00% | 14464.3     |
| ● P1         | 98.89%  | 14329.2     |

Supplement: Supplementary file 8 [file DataSheet_8.zip › fig 7-D raw/3-2.pdf]

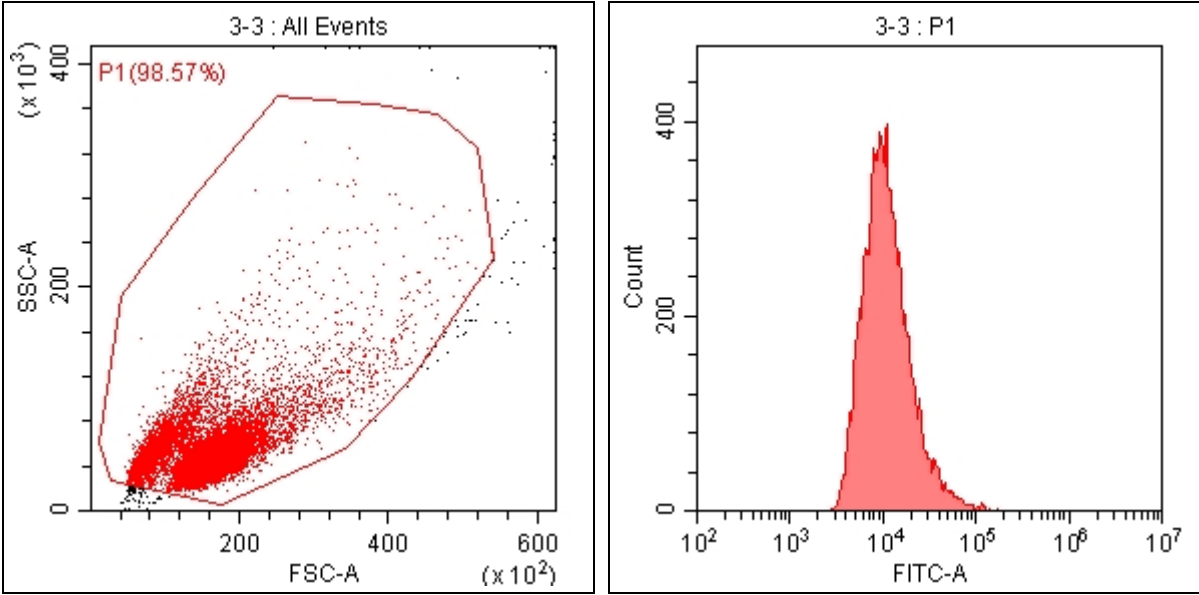

试管名称: 3-3  
样本ID:

| 群体           | %父群     | Mean FITC-A |
|--------------|---------|-------------|
| ● All Events | 100.00% | 15124.0     |
| ● P1         | 98.57%  | 14839.1     |

Supplement: Supplementary file 8 [file DataSheet_8.zip › fig 7-D raw/3-3.pdf]

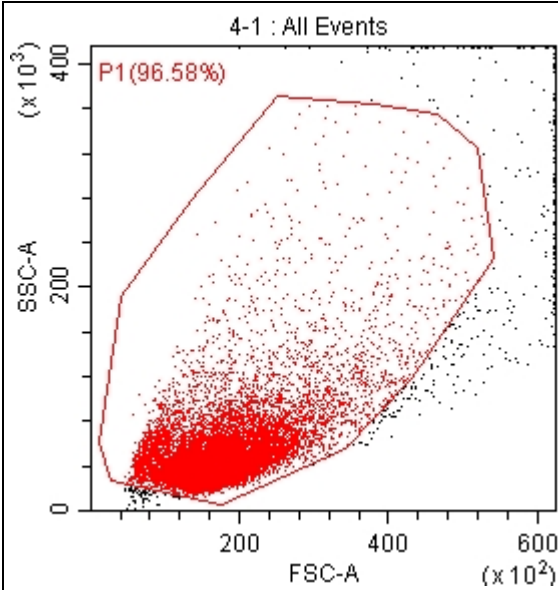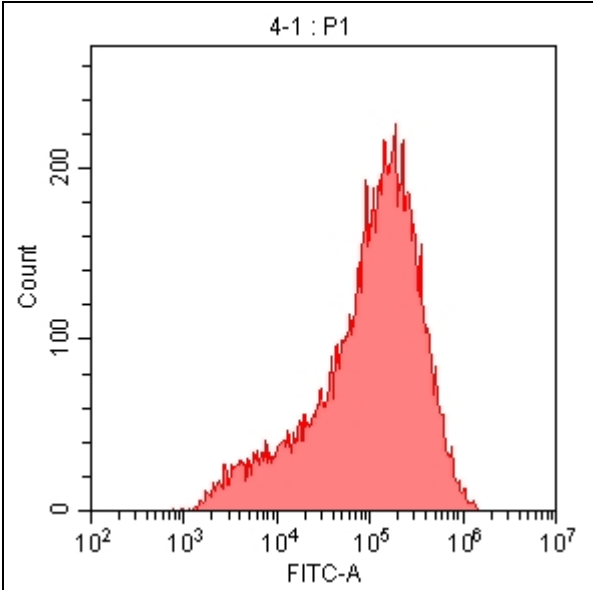

试管名称: 4-1  
样本ID:

| 群体           | %父群     | Mean FITC-A |
|--------------|---------|-------------|
| ● All Events | 100.00% | 176644.1    |
| ● P1         | 96.58%  | 166714.8    |

Supplement: Supplementary file 8 [file DataSheet_8.zip › fig 7-D raw/4-1.pdf]

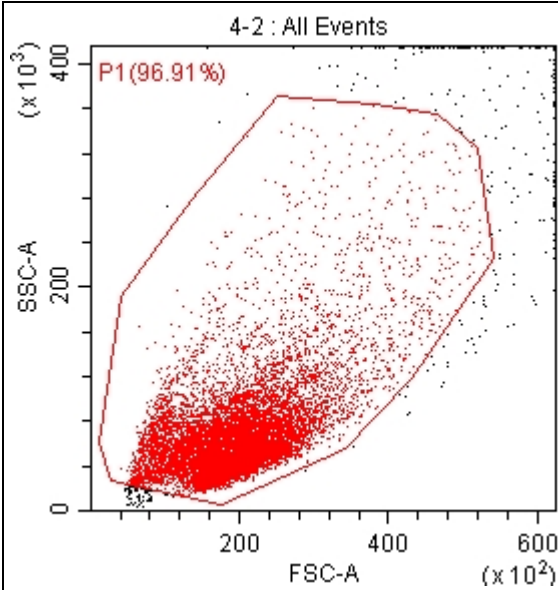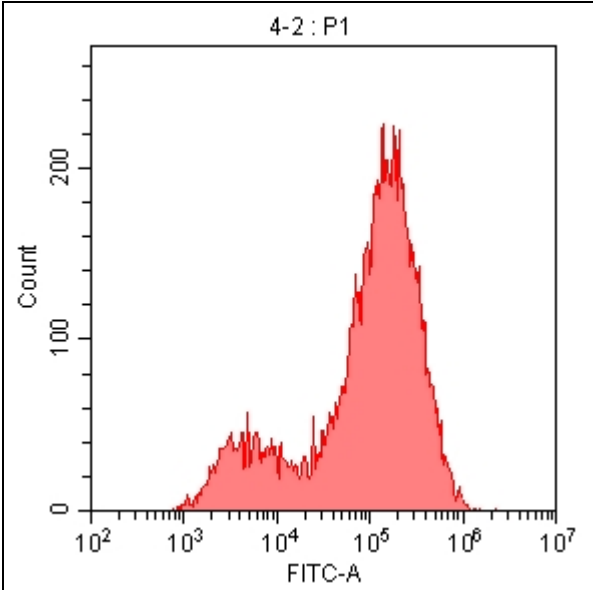

试管名称: 4-2  
样本ID:

| 群体           | %父群     | Mean FITC-A |
|--------------|---------|-------------|
| ● All Events | 100.00% | 162766.2    |
| ● P1         | 96.91%  | 157533.4    |

Supplement: Supplementary file 8 [file DataSheet_8.zip › fig 7-D raw/4-2.pdf]

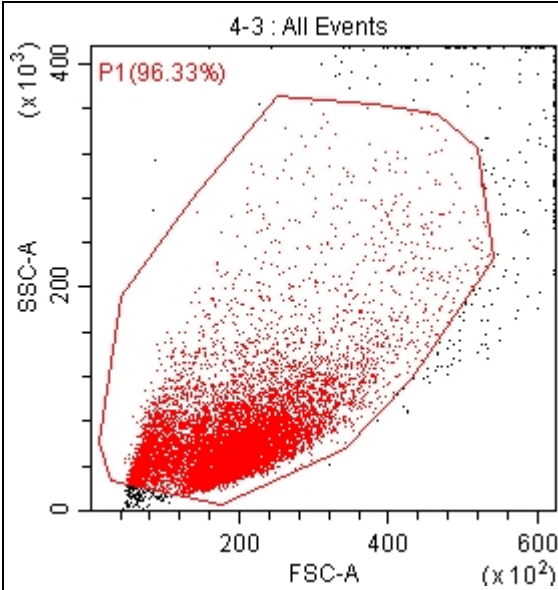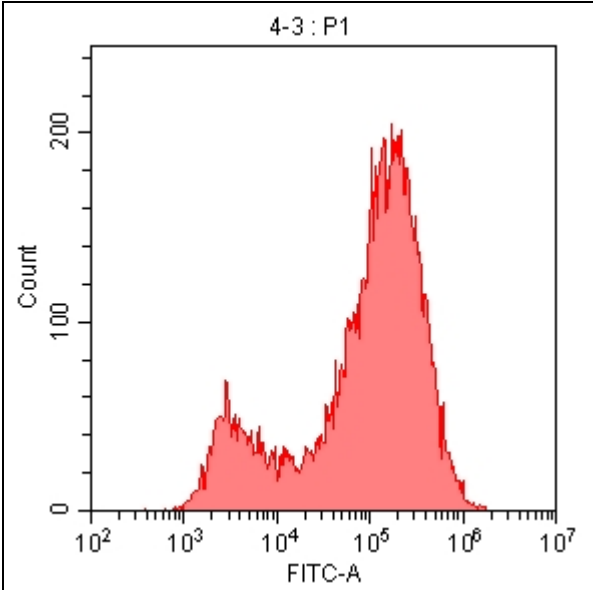

试管名称: 4-3  
样本ID:

| 群体           | %父群     | Mean FITC-A |
|--------------|---------|-------------|
| ● All Events | 100.00% | 170438.7    |
| ● P1         | 96.33%  | 165257.9    |

Supplement: Supplementary file 8 [file DataSheet_8.zip › fig 7-D raw/4-3.pdf]

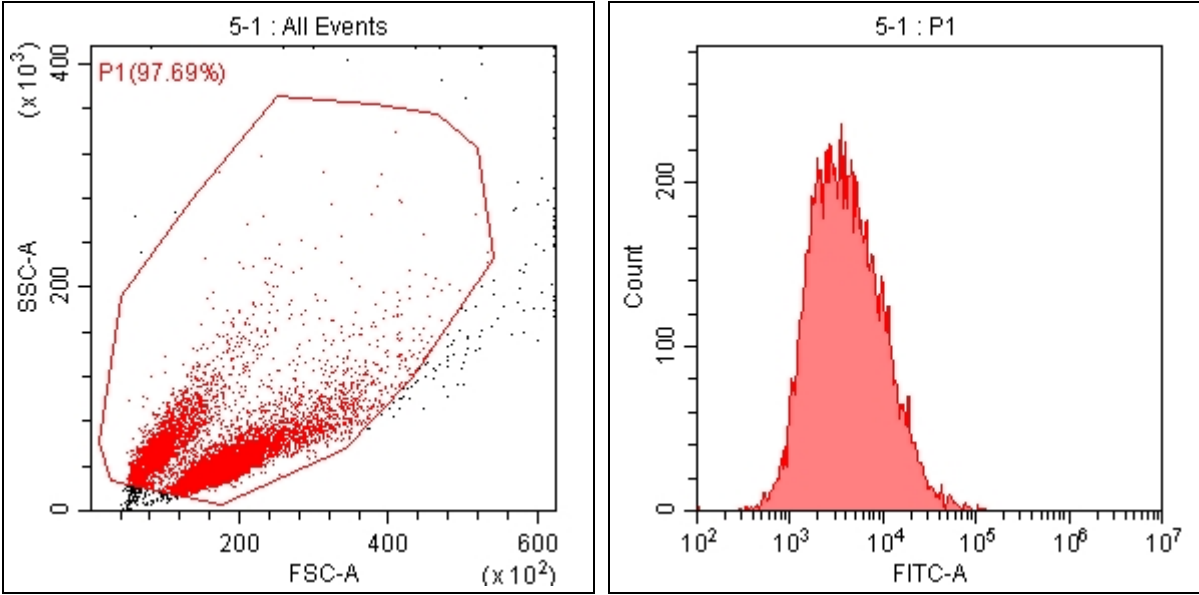

试管名称: 5-1  
样本ID:

| 群体           | %父群     | Mean FITC-A |
|--------------|---------|-------------|
| ● All Events | 100.00% | 6542.3      |
| ● P1         | 97.69%  | 6391.2      |

Supplement: Supplementary file 8 [file DataSheet_8.zip › fig 7-D raw/5-1.pdf]

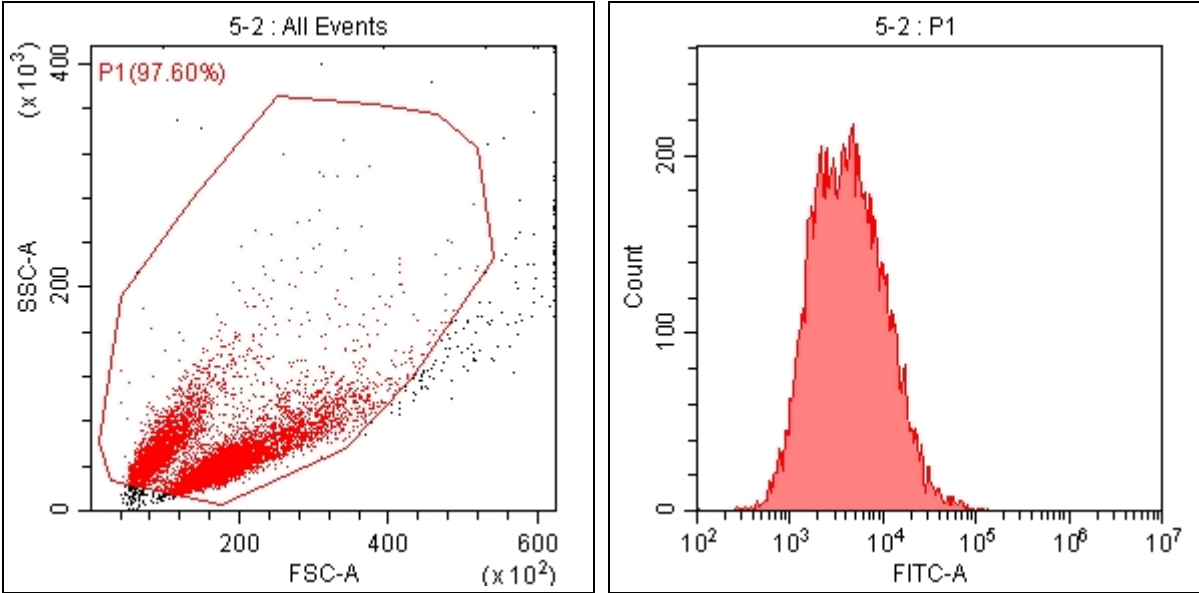

试管名称： 5-2  
样本ID：

| 群体           | %父群     | Mean FITC-A |
|--------------|---------|-------------|
| ● All Events | 100.00% | 6925.9      |
| ● P1         | 97.60%  | 6621.5      |

Supplement: Supplementary file 8 [file DataSheet_8.zip › fig 7-D raw/5-2.pdf]

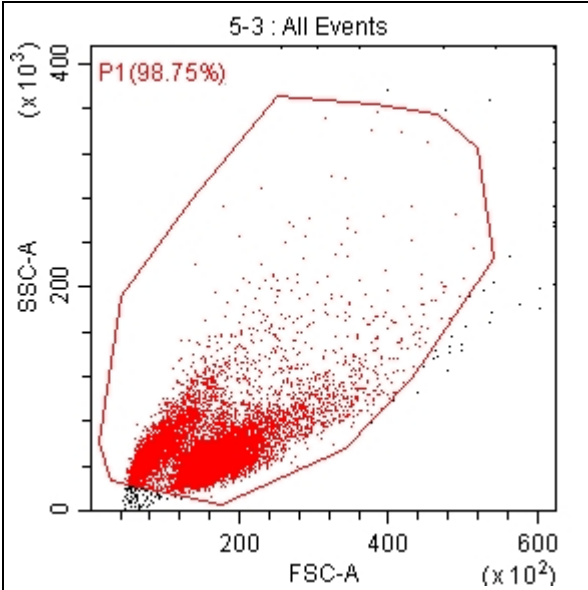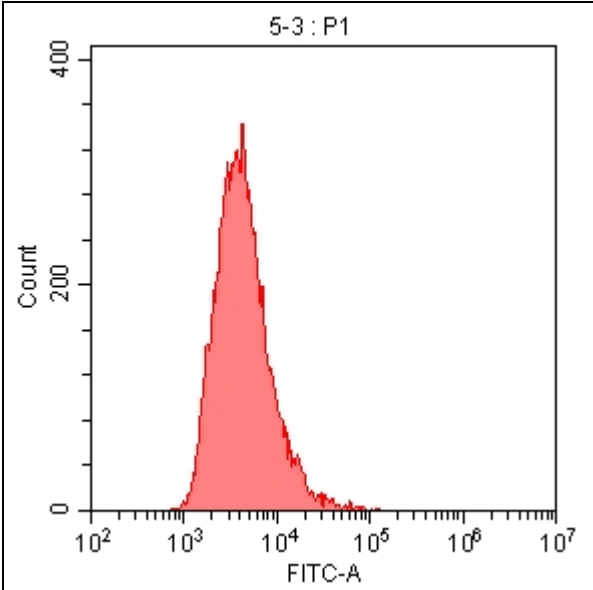

试管名称: 5-3

样本ID:

| 群体           | %父群     | Mean FITC-A |
|--------------|---------|-------------|
| ● All Events | 100.00% | 6461.7      |
| ● P1         | 98.75%  | 6334.6      |

Supplement: Supplementary file 8 [file DataSheet_8.zip › fig 7-D raw/5-3.pdf]

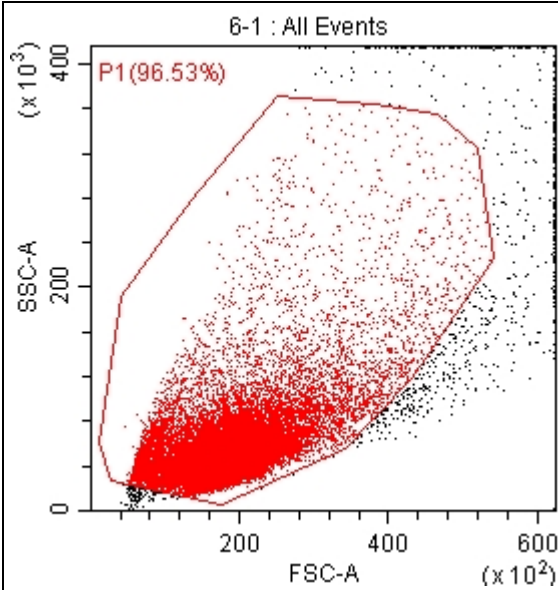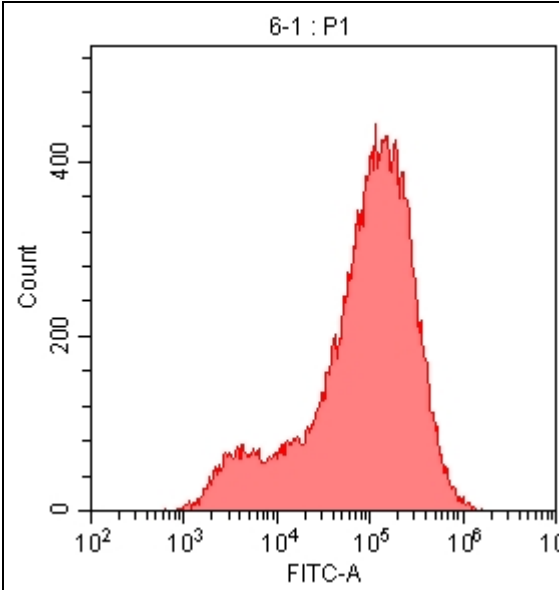

试管名称: 6-1  
样本ID:

| 群体           | %父群     | Mean FITC-A |
|--------------|---------|-------------|
| ● All Events | 100.00% | 148098.1    |
| ● P1         | 96.53%  | 141437.3    |

Supplement: Supplementary file 8 [file DataSheet_8.zip › fig 7-D raw/6-1.pdf]

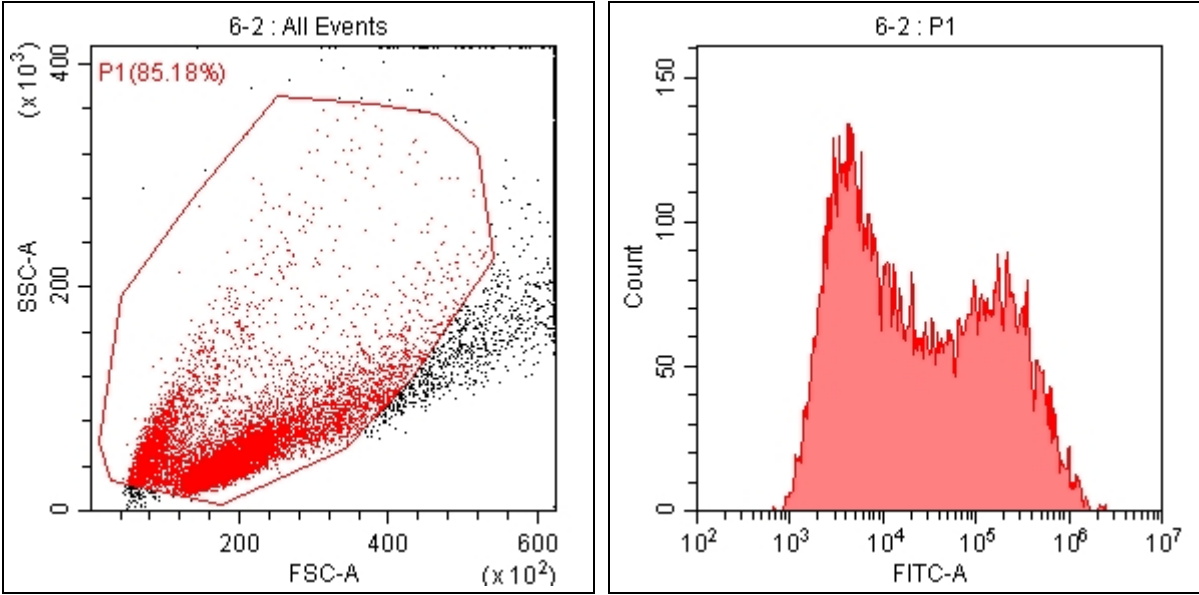

试管名称： 6-2  
样本ID：

| 群体           | %父群     | Mean FITC-A |
|--------------|---------|-------------|
| ● All Events | 100.00% | 144559.4    |
| ● P1         | 85.18%  | 107051.9    |

Supplement: Supplementary file 8 [file DataSheet_8.zip › fig 7-D raw/6-2.pdf]

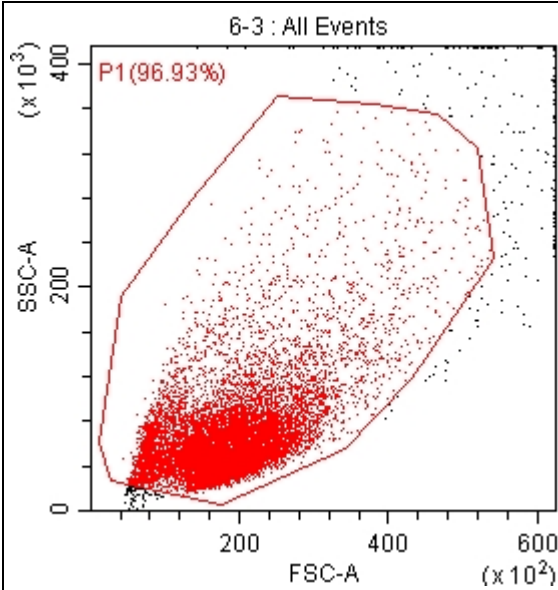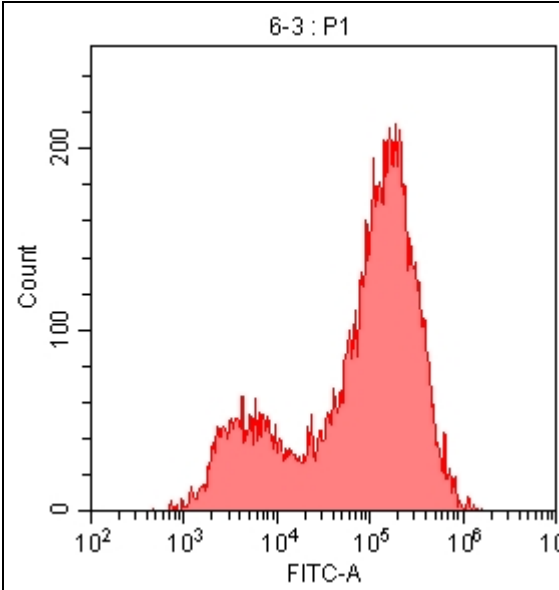

试管名称: 6-3  
样本ID:

| 群体           | %父群     | Mean FITC-A |
|--------------|---------|-------------|
| ● All Events | 100.00% | 151344.3    |
| ● P1         | 96.93%  | 145583.6    |

Supplement: Supplementary file 8 [file DataSheet_8.zip › fig 7-D raw/6-3.pdf]
