# Supplementary material for: Regulation of microglia related neuroinflammation contributes to the protective effect of Gelsevirine on ischemic stroke
Source: Front Immunol. 2023 Mar 30;14:1164278. doi: 10.3389/fimmu.2023.1164278 (PMC10098192; doi:10.3389/fimmu.2023.1164278)
Supplement: Supplementary file 4 [file DataSheet_4.zip › fig 4-B raw/fig 4-B raw.zip!/fig 4-B raw/1-3.pdf]

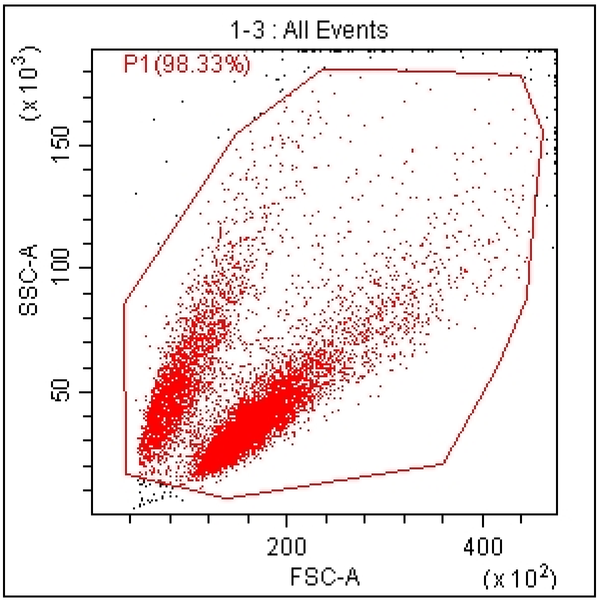

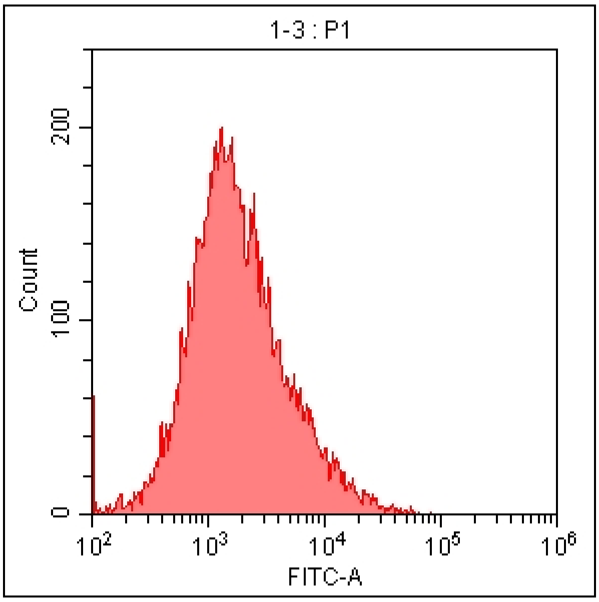

试管名称： 1-3

样本ID：

群体	%父群	Mean FITC-A
All Events	100.00%	3325.8
P1	98.33%	3159.0
