# Supplementary material for: Regulation of microglia related neuroinflammation contributes to the protective effect of Gelsevirine on ischemic stroke
Source: Front Immunol. 2023 Mar 30;14:1164278. doi: 10.3389/fimmu.2023.1164278 (PMC10098192; doi:10.3389/fimmu.2023.1164278)
Supplement: Supplementary file 4 [file DataSheet_4.zip › fig 4-B raw/fig 4-B raw.zip!/fig 4-B raw/5-2.pdf]

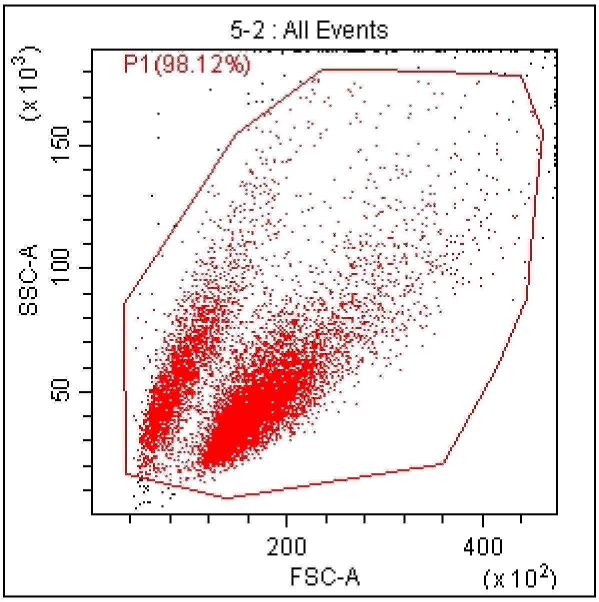

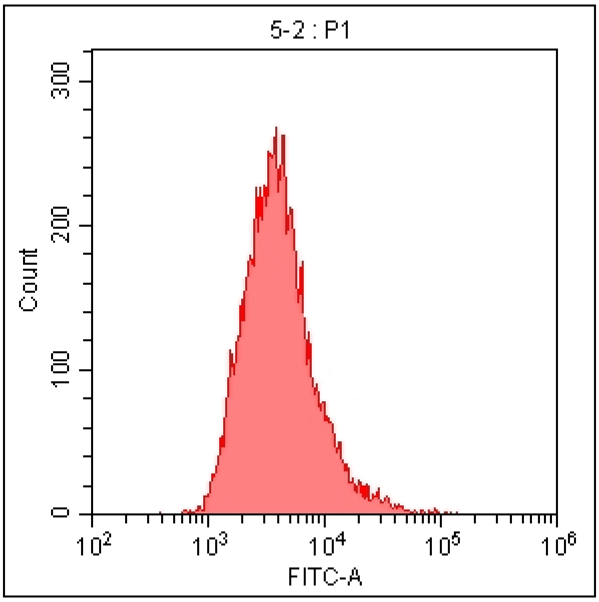

试管名称： 5-2
样本ID：

群体	%父群	Mean FITC-A
All Events	100.00%	6472.8
P1	98.12%	6083.2
