# Supplementary material for: Regulation of microglia related neuroinflammation contributes to the protective effect of Gelsevirine on ischemic stroke
Source: Front Immunol. 2023 Mar 30;14:1164278. doi: 10.3389/fimmu.2023.1164278 (PMC10098192; doi:10.3389/fimmu.2023.1164278)
Supplement: Supplementary file 6 [file DataSheet_6.zip › fig 5 raw/fig 5-G raw/inflammation.Gsea.1649955060129/HALLMARK_MTORC1_SIGNALING.html]

Details for gene set HALLMARK\_MTORC1\_SIGNALING[GSEA]

|  || Dataset | OGD\_DRUG\_DRUG.OGD\_FRUG.cls#Gs\_versus\_MCAO.OGD\_FRUG.cls#Gs\_versus\_MCAO\_repos |
| Phenotype | OGD\_FRUG.cls#Gs\_versus\_MCAO\_repos |
| Upregulated in class | Gs |
| GeneSet | HALLMARK\_MTORC1\_SIGNALING |
| Enrichment Score (ES) | 0.41941133 |
| Normalized Enrichment Score (NES) | 1.5025332 |
| Nominal p-value | 0.0 |
| FDR q-value | 0.098793894 |
| FWER p-Value | 0.598 |
Table: GSEA Results Summary

  

Fig 1: Enrichment plot: HALLMARK\_MTORC1\_SIGNALING      
 Profile of the Running ES Score & Positions of GeneSet Members on the Rank Ordered List

  

| SYMBOL | TITLE | RANK IN GENE LIST | RANK METRIC SCORE | RUNNING ES | CORE ENRICHMENT || 1 | HSPA5 | na | 265 | 0.727 | 0.0056 | Yes |
| 2 | UCHL5 | na | 488 | 0.592 | 0.0098 | Yes |
| 3 | DDIT3 | na | 490 | 0.591 | 0.0242 | Yes |
| 4 | PRDX1 | na | 562 | 0.567 | 0.0348 | Yes |
| 5 | PPP1R15A | na | 573 | 0.564 | 0.0481 | Yes |
| 6 | HSPE1 | na | 704 | 0.525 | 0.0549 | Yes |
| 7 | ATP5G1 | na | 718 | 0.521 | 0.0671 | Yes |
| 8 | FKBP2 | na | 975 | 0.468 | 0.0667 | Yes |
| 9 | NUPR1 | na | 1089 | 0.451 | 0.0725 | Yes |
| 10 | HSP90B1 | na | 1143 | 0.443 | 0.0809 | Yes |
| 11 | UFM1 | na | 1170 | 0.438 | 0.0904 | Yes |
| 12 | PSMA3 | na | 1229 | 0.431 | 0.0983 | Yes |
| 13 | SDF2L1 | na | 1249 | 0.428 | 0.1078 | Yes |
| 14 | SLC2A3 | na | 1264 | 0.426 | 0.1176 | Yes |
| 15 | ARPC5L | na | 1304 | 0.421 | 0.1261 | Yes |
| 16 | PSMA4 | na | 1348 | 0.415 | 0.1343 | Yes |
| 17 | EIF2S2 | na | 1399 | 0.411 | 0.1420 | Yes |
| 18 | EBP | na | 1463 | 0.404 | 0.1490 | Yes |
| 19 | SEC11A | na | 1523 | 0.397 | 0.1560 | Yes |
| 20 | GAPDH | na | 1541 | 0.395 | 0.1648 | Yes |
| 21 | PSPH | na | 1570 | 0.391 | 0.1731 | Yes |
| 22 | PSMC6 | na | 1625 | 0.383 | 0.1800 | Yes |
| 23 | GLA | na | 1673 | 0.377 | 0.1871 | Yes |
| 24 | CACYBP | na | 1726 | 0.371 | 0.1937 | Yes |
| 25 | PSMD14 | na | 1739 | 0.369 | 0.2022 | Yes |
| 26 | NFKBIB | na | 1764 | 0.366 | 0.2100 | Yes |
| 27 | CDKN1A | na | 1773 | 0.366 | 0.2186 | Yes |
| 28 | PSMG1 | na | 1794 | 0.363 | 0.2265 | Yes |
| 29 | HMBS | na | 1951 | 0.344 | 0.2277 | Yes |
| 30 | CALR | na | 2061 | 0.331 | 0.2308 | Yes |
| 31 | XBP1 | na | 2070 | 0.331 | 0.2385 | Yes |
| 32 | NUFIP1 | na | 2074 | 0.330 | 0.2465 | Yes |
| 33 | TRIB3 | na | 2091 | 0.329 | 0.2538 | Yes |
| 34 | PSMC2 | na | 2133 | 0.323 | 0.2598 | Yes |
| 35 | COPS5 | na | 2152 | 0.321 | 0.2668 | Yes |
| 36 | DDIT4 | na | 2226 | 0.313 | 0.2711 | Yes |
| 37 | PNP | na | 2244 | 0.310 | 0.2779 | Yes |
| 38 | IFI30 | na | 2266 | 0.308 | 0.2844 | Yes |
| 39 | ATP6V1D | na | 2307 | 0.303 | 0.2900 | Yes |
| 40 | PPIA | na | 2332 | 0.301 | 0.2963 | Yes |
| 41 | PSMB5 | na | 2587 | 0.279 | 0.2914 | Yes |
| 42 | PSMC4 | na | 2617 | 0.277 | 0.2968 | Yes |
| 43 | DHFR | na | 2621 | 0.277 | 0.3035 | Yes |
| 44 | PSMD13 | na | 2804 | 0.259 | 0.3014 | Yes |
| 45 | PSMD12 | na | 2816 | 0.258 | 0.3072 | Yes |
| 46 | QDPR | na | 2840 | 0.256 | 0.3124 | Yes |
| 47 | BTG2 | na | 2846 | 0.255 | 0.3184 | Yes |
| 48 | ASNS | na | 2862 | 0.254 | 0.3239 | Yes |
| 49 | PPA1 | na | 2866 | 0.254 | 0.3300 | Yes |
| 50 | IFRD1 | na | 2895 | 0.251 | 0.3348 | Yes |
| 51 | GOT1 | na | 2969 | 0.244 | 0.3374 | Yes |
| 52 | DAPP1 | na | 3038 | 0.238 | 0.3401 | Yes |
| 53 | SQSTM1 | na | 3060 | 0.236 | 0.3449 | Yes |
| 54 | CTH | na | 3066 | 0.236 | 0.3504 | Yes |
| 55 | LDHA | na | 3144 | 0.229 | 0.3525 | Yes |
| 56 | RAB1A | na | 3153 | 0.228 | 0.3577 | Yes |
| 57 | UNG | na | 3184 | 0.225 | 0.3618 | Yes |
| 58 | PHGDH | na | 3203 | 0.223 | 0.3664 | Yes |
| 59 | CD9 | na | 3224 | 0.220 | 0.3709 | Yes |
| 60 | TUBA4A | na | 3330 | 0.212 | 0.3712 | Yes |
| 61 | GBE1 | na | 3373 | 0.208 | 0.3744 | Yes |
| 62 | CORO1A | na | 3378 | 0.208 | 0.3793 | Yes |
| 63 | RIT1 | na | 3400 | 0.207 | 0.3834 | Yes |
| 64 | LGMN | na | 3411 | 0.206 | 0.3879 | Yes |
| 65 | UBE2D3 | na | 3444 | 0.203 | 0.3914 | Yes |
| 66 | PDAP1 | na | 3448 | 0.203 | 0.3962 | Yes |
| 67 | VLDLR | na | 3491 | 0.200 | 0.3992 | Yes |
| 68 | SLC7A11 | na | 3501 | 0.199 | 0.4036 | Yes |
| 69 | EEF1E1 | na | 3534 | 0.197 | 0.4070 | Yes |
| 70 | MTHFD2 | na | 3675 | 0.187 | 0.4051 | Yes |
| 71 | AURKA | na | 3721 | 0.185 | 0.4075 | Yes |
| 72 | TOMM40 | na | 3847 | 0.177 | 0.4061 | Yes |
| 73 | RRP9 | na | 4073 | 0.158 | 0.3996 | Yes |
| 74 | PGK1 | na | 4074 | 0.158 | 0.4034 | Yes |
| 75 | HSPD1 | na | 4188 | 0.150 | 0.4019 | Yes |
| 76 | SERP1 | na | 4202 | 0.149 | 0.4049 | Yes |
| 77 | RPN1 | na | 4205 | 0.149 | 0.4085 | Yes |
| 78 | SORD | na | 4237 | 0.147 | 0.4106 | Yes |
| 79 | HSPA9 | na | 4272 | 0.145 | 0.4126 | Yes |
| 80 | NFIL3 | na | 4292 | 0.143 | 0.4152 | Yes |
| 81 | PNO1 | na | 4339 | 0.140 | 0.4165 | Yes |
| 82 | M6PR | na | 4351 | 0.139 | 0.4194 | Yes |
| 83 | TMEM97 | na | 4449 | 0.133 | 0.4182 | No |
| 84 | FDXR | na | 4509 | 0.128 | 0.4186 | No |
| 85 | PSAT1 | na | 4682 | 0.116 | 0.4135 | No |
| 86 | HSPA4 | na | 4740 | 0.112 | 0.4136 | No |
| 87 | TCEA1 | na | 4818 | 0.107 | 0.4127 | No |
| 88 | IDH1 | na | 4951 | 0.098 | 0.4090 | No |
| 89 | MLLT11 | na | 4981 | 0.097 | 0.4100 | No |
| 90 | YKT6 | na | 4985 | 0.096 | 0.4122 | No |
| 91 | TPI1 | na | 5033 | 0.094 | 0.4124 | No |
| 92 | MAP2K3 | na | 5094 | 0.091 | 0.4118 | No |
| 93 | ALDOA | na | 5121 | 0.089 | 0.4128 | No |
| 94 | ACSL3 | na | 5200 | 0.085 | 0.4113 | No |
| 95 | BHLHE40 | na | 5305 | 0.079 | 0.4084 | No |
| 96 | SSR1 | na | 5344 | 0.077 | 0.4085 | No |
| 97 | SRD5A1 | na | 5424 | 0.072 | 0.4066 | No |
| 98 | CCT6A | na | 5468 | 0.070 | 0.4064 | No |
| 99 | IGFBP5 | na | 5917 | 0.046 | 0.3868 | No |
| 100 | ETF1 | na | 5959 | 0.044 | 0.3860 | No |
| 101 | ENO1 | na | 6025 | 0.039 | 0.3840 | No |
| 102 | LTA4H | na | 6046 | 0.038 | 0.3840 | No |
| 103 | STIP1 | na | 6080 | 0.037 | 0.3834 | No |
| 104 | RRM2 | na | 6142 | 0.035 | 0.3814 | No |
| 105 | GLRX | na | 6293 | 0.029 | 0.3752 | No |
| 106 | STC1 | na | 6434 | 0.023 | 0.3693 | No |
| 107 | CANX | na | 6437 | 0.023 | 0.3698 | No |
| 108 | RDH11 | na | 6485 | 0.021 | 0.3681 | No |
| 109 | CCNG1 | na | 6499 | 0.021 | 0.3681 | No |
| 110 | GMPS | na | 6561 | 0.018 | 0.3657 | No |
| 111 | NMT1 | na | 6707 | 0.013 | 0.3593 | No |
| 112 | EDEM1 | na | 6717 | 0.012 | 0.3592 | No |
| 113 | WARS | na | 6733 | 0.011 | 0.3588 | No |
| 114 | IMMT | na | 6823 | 0.009 | 0.3549 | No |
| 115 | RPA1 | na | 6886 | 0.006 | 0.3522 | No |
| 116 | TES | na | 6939 | 0.004 | 0.3499 | No |
| 117 | GTF2H1 | na | 6988 | 0.002 | 0.3477 | No |
| 118 | SHMT2 | na | 7002 | 0.001 | 0.3471 | No |
| 119 | CXCR4 | na | 9111 | 0.000 | 0.2499 | No |
| 120 | GGA2 | na | 13397 | -0.005 | 0.0524 | No |
| 121 | CTSC | na | 13557 | -0.008 | 0.0453 | No |
| 122 | NFYC | na | 13667 | -0.011 | 0.0405 | No |
| 123 | ABCF2 | na | 13679 | -0.012 | 0.0403 | No |
| 124 | BUB1 | na | 13776 | -0.013 | 0.0362 | No |
| 125 | PITPNB | na | 13780 | -0.014 | 0.0364 | No |
| 126 | TBK1 | na | 13939 | -0.018 | 0.0296 | No |
| 127 | CFP | na | 14087 | -0.023 | 0.0233 | No |
| 128 | ME1 | na | 14096 | -0.023 | 0.0236 | No |
| 129 | CDC25A | na | 14124 | -0.024 | 0.0229 | No |
| 130 | FGL2 | na | 14127 | -0.024 | 0.0234 | No |
| 131 | MTHFD2L | na | 14173 | -0.027 | 0.0220 | No |
| 132 | BCAT1 | na | 14208 | -0.028 | 0.0211 | No |
| 133 | SLC1A5 | na | 14226 | -0.029 | 0.0210 | No |
| 134 | PGM1 | na | 14386 | -0.035 | 0.0146 | No |
| 135 | NAMPT | na | 14416 | -0.036 | 0.0141 | No |
| 136 | TM7SF2 | na | 14681 | -0.050 | 0.0031 | No |
| 137 | INSIG1 | na | 14787 | -0.055 | -0.0003 | No |
| 138 | SERPINH1 | na | 14856 | -0.059 | -0.0020 | No |
| 139 | FADS1 | na | 14858 | -0.059 | -0.0006 | No |
| 140 | TUBG1 | na | 14895 | -0.062 | -0.0008 | No |
| 141 | POLR3G | na | 14958 | -0.065 | -0.0020 | No |
| 142 | PFKL | na | 15100 | -0.074 | -0.0067 | No |
| 143 | ACTR3 | na | 15104 | -0.074 | -0.0051 | No |
| 144 | FAM129A | na | 15128 | -0.076 | -0.0043 | No |
| 145 | SQLE | na | 15231 | -0.082 | -0.0070 | No |
| 146 | HMGCS1 | na | 15399 | -0.093 | -0.0124 | No |
| 147 | GSR | na | 15404 | -0.093 | -0.0104 | No |
| 148 | STARD4 | na | 15447 | -0.095 | -0.0100 | No |
| 149 | ACTR2 | na | 15570 | -0.102 | -0.0131 | No |
| 150 | SLC37A4 | na | 15646 | -0.106 | -0.0140 | No |
| 151 | ERO1L | na | 15732 | -0.111 | -0.0152 | No |
| 152 | SLC1A4 | na | 15763 | -0.113 | -0.0138 | No |
| 153 | TXNRD1 | na | 15772 | -0.113 | -0.0114 | No |
| 154 | P4HA1 | na | 15784 | -0.114 | -0.0091 | No |
| 155 | SKAP2 | na | 15873 | -0.120 | -0.0103 | No |
| 156 | ELOVL5 | na | 15983 | -0.127 | -0.0122 | No |
| 157 | EGLN3 | na | 16115 | -0.136 | -0.0149 | No |
| 158 | MCM4 | na | 16168 | -0.139 | -0.0139 | No |
| 159 | SLC2A1 | na | 16405 | -0.155 | -0.0210 | No |
| 160 | PSME3 | na | 16442 | -0.157 | -0.0189 | No |
| 161 | EPRS | na | 16551 | -0.165 | -0.0198 | No |
| 162 | USO1 | na | 16738 | -0.179 | -0.0240 | No |
| 163 | SLC9A3R1 | na | 16785 | -0.181 | -0.0217 | No |
| 164 | CYB5B | na | 16850 | -0.186 | -0.0201 | No |
| 165 | PLOD2 | na | 16918 | -0.190 | -0.0185 | No |
| 166 | SLC7A5 | na | 17480 | -0.229 | -0.0388 | No |
| 167 | SLA | na | 17503 | -0.230 | -0.0342 | No |
| 168 | ITGB2 | na | 17658 | -0.242 | -0.0354 | No |
| 169 | DHCR7 | na | 17666 | -0.242 | -0.0298 | No |
| 170 | CCNF | na | 17678 | -0.243 | -0.0244 | No |
| 171 | AK4 | na | 17779 | -0.251 | -0.0229 | No |
| 172 | DHCR24 | na | 17938 | -0.265 | -0.0237 | No |
| 173 | MCM2 | na | 17947 | -0.266 | -0.0176 | No |
| 174 | GCLC | na | 17950 | -0.266 | -0.0111 | No |
| 175 | PLK1 | na | 17988 | -0.269 | -0.0063 | No |
| 176 | HK2 | na | 18028 | -0.271 | -0.0015 | No |
| 177 | ATP2A2 | na | 18364 | -0.295 | -0.0097 | No |
| 178 | TFRC | na | 18581 | -0.311 | -0.0121 | No |
| 179 | ELOVL6 | na | 18683 | -0.319 | -0.0089 | No |
| 180 | ADIPOR2 | na | 18835 | -0.333 | -0.0078 | No |
| 181 | HMGCR | na | 18840 | -0.333 | 0.0002 | No |
| 182 | PDK1 | na | 19001 | -0.349 | 0.0014 | No |
| 183 | PIK3R3 | na | 19336 | -0.380 | -0.0048 | No |
| 184 | ACLY | na | 19638 | -0.410 | -0.0086 | No |
| 185 | NUP205 | na | 19751 | -0.421 | -0.0035 | No |
| 186 | GSK3B | na | 20096 | -0.455 | -0.0082 | No |
| 187 | FADS2 | na | 20280 | -0.475 | -0.0050 | No |
| 188 | LDLR | na | 20352 | -0.483 | 0.0035 | No |
| 189 | IDI1 | na | 20363 | -0.484 | 0.0149 | No |
| 190 | SLC6A6 | na | 20639 | -0.521 | 0.0149 | No |
| 191 | ADD3 | na | 20650 | -0.522 | 0.0272 | No |
| 192 | SYTL2 | na | 21001 | -0.579 | 0.0252 | No |
| 193 | ACACA | na | 21178 | -0.614 | 0.0321 | No |
Table: GSEA details [plain text format]

  

Fig 2: HALLMARK\_MTORC1\_SIGNALING      
 Blue-Pink O' Gram in the Space of the Analyzed GeneSet

  

Fig 3: HALLMARK\_MTORC1\_SIGNALING: Random ES distribution      
 Gene set null distribution of ES for **HALLMARK\_MTORC1\_SIGNALING**
