# Supplementary material for: Regulation of microglia related neuroinflammation contributes to the protective effect of Gelsevirine on ischemic stroke
Source: Front Immunol. 2023 Mar 30;14:1164278. doi: 10.3389/fimmu.2023.1164278 (PMC10098192; doi:10.3389/fimmu.2023.1164278)
Supplement: Supplementary file 6 [file DataSheet_6.zip › fig 5 raw/fig 5-G raw/inflammation.Gsea.1649955060129/HALLMARK_MYC_TARGETS_V1.html]

Details for gene set HALLMARK\_MYC\_TARGETS\_V1[GSEA]

|  || Dataset | OGD\_DRUG\_DRUG.OGD\_FRUG.cls#Gs\_versus\_MCAO.OGD\_FRUG.cls#Gs\_versus\_MCAO\_repos |
| Phenotype | OGD\_FRUG.cls#Gs\_versus\_MCAO\_repos |
| Upregulated in class | Gs |
| GeneSet | HALLMARK\_MYC\_TARGETS\_V1 |
| Enrichment Score (ES) | 0.6159267 |
| Normalized Enrichment Score (NES) | 2.2096124 |
| Nominal p-value | 0.0 |
| FDR q-value | 0.0 |
| FWER p-Value | 0.0 |
Table: GSEA Results Summary

  

Fig 1: Enrichment plot: HALLMARK\_MYC\_TARGETS\_V1      
 Profile of the Running ES Score & Positions of GeneSet Members on the Rank Ordered List

  

| SYMBOL | TITLE | RANK IN GENE LIST | RANK METRIC SCORE | RUNNING ES | CORE ENRICHMENT || 1 | LSM7 | na | 400 | 0.631 | -0.0056 | Yes |
| 2 | COX5A | na | 462 | 0.602 | 0.0038 | Yes |
| 3 | EEF1B2 | na | 504 | 0.588 | 0.0139 | Yes |
| 4 | RPL14 | na | 508 | 0.587 | 0.0257 | Yes |
| 5 | RPS3 | na | 511 | 0.586 | 0.0375 | Yes |
| 6 | RPL22 | na | 517 | 0.582 | 0.0491 | Yes |
| 7 | RPS6 | na | 566 | 0.566 | 0.0585 | Yes |
| 8 | RPL34 | na | 691 | 0.529 | 0.0635 | Yes |
| 9 | SNRPG | na | 703 | 0.525 | 0.0737 | Yes |
| 10 | HSPE1 | na | 704 | 0.525 | 0.0844 | Yes |
| 11 | PSMA6 | na | 735 | 0.516 | 0.0935 | Yes |
| 12 | RFC4 | na | 748 | 0.511 | 0.1033 | Yes |
| 13 | SNRPD2 | na | 767 | 0.507 | 0.1128 | Yes |
| 14 | EIF1AX | na | 780 | 0.503 | 0.1225 | Yes |
| 15 | SNRPD1 | na | 781 | 0.502 | 0.1327 | Yes |
| 16 | ERH | na | 804 | 0.497 | 0.1418 | Yes |
| 17 | RPL6 | na | 808 | 0.497 | 0.1518 | Yes |
| 18 | NME1 | na | 818 | 0.494 | 0.1614 | Yes |
| 19 | DUT | na | 840 | 0.490 | 0.1704 | Yes |
| 20 | PSMB3 | na | 861 | 0.484 | 0.1794 | Yes |
| 21 | SNRPB2 | na | 949 | 0.471 | 0.1849 | Yes |
| 22 | SNRPD3 | na | 993 | 0.466 | 0.1924 | Yes |
| 23 | APEX1 | na | 1088 | 0.451 | 0.1973 | Yes |
| 24 | VBP1 | na | 1092 | 0.450 | 0.2063 | Yes |
| 25 | AIMP2 | na | 1113 | 0.446 | 0.2145 | Yes |
| 26 | VDAC3 | na | 1114 | 0.446 | 0.2235 | Yes |
| 27 | NHP2 | na | 1120 | 0.445 | 0.2324 | Yes |
| 28 | RPL18 | na | 1139 | 0.443 | 0.2406 | Yes |
| 29 | HDDC2 | na | 1151 | 0.441 | 0.2490 | Yes |
| 30 | MRPL23 | na | 1184 | 0.437 | 0.2564 | Yes |
| 31 | RPS5 | na | 1199 | 0.434 | 0.2646 | Yes |
| 32 | SSBP1 | na | 1212 | 0.433 | 0.2729 | Yes |
| 33 | RPS10 | na | 1307 | 0.420 | 0.2771 | Yes |
| 34 | UBE2E1 | na | 1317 | 0.418 | 0.2852 | Yes |
| 35 | PRDX4 | na | 1328 | 0.417 | 0.2932 | Yes |
| 36 | PSMA7 | na | 1331 | 0.417 | 0.3016 | Yes |
| 37 | PSMA4 | na | 1348 | 0.415 | 0.3093 | Yes |
| 38 | SNRPA1 | na | 1357 | 0.414 | 0.3174 | Yes |
| 39 | NPM1 | na | 1388 | 0.412 | 0.3244 | Yes |
| 40 | EIF2S2 | na | 1399 | 0.411 | 0.3323 | Yes |
| 41 | PSMA2 | na | 1522 | 0.398 | 0.3348 | Yes |
| 42 | U2AF1 | na | 1531 | 0.396 | 0.3425 | Yes |
| 43 | RANBP1 | na | 1551 | 0.393 | 0.3496 | Yes |
| 44 | PSMC6 | na | 1625 | 0.383 | 0.3540 | Yes |
| 45 | NOP16 | na | 1708 | 0.373 | 0.3578 | Yes |
| 46 | PSMB2 | na | 1712 | 0.373 | 0.3653 | Yes |
| 47 | PSMD14 | na | 1739 | 0.369 | 0.3716 | Yes |
| 48 | PSMA1 | na | 1854 | 0.356 | 0.3736 | Yes |
| 49 | NAP1L1 | na | 1858 | 0.355 | 0.3807 | Yes |
| 50 | SRSF3 | na | 1888 | 0.351 | 0.3865 | Yes |
| 51 | SLC25A3 | na | 1903 | 0.348 | 0.3929 | Yes |
| 52 | CNBP | na | 1932 | 0.345 | 0.3987 | Yes |
| 53 | EIF4A1 | na | 1958 | 0.343 | 0.4045 | Yes |
| 54 | LSM2 | na | 2035 | 0.334 | 0.4078 | Yes |
| 55 | TXNL4A | na | 2046 | 0.333 | 0.4141 | Yes |
| 56 | PHB | na | 2060 | 0.331 | 0.4202 | Yes |
| 57 | COPS5 | na | 2152 | 0.321 | 0.4226 | Yes |
| 58 | RPS2 | na | 2161 | 0.321 | 0.4287 | Yes |
| 59 | SRSF7 | na | 2163 | 0.321 | 0.4352 | Yes |
| 60 | SSB | na | 2165 | 0.320 | 0.4417 | Yes |
| 61 | NOP56 | na | 2233 | 0.312 | 0.4449 | Yes |
| 62 | PHB2 | na | 2240 | 0.311 | 0.4510 | Yes |
| 63 | TYMS | na | 2246 | 0.310 | 0.4570 | Yes |
| 64 | PPIA | na | 2332 | 0.301 | 0.4593 | Yes |
| 65 | PTGES3 | na | 2335 | 0.301 | 0.4653 | Yes |
| 66 | RSL1D1 | na | 2341 | 0.301 | 0.4712 | Yes |
| 67 | EIF2S1 | na | 2354 | 0.299 | 0.4767 | Yes |
| 68 | FBL | na | 2355 | 0.299 | 0.4828 | Yes |
| 69 | PSMD7 | na | 2378 | 0.297 | 0.4878 | Yes |
| 70 | CYC1 | na | 2402 | 0.296 | 0.4928 | Yes |
| 71 | SET | na | 2415 | 0.295 | 0.4982 | Yes |
| 72 | HNRNPC | na | 2434 | 0.293 | 0.5034 | Yes |
| 73 | SERBP1 | na | 2465 | 0.290 | 0.5079 | Yes |
| 74 | NDUFAB1 | na | 2476 | 0.289 | 0.5133 | Yes |
| 75 | GNL3 | na | 2481 | 0.288 | 0.5190 | Yes |
| 76 | RAN | na | 2563 | 0.281 | 0.5210 | Yes |
| 77 | GLO1 | na | 2586 | 0.280 | 0.5256 | Yes |
| 78 | PSMC4 | na | 2617 | 0.277 | 0.5299 | Yes |
| 79 | EXOSC7 | na | 2704 | 0.269 | 0.5314 | Yes |
| 80 | CCT3 | na | 2732 | 0.266 | 0.5356 | Yes |
| 81 | C1QBP | na | 2747 | 0.264 | 0.5403 | Yes |
| 82 | UBE2L3 | na | 2794 | 0.260 | 0.5435 | Yes |
| 83 | PCNA | na | 2828 | 0.257 | 0.5472 | Yes |
| 84 | HDAC2 | na | 2882 | 0.251 | 0.5498 | Yes |
| 85 | IFRD1 | na | 2895 | 0.251 | 0.5544 | Yes |
| 86 | CLNS1A | na | 2935 | 0.247 | 0.5576 | Yes |
| 87 | CCT7 | na | 2943 | 0.247 | 0.5623 | Yes |
| 88 | IMPDH2 | na | 2955 | 0.245 | 0.5668 | Yes |
| 89 | MRPL9 | na | 2958 | 0.245 | 0.5717 | Yes |
| 90 | CCT2 | na | 2987 | 0.243 | 0.5753 | Yes |
| 91 | PSMD8 | na | 2992 | 0.242 | 0.5801 | Yes |
| 92 | TCP1 | na | 3077 | 0.235 | 0.5810 | Yes |
| 93 | ILF2 | na | 3106 | 0.233 | 0.5844 | Yes |
| 94 | LDHA | na | 3144 | 0.229 | 0.5874 | Yes |
| 95 | HNRNPD | na | 3195 | 0.224 | 0.5896 | Yes |
| 96 | CCT4 | na | 3202 | 0.223 | 0.5939 | Yes |
| 97 | CCT5 | na | 3233 | 0.220 | 0.5970 | Yes |
| 98 | CDK4 | na | 3240 | 0.219 | 0.6012 | Yes |
| 99 | HNRNPA2B1 | na | 3284 | 0.216 | 0.6036 | Yes |
| 100 | NCBP2 | na | 3367 | 0.209 | 0.6041 | Yes |
| 101 | POLE3 | na | 3398 | 0.207 | 0.6069 | Yes |
| 102 | HDGF | na | 3405 | 0.206 | 0.6108 | Yes |
| 103 | PA2G4 | na | 3489 | 0.200 | 0.6110 | Yes |
| 104 | AP3S1 | na | 3816 | 0.179 | 0.5996 | Yes |
| 105 | RPLP0 | na | 3826 | 0.178 | 0.6028 | Yes |
| 106 | MRPS18B | na | 3890 | 0.174 | 0.6035 | Yes |
| 107 | SRM | na | 3965 | 0.167 | 0.6035 | Yes |
| 108 | HNRNPA3 | na | 3970 | 0.167 | 0.6067 | Yes |
| 109 | NOLC1 | na | 4055 | 0.159 | 0.6061 | Yes |
| 110 | RRP9 | na | 4073 | 0.158 | 0.6085 | Yes |
| 111 | PGK1 | na | 4074 | 0.158 | 0.6117 | Yes |
| 112 | PRDX3 | na | 4086 | 0.157 | 0.6144 | Yes |
| 113 | KPNA2 | na | 4158 | 0.153 | 0.6142 | Yes |
| 114 | HSPD1 | na | 4188 | 0.150 | 0.6159 | Yes |
| 115 | YWHAE | na | 4295 | 0.143 | 0.6139 | No |
| 116 | EIF4E | na | 4398 | 0.137 | 0.6120 | No |
| 117 | EIF3D | na | 4572 | 0.124 | 0.6066 | No |
| 118 | DEK | na | 4586 | 0.123 | 0.6085 | No |
| 119 | XRCC6 | na | 4604 | 0.122 | 0.6102 | No |
| 120 | H2AFZ | na | 4645 | 0.119 | 0.6107 | No |
| 121 | MAD2L1 | na | 4742 | 0.112 | 0.6086 | No |
| 122 | SRSF2 | na | 4990 | 0.096 | 0.5991 | No |
| 123 | EIF4H | na | 5011 | 0.095 | 0.6002 | No |
| 124 | KARS | na | 5055 | 0.093 | 0.6001 | No |
| 125 | TRA2B | na | 5061 | 0.092 | 0.6017 | No |
| 126 | ACP1 | na | 5092 | 0.091 | 0.6022 | No |
| 127 | DDX18 | na | 5165 | 0.086 | 0.6006 | No |
| 128 | PSMD1 | na | 5203 | 0.085 | 0.6006 | No |
| 129 | HSP90AB1 | na | 5242 | 0.082 | 0.6006 | No |
| 130 | MCM7 | na | 5251 | 0.082 | 0.6019 | No |
| 131 | TRIM28 | na | 5263 | 0.081 | 0.6030 | No |
| 132 | HNRNPA1 | na | 5313 | 0.079 | 0.6023 | No |
| 133 | ORC2 | na | 5463 | 0.070 | 0.5969 | No |
| 134 | GSPT1 | na | 5488 | 0.069 | 0.5972 | No |
| 135 | ODC1 | na | 5509 | 0.068 | 0.5976 | No |
| 136 | MYC | na | 5587 | 0.064 | 0.5954 | No |
| 137 | TUFM | na | 5658 | 0.060 | 0.5934 | No |
| 138 | RUVBL2 | na | 5686 | 0.059 | 0.5933 | No |
| 139 | ETF1 | na | 5959 | 0.044 | 0.5817 | No |
| 140 | PABPC4 | na | 5975 | 0.042 | 0.5818 | No |
| 141 | CTPS | na | 6077 | 0.037 | 0.5779 | No |
| 142 | SNRPA | na | 6081 | 0.037 | 0.5786 | No |
| 143 | TARDBP | na | 6085 | 0.037 | 0.5792 | No |
| 144 | VDAC1 | na | 6111 | 0.036 | 0.5787 | No |
| 145 | BUB3 | na | 6116 | 0.036 | 0.5793 | No |
| 146 | NCBP1 | na | 6154 | 0.034 | 0.5783 | No |
| 147 | CDC20 | na | 6204 | 0.032 | 0.5767 | No |
| 148 | CCNA2 | na | 6235 | 0.031 | 0.5759 | No |
| 149 | SRSF1 | na | 6341 | 0.027 | 0.5716 | No |
| 150 | CANX | na | 6437 | 0.023 | 0.5677 | No |
| 151 | G3BP1 | na | 6461 | 0.023 | 0.5671 | No |
| 152 | CDK2 | na | 6514 | 0.020 | 0.5651 | No |
| 153 | PRPF31 | na | 6572 | 0.018 | 0.5629 | No |
| 154 | TOMM70A | na | 6661 | 0.015 | 0.5591 | No |
| 155 | CDC45 | na | 6799 | 0.010 | 0.5530 | No |
| 156 | MCM6 | na | 6932 | 0.004 | 0.5470 | No |
| 157 | PSMD3 | na | 13262 | -0.000 | 0.2550 | No |
| 158 | RNPS1 | na | 14201 | -0.028 | 0.2123 | No |
| 159 | RAD23B | na | 14224 | -0.029 | 0.2119 | No |
| 160 | CUL1 | na | 14514 | -0.041 | 0.1994 | No |
| 161 | SYNCRIP | na | 14545 | -0.042 | 0.1989 | No |
| 162 | YWHAQ | na | 14606 | -0.046 | 0.1970 | No |
| 163 | UBA2 | na | 14649 | -0.048 | 0.1961 | No |
| 164 | STARD7 | na | 14832 | -0.058 | 0.1889 | No |
| 165 | POLD2 | na | 14951 | -0.065 | 0.1847 | No |
| 166 | HNRNPU | na | 15171 | -0.078 | 0.1762 | No |
| 167 | PABPC1 | na | 15181 | -0.079 | 0.1774 | No |
| 168 | PPM1G | na | 15270 | -0.084 | 0.1751 | No |
| 169 | CBX3 | na | 15297 | -0.086 | 0.1756 | No |
| 170 | EIF3B | na | 15316 | -0.087 | 0.1766 | No |
| 171 | GOT2 | na | 15431 | -0.094 | 0.1732 | No |
| 172 | DHX15 | na | 15439 | -0.094 | 0.1748 | No |
| 173 | PWP1 | na | 15476 | -0.096 | 0.1751 | No |
| 174 | USP1 | na | 15563 | -0.102 | 0.1732 | No |
| 175 | RRM1 | na | 15819 | -0.116 | 0.1638 | No |
| 176 | SRPK1 | na | 15842 | -0.118 | 0.1652 | No |
| 177 | HNRNPR | na | 15877 | -0.120 | 0.1661 | No |
| 178 | MCM4 | na | 16168 | -0.139 | 0.1555 | No |
| 179 | MCM5 | na | 16233 | -0.143 | 0.1555 | No |
| 180 | EPRS | na | 16551 | -0.165 | 0.1442 | No |
| 181 | DDX21 | na | 16652 | -0.173 | 0.1431 | No |
| 182 | PCBP1 | na | 16717 | -0.178 | 0.1438 | No |
| 183 | ABCE1 | na | 16732 | -0.178 | 0.1468 | No |
| 184 | XPO1 | na | 16765 | -0.180 | 0.1490 | No |
| 185 | EIF4G2 | na | 17082 | -0.201 | 0.1385 | No |
| 186 | KPNB1 | na | 17215 | -0.210 | 0.1367 | No |
| 187 | PRPS2 | na | 17293 | -0.216 | 0.1375 | No |
| 188 | MCM2 | na | 17947 | -0.266 | 0.1128 | No |
| 189 | TFDP1 | na | 18032 | -0.271 | 0.1145 | No |
| 190 | SF3B3 | na | 18079 | -0.274 | 0.1179 | No |
| 191 | XPOT | na | 18121 | -0.278 | 0.1217 | No |
| 192 | IARS | na | 18515 | -0.305 | 0.1098 | No |
| 193 | CSTF2 | na | 18546 | -0.308 | 0.1147 | No |
| 194 | SF3A1 | na | 19100 | -0.359 | 0.0965 | No |
| 195 | FAM120A | na | 19787 | -0.424 | 0.0735 | No |
| 196 | CAD | na | 20845 | -0.553 | 0.0360 | No |
| 197 | SMARCC1 | na | 20934 | -0.565 | 0.0434 | No |
Table: GSEA details [plain text format]

  

Fig 2: HALLMARK\_MYC\_TARGETS\_V1      
 Blue-Pink O' Gram in the Space of the Analyzed GeneSet

  

Fig 3: HALLMARK\_MYC\_TARGETS\_V1: Random ES distribution      
 Gene set null distribution of ES for **HALLMARK\_MYC\_TARGETS\_V1**
