# Supplementary material for: Regulation of microglia related neuroinflammation contributes to the protective effect of Gelsevirine on ischemic stroke
Source: Front Immunol. 2023 Mar 30;14:1164278. doi: 10.3389/fimmu.2023.1164278 (PMC10098192; doi:10.3389/fimmu.2023.1164278)
Supplement: Supplementary file 6 [file DataSheet_6.zip › fig 5 raw/fig 5-G raw/inflammation.Gsea.1649955060129/HALLMARK_MYC_TARGETS_V2.html]

Details for gene set HALLMARK\_MYC\_TARGETS\_V2[GSEA]

|  || Dataset | OGD\_DRUG\_DRUG.OGD\_FRUG.cls#Gs\_versus\_MCAO.OGD\_FRUG.cls#Gs\_versus\_MCAO\_repos |
| Phenotype | OGD\_FRUG.cls#Gs\_versus\_MCAO\_repos |
| Upregulated in class | Gs |
| GeneSet | HALLMARK\_MYC\_TARGETS\_V2 |
| Enrichment Score (ES) | 0.47657457 |
| Normalized Enrichment Score (NES) | 1.4330081 |
| Nominal p-value | 0.020231213 |
| FDR q-value | 0.11408007 |
| FWER p-Value | 0.785 |
Table: GSEA Results Summary

  

Fig 1: Enrichment plot: HALLMARK\_MYC\_TARGETS\_V2      
 Profile of the Running ES Score & Positions of GeneSet Members on the Rank Ordered List

  

| SYMBOL | TITLE | RANK IN GENE LIST | RANK METRIC SCORE | RUNNING ES | CORE ENRICHMENT || 1 | HSPE1 | na | 704 | 0.525 | 0.0140 | Yes |
| 2 | AIMP2 | na | 1113 | 0.446 | 0.0346 | Yes |
| 3 | EXOSC5 | na | 1215 | 0.433 | 0.0681 | Yes |
| 4 | NPM1 | na | 1388 | 0.412 | 0.0965 | Yes |
| 5 | WDR74 | na | 1661 | 0.379 | 0.1174 | Yes |
| 6 | NOP16 | na | 1708 | 0.373 | 0.1482 | Yes |
| 7 | MRTO4 | na | 1946 | 0.344 | 0.1677 | Yes |
| 8 | PHB | na | 2060 | 0.331 | 0.1917 | Yes |
| 9 | NDUFAF4 | na | 2122 | 0.324 | 0.2175 | Yes |
| 10 | NOP56 | na | 2233 | 0.312 | 0.2399 | Yes |
| 11 | NIP7 | na | 2296 | 0.304 | 0.2639 | Yes |
| 12 | GNL3 | na | 2481 | 0.288 | 0.2809 | Yes |
| 13 | DCTPP1 | na | 2678 | 0.272 | 0.2958 | Yes |
| 14 | PPAN | na | 2913 | 0.249 | 0.3070 | Yes |
| 15 | UNG | na | 3184 | 0.225 | 0.3145 | Yes |
| 16 | CDK4 | na | 3240 | 0.219 | 0.3313 | Yes |
| 17 | PA2G4 | na | 3489 | 0.200 | 0.3375 | Yes |
| 18 | MPHOSPH10 | na | 3504 | 0.199 | 0.3544 | Yes |
| 19 | PUS1 | na | 3507 | 0.199 | 0.3719 | Yes |
| 20 | GRWD1 | na | 3546 | 0.196 | 0.3874 | Yes |
| 21 | SRM | na | 3965 | 0.167 | 0.3830 | Yes |
| 22 | NOLC1 | na | 4055 | 0.159 | 0.3930 | Yes |
| 23 | RRP9 | na | 4073 | 0.158 | 0.4061 | Yes |
| 24 | HSPD1 | na | 4188 | 0.150 | 0.4141 | Yes |
| 25 | SORD | na | 4237 | 0.147 | 0.4248 | Yes |
| 26 | PES1 | na | 4259 | 0.146 | 0.4367 | Yes |
| 27 | TMEM97 | na | 4449 | 0.133 | 0.4398 | Yes |
| 28 | SLC29A2 | na | 4497 | 0.129 | 0.4490 | Yes |
| 29 | SLC19A1 | na | 4731 | 0.113 | 0.4483 | Yes |
| 30 | DUSP2 | na | 4863 | 0.104 | 0.4514 | Yes |
| 31 | LAS1L | na | 4907 | 0.101 | 0.4583 | Yes |
| 32 | TFB2M | na | 4952 | 0.098 | 0.4649 | Yes |
| 33 | WDR43 | na | 4967 | 0.097 | 0.4729 | Yes |
| 34 | DDX18 | na | 5165 | 0.086 | 0.4715 | Yes |
| 35 | FARSA | na | 5354 | 0.076 | 0.4696 | Yes |
| 36 | SUPV3L1 | na | 5357 | 0.076 | 0.4762 | Yes |
| 37 | MYC | na | 5587 | 0.064 | 0.4713 | Yes |
| 38 | RCL1 | na | 5651 | 0.060 | 0.4737 | Yes |
| 39 | PRMT3 | na | 5701 | 0.058 | 0.4766 | Yes |
| 40 | RABEPK | na | 5938 | 0.045 | 0.4697 | No |
| 41 | IMP4 | na | 6106 | 0.036 | 0.4652 | No |
| 42 | NOP2 | na | 6140 | 0.035 | 0.4668 | No |
| 43 | NOC4L | na | 6174 | 0.033 | 0.4682 | No |
| 44 | TBRG4 | na | 13346 | -0.003 | 0.1398 | No |
| 45 | MAP3K6 | na | 13722 | -0.013 | 0.1237 | No |
| 46 | CBX3 | na | 15297 | -0.086 | 0.0591 | No |
| 47 | RRP12 | na | 15971 | -0.127 | 0.0394 | No |
| 48 | IPO4 | na | 16029 | -0.130 | 0.0483 | No |
| 49 | MCM4 | na | 16168 | -0.139 | 0.0542 | No |
| 50 | MCM5 | na | 16233 | -0.143 | 0.0639 | No |
| 51 | PLK4 | na | 16699 | -0.177 | 0.0581 | No |
| 52 | BYSL | na | 16741 | -0.179 | 0.0720 | No |
| 53 | MYBBP1A | na | 17366 | -0.221 | 0.0629 | No |
| 54 | PLK1 | na | 17988 | -0.269 | 0.0581 | No |
| 55 | HK2 | na | 18028 | -0.271 | 0.0802 | No |
| 56 | TCOF1 | na | 18301 | -0.291 | 0.0934 | No |
| 57 | PPRC1 | na | 18530 | -0.306 | 0.1099 | No |
| 58 | UTP20 | na | 20423 | -0.492 | 0.0666 | No |
Table: GSEA details [plain text format]

  

Fig 2: HALLMARK\_MYC\_TARGETS\_V2      
 Blue-Pink O' Gram in the Space of the Analyzed GeneSet

  

Fig 3: HALLMARK\_MYC\_TARGETS\_V2: Random ES distribution      
 Gene set null distribution of ES for **HALLMARK\_MYC\_TARGETS\_V2**
