# Supplementary material for: Regulation of microglia related neuroinflammation contributes to the protective effect of Gelsevirine on ischemic stroke
Source: Front Immunol. 2023 Mar 30;14:1164278. doi: 10.3389/fimmu.2023.1164278 (PMC10098192; doi:10.3389/fimmu.2023.1164278)
Supplement: Supplementary file 6 [file DataSheet_6.zip › fig 5 raw/fig 5-G raw/inflammation.Gsea.1649955060129/HALLMARK_WNT_BETA_CATENIN_SIGNALING.html]

Details for gene set HALLMARK\_WNT\_BETA\_CATENIN\_SIGNALING[GSEA]

|  || Dataset | OGD\_DRUG\_DRUG.OGD\_FRUG.cls#Gs\_versus\_MCAO.OGD\_FRUG.cls#Gs\_versus\_MCAO\_repos |
| Phenotype | OGD\_FRUG.cls#Gs\_versus\_MCAO\_repos |
| Upregulated in class | MCAO |
| GeneSet | HALLMARK\_WNT\_BETA\_CATENIN\_SIGNALING |
| Enrichment Score (ES) | -0.5785677 |
| Normalized Enrichment Score (NES) | -1.500785 |
| Nominal p-value | 0.007518797 |
| FDR q-value | 0.095570624 |
| FWER p-Value | 0.703 |
Table: GSEA Results Summary

  

Fig 1: Enrichment plot: HALLMARK\_WNT\_BETA\_CATENIN\_SIGNALING      
 Profile of the Running ES Score & Positions of GeneSet Members on the Rank Ordered List

  

| SYMBOL | TITLE | RANK IN GENE LIST | RANK METRIC SCORE | RUNNING ES | CORE ENRICHMENT || 1 | HEY1 | na | 1247 | 0.428 | -0.0136 | No |
| 2 | PSEN2 | na | 1521 | 0.398 | 0.0142 | No |
| 3 | HDAC2 | na | 2882 | 0.251 | -0.0225 | No |
| 4 | GNAI1 | na | 4304 | 0.143 | -0.0731 | No |
| 5 | TCF7 | na | 4825 | 0.106 | -0.0862 | No |
| 6 | MYC | na | 5587 | 0.064 | -0.1145 | No |
| 7 | SKP2 | na | 5968 | 0.043 | -0.1276 | No |
| 8 | DKK1 | na | 7803 | 0.000 | -0.2116 | No |
| 9 | DKK4 | na | 7805 | 0.000 | -0.2116 | No |
| 10 | DLL1 | na | 7990 | 0.000 | -0.2200 | No |
| 11 | NKD1 | na | 8211 | 0.000 | -0.2301 | No |
| 12 | AXIN2 | na | 8241 | 0.000 | -0.2314 | No |
| 13 | FZD8 | na | 9035 | 0.000 | -0.2678 | No |
| 14 | WNT6 | na | 9610 | 0.000 | -0.2940 | No |
| 15 | HEY2 | na | 10436 | 0.000 | -0.3318 | No |
| 16 | CUL1 | na | 14514 | -0.041 | -0.5144 | No |
| 17 | WNT1 | na | 15037 | -0.070 | -0.5311 | No |
| 18 | CSNK1E | na | 15223 | -0.081 | -0.5314 | No |
| 19 | JAG2 | na | 15793 | -0.115 | -0.5458 | No |
| 20 | HDAC11 | na | 15853 | -0.118 | -0.5365 | No |
| 21 | LEF1 | na | 16773 | -0.181 | -0.5602 | Yes |
| 22 | KAT2A | na | 16776 | -0.181 | -0.5420 | Yes |
| 23 | WNT5B | na | 16806 | -0.183 | -0.5248 | Yes |
| 24 | NUMB | na | 16905 | -0.189 | -0.5100 | Yes |
| 25 | NCSTN | na | 17428 | -0.226 | -0.5110 | Yes |
| 26 | DVL2 | na | 17478 | -0.229 | -0.4901 | Yes |
| 27 | HDAC5 | na | 17656 | -0.241 | -0.4737 | Yes |
| 28 | PPARD | na | 17742 | -0.248 | -0.4525 | Yes |
| 29 | AXIN1 | na | 18098 | -0.276 | -0.4407 | Yes |
| 30 | CCND2 | na | 18282 | -0.290 | -0.4196 | Yes |
| 31 | CTNNB1 | na | 18285 | -0.290 | -0.3903 | Yes |
| 32 | ADAM17 | na | 18595 | -0.311 | -0.3728 | Yes |
| 33 | JAG1 | na | 19110 | -0.361 | -0.3598 | Yes |
| 34 | RBPJ | na | 19556 | -0.403 | -0.3393 | Yes |
| 35 | FZD1 | na | 19601 | -0.406 | -0.3001 | Yes |
| 36 | NOTCH1 | na | 20749 | -0.537 | -0.2981 | Yes |
| 37 | NOTCH4 | na | 20914 | -0.562 | -0.2485 | Yes |
| 38 | MAML1 | na | 21260 | -0.633 | -0.2001 | Yes |
| 39 | PTCH1 | na | 21269 | -0.636 | -0.1359 | Yes |
| 40 | FRAT1 | na | 21598 | -0.755 | -0.0743 | Yes |
| 41 | NCOR2 | na | 21713 | -0.856 | 0.0074 | Yes |
Table: GSEA details [plain text format]

  

Fig 2: HALLMARK\_WNT\_BETA\_CATENIN\_SIGNALING      
 Blue-Pink O' Gram in the Space of the Analyzed GeneSet

  

Fig 3: HALLMARK\_WNT\_BETA\_CATENIN\_SIGNALING: Random ES distribution      
 Gene set null distribution of ES for **HALLMARK\_WNT\_BETA\_CATENIN\_SIGNALING**
