# Supplementary material for: Regulation of microglia related neuroinflammation contributes to the protective effect of Gelsevirine on ischemic stroke
Source: Front Immunol. 2023 Mar 30;14:1164278. doi: 10.3389/fimmu.2023.1164278 (PMC10098192; doi:10.3389/fimmu.2023.1164278)
Supplement: Supplementary file 6 [file DataSheet_6.zip › fig 5 raw/fig 5-G raw/inflammation.Gsea.1649955060129/HAN_JNK_SINGALING_DN.html]

Details for gene set HAN\_JNK\_SINGALING\_DN[GSEA]

|  || Dataset | OGD\_DRUG\_DRUG.OGD\_FRUG.cls#Gs\_versus\_MCAO.OGD\_FRUG.cls#Gs\_versus\_MCAO\_repos |
| Phenotype | OGD\_FRUG.cls#Gs\_versus\_MCAO\_repos |
| Upregulated in class | Gs |
| GeneSet | HAN\_JNK\_SINGALING\_DN |
| Enrichment Score (ES) | 0.35698104 |
| Normalized Enrichment Score (NES) | 0.98625153 |
| Nominal p-value | 0.4587912 |
| FDR q-value | 0.7687713 |
| FWER p-Value | 1.0 |
Table: GSEA Results Summary

  

Fig 1: Enrichment plot: HAN\_JNK\_SINGALING\_DN      
 Profile of the Running ES Score & Positions of GeneSet Members on the Rank Ordered List

  

| SYMBOL | TITLE | RANK IN GENE LIST | RANK METRIC SCORE | RUNNING ES | CORE ENRICHMENT || 1 | IFITM1 | na | 13 | 1.732 | 0.1659 | Yes |
| 2 | PLTP | na | 602 | 0.555 | 0.1923 | Yes |
| 3 | TAF1D | na | 826 | 0.493 | 0.2295 | Yes |
| 4 | RBP1 | na | 855 | 0.486 | 0.2749 | Yes |
| 5 | CD74 | na | 1409 | 0.410 | 0.2890 | Yes |
| 6 | SOD3 | na | 1464 | 0.404 | 0.3254 | Yes |
| 7 | OGN | na | 2073 | 0.331 | 0.3294 | Yes |
| 8 | C3 | na | 3048 | 0.237 | 0.3075 | Yes |
| 9 | CD9 | na | 3224 | 0.220 | 0.3207 | Yes |
| 10 | RGS2 | na | 3705 | 0.186 | 0.3166 | Yes |
| 11 | HSD11B1 | na | 3773 | 0.181 | 0.3309 | Yes |
| 12 | IGFBP4 | na | 3798 | 0.180 | 0.3471 | Yes |
| 13 | ENPP2 | na | 3939 | 0.170 | 0.3570 | Yes |
| 14 | CD14 | na | 4569 | 0.124 | 0.3401 | No |
| 15 | EMB | na | 4715 | 0.114 | 0.3444 | No |
| 16 | SLC10A6 | na | 5361 | 0.076 | 0.3222 | No |
| 17 | ISYNA1 | na | 5532 | 0.066 | 0.3208 | No |
| 18 | SLC29A1 | na | 5903 | 0.047 | 0.3083 | No |
| 19 | FAS | na | 6465 | 0.023 | 0.2848 | No |
| 20 | HAS2 | na | 9500 | 0.000 | 0.1459 | No |
| 21 | WNT6 | na | 9610 | 0.000 | 0.1409 | No |
| 22 | GDF10 | na | 13212 | 0.000 | -0.0240 | No |
| 23 | LIFR | na | 13712 | -0.013 | -0.0456 | No |
| 24 | ABCA1 | na | 15285 | -0.085 | -0.1094 | No |
| 25 | ERO1L | na | 15732 | -0.111 | -0.1192 | No |
| 26 | SOCS3 | na | 16838 | -0.185 | -0.1520 | No |
| 27 | LCN2 | na | 17221 | -0.210 | -0.1493 | No |
| 28 | SEMA3C | na | 17330 | -0.219 | -0.1332 | No |
| 29 | PAWR | na | 17493 | -0.230 | -0.1185 | No |
| 30 | SPIB | na | 17725 | -0.247 | -0.1054 | No |
| 31 | GHR | na | 17916 | -0.263 | -0.0888 | No |
| 32 | CP | na | 19101 | -0.359 | -0.1085 | No |
| 33 | COL6A2 | na | 19189 | -0.369 | -0.0770 | No |
| 34 | MAP4K3 | na | 19853 | -0.431 | -0.0660 | No |
| 35 | COL6A1 | na | 20104 | -0.456 | -0.0336 | No |
| 36 | AHR | na | 20172 | -0.464 | 0.0079 | No |
| 37 | LBP | na | 21555 | -0.728 | 0.0147 | No |
Table: GSEA details [plain text format]

  

Fig 2: HAN\_JNK\_SINGALING\_DN      
 Blue-Pink O' Gram in the Space of the Analyzed GeneSet

  

Fig 3: HAN\_JNK\_SINGALING\_DN: Random ES distribution      
 Gene set null distribution of ES for **HAN\_JNK\_SINGALING\_DN**
