# Supplementary material for: Regulation of microglia related neuroinflammation contributes to the protective effect of Gelsevirine on ischemic stroke
Source: Front Immunol. 2023 Mar 30;14:1164278. doi: 10.3389/fimmu.2023.1164278 (PMC10098192; doi:10.3389/fimmu.2023.1164278)
Supplement: Supplementary file 6 [file DataSheet_6.zip › fig 5 raw/fig 5-G raw/inflammation.Gsea.1649955060129/heat_map_corr_plot.html]

Heat map and correlation plot for OGD\_DRUG\_DRUG.OGD\_FRUG.cls#Gs\_versus\_MCAO  

Fig 1: heat\_map      
 Heat Map of the top 50 features for each phenotype in OGD\_DRUG\_DRUG.OGD\_FRUG.cls#Gs\_versus\_MCAO

  
  

Fig 2: Ranked Gene List Correlation Profile      
 Ranked list correlations for OGD\_DRUG\_DRUG.OGD\_FRUG.cls#Gs\_versus\_MCAO

  
  
    
