# Supplementary material for: Regulation of microglia related neuroinflammation contributes to the protective effect of Gelsevirine on ischemic stroke
Source: Front Immunol. 2023 Mar 30;14:1164278. doi: 10.3389/fimmu.2023.1164278 (PMC10098192; doi:10.3389/fimmu.2023.1164278)
Supplement: Supplementary file 6 [file DataSheet_6.zip › fig 5 raw/fig 5-G raw/inflammation.Gsea.1649955060129/index.html]

Index for xtools.gsea.Gsea inflammation.Gsea.1649955060129

### GSEA Report for Dataset OGD\_DRUG

#### Enrichment in phenotype: **Gs (3 samples)**

- 20 / 134 gene sets are upregulated in phenotype **Gs**- 7 gene sets are significant at FDR < 25%- 4 gene sets are significantly enriched at nominal pvalue < 1%- 6 gene sets are significantly enriched at nominal pvalue < 5%- Snapshot of enrichment results- Detailed enrichment results in html format- Detailed enrichment results in TSV format (tab delimited text)- Guide to interpret results

#### Enrichment in phenotype: **MCAO (3 samples)**

- 114 / 134 gene sets are upregulated in phenotype **MCAO**- 63 gene sets are significantly enriched at FDR < 25%- 20 gene sets are significantly enriched at nominal pvalue < 1%- 40 gene sets are significantly enriched at nominal pvalue < 5%- Snapshot of enrichment results- Detailed enrichment results in html format- Detailed enrichment results in TSV format (tab delimited text)- Guide to interpret results

#### Dataset details

- The dataset has 21876 features (genes)- No probe set => gene symbol collapsing was requested, so all 21876 features were used

#### Gene set details

- Gene set size filters (min=15, max=500) resulted in filtering out 23 / 157 gene sets- The remaining 134 gene sets were used in the analysis- List of gene sets used and their sizes (restricted to features in the specified dataset)

#### Gene markers for the **Gs** *versus* **MCAO** comparison

- The dataset has 21876 features (genes)- # of markers for phenotype **Gs**: 7041 (32.2% ) with correlation area 42.6%- # of markers for phenotype **MCAO**: 14835 (67.8% ) with correlation area 57.4%- Detailed rank ordered gene list for all features in the dataset- Heat map and gene list correlation  profile for all features in the dataset

#### Global statistics and plots

- Plot of p-values *vs.* NES- Global ES histogram

#### Other

- Parameters used for this analysis

#### Comments

- Timestamp used as random seed: 1649955060129

#### Warnings

- There were duplicate row identifiers in the specified dataset. One id was arbitarilly choosen. Details are below
    
  Generally this is OK but if you want to avoid this, edit your dataset so that all row ids are unique
    
    
  # of row ids in original dataset: 21878
    
  # of row UNIQUE ids in original dataset: 21876
    
  The duplicates were
    

  ```
  44256	44257	
  ```

#### Citing GSEA and MSigDB

To cite your use of the GSEA software please reference the following:

- Subramanian, A., Tamayo, P., et al. (2005, PNAS). - Mootha, V. K., Lindgren, C. M., et al. (2003, Nature Genetics).

For use of the Molecular Signatures Database (MSigDB), to cite please reference   
one or more of the following as appropriate, along with the source for the gene set as listed on the gene set page:

- Liberzon A, et al. (Bioinformatics, 2011). - Liberzon A, et al. (Cell Systems 2015).

---

Report: inflammation.Gsea.1649955060129.rpt   by user: Administrator

xtools.gsea.Gsea [Fri, Apr 15, '22 0 AM 51]

Website: www.gsea-msigdb.org/gsea
Questions & Suggestions: Contact page
