# Supplementary material for: Regulation of microglia related neuroinflammation contributes to the protective effect of Gelsevirine on ischemic stroke
Source: Front Immunol. 2023 Mar 30;14:1164278. doi: 10.3389/fimmu.2023.1164278 (PMC10098192; doi:10.3389/fimmu.2023.1164278)
Supplement: Supplementary file 6 [file DataSheet_6.zip › fig 5 raw/fig 5-G raw/inflammation.Gsea.1649955060129/JAZAG_TGFB1_SIGNALING_DN.html]

Details for gene set JAZAG\_TGFB1\_SIGNALING\_DN[GSEA]

|  || Dataset | OGD\_DRUG\_DRUG.OGD\_FRUG.cls#Gs\_versus\_MCAO.OGD\_FRUG.cls#Gs\_versus\_MCAO\_repos |
| Phenotype | OGD\_FRUG.cls#Gs\_versus\_MCAO\_repos |
| Upregulated in class | Gs |
| GeneSet | JAZAG\_TGFB1\_SIGNALING\_DN |
| Enrichment Score (ES) | 0.5921565 |
| Normalized Enrichment Score (NES) | 1.6214858 |
| Nominal p-value | 0.008547009 |
| FDR q-value | 0.048028477 |
| FWER p-Value | 0.277 |
Table: GSEA Results Summary

  

Fig 1: Enrichment plot: JAZAG\_TGFB1\_SIGNALING\_DN      
 Profile of the Running ES Score & Positions of GeneSet Members on the Rank Ordered List

  

| SYMBOL | TITLE | RANK IN GENE LIST | RANK METRIC SCORE | RUNNING ES | CORE ENRICHMENT || 1 | GHRL | na | 42 | 1.263 | 0.1370 | Yes |
| 2 | FLRT1 | na | 79 | 1.061 | 0.2522 | Yes |
| 3 | PLA1A | na | 282 | 0.713 | 0.3213 | Yes |
| 4 | HIST1H2AI | na | 583 | 0.560 | 0.3691 | Yes |
| 5 | SERPINF2 | na | 905 | 0.478 | 0.4070 | Yes |
| 6 | OAZ3 | na | 1152 | 0.441 | 0.4443 | Yes |
| 7 | CWC27 | na | 1566 | 0.392 | 0.4685 | Yes |
| 8 | GLRX2 | na | 1753 | 0.368 | 0.5004 | Yes |
| 9 | RNH1 | na | 1849 | 0.356 | 0.5353 | Yes |
| 10 | ATP2B2 | na | 2425 | 0.293 | 0.5412 | Yes |
| 11 | PSMC3 | na | 2821 | 0.257 | 0.5515 | Yes |
| 12 | SAP18 | na | 2945 | 0.246 | 0.5730 | Yes |
| 13 | MYLPF | na | 3088 | 0.234 | 0.5922 | Yes |
| 14 | EEF1A1 | na | 3958 | 0.168 | 0.5708 | No |
| 15 | STOML2 | na | 4382 | 0.138 | 0.5666 | No |
| 16 | SIGMAR1 | na | 4932 | 0.099 | 0.5524 | No |
| 17 | NAT6 | na | 5087 | 0.091 | 0.5554 | No |
| 18 | P2RX6 | na | 6145 | 0.035 | 0.5109 | No |
| 19 | KCNMB1 | na | 9474 | 0.000 | 0.3585 | No |
| 20 | ARID3B | na | 10804 | 0.000 | 0.2977 | No |
| 21 | CD244 | na | 10863 | 0.000 | 0.2950 | No |
| 22 | ABO | na | 13005 | 0.000 | 0.1970 | No |
| 23 | LSR | na | 13409 | -0.006 | 0.1792 | No |
| 24 | CSDE1 | na | 13691 | -0.012 | 0.1676 | No |
| 25 | FGF6 | na | 15000 | -0.068 | 0.1152 | No |
| 26 | CLCN7 | na | 15547 | -0.101 | 0.1013 | No |
| 27 | PCGF2 | na | 15737 | -0.111 | 0.1049 | No |
| 28 | PCF11 | na | 16159 | -0.138 | 0.1008 | No |
| 29 | TOP2A | na | 17931 | -0.264 | 0.0488 | No |
| 30 | NAB2 | na | 18156 | -0.282 | 0.0696 | No |
| 31 | SUFU | na | 20032 | -0.448 | 0.0330 | No |
| 32 | USP25 | na | 20201 | -0.467 | 0.0766 | No |
Table: GSEA details [plain text format]

  

Fig 2: JAZAG\_TGFB1\_SIGNALING\_DN      
 Blue-Pink O' Gram in the Space of the Analyzed GeneSet

  

Fig 3: JAZAG\_TGFB1\_SIGNALING\_DN: Random ES distribution      
 Gene set null distribution of ES for **JAZAG\_TGFB1\_SIGNALING\_DN**
