# Supplementary material for: Regulation of microglia related neuroinflammation contributes to the protective effect of Gelsevirine on ischemic stroke
Source: Front Immunol. 2023 Mar 30;14:1164278. doi: 10.3389/fimmu.2023.1164278 (PMC10098192; doi:10.3389/fimmu.2023.1164278)
Supplement: Supplementary file 6 [file DataSheet_6.zip › fig 5 raw/fig 5-G raw/inflammation.Gsea.1649955060129/JAZAG_TGFB1_SIGNALING_UP.html]

Details for gene set JAZAG\_TGFB1\_SIGNALING\_UP[GSEA]

|  || Dataset | OGD\_DRUG\_DRUG.OGD\_FRUG.cls#Gs\_versus\_MCAO.OGD\_FRUG.cls#Gs\_versus\_MCAO\_repos |
| Phenotype | OGD\_FRUG.cls#Gs\_versus\_MCAO\_repos |
| Upregulated in class | Gs |
| GeneSet | JAZAG\_TGFB1\_SIGNALING\_UP |
| Enrichment Score (ES) | 0.2833502 |
| Normalized Enrichment Score (NES) | 0.93395233 |
| Nominal p-value | 0.6086956 |
| FDR q-value | 0.89959955 |
| FWER p-Value | 1.0 |
Table: GSEA Results Summary

  

Fig 1: Enrichment plot: JAZAG\_TGFB1\_SIGNALING\_UP      
 Profile of the Running ES Score & Positions of GeneSet Members on the Rank Ordered List

  

| SYMBOL | TITLE | RANK IN GENE LIST | RANK METRIC SCORE | RUNNING ES | CORE ENRICHMENT || 1 | CRABP2 | na | 117 | 0.951 | 0.0321 | Yes |
| 2 | GPR75 | na | 119 | 0.948 | 0.0693 | Yes |
| 3 | RGS17 | na | 352 | 0.656 | 0.0845 | Yes |
| 4 | BUD31 | na | 473 | 0.598 | 0.1026 | Yes |
| 5 | CCL5 | na | 495 | 0.590 | 0.1248 | Yes |
| 6 | TIMM8B | na | 821 | 0.494 | 0.1293 | Yes |
| 7 | LSM5 | na | 924 | 0.474 | 0.1433 | Yes |
| 8 | NUDT21 | na | 1235 | 0.430 | 0.1460 | Yes |
| 9 | EBP | na | 1463 | 0.404 | 0.1515 | Yes |
| 10 | KCNQ4 | na | 1488 | 0.401 | 0.1662 | Yes |
| 11 | PTPN22 | na | 1550 | 0.393 | 0.1789 | Yes |
| 12 | PRDX2 | na | 1699 | 0.374 | 0.1868 | Yes |
| 13 | CCNH | na | 1752 | 0.368 | 0.1989 | Yes |
| 14 | INSL6 | na | 1756 | 0.367 | 0.2132 | Yes |
| 15 | DAP3 | na | 1804 | 0.361 | 0.2253 | Yes |
| 16 | MRPS31 | na | 1899 | 0.349 | 0.2347 | Yes |
| 17 | SPON2 | na | 2364 | 0.297 | 0.2251 | Yes |
| 18 | WBP4 | na | 2408 | 0.295 | 0.2348 | Yes |
| 19 | ITGB7 | na | 2467 | 0.290 | 0.2435 | Yes |
| 20 | ALDOC | na | 2593 | 0.279 | 0.2487 | Yes |
| 21 | CCDC85B | na | 2838 | 0.256 | 0.2476 | Yes |
| 22 | STRAP | na | 2981 | 0.243 | 0.2507 | Yes |
| 23 | SLC35B1 | na | 3119 | 0.231 | 0.2535 | Yes |
| 24 | IL11 | na | 3251 | 0.219 | 0.2561 | Yes |
| 25 | ATF5 | na | 3331 | 0.212 | 0.2608 | Yes |
| 26 | N6AMT1 | na | 3369 | 0.209 | 0.2673 | Yes |
| 27 | FARS2 | na | 3374 | 0.208 | 0.2753 | Yes |
| 28 | PRELID1 | na | 3402 | 0.206 | 0.2822 | Yes |
| 29 | SLC22A7 | na | 3688 | 0.187 | 0.2765 | Yes |
| 30 | CAPN9 | na | 3731 | 0.184 | 0.2818 | Yes |
| 31 | PLP1 | na | 3927 | 0.171 | 0.2796 | Yes |
| 32 | BAG5 | na | 3987 | 0.165 | 0.2834 | Yes |
| 33 | YAF2 | na | 4745 | 0.112 | 0.2530 | No |
| 34 | PKIG | na | 4767 | 0.111 | 0.2564 | No |
| 35 | CPSF4 | na | 4931 | 0.099 | 0.2528 | No |
| 36 | GFER | na | 4943 | 0.099 | 0.2562 | No |
| 37 | CASP3 | na | 5009 | 0.095 | 0.2570 | No |
| 38 | SEC22A | na | 5185 | 0.086 | 0.2523 | No |
| 39 | CNPY3 | na | 5248 | 0.082 | 0.2527 | No |
| 40 | NUP50 | na | 5533 | 0.066 | 0.2423 | No |
| 41 | GTF2H2 | na | 5806 | 0.052 | 0.2318 | No |
| 42 | OSM | na | 5924 | 0.045 | 0.2282 | No |
| 43 | FEZ2 | na | 5952 | 0.044 | 0.2287 | No |
| 44 | VPS4A | na | 6252 | 0.030 | 0.2162 | No |
| 45 | ABCB8 | na | 6262 | 0.030 | 0.2170 | No |
| 46 | TINF2 | na | 6530 | 0.019 | 0.2055 | No |
| 47 | CSF2RB | na | 6547 | 0.019 | 0.2055 | No |
| 48 | CORIN | na | 6591 | 0.017 | 0.2042 | No |
| 49 | PPP1R17 | na | 8380 | 0.000 | 0.1220 | No |
| 50 | IL9 | na | 8414 | 0.000 | 0.1205 | No |
| 51 | SLC6A11 | na | 8519 | 0.000 | 0.1158 | No |
| 52 | WIF1 | na | 8770 | 0.000 | 0.1043 | No |
| 53 | FSCN2 | na | 8889 | 0.000 | 0.0989 | No |
| 54 | TGM5 | na | 10051 | 0.000 | 0.0455 | No |
| 55 | GCM2 | na | 10596 | 0.000 | 0.0206 | No |
| 56 | WISP3 | na | 11990 | 0.000 | -0.0434 | No |
| 57 | LRRC6 | na | 12763 | 0.000 | -0.0789 | No |
| 58 | IL12A | na | 12867 | 0.000 | -0.0836 | No |
| 59 | ST8SIA4 | na | 12870 | 0.000 | -0.0837 | No |
| 60 | PDZK1IP1 | na | 13449 | -0.006 | -0.1100 | No |
| 61 | PTPRH | na | 13988 | -0.020 | -0.1339 | No |
| 62 | PMP22 | na | 14458 | -0.039 | -0.1539 | No |
| 63 | HSF2BP | na | 15053 | -0.071 | -0.1784 | No |
| 64 | SEC23IP | na | 15064 | -0.072 | -0.1760 | No |
| 65 | CHORDC1 | na | 15126 | -0.075 | -0.1759 | No |
| 66 | MAP2K1 | na | 15179 | -0.079 | -0.1751 | No |
| 67 | GSR | na | 15404 | -0.093 | -0.1818 | No |
| 68 | PWP1 | na | 15476 | -0.096 | -0.1812 | No |
| 69 | ABCD1 | na | 15814 | -0.116 | -0.1922 | No |
| 70 | GMEB1 | na | 16193 | -0.140 | -0.2040 | No |
| 71 | RAPGEF3 | na | 16198 | -0.140 | -0.1987 | No |
| 72 | AASS | na | 16251 | -0.144 | -0.1954 | No |
| 73 | TOPORS | na | 16504 | -0.162 | -0.2006 | No |
| 74 | CCNE1 | na | 16729 | -0.178 | -0.2039 | No |
| 75 | CCPG1 | na | 16931 | -0.191 | -0.2055 | No |
| 76 | LPCAT3 | na | 16954 | -0.193 | -0.1990 | No |
| 77 | ATP6V0A2 | na | 17054 | -0.199 | -0.1957 | No |
| 78 | PTK7 | na | 17427 | -0.225 | -0.2039 | No |
| 79 | EHMT2 | na | 17429 | -0.226 | -0.1951 | No |
| 80 | TIMELESS | na | 18140 | -0.280 | -0.2166 | No |
| 81 | NRG1 | na | 18190 | -0.284 | -0.2077 | No |
| 82 | GDNF | na | 18212 | -0.285 | -0.1974 | No |
| 83 | MT3 | na | 18390 | -0.297 | -0.1939 | No |
| 84 | ILF3 | na | 18669 | -0.318 | -0.1942 | No |
| 85 | GTF3C4 | na | 18942 | -0.343 | -0.1931 | No |
| 86 | EMILIN1 | na | 19172 | -0.367 | -0.1892 | No |
| 87 | KPNA6 | na | 19220 | -0.371 | -0.1768 | No |
| 88 | SYNGR4 | na | 19399 | -0.388 | -0.1697 | No |
| 89 | ERLIN2 | na | 19803 | -0.426 | -0.1714 | No |
| 90 | PIGN | na | 20098 | -0.455 | -0.1670 | No |
| 91 | RNF24 | na | 20185 | -0.465 | -0.1526 | No |
| 92 | CELF2 | na | 20281 | -0.475 | -0.1383 | No |
| 93 | SLC12A7 | na | 20373 | -0.485 | -0.1234 | No |
| 94 | DVL3 | na | 20890 | -0.559 | -0.1251 | No |
| 95 | MED14 | na | 21049 | -0.588 | -0.1092 | No |
| 96 | ABHD2 | na | 21202 | -0.620 | -0.0918 | No |
| 97 | SRCAP | na | 21313 | -0.645 | -0.0714 | No |
| 98 | RASAL2 | na | 21549 | -0.725 | -0.0536 | No |
| 99 | L1CAM | na | 21577 | -0.739 | -0.0258 | No |
| 100 | NEU3 | na | 21787 | -1.001 | 0.0040 | No |
Table: GSEA details [plain text format]

  

Fig 2: JAZAG\_TGFB1\_SIGNALING\_UP      
 Blue-Pink O' Gram in the Space of the Analyzed GeneSet

  

Fig 3: JAZAG\_TGFB1\_SIGNALING\_UP: Random ES distribution      
 Gene set null distribution of ES for **JAZAG\_TGFB1\_SIGNALING\_UP**
