# Supplementary material for: Regulation of microglia related neuroinflammation contributes to the protective effect of Gelsevirine on ischemic stroke
Source: Front Immunol. 2023 Mar 30;14:1164278. doi: 10.3389/fimmu.2023.1164278 (PMC10098192; doi:10.3389/fimmu.2023.1164278)
Supplement: Supplementary file 6 [file DataSheet_6.zip › fig 5 raw/fig 5-G raw/inflammation.Gsea.1649955060129/JAZAG_TGFB1_SIGNALING_VIA_SMAD4_UP.html]

Details for gene set JAZAG\_TGFB1\_SIGNALING\_VIA\_SMAD4\_UP[GSEA]

|  || Dataset | OGD\_DRUG\_DRUG.OGD\_FRUG.cls#Gs\_versus\_MCAO.OGD\_FRUG.cls#Gs\_versus\_MCAO\_repos |
| Phenotype | OGD\_FRUG.cls#Gs\_versus\_MCAO\_repos |
| Upregulated in class | Gs |
| GeneSet | JAZAG\_TGFB1\_SIGNALING\_VIA\_SMAD4\_UP |
| Enrichment Score (ES) | 0.27369055 |
| Normalized Enrichment Score (NES) | 0.913022 |
| Nominal p-value | 0.70383275 |
| FDR q-value | 0.907392 |
| FWER p-Value | 1.0 |
Table: GSEA Results Summary

  

Fig 1: Enrichment plot: JAZAG\_TGFB1\_SIGNALING\_VIA\_SMAD4\_UP      
 Profile of the Running ES Score & Positions of GeneSet Members on the Rank Ordered List

  

| SYMBOL | TITLE | RANK IN GENE LIST | RANK METRIC SCORE | RUNNING ES | CORE ENRICHMENT || 1 | SLC23A1 | na | 88 | 1.028 | 0.0442 | Yes |
| 2 | HAS3 | na | 254 | 0.742 | 0.0713 | Yes |
| 3 | BZRAP1 | na | 387 | 0.636 | 0.0951 | Yes |
| 4 | MRPL48 | na | 797 | 0.499 | 0.0997 | Yes |
| 5 | SHFM1 | na | 839 | 0.491 | 0.1208 | Yes |
| 6 | SERTAD1 | na | 922 | 0.474 | 0.1393 | Yes |
| 7 | EIF1 | na | 1009 | 0.463 | 0.1570 | Yes |
| 8 | COPZ1 | na | 1238 | 0.429 | 0.1666 | Yes |
| 9 | TPPP3 | na | 1564 | 0.392 | 0.1701 | Yes |
| 10 | CDK20 | na | 1631 | 0.382 | 0.1850 | Yes |
| 11 | GLRX2 | na | 1753 | 0.368 | 0.1966 | Yes |
| 12 | ARHGEF5 | na | 1760 | 0.367 | 0.2136 | Yes |
| 13 | MPDU1 | na | 1835 | 0.358 | 0.2269 | Yes |
| 14 | HN1 | na | 1871 | 0.352 | 0.2419 | Yes |
| 15 | MRPS31 | na | 1899 | 0.349 | 0.2570 | Yes |
| 16 | MRPL4 | na | 2620 | 0.277 | 0.2369 | Yes |
| 17 | MUC6 | na | 2712 | 0.268 | 0.2453 | Yes |
| 18 | IFI35 | na | 2904 | 0.250 | 0.2482 | Yes |
| 19 | ITIH3 | na | 2921 | 0.248 | 0.2591 | Yes |
| 20 | DAXX | na | 3030 | 0.238 | 0.2654 | Yes |
| 21 | MYLPF | na | 3088 | 0.234 | 0.2737 | Yes |
| 22 | TOM1L1 | na | 3627 | 0.190 | 0.2579 | No |
| 23 | PGRMC2 | na | 3910 | 0.172 | 0.2530 | No |
| 24 | PILRA | na | 4056 | 0.159 | 0.2538 | No |
| 25 | YWHAE | na | 4295 | 0.143 | 0.2496 | No |
| 26 | FKBP8 | na | 4433 | 0.134 | 0.2496 | No |
| 27 | RAB4B | na | 4649 | 0.118 | 0.2452 | No |
| 28 | PKIG | na | 4767 | 0.111 | 0.2451 | No |
| 29 | WDR45 | na | 4887 | 0.102 | 0.2444 | No |
| 30 | WIPI2 | na | 4954 | 0.098 | 0.2459 | No |
| 31 | PTMA | na | 5028 | 0.095 | 0.2470 | No |
| 32 | PTPRC | na | 5113 | 0.090 | 0.2474 | No |
| 33 | CNPY3 | na | 5248 | 0.082 | 0.2451 | No |
| 34 | APH1A | na | 5868 | 0.048 | 0.2189 | No |
| 35 | ENO1 | na | 6025 | 0.039 | 0.2136 | No |
| 36 | NOSIP | na | 6045 | 0.038 | 0.2145 | No |
| 37 | CDIPT | na | 6176 | 0.033 | 0.2101 | No |
| 38 | POP7 | na | 6399 | 0.025 | 0.2011 | No |
| 39 | RAMP1 | na | 7035 | 0.000 | 0.1719 | No |
| 40 | CGA | na | 7346 | 0.000 | 0.1577 | No |
| 41 | LBX1 | na | 7952 | 0.000 | 0.1299 | No |
| 42 | GPR37L1 | na | 8195 | 0.000 | 0.1188 | No |
| 43 | HGF | na | 8228 | 0.000 | 0.1173 | No |
| 44 | PPP1R17 | na | 8380 | 0.000 | 0.1104 | No |
| 45 | LHX2 | na | 8723 | 0.000 | 0.0947 | No |
| 46 | HS3ST3A1 | na | 8824 | 0.000 | 0.0901 | No |
| 47 | GABRB1 | na | 9328 | 0.000 | 0.0670 | No |
| 48 | HCN4 | na | 9831 | 0.000 | 0.0439 | No |
| 49 | RFX4 | na | 11275 | 0.000 | -0.0223 | No |
| 50 | SPINK5 | na | 11732 | 0.000 | -0.0433 | No |
| 51 | CDH6 | na | 13102 | 0.000 | -0.1061 | No |
| 52 | PDZK1IP1 | na | 13449 | -0.006 | -0.1217 | No |
| 53 | KLK11 | na | 13497 | -0.007 | -0.1236 | No |
| 54 | SATB1 | na | 13514 | -0.007 | -0.1240 | No |
| 55 | GPR45 | na | 13790 | -0.014 | -0.1359 | No |
| 56 | ACTB | na | 13965 | -0.019 | -0.1430 | No |
| 57 | CAB39 | na | 14161 | -0.026 | -0.1508 | No |
| 58 | C5AR1 | na | 14341 | -0.033 | -0.1574 | No |
| 59 | PAX9 | na | 14373 | -0.035 | -0.1572 | No |
| 60 | NAMPT | na | 14416 | -0.036 | -0.1574 | No |
| 61 | FXR2 | na | 14427 | -0.037 | -0.1562 | No |
| 62 | UBA2 | na | 14649 | -0.048 | -0.1641 | No |
| 63 | NPRL2 | na | 14990 | -0.067 | -0.1765 | No |
| 64 | STK25 | na | 15043 | -0.071 | -0.1756 | No |
| 65 | PADI3 | na | 15044 | -0.071 | -0.1723 | No |
| 66 | CELSR3 | na | 15119 | -0.075 | -0.1722 | No |
| 67 | HMGA1 | na | 15308 | -0.086 | -0.1767 | No |
| 68 | CD27 | na | 15362 | -0.090 | -0.1750 | No |
| 69 | COG2 | na | 15567 | -0.102 | -0.1796 | No |
| 70 | TOM1 | na | 15597 | -0.104 | -0.1761 | No |
| 71 | MBD1 | na | 15660 | -0.106 | -0.1739 | No |
| 72 | PCGF2 | na | 15737 | -0.111 | -0.1722 | No |
| 73 | CRADD | na | 15754 | -0.112 | -0.1677 | No |
| 74 | GRHPR | na | 16110 | -0.135 | -0.1777 | No |
| 75 | ATP2A1 | na | 16311 | -0.148 | -0.1799 | No |
| 76 | GATA4 | na | 16474 | -0.160 | -0.1799 | No |
| 77 | DNAH2 | na | 16646 | -0.173 | -0.1796 | No |
| 78 | C1QB | na | 17277 | -0.215 | -0.1985 | No |
| 79 | IGF2BP1 | na | 17355 | -0.220 | -0.1917 | No |
| 80 | UBIAD1 | na | 17540 | -0.233 | -0.1892 | No |
| 81 | OSGIN1 | na | 17566 | -0.235 | -0.1794 | No |
| 82 | SRRM1 | na | 17679 | -0.243 | -0.1732 | No |
| 83 | LPAR2 | na | 17827 | -0.255 | -0.1679 | No |
| 84 | MYO1F | na | 18143 | -0.281 | -0.1693 | No |
| 85 | CAMK2B | na | 18339 | -0.294 | -0.1645 | No |
| 86 | ERCC5 | na | 18886 | -0.339 | -0.1737 | No |
| 87 | MYBL2 | na | 19166 | -0.367 | -0.1693 | No |
| 88 | CBX2 | na | 19319 | -0.379 | -0.1585 | No |
| 89 | CBX5 | na | 19581 | -0.405 | -0.1515 | No |
| 90 | HEBP1 | na | 19600 | -0.406 | -0.1333 | No |
| 91 | CYP1B1 | na | 19841 | -0.429 | -0.1242 | No |
| 92 | NUP153 | na | 20011 | -0.446 | -0.1111 | No |
| 93 | CELF2 | na | 20281 | -0.475 | -0.1012 | No |
| 94 | FGF12 | na | 20471 | -0.499 | -0.0865 | No |
| 95 | HDAC4 | na | 20556 | -0.510 | -0.0664 | No |
| 96 | LSP1 | na | 21152 | -0.608 | -0.0653 | No |
| 97 | SRCAP | na | 21313 | -0.645 | -0.0424 | No |
| 98 | EP300 | na | 21416 | -0.674 | -0.0155 | No |
| 99 | PEG3 | na | 21637 | -0.779 | 0.0109 | No |
Table: GSEA details [plain text format]

  

Fig 2: JAZAG\_TGFB1\_SIGNALING\_VIA\_SMAD4\_UP      
 Blue-Pink O' Gram in the Space of the Analyzed GeneSet

  

Fig 3: JAZAG\_TGFB1\_SIGNALING\_VIA\_SMAD4\_UP: Random ES distribution      
 Gene set null distribution of ES for **JAZAG\_TGFB1\_SIGNALING\_VIA\_SMAD4\_UP**
