# Supplementary material for: Regulation of microglia related neuroinflammation contributes to the protective effect of Gelsevirine on ischemic stroke
Source: Front Immunol. 2023 Mar 30;14:1164278. doi: 10.3389/fimmu.2023.1164278 (PMC10098192; doi:10.3389/fimmu.2023.1164278)
Supplement: Supplementary file 6 [file DataSheet_6.zip › fig 5 raw/fig 5-G raw/inflammation.Gsea.1649955060129/KEGG_PPAR_SIGNALING_PATHWAY.html]

Details for gene set KEGG\_PPAR\_SIGNALING\_PATHWAY[GSEA]

|  || Dataset | OGD\_DRUG\_DRUG.OGD\_FRUG.cls#Gs\_versus\_MCAO.OGD\_FRUG.cls#Gs\_versus\_MCAO\_repos |
| Phenotype | OGD\_FRUG.cls#Gs\_versus\_MCAO\_repos |
| Upregulated in class | Gs |
| GeneSet | KEGG\_PPAR\_SIGNALING\_PATHWAY |
| Enrichment Score (ES) | 0.335089 |
| Normalized Enrichment Score (NES) | 1.0275677 |
| Nominal p-value | 0.38304093 |
| FDR q-value | 0.75617135 |
| FWER p-Value | 1.0 |
Table: GSEA Results Summary

  

Fig 1: Enrichment plot: KEGG\_PPAR\_SIGNALING\_PATHWAY      
 Profile of the Running ES Score & Positions of GeneSet Members on the Rank Ordered List

  

| SYMBOL | TITLE | RANK IN GENE LIST | RANK METRIC SCORE | RUNNING ES | CORE ENRICHMENT || 1 | SLC27A5 | na | 241 | 0.750 | 0.0395 | Yes |
| 2 | NR1H3 | na | 271 | 0.721 | 0.0867 | Yes |
| 3 | ACSL6 | na | 312 | 0.686 | 0.1310 | Yes |
| 4 | PLTP | na | 602 | 0.555 | 0.1551 | Yes |
| 5 | DBI | na | 915 | 0.476 | 0.1728 | Yes |
| 6 | FABP5 | na | 1018 | 0.462 | 0.1992 | Yes |
| 7 | CYP27A1 | na | 1385 | 0.412 | 0.2102 | Yes |
| 8 | APOA2 | na | 1576 | 0.391 | 0.2278 | Yes |
| 9 | PCK1 | na | 1622 | 0.384 | 0.2516 | Yes |
| 10 | SCP2 | na | 2044 | 0.333 | 0.2547 | Yes |
| 11 | FABP4 | na | 2117 | 0.325 | 0.2732 | Yes |
| 12 | PCK2 | na | 2203 | 0.315 | 0.2905 | Yes |
| 13 | ACOX2 | na | 2472 | 0.289 | 0.2977 | Yes |
| 14 | CD36 | na | 2486 | 0.287 | 0.3165 | Yes |
| 15 | CPT1C | na | 2501 | 0.286 | 0.3351 | Yes |
| 16 | ACADL | na | 3084 | 0.234 | 0.3242 | No |
| 17 | APOA5 | na | 3435 | 0.204 | 0.3219 | No |
| 18 | ACADM | na | 3960 | 0.168 | 0.3091 | No |
| 19 | LPL | na | 4214 | 0.148 | 0.3075 | No |
| 20 | ACSL5 | na | 4352 | 0.139 | 0.3106 | No |
| 21 | FABP3 | na | 4659 | 0.117 | 0.3045 | No |
| 22 | APOC3 | na | 4870 | 0.104 | 0.3018 | No |
| 23 | ACSL3 | na | 5200 | 0.085 | 0.2924 | No |
| 24 | AQP7 | na | 5287 | 0.080 | 0.2939 | No |
| 25 | ADIPOQ | na | 5554 | 0.065 | 0.2861 | No |
| 26 | EHHADH | na | 6953 | 0.003 | 0.2222 | No |
| 27 | OLR1 | na | 8004 | 0.000 | 0.1741 | No |
| 28 | SLC27A6 | na | 8026 | 0.000 | 0.1731 | No |
| 29 | GK2 | na | 8124 | 0.000 | 0.1687 | No |
| 30 | RXRG | na | 8397 | 0.000 | 0.1562 | No |
| 31 | UCP1 | na | 8852 | 0.000 | 0.1354 | No |
| 32 | FABP1 | na | 12795 | 0.000 | -0.0453 | No |
| 33 | FABP6 | na | 12799 | 0.000 | -0.0455 | No |
| 34 | FABP7 | na | 12801 | 0.000 | -0.0455 | No |
| 35 | ANGPTL4 | na | 13686 | -0.012 | -0.0852 | No |
| 36 | SLC27A2 | na | 13974 | -0.019 | -0.0971 | No |
| 37 | ME1 | na | 14096 | -0.023 | -0.1011 | No |
| 38 | ILK | na | 14151 | -0.026 | -0.1018 | No |
| 39 | SLC27A1 | na | 14324 | -0.033 | -0.1075 | No |
| 40 | PLIN1 | na | 14561 | -0.043 | -0.1154 | No |
| 41 | CYP8B1 | na | 14590 | -0.045 | -0.1137 | No |
| 42 | RXRB | na | 14636 | -0.047 | -0.1125 | No |
| 43 | CPT1B | na | 14833 | -0.058 | -0.1176 | No |
| 44 | UBC | na | 15165 | -0.078 | -0.1276 | No |
| 45 | PPARA | na | 15166 | -0.078 | -0.1223 | No |
| 46 | ACOX1 | na | 16007 | -0.129 | -0.1521 | No |
| 47 | SLC27A4 | na | 16302 | -0.147 | -0.1557 | No |
| 48 | CPT2 | na | 16367 | -0.152 | -0.1484 | No |
| 49 | FABP2 | na | 16974 | -0.194 | -0.1631 | No |
| 50 | SORBS1 | na | 17043 | -0.198 | -0.1528 | No |
| 51 | PPARD | na | 17742 | -0.248 | -0.1682 | No |
| 52 | GK | na | 17973 | -0.267 | -0.1607 | No |
| 53 | ACSL1 | na | 18400 | -0.297 | -0.1603 | No |
| 54 | ACSL4 | na | 18433 | -0.299 | -0.1416 | No |
| 55 | HMGCS2 | na | 19058 | -0.355 | -0.1463 | No |
| 56 | APOA1 | na | 19083 | -0.358 | -0.1233 | No |
| 57 | RXRA | na | 19209 | -0.370 | -0.1041 | No |
| 58 | PDPK1 | na | 20100 | -0.455 | -0.1143 | No |
| 59 | ACOX3 | na | 20150 | -0.462 | -0.0855 | No |
| 60 | FADS2 | na | 20280 | -0.475 | -0.0594 | No |
| 61 | CPT1A | na | 20765 | -0.541 | -0.0452 | No |
| 62 | CYP7A1 | na | 21190 | -0.618 | -0.0230 | No |
| 63 | PPARG | na | 21669 | -0.808 | 0.0094 | No |
Table: GSEA details [plain text format]

  

Fig 2: KEGG\_PPAR\_SIGNALING\_PATHWAY      
 Blue-Pink O' Gram in the Space of the Analyzed GeneSet

  

Fig 3: KEGG\_PPAR\_SIGNALING\_PATHWAY: Random ES distribution      
 Gene set null distribution of ES for **KEGG\_PPAR\_SIGNALING\_PATHWAY**
