# Supplementary material for: Regulation of microglia related neuroinflammation contributes to the protective effect of Gelsevirine on ischemic stroke
Source: Front Immunol. 2023 Mar 30;14:1164278. doi: 10.3389/fimmu.2023.1164278 (PMC10098192; doi:10.3389/fimmu.2023.1164278)
Supplement: Supplementary file 6 [file DataSheet_6.zip › fig 5 raw/fig 5-G raw/inflammation.Gsea.1649955060129/KEGG_TGF_BETA_SIGNALING_PATHWAY.html]

Details for gene set KEGG\_TGF\_BETA\_SIGNALING\_PATHWAY[GSEA]

|  || Dataset | OGD\_DRUG\_DRUG.OGD\_FRUG.cls#Gs\_versus\_MCAO.OGD\_FRUG.cls#Gs\_versus\_MCAO\_repos |
| Phenotype | OGD\_FRUG.cls#Gs\_versus\_MCAO\_repos |
| Upregulated in class | MCAO |
| GeneSet | KEGG\_TGF\_BETA\_SIGNALING\_PATHWAY |
| Enrichment Score (ES) | -0.5257928 |
| Normalized Enrichment Score (NES) | -1.5341494 |
| Nominal p-value | 0.0 |
| FDR q-value | 0.08929256 |
| FWER p-Value | 0.581 |
Table: GSEA Results Summary

  

Fig 1: Enrichment plot: KEGG\_TGF\_BETA\_SIGNALING\_PATHWAY      
 Profile of the Running ES Score & Positions of GeneSet Members on the Rank Ordered List

  

| SYMBOL | TITLE | RANK IN GENE LIST | RANK METRIC SCORE | RUNNING ES | CORE ENRICHMENT || 1 | DCN | na | 328 | 0.673 | 0.0184 | No |
| 2 | TGFB2 | na | 485 | 0.594 | 0.0407 | No |
| 3 | RBX1 | na | 1016 | 0.462 | 0.0393 | No |
| 4 | ACVRL1 | na | 1829 | 0.359 | 0.0198 | No |
| 5 | RHOA | na | 1999 | 0.338 | 0.0288 | No |
| 6 | BMP8B | na | 2328 | 0.301 | 0.0287 | No |
| 7 | E2F5 | na | 2910 | 0.250 | 0.0144 | No |
| 8 | ID1 | na | 2925 | 0.248 | 0.0261 | No |
| 9 | ID3 | na | 3103 | 0.233 | 0.0295 | No |
| 10 | PPP2CB | na | 4186 | 0.150 | -0.0127 | No |
| 11 | MAPK3 | na | 4918 | 0.100 | -0.0413 | No |
| 12 | COMP | na | 5235 | 0.083 | -0.0517 | No |
| 13 | MYC | na | 5587 | 0.064 | -0.0646 | No |
| 14 | TNF | na | 5838 | 0.050 | -0.0736 | No |
| 15 | INHBC | na | 5973 | 0.043 | -0.0776 | No |
| 16 | THBS3 | na | 6218 | 0.032 | -0.0873 | No |
| 17 | PPP2CA | na | 6335 | 0.027 | -0.0912 | No |
| 18 | ID4 | na | 6472 | 0.022 | -0.0964 | No |
| 19 | E2F4 | na | 6746 | 0.011 | -0.1083 | No |
| 20 | INHBB | na | 6758 | 0.011 | -0.1083 | No |
| 21 | ACVR1C | na | 6811 | 0.009 | -0.1103 | No |
| 22 | LEFTY1 | na | 7040 | 0.000 | -0.1207 | No |
| 23 | NODAL | na | 8533 | 0.000 | -0.1892 | No |
| 24 | BMP5 | na | 9000 | 0.000 | -0.2106 | No |
| 25 | BMP2 | na | 9002 | 0.000 | -0.2106 | No |
| 26 | NOG | na | 9106 | 0.000 | -0.2154 | No |
| 27 | LEFTY2 | na | 9503 | 0.000 | -0.2335 | No |
| 28 | IFNG | na | 9828 | 0.000 | -0.2484 | No |
| 29 | BMP8A | na | 9894 | 0.000 | -0.2514 | No |
| 30 | CDKN2B | na | 10239 | 0.000 | -0.2672 | No |
| 31 | PITX2 | na | 10464 | 0.000 | -0.2774 | No |
| 32 | GDF6 | na | 10743 | 0.000 | -0.2902 | No |
| 33 | GDF7 | na | 10744 | 0.000 | -0.2902 | No |
| 34 | BMPR1B | na | 12680 | 0.000 | -0.3790 | No |
| 35 | THBS4 | na | 12698 | 0.000 | -0.3798 | No |
| 36 | AMHR2 | na | 13529 | -0.008 | -0.4175 | No |
| 37 | ID2 | na | 14104 | -0.024 | -0.4427 | No |
| 38 | ACVR2A | na | 14123 | -0.024 | -0.4423 | No |
| 39 | BMP7 | na | 14140 | -0.025 | -0.4418 | No |
| 40 | PPP2R1A | na | 14223 | -0.029 | -0.4441 | No |
| 41 | INHBE | na | 14483 | -0.040 | -0.4540 | No |
| 42 | SMAD2 | na | 14505 | -0.041 | -0.4530 | No |
| 43 | CUL1 | na | 14514 | -0.041 | -0.4513 | No |
| 44 | SMAD4 | na | 14652 | -0.048 | -0.4552 | No |
| 45 | SMAD1 | na | 15765 | -0.113 | -0.5006 | No |
| 46 | RPS6KB2 | na | 15831 | -0.117 | -0.4978 | No |
| 47 | TGFB1 | na | 16186 | -0.140 | -0.5071 | No |
| 48 | MAPK1 | na | 16234 | -0.143 | -0.5022 | No |
| 49 | SMAD6 | na | 16287 | -0.146 | -0.4973 | No |
| 50 | SMAD7 | na | 16595 | -0.169 | -0.5030 | No |
| 51 | BMP4 | na | 17035 | -0.198 | -0.5133 | Yes |
| 52 | SMAD9 | na | 17080 | -0.201 | -0.5054 | Yes |
| 53 | RPS6KB1 | na | 17275 | -0.214 | -0.5036 | Yes |
| 54 | BMP6 | na | 17615 | -0.238 | -0.5074 | Yes |
| 55 | PPP2R1B | na | 18017 | -0.271 | -0.5124 | Yes |
| 56 | TFDP1 | na | 18032 | -0.271 | -0.4996 | Yes |
| 57 | ACVR1 | na | 18035 | -0.272 | -0.4862 | Yes |
| 58 | CHRD | na | 18185 | -0.283 | -0.4789 | Yes |
| 59 | FST | na | 18517 | -0.305 | -0.4790 | Yes |
| 60 | SMAD5 | na | 18677 | -0.319 | -0.4705 | Yes |
| 61 | THBS2 | na | 19115 | -0.362 | -0.4726 | Yes |
| 62 | BMPR1A | na | 19148 | -0.365 | -0.4559 | Yes |
| 63 | SMURF1 | na | 19205 | -0.370 | -0.4402 | Yes |
| 64 | LTBP1 | na | 19249 | -0.373 | -0.4236 | Yes |
| 65 | ZFYVE9 | na | 19383 | -0.385 | -0.4106 | Yes |
| 66 | SMAD3 | na | 19611 | -0.408 | -0.4008 | Yes |
| 67 | SP1 | na | 19717 | -0.418 | -0.3849 | Yes |
| 68 | ROCK1 | na | 19820 | -0.427 | -0.3683 | Yes |
| 69 | TGFB3 | na | 19860 | -0.432 | -0.3487 | Yes |
| 70 | TGFBR2 | na | 20121 | -0.457 | -0.3380 | Yes |
| 71 | SMURF2 | na | 20323 | -0.480 | -0.3234 | Yes |
| 72 | RBL2 | na | 20390 | -0.486 | -0.3023 | Yes |
| 73 | INHBA | na | 20552 | -0.509 | -0.2844 | Yes |
| 74 | GDF5 | na | 20584 | -0.514 | -0.2604 | Yes |
| 75 | ROCK2 | na | 20637 | -0.521 | -0.2369 | Yes |
| 76 | ZFYVE16 | na | 20726 | -0.533 | -0.2145 | Yes |
| 77 | TGFBR1 | na | 20821 | -0.549 | -0.1916 | Yes |
| 78 | RBL1 | na | 20834 | -0.551 | -0.1648 | Yes |
| 79 | ACVR2B | na | 21241 | -0.629 | -0.1522 | Yes |
| 80 | BMPR2 | na | 21245 | -0.629 | -0.1211 | Yes |
| 81 | CREBBP | na | 21304 | -0.643 | -0.0919 | Yes |
| 82 | EP300 | na | 21416 | -0.674 | -0.0636 | Yes |
| 83 | THBS1 | na | 21664 | -0.806 | -0.0349 | Yes |
| 84 | AMH | na | 21748 | -0.898 | 0.0058 | Yes |
Table: GSEA details [plain text format]

  

Fig 2: KEGG\_TGF\_BETA\_SIGNALING\_PATHWAY      
 Blue-Pink O' Gram in the Space of the Analyzed GeneSet

  

Fig 3: KEGG\_TGF\_BETA\_SIGNALING\_PATHWAY: Random ES distribution      
 Gene set null distribution of ES for **KEGG\_TGF\_BETA\_SIGNALING\_PATHWAY**
