# Supplementary material for: Regulation of microglia related neuroinflammation contributes to the protective effect of Gelsevirine on ischemic stroke
Source: Front Immunol. 2023 Mar 30;14:1164278. doi: 10.3389/fimmu.2023.1164278 (PMC10098192; doi:10.3389/fimmu.2023.1164278)
Supplement: Supplementary file 6 [file DataSheet_6.zip › fig 5 raw/fig 5-G raw/inflammation.Gsea.1649955060129/KEGG_TIGHT_JUNCTION.html]

Details for gene set KEGG\_TIGHT\_JUNCTION[GSEA]

|  || Dataset | OGD\_DRUG\_DRUG.OGD\_FRUG.cls#Gs\_versus\_MCAO.OGD\_FRUG.cls#Gs\_versus\_MCAO\_repos |
| Phenotype | OGD\_FRUG.cls#Gs\_versus\_MCAO\_repos |
| Upregulated in class | MCAO |
| GeneSet | KEGG\_TIGHT\_JUNCTION |
| Enrichment Score (ES) | -0.4880349 |
| Normalized Enrichment Score (NES) | -1.4898144 |
| Nominal p-value | 0.0039421814 |
| FDR q-value | 0.09672354 |
| FWER p-Value | 0.756 |
Table: GSEA Results Summary

  

Fig 1: Enrichment plot: KEGG\_TIGHT\_JUNCTION      
 Profile of the Running ES Score & Positions of GeneSet Members on the Rank Ordered List

  

| SYMBOL | TITLE | RANK IN GENE LIST | RANK METRIC SCORE | RUNNING ES | CORE ENRICHMENT || 1 | MYH3 | na | 192 | 0.803 | 0.0165 | No |
| 2 | RAB3B | na | 246 | 0.746 | 0.0375 | No |
| 3 | PARD6A | na | 906 | 0.478 | 0.0223 | No |
| 4 | CLDN15 | na | 1026 | 0.461 | 0.0313 | No |
| 5 | RHOA | na | 1999 | 0.338 | -0.0027 | No |
| 6 | VAPA | na | 2072 | 0.331 | 0.0044 | No |
| 7 | RAB13 | na | 2124 | 0.324 | 0.0123 | No |
| 8 | CSNK2B | na | 2192 | 0.316 | 0.0191 | No |
| 9 | CLDN10 | na | 2389 | 0.297 | 0.0195 | No |
| 10 | MYL12A | na | 2589 | 0.279 | 0.0191 | No |
| 11 | HRAS | na | 2716 | 0.268 | 0.0218 | No |
| 12 | CSNK2A2 | na | 2818 | 0.258 | 0.0252 | No |
| 13 | MYLPF | na | 3088 | 0.234 | 0.0202 | No |
| 14 | MYL12B | na | 3105 | 0.233 | 0.0268 | No |
| 15 | RRAS2 | na | 3196 | 0.224 | 0.0297 | No |
| 16 | CDK4 | na | 3240 | 0.219 | 0.0347 | No |
| 17 | HCLS1 | na | 3920 | 0.171 | 0.0089 | No |
| 18 | PPP2CB | na | 4186 | 0.150 | 0.0014 | No |
| 19 | PRKCG | na | 4221 | 0.148 | 0.0045 | No |
| 20 | GNAI1 | na | 4304 | 0.143 | 0.0052 | No |
| 21 | NRAS | na | 4669 | 0.117 | -0.0079 | No |
| 22 | CDC42 | na | 4900 | 0.101 | -0.0152 | No |
| 23 | PPP2R2D | na | 5218 | 0.084 | -0.0272 | No |
| 24 | MYL9 | na | 5490 | 0.069 | -0.0375 | No |
| 25 | CLDN16 | na | 5495 | 0.068 | -0.0355 | No |
| 26 | CLDN3 | na | 5559 | 0.065 | -0.0364 | No |
| 27 | PPP2R2A | na | 5644 | 0.060 | -0.0383 | No |
| 28 | ACTN2 | na | 5721 | 0.057 | -0.0400 | No |
| 29 | TJP3 | na | 5786 | 0.053 | -0.0413 | No |
| 30 | PRKCZ | na | 6115 | 0.036 | -0.0553 | No |
| 31 | PPP2CA | na | 6335 | 0.027 | -0.0645 | No |
| 32 | CTNNA2 | na | 6496 | 0.021 | -0.0712 | No |
| 33 | KRAS | na | 6543 | 0.019 | -0.0727 | No |
| 34 | IGSF5 | na | 6862 | 0.006 | -0.0871 | No |
| 35 | CRB3 | na | 6874 | 0.006 | -0.0874 | No |
| 36 | RRAS | na | 6958 | 0.003 | -0.0911 | No |
| 37 | PPP2R2B | na | 8248 | 0.000 | -0.1504 | No |
| 38 | MYH13 | na | 8554 | 0.000 | -0.1645 | No |
| 39 | JAM2 | na | 8604 | 0.000 | -0.1667 | No |
| 40 | MYL10 | na | 9092 | 0.000 | -0.1891 | No |
| 41 | CLDN22 | na | 9411 | 0.000 | -0.2037 | No |
| 42 | CLDN23 | na | 9414 | 0.000 | -0.2038 | No |
| 43 | CLDN11 | na | 9415 | 0.000 | -0.2038 | No |
| 44 | CLDN14 | na | 9417 | 0.000 | -0.2039 | No |
| 45 | CLDN17 | na | 9421 | 0.000 | -0.2040 | No |
| 46 | CLDN5 | na | 9467 | 0.000 | -0.2061 | No |
| 47 | CLDN8 | na | 9468 | 0.000 | -0.2061 | No |
| 48 | CLDN7 | na | 9469 | 0.000 | -0.2061 | No |
| 49 | PRKCQ | na | 10157 | 0.000 | -0.2377 | No |
| 50 | MYH8 | na | 11247 | 0.000 | -0.2877 | No |
| 51 | MYH4 | na | 11248 | 0.000 | -0.2877 | No |
| 52 | MYH7 | na | 11249 | 0.000 | -0.2877 | No |
| 53 | MYL2 | na | 11274 | 0.000 | -0.2888 | No |
| 54 | CLDN20 | na | 13522 | -0.007 | -0.3919 | No |
| 55 | PRKCB | na | 13570 | -0.009 | -0.3938 | No |
| 56 | CLDN19 | na | 13731 | -0.013 | -0.4008 | No |
| 57 | MYH15 | na | 13766 | -0.013 | -0.4019 | No |
| 58 | MYH6 | na | 13792 | -0.014 | -0.4026 | No |
| 59 | MYH2 | na | 13804 | -0.015 | -0.4027 | No |
| 60 | MYH7B | na | 13935 | -0.018 | -0.4081 | No |
| 61 | ACTB | na | 13965 | -0.019 | -0.4088 | No |
| 62 | PPP2R1A | na | 14223 | -0.029 | -0.4197 | No |
| 63 | F11R | na | 14292 | -0.031 | -0.4219 | No |
| 64 | CLDN1 | na | 14801 | -0.056 | -0.4434 | No |
| 65 | MRAS | na | 14846 | -0.059 | -0.4436 | No |
| 66 | EXOC3 | na | 14921 | -0.064 | -0.4450 | No |
| 67 | JAM3 | na | 15060 | -0.071 | -0.4491 | No |
| 68 | CSNK2A1 | na | 15329 | -0.088 | -0.4587 | No |
| 69 | CLDN9 | na | 15957 | -0.126 | -0.4836 | Yes |
| 70 | MYL7 | na | 15980 | -0.127 | -0.4806 | Yes |
| 71 | PRKCD | na | 16045 | -0.131 | -0.4794 | Yes |
| 72 | PARD6G | na | 16169 | -0.139 | -0.4806 | Yes |
| 73 | ACTG1 | na | 16172 | -0.139 | -0.4764 | Yes |
| 74 | GNAI2 | na | 16257 | -0.144 | -0.4757 | Yes |
| 75 | MPP5 | na | 16335 | -0.150 | -0.4745 | Yes |
| 76 | YES1 | na | 16373 | -0.153 | -0.4714 | Yes |
| 77 | CTTN | na | 16425 | -0.156 | -0.4688 | Yes |
| 78 | CLDN6 | na | 16676 | -0.175 | -0.4748 | Yes |
| 79 | AKT2 | na | 16784 | -0.181 | -0.4740 | Yes |
| 80 | AKT1 | na | 16827 | -0.184 | -0.4701 | Yes |
| 81 | ACTN3 | na | 17217 | -0.210 | -0.4814 | Yes |
| 82 | SYMPK | na | 17262 | -0.213 | -0.4767 | Yes |
| 83 | PTEN | na | 17342 | -0.220 | -0.4734 | Yes |
| 84 | CTNNA1 | na | 17378 | -0.222 | -0.4680 | Yes |
| 85 | GNAI3 | na | 17440 | -0.226 | -0.4637 | Yes |
| 86 | MYH11 | na | 17806 | -0.253 | -0.4725 | Yes |
| 87 | AKT3 | na | 17899 | -0.261 | -0.4685 | Yes |
| 88 | PPP2R1B | na | 18017 | -0.271 | -0.4654 | Yes |
| 89 | CLDN4 | na | 18144 | -0.281 | -0.4623 | Yes |
| 90 | PRKCA | na | 18221 | -0.286 | -0.4568 | Yes |
| 91 | CTNNB1 | na | 18285 | -0.290 | -0.4506 | Yes |
| 92 | PRKCH | na | 18320 | -0.292 | -0.4430 | Yes |
| 93 | OCLN | na | 18369 | -0.295 | -0.4359 | Yes |
| 94 | CLDN2 | na | 18381 | -0.296 | -0.4271 | Yes |
| 95 | EPB41L1 | na | 18438 | -0.299 | -0.4202 | Yes |
| 96 | CGN | na | 18474 | -0.301 | -0.4123 | Yes |
| 97 | MYH1 | na | 18900 | -0.340 | -0.4212 | Yes |
| 98 | TJAP1 | na | 18907 | -0.340 | -0.4107 | Yes |
| 99 | ACTN4 | na | 19490 | -0.396 | -0.4250 | Yes |
| 100 | PARD3 | na | 19505 | -0.397 | -0.4131 | Yes |
| 101 | MAGI1 | na | 19515 | -0.398 | -0.4010 | Yes |
| 102 | PRKCI | na | 19552 | -0.402 | -0.3900 | Yes |
| 103 | ZAK | na | 19557 | -0.403 | -0.3775 | Yes |
| 104 | EPB41L3 | na | 19560 | -0.403 | -0.3649 | Yes |
| 105 | EPB41L2 | na | 19621 | -0.409 | -0.3548 | Yes |
| 106 | LLGL2 | na | 19927 | -0.437 | -0.3550 | Yes |
| 107 | CTNNA3 | na | 19975 | -0.442 | -0.3433 | Yes |
| 108 | LLGL1 | na | 20012 | -0.446 | -0.3309 | Yes |
| 109 | EXOC4 | na | 20177 | -0.465 | -0.3238 | Yes |
| 110 | MAGI2 | na | 20207 | -0.467 | -0.3104 | Yes |
| 111 | SRC | na | 20255 | -0.472 | -0.2977 | Yes |
| 112 | CASK | na | 20502 | -0.503 | -0.2932 | Yes |
| 113 | TJP1 | na | 20615 | -0.518 | -0.2820 | Yes |
| 114 | ACTN1 | na | 20635 | -0.521 | -0.2664 | Yes |
| 115 | PARD6B | na | 20662 | -0.524 | -0.2511 | Yes |
| 116 | PPP2R2C | na | 20711 | -0.530 | -0.2366 | Yes |
| 117 | SPTAN1 | na | 20717 | -0.531 | -0.2201 | Yes |
| 118 | EPB41 | na | 20720 | -0.531 | -0.2035 | Yes |
| 119 | AMOTL1 | na | 20860 | -0.555 | -0.1924 | Yes |
| 120 | TJP2 | na | 20920 | -0.564 | -0.1773 | Yes |
| 121 | MYH10 | na | 21043 | -0.587 | -0.1645 | Yes |
| 122 | MPDZ | na | 21064 | -0.591 | -0.1468 | Yes |
| 123 | MLLT4 | na | 21105 | -0.599 | -0.1297 | Yes |
| 124 | MAGI3 | na | 21166 | -0.611 | -0.1132 | Yes |
| 125 | PRKCE | na | 21181 | -0.615 | -0.0945 | Yes |
| 126 | MYH9 | na | 21236 | -0.628 | -0.0772 | Yes |
| 127 | ASH1L | na | 21240 | -0.629 | -0.0575 | Yes |
| 128 | INADL | na | 21462 | -0.691 | -0.0459 | Yes |
| 129 | MYH14 | na | 21557 | -0.729 | -0.0273 | Yes |
| 130 | CLDN18 | na | 21854 | -1.329 | 0.0010 | Yes |
Table: GSEA details [plain text format]

  

Fig 2: KEGG\_TIGHT\_JUNCTION      
 Blue-Pink O' Gram in the Space of the Analyzed GeneSet

  

Fig 3: KEGG\_TIGHT\_JUNCTION: Random ES distribution      
 Gene set null distribution of ES for **KEGG\_TIGHT\_JUNCTION**
