# Supplementary material for: Regulation of microglia related neuroinflammation contributes to the protective effect of Gelsevirine on ischemic stroke
Source: Front Immunol. 2023 Mar 30;14:1164278. doi: 10.3389/fimmu.2023.1164278 (PMC10098192; doi:10.3389/fimmu.2023.1164278)
Supplement: Supplementary file 6 [file DataSheet_6.zip › fig 5 raw/fig 5-G raw/inflammation.Gsea.1649955060129/KEGG_WNT_SIGNALING_PATHWAY.html]

Details for gene set KEGG\_WNT\_SIGNALING\_PATHWAY[GSEA]

|  || Dataset | OGD\_DRUG\_DRUG.OGD\_FRUG.cls#Gs\_versus\_MCAO.OGD\_FRUG.cls#Gs\_versus\_MCAO\_repos |
| Phenotype | OGD\_FRUG.cls#Gs\_versus\_MCAO\_repos |
| Upregulated in class | MCAO |
| GeneSet | KEGG\_WNT\_SIGNALING\_PATHWAY |
| Enrichment Score (ES) | -0.4832688 |
| Normalized Enrichment Score (NES) | -1.5177536 |
| Nominal p-value | 0.0 |
| FDR q-value | 0.09111168 |
| FWER p-Value | 0.638 |
Table: GSEA Results Summary

  

Fig 1: Enrichment plot: KEGG\_WNT\_SIGNALING\_PATHWAY      
 Profile of the Running ES Score & Positions of GeneSet Members on the Rank Ordered List

  

| SYMBOL | TITLE | RANK IN GENE LIST | RANK METRIC SCORE | RUNNING ES | CORE ENRICHMENT || 1 | RAC3 | na | 194 | 0.799 | 0.0159 | No |
| 2 | PORCN | na | 658 | 0.537 | 0.0112 | No |
| 3 | RBX1 | na | 1016 | 0.462 | 0.0091 | No |
| 4 | LRP5 | na | 1309 | 0.419 | 0.0087 | No |
| 5 | PRICKLE2 | na | 1356 | 0.415 | 0.0194 | No |
| 6 | WNT10B | na | 1647 | 0.381 | 0.0179 | No |
| 7 | CACYBP | na | 1726 | 0.371 | 0.0258 | No |
| 8 | RHOA | na | 1999 | 0.338 | 0.0238 | No |
| 9 | WNT2B | na | 2097 | 0.328 | 0.0295 | No |
| 10 | WNT4 | na | 2186 | 0.318 | 0.0353 | No |
| 11 | CSNK2B | na | 2192 | 0.316 | 0.0449 | No |
| 12 | CTNNBIP1 | na | 2373 | 0.297 | 0.0458 | No |
| 13 | CSNK2A2 | na | 2818 | 0.258 | 0.0334 | No |
| 14 | FRAT2 | na | 3192 | 0.224 | 0.0232 | No |
| 15 | DAAM2 | na | 3784 | 0.181 | 0.0016 | No |
| 16 | RAC1 | na | 3821 | 0.178 | 0.0054 | No |
| 17 | SFRP4 | na | 3935 | 0.170 | 0.0055 | No |
| 18 | RUVBL1 | na | 4129 | 0.155 | 0.0014 | No |
| 19 | PPP2CB | na | 4186 | 0.150 | 0.0035 | No |
| 20 | CAMK2A | na | 4204 | 0.149 | 0.0073 | No |
| 21 | PRKCG | na | 4221 | 0.148 | 0.0112 | No |
| 22 | PPP3CA | na | 4664 | 0.117 | -0.0055 | No |
| 23 | PPP2R5C | na | 4684 | 0.116 | -0.0028 | No |
| 24 | TCF7 | na | 4825 | 0.106 | -0.0059 | No |
| 25 | WNT7A | na | 5268 | 0.081 | -0.0238 | No |
| 26 | NFATC2 | na | 5286 | 0.080 | -0.0221 | No |
| 27 | WNT11 | na | 5408 | 0.073 | -0.0254 | No |
| 28 | MYC | na | 5587 | 0.064 | -0.0316 | No |
| 29 | JUN | na | 5874 | 0.048 | -0.0433 | No |
| 30 | FOSL1 | na | 6005 | 0.041 | -0.0480 | No |
| 31 | WNT3A | na | 6232 | 0.031 | -0.0574 | No |
| 32 | PPP2CA | na | 6335 | 0.027 | -0.0612 | No |
| 33 | WNT3 | na | 6357 | 0.026 | -0.0614 | No |
| 34 | WNT7B | na | 6508 | 0.021 | -0.0677 | No |
| 35 | WNT16 | na | 6589 | 0.017 | -0.0708 | No |
| 36 | MAPK9 | na | 6752 | 0.011 | -0.0779 | No |
| 37 | SFRP1 | na | 6881 | 0.006 | -0.0836 | No |
| 38 | CCND3 | na | 6975 | 0.002 | -0.0878 | No |
| 39 | CTBP2 | na | 6978 | 0.002 | -0.0879 | No |
| 40 | WNT8A | na | 7038 | 0.000 | -0.0906 | No |
| 41 | DKK1 | na | 7803 | 0.000 | -0.1257 | No |
| 42 | DKK2 | na | 7804 | 0.000 | -0.1257 | No |
| 43 | DKK4 | na | 7805 | 0.000 | -0.1257 | No |
| 44 | CHP2 | na | 7849 | 0.000 | -0.1277 | No |
| 45 | NKD1 | na | 8211 | 0.000 | -0.1443 | No |
| 46 | NKD2 | na | 8212 | 0.000 | -0.1443 | No |
| 47 | AXIN2 | na | 8241 | 0.000 | -0.1456 | No |
| 48 | WIF1 | na | 8770 | 0.000 | -0.1699 | No |
| 49 | SFRP2 | na | 8944 | 0.000 | -0.1779 | No |
| 50 | SFRP5 | na | 8946 | 0.000 | -0.1779 | No |
| 51 | WNT10A | na | 9033 | 0.000 | -0.1819 | No |
| 52 | FZD9 | na | 9034 | 0.000 | -0.1819 | No |
| 53 | FZD8 | na | 9035 | 0.000 | -0.1819 | No |
| 54 | WNT9B | na | 9177 | 0.000 | -0.1883 | No |
| 55 | WNT8B | na | 9179 | 0.000 | -0.1884 | No |
| 56 | WNT6 | na | 9610 | 0.000 | -0.2082 | No |
| 57 | WNT2 | na | 9613 | 0.000 | -0.2083 | No |
| 58 | PPP3R2 | na | 9657 | 0.000 | -0.2103 | No |
| 59 | RAC2 | na | 10018 | 0.000 | -0.2268 | No |
| 60 | FZD10 | na | 11261 | 0.000 | -0.2840 | No |
| 61 | SOX17 | na | 11429 | 0.000 | -0.2916 | No |
| 62 | CXXC4 | na | 12839 | 0.000 | -0.3565 | No |
| 63 | PPP2R5A | na | 13335 | -0.002 | -0.3792 | No |
| 64 | WNT9A | na | 13355 | -0.003 | -0.3800 | No |
| 65 | PRKCB | na | 13570 | -0.009 | -0.3895 | No |
| 66 | PPP3R1 | na | 13636 | -0.011 | -0.3922 | No |
| 67 | PLCB2 | na | 13694 | -0.012 | -0.3944 | No |
| 68 | PPP2R1A | na | 14223 | -0.029 | -0.4178 | No |
| 69 | BTRC | na | 14297 | -0.031 | -0.4202 | No |
| 70 | PPP3CC | na | 14499 | -0.041 | -0.4282 | No |
| 71 | SMAD2 | na | 14505 | -0.041 | -0.4272 | No |
| 72 | CUL1 | na | 14514 | -0.041 | -0.4263 | No |
| 73 | SMAD4 | na | 14652 | -0.048 | -0.4311 | No |
| 74 | CER1 | na | 14683 | -0.050 | -0.4309 | No |
| 75 | NLK | na | 14891 | -0.062 | -0.4385 | No |
| 76 | WNT1 | na | 15037 | -0.070 | -0.4430 | No |
| 77 | MMP7 | na | 15110 | -0.074 | -0.4440 | No |
| 78 | NFATC4 | na | 15169 | -0.078 | -0.4443 | No |
| 79 | CSNK1E | na | 15223 | -0.081 | -0.4442 | No |
| 80 | CSNK2A1 | na | 15329 | -0.088 | -0.4463 | No |
| 81 | CTBP1 | na | 15701 | -0.109 | -0.4600 | No |
| 82 | PRKACA | na | 15891 | -0.121 | -0.4649 | No |
| 83 | TBL1XR1 | na | 16089 | -0.134 | -0.4698 | No |
| 84 | SENP2 | na | 16128 | -0.137 | -0.4674 | No |
| 85 | MAPK10 | na | 16183 | -0.140 | -0.4655 | No |
| 86 | PSEN1 | na | 16462 | -0.159 | -0.4734 | No |
| 87 | CSNK1A1 | na | 16678 | -0.175 | -0.4778 | Yes |
| 88 | LEF1 | na | 16773 | -0.181 | -0.4766 | Yes |
| 89 | WNT5B | na | 16806 | -0.183 | -0.4724 | Yes |
| 90 | PLCB4 | na | 16825 | -0.184 | -0.4675 | Yes |
| 91 | CAMK2D | na | 16846 | -0.185 | -0.4627 | Yes |
| 92 | TCF7L1 | na | 16939 | -0.192 | -0.4609 | Yes |
| 93 | MAP3K7 | na | 17079 | -0.201 | -0.4611 | Yes |
| 94 | PPP3CB | na | 17182 | -0.208 | -0.4593 | Yes |
| 95 | PPP2R5E | na | 17191 | -0.208 | -0.4533 | Yes |
| 96 | CCND1 | na | 17421 | -0.225 | -0.4568 | Yes |
| 97 | FZD2 | na | 17458 | -0.227 | -0.4514 | Yes |
| 98 | DVL2 | na | 17478 | -0.229 | -0.4452 | Yes |
| 99 | VANGL2 | na | 17602 | -0.237 | -0.4435 | Yes |
| 100 | PPP2R5D | na | 17677 | -0.242 | -0.4394 | Yes |
| 101 | PPARD | na | 17742 | -0.248 | -0.4347 | Yes |
| 102 | FZD3 | na | 17855 | -0.258 | -0.4319 | Yes |
| 103 | MAPK8 | na | 17866 | -0.258 | -0.4243 | Yes |
| 104 | DVL1 | na | 17888 | -0.261 | -0.4172 | Yes |
| 105 | PPP2R1B | na | 18017 | -0.271 | -0.4147 | Yes |
| 106 | AXIN1 | na | 18098 | -0.276 | -0.4098 | Yes |
| 107 | PRKCA | na | 18221 | -0.286 | -0.4065 | Yes |
| 108 | FBXW11 | na | 18253 | -0.289 | -0.3990 | Yes |
| 109 | CCND2 | na | 18282 | -0.290 | -0.3913 | Yes |
| 110 | CTNNB1 | na | 18285 | -0.290 | -0.3824 | Yes |
| 111 | CAMK2B | na | 18339 | -0.294 | -0.3757 | Yes |
| 112 | PRKX | na | 18548 | -0.308 | -0.3757 | Yes |
| 113 | CAMK2G | na | 18646 | -0.316 | -0.3704 | Yes |
| 114 | PLCB3 | na | 18848 | -0.334 | -0.3693 | Yes |
| 115 | PRKACB | na | 19453 | -0.392 | -0.3849 | Yes |
| 116 | TCF7L2 | na | 19504 | -0.397 | -0.3749 | Yes |
| 117 | TBL1X | na | 19525 | -0.399 | -0.3635 | Yes |
| 118 | WNT5A | na | 19541 | -0.401 | -0.3517 | Yes |
| 119 | FZD1 | na | 19601 | -0.406 | -0.3419 | Yes |
| 120 | SMAD3 | na | 19611 | -0.408 | -0.3296 | Yes |
| 121 | ROCK1 | na | 19820 | -0.427 | -0.3259 | Yes |
| 122 | DAAM1 | na | 19976 | -0.442 | -0.3194 | Yes |
| 123 | GSK3B | na | 20096 | -0.455 | -0.3107 | Yes |
| 124 | FZD7 | na | 20408 | -0.489 | -0.3099 | Yes |
| 125 | VANGL1 | na | 20444 | -0.495 | -0.2961 | Yes |
| 126 | CHD8 | na | 20466 | -0.498 | -0.2816 | Yes |
| 127 | PPP2R5B | na | 20476 | -0.499 | -0.2666 | Yes |
| 128 | ROCK2 | na | 20637 | -0.521 | -0.2578 | Yes |
| 129 | NFATC3 | na | 20856 | -0.555 | -0.2506 | Yes |
| 130 | DVL3 | na | 20890 | -0.559 | -0.2348 | Yes |
| 131 | NFATC1 | na | 20892 | -0.559 | -0.2175 | Yes |
| 132 | FZD4 | na | 20970 | -0.572 | -0.2033 | Yes |
| 133 | APC | na | 21200 | -0.619 | -0.1946 | Yes |
| 134 | FZD5 | na | 21229 | -0.627 | -0.1765 | Yes |
| 135 | LRP6 | na | 21288 | -0.639 | -0.1593 | Yes |
| 136 | CREBBP | na | 21304 | -0.643 | -0.1401 | Yes |
| 137 | NFAT5 | na | 21361 | -0.657 | -0.1223 | Yes |
| 138 | PRICKLE1 | na | 21413 | -0.674 | -0.1037 | Yes |
| 139 | EP300 | na | 21416 | -0.674 | -0.0829 | Yes |
| 140 | APC2 | na | 21564 | -0.733 | -0.0670 | Yes |
| 141 | PLCB1 | na | 21572 | -0.736 | -0.0445 | Yes |
| 142 | FRAT1 | na | 21598 | -0.755 | -0.0222 | Yes |
| 143 | FZD6 | na | 21825 | -1.125 | 0.0023 | Yes |
Table: GSEA details [plain text format]

  

Fig 2: KEGG\_WNT\_SIGNALING\_PATHWAY      
 Blue-Pink O' Gram in the Space of the Analyzed GeneSet

  

Fig 3: KEGG\_WNT\_SIGNALING\_PATHWAY: Random ES distribution      
 Gene set null distribution of ES for **KEGG\_WNT\_SIGNALING\_PATHWAY**
