# Supplementary material for: Regulation of microglia related neuroinflammation contributes to the protective effect of Gelsevirine on ischemic stroke
Source: Front Immunol. 2023 Mar 30;14:1164278. doi: 10.3389/fimmu.2023.1164278 (PMC10098192; doi:10.3389/fimmu.2023.1164278)
Supplement: Supplementary file 6 [file DataSheet_6.zip › fig 5 raw/fig 5-G raw/inflammation.Gsea.1649955060129/KENNY_CTNNB1_TARGETS_UP.html]

Details for gene set KENNY\_CTNNB1\_TARGETS\_UP[GSEA]

|  || Dataset | OGD\_DRUG\_DRUG.OGD\_FRUG.cls#Gs\_versus\_MCAO.OGD\_FRUG.cls#Gs\_versus\_MCAO\_repos |
| Phenotype | OGD\_FRUG.cls#Gs\_versus\_MCAO\_repos |
| Upregulated in class | Gs |
| GeneSet | KENNY\_CTNNB1\_TARGETS\_UP |
| Enrichment Score (ES) | 0.27401727 |
| Normalized Enrichment Score (NES) | 0.80573815 |
| Nominal p-value | 0.9142012 |
| FDR q-value | 0.9578638 |
| FWER p-Value | 1.0 |
Table: GSEA Results Summary

  

Fig 1: Enrichment plot: KENNY\_CTNNB1\_TARGETS\_UP      
 Profile of the Running ES Score & Positions of GeneSet Members on the Rank Ordered List

  

| SYMBOL | TITLE | RANK IN GENE LIST | RANK METRIC SCORE | RUNNING ES | CORE ENRICHMENT || 1 | S100A8 | na | 94 | 1.012 | 0.0666 | Yes |
| 2 | RAMP3 | na | 310 | 0.693 | 0.1053 | Yes |
| 3 | LOXL2 | na | 652 | 0.539 | 0.1274 | Yes |
| 4 | RPA2 | na | 1039 | 0.459 | 0.1418 | Yes |
| 5 | EBNA1BP2 | na | 1318 | 0.418 | 0.1583 | Yes |
| 6 | PTPN22 | na | 1550 | 0.393 | 0.1753 | Yes |
| 7 | HMGB2 | na | 1731 | 0.370 | 0.1930 | Yes |
| 8 | HAT1 | na | 1874 | 0.352 | 0.2111 | Yes |
| 9 | EXOSC2 | na | 2115 | 0.325 | 0.2229 | Yes |
| 10 | SRSF7 | na | 2163 | 0.321 | 0.2432 | Yes |
| 11 | TM4SF1 | na | 2719 | 0.268 | 0.2365 | Yes |
| 12 | SQRDL | na | 2820 | 0.258 | 0.2500 | Yes |
| 13 | UPP1 | na | 3208 | 0.222 | 0.2478 | Yes |
| 14 | SPP1 | na | 3259 | 0.218 | 0.2608 | Yes |
| 15 | TK1 | na | 3596 | 0.192 | 0.2588 | Yes |
| 16 | BAD | na | 3658 | 0.189 | 0.2693 | Yes |
| 17 | TACC3 | na | 3900 | 0.173 | 0.2703 | Yes |
| 18 | SEPHS2 | na | 4063 | 0.159 | 0.2740 | Yes |
| 19 | CD14 | na | 4569 | 0.124 | 0.2596 | No |
| 20 | IGFBP5 | na | 5917 | 0.046 | 0.2011 | No |
| 21 | CCR5 | na | 12896 | 0.000 | -0.1186 | No |
| 22 | CTSC | na | 13557 | -0.008 | -0.1482 | No |
| 23 | ETAA1 | na | 13873 | -0.017 | -0.1615 | No |
| 24 | BTRC | na | 14297 | -0.031 | -0.1787 | No |
| 25 | CAPN3 | na | 14698 | -0.050 | -0.1935 | No |
| 26 | ABI1 | na | 14894 | -0.062 | -0.1981 | No |
| 27 | CYR61 | na | 15121 | -0.075 | -0.2032 | No |
| 28 | TNFAIP2 | na | 15268 | -0.084 | -0.2040 | No |
| 29 | CCNB1 | na | 15509 | -0.098 | -0.2081 | No |
| 30 | PLK4 | na | 16699 | -0.177 | -0.2502 | No |
| 31 | SORBS1 | na | 17043 | -0.198 | -0.2521 | No |
| 32 | PKN1 | na | 17142 | -0.205 | -0.2422 | No |
| 33 | ARCN1 | na | 17439 | -0.226 | -0.2399 | No |
| 34 | CSE1L | na | 17547 | -0.233 | -0.2285 | No |
| 35 | TFDP1 | na | 18032 | -0.271 | -0.2317 | No |
| 36 | MMP2 | na | 18057 | -0.273 | -0.2136 | No |
| 37 | GNA11 | na | 18153 | -0.281 | -0.1983 | No |
| 38 | ECT2 | na | 19250 | -0.373 | -0.2224 | No |
| 39 | RBM26 | na | 19353 | -0.382 | -0.2003 | No |
| 40 | SERPIND1 | na | 19420 | -0.390 | -0.1760 | No |
| 41 | ARFGEF2 | na | 19954 | -0.440 | -0.1696 | No |
| 42 | CELF2 | na | 20281 | -0.475 | -0.1513 | No |
| 43 | CASKIN2 | na | 20504 | -0.503 | -0.1262 | No |
| 44 | SYNE1 | na | 21016 | -0.582 | -0.1089 | No |
| 45 | NCOR1 | na | 21036 | -0.585 | -0.0688 | No |
| 46 | GAN | na | 21482 | -0.698 | -0.0403 | No |
| 47 | KRT7 | na | 21689 | -0.831 | 0.0085 | No |
Table: GSEA details [plain text format]

  

Fig 2: KENNY\_CTNNB1\_TARGETS\_UP      
 Blue-Pink O' Gram in the Space of the Analyzed GeneSet

  

Fig 3: KENNY\_CTNNB1\_TARGETS\_UP: Random ES distribution      
 Gene set null distribution of ES for **KENNY\_CTNNB1\_TARGETS\_UP**
