# Supplementary material for: Regulation of microglia related neuroinflammation contributes to the protective effect of Gelsevirine on ischemic stroke
Source: Front Immunol. 2023 Mar 30;14:1164278. doi: 10.3389/fimmu.2023.1164278 (PMC10098192; doi:10.3389/fimmu.2023.1164278)
Supplement: Supplementary file 6 [file DataSheet_6.zip › fig 5 raw/fig 5-G raw/inflammation.Gsea.1649955060129/NUMATA_CSF3_SIGNALING_VIA_STAT3.html]

Details for gene set NUMATA\_CSF3\_SIGNALING\_VIA\_STAT3[GSEA]

|  || Dataset | OGD\_DRUG\_DRUG.OGD\_FRUG.cls#Gs\_versus\_MCAO.OGD\_FRUG.cls#Gs\_versus\_MCAO\_repos |
| Phenotype | OGD\_FRUG.cls#Gs\_versus\_MCAO\_repos |
| Upregulated in class | Gs |
| GeneSet | NUMATA\_CSF3\_SIGNALING\_VIA\_STAT3 |
| Enrichment Score (ES) | 0.49848315 |
| Normalized Enrichment Score (NES) | 1.2230337 |
| Nominal p-value | 0.15079366 |
| FDR q-value | 0.32251763 |
| FWER p-Value | 0.994 |
Table: GSEA Results Summary

  

Fig 1: Enrichment plot: NUMATA\_CSF3\_SIGNALING\_VIA\_STAT3      
 Profile of the Running ES Score & Positions of GeneSet Members on the Rank Ordered List

  

| SYMBOL | TITLE | RANK IN GENE LIST | RANK METRIC SCORE | RUNNING ES | CORE ENRICHMENT || 1 | ITGAL | na | 44 | 1.259 | 0.2117 | Yes |
| 2 | RORA | na | 154 | 0.852 | 0.3514 | Yes |
| 3 | BLNK | na | 439 | 0.617 | 0.4432 | Yes |
| 4 | PTPN13 | na | 969 | 0.468 | 0.4985 | Yes |
| 5 | MADCAM1 | na | 4984 | 0.096 | 0.3312 | No |
| 6 | NINJ1 | na | 5346 | 0.077 | 0.3277 | No |
| 7 | HTR3A | na | 7191 | 0.000 | 0.2433 | No |
| 8 | CEBPA | na | 7443 | 0.000 | 0.2318 | No |
| 9 | MST1R | na | 13631 | -0.011 | -0.0495 | No |
| 10 | MKNK1 | na | 13934 | -0.018 | -0.0602 | No |
| 11 | FLI1 | na | 14298 | -0.032 | -0.0715 | No |
| 12 | MAP6 | na | 14919 | -0.063 | -0.0891 | No |
| 13 | SRPK1 | na | 15842 | -0.118 | -0.1113 | No |
| 14 | IL17RA | na | 16705 | -0.177 | -0.1207 | No |
| 15 | CCNE1 | na | 16729 | -0.178 | -0.0915 | No |
| 16 | RUNX1 | na | 16837 | -0.185 | -0.0650 | No |
| 17 | BCL2 | na | 17522 | -0.232 | -0.0570 | No |
| 18 | TFRC | na | 18581 | -0.311 | -0.0527 | No |
| 19 | MAP2K7 | na | 18727 | -0.323 | -0.0045 | No |
| 20 | KIF3B | na | 19222 | -0.371 | 0.0360 | No |
| 21 | MECOM | na | 20510 | -0.503 | 0.0625 | No |
Table: GSEA details [plain text format]

  

Fig 2: NUMATA\_CSF3\_SIGNALING\_VIA\_STAT3      
 Blue-Pink O' Gram in the Space of the Analyzed GeneSet

  

Fig 3: NUMATA\_CSF3\_SIGNALING\_VIA\_STAT3: Random ES distribution      
 Gene set null distribution of ES for **NUMATA\_CSF3\_SIGNALING\_VIA\_STAT3**
