# Supplementary material for: Regulation of microglia related neuroinflammation contributes to the protective effect of Gelsevirine on ischemic stroke
Source: Front Immunol. 2023 Mar 30;14:1164278. doi: 10.3389/fimmu.2023.1164278 (PMC10098192; doi:10.3389/fimmu.2023.1164278)
Supplement: Supplementary file 6 [file DataSheet_6.zip › fig 5 raw/fig 5-G raw/inflammation.Gsea.1649955060129/PARENT_MTOR_SIGNALING_DN.html]

Details for gene set PARENT\_MTOR\_SIGNALING\_DN[GSEA]

|  || Dataset | OGD\_DRUG\_DRUG.OGD\_FRUG.cls#Gs\_versus\_MCAO.OGD\_FRUG.cls#Gs\_versus\_MCAO\_repos |
| Phenotype | OGD\_FRUG.cls#Gs\_versus\_MCAO\_repos |
| Upregulated in class | Gs |
| GeneSet | PARENT\_MTOR\_SIGNALING\_DN |
| Enrichment Score (ES) | 0.30312407 |
| Normalized Enrichment Score (NES) | 0.8425117 |
| Nominal p-value | 0.7417417 |
| FDR q-value | 0.9350381 |
| FWER p-Value | 1.0 |
Table: GSEA Results Summary

  

Fig 1: Enrichment plot: PARENT\_MTOR\_SIGNALING\_DN      
 Profile of the Running ES Score & Positions of GeneSet Members on the Rank Ordered List

  

| SYMBOL | TITLE | RANK IN GENE LIST | RANK METRIC SCORE | RUNNING ES | CORE ENRICHMENT || 1 | CRABP2 | na | 117 | 0.951 | 0.0856 | Yes |
| 2 | SLC26A10 | na | 355 | 0.653 | 0.1373 | Yes |
| 3 | KRT86 | na | 442 | 0.616 | 0.1923 | Yes |
| 4 | ENGASE | na | 829 | 0.492 | 0.2217 | Yes |
| 5 | ITGAM | na | 956 | 0.470 | 0.2609 | Yes |
| 6 | RPL12 | na | 1004 | 0.464 | 0.3031 | Yes |
| 7 | LSM4 | na | 1859 | 0.355 | 0.2980 | No |
| 8 | EREG | na | 3001 | 0.241 | 0.2688 | No |
| 9 | MYL4 | na | 3622 | 0.191 | 0.2586 | No |
| 10 | RGS16 | na | 3743 | 0.183 | 0.2706 | No |
| 11 | DNAJC4 | na | 4112 | 0.155 | 0.2687 | No |
| 12 | PTCRA | na | 4220 | 0.148 | 0.2779 | No |
| 13 | VEGFA | na | 5039 | 0.094 | 0.2494 | No |
| 14 | EIF5 | na | 5939 | 0.045 | 0.2125 | No |
| 15 | AQP6 | na | 6217 | 0.032 | 0.2029 | No |
| 16 | HGF | na | 8228 | 0.000 | 0.1108 | No |
| 17 | BMP2 | na | 9002 | 0.000 | 0.0754 | No |
| 18 | CXCL3 | na | 9141 | 0.000 | 0.0691 | No |
| 19 | TOX3 | na | 11692 | 0.000 | -0.0477 | No |
| 20 | CNTN1 | na | 11779 | 0.000 | -0.0516 | No |
| 21 | FGF5 | na | 11854 | 0.000 | -0.0550 | No |
| 22 | NRCAM | na | 12662 | 0.000 | -0.0920 | No |
| 23 | FGF2 | na | 13903 | -0.017 | -0.1471 | No |
| 24 | CEACAM1 | na | 14089 | -0.023 | -0.1534 | No |
| 25 | MMP24 | na | 14401 | -0.036 | -0.1642 | No |
| 26 | POLR3G | na | 14958 | -0.065 | -0.1834 | No |
| 27 | SSX2IP | na | 15249 | -0.083 | -0.1887 | No |
| 28 | ELOVL5 | na | 15983 | -0.127 | -0.2101 | No |
| 29 | CNN1 | na | 16792 | -0.182 | -0.2297 | No |
| 30 | SPN | na | 16941 | -0.192 | -0.2181 | No |
| 31 | FHL3 | na | 17319 | -0.218 | -0.2145 | No |
| 32 | PDGFB | na | 17563 | -0.234 | -0.2032 | No |
| 33 | CD248 | na | 18504 | -0.304 | -0.2172 | No |
| 34 | MPRIP | na | 19053 | -0.355 | -0.2084 | No |
| 35 | TGFB3 | na | 19860 | -0.432 | -0.2040 | No |
| 36 | SLC12A6 | na | 20335 | -0.481 | -0.1796 | No |
| 37 | ADAM19 | na | 20509 | -0.503 | -0.1394 | No |
| 38 | ALMS1 | na | 21050 | -0.588 | -0.1079 | No |
| 39 | ANKRD2 | na | 21567 | -0.735 | -0.0612 | No |
| 40 | WFDC2 | na | 21647 | -0.787 | 0.0104 | No |
Table: GSEA details [plain text format]

  

Fig 2: PARENT\_MTOR\_SIGNALING\_DN      
 Blue-Pink O' Gram in the Space of the Analyzed GeneSet

  

Fig 3: PARENT\_MTOR\_SIGNALING\_DN: Random ES distribution      
 Gene set null distribution of ES for **PARENT\_MTOR\_SIGNALING\_DN**
