# Supplementary material for: Regulation of microglia related neuroinflammation contributes to the protective effect of Gelsevirine on ischemic stroke
Source: Front Immunol. 2023 Mar 30;14:1164278. doi: 10.3389/fimmu.2023.1164278 (PMC10098192; doi:10.3389/fimmu.2023.1164278)
Supplement: Supplementary file 6 [file DataSheet_6.zip › fig 5 raw/fig 5-G raw/inflammation.Gsea.1649955060129/PHONG_TNF_RESPONSE_VIA_P38_COMPLETE.html]

Details for gene set PHONG\_TNF\_RESPONSE\_VIA\_P38\_COMPLETE[GSEA]

|  || Dataset | OGD\_DRUG\_DRUG.OGD\_FRUG.cls#Gs\_versus\_MCAO.OGD\_FRUG.cls#Gs\_versus\_MCAO\_repos |
| Phenotype | OGD\_FRUG.cls#Gs\_versus\_MCAO\_repos |
| Upregulated in class | MCAO |
| GeneSet | PHONG\_TNF\_RESPONSE\_VIA\_P38\_COMPLETE |
| Enrichment Score (ES) | -0.4842248 |
| Normalized Enrichment Score (NES) | -1.5672473 |
| Nominal p-value | 0.0 |
| FDR q-value | 0.07371229 |
| FWER p-Value | 0.44 |
Table: GSEA Results Summary

  

Fig 1: Enrichment plot: PHONG\_TNF\_RESPONSE\_VIA\_P38\_COMPLETE      
 Profile of the Running ES Score & Positions of GeneSet Members on the Rank Ordered List

  

| SYMBOL | TITLE | RANK IN GENE LIST | RANK METRIC SCORE | RUNNING ES | CORE ENRICHMENT || 1 | HTATIP2 | na | 200 | 0.797 | 0.0038 | No |
| 2 | S100A4 | na | 373 | 0.645 | 0.0064 | No |
| 3 | DDIT3 | na | 490 | 0.591 | 0.0107 | No |
| 4 | PPP1R15A | na | 573 | 0.564 | 0.0162 | No |
| 5 | THYN1 | na | 662 | 0.536 | 0.0209 | No |
| 6 | TUFT1 | na | 889 | 0.479 | 0.0183 | No |
| 7 | EHD3 | na | 908 | 0.477 | 0.0252 | No |
| 8 | ATF3 | na | 1001 | 0.464 | 0.0286 | No |
| 9 | FANCL | na | 1171 | 0.438 | 0.0280 | No |
| 10 | PCLO | na | 1323 | 0.417 | 0.0278 | No |
| 11 | KCNK1 | na | 1487 | 0.401 | 0.0269 | No |
| 12 | FOS | na | 1505 | 0.399 | 0.0326 | No |
| 13 | EGR2 | na | 1635 | 0.382 | 0.0329 | No |
| 14 | NDUFB2 | na | 1907 | 0.348 | 0.0261 | No |
| 15 | MSMO1 | na | 1929 | 0.345 | 0.0308 | No |
| 16 | FABP4 | na | 2117 | 0.325 | 0.0274 | No |
| 17 | PNP | na | 2244 | 0.310 | 0.0267 | No |
| 18 | DERL2 | na | 2278 | 0.306 | 0.0302 | No |
| 19 | KCNMA1 | na | 2554 | 0.282 | 0.0221 | No |
| 20 | DHFR | na | 2621 | 0.277 | 0.0236 | No |
| 21 | TMEM223 | na | 2740 | 0.265 | 0.0225 | No |
| 22 | PCNA | na | 2828 | 0.257 | 0.0227 | No |
| 23 | CKLF | na | 2833 | 0.257 | 0.0267 | No |
| 24 | CCDC85B | na | 2838 | 0.256 | 0.0307 | No |
| 25 | YIPF5 | na | 2870 | 0.253 | 0.0334 | No |
| 26 | GLIPR1 | na | 2893 | 0.251 | 0.0365 | No |
| 27 | TCP1 | na | 3077 | 0.235 | 0.0319 | No |
| 28 | GABARAPL1 | na | 3089 | 0.234 | 0.0352 | No |
| 29 | HHEX | na | 3216 | 0.221 | 0.0330 | No |
| 30 | DIO2 | na | 3237 | 0.219 | 0.0357 | No |
| 31 | IL11 | na | 3251 | 0.219 | 0.0386 | No |
| 32 | BTG1 | na | 3294 | 0.215 | 0.0402 | No |
| 33 | EGR1 | na | 3562 | 0.195 | 0.0311 | No |
| 34 | CCNL1 | na | 3974 | 0.167 | 0.0148 | No |
| 35 | NUDT4 | na | 4020 | 0.163 | 0.0154 | No |
| 36 | MAGT1 | na | 4024 | 0.162 | 0.0179 | No |
| 37 | MCL1 | na | 4123 | 0.155 | 0.0159 | No |
| 38 | TMEM97 | na | 4449 | 0.133 | 0.0031 | No |
| 39 | SDCBP | na | 4627 | 0.120 | -0.0031 | No |
| 40 | GTPBP4 | na | 4736 | 0.113 | -0.0063 | No |
| 41 | PNRC2 | na | 4753 | 0.111 | -0.0052 | No |
| 42 | CD82 | na | 4840 | 0.106 | -0.0074 | No |
| 43 | FAIM | na | 4880 | 0.103 | -0.0075 | No |
| 44 | IDH1 | na | 4951 | 0.098 | -0.0092 | No |
| 45 | PMAIP1 | na | 4999 | 0.096 | -0.0098 | No |
| 46 | ACSL3 | na | 5200 | 0.085 | -0.0176 | No |
| 47 | STK11 | na | 5246 | 0.082 | -0.0183 | No |
| 48 | PITPNA | na | 5337 | 0.077 | -0.0212 | No |
| 49 | NINJ1 | na | 5346 | 0.077 | -0.0203 | No |
| 50 | VPS35 | na | 5360 | 0.076 | -0.0197 | No |
| 51 | MNS1 | na | 5392 | 0.074 | -0.0199 | No |
| 52 | RAB22A | na | 5847 | 0.049 | -0.0401 | No |
| 53 | SNX2 | na | 5899 | 0.047 | -0.0417 | No |
| 54 | IGFBP5 | na | 5917 | 0.046 | -0.0417 | No |
| 55 | SKP2 | na | 5968 | 0.043 | -0.0433 | No |
| 56 | U2SURP | na | 6084 | 0.037 | -0.0480 | No |
| 57 | CDK2 | na | 6514 | 0.020 | -0.0675 | No |
| 58 | BCL3 | na | 6597 | 0.017 | -0.0710 | No |
| 59 | MGAT2 | na | 6639 | 0.015 | -0.0726 | No |
| 60 | MCM6 | na | 6932 | 0.004 | -0.0860 | No |
| 61 | TNFRSF11B | na | 7622 | 0.000 | -0.1178 | No |
| 62 | NCALD | na | 8563 | 0.000 | -0.1612 | No |
| 63 | CDH12 | na | 8800 | 0.000 | -0.1721 | No |
| 64 | KLRC3 | na | 9138 | 0.000 | -0.1877 | No |
| 65 | GABRA2 | na | 9338 | 0.000 | -0.1969 | No |
| 66 | TMEM51 | na | 9479 | 0.000 | -0.2033 | No |
| 67 | IL7R | na | 10988 | 0.000 | -0.2729 | No |
| 68 | RGS1 | na | 11368 | 0.000 | -0.2904 | No |
| 69 | HOXA9 | na | 11428 | 0.000 | -0.2932 | No |
| 70 | TGIF1 | na | 13271 | -0.001 | -0.3782 | No |
| 71 | RET | na | 13693 | -0.012 | -0.3974 | No |
| 72 | SYNM | na | 13927 | -0.018 | -0.4079 | No |
| 73 | TMEM87A | na | 14067 | -0.023 | -0.4139 | No |
| 74 | MTHFD2L | na | 14173 | -0.027 | -0.4183 | No |
| 75 | HOXA10 | na | 14222 | -0.029 | -0.4201 | No |
| 76 | SERPINE2 | na | 14512 | -0.041 | -0.4327 | No |
| 77 | ETS2 | na | 14586 | -0.045 | -0.4354 | No |
| 78 | SIKE1 | na | 14669 | -0.049 | -0.4384 | No |
| 79 | SV2A | na | 14686 | -0.050 | -0.4383 | No |
| 80 | ARHGAP12 | na | 14784 | -0.055 | -0.4418 | No |
| 81 | INSIG1 | na | 14787 | -0.055 | -0.4410 | No |
| 82 | SSBP2 | na | 14821 | -0.057 | -0.4416 | No |
| 83 | RHOQ | na | 14853 | -0.059 | -0.4421 | No |
| 84 | UBE2J1 | na | 14909 | -0.063 | -0.4436 | No |
| 85 | TMEM135 | na | 14912 | -0.063 | -0.4427 | No |
| 86 | RAP2C | na | 14991 | -0.067 | -0.4452 | No |
| 87 | EMP2 | na | 15070 | -0.072 | -0.4476 | No |
| 88 | CYR61 | na | 15121 | -0.075 | -0.4487 | No |
| 89 | ZFP36 | na | 15153 | -0.077 | -0.4488 | No |
| 90 | CSNK1E | na | 15223 | -0.081 | -0.4507 | No |
| 91 | PAQR4 | na | 15288 | -0.085 | -0.4522 | No |
| 92 | AP1G2 | na | 15418 | -0.093 | -0.4567 | No |
| 93 | B4GALT1 | na | 15457 | -0.095 | -0.4569 | No |
| 94 | TRAF4 | na | 15537 | -0.100 | -0.4589 | No |
| 95 | CSGALNACT1 | na | 15573 | -0.102 | -0.4588 | No |
| 96 | WRB | na | 15605 | -0.104 | -0.4585 | No |
| 97 | MAFG | na | 15768 | -0.113 | -0.4642 | No |
| 98 | TRIM24 | na | 15880 | -0.120 | -0.4673 | No |
| 99 | DLST | na | 15972 | -0.127 | -0.4695 | No |
| 100 | ACOX1 | na | 16007 | -0.129 | -0.4689 | No |
| 101 | CAV1 | na | 16173 | -0.139 | -0.4743 | No |
| 102 | SS18 | na | 16390 | -0.154 | -0.4817 | Yes |
| 103 | EXT1 | na | 16426 | -0.156 | -0.4808 | Yes |
| 104 | PTGS2 | na | 16449 | -0.158 | -0.4792 | Yes |
| 105 | ARL4C | na | 16456 | -0.158 | -0.4769 | Yes |
| 106 | ZBTB5 | na | 16584 | -0.168 | -0.4800 | Yes |
| 107 | SLC9A6 | na | 16636 | -0.172 | -0.4796 | Yes |
| 108 | ING1 | na | 16725 | -0.178 | -0.4807 | Yes |
| 109 | ENAH | na | 16759 | -0.180 | -0.4793 | Yes |
| 110 | RBM19 | na | 16763 | -0.180 | -0.4765 | Yes |
| 111 | GOLPH3L | na | 16786 | -0.182 | -0.4745 | Yes |
| 112 | CTDSPL | na | 16847 | -0.185 | -0.4743 | Yes |
| 113 | CRK | na | 16856 | -0.186 | -0.4716 | Yes |
| 114 | LGALS8 | na | 16864 | -0.187 | -0.4689 | Yes |
| 115 | PTPN12 | na | 17004 | -0.197 | -0.4721 | Yes |
| 116 | MBD4 | na | 17075 | -0.201 | -0.4720 | Yes |
| 117 | FBXL5 | na | 17144 | -0.205 | -0.4718 | Yes |
| 118 | PRKD1 | na | 17256 | -0.213 | -0.4734 | Yes |
| 119 | ITGA5 | na | 17393 | -0.223 | -0.4761 | Yes |
| 120 | IKBKE | na | 17396 | -0.223 | -0.4725 | Yes |
| 121 | LIMCH1 | na | 17453 | -0.226 | -0.4714 | Yes |
| 122 | FZD2 | na | 17458 | -0.227 | -0.4679 | Yes |
| 123 | FOXC1 | na | 17461 | -0.227 | -0.4642 | Yes |
| 124 | CAPN7 | na | 17698 | -0.244 | -0.4711 | Yes |
| 125 | ALDH3A2 | na | 17759 | -0.249 | -0.4698 | Yes |
| 126 | SEC24A | na | 17852 | -0.257 | -0.4699 | Yes |
| 127 | ETV5 | na | 17877 | -0.259 | -0.4667 | Yes |
| 128 | CDC42EP4 | na | 17966 | -0.267 | -0.4664 | Yes |
| 129 | NFATC2IP | na | 18031 | -0.271 | -0.4650 | Yes |
| 130 | KLF10 | na | 18179 | -0.283 | -0.4671 | Yes |
| 131 | MAP7D1 | na | 18208 | -0.285 | -0.4637 | Yes |
| 132 | ATP2B1 | na | 18239 | -0.288 | -0.4604 | Yes |
| 133 | MCAM | na | 18296 | -0.291 | -0.4583 | Yes |
| 134 | DUSP5 | na | 18336 | -0.293 | -0.4553 | Yes |
| 135 | ADAM17 | na | 18595 | -0.311 | -0.4621 | Yes |
| 136 | CBX4 | na | 18604 | -0.312 | -0.4574 | Yes |
| 137 | SH3BP5 | na | 18606 | -0.312 | -0.4523 | Yes |
| 138 | MYO1B | na | 18632 | -0.315 | -0.4483 | Yes |
| 139 | TCF4 | na | 18652 | -0.317 | -0.4440 | Yes |
| 140 | JRK | na | 18661 | -0.317 | -0.4392 | Yes |
| 141 | ELOVL6 | na | 18683 | -0.319 | -0.4349 | Yes |
| 142 | HMGCR | na | 18840 | -0.333 | -0.4367 | Yes |
| 143 | ERAP1 | na | 18928 | -0.342 | -0.4351 | Yes |
| 144 | NR3C1 | na | 18932 | -0.342 | -0.4296 | Yes |
| 145 | LARP1 | na | 18994 | -0.348 | -0.4268 | Yes |
| 146 | SEMA4C | na | 18998 | -0.348 | -0.4212 | Yes |
| 147 | OBSL1 | na | 19040 | -0.354 | -0.4173 | Yes |
| 148 | SHC1 | na | 19109 | -0.361 | -0.4146 | Yes |
| 149 | JAG1 | na | 19110 | -0.361 | -0.4087 | Yes |
| 150 | NKTR | na | 19197 | -0.369 | -0.4066 | Yes |
| 151 | SMURF1 | na | 19205 | -0.370 | -0.4009 | Yes |
| 152 | MAP4K4 | na | 19223 | -0.371 | -0.3956 | Yes |
| 153 | PPP1R12A | na | 19275 | -0.375 | -0.3918 | Yes |
| 154 | MAP3K11 | na | 19289 | -0.377 | -0.3862 | Yes |
| 155 | MKNK2 | na | 19425 | -0.390 | -0.3861 | Yes |
| 156 | KDM2A | na | 19429 | -0.390 | -0.3798 | Yes |
| 157 | WNT5A | na | 19541 | -0.401 | -0.3784 | Yes |
| 158 | MYO1C | na | 19568 | -0.404 | -0.3730 | Yes |
| 159 | SLC7A1 | na | 19573 | -0.404 | -0.3666 | Yes |
| 160 | MID1 | na | 19661 | -0.413 | -0.3638 | Yes |
| 161 | GFPT1 | na | 19683 | -0.415 | -0.3580 | Yes |
| 162 | NEO1 | na | 19716 | -0.418 | -0.3527 | Yes |
| 163 | FOSL2 | na | 19737 | -0.420 | -0.3467 | Yes |
| 164 | NUP210 | na | 19761 | -0.422 | -0.3409 | Yes |
| 165 | SPAG9 | na | 19774 | -0.424 | -0.3345 | Yes |
| 166 | CDC42EP1 | na | 19827 | -0.428 | -0.3299 | Yes |
| 167 | PSD3 | na | 19837 | -0.429 | -0.3233 | Yes |
| 168 | ZCCHC6 | na | 19854 | -0.431 | -0.3170 | Yes |
| 169 | WIZ | na | 19921 | -0.437 | -0.3129 | Yes |
| 170 | DYRK2 | na | 20008 | -0.446 | -0.3096 | Yes |
| 171 | OTUD4 | na | 20038 | -0.449 | -0.3036 | Yes |
| 172 | PTPRK | na | 20083 | -0.453 | -0.2982 | Yes |
| 173 | SATB2 | na | 20163 | -0.463 | -0.2943 | Yes |
| 174 | BAZ1A | na | 20211 | -0.468 | -0.2888 | Yes |
| 175 | UBR2 | na | 20251 | -0.472 | -0.2829 | Yes |
| 176 | ZMIZ2 | na | 20304 | -0.478 | -0.2775 | Yes |
| 177 | ARID4B | na | 20353 | -0.483 | -0.2718 | Yes |
| 178 | SLC5A3 | na | 20425 | -0.492 | -0.2670 | Yes |
| 179 | PHIP | na | 20436 | -0.495 | -0.2594 | Yes |
| 180 | MLXIP | na | 20484 | -0.500 | -0.2534 | Yes |
| 181 | MECOM | na | 20510 | -0.503 | -0.2463 | Yes |
| 182 | TCF20 | na | 20511 | -0.503 | -0.2380 | Yes |
| 183 | SOS1 | na | 20563 | -0.511 | -0.2321 | Yes |
| 184 | CEP68 | na | 20609 | -0.517 | -0.2257 | Yes |
| 185 | AFF1 | na | 20610 | -0.518 | -0.2172 | Yes |
| 186 | ZCCHC24 | na | 20625 | -0.520 | -0.2093 | Yes |
| 187 | ITSN2 | na | 20698 | -0.529 | -0.2040 | Yes |
| 188 | PPM1F | na | 20737 | -0.535 | -0.1970 | Yes |
| 189 | NOTCH1 | na | 20749 | -0.537 | -0.1887 | Yes |
| 190 | SVIL | na | 20754 | -0.538 | -0.1801 | Yes |
| 191 | SEC24D | na | 20815 | -0.548 | -0.1739 | Yes |
| 192 | ZMYND8 | na | 20820 | -0.549 | -0.1651 | Yes |
| 193 | BACH1 | na | 20837 | -0.552 | -0.1568 | Yes |
| 194 | WWC2 | na | 20864 | -0.556 | -0.1490 | Yes |
| 195 | KDM6B | na | 20925 | -0.564 | -0.1425 | Yes |
| 196 | FBRS | na | 20945 | -0.568 | -0.1341 | Yes |
| 197 | NCOA2 | na | 20947 | -0.569 | -0.1248 | Yes |
| 198 | NAV3 | na | 20978 | -0.573 | -0.1168 | Yes |
| 199 | HMBOX1 | na | 21058 | -0.589 | -0.1108 | Yes |
| 200 | RPRD2 | na | 21100 | -0.598 | -0.1029 | Yes |
| 201 | ACACA | na | 21178 | -0.614 | -0.0964 | Yes |
| 202 | CUX1 | na | 21277 | -0.638 | -0.0905 | Yes |
| 203 | PBX1 | na | 21314 | -0.646 | -0.0816 | Yes |
| 204 | VCAN | na | 21332 | -0.652 | -0.0717 | Yes |
| 205 | GLG1 | na | 21346 | -0.655 | -0.0616 | Yes |
| 206 | AGRN | na | 21378 | -0.662 | -0.0522 | Yes |
| 207 | MYO10 | na | 21477 | -0.696 | -0.0454 | Yes |
| 208 | HDAC9 | na | 21533 | -0.719 | -0.0361 | Yes |
| 209 | KAT6B | na | 21585 | -0.746 | -0.0263 | Yes |
| 210 | DLG5 | na | 21605 | -0.760 | -0.0148 | Yes |
| 211 | SCHIP1 | na | 21613 | -0.763 | -0.0026 | Yes |
| 212 | MBP | na | 21744 | -0.895 | 0.0060 | Yes |
Table: GSEA details [plain text format]

  

Fig 2: PHONG\_TNF\_RESPONSE\_VIA\_P38\_COMPLETE      
 Blue-Pink O' Gram in the Space of the Analyzed GeneSet

  

Fig 3: PHONG\_TNF\_RESPONSE\_VIA\_P38\_COMPLETE: Random ES distribution      
 Gene set null distribution of ES for **PHONG\_TNF\_RESPONSE\_VIA\_P38\_COMPLETE**
