# Supplementary material for: Regulation of microglia related neuroinflammation contributes to the protective effect of Gelsevirine on ischemic stroke
Source: Front Immunol. 2023 Mar 30;14:1164278. doi: 10.3389/fimmu.2023.1164278 (PMC10098192; doi:10.3389/fimmu.2023.1164278)
Supplement: Supplementary file 6 [file DataSheet_6.zip › fig 5 raw/fig 5-G raw/inflammation.Gsea.1649955060129/PID_BETA_CATENIN_NUC_PATHWAY.html]

Details for gene set PID\_BETA\_CATENIN\_NUC\_PATHWAY[GSEA]

|  || Dataset | OGD\_DRUG\_DRUG.OGD\_FRUG.cls#Gs\_versus\_MCAO.OGD\_FRUG.cls#Gs\_versus\_MCAO\_repos |
| Phenotype | OGD\_FRUG.cls#Gs\_versus\_MCAO\_repos |
| Upregulated in class | MCAO |
| GeneSet | PID\_BETA\_CATENIN\_NUC\_PATHWAY |
| Enrichment Score (ES) | -0.54458946 |
| Normalized Enrichment Score (NES) | -1.5580919 |
| Nominal p-value | 0.0028208746 |
| FDR q-value | 0.07527961 |
| FWER p-Value | 0.475 |
Table: GSEA Results Summary

  

Fig 1: Enrichment plot: PID\_BETA\_CATENIN\_NUC\_PATHWAY      
 Profile of the Running ES Score & Positions of GeneSet Members on the Rank Ordered List

  

| SYMBOL | TITLE | RANK IN GENE LIST | RANK METRIC SCORE | RUNNING ES | CORE ENRICHMENT || 1 | MMP9 | na | 268 | 0.725 | 0.0310 | No |
| 2 | SFN | na | 603 | 0.554 | 0.0488 | No |
| 3 | CTNNBIP1 | na | 2373 | 0.297 | -0.0146 | No |
| 4 | HDAC2 | na | 2882 | 0.251 | -0.0229 | No |
| 5 | AES | na | 3706 | 0.186 | -0.0495 | No |
| 6 | YWHAE | na | 4295 | 0.143 | -0.0680 | No |
| 7 | YWHAB | na | 4657 | 0.117 | -0.0775 | No |
| 8 | TCF7 | na | 4825 | 0.106 | -0.0788 | No |
| 9 | TLE1 | na | 4909 | 0.100 | -0.0766 | No |
| 10 | HBP1 | na | 5211 | 0.084 | -0.0854 | No |
| 11 | MITF | na | 5358 | 0.076 | -0.0876 | No |
| 12 | MYC | na | 5587 | 0.064 | -0.0942 | No |
| 13 | MYF5 | na | 5653 | 0.060 | -0.0936 | No |
| 14 | RUVBL2 | na | 5686 | 0.059 | -0.0916 | No |
| 15 | JUN | na | 5874 | 0.048 | -0.0973 | No |
| 16 | CBY1 | na | 6974 | 0.002 | -0.1476 | No |
| 17 | DKK1 | na | 7803 | 0.000 | -0.1855 | No |
| 18 | DKK4 | na | 7805 | 0.000 | -0.1856 | No |
| 19 | AXIN2 | na | 8241 | 0.000 | -0.2055 | No |
| 20 | NEUROG1 | na | 9123 | 0.000 | -0.2460 | No |
| 21 | SP5 | na | 9939 | 0.000 | -0.2833 | No |
| 22 | CDKN2A | na | 10240 | 0.000 | -0.2971 | No |
| 23 | PITX2 | na | 10464 | 0.000 | -0.3073 | No |
| 24 | SALL4 | na | 11083 | 0.000 | -0.3357 | No |
| 25 | KRT1 | na | 11131 | 0.000 | -0.3378 | No |
| 26 | MYOG | na | 11290 | 0.000 | -0.3451 | No |
| 27 | CAMK4 | na | 11530 | 0.000 | -0.3560 | No |
| 28 | FGF4 | na | 11853 | 0.000 | -0.3708 | No |
| 29 | ZCCHC12 | na | 11878 | 0.000 | -0.3719 | No |
| 30 | CDX4 | na | 13213 | 0.000 | -0.4331 | No |
| 31 | YWHAH | na | 13393 | -0.005 | -0.4410 | No |
| 32 | KCNIP4 | na | 13467 | -0.007 | -0.4440 | No |
| 33 | AR | na | 13568 | -0.009 | -0.4480 | No |
| 34 | TLE2 | na | 13575 | -0.010 | -0.4477 | No |
| 35 | YWHAZ | na | 13785 | -0.014 | -0.4565 | No |
| 36 | ID2 | na | 14104 | -0.024 | -0.4697 | No |
| 37 | CUL1 | na | 14514 | -0.041 | -0.4860 | No |
| 38 | YWHAQ | na | 14606 | -0.046 | -0.4874 | No |
| 39 | MDFIC | na | 14762 | -0.054 | -0.4913 | No |
| 40 | CYR61 | na | 15121 | -0.075 | -0.5032 | No |
| 41 | CACNA1G | na | 15491 | -0.097 | -0.5144 | No |
| 42 | CTBP1 | na | 15701 | -0.109 | -0.5174 | No |
| 43 | SSPO | na | 15746 | -0.112 | -0.5128 | No |
| 44 | HDAC1 | na | 15789 | -0.114 | -0.5079 | No |
| 45 | TBL1XR1 | na | 16089 | -0.134 | -0.5136 | No |
| 46 | XPO1 | na | 16765 | -0.180 | -0.5338 | Yes |
| 47 | LEF1 | na | 16773 | -0.181 | -0.5233 | Yes |
| 48 | TCF7L1 | na | 16939 | -0.192 | -0.5194 | Yes |
| 49 | IGF2BP1 | na | 17355 | -0.220 | -0.5253 | Yes |
| 50 | CCND1 | na | 17421 | -0.225 | -0.5148 | Yes |
| 51 | INCENP | na | 17772 | -0.250 | -0.5160 | Yes |
| 52 | KLF4 | na | 17885 | -0.260 | -0.5055 | Yes |
| 53 | MMP2 | na | 18057 | -0.273 | -0.4971 | Yes |
| 54 | CDH1 | na | 18147 | -0.281 | -0.4844 | Yes |
| 55 | TLE4 | na | 18187 | -0.284 | -0.4692 | Yes |
| 56 | CCND2 | na | 18282 | -0.290 | -0.4562 | Yes |
| 57 | CTNNB1 | na | 18285 | -0.290 | -0.4389 | Yes |
| 58 | SNAI2 | na | 18624 | -0.313 | -0.4357 | Yes |
| 59 | TCF4 | na | 18652 | -0.317 | -0.4180 | Yes |
| 60 | CDX1 | na | 18924 | -0.342 | -0.4100 | Yes |
| 61 | YWHAG | na | 19210 | -0.370 | -0.4010 | Yes |
| 62 | SMARCA4 | na | 19320 | -0.379 | -0.3833 | Yes |
| 63 | TNIK | na | 19424 | -0.390 | -0.3647 | Yes |
| 64 | TCF7L2 | na | 19504 | -0.397 | -0.3446 | Yes |
| 65 | TBL1X | na | 19525 | -0.399 | -0.3217 | Yes |
| 66 | BCL9 | na | 19989 | -0.443 | -0.3165 | Yes |
| 67 | ADCY7 | na | 20088 | -0.453 | -0.2939 | Yes |
| 68 | MED12 | na | 20378 | -0.485 | -0.2781 | Yes |
| 69 | CHD8 | na | 20466 | -0.498 | -0.2524 | Yes |
| 70 | DVL3 | na | 20890 | -0.559 | -0.2384 | Yes |
| 71 | NCOA2 | na | 20947 | -0.569 | -0.2069 | Yes |
| 72 | TERT | na | 21189 | -0.618 | -0.1811 | Yes |
| 73 | APC | na | 21200 | -0.619 | -0.1445 | Yes |
| 74 | VCAN | na | 21332 | -0.652 | -0.1116 | Yes |
| 75 | EP300 | na | 21416 | -0.674 | -0.0751 | Yes |
| 76 | TRRAP | na | 21427 | -0.678 | -0.0351 | Yes |
| 77 | T | na | 21762 | -0.930 | 0.0052 | Yes |
Table: GSEA details [plain text format]

  

Fig 2: PID\_BETA\_CATENIN\_NUC\_PATHWAY      
 Blue-Pink O' Gram in the Space of the Analyzed GeneSet

  

Fig 3: PID\_BETA\_CATENIN\_NUC\_PATHWAY: Random ES distribution      
 Gene set null distribution of ES for **PID\_BETA\_CATENIN\_NUC\_PATHWAY**
