# Supplementary material for: Regulation of microglia related neuroinflammation contributes to the protective effect of Gelsevirine on ischemic stroke
Source: Front Immunol. 2023 Mar 30;14:1164278. doi: 10.3389/fimmu.2023.1164278 (PMC10098192; doi:10.3389/fimmu.2023.1164278)
Supplement: Supplementary file 6 [file DataSheet_6.zip › fig 5 raw/fig 5-G raw/inflammation.Gsea.1649955060129/PID_FAK_PATHWAY.html]

Details for gene set PID\_FAK\_PATHWAY[GSEA]

|  || Dataset | OGD\_DRUG\_DRUG.OGD\_FRUG.cls#Gs\_versus\_MCAO.OGD\_FRUG.cls#Gs\_versus\_MCAO\_repos |
| Phenotype | OGD\_FRUG.cls#Gs\_versus\_MCAO\_repos |
| Upregulated in class | MCAO |
| GeneSet | PID\_FAK\_PATHWAY |
| Enrichment Score (ES) | -0.6570533 |
| Normalized Enrichment Score (NES) | -1.8358039 |
| Nominal p-value | 0.0 |
| FDR q-value | 0.010101001 |
| FWER p-Value | 0.008 |
Table: GSEA Results Summary

  

Fig 1: Enrichment plot: PID\_FAK\_PATHWAY      
 Profile of the Running ES Score & Positions of GeneSet Members on the Rank Ordered List

  

| SYMBOL | TITLE | RANK IN GENE LIST | RANK METRIC SCORE | RUNNING ES | CORE ENRICHMENT || 1 | RHOA | na | 1999 | 0.338 | -0.0711 | No |
| 2 | RAP1A | na | 2972 | 0.244 | -0.1009 | No |
| 3 | PAK1 | na | 3623 | 0.191 | -0.1191 | No |
| 4 | RAC1 | na | 3821 | 0.178 | -0.1173 | No |
| 5 | GRB2 | na | 5726 | 0.057 | -0.2012 | No |
| 6 | JUN | na | 5874 | 0.048 | -0.2050 | No |
| 7 | SH3GL1 | na | 6292 | 0.029 | -0.2223 | No |
| 8 | MAPK9 | na | 6752 | 0.011 | -0.2427 | No |
| 9 | NCK1 | na | 6802 | 0.009 | -0.2444 | No |
| 10 | FYN | na | 6884 | 0.006 | -0.2477 | No |
| 11 | RRAS | na | 6958 | 0.003 | -0.2509 | No |
| 12 | RAP1B | na | 7005 | 0.001 | -0.2530 | No |
| 13 | KLF8 | na | 9767 | 0.000 | -0.3795 | No |
| 14 | BMX | na | 13255 | -0.000 | -0.5393 | No |
| 15 | ITGB5 | na | 13587 | -0.010 | -0.5539 | No |
| 16 | ACTA1 | na | 13930 | -0.018 | -0.5685 | No |
| 17 | MAP2K1 | na | 15179 | -0.079 | -0.6209 | No |
| 18 | MMP14 | na | 15498 | -0.097 | -0.6296 | No |
| 19 | ARHGAP26 | na | 15568 | -0.102 | -0.6266 | No |
| 20 | MAPK1 | na | 16234 | -0.143 | -0.6484 | Yes |
| 21 | ELMO1 | na | 16276 | -0.145 | -0.6414 | Yes |
| 22 | CAPN2 | na | 16345 | -0.151 | -0.6354 | Yes |
| 23 | YES1 | na | 16373 | -0.153 | -0.6274 | Yes |
| 24 | PTK2 | na | 16640 | -0.173 | -0.6291 | Yes |
| 25 | ITGB1 | na | 16661 | -0.174 | -0.6195 | Yes |
| 26 | CRK | na | 16856 | -0.186 | -0.6171 | Yes |
| 27 | MAP2K4 | na | 16904 | -0.189 | -0.6078 | Yes |
| 28 | GRB7 | na | 17152 | -0.206 | -0.6066 | Yes |
| 29 | RAF1 | na | 17251 | -0.212 | -0.5982 | Yes |
| 30 | ITGA5 | na | 17393 | -0.223 | -0.5912 | Yes |
| 31 | CCND1 | na | 17421 | -0.225 | -0.5788 | Yes |
| 32 | WASL | na | 17717 | -0.246 | -0.5774 | Yes |
| 33 | BCAR1 | na | 17804 | -0.253 | -0.5660 | Yes |
| 34 | MAPK8 | na | 17866 | -0.258 | -0.5531 | Yes |
| 35 | PIK3CA | na | 18513 | -0.305 | -0.5642 | Yes |
| 36 | RASA1 | na | 18622 | -0.313 | -0.5502 | Yes |
| 37 | ARHGEF7 | na | 18968 | -0.346 | -0.5450 | Yes |
| 38 | MAPK8IP3 | na | 18997 | -0.348 | -0.5252 | Yes |
| 39 | PXN | na | 19391 | -0.386 | -0.5198 | Yes |
| 40 | ETS1 | na | 19731 | -0.420 | -0.5098 | Yes |
| 41 | GIT2 | na | 20137 | -0.460 | -0.5005 | Yes |
| 42 | SRC | na | 20255 | -0.472 | -0.4772 | Yes |
| 43 | NCK2 | na | 20375 | -0.485 | -0.4533 | Yes |
| 44 | RAPGEF1 | na | 20440 | -0.495 | -0.4262 | Yes |
| 45 | DOCK1 | na | 20523 | -0.505 | -0.3993 | Yes |
| 46 | SOS1 | na | 20563 | -0.511 | -0.3702 | Yes |
| 47 | ACTN1 | na | 20635 | -0.521 | -0.3419 | Yes |
| 48 | ROCK2 | na | 20637 | -0.521 | -0.3103 | Yes |
| 49 | BRAF | na | 20807 | -0.547 | -0.2849 | Yes |
| 50 | PTPN21 | na | 20831 | -0.551 | -0.2526 | Yes |
| 51 | ASAP1 | na | 20873 | -0.557 | -0.2207 | Yes |
| 52 | VCL | na | 20929 | -0.565 | -0.1889 | Yes |
| 53 | ARHGAP35 | na | 20999 | -0.579 | -0.1570 | Yes |
| 54 | ITGAV | na | 21226 | -0.625 | -0.1295 | Yes |
| 55 | PLCG1 | na | 21230 | -0.627 | -0.0915 | Yes |
| 56 | TLN1 | na | 21253 | -0.632 | -0.0542 | Yes |
| 57 | PIK3R1 | na | 21419 | -0.674 | -0.0209 | Yes |
| 58 | ARHGEF11 | na | 21458 | -0.689 | 0.0191 | Yes |
Table: GSEA details [plain text format]

  

Fig 2: PID\_FAK\_PATHWAY      
 Blue-Pink O' Gram in the Space of the Analyzed GeneSet

  

Fig 3: PID\_FAK\_PATHWAY: Random ES distribution      
 Gene set null distribution of ES for **PID\_FAK\_PATHWAY**
