# Supplementary material for: Regulation of microglia related neuroinflammation contributes to the protective effect of Gelsevirine on ischemic stroke
Source: Front Immunol. 2023 Mar 30;14:1164278. doi: 10.3389/fimmu.2023.1164278 (PMC10098192; doi:10.3389/fimmu.2023.1164278)
Supplement: Supplementary file 6 [file DataSheet_6.zip › fig 5 raw/fig 5-G raw/inflammation.Gsea.1649955060129/PID_IGF1_PATHWAY.html]

Details for gene set PID\_IGF1\_PATHWAY[GSEA]

|  || Dataset | OGD\_DRUG\_DRUG.OGD\_FRUG.cls#Gs\_versus\_MCAO.OGD\_FRUG.cls#Gs\_versus\_MCAO\_repos |
| Phenotype | OGD\_FRUG.cls#Gs\_versus\_MCAO\_repos |
| Upregulated in class | MCAO |
| GeneSet | PID\_IGF1\_PATHWAY |
| Enrichment Score (ES) | -0.6668536 |
| Normalized Enrichment Score (NES) | -1.6521701 |
| Nominal p-value | 0.00792393 |
| FDR q-value | 0.065362394 |
| FWER p-Value | 0.184 |
Table: GSEA Results Summary

  

Fig 1: Enrichment plot: PID\_IGF1\_PATHWAY      
 Profile of the Running ES Score & Positions of GeneSet Members on the Rank Ordered List

  

| SYMBOL | TITLE | RANK IN GENE LIST | RANK METRIC SCORE | RUNNING ES | CORE ENRICHMENT || 1 | HRAS | na | 2716 | 0.268 | -0.0940 | No |
| 2 | BAD | na | 3658 | 0.189 | -0.1158 | No |
| 3 | YWHAE | na | 4295 | 0.143 | -0.1288 | No |
| 4 | GRB2 | na | 5726 | 0.057 | -0.1879 | No |
| 5 | PRKCZ | na | 6115 | 0.036 | -0.2016 | No |
| 6 | YWHAZ | na | 13785 | -0.014 | -0.5511 | No |
| 7 | PRKCD | na | 16045 | -0.131 | -0.6397 | No |
| 8 | PTK2 | na | 16640 | -0.173 | -0.6474 | Yes |
| 9 | AKT1 | na | 16827 | -0.184 | -0.6351 | Yes |
| 10 | CRK | na | 16856 | -0.186 | -0.6154 | Yes |
| 11 | PTPN1 | na | 17059 | -0.199 | -0.6021 | Yes |
| 12 | RAF1 | na | 17251 | -0.212 | -0.5868 | Yes |
| 13 | PRKD1 | na | 17256 | -0.213 | -0.5630 | Yes |
| 14 | RPS6KB1 | na | 17275 | -0.214 | -0.5396 | Yes |
| 15 | IGF1 | na | 17740 | -0.247 | -0.5329 | Yes |
| 16 | BCAR1 | na | 17804 | -0.253 | -0.5072 | Yes |
| 17 | PIK3CA | na | 18513 | -0.305 | -0.5052 | Yes |
| 18 | SHC1 | na | 19109 | -0.361 | -0.4917 | Yes |
| 19 | GRB10 | na | 19381 | -0.385 | -0.4607 | Yes |
| 20 | PXN | na | 19391 | -0.386 | -0.4174 | Yes |
| 21 | CRKL | na | 19431 | -0.390 | -0.3751 | Yes |
| 22 | PTPN11 | na | 19508 | -0.397 | -0.3338 | Yes |
| 23 | PDPK1 | na | 20100 | -0.455 | -0.3094 | Yes |
| 24 | NCK2 | na | 20375 | -0.485 | -0.2672 | Yes |
| 25 | SOS1 | na | 20563 | -0.511 | -0.2181 | Yes |
| 26 | IRS1 | na | 20589 | -0.515 | -0.1611 | Yes |
| 27 | IGF1R | na | 21187 | -0.617 | -0.1188 | Yes |
| 28 | IRS2 | na | 21348 | -0.656 | -0.0521 | Yes |
| 29 | PIK3R1 | na | 21419 | -0.674 | 0.0209 | Yes |
Table: GSEA details [plain text format]

  

Fig 2: PID\_IGF1\_PATHWAY      
 Blue-Pink O' Gram in the Space of the Analyzed GeneSet

  

Fig 3: PID\_IGF1\_PATHWAY: Random ES distribution      
 Gene set null distribution of ES for **PID\_IGF1\_PATHWAY**
