# Supplementary material for: Regulation of microglia related neuroinflammation contributes to the protective effect of Gelsevirine on ischemic stroke
Source: Front Immunol. 2023 Mar 30;14:1164278. doi: 10.3389/fimmu.2023.1164278 (PMC10098192; doi:10.3389/fimmu.2023.1164278)
Supplement: Supplementary file 6 [file DataSheet_6.zip › fig 5 raw/fig 5-G raw/inflammation.Gsea.1649955060129/PID_RHOA_REG_PATHWAY.html]

Details for gene set PID\_RHOA\_REG\_PATHWAY[GSEA]

|  || Dataset | OGD\_DRUG\_DRUG.OGD\_FRUG.cls#Gs\_versus\_MCAO.OGD\_FRUG.cls#Gs\_versus\_MCAO\_repos |
| Phenotype | OGD\_FRUG.cls#Gs\_versus\_MCAO\_repos |
| Upregulated in class | MCAO |
| GeneSet | PID\_RHOA\_REG\_PATHWAY |
| Enrichment Score (ES) | -0.6007981 |
| Normalized Enrichment Score (NES) | -1.5986708 |
| Nominal p-value | 0.0029895366 |
| FDR q-value | 0.06555429 |
| FWER p-Value | 0.343 |
Table: GSEA Results Summary

  

Fig 1: Enrichment plot: PID\_RHOA\_REG\_PATHWAY      
 Profile of the Running ES Score & Positions of GeneSet Members on the Rank Ordered List

  

| SYMBOL | TITLE | RANK IN GENE LIST | RANK METRIC SCORE | RUNNING ES | CORE ENRICHMENT || 1 | VAV3 | na | 1228 | 0.431 | -0.0280 | No |
| 2 | ARHGEF5 | na | 1760 | 0.367 | -0.0282 | No |
| 3 | OBSCN | na | 1909 | 0.348 | -0.0122 | No |
| 4 | RHOA | na | 1999 | 0.338 | 0.0059 | No |
| 5 | ARHGEF3 | na | 2715 | 0.268 | -0.0092 | No |
| 6 | ARHGDIB | na | 2850 | 0.255 | 0.0013 | No |
| 7 | ARHGEF25 | na | 4342 | 0.140 | -0.0578 | No |
| 8 | NGEF | na | 4492 | 0.129 | -0.0561 | No |
| 9 | DEF6 | na | 4629 | 0.120 | -0.0545 | No |
| 10 | ARHGAP8 | na | 5599 | 0.063 | -0.0948 | No |
| 11 | CDKN1B | na | 5942 | 0.045 | -0.1075 | No |
| 12 | ARHGEF15 | na | 6138 | 0.035 | -0.1141 | No |
| 13 | VAV1 | na | 8205 | 0.000 | -0.2088 | No |
| 14 | ARHGDIA | na | 13269 | -0.001 | -0.4406 | No |
| 15 | ARHGAP6 | na | 13819 | -0.015 | -0.4648 | No |
| 16 | ARHGAP9 | na | 14568 | -0.044 | -0.4962 | No |
| 17 | OPHN1 | na | 16014 | -0.129 | -0.5539 | No |
| 18 | PLEKHG6 | na | 16026 | -0.130 | -0.5459 | No |
| 19 | ARHGEF18 | na | 17100 | -0.202 | -0.5817 | No |
| 20 | ARHGEF2 | na | 17184 | -0.208 | -0.5719 | No |
| 21 | ARHGEF10L | na | 17816 | -0.255 | -0.5841 | Yes |
| 22 | ARHGAP4 | na | 17860 | -0.258 | -0.5692 | Yes |
| 23 | ARHGDIG | na | 17875 | -0.259 | -0.5528 | Yes |
| 24 | MCF2L | na | 18046 | -0.272 | -0.5427 | Yes |
| 25 | NET1 | na | 18052 | -0.273 | -0.5251 | Yes |
| 26 | ARAP1 | na | 18247 | -0.289 | -0.5150 | Yes |
| 27 | ARAP3 | na | 18479 | -0.302 | -0.5058 | Yes |
| 28 | ARHGEF1 | na | 18815 | -0.331 | -0.4994 | Yes |
| 29 | SRGAP1 | na | 19204 | -0.370 | -0.4929 | Yes |
| 30 | ECT2 | na | 19250 | -0.373 | -0.4705 | Yes |
| 31 | DLC1 | na | 19334 | -0.380 | -0.4494 | Yes |
| 32 | VAV2 | na | 19992 | -0.443 | -0.4504 | Yes |
| 33 | ARHGAP5 | na | 20516 | -0.503 | -0.4413 | Yes |
| 34 | ARHGEF10 | na | 20701 | -0.529 | -0.4150 | Yes |
| 35 | ABR | na | 20756 | -0.538 | -0.3822 | Yes |
| 36 | ARHGAP35 | na | 20999 | -0.579 | -0.3553 | Yes |
| 37 | BCR | na | 21121 | -0.601 | -0.3214 | Yes |
| 38 | MYO9B | na | 21199 | -0.619 | -0.2843 | Yes |
| 39 | MCF2 | na | 21283 | -0.639 | -0.2462 | Yes |
| 40 | ARHGEF12 | na | 21366 | -0.658 | -0.2068 | Yes |
| 41 | FARP1 | na | 21381 | -0.662 | -0.1640 | Yes |
| 42 | ARHGEF17 | na | 21440 | -0.683 | -0.1218 | Yes |
| 43 | TRIO | na | 21447 | -0.686 | -0.0771 | Yes |
| 44 | ARHGEF11 | na | 21458 | -0.689 | -0.0324 | Yes |
| 45 | AKAP13 | na | 21642 | -0.784 | 0.0107 | Yes |
Table: GSEA details [plain text format]

  

Fig 2: PID\_RHOA\_REG\_PATHWAY      
 Blue-Pink O' Gram in the Space of the Analyzed GeneSet

  

Fig 3: PID\_RHOA\_REG\_PATHWAY: Random ES distribution      
 Gene set null distribution of ES for **PID\_RHOA\_REG\_PATHWAY**
