# Supplementary material for: Regulation of microglia related neuroinflammation contributes to the protective effect of Gelsevirine on ischemic stroke
Source: Front Immunol. 2023 Mar 30;14:1164278. doi: 10.3389/fimmu.2023.1164278 (PMC10098192; doi:10.3389/fimmu.2023.1164278)
Supplement: Supplementary file 6 [file DataSheet_6.zip › fig 5 raw/fig 5-G raw/inflammation.Gsea.1649955060129/PID_SHP2_PATHWAY.html]

Details for gene set PID\_SHP2\_PATHWAY[GSEA]

|  || Dataset | OGD\_DRUG\_DRUG.OGD\_FRUG.cls#Gs\_versus\_MCAO.OGD\_FRUG.cls#Gs\_versus\_MCAO\_repos |
| Phenotype | OGD\_FRUG.cls#Gs\_versus\_MCAO\_repos |
| Upregulated in class | MCAO |
| GeneSet | PID\_SHP2\_PATHWAY |
| Enrichment Score (ES) | -0.59106517 |
| Normalized Enrichment Score (NES) | -1.6188679 |
| Nominal p-value | 0.0 |
| FDR q-value | 0.05998584 |
| FWER p-Value | 0.278 |
Table: GSEA Results Summary

  

Fig 1: Enrichment plot: PID\_SHP2\_PATHWAY      
 Profile of the Running ES Score & Positions of GeneSet Members on the Rank Ordered List

  

| SYMBOL | TITLE | RANK IN GENE LIST | RANK METRIC SCORE | RUNNING ES | CORE ENRICHMENT || 1 | IL2RB | na | 822 | 0.494 | -0.0045 | No |
| 2 | LMO4 | na | 1632 | 0.382 | -0.0158 | No |
| 3 | RHOA | na | 1999 | 0.338 | -0.0099 | No |
| 4 | HRAS | na | 2716 | 0.268 | -0.0247 | No |
| 5 | NTF3 | na | 3063 | 0.236 | -0.0247 | No |
| 6 | GNAI1 | na | 4304 | 0.143 | -0.0720 | No |
| 7 | NRAS | na | 4669 | 0.117 | -0.0808 | No |
| 8 | MAP2K2 | na | 4692 | 0.115 | -0.0740 | No |
| 9 | VEGFA | na | 5039 | 0.094 | -0.0836 | No |
| 10 | IL2 | na | 5219 | 0.084 | -0.0862 | No |
| 11 | LCK | na | 5511 | 0.068 | -0.0950 | No |
| 12 | GRB2 | na | 5726 | 0.057 | -0.1010 | No |
| 13 | NTRK1 | na | 6192 | 0.033 | -0.1201 | No |
| 14 | KRAS | na | 6543 | 0.019 | -0.1349 | No |
| 15 | SDC2 | na | 6810 | 0.009 | -0.1465 | No |
| 16 | NTRK2 | na | 8058 | 0.000 | -0.2036 | No |
| 17 | NTRK3 | na | 8060 | 0.000 | -0.2037 | No |
| 18 | NOS3 | na | 8810 | 0.000 | -0.2380 | No |
| 19 | IFNG | na | 9828 | 0.000 | -0.2846 | No |
| 20 | ANGPT1 | na | 11116 | 0.000 | -0.3436 | No |
| 21 | NGF | na | 13604 | -0.010 | -0.4569 | No |
| 22 | KDR | na | 13617 | -0.011 | -0.4567 | No |
| 23 | IL2RA | na | 14028 | -0.021 | -0.4741 | No |
| 24 | IFNGR1 | na | 14409 | -0.036 | -0.4891 | No |
| 25 | JAK2 | na | 15175 | -0.079 | -0.5188 | No |
| 26 | MAP2K1 | na | 15179 | -0.079 | -0.5137 | No |
| 27 | PRKACA | na | 15891 | -0.121 | -0.5381 | No |
| 28 | BDNF | na | 16156 | -0.138 | -0.5409 | No |
| 29 | RAF1 | na | 17251 | -0.212 | -0.5768 | Yes |
| 30 | GNAI3 | na | 17440 | -0.226 | -0.5702 | Yes |
| 31 | PDGFB | na | 17563 | -0.234 | -0.5601 | Yes |
| 32 | IGF1 | na | 17740 | -0.247 | -0.5515 | Yes |
| 33 | STAT1 | na | 17788 | -0.252 | -0.5367 | Yes |
| 34 | TEK | na | 17891 | -0.261 | -0.5239 | Yes |
| 35 | PIK3CA | na | 18513 | -0.305 | -0.5318 | Yes |
| 36 | IL2RG | na | 18663 | -0.317 | -0.5173 | Yes |
| 37 | EGFR | na | 19043 | -0.354 | -0.5109 | Yes |
| 38 | SHC1 | na | 19109 | -0.361 | -0.4897 | Yes |
| 39 | IL6 | na | 19212 | -0.371 | -0.4694 | Yes |
| 40 | GAB2 | na | 19315 | -0.379 | -0.4486 | Yes |
| 41 | PTPN11 | na | 19508 | -0.397 | -0.4308 | Yes |
| 42 | GAB1 | na | 19622 | -0.409 | -0.4084 | Yes |
| 43 | JAK1 | na | 20014 | -0.446 | -0.3964 | Yes |
| 44 | JAK3 | na | 20139 | -0.460 | -0.3711 | Yes |
| 45 | FRS2 | na | 20145 | -0.461 | -0.3404 | Yes |
| 46 | EGF | na | 20244 | -0.471 | -0.3132 | Yes |
| 47 | SOS1 | na | 20563 | -0.511 | -0.2935 | Yes |
| 48 | IRS1 | na | 20589 | -0.515 | -0.2600 | Yes |
| 49 | FRS3 | na | 20667 | -0.524 | -0.2283 | Yes |
| 50 | IL6ST | na | 20881 | -0.558 | -0.2006 | Yes |
| 51 | PDGFRB | na | 20968 | -0.572 | -0.1661 | Yes |
| 52 | ARHGAP35 | na | 20999 | -0.579 | -0.1285 | Yes |
| 53 | MLLT4 | na | 21105 | -0.599 | -0.0931 | Yes |
| 54 | PAG1 | na | 21184 | -0.616 | -0.0552 | Yes |
| 55 | IGF1R | na | 21187 | -0.617 | -0.0138 | Yes |
| 56 | PIK3R1 | na | 21419 | -0.674 | 0.0209 | Yes |
Table: GSEA details [plain text format]

  

Fig 2: PID\_SHP2\_PATHWAY      
 Blue-Pink O' Gram in the Space of the Analyzed GeneSet

  

Fig 3: PID\_SHP2\_PATHWAY: Random ES distribution      
 Gene set null distribution of ES for **PID\_SHP2\_PATHWAY**
