# Supplementary material for: Regulation of microglia related neuroinflammation contributes to the protective effect of Gelsevirine on ischemic stroke
Source: Front Immunol. 2023 Mar 30;14:1164278. doi: 10.3389/fimmu.2023.1164278 (PMC10098192; doi:10.3389/fimmu.2023.1164278)
Supplement: Supplementary file 6 [file DataSheet_6.zip › fig 5 raw/fig 5-G raw/inflammation.Gsea.1649955060129/PID_WNT_CANONICAL_PATHWAY.html]

Details for gene set PID\_WNT\_CANONICAL\_PATHWAY[GSEA]

|  || Dataset | OGD\_DRUG\_DRUG.OGD\_FRUG.cls#Gs\_versus\_MCAO.OGD\_FRUG.cls#Gs\_versus\_MCAO\_repos |
| Phenotype | OGD\_FRUG.cls#Gs\_versus\_MCAO\_repos |
| Upregulated in class | MCAO |
| GeneSet | PID\_WNT\_CANONICAL\_PATHWAY |
| Enrichment Score (ES) | -0.7230293 |
| Normalized Enrichment Score (NES) | -1.65366 |
| Nominal p-value | 0.003220612 |
| FDR q-value | 0.08547171 |
| FWER p-Value | 0.182 |
Table: GSEA Results Summary

  

Fig 1: Enrichment plot: PID\_WNT\_CANONICAL\_PATHWAY      
 Profile of the Running ES Score & Positions of GeneSet Members on the Rank Ordered List

  

| SYMBOL | TITLE | RANK IN GENE LIST | RANK METRIC SCORE | RUNNING ES | CORE ENRICHMENT || 1 | WNT3A | na | 6232 | 0.031 | -0.2787 | No |
| 2 | CUL3 | na | 6751 | 0.011 | -0.3002 | No |
| 3 | NKD2 | na | 8212 | 0.000 | -0.3670 | No |
| 4 | PIP5K1B | na | 10813 | 0.000 | -0.4859 | No |
| 5 | PI4K2A | na | 13264 | -0.000 | -0.5980 | No |
| 6 | PPP2R5A | na | 13335 | -0.002 | -0.6007 | No |
| 7 | RANBP3 | na | 13427 | -0.006 | -0.6036 | No |
| 8 | GSK3A | na | 14248 | -0.030 | -0.6350 | No |
| 9 | CAV1 | na | 16173 | -0.139 | -0.6943 | Yes |
| 10 | KLHL12 | na | 16524 | -0.163 | -0.6766 | Yes |
| 11 | DVL2 | na | 17478 | -0.229 | -0.6730 | Yes |
| 12 | DVL1 | na | 17888 | -0.261 | -0.6380 | Yes |
| 13 | AXIN1 | na | 18098 | -0.276 | -0.5905 | Yes |
| 14 | CTNNB1 | na | 18285 | -0.290 | -0.5391 | Yes |
| 15 | GSK3B | na | 20096 | -0.455 | -0.5279 | Yes |
| 16 | CSNK1G1 | na | 20528 | -0.506 | -0.4432 | Yes |
| 17 | DVL3 | na | 20890 | -0.559 | -0.3443 | Yes |
| 18 | APC | na | 21200 | -0.619 | -0.2306 | Yes |
| 19 | FZD5 | na | 21229 | -0.627 | -0.1025 | Yes |
| 20 | LRP6 | na | 21288 | -0.639 | 0.0269 | Yes |
Table: GSEA details [plain text format]

  

Fig 2: PID\_WNT\_CANONICAL\_PATHWAY      
 Blue-Pink O' Gram in the Space of the Analyzed GeneSet

  

Fig 3: PID\_WNT\_CANONICAL\_PATHWAY: Random ES distribution      
 Gene set null distribution of ES for **PID\_WNT\_CANONICAL\_PATHWAY**
