# Supplementary material for: Regulation of microglia related neuroinflammation contributes to the protective effect of Gelsevirine on ischemic stroke
Source: Front Immunol. 2023 Mar 30;14:1164278. doi: 10.3389/fimmu.2023.1164278 (PMC10098192; doi:10.3389/fimmu.2023.1164278)
Supplement: Supplementary file 6 [file DataSheet_6.zip › fig 5 raw/fig 5-G raw/inflammation.Gsea.1649955060129/REACTOME_SIGNALING_BY_HIPPO.html]

Details for gene set REACTOME\_SIGNALING\_BY\_HIPPO[GSEA]

|  || Dataset | OGD\_DRUG\_DRUG.OGD\_FRUG.cls#Gs\_versus\_MCAO.OGD\_FRUG.cls#Gs\_versus\_MCAO\_repos |
| Phenotype | OGD\_FRUG.cls#Gs\_versus\_MCAO\_repos |
| Upregulated in class | MCAO |
| GeneSet | REACTOME\_SIGNALING\_BY\_HIPPO |
| Enrichment Score (ES) | -0.7158608 |
| Normalized Enrichment Score (NES) | -1.6341043 |
| Nominal p-value | 0.0080256825 |
| FDR q-value | 0.054112438 |
| FWER p-Value | 0.227 |
Table: GSEA Results Summary

  

Fig 1: Enrichment plot: REACTOME\_SIGNALING\_BY\_HIPPO      
 Profile of the Running ES Score & Positions of GeneSet Members on the Rank Ordered List

  

| SYMBOL | TITLE | RANK IN GENE LIST | RANK METRIC SCORE | RUNNING ES | CORE ENRICHMENT || 1 | YWHAE | na | 4295 | 0.143 | -0.1785 | No |
| 2 | YWHAB | na | 4657 | 0.117 | -0.1802 | No |
| 3 | CASP3 | na | 5009 | 0.095 | -0.1842 | No |
| 4 | SAV1 | na | 15899 | -0.122 | -0.6670 | No |
| 5 | WWTR1 | na | 16841 | -0.185 | -0.6868 | No |
| 6 | DVL2 | na | 17478 | -0.229 | -0.6870 | Yes |
| 7 | STK3 | na | 17781 | -0.251 | -0.6691 | Yes |
| 8 | YAP1 | na | 17920 | -0.263 | -0.6422 | Yes |
| 9 | STK4 | na | 19284 | -0.376 | -0.6570 | Yes |
| 10 | MOB1B | na | 19724 | -0.419 | -0.6242 | Yes |
| 11 | MOB1A | na | 20091 | -0.454 | -0.5836 | Yes |
| 12 | WWC1 | na | 20415 | -0.491 | -0.5364 | Yes |
| 13 | LATS1 | na | 20439 | -0.495 | -0.4750 | Yes |
| 14 | AMOTL2 | na | 20470 | -0.499 | -0.4133 | Yes |
| 15 | TJP1 | na | 20615 | -0.518 | -0.3545 | Yes |
| 16 | AMOT | na | 20654 | -0.523 | -0.2902 | Yes |
| 17 | AMOTL1 | na | 20860 | -0.555 | -0.2295 | Yes |
| 18 | TJP2 | na | 20920 | -0.564 | -0.1610 | Yes |
| 19 | LATS2 | na | 21104 | -0.599 | -0.0937 | Yes |
| 20 | NPHP4 | na | 21790 | -1.021 | 0.0039 | Yes |
Table: GSEA details [plain text format]

  

Fig 2: REACTOME\_SIGNALING\_BY\_HIPPO      
 Blue-Pink O' Gram in the Space of the Analyzed GeneSet

  

Fig 3: REACTOME\_SIGNALING\_BY\_HIPPO: Random ES distribution      
 Gene set null distribution of ES for **REACTOME\_SIGNALING\_BY\_HIPPO**
