# Supplementary material for: Regulation of microglia related neuroinflammation contributes to the protective effect of Gelsevirine on ischemic stroke
Source: Front Immunol. 2023 Mar 30;14:1164278. doi: 10.3389/fimmu.2023.1164278 (PMC10098192; doi:10.3389/fimmu.2023.1164278)
Supplement: Supplementary file 6 [file DataSheet_6.zip › fig 5 raw/fig 5-G raw/inflammation.Gsea.1649955060129/REACTOME_SIGNALING_BY_WNT.html]

Details for gene set REACTOME\_SIGNALING\_BY\_WNT[GSEA]

|  || Dataset | OGD\_DRUG\_DRUG.OGD\_FRUG.cls#Gs\_versus\_MCAO.OGD\_FRUG.cls#Gs\_versus\_MCAO\_repos |
| Phenotype | OGD\_FRUG.cls#Gs\_versus\_MCAO\_repos |
| Upregulated in class | Gs |
| GeneSet | REACTOME\_SIGNALING\_BY\_WNT |
| Enrichment Score (ES) | 0.6069026 |
| Normalized Enrichment Score (NES) | 1.8520643 |
| Nominal p-value | 0.0 |
| FDR q-value | 0.005841903 |
| FWER p-Value | 0.026 |
Table: GSEA Results Summary

  

Fig 1: Enrichment plot: REACTOME\_SIGNALING\_BY\_WNT      
 Profile of the Running ES Score & Positions of GeneSet Members on the Rank Ordered List

  

| SYMBOL | TITLE | RANK IN GENE LIST | RANK METRIC SCORE | RUNNING ES | CORE ENRICHMENT || 1 | PSMB8 | na | 30 | 1.397 | 0.0709 | Yes |
| 2 | RPS27A | na | 273 | 0.718 | 0.0970 | Yes |
| 3 | PSMB7 | na | 435 | 0.620 | 0.1217 | Yes |
| 4 | PSMB4 | na | 539 | 0.572 | 0.1466 | Yes |
| 5 | PSMA6 | na | 735 | 0.516 | 0.1644 | Yes |
| 6 | PSMA5 | na | 768 | 0.507 | 0.1891 | Yes |
| 7 | PSMB3 | na | 861 | 0.484 | 0.2099 | Yes |
| 8 | PSMD10 | na | 1137 | 0.444 | 0.2203 | Yes |
| 9 | PSMB1 | na | 1208 | 0.433 | 0.2395 | Yes |
| 10 | PSMA3 | na | 1229 | 0.431 | 0.2609 | Yes |
| 11 | PSMB6 | na | 1252 | 0.428 | 0.2820 | Yes |
| 12 | PSMA7 | na | 1331 | 0.417 | 0.3000 | Yes |
| 13 | PSMA4 | na | 1348 | 0.415 | 0.3207 | Yes |
| 14 | UBA52 | na | 1408 | 0.410 | 0.3393 | Yes |
| 15 | PSMA2 | na | 1522 | 0.398 | 0.3546 | Yes |
| 16 | PSMD4 | na | 1600 | 0.387 | 0.3711 | Yes |
| 17 | PSMC6 | na | 1625 | 0.383 | 0.3899 | Yes |
| 18 | PSMD6 | na | 1653 | 0.379 | 0.4083 | Yes |
| 19 | PSMB2 | na | 1712 | 0.373 | 0.4249 | Yes |
| 20 | PSMD14 | na | 1739 | 0.369 | 0.4428 | Yes |
| 21 | PSMA1 | na | 1854 | 0.356 | 0.4560 | Yes |
| 22 | PSME2 | na | 1953 | 0.343 | 0.4692 | Yes |
| 23 | PSME1 | na | 2090 | 0.329 | 0.4800 | Yes |
| 24 | PSMC2 | na | 2133 | 0.323 | 0.4948 | Yes |
| 25 | PSMD9 | na | 2148 | 0.321 | 0.5108 | Yes |
| 26 | PSMD7 | na | 2378 | 0.297 | 0.5157 | Yes |
| 27 | PSMC5 | na | 2572 | 0.281 | 0.5213 | Yes |
| 28 | PSMB5 | na | 2587 | 0.279 | 0.5351 | Yes |
| 29 | PSMC4 | na | 2617 | 0.277 | 0.5482 | Yes |
| 30 | PSMD13 | na | 2804 | 0.259 | 0.5531 | Yes |
| 31 | PSMD12 | na | 2816 | 0.258 | 0.5659 | Yes |
| 32 | PSMC3 | na | 2821 | 0.257 | 0.5791 | Yes |
| 33 | PSMB10 | na | 2826 | 0.257 | 0.5922 | Yes |
| 34 | PSMD8 | na | 2992 | 0.242 | 0.5971 | Yes |
| 35 | FRAT2 | na | 3192 | 0.224 | 0.5996 | Yes |
| 36 | PSMB9 | na | 3291 | 0.215 | 0.6063 | Yes |
| 37 | PSMC1 | na | 3503 | 0.199 | 0.6069 | Yes |
| 38 | PSMD11 | na | 4094 | 0.156 | 0.5879 | No |
| 39 | PPP2CB | na | 4186 | 0.150 | 0.5915 | No |
| 40 | PPP2R5C | na | 4684 | 0.116 | 0.5747 | No |
| 41 | PSMD1 | na | 5203 | 0.085 | 0.5554 | No |
| 42 | PSMD5 | na | 5729 | 0.056 | 0.5342 | No |
| 43 | PPP2CA | na | 6335 | 0.027 | 0.5079 | No |
| 44 | PSMA8 | na | 9496 | 0.000 | 0.3630 | No |
| 45 | PSMD3 | na | 13262 | -0.000 | 0.1905 | No |
| 46 | PPP2R5A | na | 13335 | -0.002 | 0.1873 | No |
| 47 | PSMD2 | na | 13368 | -0.003 | 0.1860 | No |
| 48 | PPP2R1A | na | 14223 | -0.029 | 0.1484 | No |
| 49 | BTRC | na | 14297 | -0.031 | 0.1466 | No |
| 50 | CUL1 | na | 14514 | -0.041 | 0.1389 | No |
| 51 | PSMF1 | na | 15617 | -0.104 | 0.0938 | No |
| 52 | CSNK1A1 | na | 16678 | -0.175 | 0.0542 | No |
| 53 | PPP2R5E | na | 17191 | -0.208 | 0.0415 | No |
| 54 | PPP2R5D | na | 17677 | -0.242 | 0.0319 | No |
| 55 | PPP2R1B | na | 18017 | -0.271 | 0.0303 | No |
| 56 | AXIN1 | na | 18098 | -0.276 | 0.0409 | No |
| 57 | CTNNB1 | na | 18285 | -0.290 | 0.0474 | No |
| 58 | PSME4 | na | 19389 | -0.386 | 0.0169 | No |
| 59 | PPP2R5B | na | 20476 | -0.499 | -0.0071 | No |
| 60 | APC | na | 21200 | -0.619 | -0.0082 | No |
| 61 | FRAT1 | na | 21598 | -0.755 | 0.0127 | No |
Table: GSEA details [plain text format]

  

Fig 2: REACTOME\_SIGNALING\_BY\_WNT      
 Blue-Pink O' Gram in the Space of the Analyzed GeneSet

  

Fig 3: REACTOME\_SIGNALING\_BY\_WNT: Random ES distribution      
 Gene set null distribution of ES for **REACTOME\_SIGNALING\_BY\_WNT**
