# Supplementary material for: Regulation of microglia related neuroinflammation contributes to the protective effect of Gelsevirine on ischemic stroke
Source: Front Immunol. 2023 Mar 30;14:1164278. doi: 10.3389/fimmu.2023.1164278 (PMC10098192; doi:10.3389/fimmu.2023.1164278)
Supplement: Supplementary file 6 [file DataSheet_6.zip › fig 5 raw/fig 5-G raw/inflammation.Gsea.1649955060129/SCHURINGA_STAT5A_TARGETS_DN.html]

Details for gene set SCHURINGA\_STAT5A\_TARGETS\_DN[GSEA]

|  || Dataset | OGD\_DRUG\_DRUG.OGD\_FRUG.cls#Gs\_versus\_MCAO.OGD\_FRUG.cls#Gs\_versus\_MCAO\_repos |
| Phenotype | OGD\_FRUG.cls#Gs\_versus\_MCAO\_repos |
| Upregulated in class | Gs |
| GeneSet | SCHURINGA\_STAT5A\_TARGETS\_DN |
| Enrichment Score (ES) | 0.5983096 |
| Normalized Enrichment Score (NES) | 1.3317442 |
| Nominal p-value | 0.12068965 |
| FDR q-value | 0.18746236 |
| FWER p-Value | 0.946 |
Table: GSEA Results Summary

  

Fig 1: Enrichment plot: SCHURINGA\_STAT5A\_TARGETS\_DN      
 Profile of the Running ES Score & Positions of GeneSet Members on the Rank Ordered List

  

| SYMBOL | TITLE | RANK IN GENE LIST | RANK METRIC SCORE | RUNNING ES | CORE ENRICHMENT || 1 | CASP1 | na | 78 | 1.069 | 0.3438 | Yes |
| 2 | CD48 | na | 195 | 0.799 | 0.5983 | Yes |
| 3 | MPO | na | 5052 | 0.093 | 0.4064 | No |
| 4 | AIM1 | na | 5791 | 0.053 | 0.3898 | No |
| 5 | CEBPA | na | 7443 | 0.000 | 0.3143 | No |
| 6 | TYROBP | na | 8675 | 0.000 | 0.2579 | No |
| 7 | CXCR4 | na | 9111 | 0.000 | 0.2380 | No |
| 8 | CTSG | na | 9566 | 0.000 | 0.2173 | No |
| 9 | MYCN | na | 11199 | 0.000 | 0.1426 | No |
| 10 | ELANE | na | 11444 | 0.000 | 0.1315 | No |
| 11 | BCL6 | na | 15341 | -0.088 | -0.0180 | No |
| 12 | PECAM1 | na | 16208 | -0.141 | -0.0118 | No |
| 13 | CD44 | na | 16720 | -0.178 | 0.0227 | No |
| 14 | CCDC159 | na | 17573 | -0.235 | 0.0601 | No |
| 15 | FOSL2 | na | 19737 | -0.420 | 0.0978 | No |
Table: GSEA details [plain text format]

  

Fig 2: SCHURINGA\_STAT5A\_TARGETS\_DN      
 Blue-Pink O' Gram in the Space of the Analyzed GeneSet

  

Fig 3: SCHURINGA\_STAT5A\_TARGETS\_DN: Random ES distribution      
 Gene set null distribution of ES for **SCHURINGA\_STAT5A\_TARGETS\_DN**
