# Supplementary material for: Regulation of microglia related neuroinflammation contributes to the protective effect of Gelsevirine on ischemic stroke
Source: Front Immunol. 2023 Mar 30;14:1164278. doi: 10.3389/fimmu.2023.1164278 (PMC10098192; doi:10.3389/fimmu.2023.1164278)
Supplement: Supplementary file 6 [file DataSheet_6.zip › fig 5 raw/fig 5-G raw/inflammation.Gsea.1649955060129/SCHURINGA_STAT5A_TARGETS_UP.html]

Details for gene set SCHURINGA\_STAT5A\_TARGETS\_UP[GSEA]

|  || Dataset | OGD\_DRUG\_DRUG.OGD\_FRUG.cls#Gs\_versus\_MCAO.OGD\_FRUG.cls#Gs\_versus\_MCAO\_repos |
| Phenotype | OGD\_FRUG.cls#Gs\_versus\_MCAO\_repos |
| Upregulated in class | Gs |
| GeneSet | SCHURINGA\_STAT5A\_TARGETS\_UP |
| Enrichment Score (ES) | 0.37255794 |
| Normalized Enrichment Score (NES) | 0.8822684 |
| Nominal p-value | 0.63611114 |
| FDR q-value | 0.94303364 |
| FWER p-Value | 1.0 |
Table: GSEA Results Summary

  

Fig 1: Enrichment plot: SCHURINGA\_STAT5A\_TARGETS\_UP      
 Profile of the Running ES Score & Positions of GeneSet Members on the Rank Ordered List

  

| SYMBOL | TITLE | RANK IN GENE LIST | RANK METRIC SCORE | RUNNING ES | CORE ENRICHMENT || 1 | AQP3 | na | 175 | 0.828 | 0.1860 | Yes |
| 2 | RAC3 | na | 194 | 0.799 | 0.3726 | Yes |
| 3 | CD36 | na | 2486 | 0.287 | 0.3351 | No |
| 4 | PDAP1 | na | 3448 | 0.203 | 0.3387 | No |
| 5 | MAOA | na | 4152 | 0.153 | 0.3425 | No |
| 6 | VEGFA | na | 5039 | 0.094 | 0.3239 | No |
| 7 | LIF | na | 6922 | 0.005 | 0.2389 | No |
| 8 | XK | na | 7745 | 0.000 | 0.2013 | No |
| 9 | KLHL29 | na | 10601 | 0.000 | 0.0707 | No |
| 10 | RHAG | na | 11460 | 0.000 | 0.0315 | No |
| 11 | ANK1 | na | 14036 | -0.021 | -0.0814 | No |
| 12 | FOSB | na | 14731 | -0.052 | -0.1008 | No |
| 13 | TRAF4 | na | 15537 | -0.100 | -0.1141 | No |
| 14 | PIM1 | na | 16160 | -0.138 | -0.1101 | No |
| 15 | LEPR | na | 17310 | -0.217 | -0.1117 | No |
| 16 | SOS2 | na | 19626 | -0.409 | -0.1217 | No |
| 17 | CISH | na | 21772 | -0.958 | 0.0047 | No |
Table: GSEA details [plain text format]

  

Fig 2: SCHURINGA\_STAT5A\_TARGETS\_UP      
 Blue-Pink O' Gram in the Space of the Analyzed GeneSet

  

Fig 3: SCHURINGA\_STAT5A\_TARGETS\_UP: Random ES distribution      
 Gene set null distribution of ES for **SCHURINGA\_STAT5A\_TARGETS\_UP**
