# Supplementary material for: Regulation of microglia related neuroinflammation contributes to the protective effect of Gelsevirine on ischemic stroke
Source: Front Immunol. 2023 Mar 30;14:1164278. doi: 10.3389/fimmu.2023.1164278 (PMC10098192; doi:10.3389/fimmu.2023.1164278)
Supplement: Supplementary file 6 [file DataSheet_6.zip › fig 5 raw/fig 5-G raw/inflammation.Gsea.1649955060129/ST_JNK_MAPK_PATHWAY.html]

Details for gene set ST\_JNK\_MAPK\_PATHWAY[GSEA]

|  || Dataset | OGD\_DRUG\_DRUG.OGD\_FRUG.cls#Gs\_versus\_MCAO.OGD\_FRUG.cls#Gs\_versus\_MCAO\_repos |
| Phenotype | OGD\_FRUG.cls#Gs\_versus\_MCAO\_repos |
| Upregulated in class | MCAO |
| GeneSet | ST\_JNK\_MAPK\_PATHWAY |
| Enrichment Score (ES) | -0.5720484 |
| Normalized Enrichment Score (NES) | -1.4870596 |
| Nominal p-value | 0.016949153 |
| FDR q-value | 0.09048738 |
| FWER p-Value | 0.77 |
Table: GSEA Results Summary

  

Fig 1: Enrichment plot: ST\_JNK\_MAPK\_PATHWAY      
 Profile of the Running ES Score & Positions of GeneSet Members on the Rank Ordered List

  

| SYMBOL | TITLE | RANK IN GENE LIST | RANK METRIC SCORE | RUNNING ES | CORE ENRICHMENT || 1 | GADD45A | na | 205 | 0.788 | 0.0651 | No |
| 2 | MAP3K5 | na | 1224 | 0.432 | 0.0593 | No |
| 3 | DLD | na | 3029 | 0.238 | -0.0008 | No |
| 4 | CDC42 | na | 4900 | 0.101 | -0.0769 | No |
| 5 | JUN | na | 5874 | 0.048 | -0.1169 | No |
| 6 | MAP3K9 | na | 6500 | 0.021 | -0.1436 | No |
| 7 | DUSP10 | na | 6651 | 0.015 | -0.1490 | No |
| 8 | MAPK9 | na | 6752 | 0.011 | -0.1526 | No |
| 9 | GCK | na | 8093 | 0.000 | -0.2139 | No |
| 10 | PAPPA | na | 13560 | -0.009 | -0.4634 | No |
| 11 | MYEF2 | na | 14145 | -0.025 | -0.4878 | No |
| 12 | TAB1 | na | 14274 | -0.030 | -0.4908 | No |
| 13 | MAP3K12 | na | 15613 | -0.104 | -0.5422 | No |
| 14 | MAP3K10 | na | 16054 | -0.132 | -0.5499 | No |
| 15 | MAPK10 | na | 16183 | -0.140 | -0.5426 | No |
| 16 | AKT1 | na | 16827 | -0.184 | -0.5547 | Yes |
| 17 | MAP2K4 | na | 16904 | -0.189 | -0.5403 | Yes |
| 18 | MAP3K7 | na | 17079 | -0.201 | -0.5292 | Yes |
| 19 | ATF2 | na | 17196 | -0.209 | -0.5148 | Yes |
| 20 | MAP2K5 | na | 17477 | -0.229 | -0.5061 | Yes |
| 21 | TAB2 | na | 17639 | -0.240 | -0.4908 | Yes |
| 22 | DUSP8 | na | 17719 | -0.246 | -0.4712 | Yes |
| 23 | MAPK8 | na | 17866 | -0.258 | -0.4535 | Yes |
| 24 | DUSP4 | na | 17969 | -0.267 | -0.4329 | Yes |
| 25 | IL1R1 | na | 18062 | -0.274 | -0.4113 | Yes |
| 26 | MAPK7 | na | 18686 | -0.320 | -0.4096 | Yes |
| 27 | TRAF6 | na | 18711 | -0.321 | -0.3804 | Yes |
| 28 | MAP2K7 | na | 18727 | -0.323 | -0.3506 | Yes |
| 29 | MAP3K3 | na | 18987 | -0.348 | -0.3296 | Yes |
| 30 | SHC1 | na | 19109 | -0.361 | -0.3011 | Yes |
| 31 | MAP3K11 | na | 19289 | -0.377 | -0.2737 | Yes |
| 32 | ZAK | na | 19557 | -0.403 | -0.2478 | Yes |
| 33 | GAB1 | na | 19622 | -0.409 | -0.2121 | Yes |
| 34 | NR2C2 | na | 19944 | -0.439 | -0.1853 | Yes |
| 35 | NFATC3 | na | 20856 | -0.555 | -0.1747 | Yes |
| 36 | MAP3K2 | na | 20871 | -0.556 | -0.1227 | Yes |
| 37 | MAP3K1 | na | 20919 | -0.563 | -0.0717 | Yes |
| 38 | MAP3K13 | na | 21141 | -0.606 | -0.0246 | Yes |
| 39 | MAP3K4 | na | 21183 | -0.616 | 0.0317 | Yes |
Table: GSEA details [plain text format]

  

Fig 2: ST\_JNK\_MAPK\_PATHWAY      
 Blue-Pink O' Gram in the Space of the Analyzed GeneSet

  

Fig 3: ST\_JNK\_MAPK\_PATHWAY: Random ES distribution      
 Gene set null distribution of ES for **ST\_JNK\_MAPK\_PATHWAY**
