# Supplementary material for: Regulation of microglia related neuroinflammation contributes to the protective effect of Gelsevirine on ischemic stroke
Source: Front Immunol. 2023 Mar 30;14:1164278. doi: 10.3389/fimmu.2023.1164278 (PMC10098192; doi:10.3389/fimmu.2023.1164278)
Supplement: Supplementary file 6 [file DataSheet_6.zip › fig 5 raw/fig 5-G raw/inflammation.Gsea.1649955060129/ST_WNT_BETA_CATENIN_PATHWAY.html]

Details for gene set ST\_WNT\_BETA\_CATENIN\_PATHWAY[GSEA]

|  || Dataset | OGD\_DRUG\_DRUG.OGD\_FRUG.cls#Gs\_versus\_MCAO.OGD\_FRUG.cls#Gs\_versus\_MCAO\_repos |
| Phenotype | OGD\_FRUG.cls#Gs\_versus\_MCAO\_repos |
| Upregulated in class | MCAO |
| GeneSet | ST\_WNT\_BETA\_CATENIN\_PATHWAY |
| Enrichment Score (ES) | -0.6083377 |
| Normalized Enrichment Score (NES) | -1.5179933 |
| Nominal p-value | 0.013513514 |
| FDR q-value | 0.0980183 |
| FWER p-Value | 0.638 |
Table: GSEA Results Summary

  

Fig 1: Enrichment plot: ST\_WNT\_BETA\_CATENIN\_PATHWAY      
 Profile of the Running ES Score & Positions of GeneSet Members on the Rank Ordered List

  

| SYMBOL | TITLE | RANK IN GENE LIST | RANK METRIC SCORE | RUNNING ES | CORE ENRICHMENT || 1 | RPSA | na | 1107 | 0.447 | 0.0217 | No |
| 2 | DACT1 | na | 5133 | 0.088 | -0.1483 | No |
| 3 | MVP | na | 5864 | 0.048 | -0.1739 | No |
| 4 | PIN1 | na | 6161 | 0.034 | -0.1820 | No |
| 5 | SFRP1 | na | 6881 | 0.006 | -0.2139 | No |
| 6 | CBY1 | na | 6974 | 0.002 | -0.2177 | No |
| 7 | DKK1 | na | 7803 | 0.000 | -0.2556 | No |
| 8 | DKK2 | na | 7804 | 0.000 | -0.2556 | No |
| 9 | DKK4 | na | 7805 | 0.000 | -0.2556 | No |
| 10 | NKD1 | na | 8211 | 0.000 | -0.2742 | No |
| 11 | NKD2 | na | 8212 | 0.000 | -0.2742 | No |
| 12 | AXIN2 | na | 8241 | 0.000 | -0.2755 | No |
| 13 | WIF1 | na | 8770 | 0.000 | -0.2996 | No |
| 14 | CXXC4 | na | 12839 | 0.000 | -0.4859 | No |
| 15 | GSK3A | na | 14248 | -0.030 | -0.5456 | No |
| 16 | CER1 | na | 14683 | -0.050 | -0.5574 | No |
| 17 | DKK3 | na | 15417 | -0.093 | -0.5758 | No |
| 18 | SENP2 | na | 16128 | -0.137 | -0.5862 | Yes |
| 19 | PSEN1 | na | 16462 | -0.159 | -0.5758 | Yes |
| 20 | CSNK1A1 | na | 16678 | -0.175 | -0.5572 | Yes |
| 21 | AKT2 | na | 16784 | -0.181 | -0.5327 | Yes |
| 22 | AKT1 | na | 16827 | -0.184 | -0.5048 | Yes |
| 23 | PTPRA | na | 16966 | -0.194 | -0.4797 | Yes |
| 24 | TSHB | na | 16977 | -0.194 | -0.4487 | Yes |
| 25 | DVL1 | na | 17888 | -0.261 | -0.4482 | Yes |
| 26 | AKT3 | na | 17899 | -0.261 | -0.4064 | Yes |
| 27 | ANKRD6 | na | 17963 | -0.267 | -0.3661 | Yes |
| 28 | AXIN1 | na | 18098 | -0.276 | -0.3276 | Yes |
| 29 | CTNNB1 | na | 18285 | -0.290 | -0.2891 | Yes |
| 30 | FSTL1 | na | 18483 | -0.302 | -0.2492 | Yes |
| 31 | GSK3B | na | 20096 | -0.455 | -0.2494 | Yes |
| 32 | APC | na | 21200 | -0.619 | -0.1997 | Yes |
| 33 | LRP1 | na | 21402 | -0.669 | -0.1006 | Yes |
| 34 | FRAT1 | na | 21598 | -0.755 | 0.0127 | Yes |
Table: GSEA details [plain text format]

  

Fig 2: ST\_WNT\_BETA\_CATENIN\_PATHWAY      
 Blue-Pink O' Gram in the Space of the Analyzed GeneSet

  

Fig 3: ST\_WNT\_BETA\_CATENIN\_PATHWAY: Random ES distribution      
 Gene set null distribution of ES for **ST\_WNT\_BETA\_CATENIN\_PATHWAY**
