# Supplementary material for: Regulation of microglia related neuroinflammation contributes to the protective effect of Gelsevirine on ischemic stroke
Source: Front Immunol. 2023 Mar 30;14:1164278. doi: 10.3389/fimmu.2023.1164278 (PMC10098192; doi:10.3389/fimmu.2023.1164278)
Supplement: Supplementary file 6 [file DataSheet_6.zip › fig 5 raw/fig 5-G raw/inflammation.Gsea.1649955060129/WIERENGA_STAT5A_TARGETS_GROUP2.html]

Details for gene set WIERENGA\_STAT5A\_TARGETS\_GROUP2[GSEA]

|  || Dataset | OGD\_DRUG\_DRUG.OGD\_FRUG.cls#Gs\_versus\_MCAO.OGD\_FRUG.cls#Gs\_versus\_MCAO\_repos |
| Phenotype | OGD\_FRUG.cls#Gs\_versus\_MCAO\_repos |
| Upregulated in class | Gs |
| GeneSet | WIERENGA\_STAT5A\_TARGETS\_GROUP2 |
| Enrichment Score (ES) | 0.29058132 |
| Normalized Enrichment Score (NES) | 0.86204416 |
| Nominal p-value | 0.76438355 |
| FDR q-value | 0.9418902 |
| FWER p-Value | 1.0 |
Table: GSEA Results Summary

  

Fig 1: Enrichment plot: WIERENGA\_STAT5A\_TARGETS\_GROUP2      
 Profile of the Running ES Score & Positions of GeneSet Members on the Rank Ordered List

  

| SYMBOL | TITLE | RANK IN GENE LIST | RANK METRIC SCORE | RUNNING ES | CORE ENRICHMENT || 1 | CCL5 | na | 495 | 0.590 | 0.0350 | Yes |
| 2 | CXCL10 | na | 497 | 0.590 | 0.0927 | Yes |
| 3 | CCL2 | na | 556 | 0.569 | 0.1457 | Yes |
| 4 | SLC2A3 | na | 1264 | 0.426 | 0.1549 | Yes |
| 5 | GADD45B | na | 1594 | 0.388 | 0.1778 | Yes |
| 6 | MOB3B | na | 1616 | 0.385 | 0.2145 | Yes |
| 7 | CSF2 | na | 1966 | 0.342 | 0.2320 | Yes |
| 8 | MAPK13 | na | 2791 | 0.260 | 0.2196 | Yes |
| 9 | BTG2 | na | 2846 | 0.255 | 0.2421 | Yes |
| 10 | SQSTM1 | na | 3060 | 0.236 | 0.2554 | Yes |
| 11 | GABARAPL1 | na | 3089 | 0.234 | 0.2770 | Yes |
| 12 | SPP1 | na | 3259 | 0.218 | 0.2906 | Yes |
| 13 | LAMP3 | na | 3919 | 0.172 | 0.2772 | No |
| 14 | NFIL3 | na | 4292 | 0.143 | 0.2741 | No |
| 15 | CCR7 | na | 4335 | 0.141 | 0.2859 | No |
| 16 | PSAT1 | na | 4682 | 0.116 | 0.2814 | No |
| 17 | BHLHE40 | na | 5305 | 0.079 | 0.2606 | No |
| 18 | KLF2 | na | 5535 | 0.066 | 0.2566 | No |
| 19 | CD83 | na | 6012 | 0.040 | 0.2388 | No |
| 20 | EGFL6 | na | 6198 | 0.032 | 0.2335 | No |
| 21 | COCH | na | 6229 | 0.031 | 0.2351 | No |
| 22 | FPR3 | na | 7557 | 0.000 | 0.1743 | No |
| 23 | SOCS2 | na | 7627 | 0.000 | 0.1712 | No |
| 24 | OLR1 | na | 8004 | 0.000 | 0.1540 | No |
| 25 | GPR65 | na | 8318 | 0.000 | 0.1396 | No |
| 26 | PRDM8 | na | 8671 | 0.000 | 0.1235 | No |
| 27 | CCL22 | na | 9713 | 0.000 | 0.0758 | No |
| 28 | STAP1 | na | 10794 | 0.000 | 0.0263 | No |
| 29 | IL7R | na | 10988 | 0.000 | 0.0175 | No |
| 30 | RGS1 | na | 11368 | 0.000 | 0.0001 | No |
| 31 | CCL3 | na | 12847 | 0.000 | -0.0676 | No |
| 32 | TUBB2B | na | 14661 | -0.049 | -0.1459 | No |
| 33 | TRAF1 | na | 15135 | -0.076 | -0.1602 | No |
| 34 | HOXB5 | na | 15205 | -0.080 | -0.1555 | No |
| 35 | ABCA1 | na | 15285 | -0.085 | -0.1508 | No |
| 36 | AK3 | na | 15776 | -0.114 | -0.1621 | No |
| 37 | GALC | na | 16104 | -0.134 | -0.1640 | No |
| 38 | EMP1 | na | 16227 | -0.142 | -0.1556 | No |
| 39 | KLF6 | na | 16483 | -0.160 | -0.1516 | No |
| 40 | PLEKHH3 | na | 16865 | -0.187 | -0.1508 | No |
| 41 | CKAP4 | na | 18000 | -0.269 | -0.1765 | No |
| 42 | PLAU | na | 18269 | -0.289 | -0.1605 | No |
| 43 | RNF217 | na | 18559 | -0.309 | -0.1435 | No |
| 44 | ANTXR2 | na | 18794 | -0.329 | -0.1220 | No |
| 45 | TSC22D2 | na | 18967 | -0.346 | -0.0961 | No |
| 46 | PHTF2 | na | 19203 | -0.370 | -0.0707 | No |
| 47 | TANC1 | na | 20086 | -0.453 | -0.0668 | No |
| 48 | COL6A1 | na | 20104 | -0.456 | -0.0230 | No |
| 49 | ARID5B | na | 20197 | -0.466 | 0.0184 | No |
| 50 | GNPTAB | na | 21092 | -0.597 | 0.0359 | No |
Table: GSEA details [plain text format]

  

Fig 2: WIERENGA\_STAT5A\_TARGETS\_GROUP2      
 Blue-Pink O' Gram in the Space of the Analyzed GeneSet

  

Fig 3: WIERENGA\_STAT5A\_TARGETS\_GROUP2: Random ES distribution      
 Gene set null distribution of ES for **WIERENGA\_STAT5A\_TARGETS\_GROUP2**
