# Supplementary material for: Regulation of microglia related neuroinflammation contributes to the protective effect of Gelsevirine on ischemic stroke
Source: Front Immunol. 2023 Mar 30;14:1164278. doi: 10.3389/fimmu.2023.1164278 (PMC10098192; doi:10.3389/fimmu.2023.1164278)
Supplement: Supplementary file 6 [file DataSheet_6.zip › fig 5 raw/fig 5-G raw/inflammation.Gsea.1649955060129/WILLERT_WNT_SIGNALING.html]

Details for gene set WILLERT\_WNT\_SIGNALING[GSEA]

|  || Dataset | OGD\_DRUG\_DRUG.OGD\_FRUG.cls#Gs\_versus\_MCAO.OGD\_FRUG.cls#Gs\_versus\_MCAO\_repos |
| Phenotype | OGD\_FRUG.cls#Gs\_versus\_MCAO\_repos |
| Upregulated in class | Gs |
| GeneSet | WILLERT\_WNT\_SIGNALING |
| Enrichment Score (ES) | 0.29161614 |
| Normalized Enrichment Score (NES) | 0.7086308 |
| Nominal p-value | 0.9390863 |
| FDR q-value | 0.9654297 |
| FWER p-Value | 1.0 |
Table: GSEA Results Summary

  

Fig 1: Enrichment plot: WILLERT\_WNT\_SIGNALING      
 Profile of the Running ES Score & Positions of GeneSet Members on the Rank Ordered List

  

| SYMBOL | TITLE | RANK IN GENE LIST | RANK METRIC SCORE | RUNNING ES | CORE ENRICHMENT || 1 | SRP19 | na | 396 | 0.632 | 0.1342 | Yes |
| 2 | RPL27A | na | 1302 | 0.421 | 0.1942 | Yes |
| 3 | SNRPA1 | na | 1357 | 0.414 | 0.2916 | Yes |
| 4 | ZRSR1 | na | 3399 | 0.207 | 0.2481 | No |
| 5 | TLE1 | na | 4909 | 0.100 | 0.2032 | No |
| 6 | GSTM3 | na | 7079 | 0.000 | 0.1040 | No |
| 7 | TNFRSF11B | na | 7622 | 0.000 | 0.0792 | No |
| 8 | MSX2 | na | 10055 | 0.000 | -0.0321 | No |
| 9 | EDNRB | na | 14010 | -0.021 | -0.2080 | No |
| 10 | ID2 | na | 14104 | -0.024 | -0.2066 | No |
| 11 | GYPC | na | 14182 | -0.027 | -0.2036 | No |
| 12 | CITED2 | na | 14873 | -0.060 | -0.2207 | No |
| 13 | CSTF1 | na | 15517 | -0.099 | -0.2263 | No |
| 14 | SLC2A1 | na | 16405 | -0.155 | -0.2297 | No |
| 15 | ING1 | na | 16725 | -0.178 | -0.2013 | No |
| 16 | CCND1 | na | 17421 | -0.225 | -0.1789 | No |
| 17 | GJA1 | na | 17476 | -0.228 | -0.1263 | No |
| 18 | NSMAF | na | 17822 | -0.255 | -0.0806 | No |
| 19 | NCOA3 | na | 20068 | -0.451 | -0.0746 | No |
| 20 | VCAN | na | 21332 | -0.652 | 0.0248 | No |
Table: GSEA details [plain text format]

  

Fig 2: WILLERT\_WNT\_SIGNALING      
 Blue-Pink O' Gram in the Space of the Analyzed GeneSet

  

Fig 3: WILLERT\_WNT\_SIGNALING: Random ES distribution      
 Gene set null distribution of ES for **WILLERT\_WNT\_SIGNALING**
