# Supplementary material for: Regulation of microglia related neuroinflammation contributes to the protective effect of Gelsevirine on ischemic stroke
Source: Front Immunol. 2023 Mar 30;14:1164278. doi: 10.3389/fimmu.2023.1164278 (PMC10098192; doi:10.3389/fimmu.2023.1164278)
Supplement: Supplementary file 6 [file DataSheet_6.zip › fig 5 raw/fig 5-G raw/JAKSTAT.Gsea.1649955169649/gsea_report_for_MCAO_1649955169649.html]

Report for MCAO 1649955169649 [GSEA]

| GS  follow link to MSigDB | GS DETAILS | SIZE | ES | NES | NOM p-val | FDR q-val | FWER p-val | RANK AT MAX | LEADING EDGE || 1 | KEGG\_JAK\_STAT\_SIGNALING\_PATHWAY | Details ... | 141 | -0.45 | -1.41 | 0.006 | 0.006 | 0.005 | 5093 | tags=35%, list=23%, signal=46% |
Table: Gene sets enriched in phenotype **MCAO (3 samples) **[plain text format]****
