# Supplementary material for: Regulation of microglia related neuroinflammation contributes to the protective effect of Gelsevirine on ischemic stroke
Source: Front Immunol. 2023 Mar 30;14:1164278. doi: 10.3389/fimmu.2023.1164278 (PMC10098192; doi:10.3389/fimmu.2023.1164278)
Supplement: Supplementary file 6 [file DataSheet_6.zip › fig 5 raw/fig 5-G raw/JAKSTAT.Gsea.1649955169649/KEGG_JAK_STAT_SIGNALING_PATHWAY.html]

Details for gene set KEGG\_JAK\_STAT\_SIGNALING\_PATHWAY[GSEA]

|  || Dataset | OGD\_DRUG\_DRUG.OGD\_FRUG.cls#Gs\_versus\_MCAO.OGD\_FRUG.cls#Gs\_versus\_MCAO\_repos |
| Phenotype | OGD\_FRUG.cls#Gs\_versus\_MCAO\_repos |
| Upregulated in class | MCAO |
| GeneSet | KEGG\_JAK\_STAT\_SIGNALING\_PATHWAY |
| Enrichment Score (ES) | -0.45415363 |
| Normalized Enrichment Score (NES) | -1.4132956 |
| Nominal p-value | 0.0064102565 |
| FDR q-value | 0.0064102565 |
| FWER p-Value | 0.005 |
Table: GSEA Results Summary

  

Fig 1: Enrichment plot: KEGG\_JAK\_STAT\_SIGNALING\_PATHWAY      
 Profile of the Running ES Score & Positions of GeneSet Members on the Rank Ordered List

  

| SYMBOL | TITLE | RANK IN GENE LIST | RANK METRIC SCORE | RUNNING ES | CORE ENRICHMENT || 1 | CSF2RA | na | 26 | 1.452 | 0.0480 | No |
| 2 | CSF3 | na | 128 | 0.917 | 0.0745 | No |
| 3 | CTF1 | na | 537 | 0.574 | 0.0752 | No |
| 4 | TSLP | na | 730 | 0.518 | 0.0839 | No |
| 5 | IL2RB | na | 822 | 0.494 | 0.0965 | No |
| 6 | IL20RB | na | 980 | 0.467 | 0.1051 | No |
| 7 | PIK3CG | na | 1248 | 0.428 | 0.1073 | No |
| 8 | PTPN6 | na | 1809 | 0.361 | 0.0938 | No |
| 9 | CSF2 | na | 1966 | 0.342 | 0.0982 | No |
| 10 | STAT4 | na | 2555 | 0.282 | 0.0807 | No |
| 11 | IL11 | na | 3251 | 0.219 | 0.0561 | No |
| 12 | IL12B | na | 3745 | 0.183 | 0.0396 | No |
| 13 | IL19 | na | 3774 | 0.181 | 0.0445 | No |
| 14 | SPRY2 | na | 4356 | 0.139 | 0.0225 | No |
| 15 | IL13 | na | 4636 | 0.119 | 0.0137 | No |
| 16 | BCL2L1 | na | 4927 | 0.100 | 0.0037 | No |
| 17 | SOCS1 | na | 5006 | 0.095 | 0.0034 | No |
| 18 | IFNB1 | na | 5085 | 0.091 | 0.0029 | No |
| 19 | IL2 | na | 5219 | 0.084 | -0.0004 | No |
| 20 | IL20 | na | 5308 | 0.079 | -0.0018 | No |
| 21 | MYC | na | 5587 | 0.064 | -0.0124 | No |
| 22 | SPRY1 | na | 5649 | 0.060 | -0.0132 | No |
| 23 | GRB2 | na | 5726 | 0.057 | -0.0148 | No |
| 24 | CBLC | na | 5841 | 0.050 | -0.0183 | No |
| 25 | OSM | na | 5924 | 0.045 | -0.0205 | No |
| 26 | IL5RA | na | 6464 | 0.023 | -0.0446 | No |
| 27 | CNTFR | na | 6487 | 0.021 | -0.0449 | No |
| 28 | CSF2RB | na | 6547 | 0.019 | -0.0470 | No |
| 29 | IFNGR2 | na | 6555 | 0.018 | -0.0467 | No |
| 30 | PRLR | na | 6858 | 0.006 | -0.0603 | No |
| 31 | IL13RA1 | na | 6882 | 0.006 | -0.0612 | No |
| 32 | LIF | na | 6922 | 0.005 | -0.0628 | No |
| 33 | IL10RB | na | 6952 | 0.003 | -0.0640 | No |
| 34 | CCND3 | na | 6975 | 0.002 | -0.0650 | No |
| 35 | IL21R | na | 7454 | 0.000 | -0.0869 | No |
| 36 | IL23R | na | 7469 | 0.000 | -0.0876 | No |
| 37 | SOCS2 | na | 7627 | 0.000 | -0.0948 | No |
| 38 | EPO | na | 7843 | 0.000 | -0.1047 | No |
| 39 | IL3RA | na | 7909 | 0.000 | -0.1077 | No |
| 40 | IL4 | na | 8412 | 0.000 | -0.1308 | No |
| 41 | IL3 | na | 8413 | 0.000 | -0.1308 | No |
| 42 | IL9 | na | 8414 | 0.000 | -0.1308 | No |
| 43 | IFNA13 | na | 8616 | 0.000 | -0.1400 | No |
| 44 | IFNA14 | na | 8617 | 0.000 | -0.1400 | No |
| 45 | IFNA16 | na | 8619 | 0.000 | -0.1401 | No |
| 46 | LEP | na | 8744 | 0.000 | -0.1458 | No |
| 47 | MPL | na | 8985 | 0.000 | -0.1568 | No |
| 48 | IL12RB2 | na | 9182 | 0.000 | -0.1658 | No |
| 49 | IFNA5 | na | 9402 | 0.000 | -0.1759 | No |
| 50 | IFNA7 | na | 9403 | 0.000 | -0.1759 | No |
| 51 | IFNA6 | na | 9404 | 0.000 | -0.1759 | No |
| 52 | IFNA1 | na | 9405 | 0.000 | -0.1759 | No |
| 53 | IFNA2 | na | 9406 | 0.000 | -0.1759 | No |
| 54 | PRL | na | 9408 | 0.000 | -0.1760 | No |
| 55 | IFNL2 | na | 9461 | 0.000 | -0.1784 | No |
| 56 | IFNL3 | na | 9462 | 0.000 | -0.1784 | No |
| 57 | IL20RA | na | 9473 | 0.000 | -0.1788 | No |
| 58 | IFNG | na | 9828 | 0.000 | -0.1951 | No |
| 59 | IFNK | na | 9829 | 0.000 | -0.1951 | No |
| 60 | IL10RA | na | 10241 | 0.000 | -0.2140 | No |
| 61 | TPO | na | 10272 | 0.000 | -0.2154 | No |
| 62 | IL21 | na | 10903 | 0.000 | -0.2444 | No |
| 63 | IL7R | na | 10988 | 0.000 | -0.2483 | No |
| 64 | IL22RA2 | na | 11279 | 0.000 | -0.2616 | No |
| 65 | IL22RA1 | na | 11284 | 0.000 | -0.2618 | No |
| 66 | CSF3R | na | 12165 | 0.000 | -0.3023 | No |
| 67 | IL12A | na | 12867 | 0.000 | -0.3345 | No |
| 68 | IFNA4 | na | 13482 | -0.007 | -0.3625 | No |
| 69 | LIFR | na | 13712 | -0.013 | -0.3726 | No |
| 70 | CLCF1 | na | 13910 | -0.017 | -0.3811 | No |
| 71 | IL2RA | na | 14028 | -0.021 | -0.3858 | No |
| 72 | IL12RB1 | na | 14229 | -0.029 | -0.3940 | No |
| 73 | IFNAR2 | na | 14289 | -0.031 | -0.3957 | No |
| 74 | EPOR | na | 14314 | -0.033 | -0.3957 | No |
| 75 | IFNGR1 | na | 14409 | -0.036 | -0.3988 | No |
| 76 | IFNLR1 | na | 14535 | -0.042 | -0.4031 | No |
| 77 | STAT2 | na | 14734 | -0.053 | -0.4104 | No |
| 78 | CRLF2 | na | 14829 | -0.057 | -0.4128 | No |
| 79 | IL10 | na | 14957 | -0.065 | -0.4164 | No |
| 80 | IL13RA2 | na | 15123 | -0.075 | -0.4215 | No |
| 81 | JAK2 | na | 15175 | -0.079 | -0.4212 | No |
| 82 | IL15RA | na | 15258 | -0.084 | -0.4221 | No |
| 83 | IL9R | na | 15602 | -0.104 | -0.4344 | No |
| 84 | CNTF | na | 15663 | -0.107 | -0.4335 | No |
| 85 | PIAS2 | na | 15673 | -0.107 | -0.4303 | No |
| 86 | IL15 | na | 15722 | -0.110 | -0.4288 | No |
| 87 | PIAS4 | na | 15887 | -0.121 | -0.4322 | No |
| 88 | IL22 | na | 15910 | -0.122 | -0.4291 | No |
| 89 | PIM1 | na | 16160 | -0.138 | -0.4359 | No |
| 90 | IL5 | na | 16224 | -0.142 | -0.4339 | No |
| 91 | STAT6 | na | 16496 | -0.161 | -0.4409 | No |
| 92 | AKT2 | na | 16784 | -0.181 | -0.4480 | Yes |
| 93 | AKT1 | na | 16827 | -0.184 | -0.4437 | Yes |
| 94 | SOCS3 | na | 16838 | -0.185 | -0.4379 | Yes |
| 95 | STAM | na | 17007 | -0.197 | -0.4390 | Yes |
| 96 | LEPR | na | 17310 | -0.217 | -0.4455 | Yes |
| 97 | CCND1 | na | 17421 | -0.225 | -0.4429 | Yes |
| 98 | IRF9 | na | 17430 | -0.226 | -0.4356 | Yes |
| 99 | IFNAR1 | na | 17709 | -0.245 | -0.4401 | Yes |
| 100 | PIAS1 | na | 17735 | -0.247 | -0.4329 | Yes |
| 101 | STAT1 | na | 17788 | -0.252 | -0.4267 | Yes |
| 102 | IL23A | na | 17897 | -0.261 | -0.4228 | Yes |
| 103 | AKT3 | na | 17899 | -0.261 | -0.4140 | Yes |
| 104 | GHR | na | 17916 | -0.263 | -0.4058 | Yes |
| 105 | SOCS7 | na | 18195 | -0.284 | -0.4090 | Yes |
| 106 | PIK3CD | na | 18197 | -0.284 | -0.3994 | Yes |
| 107 | OSMR | na | 18234 | -0.287 | -0.3913 | Yes |
| 108 | CCND2 | na | 18282 | -0.290 | -0.3837 | Yes |
| 109 | STAM2 | na | 18358 | -0.294 | -0.3771 | Yes |
| 110 | PIK3R2 | na | 18436 | -0.299 | -0.3706 | Yes |
| 111 | IL24 | na | 18467 | -0.301 | -0.3617 | Yes |
| 112 | PIK3CA | na | 18513 | -0.305 | -0.3535 | Yes |
| 113 | IL2RG | na | 18663 | -0.317 | -0.3495 | Yes |
| 114 | SPRED1 | na | 19084 | -0.358 | -0.3567 | Yes |
| 115 | IL6 | na | 19212 | -0.371 | -0.3500 | Yes |
| 116 | TYK2 | na | 19279 | -0.375 | -0.3403 | Yes |
| 117 | PIK3R3 | na | 19336 | -0.380 | -0.3300 | Yes |
| 118 | SOCS5 | na | 19449 | -0.392 | -0.3219 | Yes |
| 119 | STAT5A | na | 19487 | -0.396 | -0.3101 | Yes |
| 120 | PTPN11 | na | 19508 | -0.397 | -0.2976 | Yes |
| 121 | PIK3R5 | na | 19509 | -0.397 | -0.2841 | Yes |
| 122 | SOS2 | na | 19626 | -0.409 | -0.2756 | Yes |
| 123 | IL7 | na | 19686 | -0.415 | -0.2642 | Yes |
| 124 | JAK1 | na | 20014 | -0.446 | -0.2642 | Yes |
| 125 | SOCS4 | na | 20052 | -0.450 | -0.2506 | Yes |
| 126 | JAK3 | na | 20139 | -0.460 | -0.2390 | Yes |
| 127 | CBLB | na | 20162 | -0.463 | -0.2243 | Yes |
| 128 | PIK3CB | na | 20535 | -0.507 | -0.2242 | Yes |
| 129 | SOS1 | na | 20563 | -0.511 | -0.2081 | Yes |
| 130 | STAT3 | na | 20708 | -0.529 | -0.1968 | Yes |
| 131 | IL6ST | na | 20881 | -0.558 | -0.1858 | Yes |
| 132 | PIAS3 | na | 20932 | -0.565 | -0.1690 | Yes |
| 133 | STAT5B | na | 20976 | -0.573 | -0.1515 | Yes |
| 134 | SPRED2 | na | 21188 | -0.617 | -0.1403 | Yes |
| 135 | SPRY4 | na | 21209 | -0.622 | -0.1201 | Yes |
| 136 | CBL | na | 21212 | -0.623 | -0.0991 | Yes |
| 137 | CREBBP | na | 21304 | -0.643 | -0.0815 | Yes |
| 138 | EP300 | na | 21416 | -0.674 | -0.0637 | Yes |
| 139 | PIK3R1 | na | 21419 | -0.674 | -0.0410 | Yes |
| 140 | IFNE | na | 21722 | -0.867 | -0.0255 | Yes |
| 141 | CISH | na | 21772 | -0.958 | 0.0047 | Yes |
Table: GSEA details [plain text format]

  

Fig 2: KEGG\_JAK\_STAT\_SIGNALING\_PATHWAY      
 Blue-Pink O' Gram in the Space of the Analyzed GeneSet

  

Fig 3: KEGG\_JAK\_STAT\_SIGNALING\_PATHWAY: Random ES distribution      
 Gene set null distribution of ES for **KEGG\_JAK\_STAT\_SIGNALING\_PATHWAY**
