# Supplementary material for: Regulation of microglia related neuroinflammation contributes to the protective effect of Gelsevirine on ischemic stroke
Source: Front Immunol. 2023 Mar 30;14:1164278. doi: 10.3389/fimmu.2023.1164278 (PMC10098192; doi:10.3389/fimmu.2023.1164278)
Supplement: Supplementary file 6 [file DataSheet_6.zip › fig 5 raw/fig 5-G raw/KEGG.Gsea.1649954846225/gsea_report_for_Gs_1649954846225.html]

Report for Gs 1649954846225 [GSEA]

| GS  follow link to MSigDB | GS DETAILS | SIZE | ES | NES | NOM p-val | FDR q-val | FWER p-val | RANK AT MAX | LEADING EDGE || 1 | KEGG\_RIBOSOME | Details ... | 84 | 0.89 | 2.85 | 0.000 | 0.000 | 0.000 | 1787 | tags=92%, list=8%, signal=99% |
| 2 | KEGG\_OXIDATIVE\_PHOSPHORYLATION | Details ... | 98 | 0.72 | 2.40 | 0.000 | 0.000 | 0.000 | 3736 | tags=72%, list=17%, signal=87% |
| 3 | KEGG\_PARKINSONS\_DISEASE | Details ... | 96 | 0.72 | 2.37 | 0.000 | 0.000 | 0.000 | 3303 | tags=66%, list=15%, signal=77% |
| 4 | KEGG\_PROTEASOME | Details ... | 43 | 0.78 | 2.19 | 0.000 | 0.000 | 0.000 | 3503 | tags=77%, list=16%, signal=91% |
| 5 | KEGG\_PROTEIN\_EXPORT | Details ... | 21 | 0.81 | 1.99 | 0.002 | 0.000 | 0.001 | 1736 | tags=57%, list=8%, signal=62% |
| 6 | KEGG\_CARDIAC\_MUSCLE\_CONTRACTION | Details ... | 72 | 0.64 | 1.98 | 0.000 | 0.000 | 0.002 | 2718 | tags=36%, list=12%, signal=41% |
| 7 | KEGG\_HUNTINGTONS\_DISEASE | Details ... | 151 | 0.49 | 1.71 | 0.000 | 0.024 | 0.136 | 3390 | tags=53%, list=15%, signal=62% |
| 8 | KEGG\_ALZHEIMERS\_DISEASE | Details ... | 141 | 0.50 | 1.71 | 0.000 | 0.023 | 0.147 | 3756 | tags=49%, list=17%, signal=59% |
| 9 | KEGG\_SPLICEOSOME | Details ... | 122 | 0.50 | 1.68 | 0.000 | 0.026 | 0.189 | 3064 | tags=40%, list=14%, signal=46% |
| 10 | KEGG\_ANTIGEN\_PROCESSING\_AND\_PRESENTATION | Details ... | 51 | 0.56 | 1.64 | 0.006 | 0.037 | 0.272 | 2800 | tags=29%, list=13%, signal=34% |
| 11 | KEGG\_TYROSINE\_METABOLISM | Details ... | 35 | 0.58 | 1.62 | 0.014 | 0.037 | 0.298 | 6281 | tags=57%, list=29%, signal=80% |
| 12 | KEGG\_GLUTATHIONE\_METABOLISM | Details ... | 48 | 0.52 | 1.51 | 0.009 | 0.099 | 0.645 | 4309 | tags=46%, list=20%, signal=57% |
| 13 | KEGG\_DRUG\_METABOLISM\_CYTOCHROME\_P450 | Details ... | 42 | 0.48 | 1.40 | 0.039 | 0.207 | 0.914 | 2988 | tags=36%, list=14%, signal=41% |
| 14 | KEGG\_TYPE\_I\_DIABETES\_MELLITUS | Details ... | 21 | 0.57 | 1.39 | 0.067 | 0.203 | 0.919 | 5838 | tags=29%, list=27%, signal=39% |
| 15 | KEGG\_METABOLISM\_OF\_XENOBIOTICS\_BY\_CYTOCHROME\_P450 | Details ... | 39 | 0.49 | 1.38 | 0.057 | 0.195 | 0.924 | 3458 | tags=41%, list=16%, signal=49% |
| 16 | KEGG\_PROXIMAL\_TUBULE\_BICARBONATE\_RECLAMATION | Details ... | 20 | 0.55 | 1.35 | 0.101 | 0.236 | 0.974 | 2688 | tags=35%, list=12%, signal=40% |
| 17 | KEGG\_GLYCOLYSIS\_GLUCONEOGENESIS | Details ... | 54 | 0.44 | 1.32 | 0.063 | 0.270 | 0.990 | 3667 | tags=33%, list=17%, signal=40% |
| 18 | KEGG\_HISTIDINE\_METABOLISM | Details ... | 27 | 0.49 | 1.29 | 0.114 | 0.304 | 0.995 | 4581 | tags=41%, list=21%, signal=51% |
| 19 | KEGG\_DNA\_REPLICATION | Details ... | 35 | 0.46 | 1.27 | 0.113 | 0.327 | 0.997 | 3481 | tags=40%, list=16%, signal=47% |
| 20 | KEGG\_PURINE\_METABOLISM | Details ... | 148 | 0.36 | 1.26 | 0.028 | 0.337 | 0.998 | 3730 | tags=35%, list=17%, signal=42% |
| 21 | KEGG\_PYRIMIDINE\_METABOLISM |  | 92 | 0.39 | 1.26 | 0.050 | 0.323 | 0.998 | 3596 | tags=41%, list=16%, signal=49% |
| 22 | KEGG\_SNARE\_INTERACTIONS\_IN\_VESICULAR\_TRANSPORT |  | 33 | 0.45 | 1.23 | 0.128 | 0.370 | 0.999 | 2369 | tags=27%, list=11%, signal=31% |
| 23 | KEGG\_NUCLEOTIDE\_EXCISION\_REPAIR |  | 43 | 0.43 | 1.23 | 0.132 | 0.362 | 0.999 | 4103 | tags=42%, list=19%, signal=51% |
| 24 | KEGG\_NOD\_LIKE\_RECEPTOR\_SIGNALING\_PATHWAY |  | 53 | 0.41 | 1.22 | 0.107 | 0.351 | 0.999 | 2214 | tags=21%, list=10%, signal=23% |
| 25 | KEGG\_PHENYLALANINE\_METABOLISM |  | 17 | 0.52 | 1.22 | 0.218 | 0.354 | 0.999 | 2969 | tags=35%, list=14%, signal=41% |
| 26 | KEGG\_BUTANOATE\_METABOLISM |  | 32 | 0.45 | 1.20 | 0.189 | 0.369 | 0.999 | 4192 | tags=34%, list=19%, signal=42% |
| 27 | KEGG\_GALACTOSE\_METABOLISM |  | 24 | 0.46 | 1.19 | 0.219 | 0.384 | 0.999 | 1803 | tags=21%, list=8%, signal=23% |
| 28 | KEGG\_PEROXISOME |  | 77 | 0.37 | 1.19 | 0.104 | 0.371 | 0.999 | 5261 | tags=48%, list=24%, signal=63% |
| 29 | KEGG\_ARACHIDONIC\_ACID\_METABOLISM |  | 44 | 0.42 | 1.18 | 0.188 | 0.386 | 1.000 | 3463 | tags=25%, list=16%, signal=30% |
| 30 | KEGG\_RNA\_POLYMERASE |  | 26 | 0.45 | 1.17 | 0.193 | 0.387 | 1.000 | 3544 | tags=58%, list=16%, signal=69% |
| 31 | KEGG\_PENTOSE\_PHOSPHATE\_PATHWAY |  | 24 | 0.45 | 1.15 | 0.225 | 0.426 | 1.000 | 2657 | tags=25%, list=12%, signal=28% |
| 32 | KEGG\_AMINO\_SUGAR\_AND\_NUCLEOTIDE\_SUGAR\_METABOLISM |  | 40 | 0.40 | 1.14 | 0.220 | 0.441 | 1.000 | 4525 | tags=50%, list=21%, signal=63% |
| 33 | KEGG\_PRIMARY\_IMMUNODEFICIENCY |  | 34 | 0.41 | 1.12 | 0.268 | 0.470 | 1.000 | 3840 | tags=21%, list=18%, signal=25% |
| 34 | KEGG\_AMYOTROPHIC\_LATERAL\_SCLEROSIS\_ALS |  | 52 | 0.37 | 1.08 | 0.284 | 0.567 | 1.000 | 3847 | tags=35%, list=18%, signal=42% |
| 35 | KEGG\_MISMATCH\_REPAIR |  | 22 | 0.44 | 1.08 | 0.308 | 0.559 | 1.000 | 3265 | tags=36%, list=15%, signal=43% |
| 36 | KEGG\_SYSTEMIC\_LUPUS\_ERYTHEMATOSUS |  | 35 | 0.40 | 1.08 | 0.321 | 0.550 | 1.000 | 3406 | tags=23%, list=16%, signal=27% |
| 37 | KEGG\_FATTY\_ACID\_METABOLISM |  | 35 | 0.37 | 1.04 | 0.370 | 0.647 | 1.000 | 4352 | tags=43%, list=20%, signal=53% |
| 38 | KEGG\_CITRATE\_CYCLE\_TCA\_CYCLE |  | 28 | 0.39 | 1.04 | 0.373 | 0.637 | 1.000 | 5129 | tags=54%, list=23%, signal=70% |
| 39 | KEGG\_ASTHMA |  | 15 | 0.45 | 1.04 | 0.365 | 0.623 | 1.000 | 4674 | tags=27%, list=21%, signal=34% |
| 40 | KEGG\_PPAR\_SIGNALING\_PATHWAY |  | 63 | 0.34 | 1.03 | 0.352 | 0.618 | 1.000 | 2501 | tags=24%, list=11%, signal=27% |
| 41 | KEGG\_ABC\_TRANSPORTERS |  | 40 | 0.36 | 1.01 | 0.414 | 0.670 | 1.000 | 604 | tags=13%, list=3%, signal=13% |
| 42 | KEGG\_VIBRIO\_CHOLERAE\_INFECTION |  | 51 | 0.34 | 1.01 | 0.398 | 0.659 | 1.000 | 3578 | tags=33%, list=16%, signal=40% |
| 43 | KEGG\_PYRUVATE\_METABOLISM |  | 37 | 0.37 | 0.99 | 0.452 | 0.701 | 1.000 | 3735 | tags=43%, list=17%, signal=52% |
| 44 | KEGG\_CYTOKINE\_CYTOKINE\_RECEPTOR\_INTERACTION |  | 229 | 0.27 | 0.97 | 0.578 | 0.749 | 1.000 | 980 | tags=8%, list=4%, signal=8% |
| 45 | KEGG\_PRIMARY\_BILE\_ACID\_BIOSYNTHESIS |  | 15 | 0.43 | 0.97 | 0.494 | 0.743 | 1.000 | 2472 | tags=33%, list=11%, signal=38% |
| 46 | KEGG\_INTESTINAL\_IMMUNE\_NETWORK\_FOR\_IGA\_PRODUCTION |  | 32 | 0.36 | 0.96 | 0.503 | 0.754 | 1.000 | 2628 | tags=16%, list=12%, signal=18% |
| 47 | KEGG\_OLFACTORY\_TRANSDUCTION |  | 26 | 0.37 | 0.96 | 0.515 | 0.747 | 1.000 | 4507 | tags=35%, list=21%, signal=44% |
| 48 | KEGG\_VALINE\_LEUCINE\_AND\_ISOLEUCINE\_DEGRADATION |  | 41 | 0.33 | 0.96 | 0.530 | 0.733 | 1.000 | 4623 | tags=39%, list=21%, signal=49% |
| 49 | KEGG\_RNA\_DEGRADATION |  | 55 | 0.32 | 0.95 | 0.553 | 0.746 | 1.000 | 3109 | tags=35%, list=14%, signal=40% |
| 50 | KEGG\_BASAL\_TRANSCRIPTION\_FACTORS |  | 34 | 0.34 | 0.94 | 0.552 | 0.772 | 1.000 | 4739 | tags=44%, list=22%, signal=56% |
| 51 | KEGG\_ALLOGRAFT\_REJECTION |  | 16 | 0.40 | 0.91 | 0.547 | 0.840 | 1.000 | 6465 | tags=31%, list=30%, signal=44% |
| 52 | KEGG\_AUTOIMMUNE\_THYROID\_DISEASE |  | 27 | 0.34 | 0.88 | 0.683 | 0.908 | 1.000 | 5821 | tags=15%, list=27%, signal=20% |
| 53 | KEGG\_HEMATOPOIETIC\_CELL\_LINEAGE |  | 67 | 0.27 | 0.82 | 0.880 | 1.000 | 1.000 | 3251 | tags=15%, list=15%, signal=17% |
| 54 | KEGG\_ARGININE\_AND\_PROLINE\_METABOLISM |  | 50 | 0.26 | 0.79 | 0.924 | 1.000 | 1.000 | 5724 | tags=40%, list=26%, signal=54% |
| 55 | KEGG\_CYTOSOLIC\_DNA\_SENSING\_PATHWAY |  | 49 | 0.26 | 0.78 | 0.915 | 1.000 | 1.000 | 3820 | tags=20%, list=17%, signal=25% |
| 56 | KEGG\_EPITHELIAL\_CELL\_SIGNALING\_IN\_HELICOBACTER\_PYLORI\_INFECTION |  | 66 | 0.25 | 0.77 | 0.970 | 1.000 | 1.000 | 3821 | tags=29%, list=17%, signal=35% |
| 57 | KEGG\_STEROID\_BIOSYNTHESIS |  | 16 | 0.33 | 0.75 | 0.850 | 1.000 | 1.000 | 1929 | tags=19%, list=9%, signal=21% |
| 58 | KEGG\_PORPHYRIN\_AND\_CHLOROPHYLL\_METABOLISM |  | 28 | 0.28 | 0.73 | 0.928 | 1.000 | 1.000 | 3973 | tags=32%, list=18%, signal=39% |
| 59 | KEGG\_GLYOXYLATE\_AND\_DICARBOXYLATE\_METABOLISM |  | 15 | 0.32 | 0.72 | 0.881 | 1.000 | 1.000 | 4353 | tags=40%, list=20%, signal=50% |
| 60 | KEGG\_GLYCINE\_SERINE\_AND\_THREONINE\_METABOLISM |  | 31 | 0.25 | 0.68 | 0.989 | 1.000 | 1.000 | 4945 | tags=39%, list=23%, signal=50% |
| 61 | KEGG\_FRUCTOSE\_AND\_MANNOSE\_METABOLISM |  | 31 | 0.26 | 0.68 | 0.992 | 1.000 | 1.000 | 4376 | tags=32%, list=20%, signal=40% |
| 62 | KEGG\_OTHER\_GLYCAN\_DEGRADATION |  | 16 | 0.30 | 0.67 | 0.928 | 1.000 | 1.000 | 4185 | tags=44%, list=19%, signal=54% |
| 63 | KEGG\_ALANINE\_ASPARTATE\_AND\_GLUTAMATE\_METABOLISM |  | 29 | 0.26 | 0.67 | 0.976 | 1.000 | 1.000 | 2969 | tags=21%, list=14%, signal=24% |
| 64 | KEGG\_TERPENOID\_BACKBONE\_BIOSYNTHESIS |  | 15 | 0.29 | 0.65 | 0.950 | 1.000 | 1.000 | 5636 | tags=60%, list=26%, signal=81% |
| 65 | KEGG\_AMINOACYL\_TRNA\_BIOSYNTHESIS |  | 22 | 0.26 | 0.64 | 0.992 | 1.000 | 1.000 | 5576 | tags=50%, list=25%, signal=67% |
| 66 | KEGG\_SELENOAMINO\_ACID\_METABOLISM |  | 23 | 0.25 | 0.64 | 0.965 | 1.000 | 1.000 | 4581 | tags=30%, list=21%, signal=38% |
| 67 | KEGG\_ONE\_CARBON\_POOL\_BY\_FOLATE |  | 17 | 0.27 | 0.62 | 0.982 | 0.995 | 1.000 | 3675 | tags=35%, list=17%, signal=42% |
Table: Gene sets enriched in phenotype **Gs (3 samples) **[plain text format]****
