# Supplementary material for: Regulation of microglia related neuroinflammation contributes to the protective effect of Gelsevirine on ischemic stroke
Source: Front Immunol. 2023 Mar 30;14:1164278. doi: 10.3389/fimmu.2023.1164278 (PMC10098192; doi:10.3389/fimmu.2023.1164278)
Supplement: Supplementary file 6 [file DataSheet_6.zip › fig 5 raw/fig 5-G raw/KEGG.Gsea.1649954846225/gsea_report_for_MCAO_1649954846225.html]

Report for MCAO 1649954846225 [GSEA]

| GS  follow link to MSigDB | GS DETAILS | SIZE | ES | NES | NOM p-val | FDR q-val | FWER p-val | RANK AT MAX | LEADING EDGE || 1 | KEGG\_PHOSPHATIDYLINOSITOL\_SIGNALING\_SYSTEM | Details ... | 74 | -0.65 | -1.85 | 0.000 | 0.003 | 0.003 | 5320 | tags=59%, list=24%, signal=78% |
| 2 | KEGG\_FOCAL\_ADHESION | Details ... | 196 | -0.55 | -1.75 | 0.000 | 0.019 | 0.040 | 5093 | tags=52%, list=23%, signal=67% |
| 3 | KEGG\_ENDOMETRIAL\_CANCER | Details ... | 51 | -0.64 | -1.75 | 0.000 | 0.013 | 0.042 | 5104 | tags=69%, list=23%, signal=89% |
| 4 | KEGG\_BASAL\_CELL\_CARCINOMA | Details ... | 54 | -0.63 | -1.74 | 0.000 | 0.011 | 0.045 | 5104 | tags=50%, list=23%, signal=65% |
| 5 | KEGG\_ECM\_RECEPTOR\_INTERACTION | Details ... | 83 | -0.59 | -1.72 | 0.000 | 0.013 | 0.067 | 4484 | tags=43%, list=20%, signal=54% |
| 6 | KEGG\_TYPE\_II\_DIABETES\_MELLITUS | Details ... | 46 | -0.64 | -1.70 | 0.000 | 0.015 | 0.096 | 5832 | tags=52%, list=27%, signal=71% |
| 7 | KEGG\_AXON\_GUIDANCE | Details ... | 128 | -0.55 | -1.69 | 0.001 | 0.015 | 0.106 | 4549 | tags=43%, list=21%, signal=54% |
| 8 | KEGG\_INOSITOL\_PHOSPHATE\_METABOLISM | Details ... | 54 | -0.60 | -1.66 | 0.001 | 0.025 | 0.188 | 5875 | tags=61%, list=27%, signal=83% |
| 9 | KEGG\_PATHWAYS\_IN\_CANCER | Details ... | 313 | -0.48 | -1.61 | 0.001 | 0.045 | 0.347 | 5237 | tags=45%, list=24%, signal=58% |
| 10 | KEGG\_ACUTE\_MYELOID\_LEUKEMIA | Details ... | 57 | -0.59 | -1.61 | 0.001 | 0.041 | 0.348 | 6046 | tags=67%, list=28%, signal=92% |
| 11 | KEGG\_DORSO\_VENTRAL\_AXIS\_FORMATION | Details ... | 22 | -0.70 | -1.60 | 0.010 | 0.038 | 0.359 | 2834 | tags=55%, list=13%, signal=63% |
| 12 | KEGG\_SMALL\_CELL\_LUNG\_CANCER | Details ... | 82 | -0.54 | -1.59 | 0.000 | 0.043 | 0.427 | 5237 | tags=56%, list=24%, signal=73% |
| 13 | KEGG\_PROSTATE\_CANCER | Details ... | 86 | -0.54 | -1.59 | 0.000 | 0.040 | 0.427 | 5148 | tags=55%, list=24%, signal=71% |
| 14 | KEGG\_REGULATION\_OF\_ACTIN\_CYTOSKELETON | Details ... | 207 | -0.48 | -1.55 | 0.000 | 0.061 | 0.603 | 4660 | tags=42%, list=21%, signal=53% |
| 15 | KEGG\_ERBB\_SIGNALING\_PATHWAY | Details ... | 86 | -0.51 | -1.53 | 0.000 | 0.066 | 0.667 | 5237 | tags=59%, list=24%, signal=78% |
| 16 | KEGG\_ENDOCYTOSIS | Details ... | 169 | -0.48 | -1.53 | 0.001 | 0.062 | 0.669 | 4264 | tags=46%, list=19%, signal=56% |
| 17 | KEGG\_TGF\_BETA\_SIGNALING\_PATHWAY | Details ... | 84 | -0.53 | -1.53 | 0.003 | 0.059 | 0.673 | 3860 | tags=36%, list=18%, signal=43% |
| 18 | KEGG\_ARRHYTHMOGENIC\_RIGHT\_VENTRICULAR\_CARDIOMYOPATHY\_ARVC | Details ... | 74 | -0.53 | -1.53 | 0.006 | 0.057 | 0.676 | 5286 | tags=38%, list=24%, signal=50% |
| 19 | KEGG\_NON\_SMALL\_CELL\_LUNG\_CANCER | Details ... | 53 | -0.56 | -1.53 | 0.009 | 0.054 | 0.678 | 5141 | tags=55%, list=24%, signal=71% |
| 20 | KEGG\_INSULIN\_SIGNALING\_PATHWAY | Details ... | 132 | -0.49 | -1.50 | 0.003 | 0.068 | 0.775 | 5093 | tags=50%, list=23%, signal=65% |
| 21 | KEGG\_ADHERENS\_JUNCTION |  | 72 | -0.53 | -1.50 | 0.009 | 0.065 | 0.778 | 5104 | tags=54%, list=23%, signal=70% |
| 22 | KEGG\_WNT\_SIGNALING\_PATHWAY |  | 144 | -0.48 | -1.50 | 0.003 | 0.064 | 0.783 | 5415 | tags=41%, list=25%, signal=54% |
| 23 | KEGG\_LONG\_TERM\_POTENTIATION |  | 67 | -0.52 | -1.47 | 0.010 | 0.082 | 0.878 | 5988 | tags=46%, list=27%, signal=64% |
| 24 | KEGG\_FC\_GAMMA\_R\_MEDIATED\_PHAGOCYTOSIS |  | 92 | -0.50 | -1.47 | 0.006 | 0.083 | 0.890 | 5100 | tags=48%, list=23%, signal=62% |
| 25 | KEGG\_TIGHT\_JUNCTION |  | 127 | -0.47 | -1.46 | 0.004 | 0.085 | 0.907 | 4660 | tags=37%, list=21%, signal=47% |
| 26 | KEGG\_O\_GLYCAN\_BIOSYNTHESIS |  | 28 | -0.58 | -1.42 | 0.039 | 0.121 | 0.964 | 4136 | tags=39%, list=19%, signal=48% |
| 27 | KEGG\_JAK\_STAT\_SIGNALING\_PATHWAY |  | 141 | -0.45 | -1.42 | 0.008 | 0.120 | 0.966 | 5093 | tags=35%, list=23%, signal=46% |
| 28 | KEGG\_APOPTOSIS |  | 80 | -0.48 | -1.41 | 0.026 | 0.126 | 0.972 | 6068 | tags=52%, list=28%, signal=72% |
| 29 | KEGG\_MAPK\_SIGNALING\_PATHWAY |  | 258 | -0.42 | -1.40 | 0.001 | 0.132 | 0.983 | 5146 | tags=39%, list=24%, signal=51% |
| 30 | KEGG\_GNRH\_SIGNALING\_PATHWAY |  | 95 | -0.47 | -1.39 | 0.017 | 0.131 | 0.984 | 5052 | tags=43%, list=23%, signal=56% |
| 31 | KEGG\_RENAL\_CELL\_CARCINOMA |  | 66 | -0.49 | -1.39 | 0.022 | 0.133 | 0.985 | 5762 | tags=55%, list=26%, signal=74% |
| 32 | KEGG\_NEUROTROPHIN\_SIGNALING\_PATHWAY |  | 118 | -0.45 | -1.38 | 0.018 | 0.140 | 0.986 | 5721 | tags=53%, list=26%, signal=71% |
| 33 | KEGG\_NOTCH\_SIGNALING\_PATHWAY |  | 47 | -0.52 | -1.37 | 0.062 | 0.149 | 0.992 | 5462 | tags=53%, list=25%, signal=71% |
| 34 | KEGG\_GAP\_JUNCTION |  | 81 | -0.47 | -1.36 | 0.018 | 0.154 | 0.994 | 4626 | tags=37%, list=21%, signal=47% |
| 35 | KEGG\_VASCULAR\_SMOOTH\_MUSCLE\_CONTRACTION |  | 104 | -0.45 | -1.35 | 0.028 | 0.155 | 0.995 | 5052 | tags=42%, list=23%, signal=55% |
| 36 | KEGG\_MELANOGENESIS |  | 96 | -0.45 | -1.35 | 0.026 | 0.156 | 0.997 | 5104 | tags=38%, list=23%, signal=49% |
| 37 | KEGG\_CHRONIC\_MYELOID\_LEUKEMIA |  | 72 | -0.48 | -1.35 | 0.051 | 0.155 | 0.998 | 5141 | tags=56%, list=24%, signal=72% |
| 38 | KEGG\_STARCH\_AND\_SUCROSE\_METABOLISM |  | 32 | -0.54 | -1.35 | 0.074 | 0.152 | 0.998 | 5046 | tags=34%, list=23%, signal=45% |
| 39 | KEGG\_T\_CELL\_RECEPTOR\_SIGNALING\_PATHWAY |  | 106 | -0.45 | -1.34 | 0.022 | 0.157 | 0.998 | 5719 | tags=40%, list=26%, signal=53% |
| 40 | KEGG\_MELANOMA |  | 69 | -0.47 | -1.34 | 0.051 | 0.154 | 0.999 | 5141 | tags=45%, list=24%, signal=59% |
| 41 | KEGG\_PANCREATIC\_CANCER |  | 69 | -0.47 | -1.32 | 0.060 | 0.170 | 1.000 | 6108 | tags=58%, list=28%, signal=80% |
| 42 | KEGG\_GLYCOSAMINOGLYCAN\_DEGRADATION |  | 21 | -0.58 | -1.32 | 0.101 | 0.170 | 1.000 | 3903 | tags=38%, list=18%, signal=46% |
| 43 | KEGG\_THYROID\_CANCER |  | 28 | -0.55 | -1.32 | 0.085 | 0.172 | 1.000 | 5775 | tags=50%, list=26%, signal=68% |
| 44 | KEGG\_GLIOMA |  | 62 | -0.47 | -1.30 | 0.079 | 0.191 | 1.000 | 5141 | tags=56%, list=24%, signal=74% |
| 45 | KEGG\_GLYCOSAMINOGLYCAN\_BIOSYNTHESIS\_HEPARAN\_SULFATE |  | 26 | -0.55 | -1.30 | 0.106 | 0.190 | 1.000 | 5636 | tags=46%, list=26%, signal=62% |
| 46 | KEGG\_HEDGEHOG\_SIGNALING\_PATHWAY |  | 54 | -0.47 | -1.29 | 0.086 | 0.198 | 1.000 | 5234 | tags=35%, list=24%, signal=46% |
| 47 | KEGG\_TASTE\_TRANSDUCTION |  | 27 | -0.52 | -1.26 | 0.133 | 0.235 | 1.000 | 2424 | tags=30%, list=11%, signal=33% |
| 48 | KEGG\_STEROID\_HORMONE\_BIOSYNTHESIS |  | 32 | -0.51 | -1.26 | 0.127 | 0.230 | 1.000 | 2111 | tags=16%, list=10%, signal=17% |
| 49 | KEGG\_MTOR\_SIGNALING\_PATHWAY |  | 51 | -0.47 | -1.26 | 0.088 | 0.232 | 1.000 | 5230 | tags=53%, list=24%, signal=69% |
| 50 | KEGG\_UBIQUITIN\_MEDIATED\_PROTEOLYSIS |  | 125 | -0.40 | -1.26 | 0.063 | 0.234 | 1.000 | 5296 | tags=43%, list=24%, signal=57% |
| 51 | KEGG\_CALCIUM\_SIGNALING\_PATHWAY |  | 172 | -0.39 | -1.25 | 0.046 | 0.238 | 1.000 | 3731 | tags=24%, list=17%, signal=29% |
| 52 | KEGG\_LONG\_TERM\_DEPRESSION |  | 66 | -0.43 | -1.23 | 0.126 | 0.274 | 1.000 | 5643 | tags=42%, list=26%, signal=57% |
| 53 | KEGG\_LEUKOCYTE\_TRANSENDOTHELIAL\_MIGRATION |  | 113 | -0.40 | -1.21 | 0.107 | 0.299 | 1.000 | 5705 | tags=43%, list=26%, signal=58% |
| 54 | KEGG\_FC\_EPSILON\_RI\_SIGNALING\_PATHWAY |  | 77 | -0.42 | -1.21 | 0.117 | 0.299 | 1.000 | 5832 | tags=47%, list=27%, signal=64% |
| 55 | KEGG\_COLORECTAL\_CANCER |  | 61 | -0.43 | -1.20 | 0.157 | 0.305 | 1.000 | 5104 | tags=51%, list=23%, signal=66% |
| 56 | KEGG\_NEUROACTIVE\_LIGAND\_RECEPTOR\_INTERACTION |  | 260 | -0.35 | -1.16 | 0.108 | 0.388 | 1.000 | 4572 | tags=15%, list=21%, signal=18% |
| 57 | KEGG\_PROGESTERONE\_MEDIATED\_OOCYTE\_MATURATION |  | 82 | -0.39 | -1.13 | 0.215 | 0.440 | 1.000 | 5988 | tags=48%, list=27%, signal=65% |
| 58 | KEGG\_COMPLEMENT\_AND\_COAGULATION\_CASCADES |  | 61 | -0.41 | -1.13 | 0.243 | 0.433 | 1.000 | 5625 | tags=36%, list=26%, signal=48% |
| 59 | KEGG\_CELL\_ADHESION\_MOLECULES\_CAMS |  | 107 | -0.37 | -1.13 | 0.196 | 0.442 | 1.000 | 5920 | tags=34%, list=27%, signal=46% |
| 60 | KEGG\_N\_GLYCAN\_BIOSYNTHESIS |  | 44 | -0.42 | -1.11 | 0.291 | 0.488 | 1.000 | 5328 | tags=41%, list=24%, signal=54% |
| 61 | KEGG\_RIG\_I\_LIKE\_RECEPTOR\_SIGNALING\_PATHWAY |  | 63 | -0.40 | -1.10 | 0.275 | 0.495 | 1.000 | 6416 | tags=41%, list=29%, signal=58% |
| 62 | KEGG\_ADIPOCYTOKINE\_SIGNALING\_PATHWAY |  | 66 | -0.38 | -1.09 | 0.272 | 0.520 | 1.000 | 5093 | tags=42%, list=23%, signal=55% |
| 63 | KEGG\_BLADDER\_CANCER |  | 39 | -0.42 | -1.07 | 0.348 | 0.559 | 1.000 | 5141 | tags=41%, list=24%, signal=54% |
| 64 | KEGG\_RENIN\_ANGIOTENSIN\_SYSTEM |  | 15 | -0.51 | -1.07 | 0.381 | 0.555 | 1.000 | 5603 | tags=33%, list=26%, signal=45% |
| 65 | KEGG\_LYSINE\_DEGRADATION |  | 39 | -0.41 | -1.07 | 0.349 | 0.552 | 1.000 | 4959 | tags=44%, list=23%, signal=56% |
| 66 | KEGG\_GLYCEROPHOSPHOLIPID\_METABOLISM |  | 73 | -0.37 | -1.06 | 0.352 | 0.553 | 1.000 | 5506 | tags=45%, list=25%, signal=60% |
| 67 | KEGG\_NATURAL\_KILLER\_CELL\_MEDIATED\_CYTOTOXICITY |  | 104 | -0.36 | -1.06 | 0.344 | 0.546 | 1.000 | 4854 | tags=28%, list=22%, signal=36% |
| 68 | KEGG\_TOLL\_LIKE\_RECEPTOR\_SIGNALING\_PATHWAY |  | 96 | -0.36 | -1.06 | 0.343 | 0.545 | 1.000 | 6068 | tags=44%, list=28%, signal=60% |
| 69 | KEGG\_GLYCOSYLPHOSPHATIDYLINOSITOL\_GPI\_ANCHOR\_BIOSYNTHESIS |  | 24 | -0.45 | -1.06 | 0.394 | 0.539 | 1.000 | 5184 | tags=50%, list=24%, signal=65% |
| 70 | KEGG\_HYPERTROPHIC\_CARDIOMYOPATHY\_HCM |  | 83 | -0.35 | -1.02 | 0.408 | 0.632 | 1.000 | 4484 | tags=24%, list=20%, signal=30% |
| 71 | KEGG\_ALDOSTERONE\_REGULATED\_SODIUM\_REABSORPTION |  | 41 | -0.39 | -1.01 | 0.445 | 0.649 | 1.000 | 3699 | tags=37%, list=17%, signal=44% |
| 72 | KEGG\_VIRAL\_MYOCARDITIS |  | 50 | -0.37 | -1.00 | 0.462 | 0.673 | 1.000 | 5705 | tags=42%, list=26%, signal=57% |
| 73 | KEGG\_DILATED\_CARDIOMYOPATHY |  | 89 | -0.34 | -0.99 | 0.470 | 0.690 | 1.000 | 4484 | tags=21%, list=20%, signal=27% |
| 74 | KEGG\_P53\_SIGNALING\_PATHWAY |  | 62 | -0.35 | -0.98 | 0.498 | 0.703 | 1.000 | 4535 | tags=32%, list=21%, signal=41% |
| 75 | KEGG\_VASOPRESSIN\_REGULATED\_WATER\_REABSORPTION |  | 42 | -0.37 | -0.97 | 0.519 | 0.728 | 1.000 | 4063 | tags=29%, list=19%, signal=35% |
| 76 | KEGG\_VEGF\_SIGNALING\_PATHWAY |  | 74 | -0.33 | -0.94 | 0.583 | 0.800 | 1.000 | 5643 | tags=42%, list=26%, signal=56% |
| 77 | KEGG\_GLYCOSAMINOGLYCAN\_BIOSYNTHESIS\_CHONDROITIN\_SULFATE |  | 22 | -0.40 | -0.93 | 0.569 | 0.818 | 1.000 | 4965 | tags=36%, list=23%, signal=47% |
| 78 | KEGG\_MATURITY\_ONSET\_DIABETES\_OF\_THE\_YOUNG |  | 24 | -0.39 | -0.92 | 0.581 | 0.841 | 1.000 | 4475 | tags=17%, list=20%, signal=21% |
| 79 | KEGG\_CHEMOKINE\_SIGNALING\_PATHWAY |  | 168 | -0.29 | -0.92 | 0.681 | 0.833 | 1.000 | 5093 | tags=33%, list=23%, signal=42% |
| 80 | KEGG\_GRAFT\_VERSUS\_HOST\_DISEASE |  | 16 | -0.42 | -0.91 | 0.589 | 0.853 | 1.000 | 2665 | tags=6%, list=12%, signal=7% |
| 81 | KEGG\_ALPHA\_LINOLENIC\_ACID\_METABOLISM |  | 17 | -0.41 | -0.91 | 0.603 | 0.844 | 1.000 | 5870 | tags=53%, list=27%, signal=72% |
| 82 | KEGG\_BETA\_ALANINE\_METABOLISM |  | 22 | -0.39 | -0.89 | 0.643 | 0.864 | 1.000 | 5546 | tags=27%, list=25%, signal=36% |
| 83 | KEGG\_CELL\_CYCLE |  | 121 | -0.28 | -0.86 | 0.818 | 0.940 | 1.000 | 5811 | tags=36%, list=27%, signal=48% |
| 84 | KEGG\_PATHOGENIC\_ESCHERICHIA\_COLI\_INFECTION |  | 50 | -0.32 | -0.86 | 0.712 | 0.931 | 1.000 | 5932 | tags=36%, list=27%, signal=49% |
| 85 | KEGG\_OOCYTE\_MEIOSIS |  | 104 | -0.29 | -0.85 | 0.809 | 0.939 | 1.000 | 5811 | tags=36%, list=27%, signal=48% |
| 86 | KEGG\_B\_CELL\_RECEPTOR\_SIGNALING\_PATHWAY |  | 74 | -0.29 | -0.84 | 0.788 | 0.947 | 1.000 | 5093 | tags=35%, list=23%, signal=46% |
| 87 | KEGG\_GLYCEROLIPID\_METABOLISM |  | 47 | -0.31 | -0.84 | 0.763 | 0.951 | 1.000 | 4761 | tags=30%, list=22%, signal=38% |
| 88 | KEGG\_GLYCOSPHINGOLIPID\_BIOSYNTHESIS\_GANGLIO\_SERIES |  | 15 | -0.39 | -0.83 | 0.706 | 0.959 | 1.000 | 6871 | tags=47%, list=31%, signal=68% |
| 89 | KEGG\_LYSOSOME |  | 118 | -0.26 | -0.78 | 0.951 | 1.000 | 1.000 | 5945 | tags=41%, list=27%, signal=56% |
| 90 | KEGG\_NICOTINATE\_AND\_NICOTINAMIDE\_METABOLISM |  | 23 | -0.34 | -0.78 | 0.819 | 1.000 | 1.000 | 4205 | tags=26%, list=19%, signal=32% |
| 91 | KEGG\_PANTOTHENATE\_AND\_COA\_BIOSYNTHESIS |  | 15 | -0.36 | -0.77 | 0.785 | 1.000 | 1.000 | 2041 | tags=20%, list=9%, signal=22% |
| 92 | KEGG\_ETHER\_LIPID\_METABOLISM |  | 31 | -0.30 | -0.76 | 0.871 | 1.000 | 1.000 | 5506 | tags=39%, list=25%, signal=52% |
| 93 | KEGG\_PRION\_DISEASES |  | 33 | -0.30 | -0.76 | 0.897 | 1.000 | 1.000 | 3802 | tags=24%, list=17%, signal=29% |
| 94 | KEGG\_RETINOL\_METABOLISM |  | 32 | -0.29 | -0.73 | 0.917 | 1.000 | 1.000 | 3587 | tags=19%, list=16%, signal=22% |
| 95 | KEGG\_LINOLEIC\_ACID\_METABOLISM |  | 18 | -0.33 | -0.73 | 0.896 | 1.000 | 1.000 | 5506 | tags=39%, list=25%, signal=52% |
| 96 | KEGG\_PROPANOATE\_METABOLISM |  | 30 | -0.29 | -0.72 | 0.905 | 1.000 | 1.000 | 791 | tags=10%, list=4%, signal=10% |
| 97 | KEGG\_BASE\_EXCISION\_REPAIR |  | 33 | -0.28 | -0.70 | 0.941 | 1.000 | 1.000 | 3432 | tags=21%, list=16%, signal=25% |
| 98 | KEGG\_SPHINGOLIPID\_METABOLISM |  | 38 | -0.27 | -0.69 | 0.953 | 1.000 | 1.000 | 5885 | tags=37%, list=27%, signal=50% |
| 99 | KEGG\_BIOSYNTHESIS\_OF\_UNSATURATED\_FATTY\_ACIDS |  | 19 | -0.32 | -0.69 | 0.914 | 1.000 | 1.000 | 3194 | tags=21%, list=15%, signal=25% |
| 100 | KEGG\_LEISHMANIA\_INFECTION |  | 52 | -0.25 | -0.69 | 0.977 | 1.000 | 1.000 | 5691 | tags=33%, list=26%, signal=44% |
| 101 | KEGG\_TRYPTOPHAN\_METABOLISM |  | 39 | -0.26 | -0.69 | 0.969 | 1.000 | 1.000 | 4118 | tags=21%, list=19%, signal=25% |
| 102 | KEGG\_REGULATION\_OF\_AUTOPHAGY |  | 30 | -0.27 | -0.68 | 0.971 | 1.000 | 1.000 | 2258 | tags=13%, list=10%, signal=15% |
| 103 | KEGG\_CYSTEINE\_AND\_METHIONINE\_METABOLISM |  | 33 | -0.26 | -0.67 | 0.965 | 1.000 | 1.000 | 4782 | tags=27%, list=22%, signal=35% |
| 104 | KEGG\_GLYCOSPHINGOLIPID\_BIOSYNTHESIS\_LACTO\_AND\_NEOLACTO\_SERIES |  | 23 | -0.28 | -0.67 | 0.956 | 1.000 | 1.000 | 6420 | tags=39%, list=29%, signal=55% |
| 105 | KEGG\_DRUG\_METABOLISM\_OTHER\_ENZYMES |  | 29 | -0.27 | -0.66 | 0.976 | 0.994 | 1.000 | 2041 | tags=10%, list=9%, signal=11% |
| 106 | KEGG\_HOMOLOGOUS\_RECOMBINATION |  | 25 | -0.24 | -0.56 | 0.995 | 0.999 | 1.000 | 7113 | tags=60%, list=33%, signal=89% |
Table: Gene sets enriched in phenotype **MCAO (3 samples) **[plain text format]****
