# Supplementary material for: Regulation of microglia related neuroinflammation contributes to the protective effect of Gelsevirine on ischemic stroke
Source: Front Immunol. 2023 Mar 30;14:1164278. doi: 10.3389/fimmu.2023.1164278 (PMC10098192; doi:10.3389/fimmu.2023.1164278)
Supplement: Supplementary file 6 [file DataSheet_6.zip › fig 5 raw/fig 5-G raw/KEGG.Gsea.1649954846225/KEGG_ACUTE_MYELOID_LEUKEMIA.html]

Details for gene set KEGG\_ACUTE\_MYELOID\_LEUKEMIA[GSEA]

|  || Dataset | OGD\_DRUG\_DRUG.OGD\_FRUG.cls#Gs\_versus\_MCAO.OGD\_FRUG.cls#Gs\_versus\_MCAO\_repos |
| Phenotype | OGD\_FRUG.cls#Gs\_versus\_MCAO\_repos |
| Upregulated in class | MCAO |
| GeneSet | KEGG\_ACUTE\_MYELOID\_LEUKEMIA |
| Enrichment Score (ES) | -0.58726215 |
| Normalized Enrichment Score (NES) | -1.606015 |
| Nominal p-value | 0.001472754 |
| FDR q-value | 0.040534887 |
| FWER p-Value | 0.348 |
Table: GSEA Results Summary

  

Fig 1: Enrichment plot: KEGG\_ACUTE\_MYELOID\_LEUKEMIA      
 Profile of the Running ES Score & Positions of GeneSet Members on the Rank Ordered List

  

| SYMBOL | TITLE | RANK IN GENE LIST | RANK METRIC SCORE | RUNNING ES | CORE ENRICHMENT || 1 | PIK3CG | na | 1248 | 0.428 | -0.0275 | No |
| 2 | HRAS | na | 2716 | 0.268 | -0.0761 | No |
| 3 | EIF4EBP1 | na | 2735 | 0.266 | -0.0584 | No |
| 4 | BAD | na | 3658 | 0.189 | -0.0876 | No |
| 5 | NRAS | na | 4669 | 0.117 | -0.1258 | No |
| 6 | MAP2K2 | na | 4692 | 0.115 | -0.1188 | No |
| 7 | TCF7 | na | 4825 | 0.106 | -0.1174 | No |
| 8 | MAPK3 | na | 4918 | 0.100 | -0.1147 | No |
| 9 | MYC | na | 5587 | 0.064 | -0.1409 | No |
| 10 | GRB2 | na | 5726 | 0.057 | -0.1433 | No |
| 11 | KRAS | na | 6543 | 0.019 | -0.1794 | No |
| 12 | CEBPA | na | 7443 | 0.000 | -0.2206 | No |
| 13 | KIT | na | 8667 | 0.000 | -0.2767 | No |
| 14 | CCNA1 | na | 10605 | 0.000 | -0.3654 | No |
| 15 | ZBTB16 | na | 13418 | -0.006 | -0.4939 | No |
| 16 | FLT3 | na | 13729 | -0.013 | -0.5072 | No |
| 17 | IKBKG | na | 14695 | -0.050 | -0.5480 | No |
| 18 | MAP2K1 | na | 15179 | -0.079 | -0.5646 | No |
| 19 | RELA | na | 15584 | -0.103 | -0.5760 | No |
| 20 | RPS6KB2 | na | 15831 | -0.117 | -0.5791 | Yes |
| 21 | ARAF | na | 15889 | -0.121 | -0.5734 | Yes |
| 22 | PIM1 | na | 16160 | -0.138 | -0.5761 | Yes |
| 23 | MAPK1 | na | 16234 | -0.143 | -0.5695 | Yes |
| 24 | SPI1 | na | 16429 | -0.156 | -0.5676 | Yes |
| 25 | PIM2 | na | 16444 | -0.157 | -0.5573 | Yes |
| 26 | RARA | na | 16711 | -0.178 | -0.5572 | Yes |
| 27 | LEF1 | na | 16773 | -0.181 | -0.5475 | Yes |
| 28 | AKT2 | na | 16784 | -0.181 | -0.5353 | Yes |
| 29 | IKBKB | na | 16790 | -0.182 | -0.5229 | Yes |
| 30 | AKT1 | na | 16827 | -0.184 | -0.5118 | Yes |
| 31 | RUNX1 | na | 16837 | -0.185 | -0.4994 | Yes |
| 32 | TCF7L1 | na | 16939 | -0.192 | -0.4907 | Yes |
| 33 | RAF1 | na | 17251 | -0.212 | -0.4902 | Yes |
| 34 | RPS6KB1 | na | 17275 | -0.214 | -0.4764 | Yes |
| 35 | CCND1 | na | 17421 | -0.225 | -0.4674 | Yes |
| 36 | CHUK | na | 17623 | -0.238 | -0.4600 | Yes |
| 37 | PPARD | na | 17742 | -0.248 | -0.4483 | Yes |
| 38 | AKT3 | na | 17899 | -0.261 | -0.4373 | Yes |
| 39 | PIK3CD | na | 18197 | -0.284 | -0.4311 | Yes |
| 40 | PIK3R2 | na | 18436 | -0.299 | -0.4213 | Yes |
| 41 | PIK3CA | na | 18513 | -0.305 | -0.4036 | Yes |
| 42 | NFKB1 | na | 19213 | -0.371 | -0.4099 | Yes |
| 43 | PIK3R3 | na | 19336 | -0.380 | -0.3891 | Yes |
| 44 | STAT5A | na | 19487 | -0.396 | -0.3685 | Yes |
| 45 | TCF7L2 | na | 19504 | -0.397 | -0.3416 | Yes |
| 46 | PIK3R5 | na | 19509 | -0.397 | -0.3142 | Yes |
| 47 | SOS2 | na | 19626 | -0.409 | -0.2911 | Yes |
| 48 | PIK3CB | na | 20535 | -0.507 | -0.2976 | Yes |
| 49 | SOS1 | na | 20563 | -0.511 | -0.2633 | Yes |
| 50 | PML | na | 20576 | -0.512 | -0.2283 | Yes |
| 51 | STAT3 | na | 20708 | -0.529 | -0.1976 | Yes |
| 52 | BRAF | na | 20807 | -0.547 | -0.1641 | Yes |
| 53 | JUP | na | 20878 | -0.558 | -0.1286 | Yes |
| 54 | STAT5B | na | 20976 | -0.573 | -0.0933 | Yes |
| 55 | RUNX1T1 | na | 21146 | -0.607 | -0.0589 | Yes |
| 56 | MTOR | na | 21338 | -0.653 | -0.0223 | Yes |
| 57 | PIK3R1 | na | 21419 | -0.674 | 0.0209 | Yes |
Table: GSEA details [plain text format]

  

Fig 2: KEGG\_ACUTE\_MYELOID\_LEUKEMIA      
 Blue-Pink O' Gram in the Space of the Analyzed GeneSet

  

Fig 3: KEGG\_ACUTE\_MYELOID\_LEUKEMIA: Random ES distribution      
 Gene set null distribution of ES for **KEGG\_ACUTE\_MYELOID\_LEUKEMIA**
