# Supplementary material for: Regulation of microglia related neuroinflammation contributes to the protective effect of Gelsevirine on ischemic stroke
Source: Front Immunol. 2023 Mar 30;14:1164278. doi: 10.3389/fimmu.2023.1164278 (PMC10098192; doi:10.3389/fimmu.2023.1164278)
Supplement: Supplementary file 6 [file DataSheet_6.zip › fig 5 raw/fig 5-G raw/KEGG.Gsea.1649954846225/KEGG_ALZHEIMERS_DISEASE.html]

Details for gene set KEGG\_ALZHEIMERS\_DISEASE[GSEA]

|  || Dataset | OGD\_DRUG\_DRUG.OGD\_FRUG.cls#Gs\_versus\_MCAO.OGD\_FRUG.cls#Gs\_versus\_MCAO\_repos |
| Phenotype | OGD\_FRUG.cls#Gs\_versus\_MCAO\_repos |
| Upregulated in class | Gs |
| GeneSet | KEGG\_ALZHEIMERS\_DISEASE |
| Enrichment Score (ES) | 0.49565524 |
| Normalized Enrichment Score (NES) | 1.7056166 |
| Nominal p-value | 0.0 |
| FDR q-value | 0.023194745 |
| FWER p-Value | 0.147 |
Table: GSEA Results Summary

  

Fig 1: Enrichment plot: KEGG\_ALZHEIMERS\_DISEASE      
 Profile of the Running ES Score & Positions of GeneSet Members on the Rank Ordered List

  

| SYMBOL | TITLE | RANK IN GENE LIST | RANK METRIC SCORE | RUNNING ES | CORE ENRICHMENT || 1 | COX6A2 | na | 162 | 0.836 | 0.0130 | Yes |
| 2 | UQCRB | na | 430 | 0.620 | 0.0158 | Yes |
| 3 | COX6C | na | 440 | 0.616 | 0.0304 | Yes |
| 4 | COX7A2 | na | 449 | 0.612 | 0.0450 | Yes |
| 5 | COX5A | na | 462 | 0.602 | 0.0591 | Yes |
| 6 | NDUFA4 | na | 469 | 0.600 | 0.0735 | Yes |
| 7 | NDUFC1 | na | 493 | 0.590 | 0.0868 | Yes |
| 8 | UQCRH | na | 503 | 0.588 | 0.1008 | Yes |
| 9 | NDUFA2 | na | 510 | 0.586 | 0.1148 | Yes |
| 10 | NDUFB6 | na | 567 | 0.565 | 0.1260 | Yes |
| 11 | NOS1 | na | 617 | 0.549 | 0.1371 | Yes |
| 12 | COX6B1 | na | 653 | 0.538 | 0.1487 | Yes |
| 13 | NDUFA1 | na | 663 | 0.536 | 0.1613 | Yes |
| 14 | NDUFS4 | na | 674 | 0.532 | 0.1738 | Yes |
| 15 | NDUFA4L2 | na | 786 | 0.501 | 0.1809 | Yes |
| 16 | NDUFC2 | na | 793 | 0.500 | 0.1929 | Yes |
| 17 | UQCR10 | na | 800 | 0.498 | 0.2047 | Yes |
| 18 | NDUFB5 | na | 819 | 0.494 | 0.2160 | Yes |
| 19 | COX7B | na | 859 | 0.484 | 0.2260 | Yes |
| 20 | HSD17B10 | na | 996 | 0.466 | 0.2311 | Yes |
| 21 | NDUFA5 | na | 1040 | 0.459 | 0.2403 | Yes |
| 22 | PSENEN | na | 1097 | 0.449 | 0.2487 | Yes |
| 23 | COX7C | na | 1133 | 0.444 | 0.2579 | Yes |
| 24 | NDUFB3 | na | 1166 | 0.439 | 0.2672 | Yes |
| 25 | COX4I1 | na | 1200 | 0.434 | 0.2762 | Yes |
| 26 | NDUFS6 | na | 1280 | 0.424 | 0.2829 | Yes |
| 27 | COX6A1 | na | 1296 | 0.422 | 0.2925 | Yes |
| 28 | COX7A2L | na | 1310 | 0.419 | 0.3022 | Yes |
| 29 | CYCS | na | 1320 | 0.418 | 0.3119 | Yes |
| 30 | COX8A | na | 1327 | 0.417 | 0.3218 | Yes |
| 31 | NDUFS5 | na | 1350 | 0.415 | 0.3310 | Yes |
| 32 | CACNA1F | na | 1365 | 0.414 | 0.3404 | Yes |
| 33 | NDUFB10 | na | 1372 | 0.413 | 0.3502 | Yes |
| 34 | NDUFB9 | na | 1449 | 0.405 | 0.3566 | Yes |
| 35 | NDUFB4 | na | 1457 | 0.405 | 0.3662 | Yes |
| 36 | NDUFV2 | na | 1503 | 0.400 | 0.3739 | Yes |
| 37 | PSEN2 | na | 1521 | 0.398 | 0.3828 | Yes |
| 38 | GAPDH | na | 1541 | 0.395 | 0.3915 | Yes |
| 39 | NDUFS3 | na | 1637 | 0.382 | 0.3965 | Yes |
| 40 | NDUFB7 | na | 1789 | 0.363 | 0.3984 | Yes |
| 41 | UQCRQ | na | 1834 | 0.358 | 0.4051 | Yes |
| 42 | COX7A1 | na | 1863 | 0.354 | 0.4125 | Yes |
| 43 | NDUFA3 | na | 1881 | 0.352 | 0.4203 | Yes |
| 44 | NDUFB2 | na | 1907 | 0.348 | 0.4276 | Yes |
| 45 | NDUFA7 | na | 1971 | 0.341 | 0.4331 | Yes |
| 46 | NDUFA8 | na | 2185 | 0.318 | 0.4310 | Yes |
| 47 | UQCRFS1 | na | 2202 | 0.315 | 0.4380 | Yes |
| 48 | COX5B | na | 2237 | 0.311 | 0.4440 | Yes |
| 49 | APOE | na | 2372 | 0.297 | 0.4451 | Yes |
| 50 | CYC1 | na | 2402 | 0.296 | 0.4510 | Yes |
| 51 | NDUFAB1 | na | 2476 | 0.289 | 0.4546 | Yes |
| 52 | SDHD | na | 2574 | 0.281 | 0.4570 | Yes |
| 53 | NDUFA9 | na | 2575 | 0.281 | 0.4639 | Yes |
| 54 | UQCR11 | na | 2718 | 0.268 | 0.4639 | Yes |
| 55 | NDUFS7 | na | 2745 | 0.265 | 0.4691 | Yes |
| 56 | NDUFA10 | na | 2863 | 0.254 | 0.4700 | Yes |
| 57 | NDUFV3 | na | 2869 | 0.253 | 0.4759 | Yes |
| 58 | SDHC | na | 2894 | 0.251 | 0.4809 | Yes |
| 59 | UQCRC2 | na | 3080 | 0.235 | 0.4781 | Yes |
| 60 | NDUFS8 | na | 3269 | 0.217 | 0.4748 | Yes |
| 61 | NDUFA6 | na | 3303 | 0.214 | 0.4785 | Yes |
| 62 | CALM1 | na | 3305 | 0.214 | 0.4837 | Yes |
| 63 | SDHB | na | 3526 | 0.198 | 0.4784 | Yes |
| 64 | CALM2 | na | 3550 | 0.196 | 0.4821 | Yes |
| 65 | CDK5 | na | 3555 | 0.195 | 0.4867 | Yes |
| 66 | BAD | na | 3658 | 0.189 | 0.4866 | Yes |
| 67 | NDUFS2 | na | 3682 | 0.187 | 0.4901 | Yes |
| 68 | NDUFB8 | na | 3736 | 0.183 | 0.4921 | Yes |
| 69 | NAE1 | na | 3756 | 0.182 | 0.4957 | Yes |
| 70 | COX7B2 | na | 4007 | 0.163 | 0.4881 | No |
| 71 | LPL | na | 4214 | 0.148 | 0.4823 | No |
| 72 | NDUFV1 | na | 4574 | 0.124 | 0.4688 | No |
| 73 | PPP3CA | na | 4664 | 0.117 | 0.4675 | No |
| 74 | UQCRC1 | na | 4897 | 0.101 | 0.4593 | No |
| 75 | MAPK3 | na | 4918 | 0.100 | 0.4609 | No |
| 76 | CASP3 | na | 5009 | 0.095 | 0.4590 | No |
| 77 | TNF | na | 5838 | 0.050 | 0.4222 | No |
| 78 | APH1A | na | 5868 | 0.048 | 0.4220 | No |
| 79 | TNFRSF1A | na | 6041 | 0.039 | 0.4151 | No |
| 80 | CACNA1D | na | 6408 | 0.024 | 0.3988 | No |
| 81 | FAS | na | 6465 | 0.023 | 0.3968 | No |
| 82 | GRIN2A | na | 7559 | 0.000 | 0.3465 | No |
| 83 | GRIN2C | na | 7562 | 0.000 | 0.3464 | No |
| 84 | CHP2 | na | 7849 | 0.000 | 0.3332 | No |
| 85 | CALML3 | na | 8189 | 0.000 | 0.3177 | No |
| 86 | BACE2 | na | 8468 | 0.000 | 0.3049 | No |
| 87 | PPP3R2 | na | 9657 | 0.000 | 0.2502 | No |
| 88 | CACNA1S | na | 10978 | 0.000 | 0.1895 | No |
| 89 | COX8C | na | 11687 | 0.000 | 0.1569 | No |
| 90 | SNCA | na | 11941 | 0.000 | 0.1453 | No |
| 91 | MME | na | 13342 | -0.003 | 0.0809 | No |
| 92 | MAPT | na | 13499 | -0.007 | 0.0739 | No |
| 93 | COX6B2 | na | 13632 | -0.011 | 0.0681 | No |
| 94 | PPP3R1 | na | 13636 | -0.011 | 0.0682 | No |
| 95 | GRIN2B | na | 13653 | -0.011 | 0.0677 | No |
| 96 | PLCB2 | na | 13694 | -0.012 | 0.0662 | No |
| 97 | GRIN1 | na | 14095 | -0.023 | 0.0483 | No |
| 98 | IL1B | na | 14311 | -0.032 | 0.0392 | No |
| 99 | NDUFS1 | na | 14405 | -0.036 | 0.0358 | No |
| 100 | PPP3CC | na | 14499 | -0.041 | 0.0326 | No |
| 101 | COX4I2 | na | 14503 | -0.041 | 0.0334 | No |
| 102 | SDHA | na | 14573 | -0.044 | 0.0313 | No |
| 103 | CALM3 | na | 14729 | -0.052 | 0.0254 | No |
| 104 | BID | na | 14796 | -0.056 | 0.0238 | No |
| 105 | CASP8 | na | 15511 | -0.098 | -0.0067 | No |
| 106 | APBB1 | na | 15621 | -0.105 | -0.0091 | No |
| 107 | FADD | na | 15939 | -0.125 | -0.0207 | No |
| 108 | MAPK1 | na | 16234 | -0.143 | -0.0307 | No |
| 109 | ATP2A1 | na | 16311 | -0.148 | -0.0306 | No |
| 110 | CAPN2 | na | 16345 | -0.151 | -0.0284 | No |
| 111 | PSEN1 | na | 16462 | -0.159 | -0.0299 | No |
| 112 | ATF6 | na | 16486 | -0.161 | -0.0270 | No |
| 113 | CHP1 | na | 16560 | -0.166 | -0.0264 | No |
| 114 | PLCB4 | na | 16825 | -0.184 | -0.0340 | No |
| 115 | PPP3CB | na | 17182 | -0.208 | -0.0453 | No |
| 116 | IDE | na | 17370 | -0.221 | -0.0485 | No |
| 117 | NCSTN | na | 17428 | -0.226 | -0.0456 | No |
| 118 | GRIN2D | na | 17445 | -0.226 | -0.0409 | No |
| 119 | ATP2A3 | na | 18246 | -0.289 | -0.0706 | No |
| 120 | CAPN1 | na | 18291 | -0.290 | -0.0656 | No |
| 121 | ATP2A2 | na | 18364 | -0.295 | -0.0617 | No |
| 122 | EIF2AK3 | na | 18514 | -0.305 | -0.0611 | No |
| 123 | ADAM17 | na | 18595 | -0.311 | -0.0572 | No |
| 124 | APAF1 | na | 18709 | -0.321 | -0.0546 | No |
| 125 | CASP7 | na | 18712 | -0.322 | -0.0468 | No |
| 126 | GNAQ | na | 18773 | -0.327 | -0.0416 | No |
| 127 | PLCB3 | na | 18848 | -0.334 | -0.0368 | No |
| 128 | APP | na | 18882 | -0.338 | -0.0301 | No |
| 129 | CASP9 | na | 19243 | -0.373 | -0.0376 | No |
| 130 | ITPR2 | na | 19809 | -0.427 | -0.0531 | No |
| 131 | ADAM10 | na | 20004 | -0.445 | -0.0512 | No |
| 132 | ERN1 | na | 20049 | -0.450 | -0.0423 | No |
| 133 | GSK3B | na | 20096 | -0.455 | -0.0333 | No |
| 134 | CDK5R1 | na | 20194 | -0.466 | -0.0264 | No |
| 135 | ITPR3 | na | 20416 | -0.491 | -0.0245 | No |
| 136 | CACNA1C | na | 20587 | -0.514 | -0.0198 | No |
| 137 | RYR3 | na | 20981 | -0.574 | -0.0239 | No |
| 138 | ITPR1 | na | 20993 | -0.577 | -0.0103 | No |
| 139 | LRP1 | na | 21402 | -0.669 | -0.0128 | No |
| 140 | BACE1 | na | 21422 | -0.675 | 0.0028 | No |
| 141 | PLCB1 | na | 21572 | -0.736 | 0.0139 | No |
Table: GSEA details [plain text format]

  

Fig 2: KEGG\_ALZHEIMERS\_DISEASE      
 Blue-Pink O' Gram in the Space of the Analyzed GeneSet

  

Fig 3: KEGG\_ALZHEIMERS\_DISEASE: Random ES distribution      
 Gene set null distribution of ES for **KEGG\_ALZHEIMERS\_DISEASE**
