# Supplementary material for: Regulation of microglia related neuroinflammation contributes to the protective effect of Gelsevirine on ischemic stroke
Source: Front Immunol. 2023 Mar 30;14:1164278. doi: 10.3389/fimmu.2023.1164278 (PMC10098192; doi:10.3389/fimmu.2023.1164278)
Supplement: Supplementary file 6 [file DataSheet_6.zip › fig 5 raw/fig 5-G raw/KEGG.Gsea.1649954846225/KEGG_ANTIGEN_PROCESSING_AND_PRESENTATION.html]

Details for gene set KEGG\_ANTIGEN\_PROCESSING\_AND\_PRESENTATION[GSEA]

|  || Dataset | OGD\_DRUG\_DRUG.OGD\_FRUG.cls#Gs\_versus\_MCAO.OGD\_FRUG.cls#Gs\_versus\_MCAO\_repos |
| Phenotype | OGD\_FRUG.cls#Gs\_versus\_MCAO\_repos |
| Upregulated in class | Gs |
| GeneSet | KEGG\_ANTIGEN\_PROCESSING\_AND\_PRESENTATION |
| Enrichment Score (ES) | 0.55886865 |
| Normalized Enrichment Score (NES) | 1.6381273 |
| Nominal p-value | 0.005882353 |
| FDR q-value | 0.03664091 |
| FWER p-Value | 0.272 |
Table: GSEA Results Summary

  

Fig 1: Enrichment plot: KEGG\_ANTIGEN\_PROCESSING\_AND\_PRESENTATION      
 Profile of the Running ES Score & Positions of GeneSet Members on the Rank Ordered List

  

| SYMBOL | TITLE | RANK IN GENE LIST | RANK METRIC SCORE | RUNNING ES | CORE ENRICHMENT || 1 | HSPA5 | na | 265 | 0.727 | 0.0730 | Yes |
| 2 | PDIA3 | na | 1038 | 0.459 | 0.0913 | Yes |
| 3 | CTSL | na | 1068 | 0.455 | 0.1431 | Yes |
| 4 | CTSS | na | 1108 | 0.447 | 0.1936 | Yes |
| 5 | B2M | na | 1349 | 0.415 | 0.2312 | Yes |
| 6 | CD74 | na | 1409 | 0.410 | 0.2765 | Yes |
| 7 | RFXAP | na | 1620 | 0.384 | 0.3118 | Yes |
| 8 | HSPA1B | na | 1633 | 0.382 | 0.3560 | Yes |
| 9 | PSME2 | na | 1953 | 0.343 | 0.3815 | Yes |
| 10 | CALR | na | 2061 | 0.331 | 0.4154 | Yes |
| 11 | PSME1 | na | 2090 | 0.329 | 0.4526 | Yes |
| 12 | NFYB | na | 2154 | 0.321 | 0.4873 | Yes |
| 13 | IFI30 | na | 2266 | 0.308 | 0.5183 | Yes |
| 14 | CTSB | na | 2399 | 0.296 | 0.5468 | Yes |
| 15 | HSPA8 | na | 2800 | 0.259 | 0.5589 | Yes |
| 16 | LGMN | na | 3411 | 0.206 | 0.5550 | No |
| 17 | HSPA4 | na | 4740 | 0.112 | 0.5073 | No |
| 18 | HSP90AB1 | na | 5242 | 0.082 | 0.4940 | No |
| 19 | HSP90AA1 | na | 5244 | 0.082 | 0.5036 | No |
| 20 | RFXANK | na | 5643 | 0.061 | 0.4924 | No |
| 21 | HSPA1A | na | 5784 | 0.053 | 0.4922 | No |
| 22 | TAP2 | na | 6171 | 0.034 | 0.4784 | No |
| 23 | CANX | na | 6437 | 0.023 | 0.4690 | No |
| 24 | TAP1 | na | 7011 | 0.000 | 0.4428 | No |
| 25 | CD4 | na | 7312 | 0.000 | 0.4290 | No |
| 26 | IFNA13 | na | 8616 | 0.000 | 0.3693 | No |
| 27 | IFNA14 | na | 8617 | 0.000 | 0.3693 | No |
| 28 | IFNA16 | na | 8619 | 0.000 | 0.3693 | No |
| 29 | KLRC1 | na | 9136 | 0.000 | 0.3456 | No |
| 30 | KLRC2 | na | 9137 | 0.000 | 0.3456 | No |
| 31 | KLRC3 | na | 9138 | 0.000 | 0.3456 | No |
| 32 | KLRD1 | na | 9139 | 0.000 | 0.3456 | No |
| 33 | IFNA5 | na | 9402 | 0.000 | 0.3336 | No |
| 34 | IFNA7 | na | 9403 | 0.000 | 0.3336 | No |
| 35 | IFNA6 | na | 9404 | 0.000 | 0.3336 | No |
| 36 | IFNA1 | na | 9405 | 0.000 | 0.3336 | No |
| 37 | IFNA2 | na | 9406 | 0.000 | 0.3336 | No |
| 38 | KIR3DL1 | na | 11634 | 0.000 | 0.2316 | No |
| 39 | KIR3DL2 | na | 11635 | 0.000 | 0.2316 | No |
| 40 | CD8A | na | 12929 | 0.000 | 0.1723 | No |
| 41 | IFNA4 | na | 13482 | -0.007 | 0.1478 | No |
| 42 | NFYC | na | 13667 | -0.011 | 0.1407 | No |
| 43 | LTA | na | 13822 | -0.015 | 0.1354 | No |
| 44 | TAPBP | na | 14410 | -0.036 | 0.1127 | No |
| 45 | PSME3 | na | 16442 | -0.157 | 0.0381 | No |
| 46 | RFX5 | na | 16985 | -0.195 | 0.0360 | No |
| 47 | CREB1 | na | 18226 | -0.286 | 0.0127 | No |
| 48 | HSPA1L | na | 18395 | -0.297 | 0.0398 | No |
| 49 | CIITA | na | 18800 | -0.330 | 0.0598 | No |
| 50 | NFYA | na | 18885 | -0.339 | 0.0956 | No |
| 51 | HSPA2 | na | 19041 | -0.354 | 0.1299 | No |
Table: GSEA details [plain text format]

  

Fig 2: KEGG\_ANTIGEN\_PROCESSING\_AND\_PRESENTATION      
 Blue-Pink O' Gram in the Space of the Analyzed GeneSet

  

Fig 3: KEGG\_ANTIGEN\_PROCESSING\_AND\_PRESENTATION: Random ES distribution      
 Gene set null distribution of ES for **KEGG\_ANTIGEN\_PROCESSING\_AND\_PRESENTATION**
