# Supplementary material for: Regulation of microglia related neuroinflammation contributes to the protective effect of Gelsevirine on ischemic stroke
Source: Front Immunol. 2023 Mar 30;14:1164278. doi: 10.3389/fimmu.2023.1164278 (PMC10098192; doi:10.3389/fimmu.2023.1164278)
Supplement: Supplementary file 6 [file DataSheet_6.zip › fig 5 raw/fig 5-G raw/KEGG.Gsea.1649954846225/KEGG_ARRHYTHMOGENIC_RIGHT_VENTRICULAR_CARDIOMYOPATHY_ARVC.html]

Details for gene set KEGG\_ARRHYTHMOGENIC\_RIGHT\_VENTRICULAR\_CARDIOMYOPATHY\_ARVC[GSEA]

|  || Dataset | OGD\_DRUG\_DRUG.OGD\_FRUG.cls#Gs\_versus\_MCAO.OGD\_FRUG.cls#Gs\_versus\_MCAO\_repos |
| Phenotype | OGD\_FRUG.cls#Gs\_versus\_MCAO\_repos |
| Upregulated in class | MCAO |
| GeneSet | KEGG\_ARRHYTHMOGENIC\_RIGHT\_VENTRICULAR\_CARDIOMYOPATHY\_ARVC |
| Enrichment Score (ES) | -0.5313216 |
| Normalized Enrichment Score (NES) | -1.5302869 |
| Nominal p-value | 0.0058139535 |
| FDR q-value | 0.056728333 |
| FWER p-Value | 0.676 |
Table: GSEA Results Summary

  

Fig 1: Enrichment plot: KEGG\_ARRHYTHMOGENIC\_RIGHT\_VENTRICULAR\_CARDIOMYOPATHY\_ARVC      
 Profile of the Running ES Score & Positions of GeneSet Members on the Rank Ordered List

  

| SYMBOL | TITLE | RANK IN GENE LIST | RANK METRIC SCORE | RUNNING ES | CORE ENRICHMENT || 1 | SGCA | na | 374 | 0.644 | 0.0276 | No |
| 2 | CACNA1F | na | 1365 | 0.414 | 0.0109 | No |
| 3 | ITGB7 | na | 2467 | 0.290 | -0.0195 | No |
| 4 | ITGA2B | na | 2646 | 0.275 | -0.0086 | No |
| 5 | DES | na | 2929 | 0.248 | -0.0043 | No |
| 6 | CACNG4 | na | 4314 | 0.142 | -0.0579 | No |
| 7 | PKP2 | na | 4789 | 0.109 | -0.0721 | No |
| 8 | TCF7 | na | 4825 | 0.106 | -0.0663 | No |
| 9 | ITGB3 | na | 4968 | 0.097 | -0.0661 | No |
| 10 | EMD | na | 5183 | 0.086 | -0.0700 | No |
| 11 | ACTN2 | na | 5721 | 0.057 | -0.0906 | No |
| 12 | CACNG7 | na | 5797 | 0.052 | -0.0905 | No |
| 13 | CACNB2 | na | 6015 | 0.040 | -0.0976 | No |
| 14 | ITGA8 | na | 6033 | 0.039 | -0.0957 | No |
| 15 | ITGA11 | na | 6089 | 0.037 | -0.0957 | No |
| 16 | CACNG6 | na | 6184 | 0.033 | -0.0977 | No |
| 17 | DSG2 | na | 6237 | 0.031 | -0.0979 | No |
| 18 | ITGB8 | na | 6284 | 0.029 | -0.0980 | No |
| 19 | LMNA | na | 6395 | 0.025 | -0.1013 | No |
| 20 | CACNA1D | na | 6408 | 0.024 | -0.1002 | No |
| 21 | CTNNA2 | na | 6496 | 0.021 | -0.1027 | No |
| 22 | CACNG5 | na | 6556 | 0.018 | -0.1042 | No |
| 23 | ITGB6 | na | 6834 | 0.008 | -0.1163 | No |
| 24 | DMD | na | 7583 | 0.000 | -0.1506 | No |
| 25 | SLC8A1 | na | 8162 | 0.000 | -0.1771 | No |
| 26 | DSC2 | na | 8893 | 0.000 | -0.2106 | No |
| 27 | CACNG8 | na | 9762 | 0.000 | -0.2504 | No |
| 28 | CACNG1 | na | 9763 | 0.000 | -0.2504 | No |
| 29 | CACNG2 | na | 9764 | 0.000 | -0.2504 | No |
| 30 | CACNG3 | na | 9765 | 0.000 | -0.2504 | No |
| 31 | SGCG | na | 10702 | 0.000 | -0.2934 | No |
| 32 | CACNA1S | na | 10978 | 0.000 | -0.3060 | No |
| 33 | CACNA2D3 | na | 11582 | 0.000 | -0.3336 | No |
| 34 | CACNA2D2 | na | 11583 | 0.000 | -0.3336 | No |
| 35 | ITGA2 | na | 12406 | 0.000 | -0.3713 | No |
| 36 | RYR2 | na | 13400 | -0.005 | -0.4165 | No |
| 37 | CACNB4 | na | 13433 | -0.006 | -0.4176 | No |
| 38 | ITGA7 | na | 13508 | -0.007 | -0.4205 | No |
| 39 | ITGB5 | na | 13587 | -0.010 | -0.4234 | No |
| 40 | ITGA9 | na | 13709 | -0.013 | -0.4280 | No |
| 41 | ITGA4 | na | 13745 | -0.013 | -0.4288 | No |
| 42 | CACNA2D4 | na | 13824 | -0.015 | -0.4313 | No |
| 43 | ACTB | na | 13965 | -0.019 | -0.4364 | No |
| 44 | ITGA10 | na | 15634 | -0.105 | -0.5056 | No |
| 45 | CACNB1 | na | 15882 | -0.120 | -0.5086 | No |
| 46 | ACTG1 | na | 16172 | -0.139 | -0.5121 | No |
| 47 | SGCB | na | 16591 | -0.168 | -0.5196 | Yes |
| 48 | ITGB1 | na | 16661 | -0.174 | -0.5107 | Yes |
| 49 | LEF1 | na | 16773 | -0.181 | -0.5033 | Yes |
| 50 | SGCD | na | 16826 | -0.184 | -0.4929 | Yes |
| 51 | TCF7L1 | na | 16939 | -0.192 | -0.4847 | Yes |
| 52 | ACTN3 | na | 17217 | -0.210 | -0.4828 | Yes |
| 53 | CTNNA1 | na | 17378 | -0.222 | -0.4747 | Yes |
| 54 | ITGA5 | na | 17393 | -0.223 | -0.4599 | Yes |
| 55 | GJA1 | na | 17476 | -0.228 | -0.4478 | Yes |
| 56 | ITGB4 | na | 17565 | -0.234 | -0.4356 | Yes |
| 57 | CACNB3 | na | 17714 | -0.245 | -0.4253 | Yes |
| 58 | CTNNB1 | na | 18285 | -0.290 | -0.4313 | Yes |
| 59 | ATP2A2 | na | 18364 | -0.295 | -0.4144 | Yes |
| 60 | ACTN4 | na | 19490 | -0.396 | -0.4385 | Yes |
| 61 | TCF7L2 | na | 19504 | -0.397 | -0.4116 | Yes |
| 62 | CTNNA3 | na | 19975 | -0.442 | -0.4025 | Yes |
| 63 | LAMA2 | na | 20114 | -0.457 | -0.3771 | Yes |
| 64 | DSP | na | 20140 | -0.460 | -0.3463 | Yes |
| 65 | ITGA6 | na | 20245 | -0.471 | -0.3183 | Yes |
| 66 | CDH2 | na | 20561 | -0.510 | -0.2974 | Yes |
| 67 | CACNA1C | na | 20587 | -0.514 | -0.2628 | Yes |
| 68 | ACTN1 | na | 20635 | -0.521 | -0.2288 | Yes |
| 69 | ITGA3 | na | 20718 | -0.531 | -0.1957 | Yes |
| 70 | JUP | na | 20878 | -0.558 | -0.1643 | Yes |
| 71 | ITGAV | na | 21226 | -0.625 | -0.1368 | Yes |
| 72 | DAG1 | na | 21411 | -0.673 | -0.0985 | Yes |
| 73 | CACNA2D1 | na | 21475 | -0.696 | -0.0531 | Yes |
| 74 | ITGA1 | na | 21792 | -1.028 | 0.0038 | Yes |
Table: GSEA details [plain text format]

  

Fig 2: KEGG\_ARRHYTHMOGENIC\_RIGHT\_VENTRICULAR\_CARDIOMYOPATHY\_ARVC      
 Blue-Pink O' Gram in the Space of the Analyzed GeneSet

  

Fig 3: KEGG\_ARRHYTHMOGENIC\_RIGHT\_VENTRICULAR\_CARDIOMYOPATHY\_ARVC: Random ES distribution      
 Gene set null distribution of ES for **KEGG\_ARRHYTHMOGENIC\_RIGHT\_VENTRICULAR\_CARDIOMYOPATHY\_ARVC**
