# Supplementary material for: Regulation of microglia related neuroinflammation contributes to the protective effect of Gelsevirine on ischemic stroke
Source: Front Immunol. 2023 Mar 30;14:1164278. doi: 10.3389/fimmu.2023.1164278 (PMC10098192; doi:10.3389/fimmu.2023.1164278)
Supplement: Supplementary file 6 [file DataSheet_6.zip › fig 5 raw/fig 5-G raw/KEGG.Gsea.1649954846225/KEGG_AXON_GUIDANCE.html]

Details for gene set KEGG\_AXON\_GUIDANCE[GSEA]

|  || Dataset | OGD\_DRUG\_DRUG.OGD\_FRUG.cls#Gs\_versus\_MCAO.OGD\_FRUG.cls#Gs\_versus\_MCAO\_repos |
| Phenotype | OGD\_FRUG.cls#Gs\_versus\_MCAO\_repos |
| Upregulated in class | MCAO |
| GeneSet | KEGG\_AXON\_GUIDANCE |
| Enrichment Score (ES) | -0.54674774 |
| Normalized Enrichment Score (NES) | -1.6949329 |
| Nominal p-value | 0.0013227513 |
| FDR q-value | 0.014618517 |
| FWER p-Value | 0.106 |
Table: GSEA Results Summary

  

Fig 1: Enrichment plot: KEGG\_AXON\_GUIDANCE      
 Profile of the Running ES Score & Positions of GeneSet Members on the Rank Ordered List

  

| SYMBOL | TITLE | RANK IN GENE LIST | RANK METRIC SCORE | RUNNING ES | CORE ENRICHMENT || 1 | RAC3 | na | 194 | 0.799 | 0.0145 | No |
| 2 | ROBO2 | na | 351 | 0.657 | 0.0266 | No |
| 3 | DPYSL5 | na | 1245 | 0.428 | -0.0019 | No |
| 4 | EPHA1 | na | 1461 | 0.405 | 0.0001 | No |
| 5 | RHOD | na | 1911 | 0.348 | -0.0104 | No |
| 6 | RHOA | na | 1999 | 0.338 | -0.0045 | No |
| 7 | CFL1 | na | 2441 | 0.293 | -0.0162 | No |
| 8 | RND1 | na | 2642 | 0.276 | -0.0173 | No |
| 9 | HRAS | na | 2716 | 0.268 | -0.0128 | No |
| 10 | DPYSL2 | na | 2867 | 0.254 | -0.0122 | No |
| 11 | CDK5 | na | 3555 | 0.195 | -0.0381 | No |
| 12 | SEMA3G | na | 3559 | 0.195 | -0.0325 | No |
| 13 | PAK1 | na | 3623 | 0.191 | -0.0298 | No |
| 14 | RAC1 | na | 3821 | 0.178 | -0.0337 | No |
| 15 | CFL2 | na | 4282 | 0.144 | -0.0506 | No |
| 16 | GNAI1 | na | 4304 | 0.143 | -0.0474 | No |
| 17 | EFNA4 | na | 4374 | 0.138 | -0.0465 | No |
| 18 | PLXNB1 | na | 4460 | 0.132 | -0.0465 | No |
| 19 | SEMA4D | na | 4461 | 0.132 | -0.0427 | No |
| 20 | NGEF | na | 4492 | 0.129 | -0.0403 | No |
| 21 | PPP3CA | na | 4664 | 0.117 | -0.0447 | No |
| 22 | EFNA1 | na | 4668 | 0.117 | -0.0414 | No |
| 23 | NRAS | na | 4669 | 0.117 | -0.0380 | No |
| 24 | CDC42 | na | 4900 | 0.101 | -0.0456 | No |
| 25 | MAPK3 | na | 4918 | 0.100 | -0.0434 | No |
| 26 | NFATC2 | na | 5286 | 0.080 | -0.0580 | No |
| 27 | SLIT1 | na | 6158 | 0.034 | -0.0970 | No |
| 28 | SEMA5B | na | 6163 | 0.034 | -0.0962 | No |
| 29 | UNC5C | na | 6332 | 0.027 | -0.1031 | No |
| 30 | PLXNB3 | na | 6363 | 0.026 | -0.1038 | No |
| 31 | NTN3 | na | 6531 | 0.019 | -0.1109 | No |
| 32 | KRAS | na | 6543 | 0.019 | -0.1108 | No |
| 33 | SEMA3E | na | 6691 | 0.013 | -0.1172 | No |
| 34 | EPHA5 | na | 6794 | 0.010 | -0.1216 | No |
| 35 | NCK1 | na | 6802 | 0.009 | -0.1216 | No |
| 36 | FYN | na | 6884 | 0.006 | -0.1252 | No |
| 37 | SLIT3 | na | 7039 | 0.000 | -0.1323 | No |
| 38 | DCC | na | 7535 | 0.000 | -0.1550 | No |
| 39 | CHP2 | na | 7849 | 0.000 | -0.1694 | No |
| 40 | LRRC4C | na | 8338 | 0.000 | -0.1918 | No |
| 41 | SEMA6A | na | 8481 | 0.000 | -0.1984 | No |
| 42 | SEMA6D | na | 8482 | 0.000 | -0.1984 | No |
| 43 | SEMA3D | na | 8508 | 0.000 | -0.1995 | No |
| 44 | CXCR4 | na | 9111 | 0.000 | -0.2272 | No |
| 45 | PPP3R2 | na | 9657 | 0.000 | -0.2523 | No |
| 46 | UNC5A | na | 9845 | 0.000 | -0.2609 | No |
| 47 | UNC5D | na | 9847 | 0.000 | -0.2609 | No |
| 48 | RAC2 | na | 10018 | 0.000 | -0.2687 | No |
| 49 | EPHB1 | na | 10518 | 0.000 | -0.2917 | No |
| 50 | EPHA7 | na | 10519 | 0.000 | -0.2917 | No |
| 51 | EPHA6 | na | 10520 | 0.000 | -0.2917 | No |
| 52 | EPHA3 | na | 10529 | 0.000 | -0.2920 | No |
| 53 | ROBO1 | na | 12627 | 0.000 | -0.3885 | No |
| 54 | ABLIM2 | na | 13328 | -0.002 | -0.4206 | No |
| 55 | PPP3R1 | na | 13636 | -0.011 | -0.4344 | No |
| 56 | ABLIM3 | na | 13662 | -0.011 | -0.4352 | No |
| 57 | EPHA8 | na | 13752 | -0.013 | -0.4389 | No |
| 58 | CXCL12 | na | 13762 | -0.013 | -0.4390 | No |
| 59 | NTNG1 | na | 13794 | -0.014 | -0.4400 | No |
| 60 | SEMA4F | na | 14406 | -0.036 | -0.4670 | No |
| 61 | PPP3CC | na | 14499 | -0.041 | -0.4700 | No |
| 62 | NFATC4 | na | 15169 | -0.078 | -0.4985 | No |
| 63 | FES | na | 15319 | -0.087 | -0.5028 | No |
| 64 | MAPK1 | na | 16234 | -0.143 | -0.5406 | Yes |
| 65 | GNAI2 | na | 16257 | -0.144 | -0.5374 | Yes |
| 66 | EFNB3 | na | 16259 | -0.145 | -0.5332 | Yes |
| 67 | EFNA5 | na | 16303 | -0.147 | -0.5309 | Yes |
| 68 | EFNA2 | na | 16537 | -0.164 | -0.5368 | Yes |
| 69 | CHP1 | na | 16560 | -0.166 | -0.5329 | Yes |
| 70 | SEMA4A | na | 16623 | -0.171 | -0.5308 | Yes |
| 71 | PTK2 | na | 16640 | -0.173 | -0.5264 | Yes |
| 72 | ITGB1 | na | 16661 | -0.174 | -0.5223 | Yes |
| 73 | PPP3CB | na | 17182 | -0.208 | -0.5401 | Yes |
| 74 | SEMA3B | na | 17328 | -0.219 | -0.5403 | Yes |
| 75 | SEMA3C | na | 17330 | -0.219 | -0.5340 | Yes |
| 76 | LIMK2 | na | 17333 | -0.219 | -0.5276 | Yes |
| 77 | EPHA2 | na | 17359 | -0.220 | -0.5223 | Yes |
| 78 | EPHB3 | na | 17399 | -0.224 | -0.5175 | Yes |
| 79 | GNAI3 | na | 17440 | -0.226 | -0.5128 | Yes |
| 80 | PAK2 | na | 17619 | -0.238 | -0.5140 | Yes |
| 81 | PLXNA3 | na | 17886 | -0.260 | -0.5186 | Yes |
| 82 | PAK3 | na | 18126 | -0.279 | -0.5214 | Yes |
| 83 | EFNA3 | na | 18222 | -0.286 | -0.5174 | Yes |
| 84 | NTN4 | na | 18475 | -0.301 | -0.5201 | Yes |
| 85 | NTN1 | na | 18508 | -0.304 | -0.5127 | Yes |
| 86 | ROBO3 | na | 18528 | -0.306 | -0.5046 | Yes |
| 87 | RASA1 | na | 18622 | -0.313 | -0.4997 | Yes |
| 88 | SEMA6B | na | 18626 | -0.314 | -0.4906 | Yes |
| 89 | SEMA3A | na | 18654 | -0.317 | -0.4825 | Yes |
| 90 | SRGAP2 | na | 18839 | -0.333 | -0.4812 | Yes |
| 91 | SEMA4C | na | 18998 | -0.348 | -0.4783 | Yes |
| 92 | EPHB2 | na | 19034 | -0.353 | -0.4695 | Yes |
| 93 | SRGAP1 | na | 19204 | -0.370 | -0.4665 | Yes |
| 94 | RGS3 | na | 19239 | -0.373 | -0.4571 | Yes |
| 95 | EPHB4 | na | 19297 | -0.377 | -0.4487 | Yes |
| 96 | PAK4 | na | 19643 | -0.411 | -0.4525 | Yes |
| 97 | SEMA4B | na | 19764 | -0.422 | -0.4456 | Yes |
| 98 | NRP1 | na | 19765 | -0.422 | -0.4332 | Yes |
| 99 | ROCK1 | na | 19820 | -0.427 | -0.4232 | Yes |
| 100 | SEMA4G | na | 19884 | -0.434 | -0.4134 | Yes |
| 101 | GSK3B | na | 20096 | -0.455 | -0.4097 | Yes |
| 102 | EFNB2 | na | 20148 | -0.461 | -0.3985 | Yes |
| 103 | UNC5B | na | 20221 | -0.469 | -0.3881 | Yes |
| 104 | PAK6 | na | 20295 | -0.477 | -0.3775 | Yes |
| 105 | NCK2 | na | 20375 | -0.485 | -0.3669 | Yes |
| 106 | SLIT2 | na | 20562 | -0.511 | -0.3605 | Yes |
| 107 | ROCK2 | na | 20637 | -0.521 | -0.3486 | Yes |
| 108 | SEMA3F | na | 20673 | -0.525 | -0.3348 | Yes |
| 109 | MET | na | 20741 | -0.536 | -0.3222 | Yes |
| 110 | ABL1 | na | 20795 | -0.545 | -0.3086 | Yes |
| 111 | EFNB1 | na | 20812 | -0.548 | -0.2933 | Yes |
| 112 | LIMK1 | na | 20819 | -0.549 | -0.2775 | Yes |
| 113 | NFATC3 | na | 20856 | -0.555 | -0.2629 | Yes |
| 114 | NFATC1 | na | 20892 | -0.559 | -0.2481 | Yes |
| 115 | SEMA5A | na | 20899 | -0.560 | -0.2319 | Yes |
| 116 | SEMA6C | na | 21074 | -0.594 | -0.2225 | Yes |
| 117 | EPHB6 | na | 21082 | -0.596 | -0.2054 | Yes |
| 118 | PLXNB2 | na | 21175 | -0.614 | -0.1916 | Yes |
| 119 | EPHA4 | na | 21213 | -0.624 | -0.1750 | Yes |
| 120 | PLXNA1 | na | 21344 | -0.655 | -0.1618 | Yes |
| 121 | SEMA7A | na | 21359 | -0.657 | -0.1432 | Yes |
| 122 | NFAT5 | na | 21361 | -0.657 | -0.1239 | Yes |
| 123 | ARHGEF12 | na | 21366 | -0.658 | -0.1048 | Yes |
| 124 | ABLIM1 | na | 21453 | -0.688 | -0.0886 | Yes |
| 125 | L1CAM | na | 21577 | -0.739 | -0.0726 | Yes |
| 126 | PLXNA2 | na | 21627 | -0.770 | -0.0522 | Yes |
| 127 | SRGAP3 | na | 21789 | -1.013 | -0.0299 | Yes |
| 128 | PLXNC1 | na | 21830 | -1.154 | 0.0021 | Yes |
Table: GSEA details [plain text format]

  

Fig 2: KEGG\_AXON\_GUIDANCE      
 Blue-Pink O' Gram in the Space of the Analyzed GeneSet

  

Fig 3: KEGG\_AXON\_GUIDANCE: Random ES distribution      
 Gene set null distribution of ES for **KEGG\_AXON\_GUIDANCE**
