# Supplementary material for: Regulation of microglia related neuroinflammation contributes to the protective effect of Gelsevirine on ischemic stroke
Source: Front Immunol. 2023 Mar 30;14:1164278. doi: 10.3389/fimmu.2023.1164278 (PMC10098192; doi:10.3389/fimmu.2023.1164278)
Supplement: Supplementary file 6 [file DataSheet_6.zip › fig 5 raw/fig 5-G raw/KEGG.Gsea.1649954846225/KEGG_BASAL_CELL_CARCINOMA.html]

Details for gene set KEGG\_BASAL\_CELL\_CARCINOMA[GSEA]

|  || Dataset | OGD\_DRUG\_DRUG.OGD\_FRUG.cls#Gs\_versus\_MCAO.OGD\_FRUG.cls#Gs\_versus\_MCAO\_repos |
| Phenotype | OGD\_FRUG.cls#Gs\_versus\_MCAO\_repos |
| Upregulated in class | MCAO |
| GeneSet | KEGG\_BASAL\_CELL\_CARCINOMA |
| Enrichment Score (ES) | -0.63455683 |
| Normalized Enrichment Score (NES) | -1.7431902 |
| Nominal p-value | 0.0 |
| FDR q-value | 0.010627265 |
| FWER p-Value | 0.045 |
Table: GSEA Results Summary

  

Fig 1: Enrichment plot: KEGG\_BASAL\_CELL\_CARCINOMA      
 Profile of the Running ES Score & Positions of GeneSet Members on the Rank Ordered List

  

| SYMBOL | TITLE | RANK IN GENE LIST | RANK METRIC SCORE | RUNNING ES | CORE ENRICHMENT || 1 | WNT10B | na | 1647 | 0.381 | -0.0478 | No |
| 2 | PTCH2 | na | 1709 | 0.373 | -0.0235 | No |
| 3 | WNT2B | na | 2097 | 0.328 | -0.0174 | No |
| 4 | WNT4 | na | 2186 | 0.318 | 0.0016 | No |
| 5 | TCF7 | na | 4825 | 0.106 | -0.1115 | No |
| 6 | WNT7A | na | 5268 | 0.081 | -0.1259 | No |
| 7 | WNT11 | na | 5408 | 0.073 | -0.1270 | No |
| 8 | WNT3A | na | 6232 | 0.031 | -0.1624 | No |
| 9 | WNT3 | na | 6357 | 0.026 | -0.1662 | No |
| 10 | WNT7B | na | 6508 | 0.021 | -0.1716 | No |
| 11 | WNT16 | na | 6589 | 0.017 | -0.1740 | No |
| 12 | WNT8A | na | 7038 | 0.000 | -0.1945 | No |
| 13 | AXIN2 | na | 8241 | 0.000 | -0.2496 | No |
| 14 | BMP2 | na | 9002 | 0.000 | -0.2844 | No |
| 15 | WNT10A | na | 9033 | 0.000 | -0.2858 | No |
| 16 | FZD9 | na | 9034 | 0.000 | -0.2858 | No |
| 17 | FZD8 | na | 9035 | 0.000 | -0.2858 | No |
| 18 | WNT9B | na | 9177 | 0.000 | -0.2923 | No |
| 19 | WNT8B | na | 9179 | 0.000 | -0.2923 | No |
| 20 | WNT6 | na | 9610 | 0.000 | -0.3120 | No |
| 21 | WNT2 | na | 9613 | 0.000 | -0.3121 | No |
| 22 | SHH | na | 9875 | 0.000 | -0.3241 | No |
| 23 | HHIP | na | 10864 | 0.000 | -0.3693 | No |
| 24 | FZD10 | na | 11261 | 0.000 | -0.3875 | No |
| 25 | GLI2 | na | 12145 | 0.000 | -0.4279 | No |
| 26 | WNT9A | na | 13355 | -0.003 | -0.4831 | No |
| 27 | WNT1 | na | 15037 | -0.070 | -0.5550 | No |
| 28 | LEF1 | na | 16773 | -0.181 | -0.6214 | Yes |
| 29 | WNT5B | na | 16806 | -0.183 | -0.6096 | Yes |
| 30 | TCF7L1 | na | 16939 | -0.192 | -0.6017 | Yes |
| 31 | BMP4 | na | 17035 | -0.198 | -0.5917 | Yes |
| 32 | FZD2 | na | 17458 | -0.227 | -0.5946 | Yes |
| 33 | DVL2 | na | 17478 | -0.229 | -0.5788 | Yes |
| 34 | STK36 | na | 17807 | -0.254 | -0.5754 | Yes |
| 35 | FZD3 | na | 17855 | -0.258 | -0.5589 | Yes |
| 36 | DVL1 | na | 17888 | -0.261 | -0.5414 | Yes |
| 37 | AXIN1 | na | 18098 | -0.276 | -0.5309 | Yes |
| 38 | CTNNB1 | na | 18285 | -0.290 | -0.5184 | Yes |
| 39 | TCF7L2 | na | 19504 | -0.397 | -0.5454 | Yes |
| 40 | WNT5A | na | 19541 | -0.401 | -0.5179 | Yes |
| 41 | FZD1 | na | 19601 | -0.406 | -0.4911 | Yes |
| 42 | SMO | na | 19616 | -0.409 | -0.4621 | Yes |
| 43 | SUFU | na | 20032 | -0.448 | -0.4486 | Yes |
| 44 | GSK3B | na | 20096 | -0.455 | -0.4185 | Yes |
| 45 | FZD7 | na | 20408 | -0.489 | -0.3972 | Yes |
| 46 | DVL3 | na | 20890 | -0.559 | -0.3786 | Yes |
| 47 | FZD4 | na | 20970 | -0.572 | -0.3407 | Yes |
| 48 | APC | na | 21200 | -0.619 | -0.3062 | Yes |
| 49 | GLI3 | na | 21222 | -0.625 | -0.2618 | Yes |
| 50 | FZD5 | na | 21229 | -0.627 | -0.2166 | Yes |
| 51 | PTCH1 | na | 21269 | -0.636 | -0.1722 | Yes |
| 52 | APC2 | na | 21564 | -0.733 | -0.1324 | Yes |
| 53 | GLI1 | na | 21743 | -0.894 | -0.0757 | Yes |
| 54 | FZD6 | na | 21825 | -1.125 | 0.0023 | Yes |
Table: GSEA details [plain text format]

  

Fig 2: KEGG\_BASAL\_CELL\_CARCINOMA      
 Blue-Pink O' Gram in the Space of the Analyzed GeneSet

  

Fig 3: KEGG\_BASAL\_CELL\_CARCINOMA: Random ES distribution      
 Gene set null distribution of ES for **KEGG\_BASAL\_CELL\_CARCINOMA**
