# Supplementary material for: Regulation of microglia related neuroinflammation contributes to the protective effect of Gelsevirine on ischemic stroke
Source: Front Immunol. 2023 Mar 30;14:1164278. doi: 10.3389/fimmu.2023.1164278 (PMC10098192; doi:10.3389/fimmu.2023.1164278)
Supplement: Supplementary file 6 [file DataSheet_6.zip › fig 5 raw/fig 5-G raw/KEGG.Gsea.1649954846225/KEGG_CARDIAC_MUSCLE_CONTRACTION.html]

Details for gene set KEGG\_CARDIAC\_MUSCLE\_CONTRACTION[GSEA]

|  || Dataset | OGD\_DRUG\_DRUG.OGD\_FRUG.cls#Gs\_versus\_MCAO.OGD\_FRUG.cls#Gs\_versus\_MCAO\_repos |
| Phenotype | OGD\_FRUG.cls#Gs\_versus\_MCAO\_repos |
| Upregulated in class | Gs |
| GeneSet | KEGG\_CARDIAC\_MUSCLE\_CONTRACTION |
| Enrichment Score (ES) | 0.63691884 |
| Normalized Enrichment Score (NES) | 1.9817957 |
| Nominal p-value | 0.0 |
| FDR q-value | 3.8141027E-4 |
| FWER p-Value | 0.002 |
Table: GSEA Results Summary

  

Fig 1: Enrichment plot: KEGG\_CARDIAC\_MUSCLE\_CONTRACTION      
 Profile of the Running ES Score & Positions of GeneSet Members on the Rank Ordered List

  

| SYMBOL | TITLE | RANK IN GENE LIST | RANK METRIC SCORE | RUNNING ES | CORE ENRICHMENT || 1 | FXYD2 | na | 36 | 1.339 | 0.0728 | Yes |
| 2 | ATP1B1 | na | 108 | 0.965 | 0.1233 | Yes |
| 3 | COX6A2 | na | 162 | 0.836 | 0.1674 | Yes |
| 4 | TNNT2 | na | 197 | 0.797 | 0.2102 | Yes |
| 5 | UQCRB | na | 430 | 0.620 | 0.2340 | Yes |
| 6 | COX6C | na | 440 | 0.616 | 0.2679 | Yes |
| 7 | COX7A2 | na | 449 | 0.612 | 0.3016 | Yes |
| 8 | COX5A | na | 462 | 0.602 | 0.3345 | Yes |
| 9 | UQCRH | na | 503 | 0.588 | 0.3654 | Yes |
| 10 | COX6B1 | na | 653 | 0.538 | 0.3885 | Yes |
| 11 | UQCR10 | na | 800 | 0.498 | 0.4095 | Yes |
| 12 | COX7B | na | 859 | 0.484 | 0.4338 | Yes |
| 13 | COX7C | na | 1133 | 0.444 | 0.4460 | Yes |
| 14 | COX4I1 | na | 1200 | 0.434 | 0.4671 | Yes |
| 15 | COX6A1 | na | 1296 | 0.422 | 0.4862 | Yes |
| 16 | COX7A2L | na | 1310 | 0.419 | 0.5089 | Yes |
| 17 | COX8A | na | 1327 | 0.417 | 0.5314 | Yes |
| 18 | CACNA1F | na | 1365 | 0.414 | 0.5528 | Yes |
| 19 | TNNC1 | na | 1489 | 0.401 | 0.5694 | Yes |
| 20 | UQCRQ | na | 1834 | 0.358 | 0.5736 | Yes |
| 21 | COX7A1 | na | 1863 | 0.354 | 0.5920 | Yes |
| 22 | ATP1B3 | na | 2181 | 0.318 | 0.5952 | Yes |
| 23 | UQCRFS1 | na | 2202 | 0.315 | 0.6118 | Yes |
| 24 | COX5B | na | 2237 | 0.311 | 0.6275 | Yes |
| 25 | CYC1 | na | 2402 | 0.296 | 0.6365 | Yes |
| 26 | UQCR11 | na | 2718 | 0.268 | 0.6369 | Yes |
| 27 | UQCRC2 | na | 3080 | 0.235 | 0.6334 | No |
| 28 | TPM2 | na | 3334 | 0.212 | 0.6336 | No |
| 29 | COX7B2 | na | 4007 | 0.163 | 0.6119 | No |
| 30 | CACNG4 | na | 4314 | 0.142 | 0.6057 | No |
| 31 | ATP1A3 | na | 4321 | 0.142 | 0.6134 | No |
| 32 | UQCRC1 | na | 4897 | 0.101 | 0.5926 | No |
| 33 | TNNI3 | na | 5433 | 0.072 | 0.5721 | No |
| 34 | CACNG7 | na | 5797 | 0.052 | 0.5584 | No |
| 35 | TPM3 | na | 5818 | 0.051 | 0.5603 | No |
| 36 | CACNB2 | na | 6015 | 0.040 | 0.5535 | No |
| 37 | CACNG6 | na | 6184 | 0.033 | 0.5477 | No |
| 38 | CACNA1D | na | 6408 | 0.024 | 0.5388 | No |
| 39 | CACNG5 | na | 6556 | 0.018 | 0.5331 | No |
| 40 | ATP1A2 | na | 6662 | 0.015 | 0.5291 | No |
| 41 | SLC8A1 | na | 8162 | 0.000 | 0.4603 | No |
| 42 | ATP1B4 | na | 8776 | 0.000 | 0.4322 | No |
| 43 | ATP1B2 | na | 8777 | 0.000 | 0.4322 | No |
| 44 | ATP1A4 | na | 8804 | 0.000 | 0.4310 | No |
| 45 | CACNG8 | na | 9762 | 0.000 | 0.3871 | No |
| 46 | CACNG1 | na | 9763 | 0.000 | 0.3871 | No |
| 47 | CACNG2 | na | 9764 | 0.000 | 0.3871 | No |
| 48 | CACNG3 | na | 9765 | 0.000 | 0.3871 | No |
| 49 | CACNA1S | na | 10978 | 0.000 | 0.3315 | No |
| 50 | MYH7 | na | 11249 | 0.000 | 0.3191 | No |
| 51 | MYL2 | na | 11274 | 0.000 | 0.3180 | No |
| 52 | MYL3 | na | 11276 | 0.000 | 0.3180 | No |
| 53 | CACNA2D3 | na | 11582 | 0.000 | 0.3040 | No |
| 54 | CACNA2D2 | na | 11583 | 0.000 | 0.3040 | No |
| 55 | COX8C | na | 11687 | 0.000 | 0.2993 | No |
| 56 | RYR2 | na | 13400 | -0.005 | 0.2211 | No |
| 57 | CACNB4 | na | 13433 | -0.006 | 0.2199 | No |
| 58 | COX6B2 | na | 13632 | -0.011 | 0.2114 | No |
| 59 | MYH6 | na | 13792 | -0.014 | 0.2049 | No |
| 60 | CACNA2D4 | na | 13824 | -0.015 | 0.2044 | No |
| 61 | COX4I2 | na | 14503 | -0.041 | 0.1755 | No |
| 62 | TPM4 | na | 14555 | -0.043 | 0.1756 | No |
| 63 | TPM1 | na | 15133 | -0.076 | 0.1533 | No |
| 64 | ACTC1 | na | 15654 | -0.106 | 0.1354 | No |
| 65 | CACNB1 | na | 15882 | -0.120 | 0.1317 | No |
| 66 | SLC9A6 | na | 16636 | -0.172 | 0.1067 | No |
| 67 | ATP1A1 | na | 17371 | -0.222 | 0.0854 | No |
| 68 | CACNB3 | na | 17714 | -0.245 | 0.0833 | No |
| 69 | ATP2A2 | na | 18364 | -0.295 | 0.0700 | No |
| 70 | SLC9A1 | na | 19790 | -0.425 | 0.0282 | No |
| 71 | CACNA1C | na | 20587 | -0.514 | 0.0203 | No |
| 72 | CACNA2D1 | na | 21475 | -0.696 | 0.0183 | No |
Table: GSEA details [plain text format]

  

Fig 2: KEGG\_CARDIAC\_MUSCLE\_CONTRACTION      
 Blue-Pink O' Gram in the Space of the Analyzed GeneSet

  

Fig 3: KEGG\_CARDIAC\_MUSCLE\_CONTRACTION: Random ES distribution      
 Gene set null distribution of ES for **KEGG\_CARDIAC\_MUSCLE\_CONTRACTION**
