# Supplementary material for: Regulation of microglia related neuroinflammation contributes to the protective effect of Gelsevirine on ischemic stroke
Source: Front Immunol. 2023 Mar 30;14:1164278. doi: 10.3389/fimmu.2023.1164278 (PMC10098192; doi:10.3389/fimmu.2023.1164278)
Supplement: Supplementary file 6 [file DataSheet_6.zip › fig 5 raw/fig 5-G raw/KEGG.Gsea.1649954846225/KEGG_DNA_REPLICATION.html]

Details for gene set KEGG\_DNA\_REPLICATION[GSEA]

|  || Dataset | OGD\_DRUG\_DRUG.OGD\_FRUG.cls#Gs\_versus\_MCAO.OGD\_FRUG.cls#Gs\_versus\_MCAO\_repos |
| Phenotype | OGD\_FRUG.cls#Gs\_versus\_MCAO\_repos |
| Upregulated in class | Gs |
| GeneSet | KEGG\_DNA\_REPLICATION |
| Enrichment Score (ES) | 0.4620898 |
| Normalized Enrichment Score (NES) | 1.2720835 |
| Nominal p-value | 0.11290322 |
| FDR q-value | 0.32670867 |
| FWER p-Value | 0.997 |
Table: GSEA Results Summary

  

Fig 1: Enrichment plot: KEGG\_DNA\_REPLICATION      
 Profile of the Running ES Score & Positions of GeneSet Members on the Rank Ordered List

  

| SYMBOL | TITLE | RANK IN GENE LIST | RANK METRIC SCORE | RUNNING ES | CORE ENRICHMENT || 1 | RNASEH2B | na | 322 | 0.678 | 0.0679 | Yes |
| 2 | RFC4 | na | 748 | 0.511 | 0.1107 | Yes |
| 3 | RNASEH2A | na | 1025 | 0.461 | 0.1543 | Yes |
| 4 | RPA2 | na | 1039 | 0.459 | 0.2095 | Yes |
| 5 | RNASEH2C | na | 1211 | 0.433 | 0.2545 | Yes |
| 6 | SSBP1 | na | 1212 | 0.433 | 0.3073 | Yes |
| 7 | POLE4 | na | 1748 | 0.368 | 0.3276 | Yes |
| 8 | RPA3 | na | 2027 | 0.335 | 0.3557 | Yes |
| 9 | FEN1 | na | 2592 | 0.279 | 0.3638 | Yes |
| 10 | PCNA | na | 2828 | 0.257 | 0.3844 | Yes |
| 11 | POLD4 | na | 2954 | 0.245 | 0.4086 | Yes |
| 12 | RFC3 | na | 3147 | 0.229 | 0.4276 | Yes |
| 13 | POLE3 | na | 3398 | 0.207 | 0.4414 | Yes |
| 14 | PRIM1 | na | 3481 | 0.200 | 0.4621 | Yes |
| 15 | RFC2 | na | 4103 | 0.156 | 0.4527 | No |
| 16 | RNASEH1 | na | 4318 | 0.142 | 0.4602 | No |
| 17 | PRIM2 | na | 4961 | 0.098 | 0.4427 | No |
| 18 | MCM7 | na | 5251 | 0.082 | 0.4395 | No |
| 19 | POLD3 | na | 5345 | 0.077 | 0.4446 | No |
| 20 | MCM3 | na | 6087 | 0.037 | 0.4151 | No |
| 21 | RPA1 | na | 6886 | 0.006 | 0.3794 | No |
| 22 | MCM6 | na | 6932 | 0.004 | 0.3778 | No |
| 23 | POLA2 | na | 13523 | -0.007 | 0.0770 | No |
| 24 | POLE2 | na | 14842 | -0.058 | 0.0238 | No |
| 25 | POLD2 | na | 14951 | -0.065 | 0.0267 | No |
| 26 | LIG1 | na | 15381 | -0.091 | 0.0182 | No |
| 27 | RFC5 | na | 15928 | -0.123 | 0.0082 | No |
| 28 | MCM4 | na | 16168 | -0.139 | 0.0142 | No |
| 29 | MCM5 | na | 16233 | -0.143 | 0.0287 | No |
| 30 | POLD1 | na | 17195 | -0.209 | 0.0101 | No |
| 31 | MCM2 | na | 17947 | -0.266 | 0.0081 | No |
| 32 | RFC1 | na | 18103 | -0.277 | 0.0347 | No |
| 33 | POLA1 | na | 18783 | -0.329 | 0.0437 | No |
| 34 | DNA2 | na | 19062 | -0.355 | 0.0743 | No |
| 35 | POLE | na | 20020 | -0.447 | 0.0849 | No |
Table: GSEA details [plain text format]

  

Fig 2: KEGG\_DNA\_REPLICATION      
 Blue-Pink O' Gram in the Space of the Analyzed GeneSet

  

Fig 3: KEGG\_DNA\_REPLICATION: Random ES distribution      
 Gene set null distribution of ES for **KEGG\_DNA\_REPLICATION**
