# Supplementary material for: Regulation of microglia related neuroinflammation contributes to the protective effect of Gelsevirine on ischemic stroke
Source: Front Immunol. 2023 Mar 30;14:1164278. doi: 10.3389/fimmu.2023.1164278 (PMC10098192; doi:10.3389/fimmu.2023.1164278)
Supplement: Supplementary file 6 [file DataSheet_6.zip › fig 5 raw/fig 5-G raw/KEGG.Gsea.1649954846225/KEGG_DORSO_VENTRAL_AXIS_FORMATION.html]

Details for gene set KEGG\_DORSO\_VENTRAL\_AXIS\_FORMATION[GSEA]

|  || Dataset | OGD\_DRUG\_DRUG.OGD\_FRUG.cls#Gs\_versus\_MCAO.OGD\_FRUG.cls#Gs\_versus\_MCAO\_repos |
| Phenotype | OGD\_FRUG.cls#Gs\_versus\_MCAO\_repos |
| Upregulated in class | MCAO |
| GeneSet | KEGG\_DORSO\_VENTRAL\_AXIS\_FORMATION |
| Enrichment Score (ES) | -0.69964075 |
| Normalized Enrichment Score (NES) | -1.603904 |
| Nominal p-value | 0.009661836 |
| FDR q-value | 0.038044058 |
| FWER p-Value | 0.359 |
Table: GSEA Results Summary

  

Fig 1: Enrichment plot: KEGG\_DORSO\_VENTRAL\_AXIS\_FORMATION      
 Profile of the Running ES Score & Positions of GeneSet Members on the Rank Ordered List

  

| SYMBOL | TITLE | RANK IN GENE LIST | RANK METRIC SCORE | RUNNING ES | CORE ENRICHMENT || 1 | PIWIL4 | na | 487 | 0.593 | 0.0629 | No |
| 2 | SPIRE2 | na | 4424 | 0.134 | -0.0979 | No |
| 3 | MAPK3 | na | 4918 | 0.100 | -0.1061 | No |
| 4 | GRB2 | na | 5726 | 0.057 | -0.1349 | No |
| 5 | KRAS | na | 6543 | 0.019 | -0.1696 | No |
| 6 | PIWIL1 | na | 7170 | 0.000 | -0.1982 | No |
| 7 | NOTCH3 | na | 14072 | -0.023 | -0.5107 | No |
| 8 | ETS2 | na | 14586 | -0.045 | -0.5278 | No |
| 9 | MAP2K1 | na | 15179 | -0.079 | -0.5435 | No |
| 10 | MAPK1 | na | 16234 | -0.143 | -0.5712 | No |
| 11 | EGFR | na | 19043 | -0.354 | -0.6488 | Yes |
| 12 | SOS2 | na | 19626 | -0.409 | -0.6166 | Yes |
| 13 | SPIRE1 | na | 19627 | -0.409 | -0.5578 | Yes |
| 14 | CPEB1 | na | 19692 | -0.416 | -0.5010 | Yes |
| 15 | ETS1 | na | 19731 | -0.420 | -0.4424 | Yes |
| 16 | ETV6 | na | 19991 | -0.443 | -0.3905 | Yes |
| 17 | FMN2 | na | 20382 | -0.486 | -0.3386 | Yes |
| 18 | SOS1 | na | 20563 | -0.511 | -0.2734 | Yes |
| 19 | NOTCH1 | na | 20749 | -0.537 | -0.2048 | Yes |
| 20 | PIWIL2 | na | 20910 | -0.562 | -0.1313 | Yes |
| 21 | NOTCH4 | na | 20914 | -0.562 | -0.0507 | Yes |
| 22 | NOTCH2 | na | 21367 | -0.658 | 0.0232 | Yes |
Table: GSEA details [plain text format]

  

Fig 2: KEGG\_DORSO\_VENTRAL\_AXIS\_FORMATION      
 Blue-Pink O' Gram in the Space of the Analyzed GeneSet

  

Fig 3: KEGG\_DORSO\_VENTRAL\_AXIS\_FORMATION: Random ES distribution      
 Gene set null distribution of ES for **KEGG\_DORSO\_VENTRAL\_AXIS\_FORMATION**
