# Supplementary material for: Regulation of microglia related neuroinflammation contributes to the protective effect of Gelsevirine on ischemic stroke
Source: Front Immunol. 2023 Mar 30;14:1164278. doi: 10.3389/fimmu.2023.1164278 (PMC10098192; doi:10.3389/fimmu.2023.1164278)
Supplement: Supplementary file 6 [file DataSheet_6.zip › fig 5 raw/fig 5-G raw/KEGG.Gsea.1649954846225/KEGG_DRUG_METABOLISM_CYTOCHROME_P450.html]

Details for gene set KEGG\_DRUG\_METABOLISM\_CYTOCHROME\_P450[GSEA]

|  || Dataset | OGD\_DRUG\_DRUG.OGD\_FRUG.cls#Gs\_versus\_MCAO.OGD\_FRUG.cls#Gs\_versus\_MCAO\_repos |
| Phenotype | OGD\_FRUG.cls#Gs\_versus\_MCAO\_repos |
| Upregulated in class | Gs |
| GeneSet | KEGG\_DRUG\_METABOLISM\_CYTOCHROME\_P450 |
| Enrichment Score (ES) | 0.48082283 |
| Normalized Enrichment Score (NES) | 1.3952123 |
| Nominal p-value | 0.038567495 |
| FDR q-value | 0.20715715 |
| FWER p-Value | 0.914 |
Table: GSEA Results Summary

  

Fig 1: Enrichment plot: KEGG\_DRUG\_METABOLISM\_CYTOCHROME\_P450      
 Profile of the Running ES Score & Positions of GeneSet Members on the Rank Ordered List

  

| SYMBOL | TITLE | RANK IN GENE LIST | RANK METRIC SCORE | RUNNING ES | CORE ENRICHMENT || 1 | MGST1 | na | 682 | 0.531 | 0.0275 | Yes |
| 2 | GSTM5 | na | 917 | 0.475 | 0.0695 | Yes |
| 3 | MGST3 | na | 1032 | 0.460 | 0.1151 | Yes |
| 4 | GSTA4 | na | 1142 | 0.443 | 0.1592 | Yes |
| 5 | ADH5 | na | 1254 | 0.428 | 0.2015 | Yes |
| 6 | GSTO2 | na | 1295 | 0.422 | 0.2464 | Yes |
| 7 | FMO5 | na | 1498 | 0.400 | 0.2814 | Yes |
| 8 | GSTO1 | na | 1917 | 0.347 | 0.3007 | Yes |
| 9 | GSTP1 | na | 1927 | 0.346 | 0.3386 | Yes |
| 10 | GSTM2 | na | 2075 | 0.330 | 0.3684 | Yes |
| 11 | GSTA3 | na | 2150 | 0.321 | 0.4006 | Yes |
| 12 | ALDH3B2 | na | 2211 | 0.314 | 0.4327 | Yes |
| 13 | MGST2 | na | 2780 | 0.261 | 0.4356 | Yes |
| 14 | ALDH3B1 | na | 2884 | 0.251 | 0.4587 | Yes |
| 15 | GSTM1 | na | 2988 | 0.243 | 0.4808 | Yes |
| 16 | GSTZ1 | na | 3908 | 0.172 | 0.4578 | No |
| 17 | MAOA | na | 4152 | 0.153 | 0.4637 | No |
| 18 | GSTK1 | na | 5170 | 0.086 | 0.4266 | No |
| 19 | GSTA2 | na | 5397 | 0.074 | 0.4245 | No |
| 20 | FMO4 | na | 5578 | 0.064 | 0.4233 | No |
| 21 | ADH7 | na | 5804 | 0.052 | 0.4188 | No |
| 22 | ALDH3A1 | na | 6135 | 0.035 | 0.4076 | No |
| 23 | UGT1A10 | na | 6336 | 0.027 | 0.4014 | No |
| 24 | GSTM3 | na | 7079 | 0.000 | 0.3674 | No |
| 25 | CYP1A2 | na | 7814 | 0.000 | 0.3338 | No |
| 26 | ADH4 | na | 8140 | 0.000 | 0.3189 | No |
| 27 | ALDH1A3 | na | 11926 | 0.000 | 0.1456 | No |
| 28 | UGT2A3 | na | 12403 | 0.000 | 0.1238 | No |
| 29 | UGT2A1 | na | 12405 | 0.000 | 0.1237 | No |
| 30 | UGT1A9 | na | 12599 | 0.000 | 0.1149 | No |
| 31 | FMO3 | na | 13083 | 0.000 | 0.0928 | No |
| 32 | GSTA1 | na | 13344 | -0.003 | 0.0812 | No |
| 33 | CYP2E1 | na | 13590 | -0.010 | 0.0710 | No |
| 34 | AOX1 | na | 14290 | -0.031 | 0.0425 | No |
| 35 | GSTM4 | na | 14526 | -0.042 | 0.0363 | No |
| 36 | UGT1A5 | na | 15219 | -0.081 | 0.0136 | No |
| 37 | MAOB | na | 16182 | -0.140 | -0.0150 | No |
| 38 | FMO1 | na | 17389 | -0.223 | -0.0456 | No |
| 39 | FMO2 | na | 17482 | -0.229 | -0.0244 | No |
| 40 | GSTT2 | na | 18427 | -0.298 | -0.0346 | No |
| 41 | UGT1A1 | na | 21720 | -0.867 | -0.0894 | No |
| 42 | GSTT1 | na | 21731 | -0.871 | 0.0066 | No |
Table: GSEA details [plain text format]

  

Fig 2: KEGG\_DRUG\_METABOLISM\_CYTOCHROME\_P450      
 Blue-Pink O' Gram in the Space of the Analyzed GeneSet

  

Fig 3: KEGG\_DRUG\_METABOLISM\_CYTOCHROME\_P450: Random ES distribution      
 Gene set null distribution of ES for **KEGG\_DRUG\_METABOLISM\_CYTOCHROME\_P450**
