# Supplementary material for: Regulation of microglia related neuroinflammation contributes to the protective effect of Gelsevirine on ischemic stroke
Source: Front Immunol. 2023 Mar 30;14:1164278. doi: 10.3389/fimmu.2023.1164278 (PMC10098192; doi:10.3389/fimmu.2023.1164278)
Supplement: Supplementary file 6 [file DataSheet_6.zip › fig 5 raw/fig 5-G raw/KEGG.Gsea.1649954846225/KEGG_ECM_RECEPTOR_INTERACTION.html]

Details for gene set KEGG\_ECM\_RECEPTOR\_INTERACTION[GSEA]

|  || Dataset | OGD\_DRUG\_DRUG.OGD\_FRUG.cls#Gs\_versus\_MCAO.OGD\_FRUG.cls#Gs\_versus\_MCAO\_repos |
| Phenotype | OGD\_FRUG.cls#Gs\_versus\_MCAO\_repos |
| Upregulated in class | MCAO |
| GeneSet | KEGG\_ECM\_RECEPTOR\_INTERACTION |
| Enrichment Score (ES) | -0.587907 |
| Normalized Enrichment Score (NES) | -1.7196071 |
| Nominal p-value | 0.0 |
| FDR q-value | 0.012759207 |
| FWER p-Value | 0.067 |
Table: GSEA Results Summary

  

Fig 1: Enrichment plot: KEGG\_ECM\_RECEPTOR\_INTERACTION      
 Profile of the Running ES Score & Positions of GeneSet Members on the Rank Ordered List

  

| SYMBOL | TITLE | RANK IN GENE LIST | RANK METRIC SCORE | RUNNING ES | CORE ENRICHMENT || 1 | LAMC3 | na | 692 | 0.529 | -0.0096 | No |
| 2 | LAMB3 | na | 2281 | 0.306 | -0.0696 | No |
| 3 | ITGB7 | na | 2467 | 0.290 | -0.0660 | No |
| 4 | CD36 | na | 2486 | 0.287 | -0.0548 | No |
| 5 | LAMA3 | na | 2493 | 0.287 | -0.0430 | No |
| 6 | ITGA2B | na | 2646 | 0.275 | -0.0385 | No |
| 7 | CD47 | na | 2734 | 0.266 | -0.0313 | No |
| 8 | SPP1 | na | 3259 | 0.218 | -0.0462 | No |
| 9 | COL6A6 | na | 3636 | 0.190 | -0.0555 | No |
| 10 | LAMA4 | na | 3690 | 0.187 | -0.0501 | No |
| 11 | COL2A1 | na | 3863 | 0.176 | -0.0506 | No |
| 12 | GP9 | na | 4148 | 0.154 | -0.0572 | No |
| 13 | LAMC2 | na | 4333 | 0.141 | -0.0598 | No |
| 14 | VTN | na | 4384 | 0.138 | -0.0563 | No |
| 15 | COL11A1 | na | 4842 | 0.105 | -0.0728 | No |
| 16 | ITGB3 | na | 4968 | 0.097 | -0.0745 | No |
| 17 | COMP | na | 5235 | 0.083 | -0.0832 | No |
| 18 | ITGA8 | na | 6033 | 0.039 | -0.1182 | No |
| 19 | ITGA11 | na | 6089 | 0.037 | -0.1192 | No |
| 20 | THBS3 | na | 6218 | 0.032 | -0.1237 | No |
| 21 | ITGB8 | na | 6284 | 0.029 | -0.1255 | No |
| 22 | SDC2 | na | 6810 | 0.009 | -0.1492 | No |
| 23 | ITGB6 | na | 6834 | 0.008 | -0.1499 | No |
| 24 | SV2C | na | 7296 | 0.000 | -0.1711 | No |
| 25 | CHAD | na | 7760 | 0.000 | -0.1923 | No |
| 26 | GP1BA | na | 8764 | 0.000 | -0.2383 | No |
| 27 | IBSP | na | 9186 | 0.000 | -0.2577 | No |
| 28 | TNN | na | 10258 | 0.000 | -0.3068 | No |
| 29 | ITGA2 | na | 12406 | 0.000 | -0.4053 | No |
| 30 | THBS4 | na | 12698 | 0.000 | -0.4187 | No |
| 31 | TNR | na | 13505 | -0.007 | -0.4554 | No |
| 32 | ITGA7 | na | 13508 | -0.007 | -0.4552 | No |
| 33 | RELN | na | 13541 | -0.008 | -0.4563 | No |
| 34 | LAMA1 | na | 13573 | -0.009 | -0.4573 | No |
| 35 | ITGB5 | na | 13587 | -0.010 | -0.4575 | No |
| 36 | ITGA9 | na | 13709 | -0.013 | -0.4625 | No |
| 37 | ITGA4 | na | 13745 | -0.013 | -0.4636 | No |
| 38 | SV2B | na | 13788 | -0.014 | -0.4650 | No |
| 39 | GP6 | na | 13897 | -0.017 | -0.4692 | No |
| 40 | SDC4 | na | 13905 | -0.017 | -0.4688 | No |
| 41 | SV2A | na | 14686 | -0.050 | -0.5025 | No |
| 42 | ITGA10 | na | 15634 | -0.105 | -0.5416 | No |
| 43 | COL5A3 | na | 15648 | -0.106 | -0.5377 | No |
| 44 | SDC1 | na | 16516 | -0.163 | -0.5707 | No |
| 45 | ITGB1 | na | 16661 | -0.174 | -0.5700 | No |
| 46 | CD44 | na | 16720 | -0.178 | -0.5652 | No |
| 47 | VWF | na | 16951 | -0.193 | -0.5677 | No |
| 48 | ITGA5 | na | 17393 | -0.223 | -0.5786 | Yes |
| 49 | ITGB4 | na | 17565 | -0.234 | -0.5766 | Yes |
| 50 | COL11A2 | na | 17711 | -0.245 | -0.5730 | Yes |
| 51 | HMMR | na | 17908 | -0.262 | -0.5710 | Yes |
| 52 | COL5A2 | na | 18110 | -0.277 | -0.5685 | Yes |
| 53 | SDC3 | na | 18345 | -0.294 | -0.5670 | Yes |
| 54 | LAMB1 | na | 18455 | -0.300 | -0.5594 | Yes |
| 55 | COL3A1 | na | 18869 | -0.337 | -0.5643 | Yes |
| 56 | COL1A2 | na | 19087 | -0.358 | -0.5592 | Yes |
| 57 | THBS2 | na | 19115 | -0.362 | -0.5453 | Yes |
| 58 | LAMA5 | na | 19183 | -0.368 | -0.5329 | Yes |
| 59 | COL6A2 | na | 19189 | -0.369 | -0.5177 | Yes |
| 60 | COL1A1 | na | 19936 | -0.438 | -0.5336 | Yes |
| 61 | GP1BB | na | 19986 | -0.443 | -0.5173 | Yes |
| 62 | COL6A1 | na | 20104 | -0.456 | -0.5035 | Yes |
| 63 | LAMA2 | na | 20114 | -0.457 | -0.4848 | Yes |
| 64 | LAMB2 | na | 20135 | -0.460 | -0.4665 | Yes |
| 65 | ITGA6 | na | 20245 | -0.471 | -0.4517 | Yes |
| 66 | GP5 | na | 20697 | -0.529 | -0.4502 | Yes |
| 67 | ITGA3 | na | 20718 | -0.531 | -0.4289 | Yes |
| 68 | COL4A1 | na | 20907 | -0.561 | -0.4140 | Yes |
| 69 | COL5A1 | na | 21162 | -0.610 | -0.4001 | Yes |
| 70 | ITGAV | na | 21226 | -0.625 | -0.3768 | Yes |
| 71 | COL4A6 | na | 21228 | -0.627 | -0.3506 | Yes |
| 72 | TNC | na | 21238 | -0.628 | -0.3247 | Yes |
| 73 | LAMC1 | na | 21239 | -0.629 | -0.2983 | Yes |
| 74 | COL4A2 | na | 21287 | -0.639 | -0.2737 | Yes |
| 75 | AGRN | na | 21378 | -0.662 | -0.2501 | Yes |
| 76 | FN1 | na | 21407 | -0.672 | -0.2232 | Yes |
| 77 | DAG1 | na | 21411 | -0.673 | -0.1951 | Yes |
| 78 | COL6A3 | na | 21436 | -0.681 | -0.1677 | Yes |
| 79 | HSPG2 | na | 21606 | -0.760 | -0.1436 | Yes |
| 80 | THBS1 | na | 21664 | -0.806 | -0.1125 | Yes |
| 81 | COL4A4 | na | 21703 | -0.851 | -0.0785 | Yes |
| 82 | ITGA1 | na | 21792 | -1.028 | -0.0395 | Yes |
| 83 | TNXB | na | 21793 | -1.031 | 0.0038 | Yes |
Table: GSEA details [plain text format]

  

Fig 2: KEGG\_ECM\_RECEPTOR\_INTERACTION      
 Blue-Pink O' Gram in the Space of the Analyzed GeneSet

  

Fig 3: KEGG\_ECM\_RECEPTOR\_INTERACTION: Random ES distribution      
 Gene set null distribution of ES for **KEGG\_ECM\_RECEPTOR\_INTERACTION**
