# Supplementary material for: Regulation of microglia related neuroinflammation contributes to the protective effect of Gelsevirine on ischemic stroke
Source: Front Immunol. 2023 Mar 30;14:1164278. doi: 10.3389/fimmu.2023.1164278 (PMC10098192; doi:10.3389/fimmu.2023.1164278)
Supplement: Supplementary file 6 [file DataSheet_6.zip › fig 5 raw/fig 5-G raw/KEGG.Gsea.1649954846225/KEGG_ENDOCYTOSIS.html]

Details for gene set KEGG\_ENDOCYTOSIS[GSEA]

|  || Dataset | OGD\_DRUG\_DRUG.OGD\_FRUG.cls#Gs\_versus\_MCAO.OGD\_FRUG.cls#Gs\_versus\_MCAO\_repos |
| Phenotype | OGD\_FRUG.cls#Gs\_versus\_MCAO\_repos |
| Upregulated in class | MCAO |
| GeneSet | KEGG\_ENDOCYTOSIS |
| Enrichment Score (ES) | -0.48287547 |
| Normalized Enrichment Score (NES) | -1.5328622 |
| Nominal p-value | 0.0012903226 |
| FDR q-value | 0.062161945 |
| FWER p-Value | 0.669 |
Table: GSEA Results Summary

  

Fig 1: Enrichment plot: KEGG\_ENDOCYTOSIS      
 Profile of the Running ES Score & Positions of GeneSet Members on the Rank Ordered List

  

| SYMBOL | TITLE | RANK IN GENE LIST | RANK METRIC SCORE | RUNNING ES | CORE ENRICHMENT || 1 | CXCR2 | na | 110 | 0.961 | 0.0146 | No |
| 2 | DNM3 | na | 441 | 0.616 | 0.0120 | No |
| 3 | CHMP2A | na | 575 | 0.562 | 0.0174 | No |
| 4 | ADRB2 | na | 642 | 0.541 | 0.0254 | No |
| 5 | IL2RB | na | 822 | 0.494 | 0.0273 | No |
| 6 | PARD6A | na | 906 | 0.478 | 0.0333 | No |
| 7 | EHD3 | na | 908 | 0.477 | 0.0430 | No |
| 8 | ACAP1 | na | 920 | 0.475 | 0.0522 | No |
| 9 | SNF8 | na | 1161 | 0.440 | 0.0501 | No |
| 10 | VPS28 | na | 1482 | 0.402 | 0.0436 | No |
| 11 | CLTA | na | 1597 | 0.388 | 0.0463 | No |
| 12 | HSPA1B | na | 1633 | 0.382 | 0.0525 | No |
| 13 | MVB12A | na | 1779 | 0.364 | 0.0533 | No |
| 14 | ARRB2 | na | 1848 | 0.356 | 0.0575 | No |
| 15 | GRK4 | na | 1872 | 0.352 | 0.0636 | No |
| 16 | VPS37D | na | 1891 | 0.350 | 0.0699 | No |
| 17 | VPS36 | na | 2102 | 0.327 | 0.0670 | No |
| 18 | CHMP5 | na | 2146 | 0.322 | 0.0716 | No |
| 19 | RAB11A | na | 2159 | 0.321 | 0.0776 | No |
| 20 | AP2S1 | na | 2172 | 0.320 | 0.0836 | No |
| 21 | VTA1 | na | 2195 | 0.316 | 0.0890 | No |
| 22 | CHMP4B | na | 2419 | 0.294 | 0.0848 | No |
| 23 | TSG101 | na | 2507 | 0.286 | 0.0866 | No |
| 24 | CHMP2B | na | 2510 | 0.286 | 0.0924 | No |
| 25 | VPS45 | na | 2565 | 0.281 | 0.0957 | No |
| 26 | HRAS | na | 2716 | 0.268 | 0.0942 | No |
| 27 | PSD4 | na | 2751 | 0.264 | 0.0981 | No |
| 28 | HSPA8 | na | 2800 | 0.259 | 0.1012 | No |
| 29 | SH3GLB1 | na | 3145 | 0.229 | 0.0900 | No |
| 30 | CHMP3 | na | 3343 | 0.211 | 0.0853 | No |
| 31 | CLTB | na | 3390 | 0.207 | 0.0874 | No |
| 32 | AGAP2 | na | 3536 | 0.197 | 0.0848 | No |
| 33 | VPS25 | na | 4035 | 0.161 | 0.0651 | No |
| 34 | MDM2 | na | 4059 | 0.159 | 0.0673 | No |
| 35 | RAB4A | na | 4473 | 0.131 | 0.0509 | No |
| 36 | CDC42 | na | 4900 | 0.101 | 0.0334 | No |
| 37 | PSD2 | na | 5229 | 0.083 | 0.0200 | No |
| 38 | HSPA1A | na | 5784 | 0.053 | -0.0044 | No |
| 39 | CBLC | na | 5841 | 0.050 | -0.0060 | No |
| 40 | RAB22A | na | 5847 | 0.049 | -0.0052 | No |
| 41 | CHMP4C | na | 5880 | 0.048 | -0.0057 | No |
| 42 | SMAP2 | na | 6063 | 0.038 | -0.0133 | No |
| 43 | PRKCZ | na | 6115 | 0.036 | -0.0150 | No |
| 44 | NTRK1 | na | 6192 | 0.033 | -0.0178 | No |
| 45 | EPN1 | na | 6201 | 0.032 | -0.0175 | No |
| 46 | VPS4A | na | 6252 | 0.030 | -0.0192 | No |
| 47 | SH3GL1 | na | 6292 | 0.029 | -0.0204 | No |
| 48 | EHD1 | na | 6355 | 0.026 | -0.0227 | No |
| 49 | ADRB3 | na | 6376 | 0.026 | -0.0231 | No |
| 50 | ARF6 | na | 6657 | 0.015 | -0.0357 | No |
| 51 | CHMP6 | na | 6903 | 0.006 | -0.0469 | No |
| 52 | VPS37B | na | 6987 | 0.002 | -0.0507 | No |
| 53 | ADRB1 | na | 8552 | 0.000 | -0.1227 | No |
| 54 | KIT | na | 8667 | 0.000 | -0.1280 | No |
| 55 | CXCR4 | na | 9111 | 0.000 | -0.1484 | No |
| 56 | CXCR1 | na | 9116 | 0.000 | -0.1485 | No |
| 57 | PIP5K1B | na | 10813 | 0.000 | -0.2267 | No |
| 58 | IQSEC3 | na | 12880 | 0.000 | -0.3219 | No |
| 59 | CCR5 | na | 12896 | 0.000 | -0.3225 | No |
| 60 | RAB11B | na | 13288 | -0.002 | -0.3405 | No |
| 61 | SH3GL3 | na | 13366 | -0.003 | -0.3440 | No |
| 62 | RAB11FIP4 | na | 13386 | -0.005 | -0.3448 | No |
| 63 | FGFR2 | na | 13468 | -0.007 | -0.3484 | No |
| 64 | KDR | na | 13617 | -0.011 | -0.3550 | No |
| 65 | ARAP2 | na | 13674 | -0.012 | -0.3573 | No |
| 66 | RET | na | 13693 | -0.012 | -0.3579 | No |
| 67 | AP2M1 | na | 13765 | -0.013 | -0.3609 | No |
| 68 | SMAP1 | na | 13831 | -0.015 | -0.3636 | No |
| 69 | IL2RA | na | 14028 | -0.021 | -0.3722 | No |
| 70 | SH3GLB2 | na | 14050 | -0.022 | -0.3727 | No |
| 71 | STAMBP | na | 14141 | -0.025 | -0.3763 | No |
| 72 | RAB5A | na | 14396 | -0.035 | -0.3873 | No |
| 73 | VPS37A | na | 14450 | -0.038 | -0.3890 | No |
| 74 | RUFY1 | na | 14642 | -0.048 | -0.3968 | No |
| 75 | RAB5C | na | 14973 | -0.066 | -0.4106 | No |
| 76 | LDLRAP1 | na | 15124 | -0.075 | -0.4160 | No |
| 77 | CHMP1B | na | 15138 | -0.076 | -0.4151 | No |
| 78 | FGFR4 | na | 15208 | -0.081 | -0.4166 | No |
| 79 | ARFGAP2 | na | 15252 | -0.083 | -0.4169 | No |
| 80 | ARFGAP1 | na | 15423 | -0.094 | -0.4228 | No |
| 81 | VPS37C | na | 15441 | -0.094 | -0.4216 | No |
| 82 | ARRB1 | na | 15616 | -0.104 | -0.4275 | No |
| 83 | PSD | na | 15777 | -0.114 | -0.4325 | No |
| 84 | SH3KBP1 | na | 15970 | -0.127 | -0.4388 | No |
| 85 | PARD6G | na | 16169 | -0.139 | -0.4451 | No |
| 86 | VPS4B | na | 16617 | -0.171 | -0.4622 | No |
| 87 | DNM1L | na | 16777 | -0.181 | -0.4658 | No |
| 88 | STAM | na | 17007 | -0.197 | -0.4723 | No |
| 89 | PIP5K1A | na | 17050 | -0.199 | -0.4702 | No |
| 90 | EHD4 | na | 17073 | -0.201 | -0.4671 | No |
| 91 | PLD1 | na | 17249 | -0.212 | -0.4708 | No |
| 92 | EPS15 | na | 17416 | -0.225 | -0.4738 | No |
| 93 | CSF1R | na | 17613 | -0.238 | -0.4780 | Yes |
| 94 | ARFGAP3 | na | 17620 | -0.238 | -0.4734 | Yes |
| 95 | AP2A2 | na | 17753 | -0.248 | -0.4744 | Yes |
| 96 | GRK6 | na | 17789 | -0.252 | -0.4709 | Yes |
| 97 | RABEP1 | na | 17851 | -0.257 | -0.4684 | Yes |
| 98 | RNF41 | na | 17902 | -0.262 | -0.4653 | Yes |
| 99 | F2R | na | 17939 | -0.265 | -0.4616 | Yes |
| 100 | PDCD6IP | na | 18034 | -0.272 | -0.4603 | Yes |
| 101 | PLD2 | na | 18051 | -0.273 | -0.4555 | Yes |
| 102 | RAB31 | na | 18095 | -0.276 | -0.4518 | Yes |
| 103 | GIT1 | na | 18141 | -0.281 | -0.4482 | Yes |
| 104 | NEDD4L | na | 18178 | -0.283 | -0.4440 | Yes |
| 105 | ARAP1 | na | 18247 | -0.289 | -0.4413 | Yes |
| 106 | RBSN | na | 18334 | -0.293 | -0.4392 | Yes |
| 107 | STAM2 | na | 18358 | -0.294 | -0.4342 | Yes |
| 108 | HSPA1L | na | 18395 | -0.297 | -0.4298 | Yes |
| 109 | AGAP1 | na | 18416 | -0.298 | -0.4246 | Yes |
| 110 | ERBB4 | na | 18424 | -0.298 | -0.4189 | Yes |
| 111 | ARAP3 | na | 18479 | -0.302 | -0.4152 | Yes |
| 112 | TFRC | na | 18581 | -0.311 | -0.4135 | Yes |
| 113 | IL2RG | na | 18663 | -0.317 | -0.4107 | Yes |
| 114 | TRAF6 | na | 18711 | -0.321 | -0.4063 | Yes |
| 115 | HGS | na | 18723 | -0.322 | -0.4002 | Yes |
| 116 | AP2B1 | na | 18873 | -0.337 | -0.4001 | Yes |
| 117 | ACAP3 | na | 18954 | -0.344 | -0.3968 | Yes |
| 118 | ASAP3 | na | 18985 | -0.347 | -0.3911 | Yes |
| 119 | RAB11FIP5 | na | 19000 | -0.349 | -0.3846 | Yes |
| 120 | HSPA2 | na | 19041 | -0.354 | -0.3792 | Yes |
| 121 | EGFR | na | 19043 | -0.354 | -0.3720 | Yes |
| 122 | DNM2 | na | 19044 | -0.354 | -0.3647 | Yes |
| 123 | WWP1 | na | 19055 | -0.355 | -0.3579 | Yes |
| 124 | SMURF1 | na | 19205 | -0.370 | -0.3572 | Yes |
| 125 | MVB12B | na | 19266 | -0.374 | -0.3523 | Yes |
| 126 | EPN2 | na | 19346 | -0.381 | -0.3482 | Yes |
| 127 | CLTC | na | 19369 | -0.383 | -0.3413 | Yes |
| 128 | EHD2 | na | 19486 | -0.396 | -0.3386 | Yes |
| 129 | DAB2 | na | 19494 | -0.396 | -0.3308 | Yes |
| 130 | PARD3 | na | 19505 | -0.397 | -0.3231 | Yes |
| 131 | PRKCI | na | 19552 | -0.402 | -0.3170 | Yes |
| 132 | FGFR3 | na | 19694 | -0.416 | -0.3150 | Yes |
| 133 | EEA1 | na | 19725 | -0.419 | -0.3078 | Yes |
| 134 | PSD3 | na | 19837 | -0.429 | -0.3041 | Yes |
| 135 | RAB5B | na | 19940 | -0.438 | -0.2998 | Yes |
| 136 | PDGFRA | na | 19955 | -0.440 | -0.2915 | Yes |
| 137 | ITCH | na | 19957 | -0.440 | -0.2825 | Yes |
| 138 | PIP4K2B | na | 19990 | -0.443 | -0.2749 | Yes |
| 139 | AP2A1 | na | 20044 | -0.450 | -0.2681 | Yes |
| 140 | USP8 | na | 20056 | -0.450 | -0.2594 | Yes |
| 141 | NEDD4 | na | 20075 | -0.452 | -0.2510 | Yes |
| 142 | DNM1 | na | 20082 | -0.453 | -0.2420 | Yes |
| 143 | GIT2 | na | 20137 | -0.460 | -0.2351 | Yes |
| 144 | CBLB | na | 20162 | -0.463 | -0.2267 | Yes |
| 145 | ACAP2 | na | 20236 | -0.471 | -0.2204 | Yes |
| 146 | GRK5 | na | 20243 | -0.471 | -0.2111 | Yes |
| 147 | EGF | na | 20244 | -0.471 | -0.2014 | Yes |
| 148 | SRC | na | 20255 | -0.472 | -0.1922 | Yes |
| 149 | SMURF2 | na | 20323 | -0.480 | -0.1855 | Yes |
| 150 | LDLR | na | 20352 | -0.483 | -0.1768 | Yes |
| 151 | EPN3 | na | 20571 | -0.512 | -0.1764 | Yes |
| 152 | RAB11FIP3 | na | 20593 | -0.515 | -0.1668 | Yes |
| 153 | PARD6B | na | 20662 | -0.524 | -0.1592 | Yes |
| 154 | PIKFYVE | na | 20680 | -0.527 | -0.1492 | Yes |
| 155 | FLT1 | na | 20719 | -0.531 | -0.1401 | Yes |
| 156 | MET | na | 20741 | -0.536 | -0.1301 | Yes |
| 157 | ASAP1 | na | 20873 | -0.557 | -0.1247 | Yes |
| 158 | ASAP2 | na | 20926 | -0.564 | -0.1156 | Yes |
| 159 | SH3GL2 | na | 21002 | -0.579 | -0.1072 | Yes |
| 160 | PIP5K1C | na | 21145 | -0.607 | -0.1013 | Yes |
| 161 | IGF1R | na | 21187 | -0.617 | -0.0905 | Yes |
| 162 | CBL | na | 21212 | -0.623 | -0.0789 | Yes |
| 163 | ERBB3 | na | 21248 | -0.630 | -0.0676 | Yes |
| 164 | DNAJC6 | na | 21265 | -0.634 | -0.0554 | Yes |
| 165 | RAB11FIP2 | na | 21337 | -0.653 | -0.0453 | Yes |
| 166 | IQSEC1 | na | 21582 | -0.742 | -0.0413 | Yes |
| 167 | IQSEC2 | na | 21634 | -0.775 | -0.0278 | Yes |
| 168 | GRK1 | na | 21640 | -0.782 | -0.0120 | Yes |
| 169 | RAB11FIP1 | na | 21816 | -1.113 | 0.0027 | Yes |
Table: GSEA details [plain text format]

  

Fig 2: KEGG\_ENDOCYTOSIS      
 Blue-Pink O' Gram in the Space of the Analyzed GeneSet

  

Fig 3: KEGG\_ENDOCYTOSIS: Random ES distribution      
 Gene set null distribution of ES for **KEGG\_ENDOCYTOSIS**
