# Supplementary material for: Regulation of microglia related neuroinflammation contributes to the protective effect of Gelsevirine on ischemic stroke
Source: Front Immunol. 2023 Mar 30;14:1164278. doi: 10.3389/fimmu.2023.1164278 (PMC10098192; doi:10.3389/fimmu.2023.1164278)
Supplement: Supplementary file 6 [file DataSheet_6.zip › fig 5 raw/fig 5-G raw/KEGG.Gsea.1649954846225/KEGG_ENDOMETRIAL_CANCER.html]

Details for gene set KEGG\_ENDOMETRIAL\_CANCER[GSEA]

|  || Dataset | OGD\_DRUG\_DRUG.OGD\_FRUG.cls#Gs\_versus\_MCAO.OGD\_FRUG.cls#Gs\_versus\_MCAO\_repos |
| Phenotype | OGD\_FRUG.cls#Gs\_versus\_MCAO\_repos |
| Upregulated in class | MCAO |
| GeneSet | KEGG\_ENDOMETRIAL\_CANCER |
| Enrichment Score (ES) | -0.6426537 |
| Normalized Enrichment Score (NES) | -1.7494887 |
| Nominal p-value | 0.0 |
| FDR q-value | 0.013251809 |
| FWER p-Value | 0.042 |
Table: GSEA Results Summary

  

Fig 1: Enrichment plot: KEGG\_ENDOMETRIAL\_CANCER      
 Profile of the Running ES Score & Positions of GeneSet Members on the Rank Ordered List

  

| SYMBOL | TITLE | RANK IN GENE LIST | RANK METRIC SCORE | RUNNING ES | CORE ENRICHMENT || 1 | PIK3CG | na | 1248 | 0.428 | -0.0282 | No |
| 2 | HRAS | na | 2716 | 0.268 | -0.0773 | No |
| 3 | BAD | na | 3658 | 0.189 | -0.1077 | No |
| 4 | NRAS | na | 4669 | 0.117 | -0.1461 | No |
| 5 | MAP2K2 | na | 4692 | 0.115 | -0.1393 | No |
| 6 | TCF7 | na | 4825 | 0.106 | -0.1382 | No |
| 7 | MAPK3 | na | 4918 | 0.100 | -0.1356 | No |
| 8 | MYC | na | 5587 | 0.064 | -0.1619 | No |
| 9 | GRB2 | na | 5726 | 0.057 | -0.1644 | No |
| 10 | CTNNA2 | na | 6496 | 0.021 | -0.1983 | No |
| 11 | KRAS | na | 6543 | 0.019 | -0.1991 | No |
| 12 | AXIN2 | na | 8241 | 0.000 | -0.2769 | No |
| 13 | ILK | na | 14151 | -0.026 | -0.5459 | No |
| 14 | MAP2K1 | na | 15179 | -0.079 | -0.5876 | No |
| 15 | ARAF | na | 15889 | -0.121 | -0.6119 | No |
| 16 | MAPK1 | na | 16234 | -0.143 | -0.6180 | No |
| 17 | LEF1 | na | 16773 | -0.181 | -0.6305 | Yes |
| 18 | AKT2 | na | 16784 | -0.181 | -0.6187 | Yes |
| 19 | AKT1 | na | 16827 | -0.184 | -0.6081 | Yes |
| 20 | TCF7L1 | na | 16939 | -0.192 | -0.6003 | Yes |
| 21 | RAF1 | na | 17251 | -0.212 | -0.6002 | Yes |
| 22 | PTEN | na | 17342 | -0.220 | -0.5894 | Yes |
| 23 | CTNNA1 | na | 17378 | -0.222 | -0.5760 | Yes |
| 24 | CCND1 | na | 17421 | -0.225 | -0.5628 | Yes |
| 25 | AKT3 | na | 17899 | -0.261 | -0.5669 | Yes |
| 26 | AXIN1 | na | 18098 | -0.276 | -0.5573 | Yes |
| 27 | CDH1 | na | 18147 | -0.281 | -0.5406 | Yes |
| 28 | PIK3CD | na | 18197 | -0.284 | -0.5236 | Yes |
| 29 | CTNNB1 | na | 18285 | -0.290 | -0.5080 | Yes |
| 30 | PIK3R2 | na | 18436 | -0.299 | -0.4947 | Yes |
| 31 | PIK3CA | na | 18513 | -0.305 | -0.4775 | Yes |
| 32 | MLH1 | na | 19016 | -0.350 | -0.4769 | Yes |
| 33 | FOXO3 | na | 19023 | -0.351 | -0.4534 | Yes |
| 34 | EGFR | na | 19043 | -0.354 | -0.4304 | Yes |
| 35 | CASP9 | na | 19243 | -0.373 | -0.4143 | Yes |
| 36 | PIK3R3 | na | 19336 | -0.380 | -0.3928 | Yes |
| 37 | TCF7L2 | na | 19504 | -0.397 | -0.3737 | Yes |
| 38 | PIK3R5 | na | 19509 | -0.397 | -0.3470 | Yes |
| 39 | SOS2 | na | 19626 | -0.409 | -0.3247 | Yes |
| 40 | ELK1 | na | 19706 | -0.417 | -0.3001 | Yes |
| 41 | CTNNA3 | na | 19975 | -0.442 | -0.2825 | Yes |
| 42 | GSK3B | na | 20096 | -0.455 | -0.2573 | Yes |
| 43 | PDPK1 | na | 20100 | -0.455 | -0.2266 | Yes |
| 44 | EGF | na | 20244 | -0.471 | -0.2013 | Yes |
| 45 | ERBB2 | na | 20414 | -0.490 | -0.1760 | Yes |
| 46 | PIK3CB | na | 20535 | -0.507 | -0.1472 | Yes |
| 47 | SOS1 | na | 20563 | -0.511 | -0.1140 | Yes |
| 48 | BRAF | na | 20807 | -0.547 | -0.0882 | Yes |
| 49 | APC | na | 21200 | -0.619 | -0.0643 | Yes |
| 50 | PIK3R1 | na | 21419 | -0.674 | -0.0287 | Yes |
| 51 | APC2 | na | 21564 | -0.733 | 0.0142 | Yes |
Table: GSEA details [plain text format]

  

Fig 2: KEGG\_ENDOMETRIAL\_CANCER      
 Blue-Pink O' Gram in the Space of the Analyzed GeneSet

  

Fig 3: KEGG\_ENDOMETRIAL\_CANCER: Random ES distribution      
 Gene set null distribution of ES for **KEGG\_ENDOMETRIAL\_CANCER**
