# Supplementary material for: Regulation of microglia related neuroinflammation contributes to the protective effect of Gelsevirine on ischemic stroke
Source: Front Immunol. 2023 Mar 30;14:1164278. doi: 10.3389/fimmu.2023.1164278 (PMC10098192; doi:10.3389/fimmu.2023.1164278)
Supplement: Supplementary file 6 [file DataSheet_6.zip › fig 5 raw/fig 5-G raw/KEGG.Gsea.1649954846225/KEGG_ERBB_SIGNALING_PATHWAY.html]

Details for gene set KEGG\_ERBB\_SIGNALING\_PATHWAY[GSEA]

|  || Dataset | OGD\_DRUG\_DRUG.OGD\_FRUG.cls#Gs\_versus\_MCAO.OGD\_FRUG.cls#Gs\_versus\_MCAO\_repos |
| Phenotype | OGD\_FRUG.cls#Gs\_versus\_MCAO\_repos |
| Upregulated in class | MCAO |
| GeneSet | KEGG\_ERBB\_SIGNALING\_PATHWAY |
| Enrichment Score (ES) | -0.5146943 |
| Normalized Enrichment Score (NES) | -1.53311 |
| Nominal p-value | 0.0 |
| FDR q-value | 0.066051975 |
| FWER p-Value | 0.667 |
Table: GSEA Results Summary

  

Fig 1: Enrichment plot: KEGG\_ERBB\_SIGNALING\_PATHWAY      
 Profile of the Running ES Score & Positions of GeneSet Members on the Rank Ordered List

  

| SYMBOL | TITLE | RANK IN GENE LIST | RANK METRIC SCORE | RUNNING ES | CORE ENRICHMENT || 1 | SHC2 | na | 8 | 1.794 | 0.0693 | No |
| 2 | BTC | na | 293 | 0.703 | 0.0835 | No |
| 3 | PIK3CG | na | 1248 | 0.428 | 0.0564 | No |
| 4 | CDKN1A | na | 1773 | 0.366 | 0.0465 | No |
| 5 | HRAS | na | 2716 | 0.268 | 0.0137 | No |
| 6 | EIF4EBP1 | na | 2735 | 0.266 | 0.0232 | No |
| 7 | EREG | na | 3001 | 0.241 | 0.0203 | No |
| 8 | TGFA | na | 3523 | 0.198 | 0.0041 | No |
| 9 | PAK1 | na | 3623 | 0.191 | 0.0070 | No |
| 10 | BAD | na | 3658 | 0.189 | 0.0127 | No |
| 11 | CAMK2A | na | 4204 | 0.149 | -0.0065 | No |
| 12 | PRKCG | na | 4221 | 0.148 | -0.0015 | No |
| 13 | NRAS | na | 4669 | 0.117 | -0.0175 | No |
| 14 | MAP2K2 | na | 4692 | 0.115 | -0.0140 | No |
| 15 | HBEGF | na | 4822 | 0.107 | -0.0158 | No |
| 16 | MAPK3 | na | 4918 | 0.100 | -0.0163 | No |
| 17 | PLCG2 | na | 5215 | 0.084 | -0.0266 | No |
| 18 | MYC | na | 5587 | 0.064 | -0.0411 | No |
| 19 | GRB2 | na | 5726 | 0.057 | -0.0453 | No |
| 20 | CBLC | na | 5841 | 0.050 | -0.0486 | No |
| 21 | JUN | na | 5874 | 0.048 | -0.0482 | No |
| 22 | CDKN1B | na | 5942 | 0.045 | -0.0495 | No |
| 23 | KRAS | na | 6543 | 0.019 | -0.0763 | No |
| 24 | MAPK9 | na | 6752 | 0.011 | -0.0855 | No |
| 25 | NCK1 | na | 6802 | 0.009 | -0.0873 | No |
| 26 | NRG2 | na | 9206 | 0.000 | -0.1976 | No |
| 27 | NRG3 | na | 9208 | 0.000 | -0.1977 | No |
| 28 | NRG4 | na | 9209 | 0.000 | -0.1977 | No |
| 29 | SHC3 | na | 10832 | 0.000 | -0.2721 | No |
| 30 | PRKCB | na | 13570 | -0.009 | -0.3973 | No |
| 31 | MAP2K1 | na | 15179 | -0.079 | -0.4681 | No |
| 32 | RPS6KB2 | na | 15831 | -0.117 | -0.4934 | No |
| 33 | ARAF | na | 15889 | -0.121 | -0.4913 | No |
| 34 | MAPK10 | na | 16183 | -0.140 | -0.4994 | No |
| 35 | MAPK1 | na | 16234 | -0.143 | -0.4961 | No |
| 36 | PTK2 | na | 16640 | -0.173 | -0.5080 | Yes |
| 37 | AKT2 | na | 16784 | -0.181 | -0.5075 | Yes |
| 38 | AKT1 | na | 16827 | -0.184 | -0.5023 | Yes |
| 39 | CAMK2D | na | 16846 | -0.185 | -0.4959 | Yes |
| 40 | CRK | na | 16856 | -0.186 | -0.4891 | Yes |
| 41 | MAP2K4 | na | 16904 | -0.189 | -0.4839 | Yes |
| 42 | RAF1 | na | 17251 | -0.212 | -0.4916 | Yes |
| 43 | RPS6KB1 | na | 17275 | -0.214 | -0.4843 | Yes |
| 44 | SHC4 | na | 17550 | -0.233 | -0.4878 | Yes |
| 45 | PAK2 | na | 17619 | -0.238 | -0.4817 | Yes |
| 46 | MAPK8 | na | 17866 | -0.258 | -0.4830 | Yes |
| 47 | AKT3 | na | 17899 | -0.261 | -0.4743 | Yes |
| 48 | PAK3 | na | 18126 | -0.279 | -0.4739 | Yes |
| 49 | NRG1 | na | 18190 | -0.284 | -0.4657 | Yes |
| 50 | PIK3CD | na | 18197 | -0.284 | -0.4550 | Yes |
| 51 | PRKCA | na | 18221 | -0.286 | -0.4450 | Yes |
| 52 | CAMK2B | na | 18339 | -0.294 | -0.4389 | Yes |
| 53 | ERBB4 | na | 18424 | -0.298 | -0.4312 | Yes |
| 54 | PIK3R2 | na | 18436 | -0.299 | -0.4201 | Yes |
| 55 | PIK3CA | na | 18513 | -0.305 | -0.4118 | Yes |
| 56 | CAMK2G | na | 18646 | -0.316 | -0.4056 | Yes |
| 57 | MAP2K7 | na | 18727 | -0.323 | -0.3967 | Yes |
| 58 | AREG | na | 18795 | -0.329 | -0.3870 | Yes |
| 59 | EGFR | na | 19043 | -0.354 | -0.3846 | Yes |
| 60 | SHC1 | na | 19109 | -0.361 | -0.3736 | Yes |
| 61 | PIK3R3 | na | 19336 | -0.380 | -0.3692 | Yes |
| 62 | CRKL | na | 19431 | -0.390 | -0.3584 | Yes |
| 63 | STAT5A | na | 19487 | -0.396 | -0.3455 | Yes |
| 64 | PIK3R5 | na | 19509 | -0.397 | -0.3311 | Yes |
| 65 | GAB1 | na | 19622 | -0.409 | -0.3203 | Yes |
| 66 | SOS2 | na | 19626 | -0.409 | -0.3046 | Yes |
| 67 | PAK4 | na | 19643 | -0.411 | -0.2894 | Yes |
| 68 | ELK1 | na | 19706 | -0.417 | -0.2760 | Yes |
| 69 | ABL2 | na | 20073 | -0.452 | -0.2753 | Yes |
| 70 | GSK3B | na | 20096 | -0.455 | -0.2587 | Yes |
| 71 | CBLB | na | 20162 | -0.463 | -0.2437 | Yes |
| 72 | EGF | na | 20244 | -0.471 | -0.2291 | Yes |
| 73 | SRC | na | 20255 | -0.472 | -0.2112 | Yes |
| 74 | PAK6 | na | 20295 | -0.477 | -0.1945 | Yes |
| 75 | NCK2 | na | 20375 | -0.485 | -0.1793 | Yes |
| 76 | ERBB2 | na | 20414 | -0.490 | -0.1620 | Yes |
| 77 | PIK3CB | na | 20535 | -0.507 | -0.1478 | Yes |
| 78 | SOS1 | na | 20563 | -0.511 | -0.1293 | Yes |
| 79 | ABL1 | na | 20795 | -0.545 | -0.1187 | Yes |
| 80 | BRAF | na | 20807 | -0.547 | -0.0980 | Yes |
| 81 | STAT5B | na | 20976 | -0.573 | -0.0835 | Yes |
| 82 | CBL | na | 21212 | -0.623 | -0.0701 | Yes |
| 83 | PLCG1 | na | 21230 | -0.627 | -0.0465 | Yes |
| 84 | ERBB3 | na | 21248 | -0.630 | -0.0228 | Yes |
| 85 | MTOR | na | 21338 | -0.653 | -0.0016 | Yes |
| 86 | PIK3R1 | na | 21419 | -0.674 | 0.0209 | Yes |
Table: GSEA details [plain text format]

  

Fig 2: KEGG\_ERBB\_SIGNALING\_PATHWAY      
 Blue-Pink O' Gram in the Space of the Analyzed GeneSet

  

Fig 3: KEGG\_ERBB\_SIGNALING\_PATHWAY: Random ES distribution      
 Gene set null distribution of ES for **KEGG\_ERBB\_SIGNALING\_PATHWAY**
