# Supplementary material for: Regulation of microglia related neuroinflammation contributes to the protective effect of Gelsevirine on ischemic stroke
Source: Front Immunol. 2023 Mar 30;14:1164278. doi: 10.3389/fimmu.2023.1164278 (PMC10098192; doi:10.3389/fimmu.2023.1164278)
Supplement: Supplementary file 6 [file DataSheet_6.zip › fig 5 raw/fig 5-G raw/KEGG.Gsea.1649954846225/KEGG_FOCAL_ADHESION.html]

Details for gene set KEGG\_FOCAL\_ADHESION[GSEA]

|  || Dataset | OGD\_DRUG\_DRUG.OGD\_FRUG.cls#Gs\_versus\_MCAO.OGD\_FRUG.cls#Gs\_versus\_MCAO\_repos |
| Phenotype | OGD\_FRUG.cls#Gs\_versus\_MCAO\_repos |
| Upregulated in class | MCAO |
| GeneSet | KEGG\_FOCAL\_ADHESION |
| Enrichment Score (ES) | -0.54695624 |
| Normalized Enrichment Score (NES) | -1.7516636 |
| Nominal p-value | 0.0 |
| FDR q-value | 0.018556 |
| FWER p-Value | 0.04 |
Table: GSEA Results Summary

  

Fig 1: Enrichment plot: KEGG\_FOCAL\_ADHESION      
 Profile of the Running ES Score & Positions of GeneSet Members on the Rank Ordered List

  

| SYMBOL | TITLE | RANK IN GENE LIST | RANK METRIC SCORE | RUNNING ES | CORE ENRICHMENT || 1 | SHC2 | na | 8 | 1.794 | 0.0316 | No |
| 2 | RAC3 | na | 194 | 0.799 | 0.0374 | No |
| 3 | LAMC3 | na | 692 | 0.529 | 0.0239 | No |
| 4 | VAV3 | na | 1228 | 0.431 | 0.0069 | No |
| 5 | PIK3CG | na | 1248 | 0.428 | 0.0136 | No |
| 6 | RHOA | na | 1999 | 0.338 | -0.0149 | No |
| 7 | LAMB3 | na | 2281 | 0.306 | -0.0224 | No |
| 8 | ITGB7 | na | 2467 | 0.290 | -0.0258 | No |
| 9 | LAMA3 | na | 2493 | 0.287 | -0.0218 | No |
| 10 | PDGFA | na | 2504 | 0.286 | -0.0172 | No |
| 11 | MYL12A | na | 2589 | 0.279 | -0.0161 | No |
| 12 | ITGA2B | na | 2646 | 0.275 | -0.0138 | No |
| 13 | HRAS | na | 2716 | 0.268 | -0.0122 | No |
| 14 | RAP1A | na | 2972 | 0.244 | -0.0196 | No |
| 15 | MYLPF | na | 3088 | 0.234 | -0.0207 | No |
| 16 | MYL12B | na | 3105 | 0.233 | -0.0173 | No |
| 17 | SPP1 | na | 3259 | 0.218 | -0.0205 | No |
| 18 | PPP1CA | na | 3615 | 0.191 | -0.0335 | No |
| 19 | PAK1 | na | 3623 | 0.191 | -0.0304 | No |
| 20 | COL6A6 | na | 3636 | 0.190 | -0.0275 | No |
| 21 | BAD | na | 3658 | 0.189 | -0.0251 | No |
| 22 | LAMA4 | na | 3690 | 0.187 | -0.0232 | No |
| 23 | RAC1 | na | 3821 | 0.178 | -0.0261 | No |
| 24 | COL2A1 | na | 3863 | 0.176 | -0.0248 | No |
| 25 | PRKCG | na | 4221 | 0.148 | -0.0386 | No |
| 26 | LAMC2 | na | 4333 | 0.141 | -0.0412 | No |
| 27 | PGF | na | 4357 | 0.139 | -0.0398 | No |
| 28 | VTN | na | 4384 | 0.138 | -0.0386 | No |
| 29 | PARVG | na | 4452 | 0.132 | -0.0393 | No |
| 30 | COL11A1 | na | 4842 | 0.105 | -0.0554 | No |
| 31 | CDC42 | na | 4900 | 0.101 | -0.0562 | No |
| 32 | MAPK3 | na | 4918 | 0.100 | -0.0552 | No |
| 33 | ITGB3 | na | 4968 | 0.097 | -0.0557 | No |
| 34 | VEGFA | na | 5039 | 0.094 | -0.0573 | No |
| 35 | COMP | na | 5235 | 0.083 | -0.0648 | No |
| 36 | MYL9 | na | 5490 | 0.069 | -0.0753 | No |
| 37 | ACTN2 | na | 5721 | 0.057 | -0.0849 | No |
| 38 | GRB2 | na | 5726 | 0.057 | -0.0840 | No |
| 39 | BIRC2 | na | 5775 | 0.053 | -0.0853 | No |
| 40 | JUN | na | 5874 | 0.048 | -0.0890 | No |
| 41 | ITGA8 | na | 6033 | 0.039 | -0.0956 | No |
| 42 | ITGA11 | na | 6089 | 0.037 | -0.0974 | No |
| 43 | THBS3 | na | 6218 | 0.032 | -0.1028 | No |
| 44 | MYLK2 | na | 6260 | 0.030 | -0.1041 | No |
| 45 | ITGB8 | na | 6284 | 0.029 | -0.1047 | No |
| 46 | VEGFB | na | 6585 | 0.017 | -0.1182 | No |
| 47 | MAPK9 | na | 6752 | 0.011 | -0.1257 | No |
| 48 | ITGB6 | na | 6834 | 0.008 | -0.1293 | No |
| 49 | FYN | na | 6884 | 0.006 | -0.1314 | No |
| 50 | CCND3 | na | 6975 | 0.002 | -0.1355 | No |
| 51 | RAP1B | na | 7005 | 0.001 | -0.1369 | No |
| 52 | CHAD | na | 7760 | 0.000 | -0.1716 | No |
| 53 | VAV1 | na | 8205 | 0.000 | -0.1921 | No |
| 54 | HGF | na | 8228 | 0.000 | -0.1931 | No |
| 55 | MYL10 | na | 9092 | 0.000 | -0.2329 | No |
| 56 | IBSP | na | 9186 | 0.000 | -0.2372 | No |
| 57 | RAC2 | na | 10018 | 0.000 | -0.2756 | No |
| 58 | TNN | na | 10258 | 0.000 | -0.2866 | No |
| 59 | SHC3 | na | 10832 | 0.000 | -0.3130 | No |
| 60 | VEGFC | na | 10945 | 0.000 | -0.3182 | No |
| 61 | MYL2 | na | 11274 | 0.000 | -0.3333 | No |
| 62 | ITGA2 | na | 12406 | 0.000 | -0.3855 | No |
| 63 | CAV3 | na | 12457 | 0.000 | -0.3878 | No |
| 64 | THBS4 | na | 12698 | 0.000 | -0.3989 | No |
| 65 | RASGRF1 | na | 12827 | 0.000 | -0.4048 | No |
| 66 | TNR | na | 13505 | -0.007 | -0.4359 | No |
| 67 | ITGA7 | na | 13508 | -0.007 | -0.4358 | No |
| 68 | RELN | na | 13541 | -0.008 | -0.4372 | No |
| 69 | PDGFD | na | 13561 | -0.009 | -0.4379 | No |
| 70 | PRKCB | na | 13570 | -0.009 | -0.4381 | No |
| 71 | LAMA1 | na | 13573 | -0.009 | -0.4380 | No |
| 72 | ITGB5 | na | 13587 | -0.010 | -0.4384 | No |
| 73 | KDR | na | 13617 | -0.011 | -0.4396 | No |
| 74 | ITGA9 | na | 13709 | -0.013 | -0.4436 | No |
| 75 | ITGA4 | na | 13745 | -0.013 | -0.4450 | No |
| 76 | ACTB | na | 13965 | -0.019 | -0.4547 | No |
| 77 | ILK | na | 14151 | -0.026 | -0.4628 | No |
| 78 | FLT4 | na | 14284 | -0.031 | -0.4683 | No |
| 79 | VEGFD | na | 14442 | -0.038 | -0.4749 | No |
| 80 | MYLK3 | na | 14999 | -0.068 | -0.4993 | No |
| 81 | PPP1CB | na | 15011 | -0.068 | -0.4986 | No |
| 82 | MAP2K1 | na | 15179 | -0.079 | -0.5049 | No |
| 83 | PDGFC | na | 15436 | -0.094 | -0.5150 | No |
| 84 | ITGA10 | na | 15634 | -0.105 | -0.5223 | No |
| 85 | COL5A3 | na | 15648 | -0.106 | -0.5210 | No |
| 86 | MYL7 | na | 15980 | -0.127 | -0.5340 | No |
| 87 | PARVA | na | 16073 | -0.133 | -0.5358 | No |
| 88 | PPP1CC | na | 16090 | -0.134 | -0.5342 | No |
| 89 | ACTG1 | na | 16172 | -0.139 | -0.5354 | No |
| 90 | CAV1 | na | 16173 | -0.139 | -0.5330 | No |
| 91 | MAPK10 | na | 16183 | -0.140 | -0.5309 | No |
| 92 | MAPK1 | na | 16234 | -0.143 | -0.5306 | No |
| 93 | CAPN2 | na | 16345 | -0.151 | -0.5330 | No |
| 94 | PTK2 | na | 16640 | -0.173 | -0.5435 | No |
| 95 | ITGB1 | na | 16661 | -0.174 | -0.5413 | No |
| 96 | AKT2 | na | 16784 | -0.181 | -0.5437 | Yes |
| 97 | AKT1 | na | 16827 | -0.184 | -0.5424 | Yes |
| 98 | CRK | na | 16856 | -0.186 | -0.5403 | Yes |
| 99 | PARVB | na | 16857 | -0.186 | -0.5370 | Yes |
| 100 | CAV2 | na | 16878 | -0.187 | -0.5346 | Yes |
| 101 | VWF | na | 16951 | -0.193 | -0.5345 | Yes |
| 102 | ACTN3 | na | 17217 | -0.210 | -0.5430 | Yes |
| 103 | RAF1 | na | 17251 | -0.212 | -0.5407 | Yes |
| 104 | BIRC3 | na | 17295 | -0.216 | -0.5388 | Yes |
| 105 | PTEN | na | 17342 | -0.220 | -0.5370 | Yes |
| 106 | ITGA5 | na | 17393 | -0.223 | -0.5354 | Yes |
| 107 | CCND1 | na | 17421 | -0.225 | -0.5326 | Yes |
| 108 | ZYX | na | 17447 | -0.226 | -0.5297 | Yes |
| 109 | BCL2 | na | 17522 | -0.232 | -0.5290 | Yes |
| 110 | SHC4 | na | 17550 | -0.233 | -0.5261 | Yes |
| 111 | PDGFB | na | 17563 | -0.234 | -0.5225 | Yes |
| 112 | ITGB4 | na | 17565 | -0.234 | -0.5183 | Yes |
| 113 | PAK2 | na | 17619 | -0.238 | -0.5165 | Yes |
| 114 | COL11A2 | na | 17711 | -0.245 | -0.5163 | Yes |
| 115 | IGF1 | na | 17740 | -0.247 | -0.5132 | Yes |
| 116 | VASP | na | 17801 | -0.253 | -0.5115 | Yes |
| 117 | BCAR1 | na | 17804 | -0.253 | -0.5071 | Yes |
| 118 | MAPK8 | na | 17866 | -0.258 | -0.5053 | Yes |
| 119 | DIAPH1 | na | 17895 | -0.261 | -0.5019 | Yes |
| 120 | AKT3 | na | 17899 | -0.261 | -0.4974 | Yes |
| 121 | COL5A2 | na | 18110 | -0.277 | -0.5021 | Yes |
| 122 | PAK3 | na | 18126 | -0.279 | -0.4978 | Yes |
| 123 | PIK3CD | na | 18197 | -0.284 | -0.4960 | Yes |
| 124 | PRKCA | na | 18221 | -0.286 | -0.4920 | Yes |
| 125 | CCND2 | na | 18282 | -0.290 | -0.4896 | Yes |
| 126 | CTNNB1 | na | 18285 | -0.290 | -0.4845 | Yes |
| 127 | PIK3R2 | na | 18436 | -0.299 | -0.4861 | Yes |
| 128 | LAMB1 | na | 18455 | -0.300 | -0.4815 | Yes |
| 129 | PIK3CA | na | 18513 | -0.305 | -0.4787 | Yes |
| 130 | MYLK | na | 18701 | -0.321 | -0.4816 | Yes |
| 131 | COL3A1 | na | 18869 | -0.337 | -0.4833 | Yes |
| 132 | EGFR | na | 19043 | -0.354 | -0.4850 | Yes |
| 133 | COL1A2 | na | 19087 | -0.358 | -0.4806 | Yes |
| 134 | SHC1 | na | 19109 | -0.361 | -0.4751 | Yes |
| 135 | THBS2 | na | 19115 | -0.362 | -0.4689 | Yes |
| 136 | LAMA5 | na | 19183 | -0.368 | -0.4654 | Yes |
| 137 | COL6A2 | na | 19189 | -0.369 | -0.4591 | Yes |
| 138 | PPP1R12A | na | 19275 | -0.375 | -0.4563 | Yes |
| 139 | PIK3R3 | na | 19336 | -0.380 | -0.4523 | Yes |
| 140 | PXN | na | 19391 | -0.386 | -0.4479 | Yes |
| 141 | CRKL | na | 19431 | -0.390 | -0.4427 | Yes |
| 142 | ACTN4 | na | 19490 | -0.396 | -0.4384 | Yes |
| 143 | PIK3R5 | na | 19509 | -0.397 | -0.4321 | Yes |
| 144 | SOS2 | na | 19626 | -0.409 | -0.4302 | Yes |
| 145 | PAK4 | na | 19643 | -0.411 | -0.4236 | Yes |
| 146 | ELK1 | na | 19706 | -0.417 | -0.4190 | Yes |
| 147 | ROCK1 | na | 19820 | -0.427 | -0.4166 | Yes |
| 148 | COL1A1 | na | 19936 | -0.438 | -0.4141 | Yes |
| 149 | PDGFRA | na | 19955 | -0.440 | -0.4070 | Yes |
| 150 | VAV2 | na | 19992 | -0.443 | -0.4008 | Yes |
| 151 | GSK3B | na | 20096 | -0.455 | -0.3974 | Yes |
| 152 | PDPK1 | na | 20100 | -0.455 | -0.3894 | Yes |
| 153 | COL6A1 | na | 20104 | -0.456 | -0.3815 | Yes |
| 154 | LAMA2 | na | 20114 | -0.457 | -0.3737 | Yes |
| 155 | LAMB2 | na | 20135 | -0.460 | -0.3665 | Yes |
| 156 | EGF | na | 20244 | -0.471 | -0.3630 | Yes |
| 157 | ITGA6 | na | 20245 | -0.471 | -0.3546 | Yes |
| 158 | SRC | na | 20255 | -0.472 | -0.3466 | Yes |
| 159 | PAK6 | na | 20295 | -0.477 | -0.3399 | Yes |
| 160 | XIAP | na | 20306 | -0.478 | -0.3318 | Yes |
| 161 | ERBB2 | na | 20414 | -0.490 | -0.3280 | Yes |
| 162 | RAPGEF1 | na | 20440 | -0.495 | -0.3204 | Yes |
| 163 | ARHGAP5 | na | 20516 | -0.503 | -0.3148 | Yes |
| 164 | DOCK1 | na | 20523 | -0.505 | -0.3061 | Yes |
| 165 | PIK3CB | na | 20535 | -0.507 | -0.2976 | Yes |
| 166 | SOS1 | na | 20563 | -0.511 | -0.2897 | Yes |
| 167 | ACTN1 | na | 20635 | -0.521 | -0.2837 | Yes |
| 168 | ROCK2 | na | 20637 | -0.521 | -0.2745 | Yes |
| 169 | ITGA3 | na | 20718 | -0.531 | -0.2687 | Yes |
| 170 | FLT1 | na | 20719 | -0.531 | -0.2592 | Yes |
| 171 | MET | na | 20741 | -0.536 | -0.2506 | Yes |
| 172 | BRAF | na | 20807 | -0.547 | -0.2439 | Yes |
| 173 | COL4A1 | na | 20907 | -0.561 | -0.2384 | Yes |
| 174 | VCL | na | 20929 | -0.565 | -0.2293 | Yes |
| 175 | PDGFRB | na | 20968 | -0.572 | -0.2209 | Yes |
| 176 | ARHGAP35 | na | 20999 | -0.579 | -0.2119 | Yes |
| 177 | FLNA | na | 21107 | -0.600 | -0.2062 | Yes |
| 178 | PIP5K1C | na | 21145 | -0.607 | -0.1971 | Yes |
| 179 | COL5A1 | na | 21162 | -0.610 | -0.1869 | Yes |
| 180 | IGF1R | na | 21187 | -0.617 | -0.1770 | Yes |
| 181 | ITGAV | na | 21226 | -0.625 | -0.1676 | Yes |
| 182 | COL4A6 | na | 21228 | -0.627 | -0.1565 | Yes |
| 183 | TNC | na | 21238 | -0.628 | -0.1457 | Yes |
| 184 | LAMC1 | na | 21239 | -0.629 | -0.1345 | Yes |
| 185 | TLN1 | na | 21253 | -0.632 | -0.1238 | Yes |
| 186 | COL4A2 | na | 21287 | -0.639 | -0.1139 | Yes |
| 187 | FN1 | na | 21407 | -0.672 | -0.1075 | Yes |
| 188 | PIK3R1 | na | 21419 | -0.674 | -0.0959 | Yes |
| 189 | COL6A3 | na | 21436 | -0.681 | -0.0845 | Yes |
| 190 | FLNB | na | 21452 | -0.687 | -0.0730 | Yes |
| 191 | TLN2 | na | 21465 | -0.693 | -0.0612 | Yes |
| 192 | FLNC | na | 21608 | -0.761 | -0.0542 | Yes |
| 193 | THBS1 | na | 21664 | -0.806 | -0.0423 | Yes |
| 194 | COL4A4 | na | 21703 | -0.851 | -0.0289 | Yes |
| 195 | ITGA1 | na | 21792 | -1.028 | -0.0146 | Yes |
| 196 | TNXB | na | 21793 | -1.031 | 0.0038 | Yes |
Table: GSEA details [plain text format]

  

Fig 2: KEGG\_FOCAL\_ADHESION      
 Blue-Pink O' Gram in the Space of the Analyzed GeneSet

  

Fig 3: KEGG\_FOCAL\_ADHESION: Random ES distribution      
 Gene set null distribution of ES for **KEGG\_FOCAL\_ADHESION**
