# Supplementary material for: Regulation of microglia related neuroinflammation contributes to the protective effect of Gelsevirine on ischemic stroke
Source: Front Immunol. 2023 Mar 30;14:1164278. doi: 10.3389/fimmu.2023.1164278 (PMC10098192; doi:10.3389/fimmu.2023.1164278)
Supplement: Supplementary file 6 [file DataSheet_6.zip › fig 5 raw/fig 5-G raw/KEGG.Gsea.1649954846225/KEGG_GLUTATHIONE_METABOLISM.html]

Details for gene set KEGG\_GLUTATHIONE\_METABOLISM[GSEA]

|  || Dataset | OGD\_DRUG\_DRUG.OGD\_FRUG.cls#Gs\_versus\_MCAO.OGD\_FRUG.cls#Gs\_versus\_MCAO\_repos |
| Phenotype | OGD\_FRUG.cls#Gs\_versus\_MCAO\_repos |
| Upregulated in class | Gs |
| GeneSet | KEGG\_GLUTATHIONE\_METABOLISM |
| Enrichment Score (ES) | 0.5150389 |
| Normalized Enrichment Score (NES) | 1.5073305 |
| Nominal p-value | 0.008928572 |
| FDR q-value | 0.0994064 |
| FWER p-Value | 0.645 |
Table: GSEA Results Summary

  

Fig 1: Enrichment plot: KEGG\_GLUTATHIONE\_METABOLISM      
 Profile of the Running ES Score & Positions of GeneSet Members on the Rank Ordered List

  

| SYMBOL | TITLE | RANK IN GENE LIST | RANK METRIC SCORE | RUNNING ES | CORE ENRICHMENT || 1 | GPX7 | na | 364 | 0.648 | 0.0480 | Yes |
| 2 | MGST1 | na | 682 | 0.531 | 0.0865 | Yes |
| 3 | GSTM5 | na | 917 | 0.475 | 0.1232 | Yes |
| 4 | MGST3 | na | 1032 | 0.460 | 0.1639 | Yes |
| 5 | GSTA4 | na | 1142 | 0.443 | 0.2031 | Yes |
| 6 | GSTO2 | na | 1295 | 0.422 | 0.2383 | Yes |
| 7 | GPX4 | na | 1490 | 0.401 | 0.2694 | Yes |
| 8 | GSTO1 | na | 1917 | 0.347 | 0.2846 | Yes |
| 9 | GSTP1 | na | 1927 | 0.346 | 0.3187 | Yes |
| 10 | GPX1 | na | 2054 | 0.332 | 0.3460 | Yes |
| 11 | GSTM2 | na | 2075 | 0.330 | 0.3781 | Yes |
| 12 | TXNDC12 | na | 2137 | 0.322 | 0.4075 | Yes |
| 13 | GSTA3 | na | 2150 | 0.321 | 0.4390 | Yes |
| 14 | MGST2 | na | 2780 | 0.261 | 0.4362 | Yes |
| 15 | GSTM1 | na | 2988 | 0.243 | 0.4510 | Yes |
| 16 | GGCT | na | 3047 | 0.237 | 0.4720 | Yes |
| 17 | GPX6 | na | 3463 | 0.202 | 0.4731 | Yes |
| 18 | GSTZ1 | na | 3908 | 0.172 | 0.4700 | Yes |
| 19 | SRM | na | 3965 | 0.167 | 0.4841 | Yes |
| 20 | GPX3 | na | 4010 | 0.163 | 0.4984 | Yes |
| 21 | GSS | na | 4039 | 0.160 | 0.5131 | Yes |
| 22 | SMS | na | 4309 | 0.142 | 0.5150 | Yes |
| 23 | IDH1 | na | 4951 | 0.098 | 0.4955 | No |
| 24 | GSTK1 | na | 5170 | 0.086 | 0.4941 | No |
| 25 | LAP3 | na | 5388 | 0.074 | 0.4916 | No |
| 26 | GSTA2 | na | 5397 | 0.074 | 0.4986 | No |
| 27 | ANPEP | na | 5499 | 0.068 | 0.5008 | No |
| 28 | ODC1 | na | 5509 | 0.068 | 0.5071 | No |
| 29 | IDH2 | na | 5926 | 0.045 | 0.4926 | No |
| 30 | GCLM | na | 6072 | 0.037 | 0.4897 | No |
| 31 | RRM2 | na | 6142 | 0.035 | 0.4900 | No |
| 32 | GSTM3 | na | 7079 | 0.000 | 0.4471 | No |
| 33 | GGT7 | na | 11387 | 0.000 | 0.2498 | No |
| 34 | GPX2 | na | 13138 | 0.000 | 0.1696 | No |
| 35 | GPX5 | na | 13139 | 0.000 | 0.1696 | No |
| 36 | GSTA1 | na | 13344 | -0.003 | 0.1606 | No |
| 37 | PGD | na | 13398 | -0.005 | 0.1587 | No |
| 38 | GGT5 | na | 14022 | -0.021 | 0.1322 | No |
| 39 | GSTM4 | na | 14526 | -0.042 | 0.1133 | No |
| 40 | GGT6 | na | 14844 | -0.059 | 0.1046 | No |
| 41 | GSR | na | 15404 | -0.093 | 0.0883 | No |
| 42 | GGT1 | na | 15633 | -0.105 | 0.0884 | No |
| 43 | RRM1 | na | 15819 | -0.116 | 0.0915 | No |
| 44 | OPLAH | na | 16223 | -0.142 | 0.0872 | No |
| 45 | GCLC | na | 17950 | -0.266 | 0.0346 | No |
| 46 | RRM2B | na | 18181 | -0.283 | 0.0524 | No |
| 47 | GSTT2 | na | 18427 | -0.298 | 0.0709 | No |
| 48 | GSTT1 | na | 21731 | -0.871 | 0.0066 | No |
Table: GSEA details [plain text format]

  

Fig 2: KEGG\_GLUTATHIONE\_METABOLISM      
 Blue-Pink O' Gram in the Space of the Analyzed GeneSet

  

Fig 3: KEGG\_GLUTATHIONE\_METABOLISM: Random ES distribution      
 Gene set null distribution of ES for **KEGG\_GLUTATHIONE\_METABOLISM**
