# Supplementary material for: Regulation of microglia related neuroinflammation contributes to the protective effect of Gelsevirine on ischemic stroke
Source: Front Immunol. 2023 Mar 30;14:1164278. doi: 10.3389/fimmu.2023.1164278 (PMC10098192; doi:10.3389/fimmu.2023.1164278)
Supplement: Supplementary file 6 [file DataSheet_6.zip › fig 5 raw/fig 5-G raw/KEGG.Gsea.1649954846225/KEGG_GLYCOLYSIS_GLUCONEOGENESIS.html]

Details for gene set KEGG\_GLYCOLYSIS\_GLUCONEOGENESIS[GSEA]

|  || Dataset | OGD\_DRUG\_DRUG.OGD\_FRUG.cls#Gs\_versus\_MCAO.OGD\_FRUG.cls#Gs\_versus\_MCAO\_repos |
| Phenotype | OGD\_FRUG.cls#Gs\_versus\_MCAO\_repos |
| Upregulated in class | Gs |
| GeneSet | KEGG\_GLYCOLYSIS\_GLUCONEOGENESIS |
| Enrichment Score (ES) | 0.4393878 |
| Normalized Enrichment Score (NES) | 1.3189431 |
| Nominal p-value | 0.06329114 |
| FDR q-value | 0.27015728 |
| FWER p-Value | 0.99 |
Table: GSEA Results Summary

  

Fig 1: Enrichment plot: KEGG\_GLYCOLYSIS\_GLUCONEOGENESIS      
 Profile of the Running ES Score & Positions of GeneSet Members on the Rank Ordered List

  

| SYMBOL | TITLE | RANK IN GENE LIST | RANK METRIC SCORE | RUNNING ES | CORE ENRICHMENT || 1 | FBP1 | na | 1024 | 0.461 | 0.0066 | Yes |
| 2 | ADH5 | na | 1254 | 0.428 | 0.0457 | Yes |
| 3 | GAPDH | na | 1541 | 0.395 | 0.0784 | Yes |
| 4 | PCK1 | na | 1622 | 0.384 | 0.1192 | Yes |
| 5 | AKR1A1 | na | 2085 | 0.330 | 0.1363 | Yes |
| 6 | PCK2 | na | 2203 | 0.315 | 0.1675 | Yes |
| 7 | ALDH3B2 | na | 2211 | 0.314 | 0.2036 | Yes |
| 8 | PGAM2 | na | 2294 | 0.304 | 0.2352 | Yes |
| 9 | ALDOC | na | 2593 | 0.279 | 0.2539 | Yes |
| 10 | ALDH3B1 | na | 2884 | 0.251 | 0.2698 | Yes |
| 11 | DLD | na | 3029 | 0.238 | 0.2908 | Yes |
| 12 | ENO3 | na | 3081 | 0.234 | 0.3157 | Yes |
| 13 | LDHA | na | 3144 | 0.229 | 0.3394 | Yes |
| 14 | ACSS2 | na | 3148 | 0.229 | 0.3658 | Yes |
| 15 | LDHB | na | 3243 | 0.219 | 0.3869 | Yes |
| 16 | BPGM | na | 3287 | 0.216 | 0.4100 | Yes |
| 17 | ACSS1 | na | 3290 | 0.215 | 0.4349 | Yes |
| 18 | PDHB | na | 3667 | 0.187 | 0.4394 | Yes |
| 19 | PGK1 | na | 4074 | 0.158 | 0.4391 | No |
| 20 | PKM | na | 5020 | 0.095 | 0.4068 | No |
| 21 | TPI1 | na | 5033 | 0.094 | 0.4172 | No |
| 22 | ALDOA | na | 5121 | 0.089 | 0.4235 | No |
| 23 | ENO2 | na | 5217 | 0.084 | 0.4289 | No |
| 24 | ADH7 | na | 5804 | 0.052 | 0.4081 | No |
| 25 | ENO1 | na | 6025 | 0.039 | 0.4026 | No |
| 26 | ALDH3A1 | na | 6135 | 0.035 | 0.4017 | No |
| 27 | ALDH9A1 | na | 6267 | 0.030 | 0.3991 | No |
| 28 | GCK | na | 8093 | 0.000 | 0.3155 | No |
| 29 | LDHC | na | 8139 | 0.000 | 0.3134 | No |
| 30 | ADH4 | na | 8140 | 0.000 | 0.3134 | No |
| 31 | G6PC2 | na | 9932 | 0.000 | 0.2313 | No |
| 32 | GALM | na | 10134 | 0.000 | 0.2221 | No |
| 33 | PDHA2 | na | 10980 | 0.000 | 0.1834 | No |
| 34 | FBP2 | na | 11004 | 0.000 | 0.1823 | No |
| 35 | ALDH1A3 | na | 11926 | 0.000 | 0.1401 | No |
| 36 | ALDH1B1 | na | 11932 | 0.000 | 0.1399 | No |
| 37 | PGK2 | na | 12394 | 0.000 | 0.1188 | No |
| 38 | HK3 | na | 13324 | -0.002 | 0.0765 | No |
| 39 | PKLR | na | 13410 | -0.006 | 0.0732 | No |
| 40 | PGM2 | na | 13586 | -0.010 | 0.0664 | No |
| 41 | ALDH2 | na | 13607 | -0.010 | 0.0666 | No |
| 42 | PGM1 | na | 14386 | -0.035 | 0.0350 | No |
| 43 | PFKM | na | 14908 | -0.063 | 0.0185 | No |
| 44 | PFKL | na | 15100 | -0.074 | 0.0182 | No |
| 45 | DLAT | na | 15107 | -0.074 | 0.0266 | No |
| 46 | PFKP | na | 15977 | -0.127 | 0.0015 | No |
| 47 | PDHA1 | na | 16103 | -0.134 | 0.0113 | No |
| 48 | ALDOB | na | 16323 | -0.149 | 0.0186 | No |
| 49 | ALDH7A1 | na | 17116 | -0.204 | 0.0059 | No |
| 50 | HK1 | na | 17572 | -0.235 | 0.0123 | No |
| 51 | PGAM1 | na | 17749 | -0.248 | 0.0330 | No |
| 52 | ALDH3A2 | na | 17759 | -0.249 | 0.0615 | No |
| 53 | HK2 | na | 18028 | -0.271 | 0.0807 | No |
| 54 | LDHAL6B | na | 21678 | -0.823 | 0.0090 | No |
Table: GSEA details [plain text format]

  

Fig 2: KEGG\_GLYCOLYSIS\_GLUCONEOGENESIS      
 Blue-Pink O' Gram in the Space of the Analyzed GeneSet

  

Fig 3: KEGG\_GLYCOLYSIS\_GLUCONEOGENESIS: Random ES distribution      
 Gene set null distribution of ES for **KEGG\_GLYCOLYSIS\_GLUCONEOGENESIS**
