# Supplementary material for: Regulation of microglia related neuroinflammation contributes to the protective effect of Gelsevirine on ischemic stroke
Source: Front Immunol. 2023 Mar 30;14:1164278. doi: 10.3389/fimmu.2023.1164278 (PMC10098192; doi:10.3389/fimmu.2023.1164278)
Supplement: Supplementary file 6 [file DataSheet_6.zip › fig 5 raw/fig 5-G raw/KEGG.Gsea.1649954846225/KEGG_HISTIDINE_METABOLISM.html]

Details for gene set KEGG\_HISTIDINE\_METABOLISM[GSEA]

|  || Dataset | OGD\_DRUG\_DRUG.OGD\_FRUG.cls#Gs\_versus\_MCAO.OGD\_FRUG.cls#Gs\_versus\_MCAO\_repos |
| Phenotype | OGD\_FRUG.cls#Gs\_versus\_MCAO\_repos |
| Upregulated in class | Gs |
| GeneSet | KEGG\_HISTIDINE\_METABOLISM |
| Enrichment Score (ES) | 0.4855322 |
| Normalized Enrichment Score (NES) | 1.2899003 |
| Nominal p-value | 0.11357341 |
| FDR q-value | 0.30361825 |
| FWER p-Value | 0.995 |
Table: GSEA Results Summary

  

Fig 1: Enrichment plot: KEGG\_HISTIDINE\_METABOLISM      
 Profile of the Running ES Score & Positions of GeneSet Members on the Rank Ordered List

  

| SYMBOL | TITLE | RANK IN GENE LIST | RANK METRIC SCORE | RUNNING ES | CORE ENRICHMENT || 1 | DDC | na | 947 | 0.471 | 0.0605 | Yes |
| 2 | ASPA | na | 978 | 0.467 | 0.1621 | Yes |
| 3 | METTL6 | na | 1516 | 0.398 | 0.2252 | Yes |
| 4 | AMDHD1 | na | 2157 | 0.321 | 0.2667 | Yes |
| 5 | ALDH3B2 | na | 2211 | 0.314 | 0.3336 | Yes |
| 6 | ACY3 | na | 2819 | 0.258 | 0.3626 | Yes |
| 7 | ALDH3B1 | na | 2884 | 0.251 | 0.4151 | Yes |
| 8 | HDC | na | 3469 | 0.201 | 0.4327 | Yes |
| 9 | LCMT1 | na | 3580 | 0.193 | 0.4702 | Yes |
| 10 | MAOA | na | 4152 | 0.153 | 0.4779 | Yes |
| 11 | TRMT11 | na | 4581 | 0.123 | 0.4855 | Yes |
| 12 | HEMK1 | na | 5162 | 0.086 | 0.4781 | No |
| 13 | ALDH3A1 | na | 6135 | 0.035 | 0.4413 | No |
| 14 | CNDP1 | na | 6221 | 0.031 | 0.4444 | No |
| 15 | ALDH9A1 | na | 6267 | 0.030 | 0.4489 | No |
| 16 | LCMT2 | na | 8543 | 0.000 | 0.3448 | No |
| 17 | AOC1 | na | 9712 | 0.000 | 0.2913 | No |
| 18 | ALDH1A3 | na | 11926 | 0.000 | 0.1900 | No |
| 19 | ALDH1B1 | na | 11932 | 0.000 | 0.1898 | No |
| 20 | HNMT | na | 11968 | 0.000 | 0.1882 | No |
| 21 | ALDH2 | na | 13607 | -0.010 | 0.1155 | No |
| 22 | FTCD | na | 14333 | -0.033 | 0.0896 | No |
| 23 | MAOB | na | 16182 | -0.140 | 0.0358 | No |
| 24 | ALDH7A1 | na | 17116 | -0.204 | 0.0380 | No |
| 25 | HAL | na | 17241 | -0.212 | 0.0791 | No |
| 26 | ALDH3A2 | na | 17759 | -0.249 | 0.1103 | No |
| 27 | UROC1 | na | 19045 | -0.354 | 0.1295 | No |
Table: GSEA details [plain text format]

  

Fig 2: KEGG\_HISTIDINE\_METABOLISM      
 Blue-Pink O' Gram in the Space of the Analyzed GeneSet

  

Fig 3: KEGG\_HISTIDINE\_METABOLISM: Random ES distribution      
 Gene set null distribution of ES for **KEGG\_HISTIDINE\_METABOLISM**
