# Supplementary material for: Regulation of microglia related neuroinflammation contributes to the protective effect of Gelsevirine on ischemic stroke
Source: Front Immunol. 2023 Mar 30;14:1164278. doi: 10.3389/fimmu.2023.1164278 (PMC10098192; doi:10.3389/fimmu.2023.1164278)
Supplement: Supplementary file 6 [file DataSheet_6.zip › fig 5 raw/fig 5-G raw/KEGG.Gsea.1649954846225/KEGG_HUNTINGTONS_DISEASE.html]

Details for gene set KEGG\_HUNTINGTONS\_DISEASE[GSEA]

|  || Dataset | OGD\_DRUG\_DRUG.OGD\_FRUG.cls#Gs\_versus\_MCAO.OGD\_FRUG.cls#Gs\_versus\_MCAO\_repos |
| Phenotype | OGD\_FRUG.cls#Gs\_versus\_MCAO\_repos |
| Upregulated in class | Gs |
| GeneSet | KEGG\_HUNTINGTONS\_DISEASE |
| Enrichment Score (ES) | 0.49379796 |
| Normalized Enrichment Score (NES) | 1.7146208 |
| Nominal p-value | 0.0 |
| FDR q-value | 0.02436317 |
| FWER p-Value | 0.136 |
Table: GSEA Results Summary

  

Fig 1: Enrichment plot: KEGG\_HUNTINGTONS\_DISEASE      
 Profile of the Running ES Score & Positions of GeneSet Members on the Rank Ordered List

  

| SYMBOL | TITLE | RANK IN GENE LIST | RANK METRIC SCORE | RUNNING ES | CORE ENRICHMENT || 1 | COX6A2 | na | 162 | 0.836 | 0.0095 | Yes |
| 2 | UQCRB | na | 430 | 0.620 | 0.0098 | Yes |
| 3 | COX6C | na | 440 | 0.616 | 0.0218 | Yes |
| 4 | COX7A2 | na | 449 | 0.612 | 0.0339 | Yes |
| 5 | COX5A | na | 462 | 0.602 | 0.0455 | Yes |
| 6 | NDUFA4 | na | 469 | 0.600 | 0.0574 | Yes |
| 7 | NDUFC1 | na | 493 | 0.590 | 0.0683 | Yes |
| 8 | UQCRH | na | 503 | 0.588 | 0.0799 | Yes |
| 9 | NDUFA2 | na | 510 | 0.586 | 0.0915 | Yes |
| 10 | DNALI1 | na | 536 | 0.574 | 0.1019 | Yes |
| 11 | NDUFB6 | na | 567 | 0.565 | 0.1120 | Yes |
| 12 | COX6B1 | na | 653 | 0.538 | 0.1190 | Yes |
| 13 | NDUFA1 | na | 663 | 0.536 | 0.1295 | Yes |
| 14 | NDUFS4 | na | 674 | 0.532 | 0.1398 | Yes |
| 15 | POLR2G | na | 774 | 0.505 | 0.1455 | Yes |
| 16 | NDUFA4L2 | na | 786 | 0.501 | 0.1551 | Yes |
| 17 | NDUFC2 | na | 793 | 0.500 | 0.1650 | Yes |
| 18 | UQCR10 | na | 800 | 0.498 | 0.1748 | Yes |
| 19 | NDUFB5 | na | 819 | 0.494 | 0.1840 | Yes |
| 20 | COX7B | na | 859 | 0.484 | 0.1920 | Yes |
| 21 | NDUFA5 | na | 1040 | 0.459 | 0.1930 | Yes |
| 22 | VDAC3 | na | 1114 | 0.446 | 0.1987 | Yes |
| 23 | COX7C | na | 1133 | 0.444 | 0.2069 | Yes |
| 24 | NDUFB3 | na | 1166 | 0.439 | 0.2143 | Yes |
| 25 | COX4I1 | na | 1200 | 0.434 | 0.2216 | Yes |
| 26 | POLR2H | na | 1207 | 0.434 | 0.2301 | Yes |
| 27 | NDUFS6 | na | 1280 | 0.424 | 0.2354 | Yes |
| 28 | COX6A1 | na | 1296 | 0.422 | 0.2432 | Yes |
| 29 | COX7A2L | na | 1310 | 0.419 | 0.2511 | Yes |
| 30 | CYCS | na | 1320 | 0.418 | 0.2592 | Yes |
| 31 | COX8A | na | 1327 | 0.417 | 0.2674 | Yes |
| 32 | PPID | na | 1343 | 0.416 | 0.2751 | Yes |
| 33 | NDUFS5 | na | 1350 | 0.415 | 0.2833 | Yes |
| 34 | NDUFB10 | na | 1372 | 0.413 | 0.2907 | Yes |
| 35 | POLR2I | na | 1419 | 0.409 | 0.2968 | Yes |
| 36 | NDUFB9 | na | 1449 | 0.405 | 0.3037 | Yes |
| 37 | NDUFB4 | na | 1457 | 0.405 | 0.3116 | Yes |
| 38 | BAX | na | 1465 | 0.404 | 0.3195 | Yes |
| 39 | NDUFV2 | na | 1503 | 0.400 | 0.3259 | Yes |
| 40 | CLTA | na | 1597 | 0.388 | 0.3295 | Yes |
| 41 | POLR2L | na | 1608 | 0.386 | 0.3368 | Yes |
| 42 | NDUFS3 | na | 1637 | 0.382 | 0.3433 | Yes |
| 43 | POLR2J | na | 1758 | 0.367 | 0.3452 | Yes |
| 44 | NDUFB7 | na | 1789 | 0.363 | 0.3512 | Yes |
| 45 | SOD1 | na | 1799 | 0.362 | 0.3581 | Yes |
| 46 | POLR2D | na | 1820 | 0.360 | 0.3645 | Yes |
| 47 | UQCRQ | na | 1834 | 0.358 | 0.3711 | Yes |
| 48 | POLR2F | na | 1837 | 0.358 | 0.3783 | Yes |
| 49 | COX7A1 | na | 1863 | 0.354 | 0.3843 | Yes |
| 50 | NDUFA3 | na | 1881 | 0.352 | 0.3907 | Yes |
| 51 | NDUFB2 | na | 1907 | 0.348 | 0.3966 | Yes |
| 52 | POLR2C | na | 1926 | 0.346 | 0.4028 | Yes |
| 53 | NDUFA7 | na | 1971 | 0.341 | 0.4077 | Yes |
| 54 | VDAC2 | na | 1982 | 0.340 | 0.4141 | Yes |
| 55 | SLC25A5 | na | 1987 | 0.339 | 0.4208 | Yes |
| 56 | SLC25A4 | na | 2006 | 0.337 | 0.4268 | Yes |
| 57 | GPX1 | na | 2054 | 0.332 | 0.4314 | Yes |
| 58 | AP2S1 | na | 2172 | 0.320 | 0.4325 | Yes |
| 59 | NDUFA8 | na | 2185 | 0.318 | 0.4384 | Yes |
| 60 | UQCRFS1 | na | 2202 | 0.315 | 0.4440 | Yes |
| 61 | COX5B | na | 2237 | 0.311 | 0.4487 | Yes |
| 62 | TBPL1 | na | 2259 | 0.308 | 0.4540 | Yes |
| 63 | CYC1 | na | 2402 | 0.296 | 0.4535 | Yes |
| 64 | NDUFAB1 | na | 2476 | 0.289 | 0.4560 | Yes |
| 65 | SDHD | na | 2574 | 0.281 | 0.4572 | Yes |
| 66 | NDUFA9 | na | 2575 | 0.281 | 0.4629 | Yes |
| 67 | IFT57 | na | 2710 | 0.268 | 0.4622 | Yes |
| 68 | UQCR11 | na | 2718 | 0.268 | 0.4673 | Yes |
| 69 | NDUFS7 | na | 2745 | 0.265 | 0.4715 | Yes |
| 70 | NDUFA10 | na | 2863 | 0.254 | 0.4712 | Yes |
| 71 | NDUFV3 | na | 2869 | 0.253 | 0.4761 | Yes |
| 72 | HDAC2 | na | 2882 | 0.251 | 0.4807 | Yes |
| 73 | SDHC | na | 2894 | 0.251 | 0.4852 | Yes |
| 74 | TFAM | na | 3079 | 0.235 | 0.4815 | Yes |
| 75 | UQCRC2 | na | 3080 | 0.235 | 0.4863 | Yes |
| 76 | POLR2K | na | 3264 | 0.217 | 0.4823 | Yes |
| 77 | NDUFS8 | na | 3269 | 0.217 | 0.4865 | Yes |
| 78 | NDUFA6 | na | 3303 | 0.214 | 0.4893 | Yes |
| 79 | POLR2E | na | 3386 | 0.208 | 0.4897 | Yes |
| 80 | CLTB | na | 3390 | 0.207 | 0.4938 | Yes |
| 81 | SDHB | na | 3526 | 0.198 | 0.4916 | No |
| 82 | DCTN2 | na | 3674 | 0.187 | 0.4886 | No |
| 83 | NDUFS2 | na | 3682 | 0.187 | 0.4921 | No |
| 84 | NDUFB8 | na | 3736 | 0.183 | 0.4934 | No |
| 85 | COX7B2 | na | 4007 | 0.163 | 0.4842 | No |
| 86 | CREB3L4 | na | 4274 | 0.145 | 0.4749 | No |
| 87 | NDUFV1 | na | 4574 | 0.124 | 0.4637 | No |
| 88 | UQCRC1 | na | 4897 | 0.101 | 0.4509 | No |
| 89 | CASP3 | na | 5009 | 0.095 | 0.4477 | No |
| 90 | CREB3 | na | 5728 | 0.056 | 0.4158 | No |
| 91 | SOD2 | na | 5759 | 0.054 | 0.4155 | No |
| 92 | VDAC1 | na | 6111 | 0.036 | 0.4001 | No |
| 93 | GRM5 | na | 7281 | 0.000 | 0.3463 | No |
| 94 | UCP1 | na | 8852 | 0.000 | 0.2740 | No |
| 95 | SLC25A31 | na | 8939 | 0.000 | 0.2701 | No |
| 96 | DNAH3 | na | 9305 | 0.000 | 0.2533 | No |
| 97 | COX8C | na | 11687 | 0.000 | 0.1437 | No |
| 98 | TBPL2 | na | 11947 | 0.000 | 0.1318 | No |
| 99 | CREB3L3 | na | 13285 | -0.001 | 0.0703 | No |
| 100 | DNAL4 | na | 13360 | -0.003 | 0.0669 | No |
| 101 | COX6B2 | na | 13632 | -0.011 | 0.0547 | No |
| 102 | GRIN2B | na | 13653 | -0.011 | 0.0540 | No |
| 103 | PLCB2 | na | 13694 | -0.012 | 0.0524 | No |
| 104 | TBP | na | 13758 | -0.013 | 0.0497 | No |
| 105 | AP2M1 | na | 13765 | -0.013 | 0.0497 | No |
| 106 | DNAH1 | na | 13774 | -0.013 | 0.0496 | No |
| 107 | GRIN1 | na | 14095 | -0.023 | 0.0354 | No |
| 108 | NDUFS1 | na | 14405 | -0.036 | 0.0219 | No |
| 109 | COX4I2 | na | 14503 | -0.041 | 0.0182 | No |
| 110 | SDHA | na | 14573 | -0.044 | 0.0159 | No |
| 111 | BBC3 | na | 15343 | -0.088 | -0.0177 | No |
| 112 | CASP8 | na | 15511 | -0.098 | -0.0234 | No |
| 113 | CREB3L1 | na | 15551 | -0.101 | -0.0231 | No |
| 114 | HDAC1 | na | 15789 | -0.114 | -0.0317 | No |
| 115 | POLR2B | na | 15961 | -0.126 | -0.0370 | No |
| 116 | BDNF | na | 16156 | -0.138 | -0.0431 | No |
| 117 | DNAH2 | na | 16646 | -0.173 | -0.0622 | No |
| 118 | PLCB4 | na | 16825 | -0.184 | -0.0666 | No |
| 119 | TAF4B | na | 16987 | -0.195 | -0.0701 | No |
| 120 | TGM2 | na | 17151 | -0.206 | -0.0734 | No |
| 121 | NRF1 | na | 17405 | -0.224 | -0.0805 | No |
| 122 | TAF4 | na | 17720 | -0.246 | -0.0900 | No |
| 123 | AP2A2 | na | 17753 | -0.248 | -0.0864 | No |
| 124 | CREB3L2 | na | 17814 | -0.254 | -0.0840 | No |
| 125 | CREB1 | na | 18226 | -0.286 | -0.0971 | No |
| 126 | SIN3A | na | 18442 | -0.299 | -0.1009 | No |
| 127 | DCTN4 | na | 18625 | -0.314 | -0.1030 | No |
| 128 | APAF1 | na | 18709 | -0.321 | -0.1003 | No |
| 129 | GNAQ | na | 18773 | -0.327 | -0.0965 | No |
| 130 | PLCB3 | na | 18848 | -0.334 | -0.0932 | No |
| 131 | AP2B1 | na | 18873 | -0.337 | -0.0874 | No |
| 132 | PPARGC1A | na | 19078 | -0.357 | -0.0896 | No |
| 133 | CASP9 | na | 19243 | -0.373 | -0.0896 | No |
| 134 | CLTC | na | 19369 | -0.383 | -0.0876 | No |
| 135 | SP1 | na | 19717 | -0.418 | -0.0951 | No |
| 136 | RCOR1 | na | 19928 | -0.437 | -0.0959 | No |
| 137 | AP2A1 | na | 20044 | -0.450 | -0.0920 | No |
| 138 | CREB5 | na | 20307 | -0.478 | -0.0944 | No |
| 139 | DCTN1 | na | 20357 | -0.484 | -0.0869 | No |
| 140 | DNAL1 | na | 20822 | -0.549 | -0.0971 | No |
| 141 | HIP1 | na | 20961 | -0.571 | -0.0918 | No |
| 142 | ITPR1 | na | 20993 | -0.577 | -0.0816 | No |
| 143 | HTT | na | 21048 | -0.587 | -0.0722 | No |
| 144 | DLG4 | na | 21063 | -0.591 | -0.0608 | No |
| 145 | POLR2A | na | 21164 | -0.610 | -0.0530 | No |
| 146 | CREBBP | na | 21304 | -0.643 | -0.0464 | No |
| 147 | EP300 | na | 21416 | -0.674 | -0.0379 | No |
| 148 | REST | na | 21424 | -0.676 | -0.0245 | No |
| 149 | HAP1 | na | 21433 | -0.680 | -0.0111 | No |
| 150 | PLCB1 | na | 21572 | -0.736 | -0.0025 | No |
| 151 | PPARG | na | 21669 | -0.808 | 0.0095 | No |
Table: GSEA details [plain text format]

  

Fig 2: KEGG\_HUNTINGTONS\_DISEASE      
 Blue-Pink O' Gram in the Space of the Analyzed GeneSet

  

Fig 3: KEGG\_HUNTINGTONS\_DISEASE: Random ES distribution      
 Gene set null distribution of ES for **KEGG\_HUNTINGTONS\_DISEASE**
