# Supplementary material for: Regulation of microglia related neuroinflammation contributes to the protective effect of Gelsevirine on ischemic stroke
Source: Front Immunol. 2023 Mar 30;14:1164278. doi: 10.3389/fimmu.2023.1164278 (PMC10098192; doi:10.3389/fimmu.2023.1164278)
Supplement: Supplementary file 6 [file DataSheet_6.zip › fig 5 raw/fig 5-G raw/KEGG.Gsea.1649954846225/KEGG_INOSITOL_PHOSPHATE_METABOLISM.html]

Details for gene set KEGG\_INOSITOL\_PHOSPHATE\_METABOLISM[GSEA]

|  || Dataset | OGD\_DRUG\_DRUG.OGD\_FRUG.cls#Gs\_versus\_MCAO.OGD\_FRUG.cls#Gs\_versus\_MCAO\_repos |
| Phenotype | OGD\_FRUG.cls#Gs\_versus\_MCAO\_repos |
| Upregulated in class | MCAO |
| GeneSet | KEGG\_INOSITOL\_PHOSPHATE\_METABOLISM |
| Enrichment Score (ES) | -0.6046024 |
| Normalized Enrichment Score (NES) | -1.6572926 |
| Nominal p-value | 0.0014556041 |
| FDR q-value | 0.02497765 |
| FWER p-Value | 0.188 |
Table: GSEA Results Summary

  

Fig 1: Enrichment plot: KEGG\_INOSITOL\_PHOSPHATE\_METABOLISM      
 Profile of the Running ES Score & Positions of GeneSet Members on the Rank Ordered List

  

| SYMBOL | TITLE | RANK IN GENE LIST | RANK METRIC SCORE | RUNNING ES | CORE ENRICHMENT || 1 | MIOX | na | 527 | 0.577 | 0.0127 | No |
| 2 | PIK3CG | na | 1248 | 0.428 | 0.0071 | No |
| 3 | PLCD4 | na | 4218 | 0.148 | -0.1195 | No |
| 4 | TPI1 | na | 5033 | 0.094 | -0.1508 | No |
| 5 | PLCG2 | na | 5215 | 0.084 | -0.1537 | No |
| 6 | INPP5J | na | 5339 | 0.077 | -0.1544 | No |
| 7 | ISYNA1 | na | 5532 | 0.066 | -0.1589 | No |
| 8 | MINPP1 | na | 6027 | 0.039 | -0.1790 | No |
| 9 | CDIPT | na | 6176 | 0.033 | -0.1837 | No |
| 10 | PLCZ1 | na | 6484 | 0.022 | -0.1964 | No |
| 11 | PLCD1 | na | 6813 | 0.009 | -0.2109 | No |
| 12 | PIP5K1B | na | 10813 | 0.000 | -0.3941 | No |
| 13 | PIK3C2G | na | 11106 | 0.000 | -0.4075 | No |
| 14 | ITPK1 | na | 13267 | -0.001 | -0.5064 | No |
| 15 | PLCB2 | na | 13694 | -0.012 | -0.5252 | No |
| 16 | PLCE1 | na | 13860 | -0.016 | -0.5317 | No |
| 17 | IMPA1 | na | 14460 | -0.039 | -0.5567 | No |
| 18 | INPP1 | na | 14946 | -0.065 | -0.5748 | No |
| 19 | PLCD3 | na | 15310 | -0.087 | -0.5859 | No |
| 20 | INPP5K | na | 15497 | -0.097 | -0.5882 | No |
| 21 | ALDH6A1 | na | 15596 | -0.103 | -0.5860 | No |
| 22 | PIP4K2C | na | 16002 | -0.129 | -0.5964 | Yes |
| 23 | PIP4K2A | na | 16075 | -0.133 | -0.5912 | Yes |
| 24 | IPPK | na | 16242 | -0.143 | -0.5896 | Yes |
| 25 | PIK3C3 | na | 16557 | -0.166 | -0.5934 | Yes |
| 26 | IMPA2 | na | 16587 | -0.168 | -0.5840 | Yes |
| 27 | PLCB4 | na | 16825 | -0.184 | -0.5831 | Yes |
| 28 | PIP5K1A | na | 17050 | -0.199 | -0.5807 | Yes |
| 29 | INPP5A | na | 17072 | -0.201 | -0.5688 | Yes |
| 30 | PTEN | na | 17342 | -0.220 | -0.5671 | Yes |
| 31 | PI4KB | na | 17757 | -0.249 | -0.5701 | Yes |
| 32 | PIK3CD | na | 18197 | -0.284 | -0.5721 | Yes |
| 33 | IPMK | na | 18399 | -0.297 | -0.5623 | Yes |
| 34 | PIK3CA | na | 18513 | -0.305 | -0.5480 | Yes |
| 35 | INPP4A | na | 18570 | -0.309 | -0.5308 | Yes |
| 36 | PLCB3 | na | 18848 | -0.334 | -0.5222 | Yes |
| 37 | INPP5B | na | 18889 | -0.339 | -0.5023 | Yes |
| 38 | OCRL | na | 19163 | -0.366 | -0.4914 | Yes |
| 39 | SYNJ2 | na | 19191 | -0.369 | -0.4691 | Yes |
| 40 | PIP4K2B | na | 19990 | -0.443 | -0.4773 | Yes |
| 41 | INPPL1 | na | 20322 | -0.480 | -0.4618 | Yes |
| 42 | PIK3CB | na | 20535 | -0.507 | -0.4391 | Yes |
| 43 | PIKFYVE | na | 20680 | -0.527 | -0.4121 | Yes |
| 44 | PIK3C2A | na | 20684 | -0.527 | -0.3785 | Yes |
| 45 | PI4KA | na | 20686 | -0.527 | -0.3449 | Yes |
| 46 | SYNJ1 | na | 20828 | -0.550 | -0.3162 | Yes |
| 47 | INPP5E | na | 21077 | -0.595 | -0.2895 | Yes |
| 48 | PIP5K1C | na | 21145 | -0.607 | -0.2537 | Yes |
| 49 | ITPKB | na | 21198 | -0.619 | -0.2166 | Yes |
| 50 | PLCG1 | na | 21230 | -0.627 | -0.1779 | Yes |
| 51 | PIK3C2B | na | 21420 | -0.675 | -0.1434 | Yes |
| 52 | INPP4B | na | 21534 | -0.719 | -0.1026 | Yes |
| 53 | PLCB1 | na | 21572 | -0.736 | -0.0573 | Yes |
| 54 | ITPKA | na | 21817 | -1.113 | 0.0027 | Yes |
Table: GSEA details [plain text format]

  

Fig 2: KEGG\_INOSITOL\_PHOSPHATE\_METABOLISM      
 Blue-Pink O' Gram in the Space of the Analyzed GeneSet

  

Fig 3: KEGG\_INOSITOL\_PHOSPHATE\_METABOLISM: Random ES distribution      
 Gene set null distribution of ES for **KEGG\_INOSITOL\_PHOSPHATE\_METABOLISM**
