# Supplementary material for: Regulation of microglia related neuroinflammation contributes to the protective effect of Gelsevirine on ischemic stroke
Source: Front Immunol. 2023 Mar 30;14:1164278. doi: 10.3389/fimmu.2023.1164278 (PMC10098192; doi:10.3389/fimmu.2023.1164278)
Supplement: Supplementary file 6 [file DataSheet_6.zip › fig 5 raw/fig 5-G raw/KEGG.Gsea.1649954846225/KEGG_INSULIN_SIGNALING_PATHWAY.html]

Details for gene set KEGG\_INSULIN\_SIGNALING\_PATHWAY[GSEA]

|  || Dataset | OGD\_DRUG\_DRUG.OGD\_FRUG.cls#Gs\_versus\_MCAO.OGD\_FRUG.cls#Gs\_versus\_MCAO\_repos |
| Phenotype | OGD\_FRUG.cls#Gs\_versus\_MCAO\_repos |
| Upregulated in class | MCAO |
| GeneSet | KEGG\_INSULIN\_SIGNALING\_PATHWAY |
| Enrichment Score (ES) | -0.48573232 |
| Normalized Enrichment Score (NES) | -1.5042844 |
| Nominal p-value | 0.0026490067 |
| FDR q-value | 0.06768927 |
| FWER p-Value | 0.775 |
Table: GSEA Results Summary

  

Fig 1: Enrichment plot: KEGG\_INSULIN\_SIGNALING\_PATHWAY      
 Profile of the Running ES Score & Positions of GeneSet Members on the Rank Ordered List

  

| SYMBOL | TITLE | RANK IN GENE LIST | RANK METRIC SCORE | RUNNING ES | CORE ENRICHMENT || 1 | SHC2 | na | 8 | 1.794 | 0.0493 | No |
| 2 | RPS6 | na | 566 | 0.566 | 0.0394 | No |
| 3 | PRKAA2 | na | 650 | 0.539 | 0.0505 | No |
| 4 | PRKAR1B | na | 788 | 0.500 | 0.0580 | No |
| 5 | RHEB | na | 886 | 0.480 | 0.0669 | No |
| 6 | FBP1 | na | 1024 | 0.461 | 0.0733 | No |
| 7 | PIK3CG | na | 1248 | 0.428 | 0.0749 | No |
| 8 | PCK1 | na | 1622 | 0.384 | 0.0684 | No |
| 9 | PCK2 | na | 2203 | 0.315 | 0.0504 | No |
| 10 | HRAS | na | 2716 | 0.268 | 0.0343 | No |
| 11 | EIF4EBP1 | na | 2735 | 0.266 | 0.0408 | No |
| 12 | CALM1 | na | 3305 | 0.214 | 0.0206 | No |
| 13 | PRKAG1 | na | 3476 | 0.201 | 0.0183 | No |
| 14 | CALM2 | na | 3550 | 0.196 | 0.0204 | No |
| 15 | PPP1CA | na | 3615 | 0.191 | 0.0227 | No |
| 16 | EIF4E2 | na | 3651 | 0.189 | 0.0264 | No |
| 17 | BAD | na | 3658 | 0.189 | 0.0313 | No |
| 18 | PRKAG3 | na | 4161 | 0.153 | 0.0125 | No |
| 19 | EIF4E | na | 4398 | 0.137 | 0.0054 | No |
| 20 | NRAS | na | 4669 | 0.117 | -0.0038 | No |
| 21 | MAP2K2 | na | 4692 | 0.115 | -0.0016 | No |
| 22 | MAPK3 | na | 4918 | 0.100 | -0.0092 | No |
| 23 | SOCS1 | na | 5006 | 0.095 | -0.0105 | No |
| 24 | FLOT1 | na | 5347 | 0.077 | -0.0241 | No |
| 25 | GRB2 | na | 5726 | 0.057 | -0.0399 | No |
| 26 | CBLC | na | 5841 | 0.050 | -0.0437 | No |
| 27 | PRKCZ | na | 6115 | 0.036 | -0.0553 | No |
| 28 | KRAS | na | 6543 | 0.019 | -0.0744 | No |
| 29 | MAPK9 | na | 6752 | 0.011 | -0.0837 | No |
| 30 | SOCS2 | na | 7627 | 0.000 | -0.1239 | No |
| 31 | GCK | na | 8093 | 0.000 | -0.1453 | No |
| 32 | EIF4E1B | na | 8165 | 0.000 | -0.1485 | No |
| 33 | CALML3 | na | 8189 | 0.000 | -0.1496 | No |
| 34 | PPP1R3C | na | 8368 | 0.000 | -0.1578 | No |
| 35 | PPP1R3B | na | 8369 | 0.000 | -0.1578 | No |
| 36 | PPP1R3A | na | 8371 | 0.000 | -0.1578 | No |
| 37 | G6PC2 | na | 9932 | 0.000 | -0.2296 | No |
| 38 | SHC3 | na | 10832 | 0.000 | -0.2709 | No |
| 39 | FBP2 | na | 11004 | 0.000 | -0.2788 | No |
| 40 | IRS4 | na | 12166 | 0.000 | -0.3322 | No |
| 41 | HK3 | na | 13324 | -0.002 | -0.3853 | No |
| 42 | PKLR | na | 13410 | -0.006 | -0.3891 | No |
| 43 | PDE3A | na | 13506 | -0.007 | -0.3933 | No |
| 44 | GYS2 | na | 13829 | -0.015 | -0.4076 | No |
| 45 | MKNK1 | na | 13934 | -0.018 | -0.4119 | No |
| 46 | LIPE | na | 13968 | -0.019 | -0.4129 | No |
| 47 | PYGL | na | 14303 | -0.032 | -0.4274 | No |
| 48 | CALM3 | na | 14729 | -0.052 | -0.4455 | No |
| 49 | PHKG2 | na | 14799 | -0.056 | -0.4471 | No |
| 50 | TRIP10 | na | 14817 | -0.057 | -0.4463 | No |
| 51 | RHOQ | na | 14853 | -0.059 | -0.4463 | No |
| 52 | PRKAR1A | na | 14987 | -0.067 | -0.4506 | No |
| 53 | PPP1CB | na | 15011 | -0.068 | -0.4497 | No |
| 54 | MAP2K1 | na | 15179 | -0.079 | -0.4552 | No |
| 55 | INPP5K | na | 15497 | -0.097 | -0.4671 | No |
| 56 | PYGM | na | 15676 | -0.107 | -0.4723 | No |
| 57 | RPS6KB2 | na | 15831 | -0.117 | -0.4762 | No |
| 58 | ARAF | na | 15889 | -0.121 | -0.4754 | No |
| 59 | PRKACA | na | 15891 | -0.121 | -0.4721 | No |
| 60 | PRKAB1 | na | 15917 | -0.123 | -0.4699 | No |
| 61 | PPP1CC | na | 16090 | -0.134 | -0.4741 | No |
| 62 | MAPK10 | na | 16183 | -0.140 | -0.4745 | No |
| 63 | PRKAR2B | na | 16190 | -0.140 | -0.4709 | No |
| 64 | MAPK1 | na | 16234 | -0.143 | -0.4689 | No |
| 65 | PDE3B | na | 16298 | -0.147 | -0.4677 | No |
| 66 | FLOT2 | na | 16366 | -0.152 | -0.4666 | No |
| 67 | AKT2 | na | 16784 | -0.181 | -0.4807 | Yes |
| 68 | IKBKB | na | 16790 | -0.182 | -0.4759 | Yes |
| 69 | AKT1 | na | 16827 | -0.184 | -0.4725 | Yes |
| 70 | SOCS3 | na | 16838 | -0.185 | -0.4678 | Yes |
| 71 | CRK | na | 16856 | -0.186 | -0.4634 | Yes |
| 72 | SORBS1 | na | 17043 | -0.198 | -0.4665 | Yes |
| 73 | PTPN1 | na | 17059 | -0.199 | -0.4617 | Yes |
| 74 | PYGB | na | 17183 | -0.208 | -0.4616 | Yes |
| 75 | EXOC7 | na | 17201 | -0.209 | -0.4566 | Yes |
| 76 | RAF1 | na | 17251 | -0.212 | -0.4529 | Yes |
| 77 | RPS6KB1 | na | 17275 | -0.214 | -0.4481 | Yes |
| 78 | PRKAA1 | na | 17528 | -0.232 | -0.4532 | Yes |
| 79 | SHC4 | na | 17550 | -0.233 | -0.4477 | Yes |
| 80 | HK1 | na | 17572 | -0.235 | -0.4422 | Yes |
| 81 | PRKAG2 | na | 17803 | -0.253 | -0.4458 | Yes |
| 82 | MAPK8 | na | 17866 | -0.258 | -0.4415 | Yes |
| 83 | AKT3 | na | 17899 | -0.261 | -0.4357 | Yes |
| 84 | HK2 | na | 18028 | -0.271 | -0.4341 | Yes |
| 85 | ACACB | na | 18119 | -0.278 | -0.4305 | Yes |
| 86 | PIK3CD | na | 18197 | -0.284 | -0.4262 | Yes |
| 87 | PRKAB2 | na | 18245 | -0.288 | -0.4204 | Yes |
| 88 | SREBF1 | na | 18341 | -0.294 | -0.4166 | Yes |
| 89 | PIK3R2 | na | 18436 | -0.299 | -0.4127 | Yes |
| 90 | PIK3CA | na | 18513 | -0.305 | -0.4077 | Yes |
| 91 | PRKX | na | 18548 | -0.308 | -0.4008 | Yes |
| 92 | TSC1 | na | 18564 | -0.309 | -0.3929 | Yes |
| 93 | GYS1 | na | 18792 | -0.329 | -0.3942 | Yes |
| 94 | PHKB | na | 18952 | -0.344 | -0.3920 | Yes |
| 95 | PPARGC1A | na | 19078 | -0.357 | -0.3879 | Yes |
| 96 | SHC1 | na | 19109 | -0.361 | -0.3793 | Yes |
| 97 | PIK3R3 | na | 19336 | -0.380 | -0.3791 | Yes |
| 98 | SLC2A4 | na | 19402 | -0.388 | -0.3714 | Yes |
| 99 | MKNK2 | na | 19425 | -0.390 | -0.3616 | Yes |
| 100 | CRKL | na | 19431 | -0.390 | -0.3510 | Yes |
| 101 | PRKACB | na | 19453 | -0.392 | -0.3411 | Yes |
| 102 | PIK3R5 | na | 19509 | -0.397 | -0.3327 | Yes |
| 103 | PRKCI | na | 19552 | -0.402 | -0.3235 | Yes |
| 104 | PHKA2 | na | 19599 | -0.406 | -0.3143 | Yes |
| 105 | SOS2 | na | 19626 | -0.409 | -0.3042 | Yes |
| 106 | ELK1 | na | 19706 | -0.417 | -0.2963 | Yes |
| 107 | RPTOR | na | 20035 | -0.448 | -0.2990 | Yes |
| 108 | SOCS4 | na | 20052 | -0.450 | -0.2872 | Yes |
| 109 | GSK3B | na | 20096 | -0.455 | -0.2766 | Yes |
| 110 | PDPK1 | na | 20100 | -0.455 | -0.2641 | Yes |
| 111 | PHKA1 | na | 20124 | -0.458 | -0.2525 | Yes |
| 112 | CBLB | na | 20162 | -0.463 | -0.2414 | Yes |
| 113 | FOXO1 | na | 20223 | -0.469 | -0.2312 | Yes |
| 114 | TSC2 | na | 20227 | -0.470 | -0.2183 | Yes |
| 115 | PHKG1 | na | 20361 | -0.484 | -0.2110 | Yes |
| 116 | INSR | na | 20389 | -0.486 | -0.1988 | Yes |
| 117 | SH2B2 | na | 20431 | -0.493 | -0.1870 | Yes |
| 118 | RAPGEF1 | na | 20440 | -0.495 | -0.1737 | Yes |
| 119 | PIK3CB | na | 20535 | -0.507 | -0.1640 | Yes |
| 120 | SOS1 | na | 20563 | -0.511 | -0.1511 | Yes |
| 121 | IRS1 | na | 20589 | -0.515 | -0.1380 | Yes |
| 122 | PRKAR2A | na | 20760 | -0.539 | -0.1309 | Yes |
| 123 | BRAF | na | 20807 | -0.547 | -0.1179 | Yes |
| 124 | FASN | na | 21113 | -0.600 | -0.1153 | Yes |
| 125 | ACACA | na | 21178 | -0.614 | -0.1012 | Yes |
| 126 | CBL | na | 21212 | -0.623 | -0.0855 | Yes |
| 127 | MTOR | na | 21338 | -0.653 | -0.0731 | Yes |
| 128 | IRS2 | na | 21348 | -0.656 | -0.0554 | Yes |
| 129 | PIK3R1 | na | 21419 | -0.674 | -0.0400 | Yes |
| 130 | PPP1R3D | na | 21432 | -0.680 | -0.0217 | Yes |
| 131 | PTPRF | na | 21489 | -0.700 | -0.0049 | Yes |
| 132 | INPP5D | na | 21674 | -0.816 | 0.0092 | Yes |
Table: GSEA details [plain text format]

  

Fig 2: KEGG\_INSULIN\_SIGNALING\_PATHWAY      
 Blue-Pink O' Gram in the Space of the Analyzed GeneSet

  

Fig 3: KEGG\_INSULIN\_SIGNALING\_PATHWAY: Random ES distribution      
 Gene set null distribution of ES for **KEGG\_INSULIN\_SIGNALING\_PATHWAY**
