# Supplementary material for: Regulation of microglia related neuroinflammation contributes to the protective effect of Gelsevirine on ischemic stroke
Source: Front Immunol. 2023 Mar 30;14:1164278. doi: 10.3389/fimmu.2023.1164278 (PMC10098192; doi:10.3389/fimmu.2023.1164278)
Supplement: Supplementary file 6 [file DataSheet_6.zip › fig 5 raw/fig 5-G raw/KEGG.Gsea.1649954846225/KEGG_METABOLISM_OF_XENOBIOTICS_BY_CYTOCHROME_P450.html]

Details for gene set KEGG\_METABOLISM\_OF\_XENOBIOTICS\_BY\_CYTOCHROME\_P450[GSEA]

|  || Dataset | OGD\_DRUG\_DRUG.OGD\_FRUG.cls#Gs\_versus\_MCAO.OGD\_FRUG.cls#Gs\_versus\_MCAO\_repos |
| Phenotype | OGD\_FRUG.cls#Gs\_versus\_MCAO\_repos |
| Upregulated in class | Gs |
| GeneSet | KEGG\_METABOLISM\_OF\_XENOBIOTICS\_BY\_CYTOCHROME\_P450 |
| Enrichment Score (ES) | 0.48762712 |
| Normalized Enrichment Score (NES) | 1.3825033 |
| Nominal p-value | 0.05706522 |
| FDR q-value | 0.19497901 |
| FWER p-Value | 0.924 |
Table: GSEA Results Summary

  

Fig 1: Enrichment plot: KEGG\_METABOLISM\_OF\_XENOBIOTICS\_BY\_CYTOCHROME\_P450      
 Profile of the Running ES Score & Positions of GeneSet Members on the Rank Ordered List

  

| SYMBOL | TITLE | RANK IN GENE LIST | RANK METRIC SCORE | RUNNING ES | CORE ENRICHMENT || 1 | MGST1 | na | 682 | 0.531 | 0.0288 | Yes |
| 2 | GSTM5 | na | 917 | 0.475 | 0.0718 | Yes |
| 3 | MGST3 | na | 1032 | 0.460 | 0.1186 | Yes |
| 4 | GSTA4 | na | 1142 | 0.443 | 0.1637 | Yes |
| 5 | ADH5 | na | 1254 | 0.428 | 0.2070 | Yes |
| 6 | GSTO2 | na | 1295 | 0.422 | 0.2529 | Yes |
| 7 | GSTO1 | na | 1917 | 0.347 | 0.2637 | Yes |
| 8 | GSTP1 | na | 1927 | 0.346 | 0.3024 | Yes |
| 9 | DHDH | na | 2056 | 0.332 | 0.3341 | Yes |
| 10 | GSTM2 | na | 2075 | 0.330 | 0.3706 | Yes |
| 11 | GSTA3 | na | 2150 | 0.321 | 0.4035 | Yes |
| 12 | ALDH3B2 | na | 2211 | 0.314 | 0.4364 | Yes |
| 13 | MGST2 | na | 2780 | 0.261 | 0.4398 | Yes |
| 14 | ALDH3B1 | na | 2884 | 0.251 | 0.4636 | Yes |
| 15 | GSTM1 | na | 2988 | 0.243 | 0.4863 | Yes |
| 16 | CYP2S1 | na | 3458 | 0.202 | 0.4876 | Yes |
| 17 | GSTZ1 | na | 3908 | 0.172 | 0.4866 | No |
| 18 | GSTK1 | na | 5170 | 0.086 | 0.4386 | No |
| 19 | GSTA2 | na | 5397 | 0.074 | 0.4366 | No |
| 20 | ADH7 | na | 5804 | 0.052 | 0.4238 | No |
| 21 | ALDH3A1 | na | 6135 | 0.035 | 0.4127 | No |
| 22 | CYP1A1 | na | 6256 | 0.030 | 0.4106 | No |
| 23 | UGT1A10 | na | 6336 | 0.027 | 0.4101 | No |
| 24 | GSTM3 | na | 7079 | 0.000 | 0.3761 | No |
| 25 | CYP1A2 | na | 7814 | 0.000 | 0.3425 | No |
| 26 | ADH4 | na | 8140 | 0.000 | 0.3276 | No |
| 27 | ALDH1A3 | na | 11926 | 0.000 | 0.1543 | No |
| 28 | UGT2A3 | na | 12403 | 0.000 | 0.1325 | No |
| 29 | UGT2A1 | na | 12405 | 0.000 | 0.1324 | No |
| 30 | UGT1A9 | na | 12599 | 0.000 | 0.1236 | No |
| 31 | GSTA1 | na | 13344 | -0.003 | 0.0898 | No |
| 32 | CYP2E1 | na | 13590 | -0.010 | 0.0797 | No |
| 33 | GSTM4 | na | 14526 | -0.042 | 0.0416 | No |
| 34 | EPHX1 | na | 14843 | -0.058 | 0.0338 | No |
| 35 | UGT1A5 | na | 15219 | -0.081 | 0.0258 | No |
| 36 | GSTT2 | na | 18427 | -0.298 | -0.0873 | No |
| 37 | CYP1B1 | na | 19841 | -0.429 | -0.1035 | No |
| 38 | UGT1A1 | na | 21720 | -0.867 | -0.0915 | No |
| 39 | GSTT1 | na | 21731 | -0.871 | 0.0066 | No |
Table: GSEA details [plain text format]

  

Fig 2: KEGG\_METABOLISM\_OF\_XENOBIOTICS\_BY\_CYTOCHROME\_P450      
 Blue-Pink O' Gram in the Space of the Analyzed GeneSet

  

Fig 3: KEGG\_METABOLISM\_OF\_XENOBIOTICS\_BY\_CYTOCHROME\_P450: Random ES distribution      
 Gene set null distribution of ES for **KEGG\_METABOLISM\_OF\_XENOBIOTICS\_BY\_CYTOCHROME\_P450**
