# Supplementary material for: Regulation of microglia related neuroinflammation contributes to the protective effect of Gelsevirine on ischemic stroke
Source: Front Immunol. 2023 Mar 30;14:1164278. doi: 10.3389/fimmu.2023.1164278 (PMC10098192; doi:10.3389/fimmu.2023.1164278)
Supplement: Supplementary file 6 [file DataSheet_6.zip › fig 5 raw/fig 5-G raw/KEGG.Gsea.1649954846225/KEGG_NON_SMALL_CELL_LUNG_CANCER.html]

Details for gene set KEGG\_NON\_SMALL\_CELL\_LUNG\_CANCER[GSEA]

|  || Dataset | OGD\_DRUG\_DRUG.OGD\_FRUG.cls#Gs\_versus\_MCAO.OGD\_FRUG.cls#Gs\_versus\_MCAO\_repos |
| Phenotype | OGD\_FRUG.cls#Gs\_versus\_MCAO\_repos |
| Upregulated in class | MCAO |
| GeneSet | KEGG\_NON\_SMALL\_CELL\_LUNG\_CANCER |
| Enrichment Score (ES) | -0.5579094 |
| Normalized Enrichment Score (NES) | -1.5296873 |
| Nominal p-value | 0.009090909 |
| FDR q-value | 0.053991843 |
| FWER p-Value | 0.678 |
Table: GSEA Results Summary

  

Fig 1: Enrichment plot: KEGG\_NON\_SMALL\_CELL\_LUNG\_CANCER      
 Profile of the Running ES Score & Positions of GeneSet Members on the Rank Ordered List

  

| SYMBOL | TITLE | RANK IN GENE LIST | RANK METRIC SCORE | RUNNING ES | CORE ENRICHMENT || 1 | PIK3CG | na | 1248 | 0.428 | -0.0254 | No |
| 2 | HRAS | na | 2716 | 0.268 | -0.0727 | No |
| 3 | CDK4 | na | 3240 | 0.219 | -0.0804 | No |
| 4 | TGFA | na | 3523 | 0.198 | -0.0786 | No |
| 5 | BAD | na | 3658 | 0.189 | -0.0707 | No |
| 6 | RASSF1 | na | 4118 | 0.155 | -0.0803 | No |
| 7 | PRKCG | na | 4221 | 0.148 | -0.0740 | No |
| 8 | NRAS | na | 4669 | 0.117 | -0.0858 | No |
| 9 | MAP2K2 | na | 4692 | 0.115 | -0.0782 | No |
| 10 | MAPK3 | na | 4918 | 0.100 | -0.0811 | No |
| 11 | PLCG2 | na | 5215 | 0.084 | -0.0884 | No |
| 12 | GRB2 | na | 5726 | 0.057 | -0.1076 | No |
| 13 | KRAS | na | 6543 | 0.019 | -0.1436 | No |
| 14 | RASSF5 | na | 7015 | 0.000 | -0.1652 | No |
| 15 | RXRG | na | 8397 | 0.000 | -0.2285 | No |
| 16 | CDKN2A | na | 10240 | 0.000 | -0.3129 | No |
| 17 | PRKCB | na | 13570 | -0.009 | -0.4647 | No |
| 18 | RXRB | na | 14636 | -0.047 | -0.5100 | No |
| 19 | FHIT | na | 14852 | -0.059 | -0.5155 | No |
| 20 | MAP2K1 | na | 15179 | -0.079 | -0.5245 | No |
| 21 | E2F1 | na | 15769 | -0.113 | -0.5431 | No |
| 22 | ARAF | na | 15889 | -0.121 | -0.5396 | No |
| 23 | RARB | na | 16048 | -0.131 | -0.5371 | No |
| 24 | MAPK1 | na | 16234 | -0.143 | -0.5350 | No |
| 25 | E2F3 | na | 16736 | -0.179 | -0.5446 | Yes |
| 26 | AKT2 | na | 16784 | -0.181 | -0.5333 | Yes |
| 27 | RB1 | na | 16796 | -0.182 | -0.5203 | Yes |
| 28 | AKT1 | na | 16827 | -0.184 | -0.5080 | Yes |
| 29 | RAF1 | na | 17251 | -0.212 | -0.5116 | Yes |
| 30 | CCND1 | na | 17421 | -0.225 | -0.5027 | Yes |
| 31 | E2F2 | na | 17853 | -0.258 | -0.5033 | Yes |
| 32 | AKT3 | na | 17899 | -0.261 | -0.4859 | Yes |
| 33 | PIK3CD | na | 18197 | -0.284 | -0.4785 | Yes |
| 34 | PRKCA | na | 18221 | -0.286 | -0.4583 | Yes |
| 35 | PIK3R2 | na | 18436 | -0.299 | -0.4459 | Yes |
| 36 | PIK3CA | na | 18513 | -0.305 | -0.4267 | Yes |
| 37 | FOXO3 | na | 19023 | -0.351 | -0.4240 | Yes |
| 38 | EGFR | na | 19043 | -0.354 | -0.3986 | Yes |
| 39 | RXRA | na | 19209 | -0.370 | -0.3786 | Yes |
| 40 | CASP9 | na | 19243 | -0.373 | -0.3525 | Yes |
| 41 | STK4 | na | 19284 | -0.376 | -0.3264 | Yes |
| 42 | PIK3R3 | na | 19336 | -0.380 | -0.3004 | Yes |
| 43 | PIK3R5 | na | 19509 | -0.397 | -0.2788 | Yes |
| 44 | SOS2 | na | 19626 | -0.409 | -0.2538 | Yes |
| 45 | PDPK1 | na | 20100 | -0.455 | -0.2416 | Yes |
| 46 | EGF | na | 20244 | -0.471 | -0.2132 | Yes |
| 47 | ERBB2 | na | 20414 | -0.490 | -0.1845 | Yes |
| 48 | PIK3CB | na | 20535 | -0.507 | -0.1524 | Yes |
| 49 | SOS1 | na | 20563 | -0.511 | -0.1158 | Yes |
| 50 | CDK6 | na | 20612 | -0.518 | -0.0795 | Yes |
| 51 | BRAF | na | 20807 | -0.547 | -0.0478 | Yes |
| 52 | PLCG1 | na | 21230 | -0.627 | -0.0206 | Yes |
| 53 | PIK3R1 | na | 21419 | -0.674 | 0.0209 | Yes |
Table: GSEA details [plain text format]

  

Fig 2: KEGG\_NON\_SMALL\_CELL\_LUNG\_CANCER      
 Blue-Pink O' Gram in the Space of the Analyzed GeneSet

  

Fig 3: KEGG\_NON\_SMALL\_CELL\_LUNG\_CANCER: Random ES distribution      
 Gene set null distribution of ES for **KEGG\_NON\_SMALL\_CELL\_LUNG\_CANCER**
