# Supplementary material for: Regulation of microglia related neuroinflammation contributes to the protective effect of Gelsevirine on ischemic stroke
Source: Front Immunol. 2023 Mar 30;14:1164278. doi: 10.3389/fimmu.2023.1164278 (PMC10098192; doi:10.3389/fimmu.2023.1164278)
Supplement: Supplementary file 6 [file DataSheet_6.zip › fig 5 raw/fig 5-G raw/KEGG.Gsea.1649954846225/KEGG_OXIDATIVE_PHOSPHORYLATION.html]

Details for gene set KEGG\_OXIDATIVE\_PHOSPHORYLATION[GSEA]

|  || Dataset | OGD\_DRUG\_DRUG.OGD\_FRUG.cls#Gs\_versus\_MCAO.OGD\_FRUG.cls#Gs\_versus\_MCAO\_repos |
| Phenotype | OGD\_FRUG.cls#Gs\_versus\_MCAO\_repos |
| Upregulated in class | Gs |
| GeneSet | KEGG\_OXIDATIVE\_PHOSPHORYLATION |
| Enrichment Score (ES) | 0.7205632 |
| Normalized Enrichment Score (NES) | 2.3971603 |
| Nominal p-value | 0.0 |
| FDR q-value | 0.0 |
| FWER p-Value | 0.0 |
Table: GSEA Results Summary

  

Fig 1: Enrichment plot: KEGG\_OXIDATIVE\_PHOSPHORYLATION      
 Profile of the Running ES Score & Positions of GeneSet Members on the Rank Ordered List

  

| SYMBOL | TITLE | RANK IN GENE LIST | RANK METRIC SCORE | RUNNING ES | CORE ENRICHMENT || 1 | COX6A2 | na | 162 | 0.836 | 0.0189 | Yes |
| 2 | UQCRB | na | 430 | 0.620 | 0.0262 | Yes |
| 3 | COX6C | na | 440 | 0.616 | 0.0452 | Yes |
| 4 | COX7A2 | na | 449 | 0.612 | 0.0642 | Yes |
| 5 | COX5A | na | 462 | 0.602 | 0.0826 | Yes |
| 6 | NDUFA4 | na | 469 | 0.600 | 0.1012 | Yes |
| 7 | NDUFC1 | na | 493 | 0.590 | 0.1188 | Yes |
| 8 | UQCRH | na | 503 | 0.588 | 0.1369 | Yes |
| 9 | NDUFA2 | na | 510 | 0.586 | 0.1551 | Yes |
| 10 | NDUFB6 | na | 567 | 0.565 | 0.1704 | Yes |
| 11 | COX6B1 | na | 653 | 0.538 | 0.1834 | Yes |
| 12 | NDUFA1 | na | 663 | 0.536 | 0.1999 | Yes |
| 13 | NDUFS4 | na | 674 | 0.532 | 0.2162 | Yes |
| 14 | NDUFA11 | na | 697 | 0.527 | 0.2318 | Yes |
| 15 | ATP6V1G1 | na | 733 | 0.517 | 0.2465 | Yes |
| 16 | NDUFA4L2 | na | 786 | 0.501 | 0.2599 | Yes |
| 17 | NDUFC2 | na | 793 | 0.500 | 0.2754 | Yes |
| 18 | UQCR10 | na | 800 | 0.498 | 0.2908 | Yes |
| 19 | NDUFB5 | na | 819 | 0.494 | 0.3056 | Yes |
| 20 | COX7B | na | 859 | 0.484 | 0.3191 | Yes |
| 21 | NDUFA5 | na | 1040 | 0.459 | 0.3253 | Yes |
| 22 | ATP6V1F | na | 1110 | 0.447 | 0.3362 | Yes |
| 23 | COX7C | na | 1133 | 0.444 | 0.3492 | Yes |
| 24 | COX17 | na | 1153 | 0.441 | 0.3622 | Yes |
| 25 | NDUFB3 | na | 1166 | 0.439 | 0.3755 | Yes |
| 26 | COX4I1 | na | 1200 | 0.434 | 0.3877 | Yes |
| 27 | NDUFS6 | na | 1280 | 0.424 | 0.3974 | Yes |
| 28 | COX6A1 | na | 1296 | 0.422 | 0.4100 | Yes |
| 29 | COX7A2L | na | 1310 | 0.419 | 0.4226 | Yes |
| 30 | COX8A | na | 1327 | 0.417 | 0.4350 | Yes |
| 31 | NDUFS5 | na | 1350 | 0.415 | 0.4471 | Yes |
| 32 | NDUFB10 | na | 1372 | 0.413 | 0.4592 | Yes |
| 33 | NDUFB9 | na | 1449 | 0.405 | 0.4685 | Yes |
| 34 | ATP6V1E1 | na | 1450 | 0.405 | 0.4813 | Yes |
| 35 | NDUFB4 | na | 1457 | 0.405 | 0.4938 | Yes |
| 36 | NDUFV2 | na | 1503 | 0.400 | 0.5043 | Yes |
| 37 | PPA2 | na | 1579 | 0.390 | 0.5132 | Yes |
| 38 | ATP6V0B | na | 1590 | 0.389 | 0.5250 | Yes |
| 39 | NDUFS3 | na | 1637 | 0.382 | 0.5349 | Yes |
| 40 | NDUFB7 | na | 1789 | 0.363 | 0.5394 | Yes |
| 41 | UQCRQ | na | 1834 | 0.358 | 0.5487 | Yes |
| 42 | COX7A1 | na | 1863 | 0.354 | 0.5586 | Yes |
| 43 | NDUFA3 | na | 1881 | 0.352 | 0.5689 | Yes |
| 44 | ATP6V1H | na | 1896 | 0.349 | 0.5793 | Yes |
| 45 | NDUFB2 | na | 1907 | 0.348 | 0.5898 | Yes |
| 46 | NDUFA7 | na | 1971 | 0.341 | 0.5977 | Yes |
| 47 | NDUFA8 | na | 2185 | 0.318 | 0.5979 | Yes |
| 48 | UQCRFS1 | na | 2202 | 0.315 | 0.6071 | Yes |
| 49 | COX5B | na | 2237 | 0.311 | 0.6154 | Yes |
| 50 | ATP6V1D | na | 2307 | 0.303 | 0.6217 | Yes |
| 51 | CYC1 | na | 2402 | 0.296 | 0.6267 | Yes |
| 52 | NDUFAB1 | na | 2476 | 0.289 | 0.6325 | Yes |
| 53 | COX11 | na | 2539 | 0.283 | 0.6386 | Yes |
| 54 | ATP6V0D1 | na | 2573 | 0.281 | 0.6459 | Yes |
| 55 | SDHD | na | 2574 | 0.281 | 0.6548 | Yes |
| 56 | NDUFA9 | na | 2575 | 0.281 | 0.6636 | Yes |
| 57 | UQCR11 | na | 2718 | 0.268 | 0.6655 | Yes |
| 58 | NDUFS7 | na | 2745 | 0.265 | 0.6727 | Yes |
| 59 | ATP6V1C1 | na | 2830 | 0.257 | 0.6769 | Yes |
| 60 | NDUFA10 | na | 2863 | 0.254 | 0.6835 | Yes |
| 61 | PPA1 | na | 2866 | 0.254 | 0.6914 | Yes |
| 62 | NDUFV3 | na | 2869 | 0.253 | 0.6993 | Yes |
| 63 | SDHC | na | 2894 | 0.251 | 0.7061 | Yes |
| 64 | UQCRC2 | na | 3080 | 0.235 | 0.7050 | Yes |
| 65 | ATP6V0A4 | na | 3112 | 0.232 | 0.7108 | Yes |
| 66 | NDUFS8 | na | 3269 | 0.217 | 0.7105 | Yes |
| 67 | NDUFA6 | na | 3303 | 0.214 | 0.7158 | Yes |
| 68 | ATP6V0C | na | 3364 | 0.209 | 0.7196 | Yes |
| 69 | SDHB | na | 3526 | 0.198 | 0.7184 | Yes |
| 70 | NDUFS2 | na | 3682 | 0.187 | 0.7172 | Yes |
| 71 | NDUFB8 | na | 3736 | 0.183 | 0.7206 | Yes |
| 72 | COX7B2 | na | 4007 | 0.163 | 0.7133 | No |
| 73 | ATP6V1B1 | na | 4550 | 0.125 | 0.6924 | No |
| 74 | NDUFV1 | na | 4574 | 0.124 | 0.6952 | No |
| 75 | UQCRC1 | na | 4897 | 0.101 | 0.6836 | No |
| 76 | ATP4A | na | 6396 | 0.025 | 0.6156 | No |
| 77 | COX10 | na | 6450 | 0.023 | 0.6139 | No |
| 78 | ATP6V0D2 | na | 6863 | 0.006 | 0.5952 | No |
| 79 | ATP6V1G3 | na | 8088 | 0.000 | 0.5390 | No |
| 80 | ATP4B | na | 11342 | 0.000 | 0.3896 | No |
| 81 | COX8C | na | 11687 | 0.000 | 0.3738 | No |
| 82 | COX6B2 | na | 13632 | -0.011 | 0.2849 | No |
| 83 | ATP6AP1 | na | 13950 | -0.019 | 0.2709 | No |
| 84 | NDUFS1 | na | 14405 | -0.036 | 0.2512 | No |
| 85 | COX4I2 | na | 14503 | -0.041 | 0.2480 | No |
| 86 | SDHA | na | 14573 | -0.044 | 0.2462 | No |
| 87 | ATP6V1B2 | na | 14758 | -0.054 | 0.2395 | No |
| 88 | ATP6V1A | na | 15638 | -0.105 | 0.2025 | No |
| 89 | ATP6V1C2 | na | 16049 | -0.131 | 0.1878 | No |
| 90 | ATP6V0E2 | na | 16059 | -0.132 | 0.1915 | No |
| 91 | TCIRG1 | na | 16613 | -0.170 | 0.1715 | No |
| 92 | ATP6V0A2 | na | 17054 | -0.199 | 0.1576 | No |
| 93 | ATP12A | na | 17171 | -0.207 | 0.1588 | No |
| 94 | ATP6V1G2 | na | 17985 | -0.268 | 0.1299 | No |
| 95 | ATP6V1E2 | na | 18706 | -0.321 | 0.1070 | No |
| 96 | LHPP | na | 18964 | -0.345 | 0.1061 | No |
| 97 | COX15 | na | 19852 | -0.431 | 0.0789 | No |
| 98 | ATP6V0A1 | na | 19973 | -0.442 | 0.0873 | No |
Table: GSEA details [plain text format]

  

Fig 2: KEGG\_OXIDATIVE\_PHOSPHORYLATION      
 Blue-Pink O' Gram in the Space of the Analyzed GeneSet

  

Fig 3: KEGG\_OXIDATIVE\_PHOSPHORYLATION: Random ES distribution      
 Gene set null distribution of ES for **KEGG\_OXIDATIVE\_PHOSPHORYLATION**
