# Supplementary material for: Regulation of microglia related neuroinflammation contributes to the protective effect of Gelsevirine on ischemic stroke
Source: Front Immunol. 2023 Mar 30;14:1164278. doi: 10.3389/fimmu.2023.1164278 (PMC10098192; doi:10.3389/fimmu.2023.1164278)
Supplement: Supplementary file 6 [file DataSheet_6.zip › fig 5 raw/fig 5-G raw/KEGG.Gsea.1649954846225/KEGG_PARKINSONS_DISEASE.html]

Details for gene set KEGG\_PARKINSONS\_DISEASE[GSEA]

|  || Dataset | OGD\_DRUG\_DRUG.OGD\_FRUG.cls#Gs\_versus\_MCAO.OGD\_FRUG.cls#Gs\_versus\_MCAO\_repos |
| Phenotype | OGD\_FRUG.cls#Gs\_versus\_MCAO\_repos |
| Upregulated in class | Gs |
| GeneSet | KEGG\_PARKINSONS\_DISEASE |
| Enrichment Score (ES) | 0.7234413 |
| Normalized Enrichment Score (NES) | 2.3678558 |
| Nominal p-value | 0.0 |
| FDR q-value | 0.0 |
| FWER p-Value | 0.0 |
Table: GSEA Results Summary

  

Fig 1: Enrichment plot: KEGG\_PARKINSONS\_DISEASE      
 Profile of the Running ES Score & Positions of GeneSet Members on the Rank Ordered List

  

| SYMBOL | TITLE | RANK IN GENE LIST | RANK METRIC SCORE | RUNNING ES | CORE ENRICHMENT || 1 | COX6A2 | na | 162 | 0.836 | 0.0208 | Yes |
| 2 | UQCRB | na | 430 | 0.620 | 0.0295 | Yes |
| 3 | COX6C | na | 440 | 0.616 | 0.0499 | Yes |
| 4 | COX7A2 | na | 449 | 0.612 | 0.0703 | Yes |
| 5 | COX5A | na | 462 | 0.602 | 0.0901 | Yes |
| 6 | NDUFA4 | na | 469 | 0.600 | 0.1101 | Yes |
| 7 | NDUFC1 | na | 493 | 0.590 | 0.1290 | Yes |
| 8 | UQCRH | na | 503 | 0.588 | 0.1485 | Yes |
| 9 | NDUFA2 | na | 510 | 0.586 | 0.1680 | Yes |
| 10 | NDUFB6 | na | 567 | 0.565 | 0.1845 | Yes |
| 11 | COX6B1 | na | 653 | 0.538 | 0.1988 | Yes |
| 12 | NDUFA1 | na | 663 | 0.536 | 0.2165 | Yes |
| 13 | NDUFS4 | na | 674 | 0.532 | 0.2340 | Yes |
| 14 | NDUFA4L2 | na | 786 | 0.501 | 0.2459 | Yes |
| 15 | NDUFC2 | na | 793 | 0.500 | 0.2625 | Yes |
| 16 | UQCR10 | na | 800 | 0.498 | 0.2790 | Yes |
| 17 | NDUFB5 | na | 819 | 0.494 | 0.2949 | Yes |
| 18 | COX7B | na | 859 | 0.484 | 0.3095 | Yes |
| 19 | NDUFA5 | na | 1040 | 0.459 | 0.3167 | Yes |
| 20 | VDAC3 | na | 1114 | 0.446 | 0.3285 | Yes |
| 21 | COX7C | na | 1133 | 0.444 | 0.3426 | Yes |
| 22 | NDUFB3 | na | 1166 | 0.439 | 0.3560 | Yes |
| 23 | COX4I1 | na | 1200 | 0.434 | 0.3692 | Yes |
| 24 | NDUFS6 | na | 1280 | 0.424 | 0.3799 | Yes |
| 25 | COX6A1 | na | 1296 | 0.422 | 0.3934 | Yes |
| 26 | COX7A2L | na | 1310 | 0.419 | 0.4070 | Yes |
| 27 | CYCS | na | 1320 | 0.418 | 0.4207 | Yes |
| 28 | COX8A | na | 1327 | 0.417 | 0.4345 | Yes |
| 29 | PPID | na | 1343 | 0.416 | 0.4479 | Yes |
| 30 | NDUFS5 | na | 1350 | 0.415 | 0.4617 | Yes |
| 31 | NDUFB10 | na | 1372 | 0.413 | 0.4747 | Yes |
| 32 | NDUFB9 | na | 1449 | 0.405 | 0.4849 | Yes |
| 33 | NDUFB4 | na | 1457 | 0.405 | 0.4983 | Yes |
| 34 | NDUFV2 | na | 1503 | 0.400 | 0.5097 | Yes |
| 35 | NDUFS3 | na | 1637 | 0.382 | 0.5165 | Yes |
| 36 | NDUFB7 | na | 1789 | 0.363 | 0.5219 | Yes |
| 37 | UQCRQ | na | 1834 | 0.358 | 0.5320 | Yes |
| 38 | COX7A1 | na | 1863 | 0.354 | 0.5426 | Yes |
| 39 | NDUFA3 | na | 1881 | 0.352 | 0.5538 | Yes |
| 40 | NDUFB2 | na | 1907 | 0.348 | 0.5644 | Yes |
| 41 | NDUFA7 | na | 1971 | 0.341 | 0.5730 | Yes |
| 42 | VDAC2 | na | 1982 | 0.340 | 0.5840 | Yes |
| 43 | SLC25A5 | na | 1987 | 0.339 | 0.5953 | Yes |
| 44 | SLC25A4 | na | 2006 | 0.337 | 0.6059 | Yes |
| 45 | PARK7 | na | 2064 | 0.331 | 0.6145 | Yes |
| 46 | UBE2J2 | na | 2178 | 0.320 | 0.6201 | Yes |
| 47 | NDUFA8 | na | 2185 | 0.318 | 0.6306 | Yes |
| 48 | UQCRFS1 | na | 2202 | 0.315 | 0.6405 | Yes |
| 49 | COX5B | na | 2237 | 0.311 | 0.6495 | Yes |
| 50 | CYC1 | na | 2402 | 0.296 | 0.6519 | Yes |
| 51 | NDUFAB1 | na | 2476 | 0.289 | 0.6584 | Yes |
| 52 | SDHD | na | 2574 | 0.281 | 0.6634 | Yes |
| 53 | NDUFA9 | na | 2575 | 0.281 | 0.6729 | Yes |
| 54 | UQCR11 | na | 2718 | 0.268 | 0.6754 | Yes |
| 55 | NDUFS7 | na | 2745 | 0.265 | 0.6832 | Yes |
| 56 | UBE2L3 | na | 2794 | 0.260 | 0.6897 | Yes |
| 57 | UBE2L6 | na | 2844 | 0.255 | 0.6961 | Yes |
| 58 | NDUFA10 | na | 2863 | 0.254 | 0.7039 | Yes |
| 59 | NDUFV3 | na | 2869 | 0.253 | 0.7122 | Yes |
| 60 | SDHC | na | 2894 | 0.251 | 0.7196 | Yes |
| 61 | UQCRC2 | na | 3080 | 0.235 | 0.7190 | Yes |
| 62 | NDUFS8 | na | 3269 | 0.217 | 0.7177 | Yes |
| 63 | NDUFA6 | na | 3303 | 0.214 | 0.7234 | Yes |
| 64 | SDHB | na | 3526 | 0.198 | 0.7199 | No |
| 65 | NDUFS2 | na | 3682 | 0.187 | 0.7191 | No |
| 66 | NDUFB8 | na | 3736 | 0.183 | 0.7229 | No |
| 67 | COX7B2 | na | 4007 | 0.163 | 0.7160 | No |
| 68 | NDUFV1 | na | 4574 | 0.124 | 0.6942 | No |
| 69 | HTRA2 | na | 4640 | 0.119 | 0.6953 | No |
| 70 | UQCRC1 | na | 4897 | 0.101 | 0.6869 | No |
| 71 | CASP3 | na | 5009 | 0.095 | 0.6851 | No |
| 72 | PINK1 | na | 5706 | 0.058 | 0.6551 | No |
| 73 | VDAC1 | na | 6111 | 0.036 | 0.6377 | No |
| 74 | UBE2G2 | na | 6968 | 0.002 | 0.5985 | No |
| 75 | TH | na | 7699 | 0.000 | 0.5650 | No |
| 76 | GPR37 | na | 8294 | 0.000 | 0.5377 | No |
| 77 | SLC25A31 | na | 8939 | 0.000 | 0.5081 | No |
| 78 | COX8C | na | 11687 | 0.000 | 0.3820 | No |
| 79 | SNCA | na | 11941 | 0.000 | 0.3704 | No |
| 80 | SLC6A3 | na | 12144 | 0.000 | 0.3611 | No |
| 81 | UBB | na | 13338 | -0.002 | 0.3064 | No |
| 82 | COX6B2 | na | 13632 | -0.011 | 0.2933 | No |
| 83 | SLC18A2 | na | 14112 | -0.024 | 0.2722 | No |
| 84 | UBE2G1 | na | 14329 | -0.033 | 0.2633 | No |
| 85 | NDUFS1 | na | 14405 | -0.036 | 0.2611 | No |
| 86 | COX4I2 | na | 14503 | -0.041 | 0.2580 | No |
| 87 | SDHA | na | 14573 | -0.044 | 0.2563 | No |
| 88 | UBE2J1 | na | 14909 | -0.063 | 0.2431 | No |
| 89 | UCHL1 | na | 15084 | -0.073 | 0.2376 | No |
| 90 | SNCAIP | na | 16867 | -0.187 | 0.1621 | No |
| 91 | UBA1 | na | 17163 | -0.206 | 0.1555 | No |
| 92 | SLC18A1 | na | 18435 | -0.299 | 0.1073 | No |
| 93 | UBA7 | na | 18495 | -0.303 | 0.1148 | No |
| 94 | APAF1 | na | 18709 | -0.321 | 0.1159 | No |
| 95 | CASP9 | na | 19243 | -0.373 | 0.1040 | No |
| 96 | LRRK2 | na | 20458 | -0.497 | 0.0651 | No |
Table: GSEA details [plain text format]

  

Fig 2: KEGG\_PARKINSONS\_DISEASE      
 Blue-Pink O' Gram in the Space of the Analyzed GeneSet

  

Fig 3: KEGG\_PARKINSONS\_DISEASE: Random ES distribution      
 Gene set null distribution of ES for **KEGG\_PARKINSONS\_DISEASE**
