# Supplementary material for: Regulation of microglia related neuroinflammation contributes to the protective effect of Gelsevirine on ischemic stroke
Source: Front Immunol. 2023 Mar 30;14:1164278. doi: 10.3389/fimmu.2023.1164278 (PMC10098192; doi:10.3389/fimmu.2023.1164278)
Supplement: Supplementary file 6 [file DataSheet_6.zip › fig 5 raw/fig 5-G raw/KEGG.Gsea.1649954846225/KEGG_PATHWAYS_IN_CANCER.html]

Details for gene set KEGG\_PATHWAYS\_IN\_CANCER[GSEA]

|  || Dataset | OGD\_DRUG\_DRUG.OGD\_FRUG.cls#Gs\_versus\_MCAO.OGD\_FRUG.cls#Gs\_versus\_MCAO\_repos |
| Phenotype | OGD\_FRUG.cls#Gs\_versus\_MCAO\_repos |
| Upregulated in class | MCAO |
| GeneSet | KEGG\_PATHWAYS\_IN\_CANCER |
| Enrichment Score (ES) | -0.48369706 |
| Normalized Enrichment Score (NES) | -1.606395 |
| Nominal p-value | 0.0011792453 |
| FDR q-value | 0.044936355 |
| FWER p-Value | 0.347 |
Table: GSEA Results Summary

  

Fig 1: Enrichment plot: KEGG\_PATHWAYS\_IN\_CANCER      
 Profile of the Running ES Score & Positions of GeneSet Members on the Rank Ordered List

  

| SYMBOL | TITLE | RANK IN GENE LIST | RANK METRIC SCORE | RUNNING ES | CORE ENRICHMENT || 1 | CSF2RA | na | 26 | 1.452 | 0.0167 | No |
| 2 | RAC3 | na | 194 | 0.799 | 0.0189 | No |
| 3 | MMP9 | na | 268 | 0.725 | 0.0244 | No |
| 4 | FGF17 | na | 415 | 0.625 | 0.0254 | No |
| 5 | TGFB2 | na | 485 | 0.594 | 0.0295 | No |
| 6 | LAMC3 | na | 692 | 0.529 | 0.0265 | No |
| 7 | RBX1 | na | 1016 | 0.462 | 0.0172 | No |
| 8 | BIRC5 | na | 1072 | 0.454 | 0.0203 | No |
| 9 | HSP90B1 | na | 1143 | 0.443 | 0.0225 | No |
| 10 | RALA | na | 1183 | 0.437 | 0.0261 | No |
| 11 | PIK3CG | na | 1248 | 0.428 | 0.0284 | No |
| 12 | CYCS | na | 1320 | 0.418 | 0.0303 | No |
| 13 | BAX | na | 1465 | 0.404 | 0.0286 | No |
| 14 | FOS | na | 1505 | 0.399 | 0.0318 | No |
| 15 | WNT10B | na | 1647 | 0.381 | 0.0299 | No |
| 16 | PTCH2 | na | 1709 | 0.373 | 0.0317 | No |
| 17 | CDKN1A | na | 1773 | 0.366 | 0.0333 | No |
| 18 | GSTP1 | na | 1927 | 0.346 | 0.0305 | No |
| 19 | RHOA | na | 1999 | 0.338 | 0.0314 | No |
| 20 | CKS1B | na | 2057 | 0.332 | 0.0328 | No |
| 21 | WNT2B | na | 2097 | 0.328 | 0.0351 | No |
| 22 | FGF18 | na | 2160 | 0.321 | 0.0362 | No |
| 23 | WNT4 | na | 2186 | 0.318 | 0.0389 | No |
| 24 | LAMB3 | na | 2281 | 0.306 | 0.0383 | No |
| 25 | FGF21 | na | 2349 | 0.300 | 0.0389 | No |
| 26 | LAMA3 | na | 2493 | 0.287 | 0.0359 | No |
| 27 | PDGFA | na | 2504 | 0.286 | 0.0389 | No |
| 28 | FGF11 | na | 2523 | 0.284 | 0.0416 | No |
| 29 | ITGA2B | na | 2646 | 0.275 | 0.0393 | No |
| 30 | HRAS | na | 2716 | 0.268 | 0.0395 | No |
| 31 | HDAC2 | na | 2882 | 0.251 | 0.0349 | No |
| 32 | DAPK3 | na | 3051 | 0.237 | 0.0300 | No |
| 33 | CDK4 | na | 3240 | 0.219 | 0.0240 | No |
| 34 | FGF22 | na | 3423 | 0.205 | 0.0181 | No |
| 35 | EPAS1 | na | 3465 | 0.202 | 0.0187 | No |
| 36 | TGFA | na | 3523 | 0.198 | 0.0185 | No |
| 37 | EGLN2 | na | 3607 | 0.192 | 0.0170 | No |
| 38 | BAD | na | 3658 | 0.189 | 0.0170 | No |
| 39 | LAMA4 | na | 3690 | 0.187 | 0.0179 | No |
| 40 | NFKBIA | na | 3820 | 0.179 | 0.0141 | No |
| 41 | RAC1 | na | 3821 | 0.178 | 0.0163 | No |
| 42 | MAX | na | 3996 | 0.165 | 0.0103 | No |
| 43 | MDM2 | na | 4059 | 0.159 | 0.0094 | No |
| 44 | RASSF1 | na | 4118 | 0.155 | 0.0086 | No |
| 45 | PRKCG | na | 4221 | 0.148 | 0.0057 | No |
| 46 | LAMC2 | na | 4333 | 0.141 | 0.0023 | No |
| 47 | PGF | na | 4357 | 0.139 | 0.0030 | No |
| 48 | RALB | na | 4425 | 0.134 | 0.0015 | No |
| 49 | TFG | na | 4555 | 0.125 | -0.0029 | No |
| 50 | RAD51 | na | 4570 | 0.124 | -0.0020 | No |
| 51 | NRAS | na | 4669 | 0.117 | -0.0051 | No |
| 52 | MAP2K2 | na | 4692 | 0.115 | -0.0047 | No |
| 53 | TCF7 | na | 4825 | 0.106 | -0.0095 | No |
| 54 | CDC42 | na | 4900 | 0.101 | -0.0117 | No |
| 55 | MAPK3 | na | 4918 | 0.100 | -0.0113 | No |
| 56 | BCL2L1 | na | 4927 | 0.100 | -0.0104 | No |
| 57 | CASP3 | na | 5009 | 0.095 | -0.0130 | No |
| 58 | VEGFA | na | 5039 | 0.094 | -0.0132 | No |
| 59 | PLCG2 | na | 5215 | 0.084 | -0.0203 | No |
| 60 | HSP90AB1 | na | 5242 | 0.082 | -0.0204 | No |
| 61 | HSP90AA1 | na | 5244 | 0.082 | -0.0195 | No |
| 62 | WNT7A | na | 5268 | 0.081 | -0.0195 | No |
| 63 | MITF | na | 5358 | 0.076 | -0.0227 | No |
| 64 | WNT11 | na | 5408 | 0.073 | -0.0241 | No |
| 65 | VHL | na | 5444 | 0.072 | -0.0248 | No |
| 66 | CUL2 | na | 5581 | 0.064 | -0.0304 | No |
| 67 | MYC | na | 5587 | 0.064 | -0.0298 | No |
| 68 | NOS2 | na | 5724 | 0.057 | -0.0354 | No |
| 69 | GRB2 | na | 5726 | 0.057 | -0.0348 | No |
| 70 | BIRC2 | na | 5775 | 0.053 | -0.0363 | No |
| 71 | TPM3 | na | 5818 | 0.051 | -0.0376 | No |
| 72 | CBLC | na | 5841 | 0.050 | -0.0380 | No |
| 73 | JUN | na | 5874 | 0.048 | -0.0389 | No |
| 74 | CDKN1B | na | 5942 | 0.045 | -0.0415 | No |
| 75 | SKP2 | na | 5968 | 0.043 | -0.0421 | No |
| 76 | FGF9 | na | 6059 | 0.038 | -0.0458 | No |
| 77 | NTRK1 | na | 6192 | 0.033 | -0.0515 | No |
| 78 | WNT3A | na | 6232 | 0.031 | -0.0530 | No |
| 79 | TRAF2 | na | 6244 | 0.031 | -0.0531 | No |
| 80 | WNT3 | na | 6357 | 0.026 | -0.0580 | No |
| 81 | FAS | na | 6465 | 0.023 | -0.0626 | No |
| 82 | CTNNA2 | na | 6496 | 0.021 | -0.0638 | No |
| 83 | WNT7B | na | 6508 | 0.021 | -0.0640 | No |
| 84 | CDK2 | na | 6514 | 0.020 | -0.0640 | No |
| 85 | KRAS | na | 6543 | 0.019 | -0.0651 | No |
| 86 | FGF14 | na | 6581 | 0.017 | -0.0666 | No |
| 87 | VEGFB | na | 6585 | 0.017 | -0.0665 | No |
| 88 | WNT16 | na | 6589 | 0.017 | -0.0664 | No |
| 89 | MAPK9 | na | 6752 | 0.011 | -0.0738 | No |
| 90 | ARNT2 | na | 6900 | 0.006 | -0.0806 | No |
| 91 | CTBP2 | na | 6978 | 0.002 | -0.0841 | No |
| 92 | FGF10 | na | 6998 | 0.001 | -0.0850 | No |
| 93 | RASSF5 | na | 7015 | 0.000 | -0.0857 | No |
| 94 | WNT8A | na | 7038 | 0.000 | -0.0867 | No |
| 95 | CEBPA | na | 7443 | 0.000 | -0.1055 | No |
| 96 | DCC | na | 7535 | 0.000 | -0.1097 | No |
| 97 | HGF | na | 8228 | 0.000 | -0.1418 | No |
| 98 | AXIN2 | na | 8241 | 0.000 | -0.1423 | No |
| 99 | RXRG | na | 8397 | 0.000 | -0.1495 | No |
| 100 | NKX3-1 | na | 8635 | 0.000 | -0.1605 | No |
| 101 | KIT | na | 8667 | 0.000 | -0.1620 | No |
| 102 | BMP2 | na | 9002 | 0.000 | -0.1774 | No |
| 103 | WNT10A | na | 9033 | 0.000 | -0.1788 | No |
| 104 | FZD9 | na | 9034 | 0.000 | -0.1788 | No |
| 105 | FZD8 | na | 9035 | 0.000 | -0.1788 | No |
| 106 | WNT9B | na | 9177 | 0.000 | -0.1854 | No |
| 107 | WNT8B | na | 9179 | 0.000 | -0.1854 | No |
| 108 | WNT6 | na | 9610 | 0.000 | -0.2054 | No |
| 109 | WNT2 | na | 9613 | 0.000 | -0.2055 | No |
| 110 | SHH | na | 9875 | 0.000 | -0.2176 | No |
| 111 | RAC2 | na | 10018 | 0.000 | -0.2241 | No |
| 112 | FGF20 | na | 10078 | 0.000 | -0.2269 | No |
| 113 | FGF23 | na | 10079 | 0.000 | -0.2269 | No |
| 114 | FGF16 | na | 10085 | 0.000 | -0.2271 | No |
| 115 | FGF13 | na | 10092 | 0.000 | -0.2274 | No |
| 116 | CDKN2B | na | 10239 | 0.000 | -0.2342 | No |
| 117 | CDKN2A | na | 10240 | 0.000 | -0.2342 | No |
| 118 | CCNA1 | na | 10605 | 0.000 | -0.2510 | No |
| 119 | HHIP | na | 10864 | 0.000 | -0.2630 | No |
| 120 | VEGFC | na | 10945 | 0.000 | -0.2667 | No |
| 121 | FZD10 | na | 11261 | 0.000 | -0.2813 | No |
| 122 | FGF3 | na | 11852 | 0.000 | -0.3087 | No |
| 123 | FGF4 | na | 11853 | 0.000 | -0.3087 | No |
| 124 | FGF5 | na | 11854 | 0.000 | -0.3087 | No |
| 125 | GLI2 | na | 12145 | 0.000 | -0.3221 | No |
| 126 | CSF3R | na | 12165 | 0.000 | -0.3230 | No |
| 127 | ITGA2 | na | 12406 | 0.000 | -0.3342 | No |
| 128 | CCNE2 | na | 13270 | -0.001 | -0.3742 | No |
| 129 | WNT9A | na | 13355 | -0.003 | -0.3780 | No |
| 130 | ZBTB16 | na | 13418 | -0.006 | -0.3808 | No |
| 131 | FGFR2 | na | 13468 | -0.007 | -0.3830 | No |
| 132 | AR | na | 13568 | -0.009 | -0.3875 | No |
| 133 | PRKCB | na | 13570 | -0.009 | -0.3874 | No |
| 134 | LAMA1 | na | 13573 | -0.009 | -0.3874 | No |
| 135 | RET | na | 13693 | -0.012 | -0.3928 | No |
| 136 | FLT3 | na | 13729 | -0.013 | -0.3942 | No |
| 137 | FGF2 | na | 13903 | -0.017 | -0.4021 | No |
| 138 | MSH2 | na | 14302 | -0.032 | -0.4201 | No |
| 139 | VEGFD | na | 14442 | -0.038 | -0.4261 | No |
| 140 | SMAD2 | na | 14505 | -0.041 | -0.4285 | No |
| 141 | RXRB | na | 14636 | -0.047 | -0.4339 | No |
| 142 | SMAD4 | na | 14652 | -0.048 | -0.4340 | No |
| 143 | IKBKG | na | 14695 | -0.050 | -0.4353 | No |
| 144 | BID | na | 14796 | -0.056 | -0.4393 | No |
| 145 | FGF8 | na | 14837 | -0.058 | -0.4404 | No |
| 146 | FGF6 | na | 15000 | -0.068 | -0.4471 | No |
| 147 | EGLN1 | na | 15036 | -0.070 | -0.4479 | No |
| 148 | WNT1 | na | 15037 | -0.070 | -0.4470 | No |
| 149 | TRAF1 | na | 15135 | -0.076 | -0.4505 | No |
| 150 | MAP2K1 | na | 15179 | -0.079 | -0.4516 | No |
| 151 | MSH3 | na | 15250 | -0.083 | -0.4538 | No |
| 152 | RALBP1 | na | 15377 | -0.091 | -0.4585 | No |
| 153 | DAPK2 | na | 15489 | -0.097 | -0.4625 | No |
| 154 | CASP8 | na | 15511 | -0.098 | -0.4622 | No |
| 155 | TRAF4 | na | 15537 | -0.100 | -0.4621 | No |
| 156 | RELA | na | 15584 | -0.103 | -0.4630 | No |
| 157 | PIAS2 | na | 15673 | -0.107 | -0.4657 | No |
| 158 | CTBP1 | na | 15701 | -0.109 | -0.4657 | No |
| 159 | E2F1 | na | 15769 | -0.113 | -0.4674 | No |
| 160 | HDAC1 | na | 15789 | -0.114 | -0.4668 | No |
| 161 | RALGDS | na | 15860 | -0.119 | -0.4686 | No |
| 162 | PIAS4 | na | 15887 | -0.121 | -0.4683 | No |
| 163 | ARAF | na | 15889 | -0.121 | -0.4669 | No |
| 164 | FADD | na | 15939 | -0.125 | -0.4676 | No |
| 165 | RARB | na | 16048 | -0.131 | -0.4710 | No |
| 166 | PAX8 | na | 16102 | -0.134 | -0.4718 | No |
| 167 | EGLN3 | na | 16115 | -0.136 | -0.4707 | No |
| 168 | MAPK10 | na | 16183 | -0.140 | -0.4721 | No |
| 169 | TGFB1 | na | 16186 | -0.140 | -0.4704 | No |
| 170 | MAPK1 | na | 16234 | -0.143 | -0.4708 | No |
| 171 | SLC2A1 | na | 16405 | -0.155 | -0.4768 | No |
| 172 | SPI1 | na | 16429 | -0.156 | -0.4760 | No |
| 173 | PTGS2 | na | 16449 | -0.158 | -0.4749 | No |
| 174 | PTK2 | na | 16640 | -0.173 | -0.4816 | Yes |
| 175 | ITGB1 | na | 16661 | -0.174 | -0.4803 | Yes |
| 176 | RARA | na | 16711 | -0.178 | -0.4804 | Yes |
| 177 | CCNE1 | na | 16729 | -0.178 | -0.4790 | Yes |
| 178 | E2F3 | na | 16736 | -0.179 | -0.4771 | Yes |
| 179 | LEF1 | na | 16773 | -0.181 | -0.4765 | Yes |
| 180 | AKT2 | na | 16784 | -0.181 | -0.4747 | Yes |
| 181 | IKBKB | na | 16790 | -0.182 | -0.4727 | Yes |
| 182 | RB1 | na | 16796 | -0.182 | -0.4707 | Yes |
| 183 | WNT5B | na | 16806 | -0.183 | -0.4689 | Yes |
| 184 | AKT1 | na | 16827 | -0.184 | -0.4675 | Yes |
| 185 | RUNX1 | na | 16837 | -0.185 | -0.4657 | Yes |
| 186 | CRK | na | 16856 | -0.186 | -0.4642 | Yes |
| 187 | APPL1 | na | 16863 | -0.187 | -0.4622 | Yes |
| 188 | TCF7L1 | na | 16939 | -0.192 | -0.4633 | Yes |
| 189 | BMP4 | na | 17035 | -0.198 | -0.4652 | Yes |
| 190 | TRAF5 | na | 17214 | -0.210 | -0.4709 | Yes |
| 191 | PLD1 | na | 17249 | -0.212 | -0.4698 | Yes |
| 192 | RAF1 | na | 17251 | -0.212 | -0.4673 | Yes |
| 193 | BIRC3 | na | 17295 | -0.216 | -0.4666 | Yes |
| 194 | PTEN | na | 17342 | -0.220 | -0.4660 | Yes |
| 195 | CTNNA1 | na | 17378 | -0.222 | -0.4649 | Yes |
| 196 | CCND1 | na | 17421 | -0.225 | -0.4640 | Yes |
| 197 | FZD2 | na | 17458 | -0.227 | -0.4629 | Yes |
| 198 | DVL2 | na | 17478 | -0.229 | -0.4610 | Yes |
| 199 | FGF7 | na | 17518 | -0.231 | -0.4599 | Yes |
| 200 | BCL2 | na | 17522 | -0.232 | -0.4572 | Yes |
| 201 | PDGFB | na | 17563 | -0.234 | -0.4561 | Yes |
| 202 | CSF1R | na | 17613 | -0.238 | -0.4555 | Yes |
| 203 | CHUK | na | 17623 | -0.238 | -0.4530 | Yes |
| 204 | MSH6 | na | 17651 | -0.241 | -0.4512 | Yes |
| 205 | PIAS1 | na | 17735 | -0.247 | -0.4520 | Yes |
| 206 | IGF1 | na | 17740 | -0.247 | -0.4492 | Yes |
| 207 | PPARD | na | 17742 | -0.248 | -0.4461 | Yes |
| 208 | STAT1 | na | 17788 | -0.252 | -0.4451 | Yes |
| 209 | STK36 | na | 17807 | -0.254 | -0.4428 | Yes |
| 210 | E2F2 | na | 17853 | -0.258 | -0.4417 | Yes |
| 211 | FZD3 | na | 17855 | -0.258 | -0.4386 | Yes |
| 212 | MAPK8 | na | 17866 | -0.258 | -0.4359 | Yes |
| 213 | DVL1 | na | 17888 | -0.261 | -0.4336 | Yes |
| 214 | AKT3 | na | 17899 | -0.261 | -0.4308 | Yes |
| 215 | FGFR1 | na | 18053 | -0.273 | -0.4346 | Yes |
| 216 | MMP2 | na | 18057 | -0.273 | -0.4313 | Yes |
| 217 | AXIN1 | na | 18098 | -0.276 | -0.4298 | Yes |
| 218 | CDH1 | na | 18147 | -0.281 | -0.4285 | Yes |
| 219 | PIK3CD | na | 18197 | -0.284 | -0.4273 | Yes |
| 220 | PRKCA | na | 18221 | -0.286 | -0.4248 | Yes |
| 221 | CTNNB1 | na | 18285 | -0.290 | -0.4242 | Yes |
| 222 | PIK3R2 | na | 18436 | -0.299 | -0.4274 | Yes |
| 223 | LAMB1 | na | 18455 | -0.300 | -0.4245 | Yes |
| 224 | PIK3CA | na | 18513 | -0.305 | -0.4234 | Yes |
| 225 | TRAF6 | na | 18711 | -0.321 | -0.4286 | Yes |
| 226 | NFKB2 | na | 18850 | -0.334 | -0.4309 | Yes |
| 227 | MLH1 | na | 19016 | -0.350 | -0.4342 | Yes |
| 228 | EGFR | na | 19043 | -0.354 | -0.4310 | Yes |
| 229 | LAMA5 | na | 19183 | -0.368 | -0.4329 | Yes |
| 230 | RXRA | na | 19209 | -0.370 | -0.4295 | Yes |
| 231 | IL6 | na | 19212 | -0.371 | -0.4250 | Yes |
| 232 | NFKB1 | na | 19213 | -0.371 | -0.4204 | Yes |
| 233 | CASP9 | na | 19243 | -0.373 | -0.4172 | Yes |
| 234 | STK4 | na | 19284 | -0.376 | -0.4144 | Yes |
| 235 | PIK3R3 | na | 19336 | -0.380 | -0.4120 | Yes |
| 236 | CRKL | na | 19431 | -0.390 | -0.4116 | Yes |
| 237 | STAT5A | na | 19487 | -0.396 | -0.4092 | Yes |
| 238 | TCF7L2 | na | 19504 | -0.397 | -0.4051 | Yes |
| 239 | PIK3R5 | na | 19509 | -0.397 | -0.4003 | Yes |
| 240 | WNT5A | na | 19541 | -0.401 | -0.3968 | Yes |
| 241 | FZD1 | na | 19601 | -0.406 | -0.3945 | Yes |
| 242 | SMAD3 | na | 19611 | -0.408 | -0.3899 | Yes |
| 243 | SMO | na | 19616 | -0.409 | -0.3851 | Yes |
| 244 | SOS2 | na | 19626 | -0.409 | -0.3804 | Yes |
| 245 | FGFR3 | na | 19694 | -0.416 | -0.3784 | Yes |
| 246 | ARNT | na | 19728 | -0.419 | -0.3747 | Yes |
| 247 | ETS1 | na | 19731 | -0.420 | -0.3696 | Yes |
| 248 | HIF1A | na | 19807 | -0.426 | -0.3678 | Yes |
| 249 | TGFB3 | na | 19860 | -0.432 | -0.3649 | Yes |
| 250 | TPR | na | 19907 | -0.436 | -0.3617 | Yes |
| 251 | CCDC6 | na | 19937 | -0.438 | -0.3576 | Yes |
| 252 | PDGFRA | na | 19955 | -0.440 | -0.3529 | Yes |
| 253 | CTNNA3 | na | 19975 | -0.442 | -0.3484 | Yes |
| 254 | JAK1 | na | 20014 | -0.446 | -0.3446 | Yes |
| 255 | SUFU | na | 20032 | -0.448 | -0.3399 | Yes |
| 256 | GSK3B | na | 20096 | -0.455 | -0.3372 | Yes |
| 257 | LAMA2 | na | 20114 | -0.457 | -0.3323 | Yes |
| 258 | TGFBR2 | na | 20121 | -0.457 | -0.3269 | Yes |
| 259 | LAMB2 | na | 20135 | -0.460 | -0.3219 | Yes |
| 260 | CBLB | na | 20162 | -0.463 | -0.3173 | Yes |
| 261 | NCOA4 | na | 20174 | -0.464 | -0.3121 | Yes |
| 262 | TRAF3 | na | 20180 | -0.465 | -0.3066 | Yes |
| 263 | FOXO1 | na | 20223 | -0.469 | -0.3027 | Yes |
| 264 | EGF | na | 20244 | -0.471 | -0.2979 | Yes |
| 265 | ITGA6 | na | 20245 | -0.471 | -0.2920 | Yes |
| 266 | XIAP | na | 20306 | -0.478 | -0.2889 | Yes |
| 267 | FZD7 | na | 20408 | -0.489 | -0.2875 | Yes |
| 268 | ERBB2 | na | 20414 | -0.490 | -0.2817 | Yes |
| 269 | FGF12 | na | 20471 | -0.499 | -0.2781 | Yes |
| 270 | MECOM | na | 20510 | -0.503 | -0.2737 | Yes |
| 271 | DAPK1 | na | 20514 | -0.503 | -0.2676 | Yes |
| 272 | PIK3CB | na | 20535 | -0.507 | -0.2623 | Yes |
| 273 | SOS1 | na | 20563 | -0.511 | -0.2572 | Yes |
| 274 | PML | na | 20576 | -0.512 | -0.2515 | Yes |
| 275 | CDK6 | na | 20612 | -0.518 | -0.2467 | Yes |
| 276 | STAT3 | na | 20708 | -0.529 | -0.2445 | Yes |
| 277 | ITGA3 | na | 20718 | -0.531 | -0.2384 | Yes |
| 278 | MET | na | 20741 | -0.536 | -0.2328 | Yes |
| 279 | ABL1 | na | 20795 | -0.545 | -0.2285 | Yes |
| 280 | BRAF | na | 20807 | -0.547 | -0.2223 | Yes |
| 281 | TGFBR1 | na | 20821 | -0.549 | -0.2161 | Yes |
| 282 | JUP | na | 20878 | -0.558 | -0.2118 | Yes |
| 283 | DVL3 | na | 20890 | -0.559 | -0.2054 | Yes |
| 284 | COL4A1 | na | 20907 | -0.561 | -0.1992 | Yes |
| 285 | PIAS3 | na | 20932 | -0.565 | -0.1933 | Yes |
| 286 | PDGFRB | na | 20968 | -0.572 | -0.1879 | Yes |
| 287 | FZD4 | na | 20970 | -0.572 | -0.1809 | Yes |
| 288 | STAT5B | na | 20976 | -0.573 | -0.1740 | Yes |
| 289 | BCR | na | 21121 | -0.601 | -0.1733 | Yes |
| 290 | RUNX1T1 | na | 21146 | -0.607 | -0.1669 | Yes |
| 291 | IGF1R | na | 21187 | -0.617 | -0.1611 | Yes |
| 292 | APC | na | 21200 | -0.619 | -0.1540 | Yes |
| 293 | CBL | na | 21212 | -0.623 | -0.1468 | Yes |
| 294 | GLI3 | na | 21222 | -0.625 | -0.1395 | Yes |
| 295 | ITGAV | na | 21226 | -0.625 | -0.1319 | Yes |
| 296 | COL4A6 | na | 21228 | -0.627 | -0.1242 | Yes |
| 297 | FZD5 | na | 21229 | -0.627 | -0.1165 | Yes |
| 298 | PLCG1 | na | 21230 | -0.627 | -0.1087 | Yes |
| 299 | LAMC1 | na | 21239 | -0.629 | -0.1013 | Yes |
| 300 | PTCH1 | na | 21269 | -0.636 | -0.0948 | Yes |
| 301 | COL4A2 | na | 21287 | -0.639 | -0.0877 | Yes |
| 302 | CREBBP | na | 21304 | -0.643 | -0.0805 | Yes |
| 303 | MTOR | na | 21338 | -0.653 | -0.0740 | Yes |
| 304 | BRCA2 | na | 21373 | -0.661 | -0.0674 | Yes |
| 305 | FN1 | na | 21407 | -0.672 | -0.0606 | Yes |
| 306 | EP300 | na | 21416 | -0.674 | -0.0527 | Yes |
| 307 | PIK3R1 | na | 21419 | -0.674 | -0.0444 | Yes |
| 308 | APC2 | na | 21564 | -0.733 | -0.0420 | Yes |
| 309 | PPARG | na | 21669 | -0.808 | -0.0369 | Yes |
| 310 | COL4A4 | na | 21703 | -0.851 | -0.0279 | Yes |
| 311 | FGF1 | na | 21729 | -0.871 | -0.0183 | Yes |
| 312 | GLI1 | na | 21743 | -0.894 | -0.0078 | Yes |
| 313 | FZD6 | na | 21825 | -1.125 | 0.0023 | Yes |
Table: GSEA details [plain text format]

  

Fig 2: KEGG\_PATHWAYS\_IN\_CANCER      
 Blue-Pink O' Gram in the Space of the Analyzed GeneSet

  

Fig 3: KEGG\_PATHWAYS\_IN\_CANCER: Random ES distribution      
 Gene set null distribution of ES for **KEGG\_PATHWAYS\_IN\_CANCER**
