# Supplementary material for: Regulation of microglia related neuroinflammation contributes to the protective effect of Gelsevirine on ischemic stroke
Source: Front Immunol. 2023 Mar 30;14:1164278. doi: 10.3389/fimmu.2023.1164278 (PMC10098192; doi:10.3389/fimmu.2023.1164278)
Supplement: Supplementary file 6 [file DataSheet_6.zip › fig 5 raw/fig 5-G raw/KEGG.Gsea.1649954846225/KEGG_PHOSPHATIDYLINOSITOL_SIGNALING_SYSTEM.html]

Details for gene set KEGG\_PHOSPHATIDYLINOSITOL\_SIGNALING\_SYSTEM[GSEA]

|  || Dataset | OGD\_DRUG\_DRUG.OGD\_FRUG.cls#Gs\_versus\_MCAO.OGD\_FRUG.cls#Gs\_versus\_MCAO\_repos |
| Phenotype | OGD\_FRUG.cls#Gs\_versus\_MCAO\_repos |
| Upregulated in class | MCAO |
| GeneSet | KEGG\_PHOSPHATIDYLINOSITOL\_SIGNALING\_SYSTEM |
| Enrichment Score (ES) | -0.65422183 |
| Normalized Enrichment Score (NES) | -1.8525147 |
| Nominal p-value | 0.0 |
| FDR q-value | 0.0026147896 |
| FWER p-Value | 0.003 |
Table: GSEA Results Summary

  

Fig 1: Enrichment plot: KEGG\_PHOSPHATIDYLINOSITOL\_SIGNALING\_SYSTEM      
 Profile of the Running ES Score & Positions of GeneSet Members on the Rank Ordered List

  

| SYMBOL | TITLE | RANK IN GENE LIST | RANK METRIC SCORE | RUNNING ES | CORE ENRICHMENT || 1 | PIK3CG | na | 1248 | 0.428 | -0.0374 | No |
| 2 | CALM1 | na | 3305 | 0.214 | -0.1218 | No |
| 3 | CALM2 | na | 3550 | 0.196 | -0.1240 | No |
| 4 | PLCD4 | na | 4218 | 0.148 | -0.1477 | No |
| 5 | PRKCG | na | 4221 | 0.148 | -0.1410 | No |
| 6 | PLCG2 | na | 5215 | 0.084 | -0.1826 | No |
| 7 | INPP5J | na | 5339 | 0.077 | -0.1847 | No |
| 8 | DGKA | na | 5760 | 0.054 | -0.2015 | No |
| 9 | CDIPT | na | 6176 | 0.033 | -0.2190 | No |
| 10 | CDS1 | na | 6455 | 0.023 | -0.2307 | No |
| 11 | PLCZ1 | na | 6484 | 0.022 | -0.2310 | No |
| 12 | PLCD1 | na | 6813 | 0.009 | -0.2456 | No |
| 13 | CALML3 | na | 8189 | 0.000 | -0.3087 | No |
| 14 | PIP5K1B | na | 10813 | 0.000 | -0.4290 | No |
| 15 | PIK3C2G | na | 11106 | 0.000 | -0.4424 | No |
| 16 | DGKB | na | 13097 | 0.000 | -0.5336 | No |
| 17 | ITPK1 | na | 13267 | -0.001 | -0.5414 | No |
| 18 | PRKCB | na | 13570 | -0.009 | -0.5548 | No |
| 19 | DGKI | na | 13669 | -0.011 | -0.5588 | No |
| 20 | PLCB2 | na | 13694 | -0.012 | -0.5593 | No |
| 21 | DGKG | na | 13820 | -0.015 | -0.5643 | No |
| 22 | PLCE1 | na | 13860 | -0.016 | -0.5654 | No |
| 23 | IMPA1 | na | 14460 | -0.039 | -0.5911 | No |
| 24 | CALM3 | na | 14729 | -0.052 | -0.6009 | No |
| 25 | INPP1 | na | 14946 | -0.065 | -0.6078 | No |
| 26 | PLCD3 | na | 15310 | -0.087 | -0.6205 | No |
| 27 | INPP5K | na | 15497 | -0.097 | -0.6245 | No |
| 28 | PIP4K2C | na | 16002 | -0.129 | -0.6417 | No |
| 29 | PIP4K2A | na | 16075 | -0.133 | -0.6388 | No |
| 30 | IPPK | na | 16242 | -0.143 | -0.6398 | No |
| 31 | PIK3C3 | na | 16557 | -0.166 | -0.6466 | Yes |
| 32 | IMPA2 | na | 16587 | -0.168 | -0.6401 | Yes |
| 33 | PLCB4 | na | 16825 | -0.184 | -0.6425 | Yes |
| 34 | PIP5K1A | na | 17050 | -0.199 | -0.6436 | Yes |
| 35 | INPP5A | na | 17072 | -0.201 | -0.6353 | Yes |
| 36 | CDS2 | na | 17253 | -0.213 | -0.6337 | Yes |
| 37 | PTEN | na | 17342 | -0.220 | -0.6276 | Yes |
| 38 | DGKQ | na | 17481 | -0.229 | -0.6233 | Yes |
| 39 | PI4KB | na | 17757 | -0.249 | -0.6244 | Yes |
| 40 | PIK3CD | na | 18197 | -0.284 | -0.6314 | Yes |
| 41 | PRKCA | na | 18221 | -0.286 | -0.6193 | Yes |
| 42 | DGKZ | na | 18232 | -0.287 | -0.6064 | Yes |
| 43 | DGKD | na | 18419 | -0.298 | -0.6012 | Yes |
| 44 | PIK3R2 | na | 18436 | -0.299 | -0.5881 | Yes |
| 45 | PIK3CA | na | 18513 | -0.305 | -0.5775 | Yes |
| 46 | INPP4A | na | 18570 | -0.309 | -0.5658 | Yes |
| 47 | PLCB3 | na | 18848 | -0.334 | -0.5630 | Yes |
| 48 | INPP5B | na | 18889 | -0.339 | -0.5492 | Yes |
| 49 | OCRL | na | 19163 | -0.366 | -0.5448 | Yes |
| 50 | SYNJ2 | na | 19191 | -0.369 | -0.5290 | Yes |
| 51 | PIK3R3 | na | 19336 | -0.380 | -0.5180 | Yes |
| 52 | PIK3R5 | na | 19509 | -0.397 | -0.5075 | Yes |
| 53 | ITPR2 | na | 19809 | -0.427 | -0.5015 | Yes |
| 54 | PIP4K2B | na | 19990 | -0.443 | -0.4892 | Yes |
| 55 | DGKE | na | 20183 | -0.465 | -0.4765 | Yes |
| 56 | INPPL1 | na | 20322 | -0.480 | -0.4607 | Yes |
| 57 | ITPR3 | na | 20416 | -0.491 | -0.4422 | Yes |
| 58 | PIK3CB | na | 20535 | -0.507 | -0.4242 | Yes |
| 59 | PIKFYVE | na | 20680 | -0.527 | -0.4065 | Yes |
| 60 | PIK3C2A | na | 20684 | -0.527 | -0.3822 | Yes |
| 61 | PI4KA | na | 20686 | -0.527 | -0.3579 | Yes |
| 62 | SYNJ1 | na | 20828 | -0.550 | -0.3389 | Yes |
| 63 | ITPR1 | na | 20993 | -0.577 | -0.3198 | Yes |
| 64 | INPP5E | na | 21077 | -0.595 | -0.2961 | Yes |
| 65 | DGKH | na | 21106 | -0.599 | -0.2696 | Yes |
| 66 | PIP5K1C | na | 21145 | -0.607 | -0.2433 | Yes |
| 67 | ITPKB | na | 21198 | -0.619 | -0.2171 | Yes |
| 68 | PLCG1 | na | 21230 | -0.627 | -0.1895 | Yes |
| 69 | PIK3R1 | na | 21419 | -0.674 | -0.1670 | Yes |
| 70 | PIK3C2B | na | 21420 | -0.675 | -0.1358 | Yes |
| 71 | INPP4B | na | 21534 | -0.719 | -0.1077 | Yes |
| 72 | PLCB1 | na | 21572 | -0.736 | -0.0754 | Yes |
| 73 | INPP5D | na | 21674 | -0.816 | -0.0423 | Yes |
| 74 | ITPKA | na | 21817 | -1.113 | 0.0027 | Yes |
Table: GSEA details [plain text format]

  

Fig 2: KEGG\_PHOSPHATIDYLINOSITOL\_SIGNALING\_SYSTEM      
 Blue-Pink O' Gram in the Space of the Analyzed GeneSet

  

Fig 3: KEGG\_PHOSPHATIDYLINOSITOL\_SIGNALING\_SYSTEM: Random ES distribution      
 Gene set null distribution of ES for **KEGG\_PHOSPHATIDYLINOSITOL\_SIGNALING\_SYSTEM**
