# Supplementary material for: Regulation of microglia related neuroinflammation contributes to the protective effect of Gelsevirine on ischemic stroke
Source: Front Immunol. 2023 Mar 30;14:1164278. doi: 10.3389/fimmu.2023.1164278 (PMC10098192; doi:10.3389/fimmu.2023.1164278)
Supplement: Supplementary file 6 [file DataSheet_6.zip › fig 5 raw/fig 5-G raw/KEGG.Gsea.1649954846225/KEGG_PROSTATE_CANCER.html]

Details for gene set KEGG\_PROSTATE\_CANCER[GSEA]

|  || Dataset | OGD\_DRUG\_DRUG.OGD\_FRUG.cls#Gs\_versus\_MCAO.OGD\_FRUG.cls#Gs\_versus\_MCAO\_repos |
| Phenotype | OGD\_FRUG.cls#Gs\_versus\_MCAO\_repos |
| Upregulated in class | MCAO |
| GeneSet | KEGG\_PROSTATE\_CANCER |
| Enrichment Score (ES) | -0.5380183 |
| Normalized Enrichment Score (NES) | -1.5885377 |
| Nominal p-value | 0.0 |
| FDR q-value | 0.039862562 |
| FWER p-Value | 0.427 |
Table: GSEA Results Summary

  

Fig 1: Enrichment plot: KEGG\_PROSTATE\_CANCER      
 Profile of the Running ES Score & Positions of GeneSet Members on the Rank Ordered List

  

| SYMBOL | TITLE | RANK IN GENE LIST | RANK METRIC SCORE | RUNNING ES | CORE ENRICHMENT || 1 | HSP90B1 | na | 1143 | 0.443 | -0.0322 | No |
| 2 | PIK3CG | na | 1248 | 0.428 | -0.0175 | No |
| 3 | CDKN1A | na | 1773 | 0.366 | -0.0248 | No |
| 4 | GSTP1 | na | 1927 | 0.346 | -0.0161 | No |
| 5 | PDGFA | na | 2504 | 0.286 | -0.0295 | No |
| 6 | HRAS | na | 2716 | 0.268 | -0.0269 | No |
| 7 | ATF4 | na | 3226 | 0.220 | -0.0402 | No |
| 8 | TGFA | na | 3523 | 0.198 | -0.0448 | No |
| 9 | BAD | na | 3658 | 0.189 | -0.0423 | No |
| 10 | NFKBIA | na | 3820 | 0.179 | -0.0415 | No |
| 11 | MDM2 | na | 4059 | 0.159 | -0.0452 | No |
| 12 | CREB3L4 | na | 4274 | 0.145 | -0.0484 | No |
| 13 | SRD5A2 | na | 4377 | 0.138 | -0.0468 | No |
| 14 | NRAS | na | 4669 | 0.117 | -0.0548 | No |
| 15 | MAP2K2 | na | 4692 | 0.115 | -0.0506 | No |
| 16 | TCF7 | na | 4825 | 0.106 | -0.0518 | No |
| 17 | MAPK3 | na | 4918 | 0.100 | -0.0515 | No |
| 18 | HSP90AB1 | na | 5242 | 0.082 | -0.0625 | No |
| 19 | HSP90AA1 | na | 5244 | 0.082 | -0.0588 | No |
| 20 | GRB2 | na | 5726 | 0.057 | -0.0783 | No |
| 21 | CREB3 | na | 5728 | 0.056 | -0.0758 | No |
| 22 | CDKN1B | na | 5942 | 0.045 | -0.0835 | No |
| 23 | CDK2 | na | 6514 | 0.020 | -0.1088 | No |
| 24 | KRAS | na | 6543 | 0.019 | -0.1092 | No |
| 25 | NKX3-1 | na | 8635 | 0.000 | -0.2052 | No |
| 26 | INSRR | na | 12228 | 0.000 | -0.3700 | No |
| 27 | CCNE2 | na | 13270 | -0.001 | -0.4178 | No |
| 28 | CREB3L3 | na | 13285 | -0.001 | -0.4183 | No |
| 29 | FGFR2 | na | 13468 | -0.007 | -0.4264 | No |
| 30 | PDGFD | na | 13561 | -0.009 | -0.4302 | No |
| 31 | AR | na | 13568 | -0.009 | -0.4301 | No |
| 32 | IKBKG | na | 14695 | -0.050 | -0.4795 | No |
| 33 | MAP2K1 | na | 15179 | -0.079 | -0.4980 | No |
| 34 | PDGFC | na | 15436 | -0.094 | -0.5055 | No |
| 35 | CREB3L1 | na | 15551 | -0.101 | -0.5061 | No |
| 36 | RELA | na | 15584 | -0.103 | -0.5029 | No |
| 37 | E2F1 | na | 15769 | -0.113 | -0.5061 | No |
| 38 | ARAF | na | 15889 | -0.121 | -0.5061 | No |
| 39 | MAPK1 | na | 16234 | -0.143 | -0.5153 | No |
| 40 | CCNE1 | na | 16729 | -0.178 | -0.5299 | Yes |
| 41 | E2F3 | na | 16736 | -0.179 | -0.5220 | Yes |
| 42 | LEF1 | na | 16773 | -0.181 | -0.5154 | Yes |
| 43 | AKT2 | na | 16784 | -0.181 | -0.5076 | Yes |
| 44 | IKBKB | na | 16790 | -0.182 | -0.4995 | Yes |
| 45 | RB1 | na | 16796 | -0.182 | -0.4915 | Yes |
| 46 | AKT1 | na | 16827 | -0.184 | -0.4844 | Yes |
| 47 | TCF7L1 | na | 16939 | -0.192 | -0.4808 | Yes |
| 48 | RAF1 | na | 17251 | -0.212 | -0.4853 | Yes |
| 49 | PTEN | na | 17342 | -0.220 | -0.4794 | Yes |
| 50 | CCND1 | na | 17421 | -0.225 | -0.4727 | Yes |
| 51 | BCL2 | na | 17522 | -0.232 | -0.4668 | Yes |
| 52 | PDGFB | na | 17563 | -0.234 | -0.4579 | Yes |
| 53 | CHUK | na | 17623 | -0.238 | -0.4497 | Yes |
| 54 | IGF1 | na | 17740 | -0.247 | -0.4438 | Yes |
| 55 | CREB3L2 | na | 17814 | -0.254 | -0.4355 | Yes |
| 56 | E2F2 | na | 17853 | -0.258 | -0.4255 | Yes |
| 57 | AKT3 | na | 17899 | -0.261 | -0.4156 | Yes |
| 58 | FGFR1 | na | 18053 | -0.273 | -0.4102 | Yes |
| 59 | PIK3CD | na | 18197 | -0.284 | -0.4038 | Yes |
| 60 | CREB1 | na | 18226 | -0.286 | -0.3920 | Yes |
| 61 | CTNNB1 | na | 18285 | -0.290 | -0.3814 | Yes |
| 62 | PIK3R2 | na | 18436 | -0.299 | -0.3746 | Yes |
| 63 | PIK3CA | na | 18513 | -0.305 | -0.3642 | Yes |
| 64 | EGFR | na | 19043 | -0.354 | -0.3724 | Yes |
| 65 | NFKB1 | na | 19213 | -0.371 | -0.3632 | Yes |
| 66 | CASP9 | na | 19243 | -0.373 | -0.3475 | Yes |
| 67 | PIK3R3 | na | 19336 | -0.380 | -0.3344 | Yes |
| 68 | TCF7L2 | na | 19504 | -0.397 | -0.3239 | Yes |
| 69 | PIK3R5 | na | 19509 | -0.397 | -0.3060 | Yes |
| 70 | SOS2 | na | 19626 | -0.409 | -0.2926 | Yes |
| 71 | PDGFRA | na | 19955 | -0.440 | -0.2876 | Yes |
| 72 | GSK3B | na | 20096 | -0.455 | -0.2732 | Yes |
| 73 | PDPK1 | na | 20100 | -0.455 | -0.2526 | Yes |
| 74 | FOXO1 | na | 20223 | -0.469 | -0.2368 | Yes |
| 75 | EGF | na | 20244 | -0.471 | -0.2162 | Yes |
| 76 | CREB5 | na | 20307 | -0.478 | -0.1972 | Yes |
| 77 | ERBB2 | na | 20414 | -0.490 | -0.1797 | Yes |
| 78 | PIK3CB | na | 20535 | -0.507 | -0.1621 | Yes |
| 79 | SOS1 | na | 20563 | -0.511 | -0.1400 | Yes |
| 80 | BRAF | na | 20807 | -0.547 | -0.1262 | Yes |
| 81 | PDGFRB | na | 20968 | -0.572 | -0.1075 | Yes |
| 82 | IGF1R | na | 21187 | -0.617 | -0.0893 | Yes |
| 83 | CREBBP | na | 21304 | -0.643 | -0.0653 | Yes |
| 84 | MTOR | na | 21338 | -0.653 | -0.0370 | Yes |
| 85 | EP300 | na | 21416 | -0.674 | -0.0098 | Yes |
| 86 | PIK3R1 | na | 21419 | -0.674 | 0.0209 | Yes |
Table: GSEA details [plain text format]

  

Fig 2: KEGG\_PROSTATE\_CANCER      
 Blue-Pink O' Gram in the Space of the Analyzed GeneSet

  

Fig 3: KEGG\_PROSTATE\_CANCER: Random ES distribution      
 Gene set null distribution of ES for **KEGG\_PROSTATE\_CANCER**
