# Supplementary material for: Regulation of microglia related neuroinflammation contributes to the protective effect of Gelsevirine on ischemic stroke
Source: Front Immunol. 2023 Mar 30;14:1164278. doi: 10.3389/fimmu.2023.1164278 (PMC10098192; doi:10.3389/fimmu.2023.1164278)
Supplement: Supplementary file 6 [file DataSheet_6.zip › fig 5 raw/fig 5-G raw/KEGG.Gsea.1649954846225/KEGG_PROTEASOME.html]

Details for gene set KEGG\_PROTEASOME[GSEA]

|  || Dataset | OGD\_DRUG\_DRUG.OGD\_FRUG.cls#Gs\_versus\_MCAO.OGD\_FRUG.cls#Gs\_versus\_MCAO\_repos |
| Phenotype | OGD\_FRUG.cls#Gs\_versus\_MCAO\_repos |
| Upregulated in class | Gs |
| GeneSet | KEGG\_PROTEASOME |
| Enrichment Score (ES) | 0.7773998 |
| Normalized Enrichment Score (NES) | 2.1851263 |
| Nominal p-value | 0.0 |
| FDR q-value | 0.0 |
| FWER p-Value | 0.0 |
Table: GSEA Results Summary

  

Fig 1: Enrichment plot: KEGG\_PROTEASOME      
 Profile of the Running ES Score & Positions of GeneSet Members on the Rank Ordered List

  

| SYMBOL | TITLE | RANK IN GENE LIST | RANK METRIC SCORE | RUNNING ES | CORE ENRICHMENT || 1 | PSMB8 | na | 30 | 1.397 | 0.0982 | Yes |
| 2 | PSMB7 | na | 435 | 0.620 | 0.1239 | Yes |
| 3 | PSMB4 | na | 539 | 0.572 | 0.1599 | Yes |
| 4 | PSMA6 | na | 735 | 0.516 | 0.1878 | Yes |
| 5 | PSMA5 | na | 768 | 0.507 | 0.2224 | Yes |
| 6 | PSMB3 | na | 861 | 0.484 | 0.2527 | Yes |
| 7 | POMP | na | 1050 | 0.457 | 0.2766 | Yes |
| 8 | PSMB1 | na | 1208 | 0.433 | 0.3003 | Yes |
| 9 | PSMA3 | na | 1229 | 0.431 | 0.3301 | Yes |
| 10 | PSMB6 | na | 1252 | 0.428 | 0.3596 | Yes |
| 11 | PSMA7 | na | 1331 | 0.417 | 0.3857 | Yes |
| 12 | PSMA4 | na | 1348 | 0.415 | 0.4146 | Yes |
| 13 | PSMA2 | na | 1522 | 0.398 | 0.4350 | Yes |
| 14 | PSMD4 | na | 1600 | 0.387 | 0.4591 | Yes |
| 15 | PSMC6 | na | 1625 | 0.383 | 0.4853 | Yes |
| 16 | PSMD6 | na | 1653 | 0.379 | 0.5111 | Yes |
| 17 | PSMB2 | na | 1712 | 0.373 | 0.5350 | Yes |
| 18 | PSMD14 | na | 1739 | 0.369 | 0.5601 | Yes |
| 19 | PSMA1 | na | 1854 | 0.356 | 0.5802 | Yes |
| 20 | PSME2 | na | 1953 | 0.343 | 0.6002 | Yes |
| 21 | PSME1 | na | 2090 | 0.329 | 0.6174 | Yes |
| 22 | PSMC2 | na | 2133 | 0.323 | 0.6385 | Yes |
| 23 | PSMD7 | na | 2378 | 0.297 | 0.6485 | Yes |
| 24 | PSMC5 | na | 2572 | 0.281 | 0.6596 | Yes |
| 25 | PSMB5 | na | 2587 | 0.279 | 0.6789 | Yes |
| 26 | PSMC4 | na | 2617 | 0.277 | 0.6973 | Yes |
| 27 | PSMD13 | na | 2804 | 0.259 | 0.7073 | Yes |
| 28 | PSMD12 | na | 2816 | 0.258 | 0.7252 | Yes |
| 29 | PSMC3 | na | 2821 | 0.257 | 0.7434 | Yes |
| 30 | PSMB10 | na | 2826 | 0.257 | 0.7615 | Yes |
| 31 | PSMD8 | na | 2992 | 0.242 | 0.7712 | Yes |
| 32 | PSMB9 | na | 3291 | 0.215 | 0.7729 | Yes |
| 33 | PSMC1 | na | 3503 | 0.199 | 0.7774 | Yes |
| 34 | PSMD11 | na | 4094 | 0.156 | 0.7615 | No |
| 35 | PSMD1 | na | 5203 | 0.085 | 0.7168 | No |
| 36 | PSMA8 | na | 9496 | 0.000 | 0.5202 | No |
| 37 | IFNG | na | 9828 | 0.000 | 0.5051 | No |
| 38 | PSMB11 | na | 10118 | 0.000 | 0.4918 | No |
| 39 | PSMD3 | na | 13262 | -0.000 | 0.3479 | No |
| 40 | PSMD2 | na | 13368 | -0.003 | 0.3433 | No |
| 41 | PSMF1 | na | 15617 | -0.104 | 0.2478 | No |
| 42 | PSME3 | na | 16442 | -0.157 | 0.2213 | No |
| 43 | PSME4 | na | 19389 | -0.386 | 0.1139 | No |
Table: GSEA details [plain text format]

  

Fig 2: KEGG\_PROTEASOME      
 Blue-Pink O' Gram in the Space of the Analyzed GeneSet

  

Fig 3: KEGG\_PROTEASOME: Random ES distribution      
 Gene set null distribution of ES for **KEGG\_PROTEASOME**
