# Supplementary material for: Regulation of microglia related neuroinflammation contributes to the protective effect of Gelsevirine on ischemic stroke
Source: Front Immunol. 2023 Mar 30;14:1164278. doi: 10.3389/fimmu.2023.1164278 (PMC10098192; doi:10.3389/fimmu.2023.1164278)
Supplement: Supplementary file 6 [file DataSheet_6.zip › fig 5 raw/fig 5-G raw/KEGG.Gsea.1649954846225/KEGG_PROTEIN_EXPORT.html]

Details for gene set KEGG\_PROTEIN\_EXPORT[GSEA]

|  || Dataset | OGD\_DRUG\_DRUG.OGD\_FRUG.cls#Gs\_versus\_MCAO.OGD\_FRUG.cls#Gs\_versus\_MCAO\_repos |
| Phenotype | OGD\_FRUG.cls#Gs\_versus\_MCAO\_repos |
| Upregulated in class | Gs |
| GeneSet | KEGG\_PROTEIN\_EXPORT |
| Enrichment Score (ES) | 0.80922854 |
| Normalized Enrichment Score (NES) | 1.994941 |
| Nominal p-value | 0.0024630541 |
| FDR q-value | 2.0000001E-4 |
| FWER p-Value | 0.001 |
Table: GSEA Results Summary

  

Fig 1: Enrichment plot: KEGG\_PROTEIN\_EXPORT      
 Profile of the Running ES Score & Positions of GeneSet Members on the Rank Ordered List

  

| SYMBOL | TITLE | RANK IN GENE LIST | RANK METRIC SCORE | RUNNING ES | CORE ENRICHMENT || 1 | HSPA5 | na | 265 | 0.727 | 0.0909 | Yes |
| 2 | SEC61G | na | 283 | 0.712 | 0.1910 | Yes |
| 3 | SRP19 | na | 396 | 0.632 | 0.2754 | Yes |
| 4 | SPCS1 | na | 546 | 0.571 | 0.3495 | Yes |
| 5 | SRP9 | na | 609 | 0.553 | 0.4249 | Yes |
| 6 | SEC61B | na | 689 | 0.529 | 0.4963 | Yes |
| 7 | SEC62 | na | 765 | 0.508 | 0.5647 | Yes |
| 8 | IMMP1L | na | 1003 | 0.464 | 0.6196 | Yes |
| 9 | SRP14 | na | 1454 | 0.405 | 0.6564 | Yes |
| 10 | SEC11C | na | 1475 | 0.402 | 0.7124 | Yes |
| 11 | SEC11A | na | 1523 | 0.397 | 0.7666 | Yes |
| 12 | IMMP2L | na | 1736 | 0.370 | 0.8092 | Yes |
| 13 | SPCS2 | na | 2948 | 0.246 | 0.7887 | No |
| 14 | SEC63 | na | 5122 | 0.089 | 0.7018 | No |
| 15 | OXA1L | na | 5575 | 0.064 | 0.6902 | No |
| 16 | SPCS3 | na | 6079 | 0.037 | 0.6725 | No |
| 17 | SRPRB | na | 6211 | 0.032 | 0.6710 | No |
| 18 | SRP68 | na | 6653 | 0.015 | 0.6530 | No |
| 19 | SRP72 | na | 15027 | -0.070 | 0.2797 | No |
| 20 | SEC61A1 | na | 15046 | -0.071 | 0.2889 | No |
| 21 | SEC61A2 | na | 16567 | -0.166 | 0.2429 | No |
Table: GSEA details [plain text format]

  

Fig 2: KEGG\_PROTEIN\_EXPORT      
 Blue-Pink O' Gram in the Space of the Analyzed GeneSet

  

Fig 3: KEGG\_PROTEIN\_EXPORT: Random ES distribution      
 Gene set null distribution of ES for **KEGG\_PROTEIN\_EXPORT**
