# Supplementary material for: Regulation of microglia related neuroinflammation contributes to the protective effect of Gelsevirine on ischemic stroke
Source: Front Immunol. 2023 Mar 30;14:1164278. doi: 10.3389/fimmu.2023.1164278 (PMC10098192; doi:10.3389/fimmu.2023.1164278)
Supplement: Supplementary file 6 [file DataSheet_6.zip › fig 5 raw/fig 5-G raw/KEGG.Gsea.1649954846225/KEGG_PROXIMAL_TUBULE_BICARBONATE_RECLAMATION.html]

Details for gene set KEGG\_PROXIMAL\_TUBULE\_BICARBONATE\_RECLAMATION[GSEA]

|  || Dataset | OGD\_DRUG\_DRUG.OGD\_FRUG.cls#Gs\_versus\_MCAO.OGD\_FRUG.cls#Gs\_versus\_MCAO\_repos |
| Phenotype | OGD\_FRUG.cls#Gs\_versus\_MCAO\_repos |
| Upregulated in class | Gs |
| GeneSet | KEGG\_PROXIMAL\_TUBULE\_BICARBONATE\_RECLAMATION |
| Enrichment Score (ES) | 0.55218273 |
| Normalized Enrichment Score (NES) | 1.3465827 |
| Nominal p-value | 0.10054348 |
| FDR q-value | 0.23566537 |
| FWER p-Value | 0.974 |
Table: GSEA Results Summary

  

Fig 1: Enrichment plot: KEGG\_PROXIMAL\_TUBULE\_BICARBONATE\_RECLAMATION      
 Profile of the Running ES Score & Positions of GeneSet Members on the Rank Ordered List

  

| SYMBOL | TITLE | RANK IN GENE LIST | RANK METRIC SCORE | RUNNING ES | CORE ENRICHMENT || 1 | FXYD2 | na | 36 | 1.339 | 0.2234 | Yes |
| 2 | ATP1B1 | na | 108 | 0.965 | 0.3824 | Yes |
| 3 | MDH1 | na | 1279 | 0.424 | 0.4001 | Yes |
| 4 | PCK1 | na | 1622 | 0.384 | 0.4489 | Yes |
| 5 | ATP1B3 | na | 2181 | 0.318 | 0.4769 | Yes |
| 6 | PCK2 | na | 2203 | 0.315 | 0.5288 | Yes |
| 7 | SLC9A3 | na | 2688 | 0.271 | 0.5522 | Yes |
| 8 | ATP1A3 | na | 4321 | 0.142 | 0.5014 | No |
| 9 | ATP1A2 | na | 6662 | 0.015 | 0.3967 | No |
| 10 | ATP1B4 | na | 8776 | 0.000 | 0.3001 | No |
| 11 | ATP1B2 | na | 8777 | 0.000 | 0.3001 | No |
| 12 | ATP1A4 | na | 8804 | 0.000 | 0.2989 | No |
| 13 | GLUD1 | na | 15083 | -0.073 | 0.0239 | No |
| 14 | GLS2 | na | 15298 | -0.086 | 0.0285 | No |
| 15 | SLC25A10 | na | 15908 | -0.122 | 0.0212 | No |
| 16 | AQP1 | na | 16983 | -0.195 | 0.0048 | No |
| 17 | SLC38A3 | na | 17177 | -0.207 | 0.0309 | No |
| 18 | ATP1A1 | na | 17371 | -0.222 | 0.0593 | No |
| 19 | GLS | na | 18456 | -0.300 | 0.0601 | No |
| 20 | SLC4A4 | na | 20977 | -0.573 | 0.0411 | No |
Table: GSEA details [plain text format]

  

Fig 2: KEGG\_PROXIMAL\_TUBULE\_BICARBONATE\_RECLAMATION      
 Blue-Pink O' Gram in the Space of the Analyzed GeneSet

  

Fig 3: KEGG\_PROXIMAL\_TUBULE\_BICARBONATE\_RECLAMATION: Random ES distribution      
 Gene set null distribution of ES for **KEGG\_PROXIMAL\_TUBULE\_BICARBONATE\_RECLAMATION**
