# Supplementary material for: Regulation of microglia related neuroinflammation contributes to the protective effect of Gelsevirine on ischemic stroke
Source: Front Immunol. 2023 Mar 30;14:1164278. doi: 10.3389/fimmu.2023.1164278 (PMC10098192; doi:10.3389/fimmu.2023.1164278)
Supplement: Supplementary file 6 [file DataSheet_6.zip › fig 5 raw/fig 5-G raw/KEGG.Gsea.1649954846225/KEGG_PURINE_METABOLISM.html]

Details for gene set KEGG\_PURINE\_METABOLISM[GSEA]

|  || Dataset | OGD\_DRUG\_DRUG.OGD\_FRUG.cls#Gs\_versus\_MCAO.OGD\_FRUG.cls#Gs\_versus\_MCAO\_repos |
| Phenotype | OGD\_FRUG.cls#Gs\_versus\_MCAO\_repos |
| Upregulated in class | Gs |
| GeneSet | KEGG\_PURINE\_METABOLISM |
| Enrichment Score (ES) | 0.36229354 |
| Normalized Enrichment Score (NES) | 1.2588843 |
| Nominal p-value | 0.028455285 |
| FDR q-value | 0.33733678 |
| FWER p-Value | 0.998 |
Table: GSEA Results Summary

  

Fig 1: Enrichment plot: KEGG\_PURINE\_METABOLISM      
 Profile of the Running ES Score & Positions of GeneSet Members on the Rank Ordered List

  

| SYMBOL | TITLE | RANK IN GENE LIST | RANK METRIC SCORE | RUNNING ES | CORE ENRICHMENT || 1 | PDE2A | na | 148 | 0.855 | 0.0182 | Yes |
| 2 | PDE6H | na | 263 | 0.729 | 0.0342 | Yes |
| 3 | GUK1 | na | 336 | 0.669 | 0.0504 | Yes |
| 4 | ADCY3 | na | 397 | 0.632 | 0.0661 | Yes |
| 5 | NME2 | na | 664 | 0.535 | 0.0695 | Yes |
| 6 | POLR2G | na | 774 | 0.505 | 0.0792 | Yes |
| 7 | NME1 | na | 818 | 0.494 | 0.0917 | Yes |
| 8 | NME3 | na | 1070 | 0.454 | 0.0934 | Yes |
| 9 | POLR2H | na | 1207 | 0.434 | 0.0998 | Yes |
| 10 | PDE1A | na | 1246 | 0.428 | 0.1106 | Yes |
| 11 | NUDT2 | na | 1316 | 0.418 | 0.1196 | Yes |
| 12 | POLR2I | na | 1419 | 0.409 | 0.1268 | Yes |
| 13 | NME4 | na | 1470 | 0.403 | 0.1363 | Yes |
| 14 | NT5C | na | 1491 | 0.401 | 0.1471 | Yes |
| 15 | ADPRM | na | 1492 | 0.401 | 0.1588 | Yes |
| 16 | POLR1D | na | 1542 | 0.394 | 0.1681 | Yes |
| 17 | ENTPD2 | na | 1580 | 0.390 | 0.1778 | Yes |
| 18 | NME7 | na | 1581 | 0.390 | 0.1892 | Yes |
| 19 | POLR2L | na | 1608 | 0.386 | 0.1992 | Yes |
| 20 | ITPA | na | 1654 | 0.379 | 0.2082 | Yes |
| 21 | APRT | na | 1657 | 0.379 | 0.2192 | Yes |
| 22 | POLE4 | na | 1748 | 0.368 | 0.2258 | Yes |
| 23 | POLR2J | na | 1758 | 0.367 | 0.2361 | Yes |
| 24 | POLR2D | na | 1820 | 0.360 | 0.2438 | Yes |
| 25 | POLR2F | na | 1837 | 0.358 | 0.2535 | Yes |
| 26 | NUDT9 | na | 1921 | 0.346 | 0.2598 | Yes |
| 27 | POLR2C | na | 1926 | 0.346 | 0.2697 | Yes |
| 28 | NME6 | na | 2131 | 0.323 | 0.2698 | Yes |
| 29 | PNP | na | 2244 | 0.310 | 0.2737 | Yes |
| 30 | ADK | na | 2245 | 0.310 | 0.2827 | Yes |
| 31 | ADCY5 | na | 2263 | 0.308 | 0.2910 | Yes |
| 32 | AK7 | na | 2477 | 0.289 | 0.2896 | Yes |
| 33 | AK2 | na | 2519 | 0.284 | 0.2960 | Yes |
| 34 | POLR1C | na | 2531 | 0.284 | 0.3038 | Yes |
| 35 | GMPR2 | na | 2594 | 0.279 | 0.3091 | Yes |
| 36 | POLR1E | na | 2864 | 0.254 | 0.3041 | Yes |
| 37 | POLD4 | na | 2954 | 0.245 | 0.3072 | Yes |
| 38 | IMPDH2 | na | 2955 | 0.245 | 0.3143 | Yes |
| 39 | POLR3D | na | 3129 | 0.230 | 0.3131 | Yes |
| 40 | DGUOK | na | 3238 | 0.219 | 0.3145 | Yes |
| 41 | PAPSS2 | na | 3239 | 0.219 | 0.3209 | Yes |
| 42 | POLR2K | na | 3264 | 0.217 | 0.3261 | Yes |
| 43 | POLR2E | na | 3386 | 0.208 | 0.3266 | Yes |
| 44 | POLE3 | na | 3398 | 0.207 | 0.3322 | Yes |
| 45 | PRIM1 | na | 3481 | 0.200 | 0.3343 | Yes |
| 46 | ADA | na | 3482 | 0.200 | 0.3401 | Yes |
| 47 | PDE8A | na | 3520 | 0.198 | 0.3442 | Yes |
| 48 | POLR3K | na | 3544 | 0.196 | 0.3489 | Yes |
| 49 | PDE6G | na | 3599 | 0.192 | 0.3520 | Yes |
| 50 | ADCY1 | na | 3676 | 0.187 | 0.3540 | Yes |
| 51 | PDE4C | na | 3729 | 0.184 | 0.3569 | Yes |
| 52 | PDE5A | na | 3730 | 0.184 | 0.3623 | Yes |
| 53 | PRPS1 | na | 4330 | 0.141 | 0.3388 | No |
| 54 | AMPD1 | na | 4397 | 0.137 | 0.3398 | No |
| 55 | NUDT5 | na | 4435 | 0.134 | 0.3420 | No |
| 56 | ADCY4 | na | 4754 | 0.111 | 0.3306 | No |
| 57 | POLR3H | na | 4774 | 0.110 | 0.3330 | No |
| 58 | ALLC | na | 4950 | 0.098 | 0.3278 | No |
| 59 | PRIM2 | na | 4961 | 0.098 | 0.3302 | No |
| 60 | PDE1B | na | 5015 | 0.095 | 0.3305 | No |
| 61 | PKM | na | 5020 | 0.095 | 0.3331 | No |
| 62 | ENTPD6 | na | 5034 | 0.094 | 0.3353 | No |
| 63 | NT5M | na | 5239 | 0.083 | 0.3283 | No |
| 64 | POLD3 | na | 5345 | 0.077 | 0.3257 | No |
| 65 | PAICS | na | 5812 | 0.051 | 0.3058 | No |
| 66 | POLR3C | na | 5853 | 0.049 | 0.3054 | No |
| 67 | PDE6D | na | 5925 | 0.045 | 0.3034 | No |
| 68 | RRM2 | na | 6142 | 0.035 | 0.2945 | No |
| 69 | ENPP1 | na | 6350 | 0.027 | 0.2857 | No |
| 70 | ADSL | na | 6539 | 0.019 | 0.2776 | No |
| 71 | GMPS | na | 6561 | 0.018 | 0.2772 | No |
| 72 | AMPD3 | na | 6630 | 0.016 | 0.2745 | No |
| 73 | ENTPD4 | na | 7689 | 0.000 | 0.2258 | No |
| 74 | GDA | na | 8101 | 0.000 | 0.2069 | No |
| 75 | PRPS1L1 | na | 8682 | 0.000 | 0.1802 | No |
| 76 | PDE1C | na | 10739 | 0.000 | 0.0856 | No |
| 77 | PDE6B | na | 10803 | 0.000 | 0.0827 | No |
| 78 | PDE7B | na | 10806 | 0.000 | 0.0826 | No |
| 79 | GUCY2C | na | 11171 | 0.000 | 0.0659 | No |
| 80 | GUCY2D | na | 11172 | 0.000 | 0.0659 | No |
| 81 | NT5C1A | na | 11332 | 0.000 | 0.0585 | No |
| 82 | NT5C1B | na | 11333 | 0.000 | 0.0585 | No |
| 83 | URAD | na | 11563 | 0.000 | 0.0480 | No |
| 84 | ADCY2 | na | 11844 | 0.000 | 0.0351 | No |
| 85 | ADCY8 | na | 11845 | 0.000 | 0.0351 | No |
| 86 | AK5 | na | 13290 | -0.002 | -0.0313 | No |
| 87 | PKLR | na | 13410 | -0.006 | -0.0366 | No |
| 88 | AK1 | na | 13435 | -0.006 | -0.0375 | No |
| 89 | NME5 | na | 13448 | -0.006 | -0.0379 | No |
| 90 | PDE9A | na | 13492 | -0.007 | -0.0397 | No |
| 91 | PDE3A | na | 13506 | -0.007 | -0.0401 | No |
| 92 | POLA2 | na | 13523 | -0.007 | -0.0406 | No |
| 93 | PDE8B | na | 13531 | -0.008 | -0.0407 | No |
| 94 | ENTPD1 | na | 13550 | -0.008 | -0.0413 | No |
| 95 | ADCY9 | na | 13638 | -0.011 | -0.0450 | No |
| 96 | PAPSS1 | na | 14198 | -0.028 | -0.0699 | No |
| 97 | PDE11A | na | 14392 | -0.035 | -0.0777 | No |
| 98 | ENTPD3 | na | 14712 | -0.052 | -0.0909 | No |
| 99 | POLE2 | na | 14842 | -0.058 | -0.0952 | No |
| 100 | FHIT | na | 14852 | -0.059 | -0.0938 | No |
| 101 | PDE6C | na | 14884 | -0.061 | -0.0935 | No |
| 102 | POLD2 | na | 14951 | -0.065 | -0.0946 | No |
| 103 | POLR3G | na | 14958 | -0.065 | -0.0930 | No |
| 104 | PDE4B | na | 15020 | -0.069 | -0.0938 | No |
| 105 | PPAT | na | 15111 | -0.074 | -0.0958 | No |
| 106 | IMPDH1 | na | 15190 | -0.080 | -0.0970 | No |
| 107 | ENTPD8 | na | 15395 | -0.092 | -0.1037 | No |
| 108 | AMPD2 | na | 15607 | -0.104 | -0.1104 | No |
| 109 | RRM1 | na | 15819 | -0.116 | -0.1167 | No |
| 110 | POLR2B | na | 15961 | -0.126 | -0.1195 | No |
| 111 | DCK | na | 16011 | -0.129 | -0.1180 | No |
| 112 | ATIC | na | 16212 | -0.141 | -0.1231 | No |
| 113 | POLR1B | na | 16221 | -0.142 | -0.1193 | No |
| 114 | GART | na | 16226 | -0.142 | -0.1154 | No |
| 115 | PDE3B | na | 16298 | -0.147 | -0.1143 | No |
| 116 | XDH | na | 16830 | -0.184 | -0.1334 | No |
| 117 | POLD1 | na | 17195 | -0.209 | -0.1440 | No |
| 118 | PDE4D | na | 17212 | -0.210 | -0.1386 | No |
| 119 | PRPS2 | na | 17293 | -0.216 | -0.1360 | No |
| 120 | CANT1 | na | 17611 | -0.237 | -0.1437 | No |
| 121 | NT5C2 | na | 17672 | -0.242 | -0.1394 | No |
| 122 | ENTPD5 | na | 17696 | -0.244 | -0.1333 | No |
| 123 | AK4 | na | 17779 | -0.251 | -0.1297 | No |
| 124 | NPR1 | na | 17810 | -0.254 | -0.1237 | No |
| 125 | PDE4A | na | 18018 | -0.271 | -0.1253 | No |
| 126 | RRM2B | na | 18181 | -0.283 | -0.1245 | No |
| 127 | PDE7A | na | 18264 | -0.289 | -0.1199 | No |
| 128 | PDE6A | na | 18292 | -0.291 | -0.1126 | No |
| 129 | POLR3F | na | 18304 | -0.291 | -0.1046 | No |
| 130 | GUCY2F | na | 18368 | -0.295 | -0.0989 | No |
| 131 | POLR3GL | na | 18753 | -0.326 | -0.1071 | No |
| 132 | POLA1 | na | 18783 | -0.329 | -0.0988 | No |
| 133 | PNPT1 | na | 18865 | -0.336 | -0.0927 | No |
| 134 | ADCY10 | na | 19359 | -0.382 | -0.1042 | No |
| 135 | PFAS | na | 19507 | -0.397 | -0.0994 | No |
| 136 | PDE10A | na | 19584 | -0.405 | -0.0911 | No |
| 137 | GUCY1A2 | na | 19793 | -0.425 | -0.0882 | No |
| 138 | NPR2 | na | 19909 | -0.436 | -0.0808 | No |
| 139 | POLR3B | na | 19980 | -0.442 | -0.0711 | No |
| 140 | POLE | na | 20020 | -0.447 | -0.0598 | No |
| 141 | ADCY7 | na | 20088 | -0.453 | -0.0497 | No |
| 142 | POLR1A | na | 20102 | -0.456 | -0.0370 | No |
| 143 | ADCY6 | na | 20181 | -0.465 | -0.0270 | No |
| 144 | ENPP3 | na | 20618 | -0.519 | -0.0319 | No |
| 145 | POLR3A | na | 20712 | -0.531 | -0.0207 | No |
| 146 | POLR2A | na | 21164 | -0.610 | -0.0236 | No |
| 147 | NT5E | na | 21749 | -0.901 | -0.0242 | No |
| 148 | GMPR | na | 21791 | -1.024 | 0.0039 | No |
Table: GSEA details [plain text format]

  

Fig 2: KEGG\_PURINE\_METABOLISM      
 Blue-Pink O' Gram in the Space of the Analyzed GeneSet

  

Fig 3: KEGG\_PURINE\_METABOLISM: Random ES distribution      
 Gene set null distribution of ES for **KEGG\_PURINE\_METABOLISM**
