# Supplementary material for: Regulation of microglia related neuroinflammation contributes to the protective effect of Gelsevirine on ischemic stroke
Source: Front Immunol. 2023 Mar 30;14:1164278. doi: 10.3389/fimmu.2023.1164278 (PMC10098192; doi:10.3389/fimmu.2023.1164278)
Supplement: Supplementary file 6 [file DataSheet_6.zip › fig 5 raw/fig 5-G raw/KEGG.Gsea.1649954846225/KEGG_REGULATION_OF_ACTIN_CYTOSKELETON.html]

Details for gene set KEGG\_REGULATION\_OF\_ACTIN\_CYTOSKELETON[GSEA]

|  || Dataset | OGD\_DRUG\_DRUG.OGD\_FRUG.cls#Gs\_versus\_MCAO.OGD\_FRUG.cls#Gs\_versus\_MCAO\_repos |
| Phenotype | OGD\_FRUG.cls#Gs\_versus\_MCAO\_repos |
| Upregulated in class | MCAO |
| GeneSet | KEGG\_REGULATION\_OF\_ACTIN\_CYTOSKELETON |
| Enrichment Score (ES) | -0.4819405 |
| Normalized Enrichment Score (NES) | -1.5479262 |
| Nominal p-value | 0.0 |
| FDR q-value | 0.06064726 |
| FWER p-Value | 0.603 |
Table: GSEA Results Summary

  

Fig 1: Enrichment plot: KEGG\_REGULATION\_OF\_ACTIN\_CYTOSKELETON      
 Profile of the Running ES Score & Positions of GeneSet Members on the Rank Ordered List

  

| SYMBOL | TITLE | RANK IN GENE LIST | RANK METRIC SCORE | RUNNING ES | CORE ENRICHMENT || 1 | ITGAL | na | 44 | 1.259 | 0.0205 | No |
| 2 | RAC3 | na | 194 | 0.799 | 0.0279 | No |
| 3 | FGF17 | na | 415 | 0.625 | 0.0289 | No |
| 4 | ITGAM | na | 956 | 0.470 | 0.0124 | No |
| 5 | TMSB4X | na | 1014 | 0.462 | 0.0180 | No |
| 6 | BDKRB2 | na | 1078 | 0.453 | 0.0232 | No |
| 7 | BRK1 | na | 1178 | 0.438 | 0.0265 | No |
| 8 | VAV3 | na | 1228 | 0.431 | 0.0319 | No |
| 9 | PIK3CG | na | 1248 | 0.428 | 0.0387 | No |
| 10 | ARPC5L | na | 1304 | 0.421 | 0.0437 | No |
| 11 | ARPC3 | na | 1972 | 0.341 | 0.0190 | No |
| 12 | RHOA | na | 1999 | 0.338 | 0.0238 | No |
| 13 | FGF18 | na | 2160 | 0.321 | 0.0222 | No |
| 14 | FGF21 | na | 2349 | 0.300 | 0.0189 | No |
| 15 | CFL1 | na | 2441 | 0.293 | 0.0199 | No |
| 16 | ITGB7 | na | 2467 | 0.290 | 0.0239 | No |
| 17 | PDGFA | na | 2504 | 0.286 | 0.0274 | No |
| 18 | FGF11 | na | 2523 | 0.284 | 0.0316 | No |
| 19 | MYL12A | na | 2589 | 0.279 | 0.0336 | No |
| 20 | ITGA2B | na | 2646 | 0.275 | 0.0360 | No |
| 21 | HRAS | na | 2716 | 0.268 | 0.0376 | No |
| 22 | MYLPF | na | 3088 | 0.234 | 0.0246 | No |
| 23 | PFN1 | na | 3102 | 0.233 | 0.0282 | No |
| 24 | MYL12B | na | 3105 | 0.233 | 0.0322 | No |
| 25 | RRAS2 | na | 3196 | 0.224 | 0.0321 | No |
| 26 | ITGAE | na | 3360 | 0.210 | 0.0283 | No |
| 27 | FGF22 | na | 3423 | 0.205 | 0.0291 | No |
| 28 | PFN4 | na | 3492 | 0.200 | 0.0296 | No |
| 29 | CYFIP2 | na | 3557 | 0.195 | 0.0301 | No |
| 30 | PPP1CA | na | 3615 | 0.191 | 0.0309 | No |
| 31 | PAK1 | na | 3623 | 0.191 | 0.0340 | No |
| 32 | ARPC1A | na | 3708 | 0.185 | 0.0334 | No |
| 33 | RAC1 | na | 3821 | 0.178 | 0.0314 | No |
| 34 | ARPC2 | na | 4004 | 0.164 | 0.0260 | No |
| 35 | MOS | na | 4263 | 0.146 | 0.0167 | No |
| 36 | CFL2 | na | 4282 | 0.144 | 0.0184 | No |
| 37 | CD14 | na | 4569 | 0.124 | 0.0074 | No |
| 38 | ARPC1B | na | 4583 | 0.123 | 0.0090 | No |
| 39 | ARHGEF6 | na | 4587 | 0.123 | 0.0111 | No |
| 40 | NRAS | na | 4669 | 0.117 | 0.0094 | No |
| 41 | MAP2K2 | na | 4692 | 0.115 | 0.0105 | No |
| 42 | CDC42 | na | 4900 | 0.101 | 0.0027 | No |
| 43 | MAPK3 | na | 4918 | 0.100 | 0.0037 | No |
| 44 | ITGB3 | na | 4968 | 0.097 | 0.0032 | No |
| 45 | ARPC4 | na | 5342 | 0.077 | -0.0126 | No |
| 46 | MYL9 | na | 5490 | 0.069 | -0.0182 | No |
| 47 | ARPC5 | na | 5627 | 0.061 | -0.0234 | No |
| 48 | ACTN2 | na | 5721 | 0.057 | -0.0266 | No |
| 49 | ITGA8 | na | 6033 | 0.039 | -0.0403 | No |
| 50 | FGF9 | na | 6059 | 0.038 | -0.0408 | No |
| 51 | ITGA11 | na | 6089 | 0.037 | -0.0414 | No |
| 52 | MYLK2 | na | 6260 | 0.030 | -0.0488 | No |
| 53 | ITGB8 | na | 6284 | 0.029 | -0.0493 | No |
| 54 | KRAS | na | 6543 | 0.019 | -0.0609 | No |
| 55 | FGF14 | na | 6581 | 0.017 | -0.0623 | No |
| 56 | ITGB6 | na | 6834 | 0.008 | -0.0738 | No |
| 57 | RRAS | na | 6958 | 0.003 | -0.0794 | No |
| 58 | FGF10 | na | 6998 | 0.001 | -0.0812 | No |
| 59 | VAV1 | na | 8205 | 0.000 | -0.1368 | No |
| 60 | CHRM2 | na | 8472 | 0.000 | -0.1491 | No |
| 61 | CHRM3 | na | 8473 | 0.000 | -0.1491 | No |
| 62 | MYL10 | na | 9092 | 0.000 | -0.1776 | No |
| 63 | SCIN | na | 9822 | 0.000 | -0.2113 | No |
| 64 | RAC2 | na | 10018 | 0.000 | -0.2203 | No |
| 65 | FGF20 | na | 10078 | 0.000 | -0.2230 | No |
| 66 | FGF23 | na | 10079 | 0.000 | -0.2230 | No |
| 67 | FGF16 | na | 10085 | 0.000 | -0.2232 | No |
| 68 | FGF13 | na | 10092 | 0.000 | -0.2235 | No |
| 69 | PIP5K1B | na | 10813 | 0.000 | -0.2567 | No |
| 70 | MYL2 | na | 11274 | 0.000 | -0.2779 | No |
| 71 | FGF3 | na | 11852 | 0.000 | -0.3046 | No |
| 72 | FGF4 | na | 11853 | 0.000 | -0.3046 | No |
| 73 | FGF5 | na | 11854 | 0.000 | -0.3046 | No |
| 74 | PFN3 | na | 12207 | 0.000 | -0.3208 | No |
| 75 | INSRR | na | 12228 | 0.000 | -0.3217 | No |
| 76 | ITGA2 | na | 12406 | 0.000 | -0.3299 | No |
| 77 | ITGAD | na | 12410 | 0.000 | -0.3300 | No |
| 78 | FGFR2 | na | 13468 | -0.007 | -0.3787 | No |
| 79 | ITGA7 | na | 13508 | -0.007 | -0.3804 | No |
| 80 | PDGFD | na | 13561 | -0.009 | -0.3826 | No |
| 81 | ITGB5 | na | 13587 | -0.010 | -0.3836 | No |
| 82 | ITGA9 | na | 13709 | -0.013 | -0.3890 | No |
| 83 | ITGA4 | na | 13745 | -0.013 | -0.3904 | No |
| 84 | CHRM1 | na | 13809 | -0.015 | -0.3930 | No |
| 85 | FGF2 | na | 13903 | -0.017 | -0.3970 | No |
| 86 | ACTB | na | 13965 | -0.019 | -0.3995 | No |
| 87 | ITGAX | na | 14076 | -0.023 | -0.4041 | No |
| 88 | PFN2 | na | 14241 | -0.029 | -0.4112 | No |
| 89 | F2 | na | 14453 | -0.038 | -0.4202 | No |
| 90 | BAIAP2 | na | 14737 | -0.053 | -0.4323 | No |
| 91 | FGF8 | na | 14837 | -0.058 | -0.4359 | No |
| 92 | MRAS | na | 14846 | -0.059 | -0.4352 | No |
| 93 | MYLK3 | na | 14999 | -0.068 | -0.4410 | No |
| 94 | FGF6 | na | 15000 | -0.068 | -0.4398 | No |
| 95 | PPP1CB | na | 15011 | -0.068 | -0.4390 | No |
| 96 | SSH3 | na | 15066 | -0.072 | -0.4402 | No |
| 97 | WASF1 | na | 15092 | -0.073 | -0.4401 | No |
| 98 | MAP2K1 | na | 15179 | -0.079 | -0.4426 | No |
| 99 | FGFR4 | na | 15208 | -0.081 | -0.4425 | No |
| 100 | GSN | na | 15411 | -0.093 | -0.4501 | No |
| 101 | PDGFC | na | 15436 | -0.094 | -0.4496 | No |
| 102 | NCKAP1L | na | 15437 | -0.094 | -0.4479 | No |
| 103 | ITGA10 | na | 15634 | -0.105 | -0.4550 | No |
| 104 | ARHGEF4 | na | 15826 | -0.117 | -0.4618 | No |
| 105 | ARAF | na | 15889 | -0.121 | -0.4625 | No |
| 106 | WAS | na | 15945 | -0.125 | -0.4628 | No |
| 107 | MYL7 | na | 15980 | -0.127 | -0.4621 | No |
| 108 | PIP4K2C | na | 16002 | -0.129 | -0.4607 | No |
| 109 | GNG12 | na | 16015 | -0.130 | -0.4590 | No |
| 110 | PIP4K2A | na | 16075 | -0.133 | -0.4593 | No |
| 111 | PPP1CC | na | 16090 | -0.134 | -0.4576 | No |
| 112 | ACTG1 | na | 16172 | -0.139 | -0.4588 | No |
| 113 | MAPK1 | na | 16234 | -0.143 | -0.4591 | No |
| 114 | CSK | na | 16341 | -0.150 | -0.4613 | No |
| 115 | PTK2 | na | 16640 | -0.173 | -0.4720 | No |
| 116 | ITGB1 | na | 16661 | -0.174 | -0.4698 | No |
| 117 | EZR | na | 16704 | -0.177 | -0.4685 | No |
| 118 | ENAH | na | 16759 | -0.180 | -0.4678 | No |
| 119 | CRK | na | 16856 | -0.186 | -0.4689 | No |
| 120 | PIP5K1A | na | 17050 | -0.199 | -0.4743 | No |
| 121 | ACTN3 | na | 17217 | -0.210 | -0.4782 | Yes |
| 122 | RAF1 | na | 17251 | -0.212 | -0.4759 | Yes |
| 123 | LIMK2 | na | 17333 | -0.219 | -0.4757 | Yes |
| 124 | ITGA5 | na | 17393 | -0.223 | -0.4745 | Yes |
| 125 | FGD1 | na | 17422 | -0.225 | -0.4717 | Yes |
| 126 | FGF7 | na | 17518 | -0.231 | -0.4720 | Yes |
| 127 | PDGFB | na | 17563 | -0.234 | -0.4698 | Yes |
| 128 | ITGB4 | na | 17565 | -0.234 | -0.4657 | Yes |
| 129 | PAK2 | na | 17619 | -0.238 | -0.4639 | Yes |
| 130 | ITGB2 | na | 17658 | -0.242 | -0.4613 | Yes |
| 131 | CHRM4 | na | 17690 | -0.243 | -0.4584 | Yes |
| 132 | WASL | na | 17717 | -0.246 | -0.4552 | Yes |
| 133 | BCAR1 | na | 17804 | -0.253 | -0.4546 | Yes |
| 134 | DIAPH1 | na | 17895 | -0.261 | -0.4541 | Yes |
| 135 | F2R | na | 17939 | -0.265 | -0.4514 | Yes |
| 136 | FGFR1 | na | 18053 | -0.273 | -0.4517 | Yes |
| 137 | PAK3 | na | 18126 | -0.279 | -0.4500 | Yes |
| 138 | GIT1 | na | 18141 | -0.281 | -0.4457 | Yes |
| 139 | PIK3CD | na | 18197 | -0.284 | -0.4431 | Yes |
| 140 | PIK3R2 | na | 18436 | -0.299 | -0.4488 | Yes |
| 141 | PIK3CA | na | 18513 | -0.305 | -0.4468 | Yes |
| 142 | NCKAP1 | na | 18526 | -0.306 | -0.4419 | Yes |
| 143 | MYLK | na | 18701 | -0.321 | -0.4442 | Yes |
| 144 | ARHGEF1 | na | 18815 | -0.331 | -0.4435 | Yes |
| 145 | DIAPH3 | na | 18819 | -0.331 | -0.4377 | Yes |
| 146 | GNA13 | na | 18906 | -0.340 | -0.4356 | Yes |
| 147 | ARHGEF7 | na | 18968 | -0.346 | -0.4322 | Yes |
| 148 | EGFR | na | 19043 | -0.354 | -0.4293 | Yes |
| 149 | WASF2 | na | 19160 | -0.366 | -0.4281 | Yes |
| 150 | PPP1R12A | na | 19275 | -0.375 | -0.4267 | Yes |
| 151 | ABI2 | na | 19288 | -0.377 | -0.4205 | Yes |
| 152 | PIK3R3 | na | 19336 | -0.380 | -0.4159 | Yes |
| 153 | MSN | na | 19345 | -0.381 | -0.4094 | Yes |
| 154 | PXN | na | 19391 | -0.386 | -0.4046 | Yes |
| 155 | CRKL | na | 19431 | -0.390 | -0.3994 | Yes |
| 156 | CYFIP1 | na | 19457 | -0.392 | -0.3936 | Yes |
| 157 | ACTN4 | na | 19490 | -0.396 | -0.3880 | Yes |
| 158 | PIK3R5 | na | 19509 | -0.397 | -0.3817 | Yes |
| 159 | SOS2 | na | 19626 | -0.409 | -0.3797 | Yes |
| 160 | PAK4 | na | 19643 | -0.411 | -0.3731 | Yes |
| 161 | FGFR3 | na | 19694 | -0.416 | -0.3680 | Yes |
| 162 | SLC9A1 | na | 19790 | -0.425 | -0.3648 | Yes |
| 163 | ROCK1 | na | 19820 | -0.427 | -0.3585 | Yes |
| 164 | PDGFRA | na | 19955 | -0.440 | -0.3568 | Yes |
| 165 | PIP4K2B | na | 19990 | -0.443 | -0.3504 | Yes |
| 166 | VAV2 | na | 19992 | -0.443 | -0.3426 | Yes |
| 167 | RDX | na | 20023 | -0.447 | -0.3359 | Yes |
| 168 | IQGAP3 | na | 20105 | -0.456 | -0.3315 | Yes |
| 169 | EGF | na | 20244 | -0.471 | -0.3295 | Yes |
| 170 | ITGA6 | na | 20245 | -0.471 | -0.3211 | Yes |
| 171 | PAK6 | na | 20295 | -0.477 | -0.3148 | Yes |
| 172 | CHRM5 | na | 20319 | -0.480 | -0.3073 | Yes |
| 173 | GNA12 | na | 20370 | -0.485 | -0.3009 | Yes |
| 174 | DIAPH2 | na | 20426 | -0.492 | -0.2946 | Yes |
| 175 | FGF12 | na | 20471 | -0.499 | -0.2878 | Yes |
| 176 | SSH1 | na | 20496 | -0.502 | -0.2799 | Yes |
| 177 | DOCK1 | na | 20523 | -0.505 | -0.2721 | Yes |
| 178 | PIK3CB | na | 20535 | -0.507 | -0.2635 | Yes |
| 179 | SOS1 | na | 20563 | -0.511 | -0.2556 | Yes |
| 180 | ACTN1 | na | 20635 | -0.521 | -0.2496 | Yes |
| 181 | ROCK2 | na | 20637 | -0.521 | -0.2403 | Yes |
| 182 | PIKFYVE | na | 20680 | -0.527 | -0.2329 | Yes |
| 183 | ITGA3 | na | 20718 | -0.531 | -0.2251 | Yes |
| 184 | SSH2 | na | 20776 | -0.543 | -0.2180 | Yes |
| 185 | TIAM2 | na | 20783 | -0.544 | -0.2086 | Yes |
| 186 | FGD3 | na | 20786 | -0.544 | -0.1989 | Yes |
| 187 | IQGAP1 | na | 20803 | -0.546 | -0.1899 | Yes |
| 188 | BRAF | na | 20807 | -0.547 | -0.1803 | Yes |
| 189 | LIMK1 | na | 20819 | -0.549 | -0.1710 | Yes |
| 190 | TIAM1 | na | 20825 | -0.550 | -0.1614 | Yes |
| 191 | VCL | na | 20929 | -0.565 | -0.1560 | Yes |
| 192 | PDGFRB | na | 20968 | -0.572 | -0.1475 | Yes |
| 193 | ARHGAP35 | na | 20999 | -0.579 | -0.1386 | Yes |
| 194 | MYH10 | na | 21043 | -0.587 | -0.1301 | Yes |
| 195 | PIP5K1C | na | 21145 | -0.607 | -0.1239 | Yes |
| 196 | APC | na | 21200 | -0.619 | -0.1153 | Yes |
| 197 | ITGAV | na | 21226 | -0.625 | -0.1053 | Yes |
| 198 | MYH9 | na | 21236 | -0.628 | -0.0945 | Yes |
| 199 | ARHGEF12 | na | 21366 | -0.658 | -0.0887 | Yes |
| 200 | FN1 | na | 21407 | -0.672 | -0.0785 | Yes |
| 201 | PIK3R1 | na | 21419 | -0.674 | -0.0669 | Yes |
| 202 | MYH14 | na | 21557 | -0.729 | -0.0602 | Yes |
| 203 | APC2 | na | 21564 | -0.733 | -0.0474 | Yes |
| 204 | IQGAP2 | na | 21626 | -0.770 | -0.0365 | Yes |
| 205 | BDKRB1 | na | 21633 | -0.775 | -0.0229 | Yes |
| 206 | FGF1 | na | 21729 | -0.871 | -0.0117 | Yes |
| 207 | ITGA1 | na | 21792 | -1.028 | 0.0038 | Yes |
Table: GSEA details [plain text format]

  

Fig 2: KEGG\_REGULATION\_OF\_ACTIN\_CYTOSKELETON      
 Blue-Pink O' Gram in the Space of the Analyzed GeneSet

  

Fig 3: KEGG\_REGULATION\_OF\_ACTIN\_CYTOSKELETON: Random ES distribution      
 Gene set null distribution of ES for **KEGG\_REGULATION\_OF\_ACTIN\_CYTOSKELETON**
