# Supplementary material for: Regulation of microglia related neuroinflammation contributes to the protective effect of Gelsevirine on ischemic stroke
Source: Front Immunol. 2023 Mar 30;14:1164278. doi: 10.3389/fimmu.2023.1164278 (PMC10098192; doi:10.3389/fimmu.2023.1164278)
Supplement: Supplementary file 6 [file DataSheet_6.zip › fig 5 raw/fig 5-G raw/KEGG.Gsea.1649954846225/KEGG_RIBOSOME.html]

Details for gene set KEGG\_RIBOSOME[GSEA]

|  || Dataset | OGD\_DRUG\_DRUG.OGD\_FRUG.cls#Gs\_versus\_MCAO.OGD\_FRUG.cls#Gs\_versus\_MCAO\_repos |
| Phenotype | OGD\_FRUG.cls#Gs\_versus\_MCAO\_repos |
| Upregulated in class | Gs |
| GeneSet | KEGG\_RIBOSOME |
| Enrichment Score (ES) | 0.89131516 |
| Normalized Enrichment Score (NES) | 2.8527362 |
| Nominal p-value | 0.0 |
| FDR q-value | 0.0 |
| FWER p-Value | 0.0 |
Table: GSEA Results Summary

  

Fig 1: Enrichment plot: KEGG\_RIBOSOME      
 Profile of the Running ES Score & Positions of GeneSet Members on the Rank Ordered List

  

| SYMBOL | TITLE | RANK IN GENE LIST | RANK METRIC SCORE | RUNNING ES | CORE ENRICHMENT || 1 | RPS27A | na | 273 | 0.718 | 0.0059 | Yes |
| 2 | RPL22L1 | na | 311 | 0.691 | 0.0218 | Yes |
| 3 | RPS27 | na | 315 | 0.684 | 0.0392 | Yes |
| 4 | RPS15A | na | 339 | 0.668 | 0.0552 | Yes |
| 5 | RPS4X | na | 353 | 0.656 | 0.0714 | Yes |
| 6 | RPL39 | na | 380 | 0.641 | 0.0866 | Yes |
| 7 | RPS7 | na | 424 | 0.621 | 0.1006 | Yes |
| 8 | RPL7 | na | 467 | 0.601 | 0.1140 | Yes |
| 9 | RPL5 | na | 468 | 0.601 | 0.1294 | Yes |
| 10 | RPL14 | na | 508 | 0.587 | 0.1426 | Yes |
| 11 | RPS3 | na | 511 | 0.586 | 0.1575 | Yes |
| 12 | RPL22 | na | 517 | 0.582 | 0.1722 | Yes |
| 13 | RPS11 | na | 542 | 0.572 | 0.1857 | Yes |
| 14 | RPL36A | na | 548 | 0.571 | 0.2001 | Yes |
| 15 | RPL9 | na | 557 | 0.569 | 0.2143 | Yes |
| 16 | RPS6 | na | 566 | 0.566 | 0.2285 | Yes |
| 17 | RPS24 | na | 582 | 0.560 | 0.2421 | Yes |
| 18 | RPL19 | na | 620 | 0.548 | 0.2544 | Yes |
| 19 | RPL30 | na | 648 | 0.539 | 0.2670 | Yes |
| 20 | RPL23 | na | 666 | 0.534 | 0.2799 | Yes |
| 21 | RPL11 | na | 669 | 0.534 | 0.2934 | Yes |
| 22 | RPL26 | na | 690 | 0.529 | 0.3061 | Yes |
| 23 | RPL34 | na | 691 | 0.529 | 0.3196 | Yes |
| 24 | RPS25 | na | 731 | 0.517 | 0.3310 | Yes |
| 25 | RPL17 | na | 747 | 0.512 | 0.3434 | Yes |
| 26 | RPL37A | na | 756 | 0.509 | 0.3561 | Yes |
| 27 | RPS19 | na | 763 | 0.508 | 0.3688 | Yes |
| 28 | RPS17 | na | 779 | 0.503 | 0.3810 | Yes |
| 29 | RPS27L | na | 783 | 0.502 | 0.3937 | Yes |
| 30 | RPL23A | na | 789 | 0.500 | 0.4063 | Yes |
| 31 | RPL6 | na | 808 | 0.497 | 0.4182 | Yes |
| 32 | RPL10 | na | 833 | 0.491 | 0.4296 | Yes |
| 33 | RPS12 | na | 835 | 0.491 | 0.4422 | Yes |
| 34 | RPL38 | na | 836 | 0.491 | 0.4547 | Yes |
| 35 | RPS21 | na | 853 | 0.486 | 0.4664 | Yes |
| 36 | RPL21 | na | 873 | 0.482 | 0.4779 | Yes |
| 37 | RPS18 | na | 901 | 0.479 | 0.4889 | Yes |
| 38 | RPS23 | na | 945 | 0.471 | 0.4990 | Yes |
| 39 | RPS20 | na | 964 | 0.469 | 0.5102 | Yes |
| 40 | RPS16 | na | 974 | 0.468 | 0.5217 | Yes |
| 41 | RPL3 | na | 988 | 0.466 | 0.5331 | Yes |
| 42 | RPL12 | na | 1004 | 0.464 | 0.5442 | Yes |
| 43 | RPL24 | na | 1008 | 0.463 | 0.5560 | Yes |
| 44 | RPS13 | na | 1017 | 0.462 | 0.5674 | Yes |
| 45 | RPL4 | na | 1046 | 0.458 | 0.5778 | Yes |
| 46 | RPL32 | na | 1077 | 0.454 | 0.5881 | Yes |
| 47 | RPL31 | na | 1100 | 0.448 | 0.5985 | Yes |
| 48 | RPSA | na | 1107 | 0.447 | 0.6097 | Yes |
| 49 | RPS8 | na | 1116 | 0.446 | 0.6207 | Yes |
| 50 | RPL10A | na | 1122 | 0.445 | 0.6319 | Yes |
| 51 | RPL35A | na | 1129 | 0.444 | 0.6430 | Yes |
| 52 | RPL35 | na | 1132 | 0.444 | 0.6543 | Yes |
| 53 | RPL13A | na | 1135 | 0.444 | 0.6655 | Yes |
| 54 | RPL18 | na | 1139 | 0.443 | 0.6767 | Yes |
| 55 | RPL36AL | na | 1159 | 0.440 | 0.6871 | Yes |
| 56 | RPS26 | na | 1174 | 0.438 | 0.6977 | Yes |
| 57 | RPS9 | na | 1185 | 0.436 | 0.7084 | Yes |
| 58 | RPL37 | na | 1187 | 0.436 | 0.7195 | Yes |
| 59 | RPS5 | na | 1199 | 0.434 | 0.7301 | Yes |
| 60 | RPL27 | na | 1243 | 0.429 | 0.7391 | Yes |
| 61 | RPS29 | na | 1244 | 0.429 | 0.7501 | Yes |
| 62 | RPLP2 | na | 1291 | 0.423 | 0.7588 | Yes |
| 63 | RSL24D1 | na | 1293 | 0.422 | 0.7696 | Yes |
| 64 | RPLP1 | na | 1301 | 0.421 | 0.7800 | Yes |
| 65 | RPL27A | na | 1302 | 0.421 | 0.7908 | Yes |
| 66 | RPS10 | na | 1307 | 0.420 | 0.8013 | Yes |
| 67 | RPS15 | na | 1312 | 0.419 | 0.8119 | Yes |
| 68 | FAU | na | 1340 | 0.416 | 0.8213 | Yes |
| 69 | RPL18A | na | 1374 | 0.413 | 0.8303 | Yes |
| 70 | UBA52 | na | 1408 | 0.410 | 0.8393 | Yes |
| 71 | RPL36 | na | 1483 | 0.401 | 0.8462 | Yes |
| 72 | RPL41 | na | 1508 | 0.399 | 0.8553 | Yes |
| 73 | RPS28 | na | 1544 | 0.394 | 0.8638 | Yes |
| 74 | RPL8 | na | 1559 | 0.392 | 0.8732 | Yes |
| 75 | MRPL13 | na | 1670 | 0.378 | 0.8778 | Yes |
| 76 | RPL28 | na | 1732 | 0.370 | 0.8845 | Yes |
| 77 | RPL13 | na | 1787 | 0.364 | 0.8913 | Yes |
| 78 | RPS2 | na | 2161 | 0.321 | 0.8824 | No |
| 79 | RPL7A | na | 2698 | 0.270 | 0.8647 | No |
| 80 | RPL29 | na | 3618 | 0.191 | 0.8274 | No |
| 81 | RPLP0 | na | 3826 | 0.178 | 0.8225 | No |
| 82 | RPL3L | na | 4515 | 0.128 | 0.7942 | No |
| 83 | RPL15 | na | 5074 | 0.092 | 0.7709 | No |
| 84 | RPL10L | na | 8161 | 0.000 | 0.6293 | No |
Table: GSEA details [plain text format]

  

Fig 2: KEGG\_RIBOSOME      
 Blue-Pink O' Gram in the Space of the Analyzed GeneSet

  

Fig 3: KEGG\_RIBOSOME: Random ES distribution      
 Gene set null distribution of ES for **KEGG\_RIBOSOME**
