# Supplementary material for: Regulation of microglia related neuroinflammation contributes to the protective effect of Gelsevirine on ischemic stroke
Source: Front Immunol. 2023 Mar 30;14:1164278. doi: 10.3389/fimmu.2023.1164278 (PMC10098192; doi:10.3389/fimmu.2023.1164278)
Supplement: Supplementary file 6 [file DataSheet_6.zip › fig 5 raw/fig 5-G raw/KEGG.Gsea.1649954846225/KEGG_SMALL_CELL_LUNG_CANCER.html]

Details for gene set KEGG\_SMALL\_CELL\_LUNG\_CANCER[GSEA]

|  || Dataset | OGD\_DRUG\_DRUG.OGD\_FRUG.cls#Gs\_versus\_MCAO.OGD\_FRUG.cls#Gs\_versus\_MCAO\_repos |
| Phenotype | OGD\_FRUG.cls#Gs\_versus\_MCAO\_repos |
| Upregulated in class | MCAO |
| GeneSet | KEGG\_SMALL\_CELL\_LUNG\_CANCER |
| Enrichment Score (ES) | -0.539913 |
| Normalized Enrichment Score (NES) | -1.5889364 |
| Nominal p-value | 0.0 |
| FDR q-value | 0.04311433 |
| FWER p-Value | 0.427 |
Table: GSEA Results Summary

  

Fig 1: Enrichment plot: KEGG\_SMALL\_CELL\_LUNG\_CANCER      
 Profile of the Running ES Score & Positions of GeneSet Members on the Rank Ordered List

  

| SYMBOL | TITLE | RANK IN GENE LIST | RANK METRIC SCORE | RUNNING ES | CORE ENRICHMENT || 1 | LAMC3 | na | 692 | 0.529 | -0.0080 | No |
| 2 | PIK3CG | na | 1248 | 0.428 | -0.0143 | No |
| 3 | CYCS | na | 1320 | 0.418 | 0.0011 | No |
| 4 | CKS1B | na | 2057 | 0.332 | -0.0178 | No |
| 5 | LAMB3 | na | 2281 | 0.306 | -0.0143 | No |
| 6 | LAMA3 | na | 2493 | 0.287 | -0.0111 | No |
| 7 | ITGA2B | na | 2646 | 0.275 | -0.0058 | No |
| 8 | CDK4 | na | 3240 | 0.219 | -0.0231 | No |
| 9 | LAMA4 | na | 3690 | 0.187 | -0.0354 | No |
| 10 | NFKBIA | na | 3820 | 0.179 | -0.0333 | No |
| 11 | MAX | na | 3996 | 0.165 | -0.0339 | No |
| 12 | LAMC2 | na | 4333 | 0.141 | -0.0430 | No |
| 13 | BCL2L1 | na | 4927 | 0.100 | -0.0658 | No |
| 14 | MYC | na | 5587 | 0.064 | -0.0932 | No |
| 15 | NOS2 | na | 5724 | 0.057 | -0.0969 | No |
| 16 | BIRC2 | na | 5775 | 0.053 | -0.0968 | No |
| 17 | CDKN1B | na | 5942 | 0.045 | -0.1024 | No |
| 18 | SKP2 | na | 5968 | 0.043 | -0.1016 | No |
| 19 | TRAF2 | na | 6244 | 0.031 | -0.1129 | No |
| 20 | CDK2 | na | 6514 | 0.020 | -0.1243 | No |
| 21 | RXRG | na | 8397 | 0.000 | -0.2107 | No |
| 22 | CDKN2B | na | 10239 | 0.000 | -0.2951 | No |
| 23 | ITGA2 | na | 12406 | 0.000 | -0.3945 | No |
| 24 | CCNE2 | na | 13270 | -0.001 | -0.4341 | No |
| 25 | LAMA1 | na | 13573 | -0.009 | -0.4475 | No |
| 26 | RXRB | na | 14636 | -0.047 | -0.4941 | No |
| 27 | IKBKG | na | 14695 | -0.050 | -0.4945 | No |
| 28 | FHIT | na | 14852 | -0.059 | -0.4990 | No |
| 29 | TRAF1 | na | 15135 | -0.076 | -0.5086 | No |
| 30 | TRAF4 | na | 15537 | -0.100 | -0.5225 | No |
| 31 | RELA | na | 15584 | -0.103 | -0.5200 | No |
| 32 | PIAS2 | na | 15673 | -0.107 | -0.5192 | No |
| 33 | E2F1 | na | 15769 | -0.113 | -0.5185 | No |
| 34 | PIAS4 | na | 15887 | -0.121 | -0.5185 | No |
| 35 | RARB | na | 16048 | -0.131 | -0.5199 | No |
| 36 | PTGS2 | na | 16449 | -0.158 | -0.5312 | No |
| 37 | PTK2 | na | 16640 | -0.173 | -0.5322 | Yes |
| 38 | ITGB1 | na | 16661 | -0.174 | -0.5253 | Yes |
| 39 | CCNE1 | na | 16729 | -0.178 | -0.5204 | Yes |
| 40 | E2F3 | na | 16736 | -0.179 | -0.5127 | Yes |
| 41 | AKT2 | na | 16784 | -0.181 | -0.5067 | Yes |
| 42 | IKBKB | na | 16790 | -0.182 | -0.4988 | Yes |
| 43 | RB1 | na | 16796 | -0.182 | -0.4908 | Yes |
| 44 | AKT1 | na | 16827 | -0.184 | -0.4840 | Yes |
| 45 | TRAF5 | na | 17214 | -0.210 | -0.4922 | Yes |
| 46 | BIRC3 | na | 17295 | -0.216 | -0.4862 | Yes |
| 47 | PTEN | na | 17342 | -0.220 | -0.4785 | Yes |
| 48 | CCND1 | na | 17421 | -0.225 | -0.4720 | Yes |
| 49 | BCL2 | na | 17522 | -0.232 | -0.4662 | Yes |
| 50 | CHUK | na | 17623 | -0.238 | -0.4601 | Yes |
| 51 | PIAS1 | na | 17735 | -0.247 | -0.4541 | Yes |
| 52 | E2F2 | na | 17853 | -0.258 | -0.4480 | Yes |
| 53 | AKT3 | na | 17899 | -0.261 | -0.4383 | Yes |
| 54 | PIK3CD | na | 18197 | -0.284 | -0.4392 | Yes |
| 55 | PIK3R2 | na | 18436 | -0.299 | -0.4367 | Yes |
| 56 | LAMB1 | na | 18455 | -0.300 | -0.4241 | Yes |
| 57 | PIK3CA | na | 18513 | -0.305 | -0.4131 | Yes |
| 58 | APAF1 | na | 18709 | -0.321 | -0.4076 | Yes |
| 59 | TRAF6 | na | 18711 | -0.321 | -0.3932 | Yes |
| 60 | LAMA5 | na | 19183 | -0.368 | -0.3983 | Yes |
| 61 | RXRA | na | 19209 | -0.370 | -0.3829 | Yes |
| 62 | NFKB1 | na | 19213 | -0.371 | -0.3664 | Yes |
| 63 | CASP9 | na | 19243 | -0.373 | -0.3510 | Yes |
| 64 | PIK3R3 | na | 19336 | -0.380 | -0.3382 | Yes |
| 65 | PIK3R5 | na | 19509 | -0.397 | -0.3283 | Yes |
| 66 | LAMA2 | na | 20114 | -0.457 | -0.3356 | Yes |
| 67 | LAMB2 | na | 20135 | -0.460 | -0.3159 | Yes |
| 68 | TRAF3 | na | 20180 | -0.465 | -0.2970 | Yes |
| 69 | ITGA6 | na | 20245 | -0.471 | -0.2789 | Yes |
| 70 | XIAP | na | 20306 | -0.478 | -0.2602 | Yes |
| 71 | PIK3CB | na | 20535 | -0.507 | -0.2479 | Yes |
| 72 | CDK6 | na | 20612 | -0.518 | -0.2282 | Yes |
| 73 | ITGA3 | na | 20718 | -0.531 | -0.2092 | Yes |
| 74 | COL4A1 | na | 20907 | -0.561 | -0.1927 | Yes |
| 75 | PIAS3 | na | 20932 | -0.565 | -0.1685 | Yes |
| 76 | ITGAV | na | 21226 | -0.625 | -0.1539 | Yes |
| 77 | COL4A6 | na | 21228 | -0.627 | -0.1259 | Yes |
| 78 | LAMC1 | na | 21239 | -0.629 | -0.0981 | Yes |
| 79 | COL4A2 | na | 21287 | -0.639 | -0.0716 | Yes |
| 80 | FN1 | na | 21407 | -0.672 | -0.0470 | Yes |
| 81 | PIK3R1 | na | 21419 | -0.674 | -0.0173 | Yes |
| 82 | COL4A4 | na | 21703 | -0.851 | 0.0079 | Yes |
Table: GSEA details [plain text format]

  

Fig 2: KEGG\_SMALL\_CELL\_LUNG\_CANCER      
 Blue-Pink O' Gram in the Space of the Analyzed GeneSet

  

Fig 3: KEGG\_SMALL\_CELL\_LUNG\_CANCER: Random ES distribution      
 Gene set null distribution of ES for **KEGG\_SMALL\_CELL\_LUNG\_CANCER**
