# Supplementary material for: Regulation of microglia related neuroinflammation contributes to the protective effect of Gelsevirine on ischemic stroke
Source: Front Immunol. 2023 Mar 30;14:1164278. doi: 10.3389/fimmu.2023.1164278 (PMC10098192; doi:10.3389/fimmu.2023.1164278)
Supplement: Supplementary file 6 [file DataSheet_6.zip › fig 5 raw/fig 5-G raw/KEGG.Gsea.1649954846225/KEGG_SPLICEOSOME.html]

Details for gene set KEGG\_SPLICEOSOME[GSEA]

|  || Dataset | OGD\_DRUG\_DRUG.OGD\_FRUG.cls#Gs\_versus\_MCAO.OGD\_FRUG.cls#Gs\_versus\_MCAO\_repos |
| Phenotype | OGD\_FRUG.cls#Gs\_versus\_MCAO\_repos |
| Upregulated in class | Gs |
| GeneSet | KEGG\_SPLICEOSOME |
| Enrichment Score (ES) | 0.49839094 |
| Normalized Enrichment Score (NES) | 1.6808692 |
| Nominal p-value | 0.0 |
| FDR q-value | 0.026331458 |
| FWER p-Value | 0.189 |
Table: GSEA Results Summary

  

Fig 1: Enrichment plot: KEGG\_SPLICEOSOME      
 Profile of the Running ES Score & Positions of GeneSet Members on the Rank Ordered List

  

| SYMBOL | TITLE | RANK IN GENE LIST | RANK METRIC SCORE | RUNNING ES | CORE ENRICHMENT || 1 | PPIH | na | 191 | 0.804 | 0.0176 | Yes |
| 2 | LSM7 | na | 400 | 0.631 | 0.0287 | Yes |
| 3 | BUD31 | na | 473 | 0.598 | 0.0450 | Yes |
| 4 | CCDC12 | na | 633 | 0.544 | 0.0556 | Yes |
| 5 | MAGOHB | na | 685 | 0.530 | 0.0706 | Yes |
| 6 | SNRPG | na | 703 | 0.525 | 0.0871 | Yes |
| 7 | SNRPD2 | na | 767 | 0.507 | 0.1008 | Yes |
| 8 | SNRPD1 | na | 781 | 0.502 | 0.1166 | Yes |
| 9 | LSM3 | na | 794 | 0.500 | 0.1325 | Yes |
| 10 | SYF2 | na | 812 | 0.495 | 0.1479 | Yes |
| 11 | SNRNP27 | na | 923 | 0.474 | 0.1584 | Yes |
| 12 | LSM5 | na | 924 | 0.474 | 0.1740 | Yes |
| 13 | PHF5A | na | 933 | 0.473 | 0.1891 | Yes |
| 14 | SNRPB2 | na | 949 | 0.471 | 0.2039 | Yes |
| 15 | SNRPD3 | na | 993 | 0.466 | 0.2172 | Yes |
| 16 | SNRPE | na | 1160 | 0.440 | 0.2240 | Yes |
| 17 | RBM8A | na | 1217 | 0.433 | 0.2356 | Yes |
| 18 | SNRPF | na | 1261 | 0.426 | 0.2476 | Yes |
| 19 | SNRPA1 | na | 1357 | 0.414 | 0.2568 | Yes |
| 20 | SF3B6 | na | 1362 | 0.414 | 0.2702 | Yes |
| 21 | BCAS2 | na | 1370 | 0.413 | 0.2835 | Yes |
| 22 | CWC15 | na | 1431 | 0.407 | 0.2941 | Yes |
| 23 | U2AF1 | na | 1531 | 0.396 | 0.3025 | Yes |
| 24 | ISY1 | na | 1558 | 0.392 | 0.3142 | Yes |
| 25 | HSPA1B | na | 1633 | 0.382 | 0.3233 | Yes |
| 26 | SMNDC1 | na | 1698 | 0.375 | 0.3327 | Yes |
| 27 | MAGOH | na | 1730 | 0.370 | 0.3434 | Yes |
| 28 | ALYREF | na | 1743 | 0.369 | 0.3549 | Yes |
| 29 | LSM4 | na | 1859 | 0.355 | 0.3613 | Yes |
| 30 | SRSF3 | na | 1888 | 0.351 | 0.3715 | Yes |
| 31 | PRPF38A | na | 2023 | 0.335 | 0.3764 | Yes |
| 32 | LSM2 | na | 2035 | 0.334 | 0.3868 | Yes |
| 33 | TXNL4A | na | 2046 | 0.333 | 0.3973 | Yes |
| 34 | LSM8 | na | 2089 | 0.329 | 0.4061 | Yes |
| 35 | SRSF7 | na | 2163 | 0.321 | 0.4133 | Yes |
| 36 | RBM17 | na | 2239 | 0.311 | 0.4200 | Yes |
| 37 | TRA2A | na | 2262 | 0.308 | 0.4291 | Yes |
| 38 | THOC1 | na | 2275 | 0.306 | 0.4386 | Yes |
| 39 | PPIE | na | 2318 | 0.302 | 0.4466 | Yes |
| 40 | SNRNP70 | na | 2420 | 0.294 | 0.4516 | Yes |
| 41 | HNRNPC | na | 2434 | 0.293 | 0.4606 | Yes |
| 42 | LSM6 | na | 2464 | 0.290 | 0.4688 | Yes |
| 43 | HSPA8 | na | 2800 | 0.259 | 0.4619 | Yes |
| 44 | SNRNP40 | na | 2880 | 0.252 | 0.4665 | Yes |
| 45 | ZMAT2 | na | 2903 | 0.250 | 0.4737 | Yes |
| 46 | SNW1 | na | 2917 | 0.249 | 0.4813 | Yes |
| 47 | SNRPC | na | 2965 | 0.244 | 0.4871 | Yes |
| 48 | SF3A3 | na | 2976 | 0.244 | 0.4947 | Yes |
| 49 | PQBP1 | na | 3064 | 0.236 | 0.4984 | Yes |
| 50 | NCBP2 | na | 3367 | 0.209 | 0.4914 | No |
| 51 | SNRPB | na | 3406 | 0.206 | 0.4964 | No |
| 52 | SF3B5 | na | 3763 | 0.182 | 0.4860 | No |
| 53 | CTNNBL1 | na | 3791 | 0.180 | 0.4906 | No |
| 54 | PLRG1 | na | 3925 | 0.171 | 0.4901 | No |
| 55 | HNRNPA3 | na | 3970 | 0.167 | 0.4936 | No |
| 56 | PPIL1 | na | 4036 | 0.161 | 0.4959 | No |
| 57 | DDX39B | na | 4122 | 0.155 | 0.4970 | No |
| 58 | SRSF9 | na | 4811 | 0.107 | 0.4689 | No |
| 59 | SRSF5 | na | 4816 | 0.107 | 0.4723 | No |
| 60 | SRSF2 | na | 4990 | 0.096 | 0.4675 | No |
| 61 | PRPF19 | na | 5040 | 0.093 | 0.4683 | No |
| 62 | TRA2B | na | 5061 | 0.092 | 0.4704 | No |
| 63 | SART1 | na | 5266 | 0.081 | 0.4637 | No |
| 64 | HNRNPA1 | na | 5313 | 0.079 | 0.4641 | No |
| 65 | EIF4A3 | na | 5418 | 0.073 | 0.4617 | No |
| 66 | PUF60 | na | 5462 | 0.070 | 0.4621 | No |
| 67 | HSPA1A | na | 5784 | 0.053 | 0.4490 | No |
| 68 | CRNKL1 | na | 5869 | 0.048 | 0.4468 | No |
| 69 | PRPF3 | na | 5928 | 0.045 | 0.4456 | No |
| 70 | SNRPA | na | 6081 | 0.037 | 0.4398 | No |
| 71 | U2SURP | na | 6084 | 0.037 | 0.4409 | No |
| 72 | NCBP1 | na | 6154 | 0.034 | 0.4389 | No |
| 73 | SRSF1 | na | 6341 | 0.027 | 0.4312 | No |
| 74 | THOC3 | na | 6568 | 0.018 | 0.4214 | No |
| 75 | PRPF31 | na | 6572 | 0.018 | 0.4219 | No |
| 76 | PRPF4 | na | 6611 | 0.016 | 0.4207 | No |
| 77 | SRSF6 | na | 6659 | 0.015 | 0.4190 | No |
| 78 | SLU7 | na | 6664 | 0.014 | 0.4193 | No |
| 79 | HNRNPM | na | 6839 | 0.008 | 0.4115 | No |
| 80 | XAB2 | na | 6859 | 0.006 | 0.4109 | No |
| 81 | RBM25 | na | 7001 | 0.001 | 0.4044 | No |
| 82 | DHX16 | na | 13841 | -0.016 | 0.0905 | No |
| 83 | DDX5 | na | 13863 | -0.016 | 0.0901 | No |
| 84 | PRPF6 | na | 13969 | -0.019 | 0.0859 | No |
| 85 | SRSF10 | na | 14060 | -0.022 | 0.0825 | No |
| 86 | PRPF38B | na | 14257 | -0.030 | 0.0745 | No |
| 87 | CDC5L | na | 14269 | -0.030 | 0.0750 | No |
| 88 | SF3B2 | na | 14323 | -0.033 | 0.0736 | No |
| 89 | SRSF4 | na | 14615 | -0.046 | 0.0617 | No |
| 90 | HNRNPK | na | 14654 | -0.048 | 0.0616 | No |
| 91 | USP39 | na | 15049 | -0.071 | 0.0458 | No |
| 92 | HNRNPU | na | 15171 | -0.078 | 0.0428 | No |
| 93 | PRPF18 | na | 15318 | -0.087 | 0.0389 | No |
| 94 | DHX15 | na | 15439 | -0.094 | 0.0365 | No |
| 95 | U2AF2 | na | 15575 | -0.102 | 0.0337 | No |
| 96 | DDX42 | na | 15828 | -0.117 | 0.0259 | No |
| 97 | ACIN1 | na | 15913 | -0.123 | 0.0261 | No |
| 98 | RBM22 | na | 16145 | -0.137 | 0.0200 | No |
| 99 | PRPF40A | na | 16270 | -0.145 | 0.0190 | No |
| 100 | RBMX | na | 16273 | -0.145 | 0.0237 | No |
| 101 | WBP11 | na | 16471 | -0.160 | 0.0199 | No |
| 102 | DHX38 | na | 16615 | -0.170 | 0.0189 | No |
| 103 | PCBP1 | na | 16717 | -0.178 | 0.0201 | No |
| 104 | DDX23 | na | 17239 | -0.212 | 0.0031 | No |
| 105 | DDX46 | na | 17354 | -0.220 | 0.0051 | No |
| 106 | SF3A2 | na | 17534 | -0.232 | 0.0044 | No |
| 107 | SF3B4 | na | 17694 | -0.244 | 0.0051 | No |
| 108 | SF3B3 | na | 18079 | -0.274 | -0.0035 | No |
| 109 | SF3B1 | na | 18265 | -0.289 | -0.0026 | No |
| 110 | HSPA1L | na | 18395 | -0.297 | 0.0012 | No |
| 111 | DHX8 | na | 18533 | -0.307 | 0.0050 | No |
| 112 | PRPF40B | na | 18566 | -0.309 | 0.0137 | No |
| 113 | CDC40 | na | 18856 | -0.335 | 0.0114 | No |
| 114 | AQR | na | 19029 | -0.352 | 0.0150 | No |
| 115 | HSPA2 | na | 19041 | -0.354 | 0.0261 | No |
| 116 | SF3A1 | na | 19100 | -0.359 | 0.0352 | No |
| 117 | TCERG1 | na | 19116 | -0.362 | 0.0464 | No |
| 118 | EFTUD2 | na | 19323 | -0.379 | 0.0494 | No |
| 119 | CHERP | na | 20080 | -0.452 | 0.0295 | No |
| 120 | THOC2 | na | 20513 | -0.503 | 0.0261 | No |
| 121 | SNRNP200 | na | 20608 | -0.517 | 0.0388 | No |
| 122 | PRPF8 | na | 21069 | -0.592 | 0.0371 | No |
Table: GSEA details [plain text format]

  

Fig 2: KEGG\_SPLICEOSOME      
 Blue-Pink O' Gram in the Space of the Analyzed GeneSet

  

Fig 3: KEGG\_SPLICEOSOME: Random ES distribution      
 Gene set null distribution of ES for **KEGG\_SPLICEOSOME**
