# Supplementary material for: Regulation of microglia related neuroinflammation contributes to the protective effect of Gelsevirine on ischemic stroke
Source: Front Immunol. 2023 Mar 30;14:1164278. doi: 10.3389/fimmu.2023.1164278 (PMC10098192; doi:10.3389/fimmu.2023.1164278)
Supplement: Supplementary file 6 [file DataSheet_6.zip › fig 5 raw/fig 5-G raw/KEGG.Gsea.1649954846225/KEGG_TYPE_I_DIABETES_MELLITUS.html]

Details for gene set KEGG\_TYPE\_I\_DIABETES\_MELLITUS[GSEA]

|  || Dataset | OGD\_DRUG\_DRUG.OGD\_FRUG.cls#Gs\_versus\_MCAO.OGD\_FRUG.cls#Gs\_versus\_MCAO\_repos |
| Phenotype | OGD\_FRUG.cls#Gs\_versus\_MCAO\_repos |
| Upregulated in class | Gs |
| GeneSet | KEGG\_TYPE\_I\_DIABETES\_MELLITUS |
| Enrichment Score (ES) | 0.5715425 |
| Normalized Enrichment Score (NES) | 1.386713 |
| Nominal p-value | 0.06699752 |
| FDR q-value | 0.20331237 |
| FWER p-Value | 0.919 |
Table: GSEA Results Summary

  

Fig 1: Enrichment plot: KEGG\_TYPE\_I\_DIABETES\_MELLITUS      
 Profile of the Running ES Score & Positions of GeneSet Members on the Rank Ordered List

  

| SYMBOL | TITLE | RANK IN GENE LIST | RANK METRIC SCORE | RUNNING ES | CORE ENRICHMENT || 1 | IL12B | na | 3745 | 0.183 | 0.0338 | Yes |
| 2 | CPE | na | 4096 | 0.156 | 0.1930 | Yes |
| 3 | HSPD1 | na | 4188 | 0.150 | 0.3569 | Yes |
| 4 | CD80 | na | 4554 | 0.125 | 0.4800 | Yes |
| 5 | IL2 | na | 5219 | 0.084 | 0.5435 | Yes |
| 6 | TNF | na | 5838 | 0.050 | 0.5715 | Yes |
| 7 | FAS | na | 6465 | 0.023 | 0.5682 | No |
| 8 | GAD2 | na | 6803 | 0.009 | 0.5634 | No |
| 9 | PTPRN2 | na | 7928 | 0.000 | 0.5120 | No |
| 10 | PRF1 | na | 8359 | 0.000 | 0.4923 | No |
| 11 | GZMB | na | 8650 | 0.000 | 0.4790 | No |
| 12 | IFNG | na | 9828 | 0.000 | 0.4252 | No |
| 13 | GAD1 | na | 10035 | 0.000 | 0.4158 | No |
| 14 | IL1A | na | 10924 | 0.000 | 0.3751 | No |
| 15 | IL12A | na | 12867 | 0.000 | 0.2863 | No |
| 16 | CD28 | na | 12883 | 0.000 | 0.2856 | No |
| 17 | CD86 | na | 12918 | 0.000 | 0.2840 | No |
| 18 | ICA1 | na | 13395 | -0.005 | 0.2679 | No |
| 19 | LTA | na | 13822 | -0.015 | 0.2653 | No |
| 20 | IL1B | na | 14311 | -0.032 | 0.2794 | No |
| 21 | PTPRN | na | 14863 | -0.059 | 0.3208 | No |
Table: GSEA details [plain text format]

  

Fig 2: KEGG\_TYPE\_I\_DIABETES\_MELLITUS      
 Blue-Pink O' Gram in the Space of the Analyzed GeneSet

  

Fig 3: KEGG\_TYPE\_I\_DIABETES\_MELLITUS: Random ES distribution      
 Gene set null distribution of ES for **KEGG\_TYPE\_I\_DIABETES\_MELLITUS**
