# Supplementary material for: Regulation of microglia related neuroinflammation contributes to the protective effect of Gelsevirine on ischemic stroke
Source: Front Immunol. 2023 Mar 30;14:1164278. doi: 10.3389/fimmu.2023.1164278 (PMC10098192; doi:10.3389/fimmu.2023.1164278)
Supplement: Supplementary file 6 [file DataSheet_6.zip › fig 5 raw/fig 5-G raw/KEGG.Gsea.1649954846225/KEGG_TYPE_II_DIABETES_MELLITUS.html]

Details for gene set KEGG\_TYPE\_II\_DIABETES\_MELLITUS[GSEA]

|  || Dataset | OGD\_DRUG\_DRUG.OGD\_FRUG.cls#Gs\_versus\_MCAO.OGD\_FRUG.cls#Gs\_versus\_MCAO\_repos |
| Phenotype | OGD\_FRUG.cls#Gs\_versus\_MCAO\_repos |
| Upregulated in class | MCAO |
| GeneSet | KEGG\_TYPE\_II\_DIABETES\_MELLITUS |
| Enrichment Score (ES) | -0.63700175 |
| Normalized Enrichment Score (NES) | -1.6995527 |
| Nominal p-value | 0.0 |
| FDR q-value | 0.015243112 |
| FWER p-Value | 0.096 |
Table: GSEA Results Summary

  

Fig 1: Enrichment plot: KEGG\_TYPE\_II\_DIABETES\_MELLITUS      
 Profile of the Running ES Score & Positions of GeneSet Members on the Rank Ordered List

  

| SYMBOL | TITLE | RANK IN GENE LIST | RANK METRIC SCORE | RUNNING ES | CORE ENRICHMENT || 1 | PIK3CG | na | 1248 | 0.428 | -0.0187 | No |
| 2 | MAPK3 | na | 4918 | 0.100 | -0.1778 | No |
| 3 | SOCS1 | na | 5006 | 0.095 | -0.1732 | No |
| 4 | PKM | na | 5020 | 0.095 | -0.1653 | No |
| 5 | ADIPOQ | na | 5554 | 0.065 | -0.1838 | No |
| 6 | TNF | na | 5838 | 0.050 | -0.1923 | No |
| 7 | PRKCZ | na | 6115 | 0.036 | -0.2017 | No |
| 8 | CACNA1D | na | 6408 | 0.024 | -0.2129 | No |
| 9 | MAPK9 | na | 6752 | 0.011 | -0.2276 | No |
| 10 | CACNA1E | na | 6805 | 0.009 | -0.2292 | No |
| 11 | SOCS2 | na | 7627 | 0.000 | -0.2668 | No |
| 12 | GCK | na | 8093 | 0.000 | -0.2881 | No |
| 13 | KCNJ11 | na | 9082 | 0.000 | -0.3334 | No |
| 14 | PDX1 | na | 11944 | 0.000 | -0.4644 | No |
| 15 | IRS4 | na | 12166 | 0.000 | -0.4745 | No |
| 16 | ABCC8 | na | 13247 | 0.000 | -0.5240 | No |
| 17 | HK3 | na | 13324 | -0.002 | -0.5273 | No |
| 18 | PKLR | na | 13410 | -0.006 | -0.5307 | No |
| 19 | CACNA1A | na | 13593 | -0.010 | -0.5381 | No |
| 20 | CACNA1B | na | 13839 | -0.016 | -0.5479 | No |
| 21 | SLC2A2 | na | 14388 | -0.035 | -0.5699 | No |
| 22 | CACNA1G | na | 15491 | -0.097 | -0.6117 | No |
| 23 | PRKCD | na | 16045 | -0.131 | -0.6252 | Yes |
| 24 | MAPK10 | na | 16183 | -0.140 | -0.6190 | Yes |
| 25 | MAPK1 | na | 16234 | -0.143 | -0.6084 | Yes |
| 26 | IKBKB | na | 16790 | -0.182 | -0.6175 | Yes |
| 27 | SOCS3 | na | 16838 | -0.185 | -0.6031 | Yes |
| 28 | HK1 | na | 17572 | -0.235 | -0.6156 | Yes |
| 29 | MAPK8 | na | 17866 | -0.258 | -0.6058 | Yes |
| 30 | HK2 | na | 18028 | -0.271 | -0.5888 | Yes |
| 31 | PIK3CD | na | 18197 | -0.284 | -0.5710 | Yes |
| 32 | PIK3R2 | na | 18436 | -0.299 | -0.5550 | Yes |
| 33 | PIK3CA | na | 18513 | -0.305 | -0.5312 | Yes |
| 34 | PIK3R3 | na | 19336 | -0.380 | -0.5346 | Yes |
| 35 | SLC2A4 | na | 19402 | -0.388 | -0.5028 | Yes |
| 36 | PIK3R5 | na | 19509 | -0.397 | -0.4720 | Yes |
| 37 | SOCS4 | na | 20052 | -0.450 | -0.4564 | Yes |
| 38 | INSR | na | 20389 | -0.486 | -0.4281 | Yes |
| 39 | PIK3CB | na | 20535 | -0.507 | -0.3892 | Yes |
| 40 | CACNA1C | na | 20587 | -0.514 | -0.3454 | Yes |
| 41 | IRS1 | na | 20589 | -0.515 | -0.2993 | Yes |
| 42 | PRKCE | na | 21181 | -0.615 | -0.2711 | Yes |
| 43 | MTOR | na | 21338 | -0.653 | -0.2196 | Yes |
| 44 | IRS2 | na | 21348 | -0.656 | -0.1611 | Yes |
| 45 | PIK3R1 | na | 21419 | -0.674 | -0.1038 | Yes |
| 46 | MAFA | na | 21858 | -1.388 | 0.0008 | Yes |
Table: GSEA details [plain text format]

  

Fig 2: KEGG\_TYPE\_II\_DIABETES\_MELLITUS      
 Blue-Pink O' Gram in the Space of the Analyzed GeneSet

  

Fig 3: KEGG\_TYPE\_II\_DIABETES\_MELLITUS: Random ES distribution      
 Gene set null distribution of ES for **KEGG\_TYPE\_II\_DIABETES\_MELLITUS**
