# Supplementary material for: Regulation of microglia related neuroinflammation contributes to the protective effect of Gelsevirine on ischemic stroke
Source: Front Immunol. 2023 Mar 30;14:1164278. doi: 10.3389/fimmu.2023.1164278 (PMC10098192; doi:10.3389/fimmu.2023.1164278)
Supplement: Supplementary file 6 [file DataSheet_6.zip › fig 5 raw/fig 5-G raw/KEGG.Gsea.1649954846225/KEGG_TYROSINE_METABOLISM.html]

Details for gene set KEGG\_TYROSINE\_METABOLISM[GSEA]

|  || Dataset | OGD\_DRUG\_DRUG.OGD\_FRUG.cls#Gs\_versus\_MCAO.OGD\_FRUG.cls#Gs\_versus\_MCAO\_repos |
| Phenotype | OGD\_FRUG.cls#Gs\_versus\_MCAO\_repos |
| Upregulated in class | Gs |
| GeneSet | KEGG\_TYROSINE\_METABOLISM |
| Enrichment Score (ES) | 0.57860833 |
| Normalized Enrichment Score (NES) | 1.6245155 |
| Nominal p-value | 0.013623978 |
| FDR q-value | 0.03739672 |
| FWER p-Value | 0.298 |
Table: GSEA Results Summary

  

Fig 1: Enrichment plot: KEGG\_TYROSINE\_METABOLISM      
 Profile of the Running ES Score & Positions of GeneSet Members on the Rank Ordered List

  

| SYMBOL | TITLE | RANK IN GENE LIST | RANK METRIC SCORE | RUNNING ES | CORE ENRICHMENT || 1 | DDC | na | 947 | 0.471 | 0.0689 | Yes |
| 2 | ADH5 | na | 1254 | 0.428 | 0.1568 | Yes |
| 3 | METTL6 | na | 1516 | 0.398 | 0.2397 | Yes |
| 4 | ALDH3B2 | na | 2211 | 0.314 | 0.2829 | Yes |
| 5 | ALDH3B1 | na | 2884 | 0.251 | 0.3120 | Yes |
| 6 | MIF | na | 2907 | 0.250 | 0.3705 | Yes |
| 7 | GOT1 | na | 2969 | 0.244 | 0.4259 | Yes |
| 8 | FAH | na | 3570 | 0.194 | 0.4446 | Yes |
| 9 | LCMT1 | na | 3580 | 0.193 | 0.4902 | Yes |
| 10 | GSTZ1 | na | 3908 | 0.172 | 0.5163 | Yes |
| 11 | MAOA | na | 4152 | 0.153 | 0.5417 | Yes |
| 12 | TRMT11 | na | 4581 | 0.123 | 0.5515 | Yes |
| 13 | HGD | na | 5062 | 0.092 | 0.5516 | Yes |
| 14 | HEMK1 | na | 5162 | 0.086 | 0.5676 | Yes |
| 15 | AOC3 | na | 5543 | 0.066 | 0.5659 | Yes |
| 16 | ADH7 | na | 5804 | 0.052 | 0.5664 | Yes |
| 17 | IL4I1 | na | 5984 | 0.042 | 0.5682 | Yes |
| 18 | TAT | na | 6112 | 0.036 | 0.5709 | Yes |
| 19 | ALDH3A1 | na | 6135 | 0.035 | 0.5783 | Yes |
| 20 | AOC2 | na | 6281 | 0.029 | 0.5786 | Yes |
| 21 | DBH | na | 7531 | 0.000 | 0.5214 | No |
| 22 | DCT | na | 7538 | 0.000 | 0.5211 | No |
| 23 | TH | na | 7699 | 0.000 | 0.5138 | No |
| 24 | PNMT | na | 7791 | 0.000 | 0.5097 | No |
| 25 | ADH4 | na | 8140 | 0.000 | 0.4937 | No |
| 26 | LCMT2 | na | 8543 | 0.000 | 0.4753 | No |
| 27 | TPO | na | 10272 | 0.000 | 0.3962 | No |
| 28 | TYR | na | 10334 | 0.000 | 0.3934 | No |
| 29 | ALDH1A3 | na | 11926 | 0.000 | 0.3206 | No |
| 30 | AOX1 | na | 14290 | -0.031 | 0.2197 | No |
| 31 | TYRP1 | na | 14331 | -0.033 | 0.2257 | No |
| 32 | COMT | na | 14931 | -0.064 | 0.2136 | No |
| 33 | GOT2 | na | 15431 | -0.094 | 0.2131 | No |
| 34 | MAOB | na | 16182 | -0.140 | 0.2121 | No |
| 35 | HPD | na | 17120 | -0.204 | 0.2177 | No |
Table: GSEA details [plain text format]

  

Fig 2: KEGG\_TYROSINE\_METABOLISM      
 Blue-Pink O' Gram in the Space of the Analyzed GeneSet

  

Fig 3: KEGG\_TYROSINE\_METABOLISM: Random ES distribution      
 Gene set null distribution of ES for **KEGG\_TYROSINE\_METABOLISM**
